# Evolutionary History of the Toll-Like Receptor Gene Family across Vertebrates

**DOI:** 10.1093/gbe/evz266

**Published:** 2019-12-04

**Authors:** Guangshuai Liu, Huanxin Zhang, Chao Zhao, Honghai Zhang

**Affiliations:** 1 College of Life Science, Qufu Normal University, Shandong, China; 2 College of Marine Life Science, Ocean University of China, Qingdao, Shandong, China

**Keywords:** vertebrate immunity, Toll-like receptors, gene family evolution, phylogenetics, natural selection

## Abstract

Adaptation to a wide range of pathogenic environments is a major aspect of the ecological adaptations of vertebrates during evolution. Toll-like receptors (TLRs) are ancient membrane-bound sensors in animals and are best known for their roles in detecting and defense against invading pathogenic microorganisms. To understand the evolutionary history of the vertebrate TLR gene family, we first traced the origin of single-cysteine cluster TLRs that share the same protein architecture with vertebrate TLRs in early-branching animals and then analyzed all members of the TLR family in over 200 species covering all major vertebrate clades. Our results indicate that although the emergence of single-cysteine cluster TLRs predates the separation of bilaterians and cnidarians, most vertebrate TLR members originated shortly after vertebrate emergence. Phylogenetic analyses divided 1,726 vertebrate TLRs into 8 subfamilies, and TLR3 may represent the most ancient subfamily that emerged before the branching of deuterostomes. Our analysis reveals that purifying selection predominated in the evolution of all vertebrate TLRs, with mean d*N*/d*S* (*ω*) values ranging from 0.082 for *TLR21* in birds to 0.434 for *TLR11* in mammals. However, we did observe patterns of positive selection acting on specific codons (527 of 60,294 codons across all vertebrate TLRs, 8.7‰), which are significantly concentrated in ligand-binding extracellular domains and suggest host–pathogen coevolutionary interactions. Additionally, we found stronger positive selection acting on nonviral compared with viral TLRs, indicating the more essential nonredundant function of viral TLRs in host immunity. Taken together, our findings provide comprehensive insight into the complex evolutionary processes of the vertebrate TLR gene family, involving gene duplication, pseudogenization, purification, and positive selection.

## Introduction

Vertebrates comprise an extraordinary group that exhibits remarkable species richness and a wide range of ecological adaptations from aquatic to terrestrial habitats (Jetz and Fine 2012). Adaptation to the complex pathogenic environment is a major aspect of the ecological adaptations of vertebrates, imposing frequent evolutionary pressures on molecules that form direct interfaces between the host and pathogens, for example, pattern recognition receptors (Barreiro et al. 2009). Toll-like receptors (TLRs) belong to one of the most essential and functionally most characterized pattern recognition receptors playing a crucial role in innate immunity in vertebrates (Leulier and Lemaitre 2008). As TLRs are directly positioned at the host–environment interface and are potentially subject to coevolutionary dynamics with their pathogenic counterparts (Barreiro et al. 2009), they provide an excellent model for studying the natural selection caused by pathogenic microorganisms on the vertebrate genome. Additionally, vertebrate TLRs have been the focus of intense research, largely because of the link between TLR dysfunction and several diseases in humans (Mukherjee et al. 2019).

TLRs, named after the Toll protein first found in the fruit fly *Drosophila melanogaster*, are a group of evolutionarily ancient membrane-bound sensors localized to plasma membranes and endosomes (Anderson et al. 1985). In general, prototypical metazoan TLR proteins are structurally characterized by three major domains: a hydrophobic tandem leucine-rich repeat (LRR) extracellular domain (ECD) that mediates the recognition of pathogen-associated molecular patterns (PAMPs); a short transmembrane (TM) domain; and an intracellular Toll/interleukin-1 receptor (TIR) signaling domain (ICD) required for transmission of a signal to downstream pathway components (see supplementary fig. S1, Supplementary Material online) (Kawai and Akira 2010). Upon binding of PAMPs, human TLRs generally activate NF-κB signaling by recruiting cytoplasmic TIR domain-containing adaptor proteins such as MyD88 and TRIF. Eventually, MyD88- and TRIF-dependent pathways activate the transcription factors NF-κB, AP-1, and IRFs to elicit inflammatory and antipathogen responses (Lin et al. 2010). Although almost all human TLRs utilize the MyD88-dependent pathway, TLR3 activation stimulates the TRIF-dependent pathway to exert antiviral effects (Kawai and Akira 2010). It has been known for some time that TLRs and the TLR-to-NF-кB pathway components that they initiate are present in a wide variety of organisms, from many basal metazoans to mammals (Brennan and Gilmore 2018).

Genomic data from diverse organisms suggest that prototypical TLRs with three domains first appeared within the phylum Cnidaria (Wiens et al. 2007; Gauthier et al. 2010). TLRs can be classified based on the number of cysteine clusters in their ECDs: multiple cysteine cluster TLRs (mccTLRs) and single-cysteine cluster TLRs (sccTLRs). mccTLR ectodomains have two cysteine clusters at the C-terminus of the LRR (LRRCT), whereas sccTLR ECDs have only one LRRCT motif (supplementary fig. S1, Supplementary Material online). It has been suggested that mccTLRs are only present in more ancient protostome species, suggesting that this type may represent an ancestral domain structure of functional TLR proteins (Brennan and Gilmore 2018). All vertebrate TLRs and some invertebrate TLRs are sccTLRs (Leulier and Lemaitre 2008). Nevertheless, not all identified TLR proteins are prototypical TLRs, and some basal metazoans also express separate LRRs and TIR domain-only proteins defined as TLR-like proteins (Wiens et al. 2007; Gauthier et al. 2010; Simakov et al. 2013). As such, it is hypothesized that prototypical TLRs emerged from the fusion of LRR and TIR domain-only genes (Brennan and Gilmore 2018). TLR proteins also have roles in developmental processes across some basal metazoans. In particular, a recent study has revealed a conserved function for mccTLRs in axis elongation among arthropods (Benton et al. 2016).

Since the first report of Toll proteins in *D. melanogaster*, ten TLRs have been identified in humans (TLR1–10) (Barreiro et al. 2009). Although the set of human TLRs might constitute an immunological redundancy for recognizing PAMP ligands, the ten TLRs in humans are functionally partitioned into two subgroups depending on their cellular localization and ligands sensed. One group is composed of TLR1, TLR2, TLR4, TLR5, and TLR6, which are localized to cell membranes and predominantly recognize bacterial components such as lipids, lipoproteins, and proteins (nonviral TLRs). The other group is composed of TLR3, TLR7, TLR8, and TLR9, which are localized to endosomes, where they recognize microbial nucleic acids (viral TLRs) (Kawai and Akira 2010). Previous studies on primates (Barreiro et al. 2009; Wlasiuk and Nachman 2010) and carnivores (Liu et al. 2017) have reported that the selection landscape differs between viral and nonviral TLRs. Barreiro et al. (2009) concluded that this interesting dichotomy most likely reflects either the different PAMP nature of the pathogens recognized by the two sets of TLRs or the greater evolutionary flexibility of nonviral TLRs. Regardless, it remains unclear whether this intriguing selection landscape heterogeneity between nonviral and viral TLRs is a general pattern extending to the overall vertebrate group. Despite the evolved flexibility of nonviral TLRs compared with viral TLRs in certain vertebrate groups, the evolutionary patterns of TLRs have been reported as also constrained; this is presumably because of the need to maintain the well-established pathogenic sensing function (Wlasiuk and Nachman 2010; Alcaide and Edwards 2011; Babik et al. 2015; Liu et al. 2017). Nonetheless, the selection patterns of functionally conserved TLRs may be oversimplified, as evidenced by multiple instances of recurrent or episodic positive selection exerted on various taxonomic groups (Wlasiuk and Nachman 2010; Alcaide and Edwards 2011; Quach et al. 2013). Moreover, many TLR polymorphisms in humans have been associated with the divergence in susceptibility to pathogenic infection and resistance (Mukherjee et al. 2019).

In fact, several genome-wide studies to date have revealed a remarkable species-specific expansion or constriction of the TLR repertoire in many species, especially in invertebrates (Hibino et al. 2006; Huang et al. 2008; Tassia et al. 2017). With regard to vertebrates, only a single study has addressed the overall evolution of the vertebrate TLR gene family. Specifically, Roach et al. (2005) proposed that all vertebrate TLRs can be divided into six major subfamilies and that most vertebrates have exactly one gene ortholog for each TLR subfamily. However, these conclusions were based on a rather small species sampling (ten consisting of seven vertebrate, one urchin, and two ascidian species). Thus, the evolutionary history of TLR members across the vertebrate evolutionary history remains unclear, and such scientific issues are invaluable for understanding the ancestry and functional evolution of vertebrate TLRs and for identifying subsequent innovations in vertebrate-specific innate immunity. Here, we address current hypotheses and examine prior conclusions regarding vertebrate TLR gene family evolution. We also seek to illuminate the origin of the vertebrate TLR gene family within the context of TLR evolution in early-branching animals. Furthermore, through critical assessment of the selective pattern of all members of the TLR family in vertebrates, we describe the adaptive microevolutionary changes in these immune receptors to provide insight into coadaptation between the host and pathogens. This study is the most comprehensive comparative analysis to date of TLR family evolution across the most advanced metazoan subphylum Vertebrata.

## Materials and Methods

### Data and Query Sets

To explore the origin history of vertebratelike TLR genes, we examined the genomic data for 25 metazoan species (supplementary table S1, Supplementary Material online) via bioinformatics analyses. Genomic sequences of these species were retrieved from NCBI (https://www.ncbi.nlm.nih.gov/; last accessed June 20, 2019). Phylum/Subphylum taxa (Cnidaria, Nematomorpha, Annelida, Mollusca, Arthropoda, Echinodermata, Hemichordata, Cephalochordata, Urochordata, and Vertebrate) covered by these species were selected to represent the phylogenetic depth and distribution representative of all major Metazoa groups. For most studied phyla or subphyla in this study, more than one representative species were selected. To identify TLR homologs, BlastN searches (blast v2.6.0; Altschul et al. 1990) were performed using local databases constructed from the downloaded genomic sequences. For species in which TLR repertoires have been investigated in previous genome-wide studies, we retrieved all of these TLR sequences as queries. To enhance the comprehensiveness of our searches, the gene candidates were used as queries for additional rounds of BLAST searches against the genome of the same species to find any additional TLR genes. For species for which genome-wide studies of TLR repertoires have not been performed, we used the reported TLR sequences obtained by clone-sequencing and/or from transcriptomes as queries to search for TLR homologs in their respective genomes. The genomes used for analyses, the homologs identified, and their respective accession numbers are available in supplementary table S1, Supplementary Material online.

To define TLR subfamilies and demonstrate an evolutionary history for all vertebrate TLR members, we increased the number of vertebrate species. We conducted extensive BlastN searches of TLR genes against the genome sequences of 119 vertebrate species (supplementary table S2, Supplementary Material online: 24 mammals, 37 birds, 14 reptiles, 6 amphibians, and 38 fishes). TLR sequences were also mined from amphibian transcriptomes, as only six amphibian genomes are publicly available at present. Transcriptome sequencing data of 90 amphibian species were obtained from the SRA database (supplementary table S3, Supplementary Material online). De novo assemblies of the amphibian transcriptomes for each species were generated using two different assemblers, Trinity v2.4.0 (Haas et al. 2013) with the default parameters and SOAPdenovo-Trans v1.02 (Luo et al. 2012) with K31. The quality assessment of the assembled transcriptomes, including the total number, max length, and N50 of unigenes, is presented in supplementary table S3, Supplementary Material online. TLR genes from six well-assembled and annotated genomes (*Homo sapiens*, *Mus musculus*, *Gallus gallus*, *Chrysemys picta*, *Xenopus tropicalis*, and *Danio rerio*) were used as queries to search for TLR genes in vertebrate genomes and amphibian transcriptomes. The TLR sequences identified in transcriptomes assembled by both Trinity and SOAPdenovo-Trans methods were considered as candidates. Notably, the transcriptomic data sets of amphibians were assembled and used only for obtaining TLRs, and no conclusions were drawn regarding the absence of any particular TLR member. Additionally, TLR genes were identified in the genome of *Petromyzon marinus* using BLAST searches.

### Bioinformatic Retrieval of TLR Sequences

Preliminary phylogenetic analyses revealed some TLR genes to be highly divergent, with frequent species-specific gene birth and death. To ensure the completeness of the TLR gene family in species, a high BLAST *e*-value cutoff of 1e-3 was used to capture distant sequence homologs and avoid missing all true positives. Genomic regions surrounding hits were extracted and subjected to an online GeneWise analysis (https://www.ebi.ac.uk/Tools/psa/genewise/; Chojnacki et al. 2017) to predict the coding sequence and gene structure. The domain architectures of all TLR candidates were delineated using SMART (http://smart.embl-heidelberg.de/; Letunic and Bork 2018) and LRRfinder (http://www.lrrfinder.com/; Offord et al. 2010). Based on the domain architecture results, prototypical TLR proteins were determined by the presence of an ECD, a TM domain, and an ICD. sccTLRs were determined by the presence of only one LRRCT in the ECD and mccTLRs by the presence of two LRRCTs in the ECD, with one LRRCT interrupting the LRR region (Brennan and Gilmore 2018). For vertebrate TLRs, only full-length (FL) TLRs containing an intact ECD, TM domain, and ICD were retained for further analyses. Sequences with stop codons and lacking open reading frames for these domains were designated as “pseudogenes” and “partial sequences,” respectively. Finally, all putative TLR genes were verified and adjusted for gene nomenclature by conducting BLAST searches against the NCBI nr protein database as well as phylogenetic analyses.

### Multiple Alignments and Phylogenetic Analyses

To avoid the biasing effect of rapid evolution on the third codon positions on highly divergent TLR sequences retrieved from a broad range of taxa, amino acid sequences were used instead of nucleotide sequences to perform the phylogenetic analysis (Velova et al. 2018). Multiple alignments of TLR protein sequences were obtained using MUSCLE v3.6 (Edgar 2004) and then processed using SeaView v4 (Gouy et al. 2010). PartitionFinder2 v2.1.1 (Lanfear et al. 2017) was employed to select the most appropriate model of amino acid evolution through Akaike Information Criterion (Akaike 1974). For each alignment data set, best-fit evolutionary models were estimated and used separately for reconstructed phylogenetic trees; they are listed in supplementary table S8, Supplementary Material online. To explore the origin history of vertebratelike TLR genes, phylogenetic relationships of prototypical TLRs in 25 metazoan species were reconstructed using the Bayesian Inference approach with MrBayes v3.2.7 (Altekar et al. 2004). The Bayesian Inference analysis was performed with chains sampled every 2,000 generations (after 25% of trees were discarded as burn-in), with random starting trees, and with three hot and one cold chain; convergence was confirmed when the standard deviation of split frequencies was <0.01 (corresponding generation when the standard deviation of split frequencies fell <0.01 was 1,096,000). To define TLR subfamilies and demonstrate the evolutionary history of all vertebrate TLR members, a vertebrate TLR tree was reconstructed using the maximum likelihood (ML) method in RAxML HPC-PTHREADS v8.2.11 (Stamatakis 2006) with 1,000 bootstrapping replications. For the vertebrate TLR gene tree, RAxML was preferred over MrBayes because of its accessibility when working with large data set and short computation time. Graphical presentation of the phylogenies was achieved using iTOL (Letunic and Bork 2007). Percent identity for each pair of TLR amino acid sequences was calculated using the MegAlign in DNASTAR program package (DNASTAR Inc., Madison, WI). All created TLR protein alignments are shown in supplementary texts S1 and S2, Supplementary Material online.

### Estimating the Modes and Strength of Natural Selection for Vertebrate TLRs

We used selection test analyses to describe the evolutionary landscape of TLR genes across vertebrate phylogenies. For evaluation of the evolutionary landscape for each vertebrate TLR gene, the amino acid alignments generated by MUSCLE were converted into codon alignments with PAL2NAL v14 (Suyama et al. 2006). Alignments were processed using Gblocks v0.91b (Talavera and Castresana 2007) at various stringency settings to remove regions involving gaps or poorly aligned regions. However, this processing did not impact our estimation of selective pressures because the vast majority of each alignment was retained (77.7–99.0% of the positions retained in the alignments for all vertebrate TLRs; supplementary table S4, Supplementary Material online); in addition, the number of sequences obtained was not small for each of the TLR alignments.

We used the codeml method implemented in the PAML v4.7 package (Yang 2007) to estimate overall *ω* values (d*N*/d*S*; d*N*, number of nonsynonymous substitutions per nonsynonymous site and d*S*, number of synonymous substitutions per synonymous site) for each TLR gene; *ω* ratios were also estimated separately for ECD and ICD for each TLR gene by codeml method. For TLRs in each vertebrate taxon and overall vertebrates, paired Wilcoxon test of null hypotheses that the strength of selective pressures is imposed evenly among the full length of gene, the ECD and the ICD were conducted. To decrease the possibility of false positive results, we applied the false discovery rate (FDR) test to correct for multiple comparisons (Benjamini et al. 2001). We considered an FDR-adjusted *P* value (*q* value) < 0.05 as significant.

The selection landscape has been reported to differ between viral- and nonviral-sensing TLRs in primates (Barreiro et al. 2009; Wlasiuk and Nachman 2010) and carnivores (Liu et al. 2017). To test whether this hypothesis can extend to all vertebrate TLRs, we first classified vertebrate TLRs into two groups according to the type of ligand they detected. The following are nonviral TLRs: TLR1/2/6/10 ( di- or tri-acylated lipopeptides; Ozinsky et al. 2000; Chuang and Ulevitch 2001; Travassos et al. 2004); TLR4 (bacterial lipopolysaccharide [LPS]; Kim et al. 2007); TLR5 (bacterial flagellin; Hayashi et al. 2001); TLR5S (bacterial flagellin; Tsukada et al. 2005); TLR11 (protozoan profilinlike protein; Yarovinsky et al. 2005); TLR12 ( protozoan profilinlike protein; Koblansky et al. 2013; Raetz et al. 2013); TLR18 (bacterial pathogens; Shan et al. 2018); and TLR20 (protozoan profilinlike protein; Pietretti et al. 2014). Viral TLRs are as follows: TLR3 (double-stranded RNA; Alexopoulou et al. 2001); TLR7 (single-stranded RNA; Diebold et al. 2004; Heil et al. 2004); TLR8 (single-stranded RNA; Heil et al. 2004); TLR9 (bacterial DNA; Hemmi et al. 2000); TLR13 (bacterial 23S ribosomal RNA; Li and Chen 2012; Oldenburg et al. 2012); TLR19 (double-stranded RNA; Ji, Rao, et al. 2018); TLR21 (CpG oligodeoxynucleotides; Brownlie et al. 2009; Yeh et al. 2017); and TLR22 (double-stranded RNA; Ji, Ramos-Vicente, et al. 2018). The following are TLRs that sensing both viral and nonviral ligands: TLR14 (hemorrhagic septicemia virus, *Streptococcus iniae* and *Edwardsiella tarda*; Hwang et al. 2011); TLR15 (RNA viruses and lysates from yeast; Boyd et al. 2012; Chen et al. 2013; Heidari et al. 2015); and TLR25 (bacterial LPS and double-stranded RNA; Li et al. 2018). The ligands for TLR5L and TLR23 remain unknown, and we did not assign a viral/nonviral recognition status. A Wilcoxon test was then applied to assess significance between group distributions for *ω* values. A *P* value < 0.05 was considered as significant.

### Codon-Based Analyses of Positive Selection

Evolutionary analyses can be affected by recombination events, hence the existence of putative recombination breakpoints within each vertebrate TLR alignment was first analyzed using the Genetic Algorithm for Recombination Detection tool on the Datamonkey server (http://classic.datamonkey.org/; Pond et al. 2005). The significance of the recombination breakpoints was verified with the KH post-test. We tested for evidence of positive selection acting on individual residues in TLRs using PAML (Yang 2007) and Fast Unconstrained Bayesian AppRoximation (FUBAR; Murrell et al. 2013). These two programs provide independent estimates of positive selection. In PAML, codon-based substitution models (codeml) using comparison of neutral or purifying selection M7 (*ω* ≤ 1) with positive selection M8 (*ω* > 1) models were adopted. The two nested models were compared using likelihood ratio tests (Nielsen and Yang 1998) to examine whether model M8 fits better than model M7, and the Bayes empirical Bayes approach was employed to determine site-specific posterior probabilities of positive selection (>0.95) (Yang et al. 2005). FUBAR analysis was performed on the Datamonkey server (http://classic.datamonkey.org/; Pond et al. 2005) with the significance level of default posterior probability at 0.9. The FUBAR algorithm was used because it is more robust and faster than existing unconstrained fixed-effect likelihood methods (Murrell et al. 2013). The position numbering of positively selected sites (PSSs) follows the TLR sequences of representative species. The amino acid ranges of ECD and ICD for each TLR identified by SMART are provided in supplementary table S6, Supplementary Material online. We also assessed whether PSSs are disproportionately distributed among functional bipartitions of vertebrate TLR genes. Chi-square tests of null hypotheses that PSSs occur evenly among the FL, ECD, and ICD of TLRs in each vertebrate taxon and overall vertebrates were conducted. We then performed multiple testing on the *P* values using the FDR correction (Benjamini et al. 2001). A *q* value < 0.05 was considered as significant. A chi-square test was also performed to evaluate the significance between viral and nonviral TLR distributions for PSSs. In this comparison, a *P* value < 0.05 was considered as significant.

Evidence of positive selection at individual sites of orthologous TLR genes (*TLR3*, *TLR4*, *TLR5*, and *TLR7*) shared by all vertebrate groups along the ancestral branch of each vertebrate group was further detected by using the branch-site likelihood method implemented in PAML v4.7 (Yang 2007). Branch-site model analyses were conducted using the MA model (model = 2, NSsite = 2, fix omega = 0) and the MA null model (model = 2, NSsite = 2, fix omega = 1) (Zhang et al. 2005). Likelihood ratio tests were used to compare the two models (Nielsen and Yang 1998). By using the Bayes empirical Bayes approach, the sites with a posterior probability > 0.95 were considered candidates for significant selection (Yang et al. 2005).

### Assessing Function of Positively Selected Sites

Because abundant functionally important positions have been reported for human TLRs, we can assess how PSSs identified in mammalian TLRs may influence their structure and function using human TLR genes as references. We compared the mammalian PSSs identified in this study with the results of previous studies focusing on mammalian TLR evolution and identifying positive selection at the interspecific level (Wlasiuk and Nachman 2010; Areal et al. 2011; Shen et al. 2012; Liu et al. 2017) and studies that describe LBSs for human TLR1 (Jin et al. 2007), TLR2 (Jin et al. 2007), TLR3 (Bell et al. 2006; Liu et al. 2008; Wang et al. 2010), TLR4 (Park et al. 2009), TLR5 (Andersen-Nissen et al. 2007; Song et al. 2017), TLR7 (Wei et al. 2009; Zhang et al. 2016), TLR8 (Wei et al. 2009; Tanji et al. 2015), and TLR9 (Wei et al. 2009; Ohto et al. 2015). To gain insight into how substitutions at PSSs may influence the structure and function of TLR proteins, I-TASSER Suite 5.1 (https://zhanglab.ccmb.med.umich.edu/I-TASSER/; Yang et al. 2015) was utilized to predict 3D structures of vertebrate reference TLRs (human TLR1-10 and mouse TLR11-13). We used PyMOL v2.3.1 (https://www.pymol.org/) for graphical visualization of PSSs and distance measurement of any PSSs detected in our study from LBSs for the predicted 3D structural models of TLRs. PSSs identified in our study within a distance of 5 Å of LBSs were considered to be closely connected to the LBSs, that is, having a potential influence on receptor function (Velova et al. 2018). LBSs were also predicted using PyMOL for human TLR6 and TLR10 and mouse TLR11-13, for which LBSs have not been functionally determined.

## Results and Discussion

### Evolutionary Origin of Prototypical TLR Genes

**Fig. 1.**
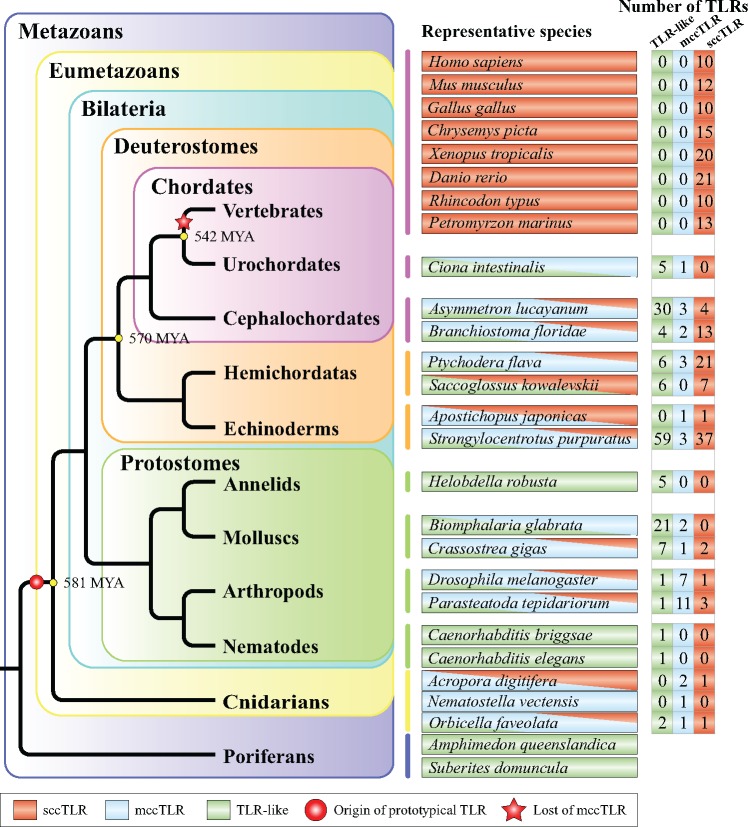
—Origin and distribution of different TLR types (sccTLRs, mccTLRs, and TLR-like) in the main metazoan lineages. A simplified phylogenic tree adapted from a recent study (Simakov et al. 2015) depicting the general relationship of the major metazoan phyla and subphyla. The numbers on particular nodes indicating calibration dates (Ma) were adopted from Simakov et al. (2015). This figure highlights the origin of prototypical TLRs, the loss of mccTLRs, and the gene numbers of different TLR types in representative species. The numbers of TLR-like, mccTLR, and sccTLR genes identified in each representative species are shown in right-hand columns.

In general, vertebrates express ∼10 TLRs, and all of them are prototypical TLRs, specifically sccTLRs (Leulier and Lemaitre 2008). Therefore, it is necessary to understand the evolutionary origin of prototypical TLR genes before we discuss the evolutionary history of the vertebrate TLR gene family. In particular, we tested a hypothesis concerning the origin of mccTLRs and sccTLRs in this section. Using a homology-based gene prediction method (see Materials and Methods), we identified TLR repertoires in the genomes of 25 species covering all major metazoan groups (fig. 1 and supplementary table S1, Supplementary Material online). Overall, we found that mccTLRs or sccTLRs are widespread in most of the studied invertebrate phyla, although they appear to be absent in the studied species of nematodes and annelids. Although the complete genomes of several poriferan species have been sequenced, no prototypical TLRs have been identified in any member of the phylum Porifera (Wiens et al. 2007; Gauthier et al. 2010; Hentschel et al. 2012). Moreover, genomic data suggest that TLR genes are absent in nonanimal phyla (Leulier and Lemaitre 2008). Together, these data support the hypothesis proposed in a previous study that prototypical TLRs originated in the eumetazoan ancestor more than 581 Ma, before the separation of bilaterians and cnidarians (Leulier and Lemaitre 2008). Our data also showed the absence of mccTLRs in all vertebrate lineages, only being identified in invertebrate species and suggesting that the loss of mccTLRs predates the emergence of present-day vertebrate main lineages 542 Ma (fig. 1).

**Fig. 2.**
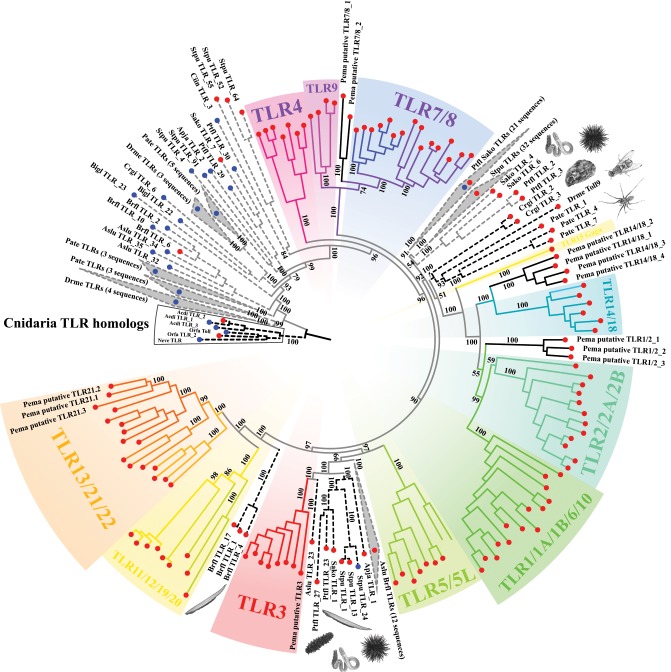
—Prototypical TLR trees for main eumetazoan lineages, providing insight into the origin of vertebrate TLR genes. Bayesian analysis was performed using FL prototypical sccTLR and mccTLR amino acid sequences from selected representative species in phyla or subphyla from Cnidaria to Vertebrata (fig. 1 and supplementary table S1, Supplementary Material online). Phylogeny was rooted with the TLRs of cnidarian species. The numbers at branches indicate the percent of posterior probability values. Dashed lines denote invertebrate prototypical TLRs, and solid lines denote vertebrate prototypical TLRs. Red circles indicate sccTLRs, and blue circles indicate mccTLRs. Major vertebrate TLR members are color coded and labeled.

A recent study hypothesized that mccTLRs and sccTLRs emerged in the phyla Cnidaria and Mollusca, respectively (Brennan and Gilmore 2018). Here, we show that although two TLRs in species of Cnidaria (Acdi TLR_2 and Orfa TLR_2) are clustered with cnidarian mccTLRs (see fig. 2), they possess the complete domain structure of sccTLRs (i.e., comprising an ECD with only one LRRCT motif, a TM, and an ICD; supplementary table S1, Supplementary Material online). Moreover, analysis of the amino acid sequences of the two sccTLRs reveals that they have a much shorter ECD (two LRRs and one LRR, respectively) compared with the mccTLRs in cnidarian species (3–12 LRRs; supplementary table S1, Supplementary Material online), which indicates that cnidarian sccTLRs may have evolved different functional mechanisms of pathogenic recognition from those of mccTLRs. Although cnidarian sccTLRs cluster with cnidarian mccTLRs, they are structurally mature sccTLRs. The above findings together indicated that mccTLRs and sccTLRs may have emerged synchronously during the evolution of Cnidaria. Nevertheless, considering the substantial difference in the number of LRRs between cnidarian sccTLRs and vertebrate sccTLRs, further functional studies should address whether cnidarian sccTLRs possess similar pathogenic recognizing functions as vertebrate sccTLRs.

Evidence for TLR expansions has been reported for the echinoderm *Strongylocentrotus purpuratus* (Hibino et al. 2006; Tassia et al. 2017) and the hemichordate *Ptychodera flava* (Tassia et al. 2017). Furthermore, we found that the numbers and types of TLR genes vary greatly in species among different phyla/subphyla groups or within the same phylum/subphylum group (fig. 1 and supplementary table S1, Supplementary Material online). For instance, two nematodes, *Caenorhabditis elegans* and *Caenorhabditisbriggsae*, both harbor only one TLR-like gene; in contrast, the echinoderms *S. purpuratus* possess a substantial expanded repertoire of 99 TLR members (59 TLR-like genes, 3 mccTLRs, and 37 sccTLRs). However, evidence for TLR expansion was not detected in the genome of other echinoderms, such as the sea cucumber *Apostichopus japonicas*, with only two TLR genes found in its genome. Although it has yet to be determined how these variations in TLR repertories affect the breadth of pathogen recognition, it is difficult to associate TLR repertories with the pathogenic resistance capacity of species. Further functional investigations are required to determine differences in such ability for different invertebrate groups.

### Evolutionary Origin of Vertebrate TLR Genes

Subsequently, we performed phylogenetic analyses utilizing FL prototypical TLRs in representative species of main metazoan lineages to gain insight into the origin of vertebrate TLR genes. The species included were selected to represent the phylogenetic depth and distribution of all major eumetazoan groups while covering all classes of vertebrates. According to this phylogenetic analysis of prototypical TLRs, TLR genes generally clustered based on their ECD subtype, that is, sccTLRs and mccTLRs tended to cluster together separately (fig. 2). Although the emergence of sccTLRs predates the evolution of bilaterians, it is difficult to establish an orthologous relationship with the TLRs of invertebrates for most vertebrate TLR genes. This indicates that most vertebrate TLRs originated shortly after the emergence of vertebrates and rapidly diversified into various TLR genes.

Curiously, however, several vertebrate TLR members clustered into specific clades with TLR homologs from some basal eumetazoans (fig. 2). The phylogenetic analysis clustered vertebrate *TLR3* genes into a TLR3-specific clade and with prototypical TLR genes from species of echinoderms (*Apostichopusjaponicas* and *S. purpuratus*), hemichordates (*Ptychoderaflava* and *Saccoglossus kowalevskii*) and cephalochordates (*Asymmetron lucayanum*). Given the strong phylogenetic support (100% posterior probability) for *TLR3* homologs among invertebrate deuterostomes and the conserved evolutionary pattern of *TLR3* following the expected phylogenetic topologies (fig. 2), these sequences are likely orthologous. It is worth mentioning that there are no *TLR3* orthologs in tunicate species, possibly because these taxa underwent apparent TLR reduction (supplementary table S1, Supplementary Material online; Tassia et al. 2017). In the phylogenetic tree, this *TLR3* clade includes sequences as ancient as those from the phylum Echinodermata, suggesting that *TLR3* genes may have emerged before the branching of deuterostomes more than 570 Ma (Simakov et al. 2015). Analysis of vertebrate TLR3 amino acid sequences revealed more LRRs than in other vertebrate TLR members, with lengths comparable to most mccTLRs in invertebrate genomes (supplementary table S1, Supplementary Material online). Interestingly, a *S. purpuratus* mccTLR (Stpu TLR_24) clustered together with this TLR3-specific group. As such, we hypothesize that vertebrate *TLR3* is likely the result of loss of an LRRCT domain in certain ancestral mccTLR homologs. Vertebrate *TLR3* was originally identified as recognizing dsRNA (double-stranded RNA) derived from viruses or virus-infected cells (Alexopoulou et al. 2001). Furthermore, genome-wide analyses have suggested that the main components (such as NF-κB, TRAF, and IRF) of vertebrate TLR-to-NF-κB signaling and type-I IFN signaling involved in antiviral immune responses triggered by TLR3 are also present in basal deuterostomes, such as *S. purpuratus* and *Saccoglossuskowalevskii* (Hibino et al. 2006; Tassia et al. 2017). Together, our observations further support the hypothesis that TLR3-mediated antiviral immune responses may represent an ancient, evolutionarily conserved immune molecular mechanism shared across deuterostomes (Tassia et al. 2017).

Our tree interspersed amphioxi *Asymmetronlucayanum* and *Branchiostoma floridae* TLR sequences among deuterostome TLRs (fig. 2). However, we obtained a single clade including three amphioxus sccTLR genes (Brfl TLR_1, TLR_4, and TLR_17) clustered with the vertebrate *TLR11*/*12*/*19*/*20* and TLR*13*/*21*/*22* clades, indicating that these vertebrate TLR genes likely diversified after the divergence of vertebrates from the subphylum Cephalochordata. In addition, our phylogenetic analysis clustered a group of molluscan and arthropodan TLRs (Crgi TLR_2, TLR_3, Pate TLR_1, TLR_4, TLR_7, and Drme Toll9) with a large vertebrate clade including *TLR1*/*1A*/*1B*/*6*/*10*, *TLR2*/*2A*/*2B*, *TLR14*/*18*, and *TLR15* genes (fig. 2), which were named the TLR1 subfamily in a previous study (Roach et al. 2005). However, this result may simply reflect errors in the phylogenetic tree, and the divergence time among vertebrate *TLR1*/*1A*/*1B*/*6*/*10*, *TLR2*/*2A*/*2B*, *TLR14*/*18*, and *TLR15* clades cannot be determined at present, as the posterior probability for the key node was low (51% posterior probability). Among these invertebrate TLR genes grouped with the large vertebrate clade, Drme Toll9 is the only sccTLR gene in the *D. melanogaster* genome (supplementary table S1, Supplementary Material online). Structural and phylogenetic divergence from other *D. melanogaster* mccTLRs suggests functional divergence of Drme Toll9 from other mccTLRs. Therefore, the evolution of Drme Toll9 provides a striking example of an arthropod TLR that may have diverged during arthropod TLR gene expansion. Further functional studies will be required to determine the specificity of pathogenic recognition by the Drme Toll9 protein as well as its downstream immune signaling proteins.

We are conscious of the fact that although we have attempted to be as exhaustive as possible, additional TLR homologs or complete sequences might be discovered in ongoing high-quality genome assemblies. Furthermore, it should be noted that we sampled only one species for both the Annelida and Urochordata phyla. The robustness of related conclusions may be hindered by this. Nonetheless, our choice of species from various animal lineages allows the study of the evolution of TLR genes to be studied at the whole-metazoan clade scale. For most studied phyla/subphyla, we were able to study TLR genes from more than one species. Therefore, our data and analysis are sufficient for obtaining a qualitatively accurate assessment of the origin and evolutionary history of the TLR gene family in vertebrates.

### Classification of Vertebrate TLR Subfamilies

**Fig. 3.**
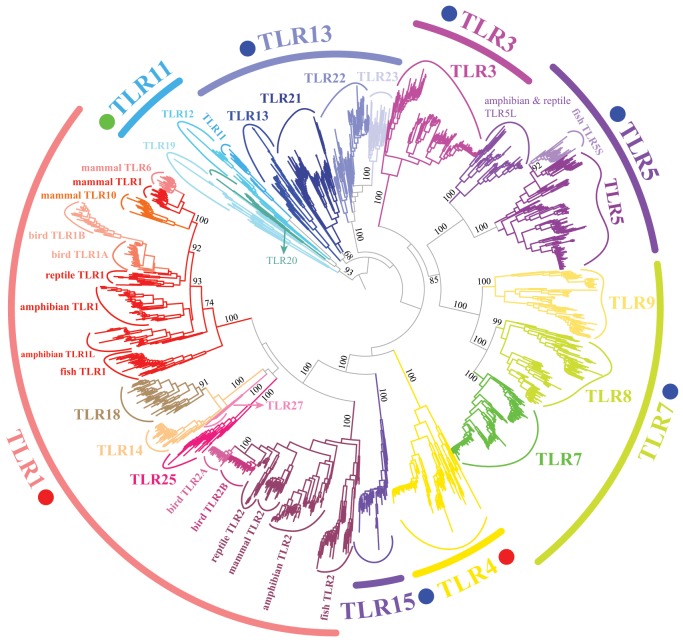
—Reconstruction of the phylogeny of vertebrate TLR genes, illustrating the division of the gene family into eight subfamilies. The unrooted ML tree was built on the basis of 1,726 TLR proteins identified from the genomes and transcriptomes of over 200 vertebrate species (see Materials and Methods). The numbers associated with internal branches denote bootstrap support values for the ML reconstruction. Subfamilies are named by the lowest ordinal TLR contained in that subfamily and shown in a unique color. The ECD architecture of each subfamily was labeled according to a previous study (Wang et al. 2016). Red, green, and blue are used to indicate three-domain, trans-three-domain, and single-domain ECD architectures for TLR subfamilies, respectively.

We next performed phylogenetic analyses to evaluate orthologous and paralogous relationships among vertebrate TLRs and to assess whether we can define TLR subfamilies. Notably, the availability of complete genome sequences and transcriptomic data from many vertebrates provides an opportunity to refresh early knowledge of the subfamily classification of vertebrate TLR members. We performed a BLAST search of TLR genes against the whole-genome assemblies and assembled transcriptomes of over 200 vertebrate species covering all major vertebrate clades. Supplementary tables S2 and S3, Supplementary Material online, list the sources of the genomic and transcriptomic data used and the taxonomic information about the studied species. In total, 1,726 intact vertebrate TLR sequences were identified. The number of TLR genes found per species ranged from 5 in the birds *Merops nubicus* and *Podiceps cristatus* to 26 in the fish *Cyprinus carpio* (supplementary table S2, Supplementary Material online). We employed the ML approach for the identified vertebrate TLRs when building the tree, and our TLR phylogenetic tree categorized all TLRs into eight subfamilies. These subfamilies are referred to as TLR1 (*TLR1*/*1A*/*1B*/*2*/*2A*/*2B*/*6*/*10*/*14*/*18*/*25*/*27*), TLR3, TLR4, TLR5 (*TLR5*/*5S*/*5L*), TLR7 (*TLR7*/*8*/*9*), TLR11 (*TLR11*/*12*/*19*/*20*), TLR13 (*TLR13*/*21*/*22*/*23*), and TLR15 subfamilies (defined by the lowest ordinal TLR contained in that subfamily; fig. 3). However, this is at odds with a previous study of the evolution of the vertebrate TLR gene family, which concluded that six major subfamilies (TLR1, TLR3, TLR4, TLR5, TLR7, and TLR11) of vertebrate TLRs exist and that most vertebrates have exactly one gene ortholog for each TLR family (Roach et al. 2005). The discrepancy between the studies is due to our inclusion of a large number of vertebrate species, which is in contrast to the few species included in the previous study. For example, our results exclusively identified the TLR15 subfamily in almost all birds and reptiles, with ECD architectures that differ from those of the neighboring TLR1 and TLR4 subfamilies in the phylogenetic tree (fig. 3). In contrast, the previous study included only one chicken *TLR15* and considered that *TLR15* may have derived from the TLR1 subfamily. Another discrepancy is the definition of the TLR13 subfamily. In our study, TLR13 was treated as a subfamily separate from that of TLR11 because these two subfamilies have different ECD architectures, which was consistent with a recent study (Wang et al. 2016). The interpretation of our phylogenetic results is notable on one count. One genomic cluster consisting of *TLR1*/*6*/*10* is located in close proximity in most mammalian clades (concluded from our BLAST analysis). *TLR2* is also relatively close to these three TLRs in mammalian genomes. Due to their close genomic proximity, it would be expected that the genomic proximity and evolutionary relationship analyzed here are not independent, which may have an effect on the outcome of the phylogenetic analysis. However, the close genomic proximity of these TLR genes was not considered in our phylogenetic analysis, and we speculate that this situation was also true for the cluster consisting of the *TLR7*/*8* genes.

With covering the gene members of *TLR1*/*1A*/*1B*/*2*/*2A*/*2B*/*6*/*10*/*14*/*18*/*25*/*27*, subfamily TLR1 is the one which includes the most TLR members within all eight subfamilies. TLR1 is also the largest TLR subfamily, comprising approximately one-third of the total number of vertebrate TLRs evaluated in the present study. Moreover, subfamily TLR1 is highly variable with regard to repertoire among different vertebrate taxa due to extensive gene gain and loss (such as *TLR18*, *TLR25*, and *TLR27* in fishes; *TLR1A*, *TLR1B*, *TLR2A*, and *TLR2B* in birds; *TLR6* and *TLR10* in mammals), suggesting more class-specific adaptations than for other subfamilies. *TLR14* is also found in nearly all species of amphibians and reptiles (supplementary table S2, Supplementary Material online), indicating that this gene originated 365 Ma after the divergence of amphibians from fishes (Carroll 2009). With regard to the phylogenetic relationship among the gene members in subfamily TLR1, there is a discrepancy between our results and the previous report from Velova et al. (2018). In our tree, mammalian *TLR1*, *TLR6*, and *TLR10* were clustered together, avian *TLR1A* and *TLR1B* were clustered together, and avian *TLR2A* and *TLR2B* were clustered together (fig. 3), suggesting that most members of the TLR1 subfamily in birds and mammals arose independently. However, in the results from Velova et al. (2018), avian *TLR1A* was clustered with mammalian *TLR10*, and avian *TLR1B* was clustered with reptile *TLR1*. We speculate that this discrepancy can be attributed to the difference in TLR data sets used for phylogenetic analysis. In the present study, the FL TLR amino acid sequences of all TLR gene members were used for the tree construction (fig. 3). However, in the study by Velova et al. (2018), a shorter, nonconverted region of *TLR1* gene members was identified and used for the tree construction. Future research on the genomic organization of *TLR1* gene members covering all major vertebrate clades is probably the key to illustrate the evolutionary relationship among vertebrate *TLR1* gene members.

*TLR3* is the only member of the TLR3 subfamily. Although two rounds of whole-genome duplication have occurred at the base of vertebrate radiation (Dehal et al. 2005), *TLR3* exists as a single gene in the genomes of all vertebrate species, with no pseudogenization, loss, or duplication (supplementary table S2, Supplementary Material online). This was also confirmed by the syntenic relationships of *TLR3* in five representative vertebrate species (supplementary fig. S2, Supplementary Material online). Furthermore, the phylogenetic analysis of vertebrate TLR3 can well recapitulate the phylogeny of vertebrate evolution (fig. 2). Hence, TLR3 is the most conserved TLR subfamily within vertebrates, indicating a fundamental role in the vertebrate antipathogen response.

Similarly, subfamily TLR4 contains only the *TLR4* gene, which originated before jawed vertebrates (supplementary fig. S3, Supplementary Material online). By forming a heterodimer with MD-2, TLR4 recognizes structurally diverse LPS molecules on the cell surface (Park et al. 2009). *TLR4* is extremely conserved as a single gene copy in most species genome, though there is evidence for extensive loss of this gene in the genomes of fishes and amphibians (supplementary table S2, Supplementary Material online). Although the arrangement of the *TLR4* surrounding genes is altered in zebrafish, the genomic organization for *TLR4* is very conserved in three amniotic species (supplementary fig. S2, Supplementary Material online). Additionally, some teleosts possess multiple copies of *TLR4*, such as four gene copies in *Astyanax mexicanus* and *Cyprinus carpio* and three in *Danio rerio*. Percent identities for paired *TLR4* amino acid sequences within the same species were 90.8–95.5% for *Astyanaxmexicanus*, 69.5–88.1% for *Cyprinus carpio*, and 64.7–80.3% for *Danio rerio*. The wide range of paired percent identities for *TLR4* paralogs may suggest different evolutionary histories of these paralogs within the species.

Subfamily TLR5 includes three members: *TLR5*, *TLR5S*, and *TLR5L*. Recent studies have shown that *TLR5* pseudogenization occurred independently several times along certain linages in birds (Bainova et al. 2014; Velova et al. 2018) (see also supplementary table S2, Supplementary Material online), though such events were not limited to birds. In this study, we found the fish *Amphiprion ocellaris* and reptile *Sphenodon punctatus* to harbor nonfunctional *TLR5* pseudogenes with stop codons in their sequences. Soluble short forms of *TLR5*, that is, *TLR5S* and *TLR5L*, are present in fishes, amphibians, and reptiles (supplementary tables S2 and S3, Supplementary Material online). Both fish *TLR5S* and amphibian/reptile *TLR5L* receptors lack TM domain and ICD and possess similar protein structures. This finding raises an interesting problem regarding whether these two analogs are orthologous genes or arose independently. Our phylogenetic analysis demonstrated that fish *TLR5S* were grouped together with vertebrate *TLR5* but was determined to be phylogenetically distant from amphibian/reptile *TLR5L* (fig. 3). Moreover, fish *TLR5S* and fish *TLR5* share 37.7–90.7% identity with each other, which is much higher than that between fish *TLR5S* and amphibian *TLR5L* (30.1–39.1%) and between fish *TLR5S* and reptile *TLR5L* (30.3–37.5%). Based on the phylogenetic results, we hypothesize that *TLR5S* and *TLR5L* may have arisen independently via duplication of the ECD of *TLR5* in the evolutionary history of fish and amphibian taxa. *TLR5* is recognized as a duplicated gene in zebrafish; however, the arrangement of the *TLR5* surrounding genes is somewhat conserved among the five vertebrate species (supplementary fig. S2, Supplementary Material online).

The three members of the TLR7 subfamily (*TLR7*, *TLR8*, and *TLR9*) mapped to the root of the vertebrate tree (fig. 3 and supplementary fig. S3, Supplementary Material online), indicating that the divergence of the TLR7 subfamily occurred before the divergence of fishes. Interestingly, *TLR8* and *TLR9* have been lost from all species of birds. However, consistent with previous studies (Grueber et al. 2012; Velova et al. 2018), we found that the *TLR7* gene appears to have recently duplicated in some bird species, such as in *Cuculus canorus*, *Mesitornis unicolor*, and *Taeniopygia guttata*. Although these three genes have been proposed to be redundant against most common viruses (Hemmi et al. 2000; Diebold et al. 2004; Heil et al. 2004), avian *TLR7* are specifically involved in the recognition of a specific highly pathogenic influenza virus (HPAIV) (Chen et al. 2013), which is a significant threat to avian health (Sutton 2018). The critical immunological role played by avian *TLR7* in defending against such highly pathogenic viruses provides a possible explanation for the independent duplication of this gene in recent avian history (Velova et al. 2018). In particular, avian *TLR7* possesses the highest d*N*/d*S* value and the highest accumulation of positively selected sites among vertebrate *TLR7* genes (see table 1). However, the immunological implications of the loss of *TLR8* and *TLR9* genes in the ancestor of modern birds require further study.

**Table 1:** Selection Analysis of Vertebrate TLRs

Taxa	Gene	Recognition Status^a^	Sequences Number^b^	aa Length^c^	d*N*/d*S*^d^	PSS^e^	PSS/Ref.^f^ (‰)
Fish	*TLR1*	Nonviral	34	795	0.248	4	5.0
	*TLR2*	Nonviral	40	788	0.257	4	5.1
	*TLR3*	Viral	35	903	0.217	2	2.2
	*TLR4*	Nonviral	15	816	0.306	2	2.5
	*TLR5*	Nonviral	32	881	0.259	6	6.8
	*TLR5S*	Nonviral	21	641	0.264	4	6.2
	*TLR7*	Viral	39	1,036	0.144	8	7.7
	*TLR8*	Viral	49	1,019	0.250	7	6.9
	*TLR9*	Viral	37	1,057	0.216	5	4.7
	*TLR13*	—	4	—	—	—	—
	*TLR18*	Nonviral	38	854	0.128	0	0
	*TLR19*	Viral	8	958	0.189	1	1.0
	*TLR20*	Nonviral	9	950	0.379	16	16.8
	*TLR21*	Viral	38	989	0.236	4	4.0
	*TLR22*	Viral	49	947	0.306	6	6.3
	*TLR23*	—	29	941	0.399	32	34.0
	*TLR25*	Both	23	821	0.200	1	1.2
	*TLR27*	—	3	—	—	—	—
		—		14,396	—	102	7.1
Amphibian	*TLR1*	Nonviral	45	842	0.289	6	7.1
	*TLR1L*	Nonviral	28	796	0.260	1	1.3
	*TLR2*	Nonviral	56	780	0.287	5	6.4
	*TLR3*	Viral	38	895	0.239	1	1.1
	*TLR4*	Nonviral	8	872	0.281	1	1.1
	*TLR5*	Nonviral	28	879	0.270	4	4.6
	*TLR5L*	—	49	653	0.262	0	0
	*TLR7*	Viral	15	1,054	0.182	1	0.9
	*TLR8*	Viral	19	1,037	0.226	1	1.0
	*TLR9*	Viral	20	1,030	0.239	0	0
	*TLR12*	Nonviral	7	894	0.286	1	1.1
	*TLR13*	Viral	25	948	0.309	3	3.2
	*TLR14*	Both	38	835	0.225	0	0
	*TLR19*	Viral	21	950	0.325	4	4.2
	*TLR21*	Viral	28	937	0.273	3	3.2
	*TLR22*	Viral	17	944	0.268	1	1.1
		—		14,346	—	32	2.2
Reptile	*TLR1*	Nonviral	17	654	0.334	1	1.5
	*TLR2*	Nonviral	20	783	0.420	18	23.0
	*TLR3*	Viral	14	896	0.270	0	0
	*TLR4*	Nonviral	13	840	0.326	2	2.4
	*TLR5*	Nonviral	13	885	0.398	10	11.3
	*TLR5L*	—	9	654	0.236	0	0
	*TLR7*	Viral	13	1,059	0.284	2	1.9
	*TLR8*	Viral	11	1,049	0.338	0	0
	*TLR9*	—	2	—	—	—	—
	*TLR13*	Viral	9	948	0.322	1	1.1
	*TLR14*	Both	13	835	0.126	2	2.4
	*TLR15*	Both	8	823	0.341	5	6.1
	*TLR21*	Viral	11	956	0.153	1	1.0
	*TLR22*	Viral	11	952	0.395	1	1.1
		—		11,334	—	43	3.8
Bird	*TLR1A*	Nonviral	32	818	0.354	17	20.8
	*TLR1B*	Nonviral	29	652	0.385	28	42.9
	*TLR2A*	Nonviral	23	792	0.376	22	27.8
	*TLR2B*	Nonviral	30	732	0.337	21	28.7
	*TLR3*	Viral	37	896	0.334	20	22.3
	*TLR4*	Nonviral	35	843	0.417	27	32.0
	*TLR5*	Nonviral	34	861	0.414	33	38.3
	*TLR7*	Viral	39	1,059	0.306	24	22.7
	*TLR15*	Both	34	868	0.258	20	23.0
	*TLR21*	Viral	8	972	0.082	0	0
		—		8,493	—	212	25.0
Mammal	*TLR1*	Nonviral	16	786	0.413	6	7.6
	*TLR2*	Nonviral	24	784	0.325	8	10.2
	*TLR3*	Viral	23	904	0.240	6	6.6
	*TLR4*	Nonviral	22	839	0.414	21	25.0
	*TLR5*	Nonviral	23	858	0.317	10	11.7
	*TLR6*	Nonviral	22	796	0.383	5	6.3
	*TLR7*	Viral	22	1,049	0.257	12	11.4
	*TLR8*	Viral	23	1,038	0.286	24	23.1
	*TLR9*	Viral	22	1,032	0.162	11	10.7
	*TLR10*	Nonviral	17	811	0.346	1	1.2
	*TLR11*	Nonviral	11	931	0.434	14	15.0
	*TLR12*	Nonviral	14	906	0.416	19	21.0
	*TLR13*	Viral	9	991	0.332	1	1.0
		—		11,725	—	138	11.8
		—		60,294	—	527	8.7

Note.—Natural selective analysis was excluded for fish *TLR13*, *TLR27*, and reptile *TLR9* because there are only a limited number of sequences for these genes.

^a^ TLR genes were classified into two groups (viral and nonviral) according to the microorganism ligands they recognize. “Both” indicate TLRs that can bind viral and nonviral ligands. The ligands for TLR5L and TLR23 remain unknown; we did not assign a viral/nonviral status. See Materials and Methods for detailed criteria of viral and nonviral classification.

^b^ Number of sequences for each TLR gene.

^c^ The protein sequence length in reference vertebrate TLRs (NCBI accession numbers are listed in supplementary table S6, Supplementary Material online).

^d^ Estimated mean d*N*/d*S* for each TLR gene using the codeml method. d*N*, number of nonsynonymous substitutions per nonsynonymous site; d*S*, number of synonymous substitutions per synonymous site.

^e^ Number of PSSs detected in the investigated species per TLR gene by any one of codeml (M7/M8) and FUBAR methods. See supplementary table S5, Supplementary Material online, for more details.

^f^ The percentage of PSS per entire TLR amino acid sequence.

Four TLR genes (*TLR11*, *TLR12*, *TLR19*, and *TLR20*) were classified into subfamily TLR11, though not all four members exist in each vertebrate taxon. Although the TLR11 subfamily can be tracked back to fishes, no orthologous lineage of TLR11 subfamily member extends from fishes to mammals, which suggests a high turnover rate of the *TLR11* lineage. Interestingly, no members of the TLR11 subfamily were observed in birds or reptiles. Indeed, the *TLR11* gene is unique to mammalian species, and *TLR20* was found only in some species of fishes. *TLR19*, present in fishes, appears to have been lost from amniotes but has expanded in some amphibian species.

*TLR13*, *TLR21*, *TLR22*, and *TLR23* grouped into the subfamily TLR13. The ECDs of subfamily TLR13 members possess a single-domain architecture, which is different from that of subfamily TLR11 (Wang et al. 2016). Therefore, we split the original subfamily TLR11 into two groups: the new subfamily TLR11 and the subfamily TLR13. The *TLR13* gene is the only member of the TLR13 subfamily identified in mammals and *TLR21* is the only member of the TLR13 subfamily identified in birds. Some fish species have multicopy *TLR23* genes, whereas it was lost in the tetrapod ancestral lineage (supplementary fig. S3, Supplementary Material online).

Because the phylogenetic analysis revealed that it does not group with any other identified TLR subfamilies (fig. 3), TLR15 might be classified into an independent subfamily. We found that *TLR15* is present in nearly all studied avian and partial reptilian genomes but is absent in mammalian genomes, indicating that this gene emerged 310 Ma after the divergence of bird/reptile and mammalian ancestral linages. As *TLR15* underwent pseudogenization or was lost in some reptile species but is functional in birds (supplementary table S2, Supplementary Material online), we expect *TLR15* to be more indispensable in birds compared with reptiles for sensing the pathogenic ligands of RNA viruses and lysates from yeasts (Boyd et al. 2012; Chen et al. 2013; Heidari et al. 2015).

Our analysis indicated that the TLR gene family is highly variable in number among different vertebrate groups due to extensive gene gain and loss. The majority TLRs were already present at the emergence of fish but were since lost from certain lineages. In this study, we identified an overall increased number in TLR repertoires in species along the evolutionary transition from fish to amphibian and a subsequent decrease from amphibian to terrestrial vertebrate (supplementary fig. S3, Supplementary Material online). The observed greater number of TLRs in amphibians than in other classes may have contributed to adaptation to the complex pathogen environment of amphibious habitats.

### Modes and Strength of Natural Selection on Vertebrate TLR Genes

Despite several attempts to understand the adaptive evolutionary pattern of vertebrate TLRs, a clear picture of the evolution of this gene family has not emerged, in part because previous studies have generally focused on a subset of vertebrate TLRs or were limited to specific vertebrate clades (Barreiro et al. 2009; Wlasiuk and Nachman 2010; Alcaide and Edwards 2011; Babik et al. 2015; Liu et al. 2017; Velova et al. 2018). In this section, we tested the modes and strength of natural selection acting on all vertebrate TLRs. Specifically, the selection landscape has been reported to differ between viral and nonviral TLRs in the human population (Barreiro et al. 2009) and specific vertebrate clades (Wlasiuk and Nachman 2010; Velova et al. 2018), and we try to extend this interesting dichotomy to the overall vertebrate group. Values for d*N*/d*S* (*ω*) (table 1 and supplementary table S4, Supplementary Material online) suggest that all vertebrate TLR genes are under purifying selection (*ω* values ranging from 0.082 for *TLR21* in birds to 0.434 for *TLR11* in mammals), which is indicative of functional constraints for vertebrate TLR genes. It is not surprising that although TLR proteins lie directly at the host–environment interface, many functional residues are highly conserved to provide the rigid structural framework for recognizing structurally conserved PAMPs of pathogens (Werling et al. 2009). Nevertheless, it is also noteworthy that the range of vertebrate TLR *ω* values (0.082–0.434) and their mean values for each vertebrate group (0.250–0.333) are comparable with those of many rapidly evolving genes, such as mammalian reproduction-related genes (Swanson et al. 2001) and avian ossification-related genes (Zhang et al. 2014).

Despite such evidence of purifying selection acting on vertebrate TLR genes overall, instances of statistically significant positive selection (as detected by PAML and FUBAR methods) were observed across almost all vertebrate TLR genes, at a total of 527 of 60,294 (8.7‰) amino acid positions (table 1 and supplementary tables S5 and S6, Supplementary Material online). Within 527 PSSs, 148 PSSs (28.1%) were identified by both methods (supplementary table S5, Supplementary Material online), thus indicating that these sites are under strong positive selection. The number of PSSs varied substantially across TLRs (from 0‰ for several TLRs to 42.9‰ for the bird *TLR1B*; table 1). For orthologous genes shared by all five vertebrate groups (*TLR3*, *TLR4*, *TLR5*, and *TLR7*), *TLR3* displays the lowest proportion of PSSs (6.5‰), and *TLR7* has the lowest mean *ω* value (0.281), indicating that these two genes are under stronger purifying selection than are other orthologous TLRs. This pattern was confirmed through branch-site analysis: only 3 sites were deemed positively selected along the ancestral branch of each vertebrate group for *TLR3* and *TLR7* compared with 20 sites for *TLR4* and *TLR5* (supplementary table S7, Supplementary Material online). Other genes, such as bird *TLR1B*, *TLR4*, and *TLR5* and fish *TLR23*, display high accumulation of codons that exhibiting positive selection. Our results are mostly consistent with the findings of Velova et al. (2018), who suggested that positive selection is acting more on bird *TLR1B*, *TLR2A*, *TLR4*, and *TLR5* than on other bird TLRs. Interestingly, in a severely bottlenecked population of New Zealand’s Stewart Island robin, the greatest number of haplotypes compared with other TLRs was found for *TLR4* (Grueber et al. 2012). Previous studies have detected signatures of balancing selection on some TLR genes from primates (Dannemann et al. 2016) and rodents (Kloch et al. 2018), and the balancing selection on TLR genes may also contribute to species adaptation to the pathogenic environment. It is reasonable to speculate that balancing selection, which has been demonstrated to frequently target immune-related genes (Ferrer-Admetlla et al. 2008; Key et al. 2014), is probably an important force in shaping the evolution of vertebrate TLR genes.

Our results also indicated that the proportion of PSSs of TLRs varies between different vertebrate groups (from 2.2‰ for amphibians to 25.0‰ for birds; table 1), reflecting class-specific differences in the strength of natural selection acting on the antipathogen system. Although the overall pan-genomic background evolutionary rate in birds was much lower than that in mammals (Zhang et al. 2014), bird TLRs exhibited substantially high accumulation of codons exhibiting positive selection when compared with mammals (table 1). However, a high accumulation of codons exhibiting positive selection was not detected in the ancestral branch of modern birds for four orthologous genes shared by all five vertebrate groups (supplementary table S7, Supplementary Material online). Highly mobile and migratory habits expand the geographic ranges and increase the dispersal ability of most modern birds and are expected to enhance the spread of pathogens and risk, with more severe challenges from diverse pathogens (Altizer et al. 2011; O’Connor et al. 2018). We hypothesize that the substantial adaptive evolution among avian TLRs may increase the ability of pathogenic sensing and defense. Amphibians are a very diversified group of vertebrates that connect aquatic and terrestrial taxa (William and Trueb 1994). The uniqueness of such a life history exposes amphibians to a complex pathogenic environment derived from both water and land, and we assume that pathogen diversity might also result in substantial adaptive evolution of this gene family. However, this assumption does not agree with our result that TLR proteins of amphibians are overall functionally conserved, exhibiting the lowest level of positive selection among different vertebrate taxa (table 1). If our theoretical prediction is reasonable, it is possible that the expansion of the amphibian TLR gene family, but not site variations, may contribute to adaptation to the complex pathogenic environment of amphibians.

**Fig. 4.**
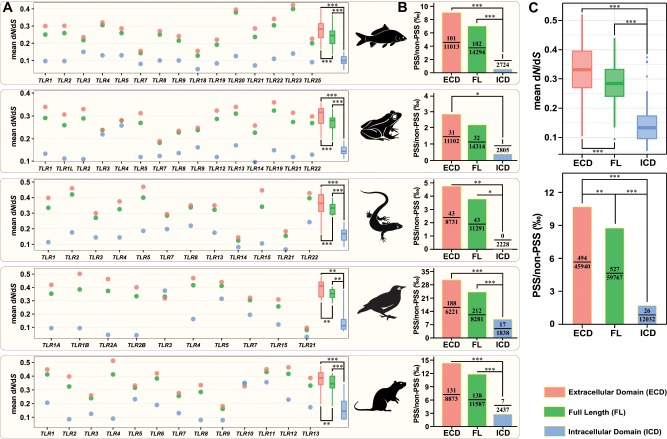
—Estimation of the intensity of natural selection acting on the FL, ECD, and ICD of each vertebrate TLR gene. (*A*) Differences in mean d*N*/d*S* values (*ω*) across the ECD, FL, and ICD of TLR genes in fishes, amphibians, reptiles, birds, and mammals. Mean *ω* values were estimated using the codeml method. Overall, *ω* values were significantly higher in the ECD than in the FL and ICD of TLR genes in all five vertebrate taxa (paired Wilcoxon test). (*B*) Distribution of PSSs among the ECD, FL, and ICD of TLR genes in different vertebrate taxa. PSS/non-PSS indicates the ratio of the number of PSSs identified in ECD, FL, and ICD of TLR genes to the number of sites which were not PSSs. PSSs are unevenly distributed between the ECD and ICD of TLR genes (Pearson’s chi-square test). (*C*) Patterns of natural selection acting on the ECD, FL, and ICD of TLR genes in the overall vertebrate group. Mean *ω* values were estimated using the codeml method. PSS/non-PSS indicates the ratio of the number of PSSs identified in ECD, FL, and ICD of TLR genes to the number of sites which were not PSSs. Asterisks indicate FDR-adjusted *P* value (*q* value): *q <* *0.05, **q <* *0.01, and ***q <* *0.001. See supplementary tables S4 and S5, Supplementary Material online, for detailed information.

We evaluated the evolutionary pattern among different functional domains of TLRs by estimating global *ω* ratios. For TLRs in each vertebrate group (fig. 4*A*) and vertebrates as a whole (fig. 4*C*), we observed significantly higher mean *ω* values in ligand-binding ECDs than in the corresponding FL gene and ICDs (paired Wilcoxon test, all FDR-adjusted *P* values < 0.01). The domain-specific *ω* values show that the vertebrate ECD evolved faster than did the ICD, as previously reported in primates (Wlasiuk and Nachman 2010). Furthermore, as previously shown in birds (Grueber et al. 2014; Velova et al. 2018), we found a general pattern in each vertebrate group and in all vertebrates that the majority of TLR PSSs (with 88.7% in bird to 100% in reptile; fig. 4*B* and *C* and supplementary table S5, Supplementary Material online) are significantly situated in ECDs (Pearson’s chi-square, all FDR-adjusted *P* values < 0.05). This finding suggests that pathogen-mediated selective pressures are particularly diverse for TLR ECDs. These heterogeneous evolutionary rates across TLR proteins reflect not only coevolution between the ligand-binding ECDs of the host and PAMPs of pathogens but also the vital function of ICD maintenance for downstream signal transmission. As domains are distinct functional and/or structural units in a protein, it is reasonable to consider that different domains likely evolve under different selection regimes. Nonetheless, for domains of membrane-spanning proteins, PSSs are not always significantly situated in ECDs. For example, regarding visual opsin genes (*rh1*, *sws1*, and *lws*) in snakes, most of the amino acid sites inferred to be under positive selection are located in TM domains, which is supposed to have a substantial impact on the tertiary structure, thermal stability, and aspects of the opsin retinal binding pocket (Simoes et al. 2016). The above results suggest that the same domain may be subject to different selection patterns in different proteins.

**Fig. 5.**
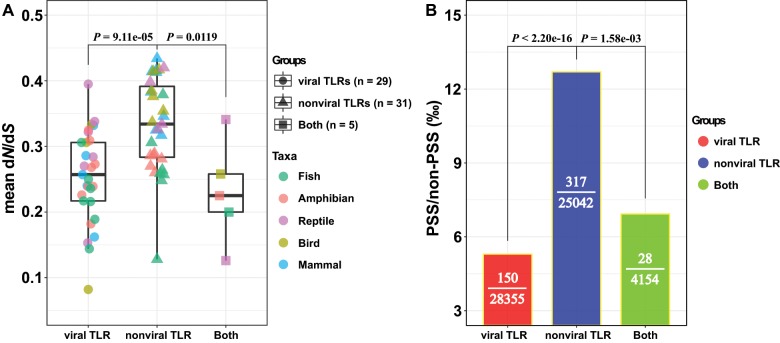
—Different selective patterns among different vertebrate TLR groups (viral TLRs, TLRs sensing viral ligands; nonviral TLRs, TLRs sensing nonviral ligands; Both, TLRs that sensing both viral and nonviral ligands). (*A*) Strength of purifying selection acting on the three groups of TLRs, as measured by estimated mean d*N*/d*S* values (*ω*). Mean *ω* values were estimated using the codeml method. A significant difference in *ω* was detected between nonviral TLRs and viral TLRs and TLRs that sensing both viral and nonviral ligands (two-tailed Wilcoxon test; *W* = 715, *P *=* *9.11e-05; *W* = 133, *P *=* *0.0119). Colors represent different vertebrate taxa. Shapes represent different vertebrate TLR groups. (*B*) Distribution of PSSs among the three groups of TLRs. PSS/non-PSS indicates the ratio of the number of PSSs to the number of sites which were not PSSs. PSSs are unevenly distributed between nonviral TLRs and viral TLRs and TLRs that sensing both viral and nonviral ligands (Pearson’s chi-square test; *P *<* *2.20e-16; *P *=* *1.58e-03).

By pooling all vertebrate TLRs, we observed that the *ω* values of nonviral TLRs were significantly higher than those of viral TLRs (fig. 5*A*; two-tailed Wilcoxon test, *P *=* *9.11e-05). This interesting dichotomy between vertebrate nonviral and viral TLRs indicates that viral TLRs are under stronger evolutionary constraints than are nonviral TLRs. Moreover, we also observed that positive selection is acting more on nonviral than on viral TLRs (fig. 5*B*; Pearson’s chi-square test, *P *<* *2.20e-16). This is most likely because nonviral microbes display more complex PAMPs (Barreiro et al. 2009), which can be simultaneously detected by different nonviral TLRs (such as lipopeptides in *TLR1*/*2*/*6*/*10*, profilinlike protein in *TLR11*/*12*/*20*, and flagellin in *TLR5*/*5S*), compared with the low structural variation for viral ligands that are almost specifically recognized by corresponding viral TLRs (e.g., ds RNA by *TLR3*, bacterial DNA by *TLR9*, bacterial 23S ribosomal RNA by *TLR13*, and CpG oligodeoxynucleotides by *TLR21*). This supports the previous findings by Velova et al. (2018) in birds and Wlasiuk and Nachman (2010) in primates. Similar results were also reported in a population-level study of humans by Barreiro et al. (2009), who found higher evolutionary flexibility among nonviral TLRs than viral TLRs. Notably, we extended this interesting dichotomy to the overall vertebrate group. It is also worth mentioning that significantly higher *ω* values (fig. 5*A*) and a greater ratio of PSSs in total positions (fig. 5*B*) were also observed for nonviral TLRs than for TLRs that sensing both viral and nonviral ligands, highlighting the strong selective constraints on the viral-sensing TLRs and the essential nonredundant role played by viral-sensing TLRs in host survival.

The interpretation of our selection results is notable on one count. Genetic Algorithm for Recombination Detection analyses found evidence of 72 recombination breakpoints in 30 out of 68 vertebrate TLR genes (supplementary table S5, Supplementary Material online). However, recombination is not taken into account in our study. Among the 527 PSSs analyzed in the present study, most (441, 83.7%) were also identified as PSSs in the data set when recombination was considered (data not shown), indicating good reproducibility between these two analytic strategies. Additionally, the presence of putative recombination points in many vertebrate TLR genes may imply the importance of recombination mechanisms in the functional and structural evolution of vertebrate TLR genes.

### Functional Assessment of Positively Selected Sites Identified in Mammalian TLR Genes

**Fig. 6.**
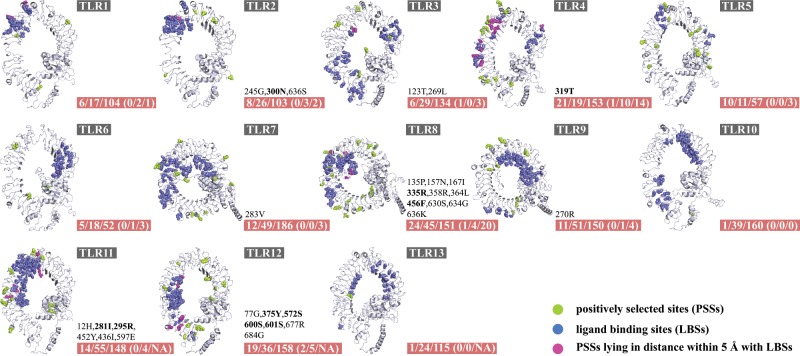
—PSSs of mammalian TLRs visualized on 3D structures of human and mouse TLRs. The PSSs identified by any one of codeml (M7/M8) and FUBAR methods were considered and shown in green. For position information of each PSS, see supplementary table S5, Supplementary Material online. The LBSs described in previous studies are shown in blue. Purple coloration highlights PSSs at LBSs or within 5 Å of LBSs. The first three numbers shown in red rectangles are the total numbers of PSSs, LBSs for each TLR, and sites within 5 Å to LBSs; in parentheses are the numbers of PSSs at LBSs, PSSs at sites within 5 Å of LBSs, and PSSs detected in other studies. The positions of PSSs detected by both methods are shown, in which these PSSs at or very near known LBSs (i.e., within 5 Å of LBSs) are highlighted in bold.

We further evaluated the functional effects of PSSs detected on mammalian TLRs because there are many reliable functional studies on human TLRs that can be used as functional references. Using the codeml M7/M8 and FUBAR methods, only 138 of 11,725 codons (11.8‰; table 1 and supplementary table S5, Supplementary Material online) exhibited significant patterns of positive selection, with the number of PSSs differing between 13 mammalian TLRs (from 1.0‰ to 25.0‰; table 1 and supplementary table S5, Supplementary Material online). A total of 35 of the 138 inferred PSSs are located at or very near known ligand-binding sites (i.e., LBSs or sites within 5 Å of LBSs; fig. 6), with the probability of changing the ligand-binding properties of these receptors. Interestingly, we found that positive selection is more likely to be at or near known LBSs than other sites (Pearson’s chi-square test, *P *=* *0.027), further reflecting the coevolutionary dynamics between the TLR proteins and their pathogenic counterparts. Moreover, some PSSs or neighboring positions are polymorphic in humans, and they have been reported to be associated with disease phenotypes. For example, TLR2 S450 (neighboring PSS G453) shows an association with susceptibility to lymphatic filariasis, which is caused by *Wuchereria bancrofti* (Junpee et al. 2010), and TLR4 299G (neighboring PSS Y295) has been linked to different levels of susceptibility to bacterial infections (Kiechl et al. 2002) as well as a higher prevalence of atopic asthma in Swedish children (Fageras Bottcher et al. 2004). Both of these sites (G453 in TLR2 and Y295 in TLR4) have also been identified as positively selected sites in previous studies (Wlasiuk and Nachman 2010; Areal et al. 2011). Among the 138 PSSs, 31 are inferred by both methods (codeml M7/M8 and FUBAR) (supplementary table S5, Supplementary Material online). A total of 10 of the 31 inferred PSSs are located at or near known LBSs. Additionally, the PSSs identified in the present study are generally consistent with those identified in other studies, especially in TLR4 and TLR8 (fig. 6). The PSSs in these two cases may indicate that the target sites are under strong positive selection and promising candidates for further functional research.

The assessment of the functional effects of PSSs on mammalian TLR genes is notable on one count. The evidence for positive selection in the present study is based on testing amino acid variants in TLR coding regions. Previous studies on primates (Enard et al. 2010) and specifically humans (Barreiro et al. 2009; Laayouni et al. 2014; Dannemann et al. 2016; Deschamps et al. 2016) have shown that signals of selection for TLRs were often detected on variants located in noncoding regions, such as for the *TLR1*/*6*/*10* gene cluster. Moreover, several regulatory and phenotypic associations between such noncoding variants and host immunity and pathogenic resistance had been revealed in a previous study (Dannemann et al. 2016). However, in the present study, the picture of selection is limited to coding regions, and those noncoding, positively selective variants cannot be covered. Hence, future functional studies should focus not only on the variants located in TLR coding regions but also on the variants located in TLR noncoding regions.

## Conclusions

To our knowledge, this is the first study investigating the evolutionary history of the vertebrate TLR gene family in a large number of species and most vertebrate groups. Our analysis provides a broad outline of the complex evolutionary processes of the vertebrate TLR gene family, including gene duplication, pseudogenization, purification, and positive selection. Specifically, we show that although the emergence of sccTLRs predates the separation of bilaterians and cnidarians, most vertebrate TLR members originated shortly after vertebrate emergence. Our phylogenetic analyses divided vertebrate TLRs into eight subfamilies, and TLR3 may represent the most ancient subfamily that emerged before the branching of deuterostomes. Moreover, we extended the interesting dichotomy that viral TLRs evolved under stronger purifying selection than nonviral TLRs from the level of the human population and the specific vertebrate clade to the overall vertebrate group. Positive selection is more likely to be at or near known LBSs than other positions of TLR proteins, reflecting the coevolutionary dynamics between the TLR proteins and their pathogenic counterparts. To thoroughly understand the complex evolutionary history of the vertebrate TLR gene family, further research would benefit not only from refinement of the evolutionary relationship among TLR members within specific TLR subfamilies but also from defining the pathogenic sensing function for all vertebrate TLR proteins to identify vertebrate group-specific immune innovations.

## Supplementary Material

evz266_Supplementary_Data

## Notes

**Data deposition**: All genome sequences and transcriptomic RNA-seq data used in this study were previously published and downloaded from public databases. The data sets used for analyses, identified TLR homologs, and their respective accession numbers are available in supplementary tables S1–S3, Supplementary Material online. The sequence alignments generated in this study are shown in supplementary texts S1 and S2, Supplementary Material online.
