# Supplementary material for: Evolutionary History of the Toll-Like Receptor Gene Family across Vertebrates
Source: Genome Biol Evol. 2019 Dec 4;12(1):3615–34. doi: 10.1093/gbe/evz266 (PMC6946030; doi:10.1093/gbe/evz266)
Supplement: evz266_Supplementary_Data [file evz266_supplementary_data.zip › Supplementary text S1.docx]

Supplementary text S1. Codon alignments for sccTLRs and mccTLRs of metazoan species.

>Pate_TLR_9

------------------------------------------------------------

------------------------------------------------------------

---------MRCPFLSI-------------------------------------------

------------------------------------GDNAFPHAHLLFKLHLSHTEFH-R

I-PSA-----------------------IRNLKTLKALKIVNGK----------------

-----------------------LTEITTELQSMS----ELVNLSLAQNKI---------

------SSLLGSAFKG----DLKLQSIIL---TSNRLSS-------------LDIKI---

-------FDQCPSLRLVRLDK--------------------------NDLL---TVD---

------------GLFRN--SNLMN---------------------IDLSFNA-LSTIPED

------------------------------------------------------LFNNLE

N--------------LMGINLEGNFI------RS--IGNCFAQSHRLECINLSNNMIK--

------------------------------------------------------------

----------------------------------------------------------SC

NKTFHSLESLKTINL----RHNSLPSNFI------------------------------R

SIGNCFAQSH--RLEC-----------INLSNNMIKS-----------------------

---------------------------------------------------------CNK

TFHSLQSLKTINLRHNSLP----SI-------------TRNYF----SSLPLLEEL----

----------ILKQN---------------------------------------------

--NISQ-----IDSEAFS--EL---E------RLKKIDLSD--NR---LTTLSRES----

----FPRALK-----------------------------IQEII---------------T

TGNNWACDCRLL----------------------------WIK---EWSEHA-TF-----

---KNLNNEFN------CSSPLSFKSK------------L----------------LNQL

--------------TKQDL-SWPKE-------------------------------DCPT

GCQCSCAPYQEDGYANVD------------------------------------------

----CSSKRLSRILDVNDTRCSFEEDRENMRGR----VIITLS----------EIELCP-

-------------------KIIRNYILMG-------------------------------

------------------------------------------------------------

------------------------------------------------------------

--------------------------VGGAGEGVSLNLSKMMEQKST-------------

----AV------KQLTGGIAHLFKQNKI----PSRFPELTIELNLRE----NLLSNPIEL

N-----LPPM-SKLLTLNLERN--FISRLDFSL--------PNNIETLRLAEN-NIT---

--RFFNNP-----P------------------------SNIK-TFTLSH----NPWQCD-

--CDTLP-----------------------------------------------F--RKW

IM----VESSKI-------------LDVN---------DTRCSFEEDRE----NM---RG

RVIIT-----------------LSEIE-LCPK------LI--------------------

----------RNYILMGVGGAVILMMTVASILLY---FRYRY-HLKVWLY----------

-----SRGW--RCFKKRDKRDD-GK-------------LFDAYISFTD---E--DADS-V

RKHF--LPELE------EKHP----FY----KLFVP-QRDIKEDNF-EINYLME-RVVDS

KRIIILLTKH---YLLNEFC-MEMLRMAFA--NSLEEKL---HQ----VILVKIGPLP-P

IKEMDQSLKI--VMESSRCLKF------------GTRLFWEMLRYEMP------------

----------------------E---------------------KS--------P--NID

DYELLDDQPDDVPLIQEL------------------------------------------

------------------------------------------------------

>Bigl_TLR_23

------------------------------------------------------------

------------------------------------------------------------

------------------------------------------------------------

------------------------------------MNCSVHCRCRDSTLESTMKQYY-F

K--------------------------------HLSIMGAAYRP----------DIQ---

------------------N----FDIKSDYMSFLGIFAPILHNIALVSCEL---------

--SDGRNHTITSLLNG--VDHGMYTALDV---KCTKKET-------------IIWDI---

-------PSWHTSFNIFVSDN--------------------------CNVTTYSQLV---

--------VPMNMWIMD----------------------------IDAGGEHVMRTIDLS

YT----------------------------------------------------------

---------------LNTLSLALAGSRSKF---------IPQKWSNAYMCSVTLLSFK--

------------------------------------------------------------

---------DNWLEDFNCIITLTKRLD-------------------------------TL

NLEGNVMTRFPKCLL----DSKYVFLNYL------------------------------S

LAHNRIQDLS--PM-------------YDLPGNNGVP-----------------------

------------------------------------------------------------

------DINIINLSYNDIS----EV---------------HSL----RDMGRLKIL----

----------DLSHN---------------------------------------------

--KIHE-----ISGNAFV--SL---K------YLNTLLLGN--NR---LFKLDLQM----

----LLPSSN-----------------------------LEKLD---------------V

SHNYI-LSVNEG----------------------------------NIVNISKST-----

---LELDLQYN---------RLSNPP------------------------------LKDC

--------------RKLLL-TNTNL----------------------------------K

ILSAYNPYLCDCSFIGFE------------------------------------------

------SCMKWLDEQNKSSAKHVFQDLNQM-KC----SSPPSNKGITIRDLNFHRYCVV-

------------------LEDCPPSCTCY-------------------------------

------------------------------------------------------------

------------------------------------------------------------

--------------------------LQER---DILKVN---CSSRR-------------

----FL-----------------EMPVI----IPNLTNVYTVLYLDH----NPLQ---SL

N-----YQPYLSRLSEIYIDNC--LLTTVMPSA-IAA----LKNIRVMTLHNN-LLQKLP

T-STRNIT-----L------------------------EKAT-NITLHN----NRWACS-

--CESL--------------------------------------------W---L--PRW

IS----RHK----------------AVLW------KPGNILC-----------DY---FQ

KPLED-----------------VSEADLNCKS------WS--------------------

----------AMDNFLTVILFVLSTVATVIL--F---FCYNT-DICAIVY----------

-----SKLG--IEFNSRLLYGD-QY----------C--PFDILISYGQ---D--NYKW-V

VDTL--VPYLE------KNPG----GY----RVCLN-HREFPSSDC-VLETLPT-AVRLS

RSAILVLSKE---FLQKEWC-MLEVRVAIQ--RLLLVG----SK----LLIICMDKVNVD

ELSP----ELRAYIHTHHYLRY------------DEHDFWVKLDLFLP------------

------------------------------------------------------R-KLIR

NSEPVVQDSSLAAGGDCASKSIDKCEENEGTDCTEAL-----------------------

------------------------------------------------------

>Ciin_TLR3

------------------------------------------------------------

---------------------------MLSNGSCIVNPR---------------------

---------LNMLKLFLRNL----------------------------------------

------------------------------------NYSNIPHCIPNSVTTMSLIDIP-L

T-SFN--------KDINT-TGLQEFVKWPE---SLEVLYIKN------------------

------------------ARTGPIGNH-VFKNMPL----SLRTLQISSCKI---------

-------PWPRVSIRW----PRNINHLTL---IYCKVET-------------TDSTT---

-------FKDLVRVDFLSMRG--------------------------NLMT---DVP---

--------------FGL-PQTTKT---------------------LDVSYNR-MRTVSDN

------------------------------------------------------VWGGLN

N--------------LTKLYISNNQL---VSIPK--Y--LPSSLEQLNLKENQISYSD--

--------------------------------------------RDALERLVNLRQLDMS

YNRLLNMPQGLPASSMRRLTLNNNEIG-------------------------------LI

RSPADYKYCVNCSSI----SLQNNPWVCNQNLINMMLWLEQLQSTLRITLSGFCASIQTD

LGRVELKRIL--HY-------------MTYMASSFHN-----------------------

------------------------------------------------------------

CLYDVQNLQCKNASLSQLP----RP----------------------SPLGLLTVL----

----------VTNNK---------------------------------------------

--NLTL-----VPDNMFK--LQ---N------KLTSLNLES--NG---ITRFPRGL----

----PSS--------------------------------LLVIY---------------L

SYNKITAITEED----------------------------QS----TLDQLV-NL-----

---KQLYLSGN---------LIKILYD------------YQ------------LQRMASL

--------------RWLAL-NNNPMECDCSMQSLS---------------------LWYI

QAETTYHYTSIQRYLEPL------------------------------------------

-CVEPPNRRNQRITTIFGAYYYEYNCIPRVCTHRQGNLDCSYT--TQQSKNRTRFNVLP-

--------------------TIPRNTYWL-------------------------------

------------------------------------------------------------

------------------------------------------------------------

--------------------------TLDL---SNLQLE---NEKIS-------------

----LT----------------------------QLTRLTT-LNLTG----NKLT---SI

P-----LQGLPRSIENINLSRN--KISTLPATTLITC--YLP-NLKQLDLRNN-SFSTIQ

T-QEVS-I-----F------------------------LAVT-SVLLKG----NPLECN-

--CKLRP-----------------------------------------------L--ITW

IQ----TNEKNE-------------QDLS----THDLKDLIC---FTPK----RF---EG

RFIIN-----------------LSE---SEYC------PV--------------------

---------VNLSLIGGLVGGFTALLIIIIIIVNIYLYKKRKKQERRDIV----------

-----QGFK--DLLEKEAMNEG-DPETGVGVAPVTY--EYDAFVSYVS---DSDDVEF-V

YKML--EEMEE------KRER----------KMCIH-ERDFTPGRG-IADNIVE-CISTS

RRMVLVVSRK---YASSAWC-QYEVQIALT--ELHAKRR---GR---LLVPILLEDVTRD

EQYAGSVTTILSAITAIQAPKAQ-----DNDR--TWANFWNKLDKTLT------------

------------------------------------------------------------

------------------------------------------------------------

------------------------------------------------------

>Drme_Toll3

------------------------------------------------------------

------------------MELHTKIFILIIINPNIFICE---------------------

---------SKQMKLLLTIP----------------------------------------

------------------------------------DDYCETYCGGICNDTVASKTCD-R

E-AYDYIATNEYHLIMRD-GHLEVNWKIP----DPNIFIISPISKENRLKLNELIVS---

------------------DTSYPIRAVDYLRQL------GVETVTRFENQI---------

---NSFKVIERDVHYI--NGPKSLKIIIQ---SNLYLNE-------------IIEYI---

-------NKTTDNVNEIIINA--------------------------YKTVENQQIA---

--------LDN-LIFNG-KSHLRS---------------------LTFIGFQ-IENLSTK

------------------------------------------------------PFAQFI

N--------------LKRMVLTNCTV-RNLTFLR--T--LQKSLEHLELDIDNEVDLK--

--------------------------------------YFTNFSSLKFMKVRNYIPNKNF

TALICTHKNCNFIRGINGLECPKLCQC-----------------------------LYII

DDLELNIDCSNLGLL----QIPPLPIPSY-GGVKLNFSNNSLSQLPTMTLPGYKLVKRLD

VSRNRLTNLS--INHLPAKLDY-----LDVSFNEIIN-----------------------

--------------------------------------------------------MGND

VIKYLRTVPIFKQTGNQWT----IH------------CDDKPLLNFFRHLKLIIRM----

------------------------------------------------------------

--KSAE-----MKPMFLH--SL---T------ELPKGFLKFLGKH---FIWLGVRK----

----QEYYLI------------------------NEEQLLQSMH---------------R

KLNNLNTIMS------------------------------------IYKYMEWLH-----

---RKLIFVNR-----------EYDLF------------YIRQMAAPCPHKCECCYSRDS

--------------LILKIDCRNKF-----------------------------------

VYNFPDIVARNSRLMGKQ------------------------------------------

----NMSSPMELHLSKNNISNITIAMLPKELRF----LDLRFN----------NLVTLD-

-----------------------DKVLSY-------------------------------

------------------------------------------------------------

------------------------------------------------------------

-------------------------LKKNS---IKTKLS---GNPWN-------------

----CD----------------CKSRSV----LSILRDHEP-LEYDVTLKRCNIS---PT

DCPDVCVCCL-DNLTWPSFIVDCRGEGLLQMPS-LSS------RVTYVDLRNN-NLTALS

Q-KNRS-S-----I------------------------ENRSLKLHLLD----NPWSCS-

--CNDIE-----------------------------------------------K--INF

MK----SVSSSI-------------VDFT---------EIKC------S---------NG

EKLVS-----------------INQ---HIVC------PS--------------------

----------DLFYYLALAISLVATIIALNFLIW---FRQPV-LVWFYEH----------

-----GVCL--SLSAKRELDKD-K--------------RFDAFLAFTH---K--DEA--L

LEEF--VDRLE------RGRP----RF----QLCFY-LRDWLAGES-IPDCIGQ-SIKDS

RRIIVLMTEN---FMNSTWG-RLEFRLALH--ATSRDRC---KR----LIVVLYPNVKNF

DSLD---SELRTYMAFNTYLER------------SHPNFWNKLIYSMP------------

------------------------------------------------------------

--LLPSYVD---------------------------------------------------

------------------------------------------------------

>Drme_Toll4

------------------------------------------------------------

------MEHSKLWDLRPEVRERRFKWTSDGQQQQQQQLGWCNNKDDPPNSHQKSKSNNDA

RNLNSRVRVRARVRVVPGEMRMGDINCSNGLGNIREDYCEIYLDELGENGTCSIANNEVT

TEDYQMKLVFLKLEINWTSPVFHGWNIFKIC-----NETDYELVIISVLGIRSEVDMR-I

S-PAVQYLSLLGIREISG-YDI------YLPSVLITEMDVHHANGPKMVTFKYLYDS---

------------------TVNSVITNN-YIRKTMN----NTEKIKIYYHNT---------

-FEKTTLTMEKNIFHG----KNKMSALIF---NGLKIKG-------------LTNNT---

-------FENLTSLNTLIFDN--V---------------FLKDLSFLRSSTLQSSLT---

--------YCI-MKVDN-MVDLKS---------------------FEKFTNLEIIEVSQY

K-----------------------------------------------------GFKNFT

AF-------------ICEPYKSHCKF-TLGINEV--A--CPLKCNCSYNRDKSQLEID--

-----------------------------------CWQKNLTTIPSLPVPKKGSSALVFQ

SNLLAELP-DNSLEGYHNLKSLDVSYN--------------------------QLTSLSV

SQLPESLHYLDIRHN----KITTLSPQVV-EYLYSVNVFNQYGNKWSIYCDEYHL-QEFF

WYKAKLLRIK--TSKFQTIMEY-----IELSSKGSFV-----------------------

------------------------------------------------------------

ENFFVQNIDQLYLEANEDE----II-------------DAFGPSDKYFNLKLMEAL----

-------------NH---------------------------------------------

---AIW-----LFSGEFD--EI---I-------LHHLNSPC--PY---RCSCCFEW----

----HTGEFL-----------------------------INCRN---------------L

SLDIY-PRLP------------------------------------NSIPYK--------

---TTLYLDRN---------EIRKLTN------------TE--------SLVVAG-HASI

--------------HKLHM-SQNLL-------------------------------RELP

LHLLP----ENITYLDVR------------------------------------------

---------NNLLKYLDDGVIAFLEYRENI-TK----IELSGNPWECNCKAKAFLSFLR-

-RHEPMEYETVLRRVEITDDKCPEDCICC-------------------------------

------------------------------------------------------------

------------------------------------------------------------

-----------------------VDTSNSDSLAYVVDCS---GKELS-------------

----EI-------------------PQL----PTPTYGQTT-LVFER----NSLK---KW

P--SSLLPGY-SSVTRFYLAHN--RLSDIDQLP--------D-KLEYLDISNN-NFSALD

D-RVRG-F-----L--------------------QKRMNSSQLQLSLFG----NPWTCR-

--CEDKD-----------------------------------------------F--LVF

VK--------EQ-------------AKNI-----ANASAIQC-----------ID---TG

RSLIE-----------------VEE---TDIC----------------------------

----------PSVLIYYTSLAVSLLIIALSINVF---ICFRQ-PIMIWFY----------

-----EHEI----CLSLAARRE-LD-----ED---K--KYDAFLSFTH---K--DED--L

IEEF--VDRLE------NGRH----KF----RLCFY-LRDWLVGES-IPDCINQ-SVKGS

RRIIILMTKN---FLKSTWG-RLEFRLALH--ATSRDRC---KR----LIVVLYPDVEHF

DDLD---SELRAYMVLNTYLDR------------NNPNFWNKLMYSMP------------

------------------------------------------------------HASHLK

RSRSDAETKV--------------------------------------------------

------------------------------------------------------

>Bigl_TLR_22

------------------------------------------------------------

--------------------------MAAGVSVGHVSGC---------------------

---------QLYILAVCLTLGLQLADS---------------------------------

------------------------------------LELLQNVPTTTYEPFNCPLECNCP

R-------SNSTLSSMPQYYTICTVSLVSPNNAAVRSILQSISTPKTAVLYMTCSYSQIN

LYEE--------------PPVSELWDG-AFEAM-T----SLRQLTFTKCAF---------

------QKLTRGAFEG----LTYLKKLSV---QYANIRE-------------LDANL---

-------LSNMQLLETLEISH--------------------------SSLR---NLF---

-------------SLCS-YTSLKN---------------------LNLSFNH-LANLEDL

GI--------------------------------------------------NCGGKSLH

N--------------LESLDMRNNLLTEIPNW----LSENLLNLHYLYLSGNLIENYD--

--------------------------------------------HLPLKNFSSLYLMDLS

NNSLTEIK-KDFLLGCDNLQYLYMSRN------------------------PIIYIQRQF

LKAVSNLVELEMVESRLTDSIWLEISDISKRLRILNLSRNRLTKINENTMSDLRL-EVLN

VSYNRIVGLNSNAFGSQTNLIT-----LDLSYNLITD-----------------------

---VPVRFSQNMTNLVHLLLNNNNIKV-----------------------------VQSE

AFMGLGKLESLDLSFNSLQ---------------------ELMPQVVGTLEHIVNV----

----------NLSYN---------------------------------------------

--HLRV-----LNSDLFF--KF---K------QMKHLNVSH--NA---LQELPF------

----LYGNVA-----------------------------LQDLD---------------A

SFNNITKVIAQ-----------------------------------TFQDLK-EL-----

---QTISLSHN---------LLSSLPFRMFKGCDNVKTIYLSFNLLSHLDDDFFTSSPRL

--------------TFIDL-SHNKI----------------------------------T

AMNNIFRYLNHLKFLQLS--------------------------------YNKITTLLRN

QLPRSLETLDISNNNIHQISSHTFKTLSNL-RY----VDLSVN----------NLTTLS-

---------QDEVEIAYNLLSKPTFNLVYNPLVCDCKLEWLKDWYDGKFKDTGTLPTFQT

TLTYGCISPLYSTKMPITSLRSDEFLCHYEKHCDKTCVCCDYDVCHCKYTCPSSCQCYIG

DKFLNIHQVHCFNANLTDVPGK--------------------------------------

-------------------------IPEGA---TLLRLD---GNNLP-------------

----SL------------------REHS----FLGLTHVVD-LYLNN----SHIH---TV

E--NNTFKGM-KSVRSLFLNNN--LLTIISPGV-FSG----LENLERIFLQNN-FISLID

P-QALLLPPYLYLINLRENDLNTLPIDGLWGFVNRSRESGLKVRFSLSQ----NPYSCQ-

--LDFVCK----------------------------------------------F--VLF

IR-----DSADC-------------IEDI--------SDIKC---SSNSLGQQSYYQ-DG

FTLLD-----------------FQI-E-LCSENQSFPTNM--------------------

SRNSVHSSSAKGETYALIAACVVIAFGLALLIVA---YMNRD-FLQVLCF----------

-----TRFG-LRVFKMAKATEDNDR-------------PYDAFISYSS---K--DEDF-V

IHQL--APRLE------NGDK----KF----QLCVH-YRDFPVGAC-IAETIVR-SVEAS

KRTILVVSDN---FLDSEWC-RFEFQTAHQ--QVLNERR---NR----VILILMHDLD-T

EKLD-STLKV--YMRTRTYLKY------------DDPWFWEKLMFAMP------------

---------------------------DVQHRKPPENIPCHMNGNMQYMPQNVTL-QHPH

RRVPTSCNGVRCETIHNDMYEIPILDSGSVHYQLANGRCCCTHTNSAYHNSDLSDSTSGF

HNGSVSSYGHYEEVGPSSSSMQSTPHKFVGTPPPVPSIPKEGFLPIGRVKTAYV

>Pate_TLR_10

------------------------------------------------------------

------------------------------------------------------------

---------MNVYGINVFLS----------------------------------------

------------------------------------LFTLALGINFDEDNDIASKNCS-E

L-LPGCDCYDLETIGIIQ-VKC------WNATELFNTSYLMDGSIFGVNETYEIIVE---

------------------GGVSALPPR-SLEGL------IVYRLILDDPGI---------

------LDIHEDAFEN----VLRLKRFHV---RGSSLKK-------------IPNLG---

--------PVRDSLQYLNLDN--------------------------SLLT---SLE---

--------GHA---LKN-FSFLEN---------------------ISFFNNS-IQQIDSD

------------------------------------------------------VFEGTN

N--------------VIIFDLSYNKL-AYL--PS--H--LFDSWKSLRKVALSHNQLL--

-------------------------------------------HVKNLFQFATPLFIYLD

YNNLTDIN-AILPSKNLNLTTLLIS--------------------------NNPITKVTP

TCFNDKAPNIKFIYM----DHCLIRKFDV-------SHYQNLAHLNTLDLSYNML-E--E

MPEQSINFGF--NLELVLVGNQIREFHAEMGYNVKRL-----------------------

---------------------------------------------YLTSNNL----KKLG

KTLRFTQLTEVSIDKNELQ----QL-------------AVEDF----VGVNELNVF----

----------KVQEN---------------------------------------------

--KIAR-----IDREAFD--TI---R-----NDLNNVDLSC--NL---LKSLNGSI----

----RFLSQ------------------------------LKYLN---------------L

TSNLI-EKFEDR----------------------------------EFAGLN-EL-----

---SELYIRGN---------RIVELGD------------Q-------------LESLPQL

--------------QFLVL-SSNRI-------------------------------QSLK

MEQIP----KSLQYLYLR------------------------------------------

DNPMQCDCRLLPFLQWLNSSGSPITDYPLCLST----ASNSSK----------RVQSCP-

---------------------TGCNCYCT-------------------------------

------------------------------------------------------------

------------------------------------------------------------

--------------------------KDADNYFMTLDCS---FKNMS-------------

----VL----------PQLLTVNSVDDS----FHLKINFEM-QFYEE----NSTV-----

---ENSIAVI-DTVRGINLSNN--NLESLENAR-WPS------DLRQLFLHNN-KLRKMT

N-VILL-D-----F------------------------PVLR-KVSLSG----NPWICD-

--CDTVE-----------------------------------------------F--RRC

IL----SKEEII-------------LDGN---------QTLCGKSDESG--NNAL---EG

KAIWT-----------------LDDRD-LCPT------FI--------------------

----------GLYFSIGFGLIAFSLVITVLKIVH---TRYEK-DIKVWLY----------

------SHG--IKWVKEKDIDK-DK-------------KYDAFISYS-------DKDFDA

VQNL--IRTIE------AKQP----MS----RLHFH-FRDFLGGAP-IEQNIIH-AVQNS

KRTIVVLSKN---YQESEWC-IFEFQRARS--QTLKDKV---NR----ILIIRMGELP--

DNLD-DNIKA--HLKSTTYLIW------------GEKFFWEKLFYALP------------

----------------------T-------------------------------S-GQKN

DKISSSDFNSKSCLVGI-------------------------------------------

------------------------------------------------------

>Pate_TLR_13

------------------------------------------------------------

-------------------------------MKLYGICL---------------------

---------LLCLFVFSNCKDSYLEDIDLEA-----------------------------

------------------------------------FDFDGPGPGVVCNMLPSDCNCDDL

YSLGRIWCANFTWHSHINFDTL----DEFSVINSNRGINLDGKKPNARCSELLKDCFC--

------------------MHLEAIAQHSCWDAIFNEQEDDISNRKCSNRPP---------

----YCKYRWYGCCDN----IYEDQDLDT---ENDEL---------------IPGCN---

-------FYEFETVTLVKCSE--------------------------ATEL---------

--------FAPKYLFNRSIFDVNV--------------------TYDIVVDGVVTTLPAR

SF-----------------------------------------------EGIKLFRIYLN

N--------------LDNEDIQENVLENVISLSI--FHVHCSSLKFIHLAYNNISDVA--

--------------------------------------------AILPTTAWNLTTLNIS

SNPIEQVPDDFFQVKAPNIKYLMMGHC---------------------------QIRNFN

MSHYKNLLHLQVLDL----SYNRIEVIPY-------------------PRIAFRLLVYLS

LEGNLIREIK--AFKTRLAI-------LNLFNNRLLS-----------------------

----------------------------------------------------------LG

TVLRFGQVRYVHVAKNEIQ----QL-------------TAWDF----KGLKELFEF----

----------KAQEN---------------------------------------------

--QIKM-----IERLTFA--GY---R-----NELRILNLNH--NH----LRTLNGS----

----FQYLSE-----------------------------LRELY---------------L

ASNFI-SVFEKG----------------------------------EFLGLK-SL-----

---YYLKINGN---------RIVTVGN------------C-------------LRNLSKL

--------------DILYL-SNNSI-----------------------------------

ETLRKEQMPDTLKYVDLS----------------------------------------VN

PFRCDCEFLPFIQWVFANPKAPHVFGGRKI-CV----PEFPSD----------NHHCPS-

----------------------QCDCYCA-------------------------------

------------------------------------------------------------

------------------------------------------------------------

--------------------------NDDEEYFMAVDCS---SKNIS-------------

----II--------------PQFLTAYS----VENSPHFKINLTFRF----HMVRKHYTI

D--KSVVIA--DTVGEINLSDN--NLESLTNIQ-IPN------GLRRLFLHDN-KLKTLP

S-SLLA-D-----F------------------------SRLH-LVSVFS----NPWRCD-

--CHAID-----------------------------------------------F--RRW

IV-----TNHEK-------------IKNV--------NQTLCGNVGESG--EYLL---KG

KAIWT-----------------LDETD-ICQT------FI--------------------

----------ELYFSVAFGFIAFFLIITVLKIVH---TRYKM-NINAWLY----------

------AHG--VIWVKEKDIDRDK--------------IFDAFLSFSH---K--DQEL-V

VTDI--ISVIE------VKQP----MT----RLCLH-YKHFKAGDF-IDQNIFN-AVQNS

KRTVIMLSKN---FLESEWC-IYEFRAAHL--QALKDKI---NR----VIIIKLGELP-D

DLHP--DIKM--SLENTTYLTW------------GEKYFWDKLFYVLP------------

----------------------T--------------------SGQ--------R--LSK

TTKVKADNYLMLHELK--------------------------------------------

------------------------------------------------------

>Pate_TLR_3

------------------------------------------------------------

---------MVFYHIIVLGIVLSEAKSVCKFKGSGVDCN---------------------

---------LSLSPTTSLLR----------------------------------------

------------------------------------CQSKCWLKPYSIRRSMVDTNVS-K

I-TFANPFGWEIFKEVIS-YECEDEIMFVDRAYNNSEEQCEKSNHAKYKLMNLLDVF---

----------EIDNIKKLELSHFINVS-IFTNIYP----QLFTLNIHHNVF---------

-----PGNISSSTIHF----TNPLRKLYL---THNQLQF-------------LPDHI---

-------FSSSYSLRKLDLSS--------------------------NNLT---EIN---

--------RRH---FQK-LKNLTS---------------------LNISRNK-LVCISPN

------------------------------------------------------TFSDLN

H--------------LRTLNLSHNDL-LTLTNSK--DKTDEDDSNEEVNSTSELQIHD--

------------------------------------------------------------

---------SNFLQHLQSLRNLDLSNN---------------------------IITILP

NNMFKNHSSLYYMDL----SHNFLRKCY-------------------------------R

FSAENYLHI------------------LNLSHNLIQI-----------------------

--------------------------------------------------------FGRL

CFKNLKNLKILNISSNLIK----EL-----------------------KLDFGKSV----

----------EILDA---------------------------------------------

--SHNK-----IATVLIS--GK---I------NIRVLILFD--NE---ITSFFSLS----

----HLSPL------------------------------LELLL---------------L

DYNKIYDTNFLK----------------------------------EYPYFQ-EVNHT--

---VTISVTFN---------NISTFDT------------SF----------MKDKNIKNH

--------------DEHALNAAHGI----------------------------------L

LDISFNPINCDCEMYHMK-----------------------------------------Q

YIVESNRHKLLSLINSENIKCAIPEPLKNH-SV----QFLHDD----------NFTCTF-

------------------KNYCPRFCTCG-------------------------------

------------------------------------------------------------

------------------------------------------------------------

--------------------------SRGKDRKIFVNCS---GLELH-------------

----EA---------------------D----KKPQFEVSI-LYFNK----NNLI---NL

S----SLNEW-TKLTDLWVDNN--RLGTLEGWI-IPP------NLLFLSVRGN-RLKSLS

N-NMIQ-F-----I-----------------------PTQKDFQLFFGL----NSINCD-

--CESET-----------------------------------------------F--KQF

LL----GHGEVV-------------KDVR---------EITC---NQKV--EEKV---VE

TPLIE-----------------MSE---PELCAQQITAKY--------------------

---------KQKVLTIFFCILLLIIIVTVGL------YIRQR-ELIHSFL---------Y

VHC--NKLF--CFYPEVADLEE-K--------------LYDAFVAYSS---S--DKRL-V

MNLL---NELE------QNAP----FF----KLCIH-ERDWIPGEI-ITETIVK-SVRSS

RRTIIVLSES---FISSPWF-KVELKVAIS-----QEQM---NP----IIVIMVDK---S

ISLNELNRELRDAISKRTYLEW------------GERWFWEKLRYAMP------------

---------------------------------------------------------HKV

------------------------------------------------------------

------------------------------------------------------

>Stpu_TLR_4

------------------------------------------------------------

--------------------------MATEVHSTKTRLC---------------------

---------LCVFGVLLSMI----------------------------------------

------------------------------------PATFSLSCNVPDLPCLCEGPTQ-G

S-GDHIEYHRITCFLTGE-WNVTIGVIPLT---RSLILSCSHGG----------NGT---

------------------EEPADLKED-LFHKFAG----VLQNLTMRRCKI---------

------GNLPTEIFTD----LILLQRLLL---TSVDLNG-------------ERLSA---

-------IGGIKTNASVKLYS--------------------------NKLH---SLN---

--------SDS---FGNYFSNVSS---------------------LDLYSNS-VQEIGNG

------------------------------------------------------TFAMFP

S--------------LRKLFLGNNTI------SV--V--KEGAFSGLQRLSELNVRGN--

------------------------------------------------------------

---------PSMFDGSWCL-----------------------------------------

---FKNVPFLTTLDV----SFTGLTNATQ-------FNCAPLVHLRRLLIHDNNLT---K

LDGSVFALMP--NITF-----------IDVSNNDLEY-----------------------

-------------------------------------------------------IHSSA

FHGGLNGLNHVDMSGNHLR----DF-------------PILAF----ESTPNIKSI----

----------NISYN---------------------------------------------

--YLRV-----IKKGTFS--GQ---A------SLQTIDLSF--NR---LHTIDMFG----

----FVALDN-----------------------------LTMID---------------L

RHNNF-AIFPEN----------------------------VVW---PFDIQPPHIPIKTF

LQGNNFNCGCH-WMFYIRRGEFKDTYP------------FFILSDNSTWTCKAPSPVANK

--------------PMMTLPLEDFW-------------------------------CPYY

NDEACRRGSCECYSRDVD------------------------------------------

---------EANVFFCNNNTMHSLPNFPANTFM----FECDGC----------LIDDQV-

--------------------TLQVGAFQA-------------------------------

------------------------------------------------------------

------------------------------------------------------------

--------------------------SARL---TFLVLQ---DIGLE-------------

----RI------------------LPGA----LQEFPNLQQ-LDVSY----NVLN---TF

S--DDIITNL-THLHTVDLSNN--RVNSLNSNT-FAT----NLNLTTVKLHSN-NLKTLE

D-GVFN-S-----T------------------------TYLE-VLTLHN----NPFVCN-

--CSLF--------------------------------------------W---L--KQW

LQ----SHLDVV-------------PQLY---------DVKCYVNSSNP---------DL

YPIIQ-----------------VADLDFGCYNPDVLTVQE--------------------

----------YNAVIVTSTLTLLFLMVGAVT------FRHRR-AIRVILY----------

-----TRYG----FHVLHDDDD-DDVVLDNVR---W--EYDAYIAYSD---E--DIQF-V

LENI--IPILE-----DDDNL----RY----KLCVR-HRDFPPGGC-IATTIVT-SLEAS

RRSIVLISRS---FLQDEWR-LLEFKTAHQ--RVLKDKR---NK---NLILVFLEDLT-K

DEMD---DDMRYYVTANAYLST------------TDRLFRENLLYEMP------------

---------------------------------------------------------RRP

LGEIHGDVDER-------------------------------------------------

------------------------------------------------------

>Pate_TLR_5

------------------------------------------------------------

-------------------MTNFLSCVSVSLIFITVNVMGCKNELFKNVAFNCELSIHTN

NQSLYCKSSILPVSNTFYILHLHQRKEIKITT----------------------------

------------------------------------QGFFESFMKSNFENDSAMYSCE-N

H-PMISDTDLIAEEFCIK-FD-------SCIEQVASFINTSFVQ----------QLN---

------------------ITNANIALL-SFQDL-M----NLTSISILNNNV---------

------SHVSKKLFKT----QTILETLVL---KNDCIQH-------------LQAGT---

-------FEAQDKLISLDLSI--------------------------NTLK---HFP---

--------KEA---LIN-LINLKY---------------------LNVSNNL-IETIANN

------------------------------------------------------DFTVLL

H--------------LNELDLSNNKL-TTIDANT--FN-SNGLLKVLHLSCNNFKVLP--

------------------------------------------------------------

---------ELLFSKLINLEKIYCRNC-------------------------------EL

HKLDEDLFVNNVKLL----RFDFSENNIS------------------------------V

LPSEIFRNQV--FLEI-----------INLSKNKISY-----------------------

----------------------------------------------------------LN

SFKNKPRLHALFMSENNLL----AL-------------NEDTF----LEAPSTFWI----

----------ILNKN---------------------------------------------

--NLTS-----INDNHIK--HL---R------NVGFLDLSN--NK---ITNIELGK----

----TTEVM------------------------------IRVLY---------------V

SNNLVKKFDV------------------------------------EWRSFV-YL-----

---EILDMDYN---------QIKYIEI------------PP----------CIPN-LKQT

--------------ITISF-KFNNITKVGLKSLLRDEKRAITDRTMAGCLFNGMAKNFID

LTHNPLHCDCDLYPLHNY------------------------------------------

-IVRKTGIKLDSFLNIENLTCNSPPRLRNE-LV----SKLPGD----------VFSCQV-

------------------VEDCPKSCTCG-------------------------------

------------------------------------------------------------

------------------------------------------------------------

--------------------------IRGQDDEIFVNCT---NRGLE-------------

----TI---------------------P----ENLPADTTV-LYFSN----NNLR---NF

Y--SLNSHSY-KNITEIHADNN--KITTLHGLK-MPE------NLKYLSLKEN-RIRDFP

E-SFSD-F-----L-----------------------NDHKDFKLFLSN----NNTYCD-

--CEKKT-----------------------------------------------L--KNF

LL----KNSASI-------------RDVA---------NITC-EIDNNG----TI---SI

LPLYK-----------------IPD---SILCPKFNGQNL--------------------

----------SFKITIWLSILFFTMITILLV--Y---YKQRQ-LILSFLY----------

-----IHCE--QLFQLLCEENE-QM-----EE---K--IFDAFIAYSSCNRD-------I

VMKL--IEELE------EKDP----FF----KLCIH-ERNWLPGQY-ISDNIIH-SVQSS

KRTIIVLSDD---FISSPWF-RLELRAAVF--KVSKDKM---NK----IIVILADN---S

TSLDGIDTELRHVITKRTYLVW------------GERWFWEKLKYAMP------------

------------------------------------------------------HKSREL

PEDRYITLNDRRGSSTTTLIESGANSVIPVVV----------------------------

------------------------------------------------------

>Pate_TLR_14

------------------------------------------------------------

----------------------------MIMESHRLTLL---------------------

---------IVACFTLCSAVTQKCSIKET-------------------------------

------------------------------------RADYFCVCDALDGTNGWTYTCI-D

N-MNLEAAFTVKYIVGRS-VIFQCGSEEPHYSLLLHQLDLEDIKTFAFKSCPLPAVPYDE

ILPFYDHSPVEQIKVERIKGNASFTNE-VFGNFTE----TLKTLILTDNGI---------

------ENISESLFSN----FTHLKYLSL---SDNRIKS-------------LQSRV---

-------FVGLENLTTLEVTN--------------------------NLLE---KLP---

--------FDV---FQD-QTLLEK---------------------LYLYKNK-LRELPDD

------------------------------------------------------LFKNLI

N--------------LKILDLADNQL-ILLPNRI--F----ETLSNVVSIRLRANWLG--

-----------------------------------------TVPEDLFRNCTRMQVIDLS

INRFMEPLSENLFHGLTHLENISIHDC------------------------NLTLIQEGF

FSRNPNLTHLNLEEN----SISSLPGKIF----------GNNSKLKELNVNFNLL-N--S

LPVDLFPNHL----KLEK---------VSLFRNNISS-----------------------

--------------------------------------------------------IPDR

LFKRARNVRTLVLGRNRIQ----NA-------------SYAVF----QDLPNLEEL----

----------DLSVN---------------------------------------------

--NLTY-----FKLDLNE--------------NLKKLDLSY--NN---LSKMPAIN----

----WIQHLQ-----------------------------LQKVN---------------L

EHNKLSFLEV------------------------------------PWLHST-NH-----

---PIINLANN---------KIRTVSV------------NN----------VLINDLGIK

--------------SEEASENYNNF-------------------------------VETR

IVLNSNPFLCDCHLFKFY------------------------------------------

---------KYIKESNGSPRSVRIDNIQNLSCH----EPFFNR----------KIVSLE-

----------PHEFTCDLQNECSSACHCY-------------------------------

------------------------------------------------------------

------------------------------------------------------------

--------------------------YRASDNANIVNCS---NHGLK-------------

----YL---------------------P----DHVPSNTSV-LYFSD----NLLT---NM

D--DFNQKRW-ENLTDIYLNHN--LVSNVDNWT-IPV------QLKGISLQGN-KLRHLS

E-QFMGFV-----S------------------------KAPHFHLALSS----NPWICN-

--CSAMK-----------------------------------------------F--KKW

LT----EHYKKI-------------GDVQ---------RITC---GNRLKLNNSL---VH

TPILT-----------------TPD---DILCPLDNWPDK--------------------

----------VHLITVSVICGVLALLLFIVIVLY---YRNKQ-TVIAYVY---------I

HMH--HVFT--CFFNEEDMDED-K--------------IFDAFVSYSC---S--DRD--V

AMEL--IEELE------KKDP----RF----NLCIH-ERNWIAGNQ-ISWNIFN-SVHNS

KRTILVISKA---FLESMWF-QVEFHTAYY--QMLEDKI---DR----LIIIVKGDLP-P

KENMDKDLQY--LLSTKTYLIW------------EEKWFWEKLKYAMP------------

----------------------H---------------------KK--------Q--LLP

NDVLALKDRPDSEKVKPIDNQIAILSSSDCKTKVHDPNRSTLHLVKSVNGN---------

------------------------------------------------------

>Drme_Toll

------------------------------------------------------------

--------------------------MSRLKAASELALL---------------------

---------VIILQLLQWPG----------------------------------------

------------------------------------SEASFGRDACSEMSIDGLCQCA-P

I-MSEYEIICPANAENPT-FRL------TIQPKDYVQIMCNLTDTTDYQQLPKKLRI---

------------------GEVDRVQMRRCMLPGHTPIASILDYLGIVSPTT------LIF

ESDNLGMNITRQHLDR----LHGLKRFRF---TTRRLTH-------------IPANL---

-------LTDMRNLSHLELRA---------------------------------NIE---

--------EMPSHLFDD-LENLES---------------------IEFGSNK-LRQMPRG

------------------------------------------------------IFGKMP

K--------------LKQLNLWSNQL-HNLTKHD--FE-GATSVLGIDIHDNGIEQLP--

--------------------------------------------HDVFAHLTNVTDINLS

ANLFRSLP-QGLFDHNKHLNEVRLM-----------------------------------

---NNRVPLATLPSR----LFANQPELQI------LRLRAELQSLPGDLFEHSTQITNIS

LGDNLLKTLP--ATLLEHQVNLLS---LDLSNNRLTH-----------------------

--------------------------------------------------------LPDS

LFAHTTNLTDLRLEDNLLT----GI-------------SGDIF----SNLGNLVTL----

----------VMSRN---------------------------------------------

--RLRT-----IDSRAFV--ST---N------GLRHLHLDH--ND---IDLQQPLL----

----DIMLQT------------------QINSPFGYMHGLLTLN---------------L

RNNSIIFVYN------------------------------------DWKNTMLQL-----

---RELDLSYN---------NISSLGY------------EDLAFLSQNRLHVNMT-HNKI

--------------RRIALPEDVHLGEGYNN-------------------------NLVH

VDLNDNPLVCDCTILWFI-----------------------------QLVRGVHKPQYSR

QFKLRTDRLVCSQPNVLEGTPVRQIEPQTLICP----LDFSDD---------PRERKCP-

-----------------------RGCNCH-------------------------------

------------------------------------------------------------

------------------------------------------------------------

--------------------------VRTYDKALVINCH---SGNLT-------------

----HV-----------------PRLPN----LHKNMQLME-LHLEN----NTLL---RL

P--SANTPGY-ESVTSLHLAGN--NLTSIDVDQ-LPT------NLTHLDISWN-HLQMLN

A-TVLG-F-----L---------------------NRTMKWR-SVKLSG----NPWMCD-

--CTAKP-----------------------------------------------L--LLF

TQ----DNFERI-------------GDRN---------EMMC---VNAE---------MP

TRMVE-----------------LSTND-ICPA------EK--------------------

----------GVFIALAVVIALTGLLAGFTAALY---YKFQT-EIKIWLY----------

-----AHNL--LLWFVTEEDLD-KD----------K--KFDAFISYSH---K--DQSF-I

EDYL--VPQLE------HGPQ----KF----QLCVH-ERDWLVGGH-IPENIMR-SVADS

RRTIIVLSQN---FIKSEWA-RLEFRAAHR--SALNEGR---SR----IIVIIYSDIGDV

EKLD---EELKAYLKMNTYLKW------------GDPWFWDKLRFALP------------

----------------------HRRPVGNIGNGALIKTALKGSTDDKLELIKPSPVTPPL

TTPPAEATKNPLVAQLNGVTPHQAIMIANGKNGLTNLYTPNGKSHGNGHINGAFIINTNA

KQSDV-------------------------------------------------

>Drme_Toll5

------------------------------------------------------------

-------------------------------MLTYLPVV---------------------

---------WLFFALLVLRSATGQIIPLPTF-----------------------------

------------------------------------CLGLSPQCTCAAEGNVVRFHCP-D

E-YAM-----LLEVSEPG-ASLYMSYYASTELQWLPRFNISSLV----------KIE---

------------------FDAYIFWPEKFLSDL-------LKTLGVQTVKT---------

------IIFRDRTLET----VVTRDVLNS---GNGYMET------------SQPENI---

---TTWHFGSVPGLKKFKFFS---------------------------------HVP---

--------ELQESIFHG-FDTLRD---------------------LHLS-----------

------------------------------------------------------------

---------------VNVTTLPGNML-ST----------VNGTLKTLTIESPGIVSFG--

------------------------------------------------------------

---------NPLLRELQQLRNLSLA-----------------------------------

--------LIHPFHE----RDKQLQPHFF----------GSMTNLEEVRLASATS----S

VNRSMFKGTN--KLQL-----------IKMNGNDDLM-----------------------

------------------------------------------------------------

------------------------------------ELPGEIF----LDQVNLKTL----

----------DLSCN---------------------------------------------

--AIVT-----LHEDVFK--GL---G------NLTLLDLSK--NR---LTNLSSTI----

----FAPLTS-----------------------------LNVLR---------------L

NKNSL-TAMSPS----------------------------------VFQDVV-SL-----

---NYIEMVNT----------------------------------------QFYGATLLM

--------------NYEAVVCTND------------------------------------

---------EACQYKSAE------------------------------------------

------------WQCDPRCICWVQRSVGSL--I----VDCRGT----------SLEELP-

------------------------------------------------------------

------------------------------------------------------------

------------------------------------------------------------

------------------------------------DLP---RTTLL-------------

----ST----------------------------------V-LKVGN----NSLT---SL

P-TVSEHSGY-ANVSGLFLSDN--NLTSLGSGDQLPD------NLTHLDVRGN-QIQSLS

E-EFLL-F-----L----------------------QEPNNTMTLSLSG----NPITCG-

--CESLS-----------------------------------------------L--LFF

VR----TNPQRV-------------RDIA---------DIVC-----------TK---QK

KSFQQ-----------------MEA---FELC----------------------------

---------PSYVLLISCVVGGLVIVICLLTVFY---LMFQQ-ELKIWLY----------

-----NNNL--CLWWVSEEELD-KD----------K--TYDAFISYSH---K--DEE--L

ISKL--LPKLE------SGPH----PF----RLCLH-DRDWLVGDC-IPEQIVR-TVDDS

KRVIIVLSQH---FIDSVWA-RMEFRIAYQ--ATLQDKR---KR----IIIILYRELE--

-HMNGIDSELRAYLKLNTYLKW------------GDPLFWSKLYYAMP------------

---------------------------------------------------------HNR

RVLKGQKKHAGPLI----------------------------------------------

------------------------------------------------------

>Stpu_TLR_91

------------------------------------------------------------

--------------------------MGIFIKGQFLLTI---------------------

---------LLNLISVCVCEEHVNSAIFTPYVIECPVV----------------------

------------------------------------PLPTGNSSCQCDPPNITVTDTD-S

LAAFQYVSCKLTPELISATNPD------PFYFPRSRRVYIDC------------------

------------------S----WNNSLSYSGF-------LMSAVKECGDIAEEVDPFGD

VDCFTPFNITADTYMG----LPSLQKLTL---ALEDGIS-------------IKPDA---

-------FSPLKNLEVLSLVQ--------------------------LDLK---SLH---

--------PEV---FRG-LTHLRQ---------------------LSLWDND-IRELPDG

------------------------------------------------------IFNDLQ

D--------------LQVLSLRSNSITHISRDLL--K--PLEFLNTLYLDDNNISSIH--

------------------------------------------------------------

---------ADAFRSMRSLEEVDLSRN------------------------------NLD

EKFSLSFLGTEKLLL----SYNSLT----------------------------------S

FTEDTVPGVR--GYTTS----------LDLSHNLISS-----------------------

------------------------------------------------------------

--------------------------------------ISESVFQTDELLSSLSYV----

----------VLRNN---------------------------------------------

--RLES-----LPTNVLR--WA---R------RLTFIDFSY--NS---LETLHDGL----

----FDIQSN---------P-------------------LGEQR---------------L

AGNQLVVRLGGN----------------------------------PFTCDC-RL-----

---TWFRIYDGQDVWISDRDDIECFSP------------PN---------------LNGI

--------------PLFSI-RPEHF----------------------------ECPLSDS

LCPEHCQCYEVMKDLGLQ------------------------------------------

-----TPRRVINVECESENLTRIPRGIPSNTSS----LDLTGN----------IWNTLR-

-----------------------KSMLDT-------------------------------

------------------------------------------------------------

------------------------------------------------------------

-------------------------RMPFL---SDLTLT---RCSIV-------------

----RI------------------ESGA----FRLLNSVLQ-LKLDG----NNFR---NI

T--KGTFQGL-TRLNTLYLNHS--SIRTIADGV-FLD----TPSLTYLYLHGN-FLTVLP

AIQHFP--------------------------------QSLE-IVSLQE----NPLTCS-

--CNLIS-----------------------------------------------L--QGL

VK-------YTL-------------EVKG---------NVTC-----------RE---RD

GTTVS-----------------LANLD-SSYC---RKHGT--------------------

----------SPLLAPLASLGAITGVLVVVLICV---FLYKKNKTLFQLM---------L

LRY----------FPQDLSEDDANK-------------PFDVFISYCQ---L--DDEF-V

LRYL--VPLLE-----TEDEP----SY----TICLH-HRHFVPGDT-IANNIVS-AVAQS

RRVILVLSDN---FLQSDWC-MYEFRMAHL--QALHDRR---NT----LLIITLGDIS-Q

DSLD---PDLKAYIRTTTYLESF-----DSKF--KNKLFLALKRGRSN------------

--------------------------------------------------------QRTA

RNQIKLVDFDGI------------------------------------------------

------------------------------------------------------

>Apja_TLR_2

------------------------------------------------------------

------------------------------------------------------------

---------MKNMTDTSLVT----------------------------------------

------------------------------------IERLTIYACNYLEIHESDLDAF--

----------------PSLSTL-----------EIRYGSLGY------------------

-----------------------ISPR-ALCGSISLSFLDFYVDAIADNITSPYPEAFIG

NTCNNDQTLAAGNLHSGETFLPMLQEMSL---SNFQFSE------------PIPENA---

-------FQNLLHLQILQLSF--------------------------ISFS---------

--------SNETFMSLDVLSNLES---------------------LQINHPTSYINLDFF

FG---------------------------------------------------NVLPKLQ

N--------------LKKLWLLETNIKTLSPVRD--VFSNNPLIEEISFFWNKLSFIH--

------------------------------------------------------------

---------ELTFRNLSNLRSLDLAVN---------------------------------

----KNLTSLQHDFLRGLINLEELILKDCSLKSLNEVDFSDVTSLKTLSAEENDI----T

EIPNIFSNFP--EQDVVLSRIQT----IKLQSNNIKC-----------------------

--------------------------------------------------------VKLF

TFSNLAHLSEIDLSLNLIS-----------------SVDDKAF----YNLENIESI----

----------NLAGN---------------------------------------------

--RLAV-----ISPKSLF--NL---Q------GLYLLDLKD--NM---LTYFPTFPCNVN

SRLGLNGLPS-----------------------------IRVLT--------------DL

RRNNLQCDCS------------------------------------MFEFLY--------

---HFIAFGFT--------EGCSLPSL------------YDREKYWPGVHYLVNQRFHQA

--------------ELLCFNPGGVF-------------------------------YIKD

ILDKPSLFLSDISNTSYF----------------------------------------SC

PRGCRCIRACQASVNIVSCRNQSFTDIPSDLDESTEYLFLQNN----------RIESIP-

-----------------------KSSLDV-------------------------------

------------------------------------------------------------

------------------------------------------------------------

--------------------------VPNL---QYLNLH---DNRVN-------------

----HI---------------------E----KGSLDQLLD-VDLSD----NRLT---EL

P--RGTMMSL--SLKYLNFSKN--DISSLYAAS-FAG----LPFLEILDLSHN-EITVFP

S-GLFD-N-----L------------------------TKLT-SVFIGG----NPLNCT-

--CDMLY-----------------------------------------------L--SKW

YRQASFKPNGTR-------------PDTD-------FGQIEC---VPFA---------NG

TQLSK-----------------WIDDH-ETSCLSVSEEPV--------------------

----VVTGSNSALVSVLLIIITILTVVFITTLVI---YRYHL-EFSVMVY----------

-----ARTG--FRCFQISREDESSK-------------DFDAFISFSN---Q--DNDF-V

LNDI--LPRLE------NHSP----PW----KLCIH-HRDFAVGES-IATNILN-AIERS

KRTIIILSTQ---FLESEWC-SYEFRAAHS--QALRERS---QK----ILLVMFNDVD-K

STLD---KELRAYISANTYLRT------------DDTMFWSKLKYALP------------

---------------------------------------------------------EPI

NSTEESTL----------------------------------------------------

------------------------------------------------------

>Aslu_TLR_32

------------------------------------------------------------

--------------------------MAKLKFLEISYNH---------------------

---------IRNLSDSLFVL----------------------------------------

------------------------------------PNLKRLHINYNKIESLDGSPFK-N

L-RELETLLLFGMSPLKN-VSL------PGPGGETWSLRGSLRF----------------

-----------------------VYPS-SFEGL-S----NLKELKLAGNSI---------

------RLILNETFGQ----LIRLEHLNL---SLGLISA-------------IKNDG---

-------FKGLQSLQSLDLSY--------------------------NNLS---YIR---

--------GSL---FKN-LQTLRI---------------------LNLEGNF-IQSLSEI

------------------------------------------------------TFLGLQ

L--------------LTRLDVGYNRL------ID--V--QTDTFRSLESLEWLSLYHN--

------------------------------------------------------------

---HIRSI-EGFLSGLSSTRCEEIDIS---------------------------------

---FNNVSSLDATVL----TTLALSGR-----------------IVTLNLSHNAL-SEVY

VTTSLYAVSG--YYPLMLV--------LDLRSNRFES-----------------------

------------------------------------------------------------

-----------------------------------------------IPFQLDKYS----

----------FFS-----------------------------------------------

-----------SPRIVID--KT---D------AERYLVVGQHDDV---SLLIHPNP----

----FTCDCR----------------------VYEL---LQNIE---------------I

AQTGALYTKT------------------------------------DFTEITCHMPKSLR

GI-KLVDLSSS--------------QA------------WCVKNDPYNCPATCNCMVQGA

--------------RNSTISPWNER-----------------------------------

---------VDCSRRDLL------------------------------------------

---------------------NIPSDIPSLTTI----LHLERN----------NIGVVY-

-----------------------RAVFTE-------------------------------

------------------------------------------------------------

------------------------------------------------------------

--------------------------CRIT---RELYLS---DNNIT-------------

----KI------------------ELMA----FSHLKSLEI-LYLDG----NNIN---DI

T--GQDFLSL-VNLRELYLNRS--RVRTVGSDA-FRD--L-P-SLNVLHLENN-LLKDLP

K-DLFS-T-----S------------------------ENLH-HLSLTG----NSFQCD-

--CNIL--------------------------------------------W---F--KYW

MK----NKESI--------------LVGS---------NITC---THEQ----TH---SR

VTILS-----------------LSE-N-ILGCDHAAADRR--------------------

----------SKITIGLSVVVVLLSMLMILGIVL---FKKKK-DLQVYIY----------

-----ARYG--WRFKEEAEDMD-------------K--PYDAFLSYSK---H--DLDF-I

VNEL--LPGLE------NREP----PF----RVCLH-HRDFIPGIP-IADNILN-AVEES

RRTIVVISRN---FLESDWC-QLEFQAAHM--QMLQDRA---TR----LIIVLLEDIP-V

DDAP-PDIKH--YLKTKTYLKW------------GDERFWERLVYVMP------------

------------------------------------------------------R-PRPA

GQLVQNMEEEQLEVIRGQEQLEMLEEQREMEIEEHEQLGIDEQQLEIEG-----------

------------------------------------------------------

>Brfl_TLR_10

------------------------------------------------------------

------------------------------------------------------------

------------------------------------------------------------

-------------------------------------------MANSSVLAHGLGTCVEH

G-----------------------------------------------------------

----------------------------DVTALMA----LLDRVGVDAKDE---------

---------ADTAYRP-----NGVDMLYL---PQRWCDR---------------------

------------------------------------------------------QVC---

--------DCG-------------------------------------------------

------------------------------------------------------------

---------------LYELMLN--------------------------------------

------------------------------------------------------------

------------------------------------------------------------

------------------------------------------------------------

---------------------------LDVAKQGAL------------------------

------------------------------------------------------------

------------------------------------------------------------

------------------------------------------------------------

-----------FTKTDFR--------------DMECAFPDA-------------------

-----------------------------------------------------------L

SGRRVVDLKPSE----------------------------------LWCSE---------

---ECYKRPHY-----------------------------------------FCSCYEHA

------------------------------------------------------------

---------DDISNVTII------------------------------------------

-------------------------------------LHLEGN----------QLIVIS-

-----------------------QAVLPQ-------------------------------

------------------------------------------------------------

------------------------------------------------------------

--------------------------LLMI---RELYLN---DNNIS-------------

----YV------------------GDQA----FKNLLSLEI-LRLDG----NNIS---EL

N--STVFKSL-SNLRELYLNHS--GVQYLAADM-FQD----LASLQELHLENN-WLQSLP

E-NMFD-G-----L------------------------KKLR-SLSIHG----NPLHCE-

--CDVL--------------------------------------------W---F--TNW

LR----SRESFL-------------SQGH---------NVSC--LVNTK---------VK

RDILS-----------------LSS-A-QLDCNGLQAAQA--------------------

----------RTRLIVGLSIPLVLVTIILVCVII---IVKRKEDIQVYLY----------

-----ARYG--WRFQEEEEDED-------------K--EYDAFLSYSQ---H--DLDV-V

MHDV--LPALE------NREP----PY----RVCLH-HRDFLPGIP-IAENIAN-AVNSS

KRTIILLSNN---FLESDWC-QFEFQAAHA--QMLQDRA---NR----VIVVLLDDVP-A

ENAP---PDIQHYLNTNTYLKW------------GDERFWERLIYVMP------------

------------------------------------------------------R-PRQH

AQDMDAQNMDGDQLVLVELDHNG-------------------------------------

------------------------------------------------------

>Brfl_TLR_6

------------------------------------------------------------

------------------------------------------------------------

------------------------------------------------------------

------------------------------------------------------------

------------------------------------------------------------

------------------------------------------------------------

------------------------------------------------------------

------------------------------------------------------------

------------------------------------------------------------

------------------------------------------------------------

------------------------------------------------------------

------------------------------------------------------------

------------------------------------------------------------

------------------------------------------------------------

------------------------------------------------------------

------------------------------------------------------------

------------------------------------------------------------

-------------MT---------------------------------------------

------------------------------------------------------------

------------------------------------------------------------

------------------------------------------------------------

------------------------------------------------------------

------------------------------------------------------------

------------------------------------------------------------

------------------------------------------------------------

------------------------------------------------------------

------------------------------------------------------------

------------------------------------------------------------

------------------------------------------------------------

--------------------------------FSNFSSLEI-LRLDG----NNIS---QI

N--SKVFQSL-YNLRELYLNHS--GVRLLGEDT-FHD--L-E-SLQKLHLENN-GLQSLP

E-NTFA-G-----L------------------------KNLR-SLGIHG----NPLNCD-

--CDVL--------------------------------------------W---L--ANW

LR----SRRFLL-------------SKGF---------NVTC--LANTK---------VM

RNVLS-----------------LSS-A-QLGC------DD--------------------

---LQAAQARARLTIGLSVSLVLVTFISVCMIII---ARHKD-AIQVYLY----------

-----ARYG--WRFREEVEDED-------------K--EYDAFLSYSQ---H--DLDL-V

LHDV--LPALE------NREP----PF----RVCLH-HRDFLPGVP-IAENILN-AVSAS

KRTIILLSNN---FLESDWC-QLEFQAAHA--QMLQDRA---NR----VIVILLDDVL-T

ENAP---PEIQHYLRTNTYLKW------------GDDRFWERLIYAMP------------

------------------------------------------------------RRRPHA

QDMYMDGDQLAMVELDHNR-----------------------------------------

------------------------------------------------------

>Brfl_TLR_2

------------------------------------------------------------

-------------------------------MAKALPVF---------------------

---------FLIICAISGDV----------------------------------------

------------------------------------AMDTVDNGSSGDESSPQ-AFCN-C

I-RTGPTSRQCNCTDLSN-LTN------AQNRLDVSGYLSDDDDDDLFVVSEITVRC---

------------------RQKSSTNTSGLFDFLPP----SIKSLTLINCVQ---------

------QYIGKEVFYG----IPNVEDLAIVNFPPGKRND-----------SVFFSYF---

-------YDNSSRTLELDPEN--------------------------NFLQ---SLS---

--------PTT---FEN-MPYLLD---------------------LNLANNL-LTTVTRE

------------------------------------------------------NVSPLL

N--------------LRTLDVHGNQ-------LE--T--IDEMFHGITSDVCEKIDAS--

------------------------------------------------------------

------YN-KVWLLAISSLEILGIV-----------------------------------

----GKTVNVDLSHN----NLQILYHT--------------------------------P

PESYISEDVL----HLSLS--------LDLRWNQFAS-----------------------

------------------------------------------------------------

FPLELATYRSFSRSRISIR----EI-------------SGQKY----GDISLLMTQ----

------------------------------------------------------------

---NPL-----ICDCALY--EL---I--------VNLDVAK--QG---VLYTKTDF----

----QDMVCA----------------------VPDELRGRRVVD---------------L

RPSELWCSEE------------------------------------CYNRFY--------

---ACFCYEHE-------GEMFKSPQP------------WCFPEHNACPSECSCS-FQGQ

--------------LHSATAPYNEL-----------------------------------

---------VNCAGRNLS------------------------------------------

--------------------SIPVEISNVT-TI----LHLEGN----------QLRVIS-

-------------------QTVLPELLMV-------------------------------

------------------------------------------------------------

------------------------------------------------------------

---------------------------------RELYLN---DNNIS-------------

----YV----GAMA------------------FSAFNSLEI-LRLDG----NNIS---NI

G--STIFKSL-SNLRELYLNHS--GVRYLSVDT-FHD--L-A-SLQKLHLENN-RLQSLP

E-NMFA-G-----L------------------------KKLR-SLSIHG----NPLNCD-

--CDVL--------------------------------------------W---F--ANW

LR-----------------SRAF--LLAQ-------GHNVSC--LTKTK---------VA

RDILS-----------------VSS-S-QLDC------DD--------------------

---VQAARARNRLIVGVSIPMGLVIIILVCVIVI---VRRKE-AIQVYLY----------

-----ARYG----WRFREEEED--------ED---K--EYDAFLSYSQ---H--DLDV-V

LHDI--LPALE------NREP----PF----RVCLH-HRDFLPGVP-IAENIAT-AVNSS

KRTIILLSNN---FLESDWC-QFEFQAAHA--QMLQDRA---NR----VIVVLLDDVP-A

ENAP---PDIQHYLNTNTYLRW------------GDERFWERLIYVMP------------

---------------------------------------------------RPRP-HAQD

EDGDQLAMVELDHNR---------------------------------------------

------------------------------------------------------

>Aslu_TLR_34

------------------------------------------------------------

------------MSNEVFPHTPFSQKADHNTGVMVGALR---------------------

---------VFLIFVMCSISRGAAMEMPE-------------------------------

------------------------------------MQHNCNCSQTVSGTLLT-CACP-D

L-YTLTSVQERAYVAMF--LSS------PL---EVTTFEVTCAH----------RSV---

------------------K----SPNA-VFDNLPA----SVTKLSLTNCFW---------

------QNISKEIFYG----MPRVEELTI---TNIWSLGDEDLLTNQTDTVELDPEL---

-------FLPSPQLQKLTLGF-----------------------LFLDALPK--ALY---

--------QAV-NGTYP-LQNLRS---------------------LILTGNR-IPYLKPE

------------------------------------------------------HLQHLQ

N--------------LRSLNVQYNS-------IS--N--LKMSFPFLPNLQELYTNLN--

------------------------------------------------------------

--YINCLD-GSPFQNLSNLQSLDLSGQ----------FSTNTNPSKKGGIGALGFIFPSS

FSGLFKLKSLRLALS----YVHSVQNGTF----------EGLVQLEQLNISDGLI-G--N

IEVLGFQGLG----ALKS---------LDLSYN-SLS-----------------------

---------------------------------------------------T----ILPA

AFNGLNSLLTLHLQGNALL----YL-------------IGSTF----ESMPSLSEL----

----------YLANN---------------------------------------------

--RFTV-----LSGDIFK--PL---I------NLKKLDVHG--NQ---LITAEDMF----

----FGITSH---------------F-------------CEEID---------------A

SHNELATFDLSS-------------------------------L--SNLGIPGKT-----

---IQVDLSHNELLTVYTSSFYFSLNA------------FHLSARVDLRWNLFPGLPLEF

--------------AKYGSFNQSFITM-----------------------------FDVS

LLITQNPLICDCRLYELL-----------------------------------------L

NLDVAQKGALYTQTDFQDMRCTFPATLHGR-RV----TDVHPS----------ELWCLE-

-----------------ECYNQPDNCFCL-------------------------------

------------------------------------------------------------

---------MDAGEMLTTGNCKTKELTCQSECSCWMQGQLHSSTAPWNQLVDCSGKNLSS

VPYGISNVTTYLLLQDNSLTAISQAVLPELLMIRELYLQ---DNNIT-------------

----YV------------------SPTT----LRNFTSLEI-LRLDG----NDIS---EI

N--STTFKSL-SNLQKLYINDS--GLTYIAPGT-FHY--M-S-NLKELHLENN-RLKVLH

E-NMFD-G-----L------------------------DSLE-LLGIHG----NPLECT-

--CDLL--------------------------------------------W---F--TNW

LR----TAAFLL-------------SKGH---------NVTC--ADTSS---------ST

RDIMS-----------------LSS-A-QLGC------DA--------------------

-------EARNRLIVGLSTALALVIIALILVIVI---VKRKE-DIQVFLY----------

-----VRYG----WRLREEEED--------DD---K--EYDAFLSYSQ---H--DLDL-V

VQDV--LPVLE------NREP----PF----RICLE-DRDFLGGAP-IFDNIFN-AVNVS

KRTIVFLSNN---FLKSELC-QFEFQAAHA--QTLQDKA---NR----LIIILLDDIPAE

NAPP------------------------------DIQHY---------------------

------------------------------------------------------------

------------------------------------------------------------

------------------------------------------------------

>Aslu_TLR_35

------------------------------------------------------------

------------------------------------------------------------

---------HQPVNGTYPLQ----------------------------------------

------------------------------------NLRSLNLLGNTIPYLKA-EHLQ-H

L-LNL----------------------------RSLNVQLNRIS----------NLT---

------------------S---------TFPFL-P----HLQALYINWNFI---------

------NSLDGSPFQN----ISNLQSLDLSSQFSANHNA--SRKYDKSALEFIFPSS---

-------FRGLSKLKSLRLTV--------------------------NRIQ---SIQ---

--------NGT---FES-LMQLEQ---------------------LNISDGL-IDNIELL

------------------------------------------------------GFQGLG

A--------------LKSLDLSYNNL------RT--V--LPDVFNSLNSLLTLHLEGN--

--------------------------------------ALLYLSGTTFESMPSLSELYLG

NNNLTVLP-GDVFKPLLNLKKLDVH-----------------------------------

---GNQLVVVEDMFF----AISSCEE----------------------------I-DA-S

YNELVAFDLS----SLNNLGIPGKTIRVNLSHN-KLI-----------------------

------------------------------------------------------------

----------ILLVSEAYL----SV----------------------HAYSLSVRV----

----------DLRWN---------------------------------------------

---LFP-----GLPFGLA--KY---G------SFNQSSITA--TS---FDLSLLIT----

----QNPLIC----------------------DCRLYELLMNLD---------------V

AQKGSLYTKT------------------------------------DFQDMR-CA-----

---YPWTLQDR---------RVTDVQP--------------------------NE-LWCL

--------------EKCYNQPYNCF-------------------------------CFMG

ADAKEVITTTNCKTKELT------------------------------------------

-----------------CPSECSCWMQGQL-HS----STAPWN----------QLVDCY-

-------------------GGNLSSVPAG-------------------------------

------------------------------------------------------------

------------------------------------------------------------

-------------------------ISNVT---TTLLLD---GNNLR-------------

----VI----NQAV------------------LPELFILRE-LYLQD----NNIS---YI

S--PTTFSNF-TSLEILRLDGN--DISEINSTT-FKS--L-S-RLKELHLENN-RLKGLH

E-NIFD-G-----L------------------------KNLP-LLGIHG----NPLERT-

--CGLL--------------------------------------------W---F--TNW

LR--------SS-------AAMF--LKGH---------NVTC--TDTSA---------VT

RDILS-----------------LSS-A-NLGC------DD--------------------

---VRSEEARNRLIVGVLSALAIVIMILVCVIVI---IKRKE-DTQVYLY----------

-----ARYG----WRFREEEEE--------DD---K--EYDAFLSYSQ---H--DLDL-V

VQDV--LPVLE------NREP----PF----RVCLH-HRDFPPGAP-IADNILN-AVSAS

KRTLVLLSNN---FLESDWC-QLEFQAAHA--QMLQDRA---NR----VIVIMLDDIPAE

NPLR---------------------------------------TFSTT------------

------------------------------------------------------------

------------------------------------------------------------

------------------------------------------------------

>Neve_TLR

------------------------------------------------------------

-----------------------------MKGSILRQVT---------------------

---------QCFCNAWVSVL----------------------------------------

------------------------------------FLRALLIAGAPSRDENCKKECA-N

L--RMPSTFRSPTGQIPM-FFT------KCEIKGGAECSLDLGP----------WIQ---

------------------P----LANS-STVQY------YLAIVCRSSTRI---------

------------IFCN----SPEVKRKNV-----------------------ILFYQ---

-------MAGPCSVTVHDVSV---------------------------------------

--------------LGN-ATDYRV---------------------QLFTHGAELLYADTE

------------------------------------------------------SITGLR

N--------------IGTFSLQSS--------GT--G--IPRILTGFEWPRMAEVLLS--

------------------------------------NLSITEIPEQFKTAMPRLQALDLN

NNSLTRPPDFPWSHKPLSLPRNLSR---------------------------------LP

VFNHHYQEGSVVQPR----LYRRFLVLDY-NQIRNLSQYPFTGHLQKLSIKGNGL-R--V

IGGSCFSNLS--GVNI-----------IDLSNNEIRD-----------------------

--------------------------------------------------------FPEQ

LFRGQGSMLELRFNHNFLS----TL-------------PNRVF----TDMKRLKRL----

----------YLNNN---------------------------------------------

--RLQR-----LQAGLLY--GN---E------EIETLTLND--ND---LTEIENNA----

----LPENSN---------------T-------------LKTLT---------------L

QRNRL-TRVP------------------------------------RAVFLLRNL-----

---ESADLSSNAITFGGILDVLDSVTA------------DQLFYNLRRSASSSDNQLKST

--------------KVELNLANNGISSIDIGSLNKTQLGKLKVIL-----------RVYH

IDLRDNPLICNCKLTALF----------------------------------------RL

LKRLTADYPDVTHAQFDSWICSQPTRLRNV-AL----LRVPEN----------QFQCIM-

-----------------DLENCPRECTCA-------------------------------

------------------------------------------------------------

------------------------------------------------------------

--------------------------VREIDQTVLVDCS---ERGLH-------------

----RL---------------------P----FKMPAGELE-VNLRG----NAIR---EL

P-----WRHYLGNITVLELSNN--EIKELNMTF-VDS--L-A-RVVNLAINDN-KLKYLP

R-GVTNLT-----A-----------------------REGFR-SLSISH----NFFVCD-

--CYAS--------------------------------------------W---M--RDW

LA----NNTDKI-------------EDTS---------SILC----ASG----RL---EG

LPIIS-----------------VPLSDFNCSAYRPPVPGP--------------------

-------ITDGLSLLLAIVLAVLLVLSVVAFVMT---YCFRW-EMKILMY----------

-----THFN----WHPFDRVDD-TDVSK----------IYDTFISYSS---Q--DASW-V

RETL--QRTLE------SHVP----PY----RLCIH-DRDFEIGAS-IHDNILN-SVRLS

KRMIMVLSNH---FIASEWC-RLEFRAAHQ--KVLEDRT---NY----LIIILFDDVD-P

STLD-DETKL--YLRTNTYLSV------------SNKWFWQKLFYALP------------

------------------------------------------------------K--PLA

PPQSYEGHVEMSKV----------------------------------------------

------------------------------------------------------

>Orfa_TLR_2

------------------------------------------------------------

------------------------------------------------------------

------------------------------------------------------------

------------------------------------------------------------

------------------------------------------------------------

------------------------------------------------------------

----------------------------------------------------MPRGL---

------------------------------------------------------------

------------------------------------------------------------

------------------------------------------------------------

------------------------------------------------------------

------------------------------------------------------------

------------------------------------------------------------

------------------------------------------------------------

------------------------------------------------------------

------------------------------------------------------------

------------------------------------------------------------

------------------------------------------------------------

-----------IE-----------------------------------------------

---------------------------------------LNLMS----------------

------------------------------------------------------------

------------------------------------------------------------

------------------------------------------------------------

------------------------------------------------------------

------------------------------------------------------------

------------------------------------------------------------

------------------------------------------------------------

------------------------------------------------------------

------------------------------------------------------------

---------------------------------------------------NDIR---DI

P----AFPYL-ENVTVLKMTNN--KVERLKAST-VEK--L-K-RVEILLIDAN-NLTTLP

R-EIES--------------------------------LNFT-TLALEQ----NLFKCD-

--CTTK--------------------------------------------W---M--KHW

LV----KNRRR--------------IRNI--------EKVFC---NSEH----AL----G

RAIYS-----------------LPD-D-EFICATIKEKNT--------------------

------GKTVPIGTIAACTLGGLLALILIVGIVL---YKYYR-EVKVFMY----------

-----THFN----WHPFDRIDD-SD-----PN---K--IYDAFISFSG---N--DFDW-V

KTTL--QERLE------NHDP----PY----KLCFH-HRDFPVGEP-IVENIFK-SVDQS

KRMLVVLSSS---YAKSDWC-LMEFREAHR--KVLEDRM---KY----LIVILFDDVD-T

TELD---EEFKLYLRTNTYLSV------------SDKGFWQKLYYAMP------------

-----------------------------------------------PPSSTESVEERSF

ENVALSGNIDSSAEILQQEALEMAHNTADTIVLVEQ------------------------

------------------------------------------------------

>Orfa_Toll

------------------------------------------------------------

-----------------MFVKSQTALIQSNLHILVLDQR---------------------

---------HTVLLATSLVFDTKGSLIIFS------------------------------

------------------------------------CLVLFAIEATGQQRRRF-LHCG-A

S-FCLLKRSMSSFFETGN-GTL------IKEQAVICVLKSDK------------------

-----------------------VRDR-CVVDISL----ILKTITTPQDVV---------

-----------LHFAA--VCLTPMEIAFY---NSLNATK---------------------

-------KNAIFYLQIKGHCS---------------------------------FSA---

--------DGI-SHWGK-ATDFRV---------------------FYLMENSTLLEGNKT

LA--------------------------------------------------NNSRRALE

N--------------IGTLMIDKSKL-RTLPKMF--S--STKVWPRMAEVVFSKLQLT--

-----------------------------------------SIPPELNTTMPFLQSLELA

NNKLTTPP-PFPWCKATLKLPRGLQ--------------------------------RTP

TGNHHYQFGTNVRPN----IYRRFLDLSY-NNIEDLSTHNFRGFLNKLTLEGNGL-K--V

IGTSCFRNLK----GIHV---------ISLSKN-KLK-----------------------

---------------------------------------------------S----LPSE

LFQGQDSLLELRLDHNNIS----II-------------PNDLF----KTVTQIKRI----

----------DLHSN---------------------------------------------

--KLSC-----IPQELFS--KL---K------NIKILHLED--NH---ITQVHDKA----

----FSIDSS---------------S-------------LQNIY---------------L

QKNKI-SRIP------------------------------------LTLLLQRHA-----

---VKIDLSFNQLTFQDFNRLIQELDL------------ETFLYHHRHTASSSQMRLQES

--------------LKSISFAHNKFTTINIEAFNRTEELTFEYLL-----------RVYE

IDMSGNLLLCDCKILLLS----------------------------------------RW

LRALVQRHTRIRNEQFQTWKCAAPTELKGK-PI----LSVDEN----------RFKCQR-

-----------------NLENCPHGCLCL-------------------------------

------------------------------------------------------------

------------------------------------------------------------

--------------------------VRALDGTVVIDCK---GRNLT-------------

----AI---------------------P----PKVPSGRIE-LKLED----NNIR---EI

P----PYPYM-ENVTALYLTHN--KIQVLNKST-VRR--F-T-RIKVLFIDSN-KLTYLP

K-NIEN--------------------------------LNFT-SLALHH----NFFKCD-

--CTTL--------------------------------------------W---I--KHW

LQ----RKQSK--------------ILHI--------KNVLC---NSEG----ST---QG

KAIYT-----------------LPN-E-EFVC------KK----------------NKKD

IPTTQSITKDKTFKIIALTLGGALVLTFIAFIVA---YKYRG-EMKVLMY----------

-----THFN----WHPFDRVDD-SD-----PR---K--IYDAFVSYSG---S--DHQW-V

VNTL--QERLE------HHDP----PY----KLCIH-HRDFVVGAP-IQENILN-SVDQS

KRMLMVLSRN---FLKSEWC-LLEFRAAHR--KVLEDRM---NY----LIIILFDGIN-M

DELD---DEMKLYMRTNTYLSV------------SYKWFWEKLYYAMP------------

---------------------------------------------------------QST

DRQFRARDLSSISSNAGMQYSTETVFKNEAYLKVTDILE---------------------

------------------------------------------------------

>Acdi_TLR_1

------------------------------------------------------------

--------------------------------MWTDTIL---------------------

---------IVFLICALTTSGLETKRIVDCKQSRCVLR----------------------

------------------------------------EYQRSKLRSKSKTLKIQEIQCS-I

Q-TNQRGSKCVVDIHAVL-TAV------QTSQDTILYFNATCLT--------PVNIT---

------------------F----KNSQNAIKKN------LISCLDLRGHCS---------

-ISTSDMSKWGNATDF--RVFNMLDNAVL---VNDNSVQ-------------ISNRP---

-------IPGLENISRLMLYK--------------------------TQTKKIPEIF---

--------KKY-SSWPS-MAEIAF---------------------VDLQLTSIPTELKTT

MP----------------------L-----------------------------------

---------------LQSIILQHNN--------L--T--KPPDFPWYNDTLNLPRGLR--

---------------------------------------------------------RNT

FYDLYYED-RKIVPPTIYPRYLDLSFN--------------------------MIEDLSA

HEFRGRLNFLYLKGN----RLKSIGRHCF----------RTLKGVEMIDLSHNNL-Q--H

LPSQLFRQLN--DLLLLR---------LNFNQISNIP-----------------------

----------------------------------------------------------KD

LFNSQKQIKRIDLDHNKIK----SI-------------PKGLF----SELNYMEKL----

----------HLQNN---------------------------------------------

--HITA-----IDEEAFA--TD---S-----SSLSEIHLQN--NQ---FTRVPISL----

----LLLRQ------------------------------ARHID---------------L

SFNRL-----------------------------------------TFQDLDKTI-----

---AEMDDTFV-------SQFYETHFF------------LRKSVTVIQISLAHNH-FTTI

--------------DIAGMDQSRRIRFEYFL-------------------------RVYE

INMTGNPLLCDDKILGFV-------------------------------------RWLKQ

WMQNNIGLRVVRPQQFSTWKCAAPMAIKDK-LI----LSLRED----------QFRSNR-

-----------------NLSNCPKECTCY-------------------------------

------------------------------------------------------------

------------------------------------------------------------

--------------------------VRSMDETVIVDCK---EKDLV-------------

----AM---------------------P----RSVPDGQTE-MFLQS----NNIR---EI

P----SYGYL-ENVTSLYLSHN--QIQRLDEKT-IDR--L-K-RIETLFIDSN-KLTTLP

R-NIEN--------------------------------VSFT-KISLQH----NFFRCD-

--CETK--------------------------------------------W---M--KQW

LL--------RE-------------EAHV-----DNIENILC----HSD----HV---QG

KAISR-----------------LPD-E-EFLCLEGKNDKS--------------------

----QNLAEPPAFKITAYTLGGLLFVSLVAFAVG---YKFRS-EAKVFMY----------

-----THFN----WHPFDRIND-LD-----PN---K--PYDAFISFSG---N--DYEW-I

CNTL--CVRLE------NHDP----PY----KLCLH-HRDFLVGAP-IQQNIFD-GIERS

KRMIMTLSKH---FVRSEWC-LLEFRAAHQ--KVLEDRI---NY----LIIILFDDVD-M

AEVD---DEIKLYMRTNTYLSV------------KNKWFWEKLFYALP------------

---------------------------------------------------------QNT

NRETEAKDCHRSAHVNPTADEDNDLSSEEAQV----------------------------

------------------------------------------------------

>Acdi_TLR_2

------------------------------------------------------------

------------------------------------------------------------

------------------------------------------------------------

------------------------------------------------------------

------------------------------------------------------------

-----------------------MPRS---------------------------------

----------------------------------------------------VPDGQ---

------------------------------------------------------------

------------------------------------------------------------

------------------------------------------------------------

------------------------------------------------------------

------------------------------------------------------------

------------------------------------------------------------

------------------------------------------------------------

------------------------------------------------------------

------------------------------------------------------------

------------------------------------------------------------

------------------------------------------------------------

------------------------------------------------------------

------------------------------------------------------------

------------------------------------------------------------

------------------------------------------------------------

------------------------------------------------------------

------------------------------------------------------------

------------------------------------------------------------

------------------------------------------------------------

------------------------------------------------------------

------------------------------------------------------------

---------------------------------TELFLQ---SNKIE-------------

----EI------------------------------------------------------

----PSYSYL-ENVTALYLSHN--NIERLNEKT-IDR--L-K-RIEILFIDSN-KLTTLP

R-NIEN--------------------------------VSFI-KISLQH----NFFRCD-

--CKTK--------------------------------------------W---M--KHW

LL--------RQ-------------EAHI-----DNIENILC----HSD----HV---KG

KAISR-----------------LPD-E-EFVCPAPGKSGH--------------------

------QAESPAFKITAYTLGGLLLVFLVAFAVG---YKFRG-EVKVFMY----------

-----THFN----WHPFDRIND-LD-----PN---K--IYDAFISFSG---I--DYEW-I

SNTL--CARLE------NHDP----PY----KLCLH-HRDFLVGAP-IQQNIFN-GIEKS

KRMIMILSKN---FVKSEWC-LLEFRAAHQ--KVLEDRI---NY----LIIILFDDVD-M

AEVD---DEIKLYMRTNTYLSI------------KNKWFWEKLFYALP------------

---------------------------------------------------------QNS

KRETEAKDCHRSAHVNSTADGDNNLSSERAQV----------------------------

------------------------------------------------------

>Acdi_TLR_3

------------------------------------------------------------

------------------------------------------------------------

------------------------------------------------------------

------------------------------------------------------------

------------------------------------------------------------

----------------------------------------MEVLHLENNHI---------

------TAIDDEAFAT---DSSSLLEIHL---QNNKFTR-------------APISL---

--------LLLRQARHIDLSF--------------------------NRLT---------

------------------FQDLDK------------------------------------

------------------------------------------------------TIAEVD

D-----------------------------------------------------------

------------------------------------------------------------

------------------------------------------------------------

-------------------KFVYQFLNTH-------------------------------

---------------------------FPLGQSVIQI-----------------------

------------------------------------------------------------

------------------------------------------------------------

----------SFAHN---------------------------------------------

--------------------------------HFTTIDIEG-------MNQTRRIR----

----FEYFLR-----------------------------VYEIN---------------M

TGNPL---LCDG-----------------------------------------KL-----

--------------------------------------------------------LGFV

--------------RWLKEWMQNNT-----------------------------------

----------GLRVVRPQ------------------------------------------

--------------QFSTWKCAAPMAIKDK-PI----LSLRED----------QFISNR-

-----------------NLSNCPKQCTCY-------------------------------

------------------------------------------------------------

------------------------------------------------------------

--------------------------VRSIDGTVIVDCK---GNDLV-------------

----TM--------------------------PRSVPEGQTALFLQS----NNIR---EI

P----SYGYL-ENVTALYLSHN--NIERLDQKT-IDR----LKRIEILFIDSN-KLTTLP

R-NIKN--------------------------------VSFT-KISLQH----NFFRCD-

--CKTK--------------------------------------------W---M--KQW

LL-----REEAH-------------VDNI--------ENILC----YSD----HV---QG

KAISR-----------------LPDEEFVCLVGENDKSGN--------------------

------PVESPAFKITAYTLGGLLLVFLVAFAVG---YKFRG-EVKVFMY----------

-----THFN----WHPFDRINDSDPNK-----------TYDAFISFSG---I--DYEW-I

SNTL--CARLE------NHDP----PY----KLCLH-HRDFLVGAP-IQQNIFD-GIERS

KRMIMILSKN---FVKSEWC-LLEFRAAHQ--KVLEDRI---NY----LIIILFDDVD-M

AEVD-DEIKL--YMRTNTYLSV------------KNKWFWEKLFYALP------------

-----------------------------------QNSNRETKANDCHRGAHVNSTADED

NDLSNRNGYKRNDNVAEQGRTEQLGLVWNKTKIIDNLYSWTRKLRSSDNRSPVK------

------------------------------------------------------

>Sako_TLR_7

------------------------------------------------------------

---------------------MITSTKATTHKMNISLLK---------------------

---------SVLIIMMADIT----------------------------------------

------------------------------------IQLECPRGCTCDVNESNIYQFD-N

I-LCGWGSFSARGDTTSI-AATTKLSMHCDSKPLINAVQFTLHHLQNKSVLTEVEFT---

------------------FCPVPILTNRSIPFL-P----NLRILNITNSDL---------

------NSITDNALQN----LPSLSVINL---TNNKLSV-------------IPDTI---

-PGHSHNTTVASKIQELYLDG--------------------------NSLQ---NIC---

--------NQT---FKE-FTNLKV---------------------LTMAMTGQHETLPEG

------------------------------------------------------LFMNLH

H--------------LKTLDLSRNTI------PH--I--SPTLFDNTTDLEVLLLYSN--

--------------------------------------ALQVISTGTFNNLRKLRILDLS

LNIIHTLS-VDVFNGMESLQHLNLRYN-----------------------------MFLD

LPSNLFHGLHNLQHI----DLSEIEFSWS-RVVLPDDLFIDIPMLKTLDISKANI-T--S

LRNMTFAGSV----ALEE---------INFSTN-HIK-----------------------

-------------------------------------------------LIP----KDAV

LFSHAINLRTVDFSYNEIS----SI-------------ESHAF-----QDSQLRSI----

----------DLSHN---------------------------------------------

--NITI-----IQKHTFY--KL---S------QLNTLDLSH--NY---IYFIHAVG----

----LFQLPN-----------------------------LKELR---------------L

NNNLLTNFQF------------------------------------FLYARRGALVQPLP

ALPDFLHFDDP-------MLVYMANNP------------FNCDCQMFAKLFNTSG-HARY

--------------EHILWPGTDQLINQRFMPDPSSLVCYRPMRLRGRSIMSLKKNEYWC

ETTSFCPDNCTCYMVPVE------------------------------------------

-------NSLIVNCSYRGFTEIPDNLWFGI-SY----LLLEGN----------YITRIT-

-----------------------NNKLNH-------------------------------

------------------------------------------------------------

------------------------------------------------------------

--------------------------FTRL---WELYLN---NNAIQ-------------

----II------------------DDKA----FHNLTSLIT-LDLSY----NLIT---KI

T---NEFTTM-SNLENLYLNFN--NIDYISTNSFVSG----G-RLKELMLDNN-MLESID

V-DMFN-N-----T------------------------NSLT-MLTLHN----NLYQCD-

--CQTP--------------------------------------------WVD-F--KHW

LQ----QPSINA-------------IVAF-------QYNITC---STNN----TL---ED

VPILQ-----------------ANE-N-DLNCTTSTDSQV--------------------

-----INVIQNRIIGAVLGLAVIAMIIGVTV------FKFRN-IIRVIIY----------

-----NRTG--WRLHHNKDYED-AK-------------IYDAFLSFSS---E--DLPW-V

KNSL--LKKLE------NHVP----PY----KICIH-HRDFIIGEC-IATNILD-AVEKS

RRIIVILSNN---FLRSEWC-AYEFNQAHL--QVVRDKS---CR----LIVILMEKIP-Q

QDID-KEIKM--YLKTNTYLEM------------NDPMFWEKLYYVMP------------

---------------------DV-------------------------------H-GRKD

ENADEEQLVENI------------------------------------------------

------------------------------------------------------

>Ptfl_TLR_29

------------------------------------------------------------

---------------------------MNARVSRIFSLL---------------------

---------LIIAAGICTQGQL--------------------------------------

------------------------------------NIPIIPGCIFDEDLETSEARCW--

---SAALSNLDNTTKIPPTASLDFFCDSSLAGDPLTFILGRMQDTELLQRLAIQNCPIN-

-----------------------FLDASDFVQL-P----GLQQLNLTYCGV---------

------NEISANAFSA----MPNLRSVNL---GGNSFSS-------------LPSAL--T

CRDSNHSCGIPSRLEELNLQS--------------------------NDFR---------

--------KLSSNAFSE-LPELKK---------------------LNLARNR-LQSLRGD

------------------------------------------------------IFGSLQ

H--------------LQELDLRLNQLATLPAE----LFHNQSKLEFLYLGSNDIQALD--

--------------------------------------------QDVLTNLASLKVLDLS

FNDFTTLP-VGTFEGLRNLQRLNLMNN--------------------------DFKNSLP

VDLLQGLVSLEAINL----SDIEFEQSGF---VLPYGLFRNLPSLRSVLLRRANVT---S

IRQLSFTGCT--IFST-----------VDLSYNHIQD-----------------------

------------------------------------------------------IPSDFL

MTIDAPTLEVLNLAHNNIS-----------------DLQIRAF----QNSDKLREI----

----------SLGHN---------------------------------------------

--HLKT-----ITRYAFY--EL---S------ALQILDLSH--NQ---IQSIDFTG----

----LYQLPS-----------------------------LVELR---------------L

NDNRL-TNFQIF----------------------------------MYARAG-ALRVPEE

LRPDFIDFDKP-MSVYYEGNPLTCDCH------------MFETLYNKTGHSRYEHYLWPG

--------------AQILM-NQRFM----------------------------PDPSALV

CSNPPNVRGEQIQTLDRN--------------------------------LFWCEMPEFC

PQNCTCSAIAVQNRLIANCSNRGLTEIPADLWWGTHFLLLHNN----------NITRIS-

-----------------------QNQLKN-------------------------------

------------------------------------------------------------

------------------------------------------------------------

--------------------------LTYM---LELHMD---NNSIS-------------

----DI------------------EDGA----FDDLISALI-VNLGS----NKLQ---SL

S--APDFRGL-SSVETLNLDHN--NISRIDVNS-FQN----LTRLKSLTLDNN-SLRSLK

P-GVFNGT------------------------------KSLE-YLRLDS----NPFVCD-

--CQL---------------------------------------------WWLDF--KEW

LL----KPNVNR-------------LLDG-------RYNINC---TTHR----ET---NS

SEIWS----------------LLLLTEDDIMCNDTGDDYT--------------------

-----GLQLTDSNSKIIASVLGLAVVILASLALL---FKCRG-VLQVVIY----------

-----NRTG--WRLFKAEDFDESK--------------MYDAFLSFSS---E--DLPW-V

KNTL--LKNLE------NHDP----PF----VVCIH-HRDFLVGAC-IAENITE-AIEKS

RRTILVLSNN---FLESEWC-AYEFKQAHH--QVLIDKS---SR----LIVLLMEDVD-T

GKLD---QDLKMYLKTNTYLER------------DDPLFWQKLYSAMP------------

------------------------------------------------------DGSDVK

RQTKPKVEKGYPGPDNELLLIEEI------------------------------------

------------------------------------------------------

>Ptfl_TLR_30

------------------------------------------------------------

----------------------------MGALKFTVILV---------------------

---------WLVQSLSIVEIQALQACPTG-------------------------------

------------------------------------CTCEFSELTTLLYNSVF-CTVA-N

M-MSSTLPITLPPTLILN-IHY------GSGFSTIEPLNITEWIHHGADLIKLDVYG---

------------------FPLYRITEM-DFPYL-P----NLLVLNITYCGT---------

------REVSPNVFQR----FPKLKVLGM---SGNSLSK-------------VPSAL-TC

IGQPQCDDHLPKQLERLDLRA--------------------------NSIR---RLQ---

--------DSA---FTH-LVNLTS---------------------LNLGLNGLITLQEG-

------------------------------------------------------IFDPLQ

N--------------LQYLSLYNNAI-GDM--PL--T--IFQHLTNLEALDLGSNSLK--

-----------------------------------------AIDSRMFRNLINLKVLDIS

LNLLSTLP-VDIFTGLVNLQAVSLHYN-----------------------------NMKT

LPRDLFQDLPVVRYI----DLSESDQEWS-RSNLPADLIRNIPSLKTLNLKKTGI-E--S

LANLTFEGCT----SLSK---------LDLSLN-FIG-----------------------

-------------------------------------------------EIP----KGFL

NQVNAPSVEYLYMQSSNIS----HL-------------AKYAF----KDSSRLREI----

----------DLSHN---------------------------------------------

--NLKT-----LPKYAFY--RL---S------ALKILDLSH--NN---IFHIEDES----

----LYRLQD-----------------------------LQELK---------------L

NDNFL-TNLN------------------------------------LFPYIQAALTIK--

---TFLNMEAN---------PLSCDCD------------MFTVLYKYNQTGYPRK-QDLD

--------------HLIFHPALNSL-------------------------------QCHS

PENVQGRSISSLEFYDFW-----------------------------CELPVLCPKHCTC

RRQAFDTSVFLTNCSFQGFLDFPNKVWQYT-TI----LMLQGN----------NITHIS-

-----------------------ENQFEN-------------------------------

------------------------------------------------------------

------------------------------------------------------------

--------------------------MTDL---QELNLA---MNSIT-------------

----WI----EEGA------------------LRYLSKLTY-FDLRS----NKLQ---TV

S--GSEFSFM-PNLQYLNLNFN--NISHIHMNS-FKS--L-R-NLEYLLLDHN-RLEDID

V-GMFE-M-----T------------------------TKLK-ELSVDH----NPYVCT-

--CQ-LW-------------------------------------------W---VNLQEF

IL------SHEN-------------VIPN-------KYDINC---TTPY----KINSTNK

WPMLQ-----------------LKE-A-NLDC------ND----------------TDPN

QGGTYHIKLTDMESRIIAGVLGLTAVVMVAMATI---FKFRN-NIRVVVY----------

-----SITG----WRILEKTEV-FE-----EN---K--VYDAFLSFSS---E--DLDW-V

KNTL--LKNLE------EHDP----PY----KVCIH-HRDFIVGAC-IAESIVE-AIEQS

SRTILVLTNN---FLESEWC-AYEFKQAHH--QVLLDKS---SR----LIVLIMEDID-Q

DKLD-RELKT--YLQTNTYLEK------------EDPLFWEKLYYAMP------------

------------------------------------------------------HVKHIR

KLNRPKRQHWRRGGYDNELVEIHGE-----------------------------------

------------------------------------------------------

>Stpu_TLR_55

------------------------------------------------------------

------------------------------------------------------------

---------MVSSSSSLTDG----------------------------------------

----------------------------ACP------------LCSCSDYGKS-IDC---

---------------------------------FTEQLNTSY------------------

----------------------------PFADIDI-------------------------

--------------------APEVTSMVITGGDTDEDRP---------------------

------------------------------------------------------------

------------------------------------------------------------

------------------------------------------------------------

------------------------------------------------------------

------------------------------------------------------------

------------------------------------------------------------

------------------------------------------------------------

---------------------------LELRDS-VLG-----------------------

------------------------------------------------------------

-----------------------------------------------RSFPNLRSL----

----------HFSTC---------------------------------------------

--SIGI-----IEDDAFE--QM---P------YLEELIFYK--NG---------------

---------------------------------------IQNIP----------------

------------------------------------------------------------

---RALKLLAN-----------------------------------------------SL

--------------NKLEITSQGKL-----------------------------------

--------------IDAF------------------------------------------

---------------------SGLNEFNKL-TI----LDISSN----------SICYLE-

-----------------------RRSFMG-------------------------------

------------------------------------------------------------

------------------------------------------------------------

--------------------------LSKL---ETLSLW---NNNIE-------------

----NI----DYGV------------------FIPIVNLKT-LDLSH----NKLR---FI

P---SAIKSL-IHLQELDSSYN--NISDVHNFE-FIT--YMP-NLTTLKLHGN-VISIID

C-VSVD-V-----L---------------------LNSSSLV-DIRLCN----NPYRCNK

TLCGFMY-------------------------------------------F---Y--TTH

LS----FSPFDA------LDPFR--VLPI-------KCTYYC---GSPF----GY---FG

NTLGN-----------------VYG-E-LCTIGLPIPTSV--------------------

---PEVVQKNRKSVRVIATVLGVLIAVGVAF--G---LLVYF-ALRRLGH---------L

RRG--FIFA----GQGFVKFNN-NR-----RN---ADIVFDALVYNHV---Q--ETHF-I

DDRL--RPRLE------DP-P---NDF----RLCLPLTRDFRLGGK-KLNNLRE-SMISS

RCAMFVISEA---FIQDARC-KQALEVACE--FLHRDDLGPAHLKQTGLILIVLDPVL-L

DQLP-ETLRV--LVDRLVTLEWD-----NL----NEERCWKDLNRPLE------------

----------------------Q-------------------------------F--REQ

NEI---------------------------------------------------------

------------------------------------------------------

>Stpu_TLR_52

------------------------------------------------------------

------------------------------------------------------------

------------------------------------------------------------

-----------------------------------MNNSVQVRVWVWVCSLLFVGRCT--

------------------------------------------------------------

---------------------------------------SLTFCDYCYCDT---------

------PDIWCSGVNF----HEALNHT-------------------------IPSRV---

----------ASNTTFLNVNI---------------------------------------

--------------FDG-------------------------------------------

------------------------------------------------------------

-----------------EMILANDTL----------------------------------

------------------------------------------------------------

------------------------------------------------------------

------------------------------------------------------------

------------------------------------------------------------

------------------------------------------------------------

-----------------------------------------------SVFPALQRL----

----------SISTY---------------------------------------------

--QVSF-----VDQFVFE--GL---Y------ELSYIYLER--NG---LHEIPSEV----

----LFHLRTS----------------------------LQELH---------------L

VNQRL-RNIPSN----------------------------------AFAELV-NL-----

---QELTITYN-------------------------------------------------

------------------------------------------------------------

-----------LNYLDIS------------------------------------------

--------------------PDAFNGLAKL------------------------------

------------------------------------------------------------

------------------------------------------------------------

------------------------------------------------------------

---------------------------------KGLCLS---SCNIR-------------

----HF------------------PNTT----FSGLHTLHE-LDLSY----NRVV---EV

P---TAIQTL-KSLMKLDLSHNKLLVNPMDLMF-LRS----TTKLHTLQMRYC-AISKIL

P-DAVD-N-----L---------------------KASRDLT-VANFDG----NPFNCTE

DLCSFAS-------------------------------------------W---Y--VSL

PGLSTSTRSPDF-------------IPYITLSPPPGKGPYQC-----------DI---YG

MPLKA-----------------FFS-E-SCLPRPDPSPSI--------------------

-----FPPGETPWHVVIIAVTVILVAFVVTILSF---VVWKFRLINYYHH--------HI

GVSFQRHVN----YGAVGDNNH------------EY--VFDAYVSHHD---D--DKSF-V

EDEM--LPRLE------DEH-----GF----DMCVS-FRNFRLGSN-LLENVSS-AQDVS

RAIIFIINER---FMQNGQC-KLELEMAST--RMLEDET---GHEGQRLIIILMEVLA-P

ELVN---STLRVLLNHVAYLEWD-------PA--AEERCWGQLIATLD------------

---------------------------------------------------------TLM

PERNA-------------------------------------------------------

------------------------------------------------------

>Stpu_TLR_64

------------------------------------------------------------

------------------------------------------------------------

------------------------------------------------------------

------------------------------------------------------------

------------------------------------------------------------

------------------D---------TFQSF-N----ALQRLVIDHCPV---------

------TFVSEFVFEG----LNELRYISL---TLNSLTN-------------VPIKA---

------------------------------------------------------------

------------------LSRLKT------------------------------------

---------------------------------------------------------PLE

E-----------------------------------------------------------

------------------------------------------------------------

------------------------------------------------------------

------------------------------------------------------------

---------------------------LHLVHQQITN-----------------------

------------------------------------------------------------

------------------------------------------------------------

------------------------------------------------------------

-----------IPANSFA--QF---G------SLRKLTIKY--NF---------------

------------------------------------------------------------

------------------------------------------------------------

---HDLDISAN-------------------------------------------------

------------------------------------------------------------

------------------------------------------------------------

----------------------AFASLANL------------------------------

------------------------------------------------------------

------------------------------------------------------------

------------------------------------------------------------

---------------------------------THLYLS---NNGIR-------------

----LL------------------HDTT----FMGLHNLQE-SDLSY----NLLV---AV

P---TAIHAL-KSLKKLDLSHN--QLLDNPRNLLFLV---GMPKLQTLEMGSC-AISEIS

S-DAVD-N-----L---------------------MTSKDLA-VANFDG----NPFNCTK

DLCSFAS-------------------------------------------W---Y--LSL

PEPPSTTRSPYY-------------IPFITVSPPPGKGPYRC-----------KS---SG

QPLLE-----------------FYS---NSYCLPAPDLTP--------------------

SNVPDPADDSTPWYAIVVPVTLIPVAFVLVIVSF---VIWKF-RLINQYH----------

-----HHFG--IHFQRRANNGA--------VQRNNFEYIFDAYVSHHE---D--DKPF-V

QDEM--LPRLE------DEN-----GF----DLCLS-FRNFRLGSN-LLENVSS-AQDVS

RAVIFIINER---FMQNGQC-KLELEMAST--RMLEDEM---DHGVQRLILILMEVLE-P

DLMN-NTLRM--LLNHVAYLEWD-------PT--VEDRCWGQLIATLR------------

----------------------T---------------------MV--------P--EGN

SDDERGANDERNAGDEPLCNQYEQRV----------------------------------

------------------------------------------------------

>Pate_TLR_4

------------------------------------------------------------

------------------------------MELMKMKIL---------------------

---------LSLLLTVQLIIGFETLSVETTL-----------------------------

------------------------------------FQNGTAVLFLEQNGTTSPSSLQ-N

GTEILFLEQNGTRLLIPEQNNT------ATLPSGKNYISLMPIE----------------

------------------QNTSLLSSDRKFEMLSDKNTILRNFLQTGDSNLNHATRQNTP

ILDLNGTFLHITILNR--HNFTTLDEVSIMTANSSRLFDEILKDIRKVLKNNDSNDDPFD

SNFASGKFSVLMNISHDTVTH--------------------------NEL----NID---

--------FSS---FYI-LTHHFK---------------------CNLDKNDELTISCKN

VS-------------------IY-------------------------------SLTQYL

P--------------NNTVRLEITDSPTVTLTKP--LFSYSTKLTYLRLSNNQHRFIT--

---------------------------------------------RAFTGLQSLQYLDLS

DNNIMAV--KYAFSYLSNLKYLNLSKN-------------------------SFITIEVV

ANALEGLKHLEILVL----DHNWRIFRIR----KNELKPLSNSNITKLHLFNTTMS---I

AEKGAFEHLK--KLEV-----------LDLSVNYLNE-----------------------

---------------------------------------------EALTNVTSSLKNAPI

RYLFLQEWVHLNVFPSESL----KFLENTSIQHIYLSRNYFIKIHKLPYIPSLQSL----

----------WIDQC---------------------------------------------

--SVRI-----TDNDLIS--QL---P------NLTVLVLSG--NN---FLRFNVPS----

----SRSLKI-----------------------LQLSRQLTRYE---------------M

KEFEISGVF-------------------------------------AFKNTP-EL-----

---EFIDFTSN---------LFPETLS------------RY----------DFFG-LHKL

--------------KRLIL-----------------------------------------

---------SDVDLVAIE------------------------------------------

--------------------DYAFETLGSL-EI----LDLSHN----------RLRYLS-

-----------------------NATLFG-------------------------------

------------------------------------------------------------

------------------------------------------------------------

--------------------------LHKL---TDLYLS---NNHLI-------------

----LP-----------------DELNP----FQQTPLLTI-ILLNN----NRIE---KL

P--KGIFNFT-DHLELLILSQN--KIHPWDEPI-FGE----NLTVNTLGLSQN-YIDYVT

P-TMLS-E-----F------------------------QKNTSYLDISR----NPFNCSN

--CGLRQ-----------------------------------------------F--QAF

LK------STPI-------------QMGLPYSINKGIDLVKC---SLPN----KL---AD

QSLVV-----------------VSF-A-DCSE------FE--------------------

-------KLLSLSFIVLISVGSIFFIMVACFC-----YSFRW-YIRYWVF---------H

VQS--KFKE--RRTSNRKSERV-------------Y--KYDAFISYNS---N--DTSW-I

ASFL--IPALE------RQDP----KL----KLCIH-DRDFEVGRF-ITENILE-AIENS

RKVILILTEE---FVKSEWC-MFELHMAQH--RLFDETR---DC----LILIKLKKVD-K

KFYT---KHLKYLEKTRTCILWP-----DTLT--DQHVFWEKIRKLLG------------

--------------------------------------------------------NTQP

QNEYELAGFEI-------------------------------------------------

------------------------------------------------------

>Pate_TLR_7

------------------------------------------------------------

------------------------------------------------------------

---------MRMFSTTLILL----------------------------------------

------------------------------------LGFLELVTGFHTHISSSSLSAQ-N

KTLSLSSEQNRTLSMPSEGKEAPSLYKKSEAPLHFLPTNISHDS----------------

------------------NHALQRNAR-SFSLNRT----NLNNVPLNSHNI--------T

TLHDVPIRITDSQLLV--IRALDFDLFDIRLFDSNDLLF-------------QVLNT---

-------SEGNESFEIPIVTI--------------------------NNTSEYNETG---

--------GALESSYYL-IRYFKC----------------------NMDQNDDVSISCKN

VSIY--------------------------------------------------DVAKYL

P--------------NDTASLEITNSPTMKLLKP--LSRSLKKLKYLRLSNNEHRYIT--

---------------------------------------------RAFVGLENLEHLDLS

DNNIMAV--KNAFSYLSNLKYLNLSKN-------------------------SFITIEVV

ANALEGLKHLEILVLDHNWRIFRIRKNDL--------KPLSNSNITKLHLFNTTMS---I

AEKGAFEHLT--KLEV-----------LDLSVNYLNE-----------------------

---------------------------------------------EALTNVTSSLKNAPI

RYLFLQEWVHLNVFPSESL----KFLENTPINRIDLSHNYFIEVHKVPYIPSLQSL----

----------WLTHC---------------------------------------------

--AVKF-----IDHDVIS--QL---P------NLTELVLSN--ND---LVHYDISS----

----SKSLKT-----------------------------LILSD-------------QAS

RFKTVELTLSDF----------------------------------GFKNTS-NL-----

---EIIDLSSN---------LLPRTLH------------RF----------VLFG-LKNL

--------------RALSL-----------------------------------------

---------KSAGLINIE------------------------------------------

--------------------DYAFETLSSL-NT----LNLQIN----------NIKYLS-

-----------------------NATLFG-------------------------------

------------------------------------------------------------

------------------------------------------------------------

--------------------------LHKL---TNLYLS---YNLLT-------------

----LM-----------------DWGNP----FQSTPSLVN-LLLDN----NRIE---NL

P--KDMFAFT-DHLELLILLRN--KISPWDEPI-LGE----NSTVSTFALSQN-NIDYAT

P-TMLS-E-----F------------------------RKITGFLDIGR----NPFNCSK

--CGMKN-----------------------------------------------F--QNF

LN------STHI-------------QMGLPLRINQEKISVKC---MLPN----KL---QG

QPLVE-----------------VDLPFTDCTE------QE--------------------

----------ELVNVFTIVLICLLSLFVAVMAYTC--YTFRW-YIRYWVF---------H

IKS--EVKE--RRSSNSKHERT-------------Y--KYDAFVSYNS---S--DTPW-I

VSFL--IPALE------NQDP----KL----KLCIH-DRDFKVGWL-ITDNILD-AIENS

RKVILILTEE---FVKSEWC-MFELHMAQH--RLFDETR---DS----LILIKLKQVD-K

KFYT---KNLKYLEKTRTCLLWP-----DNLP--DQKLFWLKVRKLLG------------

--------------------------------------------------------HPHL

QSESEVMGLEQYRSINKI------------------------------------------

------------------------------------------------------

>Crgi_TLR_6

------------------------------------------------------------

---------MNKHHAPQDIIALQSPISYSYNIPFFSVKE---------------------

---------HLLLLGIAFIL----------------------------------------

--------------------VGLARGENVLTSYP--CPRQCNCYQGSVDEEVV-SIVC-Q

V-DYI-------HPDDDF-YVI------RTQITSILYIICTEKD----------TLS---

------------------H----VKDG-MFNNL-E----SFMGLSVENCRV---------

------SYMPGNFLSG----LQSLEQIEI---KSAGTLE-------------MEDSV---

-------FHQVPKLTHVTIAS--------------------------SHVV---KMP---

--------D-----LCK-LSNLKF---------------------LNVSDND-FQSMEST

GV-------------------------------------------------LCKNDTVLP

H--------------LTTLILDKNSI---FNISS--G--SLKSLPNLNDFRIADGNLVNI

EEDALSDIQKITFLDL-------------------TNNAISNVSVSQFSWNRELEVLGLG

RNPLKQIH-PKTFSSVKNLIVLTLDYSGLDNAVWESLYPFRQLKDLQLQGNSVTMLNRTV

LRSLSSLRNLDLGDN----RISDLSTEMF----------QAMAELQFLHVNQNNL-T--E

IRNNTFLGLQ----KLVA---------LDLRGN-RIK-----------------------

--TIEKGAFSHFVSLAELDLSNNCLSEIPSFNNATTLQSLDLSSN---YIQL----LSSN

ALKGLQNLQNLVLFNNSIK----QI-------------ETGVF----RFVPALRTA----

----------DFSFN---------------------------------------------

--EIHR-----ILKETFE--GL---Q------QLSTLFLQN--NL------IENIS----

----IEGH------------------------DIPG---LRTLN---------------L

SSNLLSSKIASG----------------------------------MFPDNV--------

---ENLDLSYN---------NISDITE------------Y-----------AFYS-YEHL

--------------RRLSL-KGNKL----TTLAMND--------------------LAVP

IDQSR----RTIVYISDN------------------------------------------

---PFDCDCKLIWLRKKVNEISMNSGFPMV-GD----IYSVQCEKGYRIQEPVPFYSIP-

----------------------TRDMLCE-------------------------------

----------------------------YTTECTKNCFCCEFEPCDCKFVCPYGCSCYSS

ADFMTTHYVQCTKRGLTKIPAG--------------------------------------

-------------------------FPASS---TEVSLD---RNNIS-------------

----EI----YSSS------------------FVGLIYLRV-IHLDH----SGIT---TL

A--NNSFIGL-LQLKTLYLNNN--ELQEINRGV-FNK--L-W-NLTELHLEYN-NIAYIE

E-GAFSAL-----TSLSTLFLDHNLLISLPQSATNHFFWFLS-NIRLGE----NPWSCS-

--CDVMA-------------------------------------------E---F--IPM

VM--------NR--------SMV--ISDY--------SDMFC---KETG----EN---AN

FSMKDVLVKRC-----------TNITS-EQVFEMKQFDNW--------------------

---------PNILKILVITVAAIILTTFFII--I---VICLW-RPIVLFT----------

-----HRKC----KCCVRKRYP-ED-----GD---K--SFDAFLAYSH---K--DDDY-V

TREF--IPRLE------NE-L----KY----RLCVY-YRDFPIGGT-IADTVAS-SINRS

KRTILLVSKH---FNDHEWR-NTAFQHSFG--GLFKQKD---NH----LIIVLLDDAK-G

MKLD-RQLKV--LVKSHHVISY------------RDMCFWEQLQYKMG------------

----------------------S---------------------SK--------R--SIV

RNNTPDLILNHSYTQQQDADGYETPVSSSASNTETDKCRRSLDSINNIYEEIRSSKLSDI

SLV---------------------------------------------------

>TLR11_Mumu

------------------------------------------------------------

-------------------MLKESMPRMERHQFCSVLLI---------------------

---------LILLTLVSLTLTGWAWTI---------------------------------

------------------------------------PDCIIADSLLFPNLSYYIPFCT-S

A-PGLHLLASCSNVKNLN-QTL------KRVPRNTEVLCLQG------------MVP---

------------------T----LPAK-AFIRF-H----SLQLLRLQLRTT---------

-------SVTSRTFQG----LDQLQYLFF---DHHAPCC---------LSLFLSPNC---

-------FESLRSLSSLSFQG--------------------------YCLTYSQSIYLPT

SLRHLTLRNSCLTKFQD-LQRLFP--------------------DLLLSTSSTPNIKPGA

PF----------------------------------------------------------

---------------LETLDLSYNLQ-L-----------KQAGVRDLYGLTLHSLILD--

------------------------------------------------------------

--GTPLKALDLTDSGLLHLHFLSLV---------------------------------GT

GIEKVPASLTGYSEL----RALDLGKNQI-QNILENGEIPGYKALEFLSLHDNHL-Q--T

LPTRFLHTLP----QLQK---------LNLSMNKLGP-----------------------

---------------------------------------------------I----LELP

EGLFSTNLKVLDLSYNQLC----DV-------------PHGAL----SLLSQLQEL----

----------WLSGN---------------------------------------------

--NISS-----LSNESLQ--GL---R------QLRTLDLSW--NQ---IKVLKPGW----

----LSHLPA-----------------------------LTTLN---------------L

LGTYLEYILG------------------------------------IQLQGPKML-----

---RHLQLGSY---------PILDIYP------------PW---------------PPTL

--------------LSLEIQAESCI-------------------------------QFMI

HSGQPFLFLENLTLETSI------------------------------------------

---------------LLLKPDNITIHFPSL-RR----LTLRGY----------SFIFST-

-----------------------SQLQRF-------------------------------

------------------------------------------------------------

------------------------------------------------------------

-------------------------FPQQLPLLEHFFIW---CENSY-------------

----AV-------------------DLY----LFGMPRLRV-LELGY----LNFFYESST

MKLEMLLKEV-PQLQVLALSHL--NLRNLSVSS-FKS----LQDLKLLLFNSE-RALEMN

S-NLQE-F-----I------------------------PQMPQYVYFSD----VTFTCQ-

--CEAS--------------------------------------------W---L--ESW

AT----RAPNTF-------------VYGL--------EKSIC--IANAS----DY---SK

TLLFS-----------------FLA-T-NCPH------GT--------------------

----------EFWGFLTSFILLLLLIILPLI------SCPKWSWLHHLWT----------

-----LFHT--CWWKLCGHRLR-GQ----------F--NYDVFISYCE---E--DQAW-V

LEEL--VPVLE-------KAPPEGEGL----RLCLP-ARDFGIGND-RMESMIA-SMGKS

RATLCVLTGQ---ALASPWC-NLELRLATY--HLVARPG---TT---HLLLLFLEPLD-R

QRLH-SYHRLSRWLQKEDYFDLS-----------QGKVEWNSFCEQLK------------

---------------------------------------------------------RRL

SKAGQERD----------------------------------------------------

------------------------------------------------------

>TLR12_Mumu

------------------------------------------------------------

--------------------------MGRYWLLPGLLLS---------------------

---------LPLVTGWSTSN----------------------------------------

------------------------------------CLVTEGSRLPLVSRYFTFCRHS-K

L-SFLAACLSVSNLTQTL-EVV------PR---TVEGLCLGG------------TVS---

------------------T----LLPD-AFSAF-P----GLKVLALSLHLT---------

-------QLLPGALRG----LGQLQSLSF---FDSPLRR----------SLFLPPDA---

-------FSDLISLQRLHISG---------------------------------PCL---

--------DKK-AGIRL-PPGLQW---------------------LGVTLSC-IQDVGEL

AG----------------------------------------------------MFPDLV

QGSSSRVSWT-----LQKLDLSSN--------WK--L--KMASPGSLQGLQVEILDLT--

------------------------------------------------------------

----RTPL-DAVWLKGLGLQKLDVLYA-----------------------QTATAELAAE

AVAHFELQGLIVKES----KIGSISQEAL----------ASCHSLKTLGLSSTGL-T--K

LPPGFLTAMP----RLQR---------LELSGN-QLQ-----------------------

-------------------------------------------------SAV----LCMN

ETGDVSGLTTLDLSGNRLR----IL-------------PPAAF----SCLPHLREL----

----------LLRYN---------------------------------------------

--QLLS-----LEGYLFQ--EL---Q------QLETLKLDG--NP---LLHLGKNW----

----LAALPA-----------------------------LTTLS---------------L

LDTQI-RMSPEP----------------------------------GFWGAK-NL-----

---HTLSLKLP---------ALPAPAV------------LF----------LPMY-LTSL

--------------ELHIA-SGTTE-------------------------------HWTL

SPAIF----PSLETLTIS------------------------------------------

----------GGGLKLKLGSQNASGVFPAL-QK----LSLLKN----------SLDAFC-

-------------------SQGTSNLFLW-------------------------------

------------------------------------------------------------

------------------------------------------------------------

-------------------------QLPKLQSLRVWGAG---NSSRP-------------

----CL--------------------------ITGLPSLRE-LKLASLQSITQPR---SV

Q-LEELVGDL-PQLQALVLSST--GLKSLSAAA-FQR--L-H-SLQVLVLEYE-KDLMLQ

D-SLRE-Y-----S------------------------PQMPHYIYILE----SNLACH-

--CANA--------------------------------------------W---M--EPW

VK-----RSTKT-------------YIYI-------RDNRLC---PGQD----RL---SA

RGSLP-----------------SFLWD-HCPQ------TL--------------------

----------ELKLFLASSALVFMLIALPLL--Q---EARNS-WIPYLQA---------L

FRV--WLQG-------LRGKGD-KG-----KR---F--LFDVFVSHCR---Q--DQGW-V

IEEL--LPALE------GFLPAGL-GL----RLCLP-ERDFEPGKD-VVDNVVD-SMLSS

RTTLCVLSGQ---ALCNPRC-RLELRLATS--LLLAAPS--PPV----LLLVFLEPIS-R

HQLP-GYHRLARLLRRGDYCLWP-----EEEE--RKSGFWTWLRSRLG------------

------------------------------------------------------------

------------------------------------------------------------

------------------------------------------------------

>TLR9_Xetr

------------------------------------------------------------

----------------------IVSLHAQQKRKVFLPLS---------------------

---------LGLILGVCLAF----------------------------------------

------------------------------------CKIPHFLPCDDFNNAST-VICR-E

R----------QLIHVPHIVSQ-----------SVKVFDLAI------------------

------------------NEIRLLANS-TFSGV-P----NLVILNLSDNCQ---PSNLRP

YKEICRLIIEPHALVS----LKSLTNLDL---SGNSLTS-------------IPPLP---

-----------ENIKFLNLNL--------------------------NQIH---MIS---

--------GWE---FSR-LNNVKM---------------------IRLGYNCFYSKQCEP

FH------------------------------------------------LSDSAFINNE

F--------------LEELALSFNNI-----TSF--PQNLPSSIRILDLSENKISKID--

-REDLCNLNNLHSLDLQWNCQRCDHALQACYPC--KNNSALLLVPGVFDCLHKLSYLNLR

GNSLHTLH-SSLFSSLTNLSHLVLSDN---------------------------FLNLET

ETFFSALTNVKELNL----DFNFKPYSMY-ERLVLNSNVARMKSLERITIVGYFFNV--L

DEEGIMPLLS--LPNLRE---------ISLRTNFILK-----------------------

---VNLSMLFTHKPLRFISLSENL--------------------------------ISFE

EHKHGQKLAMHPPLFDRGN----QVDGGWMDSHCGPKQVEAGEVEYPGCWQYNHSV----

----------DLSFN---------------------------------------------

--NIGS-----IYPDEFL--GM---D------EIECLNMSY--NY--INQRLNGTQ----

----FGHLKS-----------------------------LRHLD---------------L

SHNRF-DMYYYK----------------------------------ALSELP-RL-----

---KILNLAHN---------DYQFMMK------------GV------NHRLDFLENLTSL

--------------VELNL-NNNFI----------------------------------G

LRITRELKSHSLETLRFR------------------------------------------

-----NNELGSSWQYGKDTYLNMFTNLRSL-KI----LDISYN----------QLPVIP-

-----------------------NEVLEK-------------------------------

------------------------------------------------------------

------------------------------------------------------------

-------------------------LPESL---QKLNLS---HNKLY-------------

----TF------------------NWAK----TAHLGNLAI-LDLGF----NALT---KL

R--ANLTE---SNIAFLNLTYN--KINSLDKDF-FDS----FSELKQLILSNN-LIKTIH

I-TSFPIN-----F-----------------------LQNLD-SLDVSG----NPFQCT-

--CKAY--------------------------------------------W---F--ITF

LM-----ETEVT-------------VDHL-------STGMKC---DSPD----SL---RG

RSLLS-----------------MDP-Q-SCQE------LY--------------------

----------GHVCFIWSSILVIFLMVIPTI--W---NLFFW-NLWYAGH---------L

IVA--TLRS----YTKLHDKST-------------E--HFDAFIAFNTKNSS--VRDW-V

YNEL--LVQLE------SPERG---GF----TLCLE-ERDWIAGRS-SIENLYD-SIYRS

KKTIFIITRE---WFNCGLL-RHAFFMSNQ--RLLDEKK---DV----VALVVLDH---K

MKMS-QYFLTRKRLCPKSFLNWP-----CNPK--AHSHFWHMLRIYIR------------

---------------------------------------------------------QDS

RRCCGSQLKKYVDK----------------------------------------------

------------------------------------------------------

>TLR8b_Dare

------------------------------------------------------------

------------------------------------------------------------

--------MIVAFFLLCTVHSSEGYN----------------------------------

------------------------------------WAWRKLPCDVNLSNTSVTLDCS-E

R----------YLKKIP-----------KNLIWNTTNLNLAN------------------

------------------NKIHNISKD-AFWNL-N----NVTWIDLRRNQI---------

---EKCHEKDNGVFSR----LTNLKTLLL---DNNKISV-------------LPKNL---

----------PAGLQWLSLNS--------------------------NHIK---SIE---

--------QSD---FKG-MTKLTV---------------------LKLNKNC-YHNISAE

------------------------------------------------------LTIQNE

T--------------FQHLQLTELQLSKNGLHNV--PFALPRTLHNLSLLLNRIDHVH--

-ESDLNHLTRLKVLDLSGNCPICFTTPFPCTSCQ-TNNNALQIHPNAFSKLSQLQDLRLS

GNSLQSIN-SMWFQNLTNLKYLYLSFN-------------------------SLISEFES

GQFFSVLPQVEVVDI----SYNNPSERIY-PRLKLSEGFSRLESLQTLHLEGYIFH---K

LSEDDLRPLF--SLRNLSV--------LNLAVNFLQQ-----------------------

---VNLSVFRNFHNLSLISLIDNRLTFSSPIRRWEGQ-------------------SKSG

FKDDNQGDHREGPYIHTNE----EF-------------RHYPPFTKAECLATGPVL----

----------DLSRN---------------------------------------------

--NIYH-----VNPPLFT--GA---E------NITCLNLSS--NF--IVSYFNGTE----

----FAHFPK-----------------------------LKYLD---------------L

SHNRI-YMHSDS----------------------------------ALSELK-AL-----

---EVLDLSHN-----QHYFEVAGVRN------------CL----------TFLENLQFL

--------------KVLNL-SWNEI----------------------------------N

MLTNKTLQSDSLNELQFQ------------------------------------------

------GNRLDIMWKKQRGYQSLFKSLSNL-TY----LDISYN----------KLSEIP-

-----------------------DDIFDY-------------------------------

------------------------------------------------------------

------------------------------------------------------------

-------------------------FPKTL---RYISMS---RNTLT-------------

----DF------------------AWEQ----LQSLPQLET-LDLSK----NKLR---VV

P--RKLSKHT-RSLKVLDLSHN--QISKLRYSF-LEN----VKSLQILNFANN-KLKHLG

A-SSFTTG----------------------------SNHQLQ-ILDLQR----NPIHCT-

--CNLLD-----------------------------------------------F--ILW

LE-----KSDTI-------------LPRL-------ATDVLC---DLPE----SK---RG

HPMVS-----------------LDFKN-ACIN------NS--------------------

---------IAEILYILTSSVITLVMCTTIG--I---HVFYW-DISYAYN---------F

CMA--RFKS-----YYLKTNDC----------------IYDAFVMYDTKDPM--VAEW-V

LNHL--RLELE------DRGR----HVR---PLCLE-ERDWTPGIP-IMDNLNL-SVHRS

RKTIFVLTEG---FVHSGIF-KMAAFLAQQ--RLLEEGV---DV----MVLVLLEPVL-R

QS---RILNLRRCLCGHSVLEWP-----RNPA--AEGWFWQSLRNAVR------------

---------------------------------------------------------FES

QGVQSKMFKNYFNG----------------------------------------------

------------------------------------------------------

>TLR8a_Dare

------------------------------------------------------------

------------------------MVKNLDIGLILILKL---------------------

---------TLALSELNVRI----------------------------------------

------------------------------------LKTQPCDIHENITAETVVVNCR-G

RKLKVL----------------------PQFLANTTYIDLSE------------------

------------------NYIKNLTVQ-SFHGL-E----NLTLLNLNWLNQ---------

---NREVVIAKGVFSN----LTKLRVLDL---NGIKLKY-------------IPKDI---

----------PKNLEKLSLVE--------------------------NKIT---WIN---

--------LTT---FEH-VKNLSV---------------------LYLSNNCYYWNPCSR

RY-----------------------------------------------RIEKGSLSYLV

N--------------LKRLTLSFNNL-----TQV--PIGLPVSLERLELGSNSLTYIG--

-EHDFRGLFNLTVLKIQGNCPRCHTAPYPCIP---CKNTSIEIHPQAFSDLRNLHILHLA

GNSIKSIN-PAWFANLSNLQQLFLSFN----------------------LLFSAITDPAD

TVFLGNLPLLTKLDL----SYNFAFKTYP-LTVVLSPGFANLTSLRSLHLRGLVFR---K

IQKDTFKSLF--DLQHLNV--------LDVGVNFIVF-----------------------

------ATSYIFQHVRLLYLAENRLYPVTVNGE-----------------------LSKA

TFVGSNHRSIMPLMAEPLD----TF-------------DAPKNLVKSECYNAGRVL----

----------DLSRN---------------------------------------------

--NLFL-----ISPEQFD--TY---G------NISCLNLSR--NG--FSTAPNGSE----

----FTSLPN-----------------------------LKYLD---------------L

SFNKV-DLAYDN----------------------------------AFRELQ-SL-----

---EVLDISYN--SHYFTVAGVTHNMM------------F-------------LQYLPSL

--------------KVLNM-SFNSI----------------------------------N

TLTTKTMSSKSLRELQFR------------------------------------------

-----GNKLGRMWRDKDNTYVMIFKNFTNL-IH----LDISNN----------SIGKIP-

-----------------------YTVYTQ-------------------------------

------------------------------------------------------------

------------------------------------------------------------

-------------------------LPITI---QRLQMS---HNQLA-------------

----NI------------------NWTM----LRRFQNLRE-LILHD----NNII---EI

A--SNLSVDV-PSLELLNLQHN--RISKLAIGF-LQG----VVNLKELDLSYN-YLITVN

Q-STFPTE------------------------------SDLK-MLWLHG----NPFHCT-

--CNLLE-----------------------------------------------F--VLW

IL-----DTNVK-------------IPRL-------VTGVTC---TMPE----ER---KG

LAVIK-----------------FDI-Q-ECID------DQ--------------------

---------LAFVAYFISAACIICTTFAAIT--M---HLFYW-DVSYLYY---------Y

LKA--RFTG----YQQLSSESC----------------IYDAFITYDTKDPQ--VSDW-V

LNHL--RVQLE------ERSE----LFL---PICLE-ERDWIPGSP-VLDSLTQ-SIQNS

RKTVFVLTEG---YVNSGSF-KLAVFLAHQ--RLLEENE---DV----IVLLLLEPVL-Q

HS---HFVRLRRRLCARSILEWP-----HSSS--AEAWFWQSLRNAIR------------

---------------------------------------------------------VDN

QALYSELYSRYFTTK---------------------------------------------

------------------------------------------------------

>TLR7_Rhty

------------------------------------------------------------

------------MLILLVYKQKLERENLVVVNSARALLL---------------------

---------HVLFLFISVSKSTANI-----------------------------------

------------------------------------WFPKSLPCDVTKNGPAVVVDCSER

E-----------LTHIP-----------TGFPSNATNISLTI------------------

------------------NHILEVHST-SFSGL-S----NLTEIDLRCNCV-PVRLGPKD

RVCTKPLRVQKGAFSS----LPALRSLYL---DGNQLNE-------------LPQGL---

----------PHTLTMLSLEA--------------------------NSIF---------

--------VLTKANLSV-MSNLEA---------------------IYLGQNCYYRNPCNI

SY-----------------------------------------------QIEQDAFYGLS

N--------------LTTLSLKDNNL-----TYI--PGKLPSSLKLLLLYNNVIKKVN--

-DKDFAELPELEILDLSGNCPRCYNAPYPCQPC--QSPSYIQIHPNAFQTLNKLKILRLQ

SNSLSTVS-SSWFKNTSNLQILDLSEN-------------------------FLLKEIGN

AAFLKYLTKLEVIDL----SFNYELKLYA-KYLNLSKNFSKLKSLQSLRIRGYVFKSLKD

EYLKPLWKLR--NLKL-----------LDLGTNFIKV-----------------------

---ANLQIFNKFEGLKLLDLSENKISPSSGESLSA---------------------WCGS

NEPSQAAGAFYDWSDQMHYFRYDGYGRSCKFKAEKEASYPFVPSRQTECGSYSSTL----

----------DLSRN---------------------------------------------

--NIFF-----VGPTLFE--GL---S------SLRCLNLSG--NA--LSQTLNGSE----

----FQHLPD-----------------------------LLYLD---------------L

SNNRI-DLLYET----------------------------------AFQELK-KL-----

---RVLDLSHN-----SHYFEMEGLTH------------RL----------GFIQNLTAL

--------------SKLFL-NGNAI----------------------------------H

SSADTVLRSRSLQVLEFR------------------------------------------

-----GNRLDYMWRDGTKRYLRLFGGLDNL-TR----LDLSSN----------LLTFVP-

-----------------------SPVFEH-------------------------------

------------------------------------------------------------

------------------------------------------------------------

-------------------------LPPQL---EELVLA---TNRLR-------------

----SF------------------HWSG----LRLLNSLRL-LDLGG----NQLS---CV

P---RSLSDYATGLRCLVLAGN--RIAHLGRYF-LRG----AASLRELDLSSN-RLRTID

A-SSLPPPRPLEEA-------------------GKGSWAGLR-VLRLDG----NPFACN-

--CDAAS-------------------------------------------W---L--AWW

LN-----RTSVH-------------VPRL-------ATGVTC---ASPR----VH---LG

RSVLS-----------------LDR-R-ACEL------DS--------------------

---------LGAGLHAATAALTLLLLLVSLAA-----RRLSW-HARYAYY---------L

CGA--KLRG----YRRLPSSPTEST-------------PYAAFVAYDTHDSL--VTDW-V

LKEL--ITHLE-----ESGDR----RL----CLCLE-DRDWVPGRL-VLENLSH-SIHRS

RKTVFILTRP---YVATGQF-RTAFHMAYQ--RLLDEKL---DV----IVLVLLDKVW-Q

RS---RYLRLKKRLSRHSVLEWP-----RNPR--AQPLFWQRLRNTLT------------

------------------------------------------------------TDSRLQ

RGKLFNEIV---------------------------------------------------

------------------------------------------------------

>TLR7_Dare

------------------------------------------------------------

----------------------------MTEKTMIIFAS---------------------

---------FISLLVAAEWY----------------------------------------

------------------------------------PKSLKCDVSLASNGTEVSVDCTER

S-----------LTEVP-----------LGIPTNTTNLTLTI------------------

------------------NHIPHVMNN-SFDNL-H----NITEIDLRCNCV-PVKVGPKD

RVCSQSVSIDNGTFWK----LKNLKSLYL---DGNQLSS-------------IPKGL---

----------PANIVLLSLEI--------------------------NSIY---------

--------SILQENLTE-LTNIRT---------------------LYLGQNCYFRNPCNQ

SY-----------------------------------------------YIEKDAFMLLD

K--------------MTLLSLKSNNL-----SYI--PNQLPSSLKELYLYNNNIEKIT--

-ENDFCNLTELEVLDLSGNCPRCYNAPFPCIPC--PNNAPLQIHPNSFKTLRNLKTLRLH

SNSLTNIP-PEWFQSLADLTLLDLSSN-------------------------FLAKEITC

TSFPSLLPKLEELDL----SFNYELQVYP-ASLSLSESFSQLKSLRVLRIRGYVFQELKL

QDIQPLTNLT--YLEF-----------LDLGTNFIKI-----------------------

---AQLSILKNLKNFKIINLSDNKISVPSEGEFSFSNHREAYYGSPMSQGAQYHNGEVKD

MHYFLYDEFARSCKYKDKE-------------------LWIPSPFNNDCSSFGKTL----

----------DISRN---------------------------------------------

--NIFF-----LHSKFL---NL---G------ELRCLNLSG--NA--MSQSLNGSE----

----FVQLTN-----------------------------LQYLD---------------F

TDNRL-DLMYPS----------------------------------AFQELS-NL-----

---VVLDISRN-----SHYFVAEGLTH------------ML----------NFTENLSKL

--------------RKLIM-NDNQI----------------------------------S

TSTNTEMKSYKLEHLEFK------------------------------------------

-----GNRLDMLWRDGDTRYVNYFKNLMSL-KT----LDISRN----------NLNFIP-

-----------------------LVVFQG-------------------------------

------------------------------------------------------------

------------------------------------------------------------

-------------------------LPNTL---TKLYIT---DNKLK-------------

----LF------------------KWEG----LVYLKSLLL-LDLTG----NLLT---EV

P---SCLSNYTKSIQTLVLSKN--KIVKLSPNF-LKD----AFSLKILDLSYN-SIQFID

E-SSFPEN-----V-----------------------IDHLQ-TLYLNN----NMFVCS-

--CNAT--------------------------------------------W---L--VRW

IN-----RTSVN-------------IPRL-------ASDVTC---ASPS----AQ---KG

QSVIF-----------------LNL-Q-ACQH------NS--------------------

---------LSIILCIFQTTLILTILTLTISS-----HLFLW-DVWYIYH---------F

CLA--KLKG----YRRLSSNSA----------------VYDAFVIYDTTDPA--VQEW-V

MQEL--RVHLE-----DKGDP----RM----NLCLE-ERDWVPGCP-LIENLSQ-SIQLS

QRTVFILTER---YIRSGSF-RTAFYLAHQ--RLMDERN---DV----IVLIFLERMP--

--CHSKYLRLRKRLCIKEL---------KGTSGSVSGALW-RLRVNII------------

---------------------------------------------------------HSS

RRVL--------------------------------------------------------

------------------------------------------------------

>TLR7_Xetr

------------------------------------------------------------

-------------------MHGKTFKVFYFGMRRQLLFF---------------------

---------LISILSFSGLLATNWF-----------------------------------

------------------------------------PKSLPCDVEQNAKGNVIVVDCSDR

H-----------LTSIP-----------WGIPTNVTNLTLTI------------------

------------------NHIPRISVD-SFAEF-T----NLVELDFRCNCV-PAKVGPKD

HVCTKRLDVEDRSFAS----LYNLRSLYL---DGNQLIE-------------FPKGL---

----------PPNLQLLSLEI--------------------------NNII---------

--------SISRNNLSE-LSNIQM---------------------LYLGQNCYHRNPCSD

SF-----------------------------------------------KIEKDAFKDLK

N--------------LSILSMKSNNL-----SFV--PGGLSDSLKELYLYNNAIQYIE--

-EHDLENLINLEILDLSGNCPRCYNSPFPCTPC--PNNAPIQIHPKAFSSLKNLQVLRLH

SNSLRSIP-EQWFKNNRNLQVLDLSEN-------------------------FLASEIST

ANFLKYIPSLKSLDL----SFNFELQVYP-SDLKLSSIFSSLASLETLRIRGYVFQNLKK

NNLMPLVHLP--NLTL-----------LDLSTNFIKV-----------------------

---ADFSLFPKFKSLQTIILSNNKISPSSEANIDSCSASQVS--------------SGHY

IGRTFQEVHYFEYDENARKCKAKDK-----------ENFTFKLFLNESCQAYGQSL----

----------DLSQN---------------------------------------------

--NIFF-----VKATDFT--NL---S------FLKCLNLSG--NA--ISQTLNGSE----

----FRNLNR-----------------------------LKYLD---------------F

SNNRI-DLLYST----------------------------------AFQELT-EL-----

---EVLDISNN-----DHYFLAEGITH------------VF----------NFTKNLEKL

--------------TKLMM-NNNQI----------------------------------S

TSTNRHLVSQSLRILEFK------------------------------------------

-----GNYLNILWKDGDTRYLNFFKNLNKL-YK----LDISEN----------SLTFVP-

-----------------------PGVFEG-------------------------------

------------------------------------------------------------

------------------------------------------------------------

-------------------------MPPDL---LELYLA---RNKLK-------------

----TF------------------SWDK----LHLLEKLSV-LDLSN----NYLT---TV

P--RELSNCT-SSIKKLILSNN--KIKKLTPFF-LRG----SVSLKYLDLSDN-LIQNIG

H-SSFPED-----V-----------------------LDNLT-ELLLQG----NPFKCN-

--CNLV--------------------------------------------W---L--VSW

IN-----QTKVY-------------IPNL-------VTGVTC---SGPG----AH---RG

QSLVL-----------------LDL-Y-TCEQ------YH--------------------

---------LNLILHALSASFIICLMVVSVSS-----HLFYW-DFWFIYH---------L

FKA--KIHG----YKRFPKC------------------CYDALIMYDTKDSA--VSDW-V

FNDL--VNILE------KQGN----KML---NLCLE-ERDFLAGQP-FLDNLSE-SIQIS

RKTVFVLTRK---YVKKGHF-KTAFYMAHQ--RLIEEKV---DV----IILILLEKTL-Q

RS---RYLRLRKRLCANSVLYWP-----SNPN--SQSYFWHCLKSAIA------------

-----------------------------------------------------TENQMGY

DKLFKDHT----------------------------------------------------

------------------------------------------------------

>TLR7_Hosa

------------------------------------------------------------

-------------------------MVFPMWTLKRQILI---------------------

---------LFNIILISKLLGARWF-----------------------------------

------------------------------------PKTLPCDVTLDVPKNHVIVDCTDK

H-----------LTEIP-----------GGIPTNTTNLTLTI------------------

------------------NHIPDISPA-SFHRL-D----HLVEIDFRCNCV-PIPLGSKN

NMCIKRLQIKPRSFSG----LTYLKSLYL---DGNQLLE-------------IPQGL---

----------PPSLQLLSLEA--------------------------NNIF---SIR---

--------KEN---LTE-LANIEI---------------------LYLGQNCYYRNPCYV

SY-----------------------------------------------SIEKDAFLNLT

K--------------LKVLSLKDNNV-----TAV--PTVLPSTLTELYLYNNMIAKIQ--

-EDDFNNLNQLQILDLSGNCPRCYNAPFPCAPC--KNNSPLQIPVNAFDALTELKVLRLH

SNSLQHVP-PRWFKNINKLQELDLSQN-------------------------FLAKEIGD

AKFLHFLPSLIQLDL----SFNFELQVYR-ASMNLSQAFSSLKSLKILRIRGYVFKELKS

FNLSPLHNLQ--NLEV-----------LDLGTNFIKI-----------------------

---ANLSMFKQFKRLKVIDLSVNKISPSGDSSEVGFCSNARTS-------------VESY

EPQVLEQLHYFRYDKYARSCRFKNK-------------EASFMSVNESCYKYGQTL----

----------DLSKN---------------------------------------------

--SIFF-----VKSSDFQ--HL---S------FLKCLNLSG--NL--ISQTLNGSE----

----FQPLAE-----------------------------LRYLD---------------F

SNNRL-DLLHST----------------------------------AFEELH-KL-----

---EVLDISSN--SHYFQSEGITHMLN------------F-------------TKNLKVL

--------------QKLMM-NDNDI----------------------------------S

SSTSRTMESESLRTLEFR------------------------------------------

-----GNHLDVLWREGDNRYLQLFKNLLKL-EE----LDISKN----------SLSFLP-

-----------------------SGVFDG-------------------------------

------------------------------------------------------------

------------------------------------------------------------

-------------------------MPPNL---KNLSLA---KNGLK-------------

----SF------------------SWKK----LQCLKNLET-LDLSH----NQLT---TV

P--ERLSNCS-RSLKNLILKNN--QIRSLTKYF-LQD----AFQLRYLDLSSN-KIQMIQ

K-TSFPEN-----V-----------------------LNNLK-MLLLHH----NRFLCT-

--CDAV--------------------------------------------W---F--VWW

VN-----HTEVT-------------IPYL-------ATDVTC---VGPG----AH---KG

QSVIS-----------------LDL-Y-TCEL------DL--------------------

---------TNLILFSLSISVSLFLMVMMTAS-----HLYFW-DVWYIYH---------F

CKA--KIKG----YQRLISPDC----------------CYDAFIVYDTKDPA--VTEW-V

LAEL--VAKLE-----DPREK----HF----NLCLE-ERDWLPGQP-VLENLSQ-SIQLS

KKTVFVMTDK---YAKTENF-KIAFYLSHQ--RLMDEKV---DV----IILIFLEK---P

FQKS-KFLQLRKRLCGSSVLEWP-----TNPQ--AHPYFWQCLKNALA------------

---------------------------------------------------------TDN

HVAYSQVFKETV------------------------------------------------

------------------------------------------------------

>TLR7_Mumu

------------------------------------------------------------

-------------------------MVFSMWTRKRQILI---------------------

---------FLNMLLVSRVFGFRWF-----------------------------------

------------------------------------PKTLPCEVKVNIPEAHVIVDCT-D

K----------HLTEIP-----------EGIPTNTTNLTLTI------------------

------------------NHIPSISPD-SFRRL-N----HLEEIDLRCNCV-PVLLGSKA

NVCTKRLQIRPGSFSG----LSDLKALYL---DGNQLLE-------------IPQDL---

----------PSSLHLLSLEA--------------------------NNIF---SIT---

--------KEN---LTE-LVNIET---------------------LYLGQNCYYRNPCNV

SY-----------------------------------------------SIEKDAFLVMR

N--------------LKVLSLKDNNV---TAVPT--T--LPPNLLELYLYNNIIKKIQ--

-ENDFNNLNELQVLDLSGNCPRCYNVPYPCTPC--ENNSPLQIHDNAFNSLTELKVLRLH

SNSLQHVP-PTWFKNMRNLQELDLSQN-------------------------YLAREIEE

AKFLHFLPNLVELDF----SFNYELQVYH-ASITLPHSLSSLENLKILRVKGYVFKELKN

SSLSVLHKLP--RLEV-----------LDLGTN-FIK-----------------------

--IADLNIFKHFENLKLIDLSVNKISPSEESREVG---------------------FCPN

AQTSVDRHGPQVLEALHYF----RYDEYARSCRFKNKEPPSFLPLNADCHIYGQTL----

----------DLSRN---------------------------------------------

--NIFF-----IKPSDFQ--HL---S------FLKCLNLSG--NT---IGQTLNGS----

----ELWPLR------------E----------------LRYLD---------------F

SNNRL-DLLYST----------------------------------AFEELQ-SL-----

---EVLDLSSN--SHYFQAEGITHMLN------------F-------------TKKLRLL

--------------DKLMM-NDNDI----------------------------------S

TSASRTMESDSLRILEFR------------------------------------------

-----GNHLDVLWRAGDNRYLDFFKNLFNL-EV----LDISRN----------SLNSLP-

-----------------------PEVFEG-------------------------------

------------------------------------------------------------

------------------------------------------------------------

-------------------------MPPNL---KNLSLA---KNGLK-------------

----SF------------------FWDR----LQLLKHLEI-LDLSH----NQLT---KV

P--ERLANCS-KSLTTLILKHN--QIRQLTKYF-LED----ALQLRYLDISSN-KIQVIQ

K-TSFPEN-----V-----------------------LNNLE-MLVLHH----NRFLCN-

--CDAV--------------------------------------------W---F--VWW

VN-----HTDVT-------------IPYL-------ATDVTC---VGPG----AH---KG

QSVIS-----------------LDL-Y-TCEL------DL--------------------

---------TNLILFSVSISSVLFLMVVMTTS-----HLFFW-DMWYIYY---------F

WKA--KIKG----YQHLQSMES----------------CYDAFIVYDTKNSA--VTEW-V

LQEL--VAKLE-----DPREK----HF----NLCLE-ERDWLPGQP-VLENLSQ-SIQLS

KKTVFVMTQK---YAKTESF-KMAFYLSHQ--RLLDEKV---DV----IILIFLEKPL-Q

KS---KFLQLRKRLCRSSVLEWP-----ANPQ--AHPYFWQCLKNALT------------

---------------------------------------------------------TDN

HVAYSQMFKETV------------------------------------------------

------------------------------------------------------

>TLR7_Chpi

------------------------------------------------------------

------------------MRLNDEMTLLAFHTWPSNRLL---------------------

---------FLLLFLFSKLLSARWF-----------------------------------

------------------------------------PKSLPCDVKVEASKANVIVDCSDR

R-----------LTKIP-----------PGIPSNTTNLTLTI------------------

------------------NHIPNIYPI-SFVHL-D----NLVEIDFRCNCV-PVRLGPKD

HVCTRRLQIQNSSFAT----LTKLESLYL---DGNQLSE-------------IPRGL---

----------PPNLRLLSLEA--------------------------NSIF---SIT---

--------KEN---LSE-LGNIEM---------------------LYLGQNCYYRNPCNV

SF-----------------------------------------------EIEEEAFQDLR

N--------------LTVLSLKANNL-----TYI--PHNLSSTLKELYLYNNMIQKVQ--

-EHDLNDLYNLEILDLSGNCPRCYNAPFPCTPC--PNNAPIWIHHKAFDALKQLKILRLH

SNSLHNVP-SSWFKNTKNLKVLDLSQN-------------------------FLAKEIGE

ACFLNFIPNLVELDL----SFNFELQVYS-SFLNLSKTFSSLSHLEILRVKGYVFR---E

LSQENLRPLL--HLRNLTV--------LDLGTNFIKL-----------------------

---ANLSMFKKFPSLKMIDLSVNKISPSSGEFNNHGFCS-----------------IPMA

SVDQYKTQMVQEMHYFRYD----EY--GRSCKSKDKESASFQPFVNEDCLSYGETL----

----------DLSRN---------------------------------------------

--NIFF-----INPSDFQ--HL---T------FLKCLNLSG--NA--ISQTLNGSE----

----FYPLSG-----------------------------LKYLD---------------F

SNNRI-DLLYSA----------------------------------AFQELK-DL-----

---EILDLSDN--KHYFLAEGITHKLD------------F-------------TKNLTFL

--------------KKLMM-NGNEI----------------------------------S

TSTNMGMESHSLQTLEFK------------------------------------------

-----GNHLDVLWRDGDTRYLSFFKNLTNL-EQ----LDISYN----------SLRFLP-

-----------------------PGVFEG-------------------------------

------------------------------------------------------------

------------------------------------------------------------

-------------------------MPPRL---KVLSLT---NNMLK-------------

----SF------------------NWGK----LHFLEKLEA-LDLSN----NLLS---TV

P--RELSNCS-ATLHKLILQNN--RIRRLTKYF-LRD----AFQLKYLDLSSN-KIQIIK

K-SSFPEN-----V-----------------------ISNLE-MLLLHG----NPFKCI-

--CDAV--------------------------------------------W---F--VWW

IN-----QTEVT-------------IPLL-------ATDVTC---AGPG----AH---KG

KSVVL-----------------LDL-Y-TCEL------DS--------------------

---------SHVILYSVSASAILCLMVFTVTS-----HLYFW-DVWYSYH---------F

CTA--KIKG----YRRLHSPET----------------CYDAFISYDNEDPA--VTEW-I

LKEL--VENLE-----DQKDK----QF----NLCLE-ERDWLPGQP-VLDNLSQ-SIQLS

RKTIFVLTNK---YTTSGNF-KTAFYMAHQ--RLMDEKV---DV----IILIFLERAL-K

KS---KYLRLRKRLCSSSVLEWP-----TNPR--SQCYFWQCLKNALA------------

---------------------------------------------------------TNN

DMTYNKLFRETV------------------------------------------------

------------------------------------------------------

>TLR7_Gaga

------------------------------------------------------------

---------------MTNLSEVAAHRKMVHHARTSNALL---------------------

---------FVLLFLFPMLLSGR-------------------------------------

------------------------------------WFPKTLPCDVEAFESTVRVDCSDR

R-----------LKEVP-----------RGIPGNATNLTLTI------------------

------------------NHIPRISPA-SFTQL-E----NLVEIDFRCNCV-PPRLGPKD

NVCVTPPSIENGSFAA----LTRLKSLYL---DANQLSK-------------IPRGL---

----------PATLRLLSLEA--------------------------NNIF---SIK---

--------KNT---FSE-LRNIEL---------------------LYLGQNCYYRNPCNV

SF-----------------------------------------------EIEETAFLNLK

N--------------LTVLSLKSNNL-----TFI--PPNLSSTLKELYIYNNRIQEVQ--

-EHDLSNLYNLEILDLSGNCPRCYNAPYPCTP---CPNISIKIHSKAFYSLKKLRILRLH

SNSLQSIP-SSWFKNIKNLKNLDLSQN-------------------------FLIKEIGD

AEFLKLIPSLVELDL----SFNFELQMYS-PFLNLSKTFSCLSNLETLRIKGYVFKELRE

ENLDPLLNLR--NLTV-----------LDLGTNFIKI-----------------------

---ADLRVFKKFRSLKIIDLSMNKISPSSGEGNFYGFCSDHRIT------------VEQY

SRHVLQEMHYFRYDEYGRSCKSKDK-----------EADSYQPLVNGDCMSYGETL----

----------DLSRN---------------------------------------------

--NIFF-----VNSIDFQ--DL---S------FLKCLNLSG--NA--ISQTLNGSE----

----FYYLSG-----------------------------LKYLD---------------F

SNNRI-DLLYST----------------------------------AFKELK-FL-----

---EILDLSNN--KHYFLAEGVSHVL-------------------------SFMKNLAYL

--------------KKLMM-NENEI----------------------------------S

TSISTGMESQSLQTLEFR------------------------------------------

-----GNRLDIFWSDGKKEYLSFFKNLTNL-EQ----LDISSN----------MLNFLP-

-----------------------PDVFEA-------------------------------

------------------------------------------------------------

------------------------------------------------------------

-------------------------MPPEL---KILNLT---SNRLH-------------

----TF------------------NWGK----LHLLTKLIT-LDLSN----NLLT---TV

P--RKLSNCT-STLQELILRNN--RITRITKYF-LRG----AIQLTYLDLSSN-KIQIIK

K-SSFPEN-----I-----------------------INNLR-MLLLHN----NPFKCN-

--CDAV--------------------------------------------W---F--VGW

IN-----QTQVA-------------IPLL-------ATDVTC---AGPG----AH---KG

RSLVF-----------------LDL-N-TCEL------DT--------------------

---------SYFIMYALSTSAVLCLMMFAVMS-----HLYFW-DVWYSYH---------Y

CTA--KLKG----YRRIPLPDA----------------CYDAFIAYDNTDLA--VNEW-V

MTEL--VEKLE-----DQKAR----QF----NLCLE-ERDWLPGQP-VFDNLSQ-SIQLS

KKTIFVLTNK---YIKSGTF-KTTFYMAHQ--RLLDEKI---DV----IILIFLEKVL-Q

KS---RYVQLRKRLCRSSVLEWP-----TNPR--SQPYFWQRLKNAIA------------

----------------------M---------------------NN--------T--LSY

NKLLQETV----------------------------------------------------

------------------------------------------------------

>TLR8_Rhty

------------------------------------------------------------

---------------MGIFQYLKIKEKMVKPTSASALIL---------------------

---------LCFLFLLKIVVPLTRSWI---------------------------------

------------------------------------PRSLPCDVSIQNNGYSIQVDCRNS

R-----------LRSIP-----------ANIPANATSLNLAN------------------

------------------NQISNISSY-QFSNL-Q----NLTKLDLSQNVS---------

--PGKNMNIAEDSFSS----LIKLQELYL---DQNHLKR-------------IPKKL---

----------SFSLQLLSLKG--------------------------NKIL---------

--------NITKEDLPN-LSNIET---------------------LHLSQNCYYHNPCKV

SN-----------------------------------------------YIHDGAFSSGN

K--------------LKNLSLGSNNLTSV-------PRNLPETLSSLYLNKNKIQIIN--

-HDHFERLIDLEFLDLSENCPRCFNAPFPCERC--PGDSSIHIHPDAFQRLPKLQTLILS

SNSLRKIQ-SSWFQNCTSLKVLYLQMN-------------------------FLIDEIAT

GDFLNHLPRLEKIDL----SFNYKITHYF-KNINLSKNFSKLVSLTELNIKGYVFKELDY

QDLKPLRHLH--NLTV-----------LNLGTNFIKQ-----------------------

---ANLTIFKNIKSLKIIDLSENRISPPSVNRNLLH--------------------DSTY

KGKSLPYVESSLQKSNLLPWDSHLGHESLNNDDDYIFGPNMKR----DCHSYGMTL----

----------DLSLN---------------------------------------------

--SIFF-----ISPKQFK--GL---E------KIRCLNLSS--NA--IGQALNGTE----

----FASLSN-----------------------------LKYLD---------------L

SHNRLDPAYDN-----------------------------------AFKELW-QL-----

---EVLDLSYN-----PHYFVVEGVTH----------------------KMGFIENLKYL

--------------KKLNL-GYNAI----------------------------------F

TLTEKGLNSTSLEVLKFH------------------------------------------

-----GNRLDILWKREDYRYISLFKNLINL-TH----LDLSYN----------KLECIP-

-----------------------SEIYEN-------------------------------

------------------------------------------------------------

------------------------------------------------------------

-------------------------LPYNL---SYLSLS---HNKLR-------------

----KF------------------SWPP----LRFLKNLLT-LDLSY----NYLT---IA

P--DRLYNST-KSLQKLLLKQN--RISQLPISF-LTS----ANRVKYLDLSYN-KIHMLQ

Q-TVFPRS----------------------------EQLFLE-VLVLKG----NPFYCI-

--CELVP-----------------------------------------------F--IAW

IN-----TCDLD-------------IPQL-------ATDVTC---GSPE----SQ---RG

QSIIL-----------------LDR-H-TCAM----------------------------

---------DDVAASLSLASAIIIFCTTFIAVTH---HLFYW-DVWYLYN---------F

CAS--RLKQ--YQYHTFKVQSC----------------SYDAFVAYDTSDLA--VTDW-I

VNEL--LVHLE------DKDER---QL----CLCLE-ERDWELGMP-VVENLSQ-SIHKS

KKTVFVLTRR---YVKSGSF-KTAFYMAHQ--RLMDKNE---DV----IVLILLEPVL--

--INSKYLKLRKRLCRSSVLYWP-----KNPN--SEDFFWHCLRNAIA------------

---------------------------------------------------------ANN

NSKYNTIAEFS-------------------------------------------------

------------------------------------------------------

>TLR8.1_Xetr

------------------------------------------------------------

-------------------------------MPPLLQTA---------------------

---------LLVVVTTCNSLAYSDMT----------------------------------

------------------------------------PNNRTIPCRITENGFFVSFDCSAR

R---------LKMVPHPITYNS-----------DSAELLLSQ------------------

------------------NLILTINNE-SFYSW-H----NLTKIDLNWNHY-PKSRLNNT

DLCKRGLEIGNETFSY----LTKLEELFI---DHNFLCK-------------IPQGI---

----------PSTVQTLSLSY--------------------------NNIF---------

--------SVKKQILSP-LINLKK---------------------LFLNHNCYFGNECDH

VV-----------------------------------------------DIEEGTFAGLT

E--------------LTVLDLSFNNM-----TRV--PAKLPASLKELYLSNNNIQIIH--

-SDDLQNLVNLEVLFLNGNCPRCFNANYPCKNL--CKQISITIDPLAFQNLKNLTELHLG

STSLRTIP-PIWFQNTTQLKILNLQLN-------------------------YLVNEIAS

ADFLLNLPFLEVLDL----SFNYDLRSYA-NNINISNHFSKLISLQELHIQGYVFKHIAA

KNLSPLINLP--KLAT-----------INLGINFIKQ-----------------------

---VDFEVFQNISGLKLIYLSENRITPFSETNKQLFGVC-----------------KDQS

YRISSPGVTFPTQSNFQMT-------------------KIFSHLVKPQCSSRGKTL----

----------DLSLN---------------------------------------------

--SIFF-----IDPEEFR--SF---S------DVSCLNLSF--NS--IGQDLNGTE----

----FIYLKN-----------------------------LTYLD---------------L

SFNKL-DFDSIN----------------------------------AFQELP-SL-----

---EVLDLSYN-----SHYFIVDGVTH------------RL----------KFIENLQYL

--------------KVLNL-SWNKI----------------------------------S

TLTDFRLTSHSLKELQFS------------------------------------------

-----GNRLDVLWKNEDKRYQELFKNLYNL-TC----LDISYN----------RLNKMT-

-----------------------EVELSN-------------------------------

------------------------------------------------------------

------------------------------------------------------------

-------------------------LPLSL---TELYLN---NNKLD-------------

----YF------------------GWKA----LKAYKNLKH-LDLSH----NKLT---MI

M--GNLSIHT-YSLSSLIISYN--SISSLPVAF-LHK----SRNLSELDLSFN-HLKSIN

S-SVFLSG----------------------------SENYLT-VLGLKG----NPFVCT-

--CEITD-----------------------------------------------F--ISW

IY-----ANNVT-------------IPRL-------ATDVNC---AAPE----NK---KG

SGIIF-----------------FDV-H-TCDL------DG--------------------

---------AAMILYFFSVFLVMSITVLPIL--I---NVFSW-DLWYVYH---------L

CVA--KLR-----LHKVCKSKC----------------LYDVFITYDNKDPN--VSDW-I

FNEL--CQHLE-----DKGDK----HM----YLCLE-ERDWEPGKA-IIDNLAH-SINQS

NKTLFVLTKK---YVKSGKF-KTAFYLALQ--KLMDENM---DV----IVIVLLEPVL-Q

NS---QYLRLRRKICKSSIMEWP-----KNPN--TKSFFWQRMKNVLL------------

-----------------------------------------------------TDNCNRY

NNFYTDTIAN--------------------------------------------------

------------------------------------------------------

>TLR8.2_Xetr

------------------------------------------------------------

----------------MDVRIIGEGEEGKAKMTGSLQTL---------------------

---------VLVVLITCNSLECSDTN----------------------------------

------------------------------------PNNRTIPCKIAENGSSVSFDCS-A

R--------WLQMVPYPIKYSS-----------DSVELLLSQ------------------

------------------NLILTINNE-SFHSW-H----NLTKIDLNWNHY-PKSRLDNA

DICKRGLEIENGTFSY----LTKLEKLFI---DHNYLCK-------------IPQGI---

----------PSTLTFLSLSY--------------------------NNIF---------

--------SVKKQILSP-LINLKN---------------------LFLSNNCYFGNECGQ

VL-----------------------------------------------DIEEGTFSGLT

E--------------LTELSLSFNNL-----THV--PSKLPASLKQLYLSNNNIQIIN--

-RNDFHNLVNLEVLYLSGNCPRCFNANYPCKNL--CPNTSITIDHFAFQNLKNLTELHLS

STSLKTIP-PTWFQNTTQLKKLYLERN-------------------------YLVNEIAS

ADFLLNLPFLEVLDL----SFNYDLRSYT-NNINISDHFSKLVSLKELHIQGYVFKHIAA

NNLAPLLNLS--KLKI-----------LNLGTNFIRQ-----------------------

---VDFKIFQQFTGLELIYLSENRITPFSEKNNKMKLVEGY---------------EDKH

SRVSSPGVSFPTQFNFQMT-------------------KTFSEVVKPQCSSRGKTL----

----------DLSLN---------------------------------------------

--SIFF-----IDPKEFR--SF---S------DVSCLNLSS--NG--IGQDLNGTE----

----FIYLKN-----------------------------LTYLD---------------L

SFNKL-DFDSIN----------------------------------AFQELP-SL-----

---EVLDLSYN-----SHYFIVDGVIH------------SL----------KFIENLQHL

--------------KVLNL-SWNKI----------------------------------S

TLTDFRLTSHSLKELQFS------------------------------------------

-----GNRLDVLWKNEDKRYHKLFMNLSSL-TC----LDISYN----------RLSKIK-

------------------------GELRY-------------------------------

------------------------------------------------------------

------------------------------------------------------------

-------------------------LPLSL---TELYLN---NNELV-------------

----YF------------------GWEE----LQAYENLKY-LDLSH----NKLT---MI

M--GNLSIHT-YSLSSLIISYN--SISSLPVAF-LHK----ARNLSELDLSFN-HLKSIN

S-SVLLSG----------------------------SENYLK-VLGLKG----NPFVCT-

--CEITD-----------------------------------------------F--ISW

IY-----ANNVT-------------IPRL-------ATDVNC---ATPE----NK---KG

SGIIY-----------------FDV-H-TCDL------DG--------------------

---------AAMILYFFSVFLVMSITVLPIL--I---NVFSW-DLWYVYH---------L

CVA--KLR-----LHKVCKSEC----------------LYDVFITYDNKDPN--VNDW-I

FNEL--CQHLE-----DKGDK----HM----YLCLE-ERDWEPGKA-IIDNLAH-SINQS

KKTLFVLTKK---YVKSGKF-KIAFYLALQ--KLMDENM---DV----IVIVLLEPVL-Q

NS---QYLKLRRKICKSSIMEWP-----KNPN--AKGLFWQRMKNVLL------------

----------------------T---------------------DN--------C--KRY

NNFYTDPIENYE------------------------------------------------

------------------------------------------------------

>TLR8.2_Chpi

------------------------------------------------------------

-------------------------------MAAISYKL---------------------

---------LCLLFMSHVESGIFYYPWV--------------------------------

------------------------------------SKKVPCKVHVENSSSSIIFDCR-H

Q----------HLKAVPLEIND-----------NATCLILSY------------------

------------------NRIKNVSNV-IFQKF-Q----NLTQLYLNYNAI---------

---NQSMAQPLGLFRN----LTKLEKLAL---SHNHLQE-------------VPKGL---

----------SPSITSLELNA--------------------------NKIV---SIK---

--------NNT---FSE-LKNLKE---------------------LYMDRNCYYSNPCGK

TF-----------------------------------------------EIEDEAFVALT

N--------------LTVLSLSYNNL-----TRV--PLNLPSSLRELYLGFNRITRIS--

-QGDFNELVNLHLLDLSRNCPRCYNAPFPCEPC--TINSSIQIHQFAFQNLNKLKTLVLT

STSLTSVP-AIWFQNMTQLKVLHLAFN-------------------------YLQNEIAS

GEFLRELTSLQELDL----SFNFEEQVYL-SYLNLSQHFSSLISLKRLYIKGYVFQDLCE

KHLKPLVALK--KLNI-----------LDLGINFIKQ-----------------------

---IDLAVFQNFSNLTEIYLTDNRISPFVGDN------------------------NCLL

EQVGKKAFRKYCHLTLERE----GQPSPSVMQKYKKIDSFLYYILRPQCSSYGKAL----

----------DMSSN---------------------------------------------

--SLIF-----INPNQFK--SF---K------DIACLNLSS--NG---INQAFNGT----

----EFNLTK-----------------------------LKYLD---------------L

SNNKLDFAYGF-----------------------------------AFNEMK-LL-----

---EVLDLTHN-----KHYFRLAGITL------------RL----------TFIEKLPQL

--------------KVLNL-SWNAI----------------------------------S

RLTDRQLSSKSLEELVFK------------------------------------------

-----GNCLDILWGDKHERYIHFFKNLGSL-TY----LDISHN----------RLLKIP-

-----------------------TRAFLS-------------------------------

------------------------------------------------------------

------------------------------------------------------------

-------------------------LPPNL---TQLFLN---NNRLQ-------------

----VF------------------IFAN----LTRLKYLKL-LDLSQ----NNFK---TV

H---ISFK---QSLQSLLLRGN--RISEIALDF-SNT----NGSLLFLDLSHN-KLKYMN

Q-STLV-H-----I------------------------QGVK-YLKLKG----NPFDCT-

--CQNSD-----------------------------------------------F--IKW

IQ-----TTNIY-------------IPQL-------ATKVNC---AIPD----KH---RK

KSIVS-----------------IDL-H-ACAL------EE--------------------

---------VAATLFYVSFFVVINIMLIAVT--K---HFFYW-DVWYTYY---------I

CAA--KLKG----YKSTATDKA----------------LYDAYIAYDTQDAT--VTDW-V

INEL--RFRLE-----ENEDK----HV----LLCLE-ERDWEPGKA-VIDNLAQ------

------------------------------------------SI----------------

------------------------------------------------------------

------------------------------------------------------------

------------------------------------------------------------

------------------------------------------------------

>TLR8.1_Chpi

------------------------------------------------------------

------------------------------MNPTIPNLI---------------------

---------WRLLLAYGTSEILAET-----------------------------------

------------------------------------KYPRTLPCDVSVNNSSVIFDCSAR

Q-----------LRSVP-----------AAMHGNVTELKPSD------------------

------------------NLIKEVFKK-SFQGL-N----NLMKIDLNRNHY-SKGEEEAP

DLCKKGMVIENGAFAN----LTKLRELLA---DENHLCK-------------IPVGM---

----------PLSLTSLSLRY--------------------------NNML---------

--------SVCRQHFSE-LTQLKE---------------------LYMDGNCYYGNPCEK

PF-----------------------------------------------LADNGAFSDLT

I--------------LTVLSLAFNNL-----TRV--PSKLPSSLRKLYLNSNKIKTIN--

-QDDFNELSNVEVLDLSGNCPRCYNAPYPCEPC--SGDSAIQIHPLAFQHLKNLQNLNLS

STSLIRLP-ASWFYNTTQLKVLHLEFN-------------------------YLIKEIAS

GEFLLQLPYLEVLDL----SFNYARKSYP-RYINISDKFSNLVSLQQLHLRGYVFKELKS

KHLRPLINLT--KLHT-----------LNLGVNFIKQ-----------------------

---IDFSVFQLFANLTTISLSDNRISPILEGSN-----------------------NSVI

RRESVQNHVIQSRSTDTDLEPSVNSMLPAEGKSSSSVYNSIFPLIKPQCSMYGKSL----

----------DLSLN---------------------------------------------

--SIFF-----IDQEQFK--SF---H------DIACLNLSS--NG--IGQALNGTE----

----FIFLPN-----------------------------LKYLD---------------L

SFNKL-DLAYHY----------------------------------AFYELP-KL-----

---EVLDLSYN-----VHYFIVSGITH------------RL----------GFTENLPYL

--------------KVLNL-SYNAI----------------------------------F

TLTEPNLTSSSLKELVFK------------------------------------------

-----GNRLDILWKKGDNRYINIFKNLCNL-TH----LDISHN----------RLHEIP-

-----------------------TKAFCG-------------------------------

------------------------------------------------------------

------------------------------------------------------------

-------------------------LPQSL---IELHIT---NNELK-------------

----YF------------------DWSA----LQQFQNLTL-LDLSS----NELS---FV

T--DNLANCT-ASLQRLVLRQN--KISQLADGF-FNK----ASSLLHLDLSYN-ELPSIN

Q-SIRQYD----------------------------NFIYLE-LLDLKG----NPFECT-

--CATVD-----------------------------------------------F--KNW

IN----HYVNVS-------------IPRL-------ATDVIC---ATPG----DQ---RG

KSIIS-----------------LD----IYAC----------------------------

----------TLDNIAAICFSLSFFIILTIMTTAITKHLFYW-DAWYIYY---------F

CTA--KLKG----YKSLGMTKA----------------LYDAYIAYDTKDMA--VTDW-V

INEL--RFRLE-----ENEDK----HV----LLCLE-ERDWEPGKA-VIDNLAQ-SIHHS

RKTIFVLTER---YVKNGNF-KTAFYIALQ--RLMDENT---DV----IVFILLEPVL-Q

HS---QYLRLRRRICKSSVLDWP-----KNPH--AEGLFWQRLKSVVL------------

-----------------------------------------------------TENYKRY

NALYTDSIK---------------------------------------------------

------------------------------------------------------

>TLR8_Hosa

------------------------------------------------------------

----------------------------MFLQSSMLTCI---------------------

---------FLLISGSCELCAEE-------------------------------------

------------------------------------NFSRSYPCDEKKQNDSVIAECSNR

R-----------LQEVP-----------QTVGKYVTELDLSD------------------

------------------NFITHITNE-SFQGL-Q----NLTKINLNHNPN-VQHQNGNP

GIQSNGLNITDGAFLN----LKNLRELLL---EDNQLPQ-------------IPSGL---

----------PESLTELSLIQ--------------------------NNIY---------

--------NITKEGISR-LINLKN---------------------LYLAWNCYFNKVCEK

TN------------------------------------------------IEDGVFETLT

N--------------LELLSLSFNSL-----SHV--PPKLPSSLRKLFLSNTQIKYIS--

-EEDFKGLINLTLLDLSGNCPRCFNAPFPCVPC--DGGASINIDRFAFQNLTQLRYLNLS

STSLRKIN-AAWFKNMPHLKVLDLEFN-------------------------YLVGEIAS

GAFLTMLPRLEILDL----SFNYIKGSYP-QHINISRNFSKLLSLRALHLRGYVFQELRE

DDFQPLMQLP--NLST-----------INLGINFIKQ-----------------------

---IDFKLFQNFSNLEIIYLSENRISPLVKDTRQSYA-------------------NSSS

FQRHIRKRRSTDFEFDPHS----------------NFYHFTRPLIKPQCAAYGKAL----

----------DLSLN---------------------------------------------

--SIFF-----IGPNQFE--NL---P------DIACLNLSANSNA----QVLSGTE----

----FSAIPH-----------------------------VKYLD---------------L

TNNRL-DFDNAS----------------------------------ALTELS-DL-----

---EVLDLSYN-----SHYFRIAGVTH------------HL----------EFIQNFTNL

--------------KVLNL-SHNNI---------------------------------YT

LTDKYNLESKSLVELVFS------------------------------------------

-----GNRLDILWNDDDNRYISIFKGLKNL-TR----LDLSLN----------RLKHIP-

-----------------------NEAFLN-------------------------------

------------------------------------------------------------

------------------------------------------------------------

-------------------------LPASL---TELHIN---DNMLK-------------

----FF------------------NWTL----LQQFPRLEL-LDLRG----NKLL---FL

T---DSLSDFTSSLRTLLLSHN--RISHLPSGF-LSE----VSSLKHLDLSSN-LLKTIN

K-SALETK-----T-----------------------TTKLS-MLELHG----NPFECT-

--CDIGD-----------------------------------------------F--RRW

MD----EHLNVK-------------IPRL--------VDVIC---ASPG----DQ---RG

KSIVS-----------------LEL-T-TCVS------DV--------------------

----------TAVILFFFTFFITTMVMLAALAH----HLFYW-DVWFIYN---------V

CLA--KVKG----YRSLSTSQT----------------FYDAYISYDTKDAS--VTDW-V

INEL--RYHLE-----ESRDK----NV----LLCLE-ERDWDPGLA-IIDNLMQ-SINQS

KKTVFVLTKK---YAKS-WNFKTAFYLALQ--RLMDENM---DV----IIFILLEPVL-Q

HS---QYLRLRQRICKSSILQWP-----DNPK--AEGLFWQTLRNVVL------------

---------------------TE-------------------------------N-DSRY

NNMYVDSIKQY-------------------------------------------------

------------------------------------------------------

>TLR8_Mumu

------------------------------------------------------------

-------------------------MENMPPQSWILTCF---------------------

---------CLLSSGTSAIFHKA-------------------------------------

------------------------------------NYSRSYPCDEIRHNSLVIAECN-H

R----------QLHEVPQTIGK-----------YVTNIDLSD------------------

------------------NAITHITKE-SFQKL-Q----NLTKIDLNHNAK-----QQHP

NENKNGMNITEGALLS----LRNLTVLLL---EDNQLYT-------------IPAGL---

----------PESLKELSLIQ--------------------------NNIF---------

--------QVTKNNTFG-LRNLER---------------------LYLGWNCYFKCNQTF

KV-------------------------------------------------EDGAFKNLI

H--------------LKVLSLSFNNL-----FYV--PPKLPSSLRKLFLSNAKIMNIT--

-QEDFKGLENLTLLDLSGNCPRCYNAPFPCTPC--KENSSIHIHPLAFQSLTQLLYLNLS

STSLRTIP-STWFENLSNLKELHLEFN-------------------------YLVQEIAS

GAFLTKLPSLQILDL----SFNFQYKEYL-QFINISSNFSKLRSLKKLHLRGYVFRELKK

KHFEHLQSLP--NLAT-----------INLGINFIEK-----------------------

---IDFKAFQNFSKLDVIYLSGNRIASVL---------------------------DGTD

YSSWRNRLRKPLSTDDDEFDPHVNF------------YHSTKPLIKPQCTAYGKAL----

----------DLSLN---------------------------------------------

--NIFI-----IGKSQFE--GF---Q------DIACLNLSFNANT----QVFNGTE----

----FSSMPH-----------------------------IKYLD---------------L

TNNRL-DFDDNN----------------------------------AFSDLH-DL-----

---EVLDLSHN-----AHYFSIAGVTH------------RL----------GFIQNLINL

--------------RVLNL-SHNGI---------------------------------YT

LTEESELKSISLKELVFS------------------------------------------

-----GNRLDRLWNANDGKYWSIFKSLQNL-IR----LDLSYN----------NLQQIP-

-----------------------NGAFLN-------------------------------

------------------------------------------------------------

------------------------------------------------------------

-------------------------LPQSL---QELLIS---GNKLR-------------

----FF------------------NWTL----LQYFPHLHL-LDLSR----NELY---FL

P---NCLSKFAHSLETLLLSHN--HFSHLPSGF-LSE----ARNLVHLDLSFN-TIKMIN

K-SSLQTK-----M-----------------------KTNLS-ILELHG----NYFDCT-

--CDISD-----------------------------------------------F--RSW

LD----ENLNIT-------------IPKL--------VNVIC---SNPG----DQ---KS

KSIMS-----------------LDL-T-TCVS------DT--------------------

---------TAAVLFFLTFLTTSMVMLAALV--H---HLFYW-DVWFIYH---------M

CSA--KLKG----YRTSSTSQT----------------FYDAYISYDTKDAS--VTDW-V

INEL--RYHLE-----ESEDK----SV----LLCLE-ERDWDPGLP-IIDNLMQ-SINQS

KKTIFVLTKK---YAKS-WNFKTAFYLALQ--RLMDENM---DV----IIFILLEPVL-Q

YS---QYLRLRQRICKSSILQWP-----NNPK--AENLFWQSLKNVVL------------

----------------------T---------------------EN--------D--SRY

DDLYIDSIRQY-------------------------------------------------

------------------------------------------------------

>Pema_putative_TLR7_8.1

------------------------------------------------------------

------------------------------------------------------------

------------------------------------------------------------

------------------------------------MYHQNLPSTHCNHKFTIEEDAFVD

L-------------------------------LNLTSLSLNI------------------

------------------NSLILIPKK-----LPS----SLKSLWLEFNHI---------

------TTLSSGDFSG----LHTLQNLHL---CCNCYLS-----NACYKSFNISQDV---

--------FTLPNLTFIDLSN--------------------------NNLT---DVK---

--------LNL-------STTIRN---------------------LVIGNND-IRKLED-

------------------------------------------------------YFHGMM

H--------------LESLDVALNC-----------PRCITASFPCKQCPNNEPLDID--

--------------------------------------------TFIFSDMPLLTHIDLA

STSLKKIP-PQLFENNTHMTYLDLSYN-------------------------YLGGEIEN

GIFLNYLQRIEHLNL----SFNYYFEKYP-VELKLSSNFANLTYLKGLIFQYLHINN--L

NPLKTLLNLTS----------------INVSLNFIKQ-----------------------

---VNIMAFKGLSQLNEIIISENLISPE----------------------------NNPG

CIIPKPNVFVNNVENNESSVLLLIS-------------KHDKKVGVPQCKMYNKVL----

----------DLGNN---------------------------------------------

--SIFF-----IQKNYFH--DM---E------NIECLVLSR--NF--INQALNGTE----

----FSNLPK-----------------------------LKYLD---------------L

SYNRISLVFNT-----------------------------------AFSELP-YL-----

---EVLDLSYN-----QYYFSLDGLVH----------------------SINFISGMSAL

--------------TELRL-VGNGI---------------------------------RS

LSTDSPLYSNTLKTLNFQ------------------------------------------

-------GNRLDILWEDGKHLNLFENFTQL-QN----LDISHN----------KIRFLL-

----------------------KNSVFVH-------------------------------

------------------------------------------------------------

------------------------------------------------------------

--------------------------LKKL---KVLNIS---HNRLT-------------

----RI------------------PWDE----LAKLEHLQQ-LDLSY----NHLT---QL

S----DVSNS-TSITKLIMHNN--NIAFISADF-VKS----MRRLTVLDIRRN-NLTAES

Q-ERFSIH----------------------------NMKHLE-GLQLSG----NPFLCT-

--CSNV--------------------------------------------W---C--LSW

IN-----STTVI-------------IPKL-------ATNVKC---KYPL---DTF---SG

KNIGV-----------------IFSLN-ESQC------EY--------------------

-------ITTGMVLFLIYNVLIISLMVTSFI--W---VRYRW-NFAHLFR----------

-----SLCRGKVNYTYENLNQN----------------AYDAFVAYDSSNVD--VCEW-V

LKEF--RVHLE------EKS-----DYPSCCHLCIE-DRDWLPGIS-ISNNLAT-SVYNS

RKTVFLLTKD---YITSGHF-RQAWSMAQQ--RMIDEKK---DV----MVFVMLERMP-S

RFLYSRYMRMRKRLCPDSFLQWP-----PNPH--AQHLFWKCLRAEIT------------

---------------------------------------------------------KSQ

TEQYWQVYEITV------------------------------------------------

------------------------------------------------------

>Pema_putative_TLR7_8.2

------------------------------------------------------------

-------------------------MPYSTFIRLHTAPF---------------------

---------FLLLHLLLLPRGTTPI-----------------------------------

------------------------------------PMQPRGGGRSWPRTLPCEVDVVKE

TAAITVDCSGLGLTHIP-----------FGIPKHVTQLNLSN------------------

------------------NKICGVQRD-DFIHFQD----TLRDLDLSGNCP-SSVTSPNC

PCDNGNFTLGAAALSS-----LNLSSLNL---GGNSFSR-------------VPSEL---

----------PRSLHTLNLQF--------------------------NRIS---------

--------QVFASDFEY-HSALRI---------------------LHLGYNCFVHNPCGR

AF-----------------------------------------------GVEPGAFSAFN

---------------LSELHLPSNNVSNP-------PTGLPASLTLLDLSGNQLRTFE--

-QKHLSGLSSLCLLDLSMNCPRCYNAPYPCHPC--PDNGPLNITHDAFFNVTELRSLNLR

SVSLRSIP-SQMFKNNPHLTHLDLSLN-------------------------YLAKALAE

GSFLLHLKRLEVLDL----SFNYEPGHYF-KELNLSSHFAALTSLKKLMIKGYVFERLED

SQIDVLHNLS--HLTS-----------LDLGINFINH-----------------------

---LDLAIFKKMSGLSSIFLSQNQISPPVEKRGEGLQ-------------------FGLG

ESQKVPGIERIILDDRTHS---------------------ITSAKYEECKSYGKLL----

----------DLSVN---------------------------------------------

--NIFF-----INPMQFN--GA---E------DVHCLNLST--NA--IGQTLNGSE----

----FVNLPN-----------------------------LKYLD---------------L

SFNRL-DLFHSS----------------------------------ALTELK-SL-----

---EVLDLSYN-----RHYFSMDGIVH------------NI----------NFISNMTLL

--------------RKLNL-GWNGI---------------------------------KS

LGPTTVIRSHTLTDLHFD------------------------------------------

-----GNNLNVLWEDGNENNYNVFENLTHI-KM----LNISYN----------NLNSIP-

-----------------------NGSVLL-------------------------------

------------------------------------------------------------

------------------------------------------------------------

-------------------------HLRNI---RELIAN---HNHLN-------------

----NF------------------PWDE----LRYMANLSV-LSLDY----NRLQ---AL

P--ISLLDFN-ISITKLSISHN--HLTTISRYF-LLH----APSLAILDVAYN-KIDGAH

R-DDFPKD---------------------------A-LDHLQ-KLMLEG----NPFLCT-

--CENI--------------------------------------------W---L--AQW

IN-----STDVN-------------IPHV-------ATGLLC---DRPD----GH---RH

KSVLV-----------------TLD---FLEC------EF--------------------

---------IGLMLGLFLLYNTIILLLMASSFVW---ARYRW-DVAYTARFYAARCCRGH

SNGGSGSNG--QDYRKLEC-------------------GYDAFVAYDSSDMD--VCEW-V

LQEM--RVHME------EAGPYGAQRTF---RLCLE-DRDWVPGMS-VADNLVA-SVHAS

RRTVFVLTRA---FAASGRF-REAFLMSHQ--RLLDEKV---DM----VILVMLERMR-S

SFTRSRYMRLRQRLSPGSILRWP-----TNPH--AQKIFWQGLRDALA------------

------------------------------------------------------G--PSK

AATQAAKRRR--------------------------------------------------

------------------------------------------------------

>TLR9_Dare

------------------------------------------------------------

-----------------------------MFGPMVSLIL---------------------

---------LLNQFQLFAAS----------------------------------------

------------------------------------HPQFYPCESHSTKDGHINVDCQHR

R-----------LSKVPRFTSP-----------SVISLNLNN------------------

------------------NHIHRIKGD-AFSGL-P----NLKYLSLMWNCI--SDRLKEA

RWPLCSVNIDPDAFVG----LKNLTSLQL---AGNSLKM-------------IPPLP---

-----------KQLEVLGLEF--------------------------NNIF---NIVK--

--------PLG-------TPQLKQ---------------------LLLSKNCFYANPCHQ

PY-----------------------------------------------FINSSVFQDLP

E--------------LLNLTLSYNNLTAI-------PSYLPGSLESLDLRENTIDHIN--

-KESFANLRNLRHLNLGWNCQRCDHASDPCFPCP-NNQSLDLHQDAFLDQRDSLVSLHLQ

GNSLRTLP-RHLFINLHKLQELDLSSN-------------------------FLAFTIQN

GTFYEELQNVVILNL----LYNYEPLKTF-PELNLSPYIEKMASLRELYLSGFFFKKLSN

RSIAPLVKLP--RLEV-----------LDLRMNFICD-----------------------

---ISIDGLSQLRTLRRVDLSQNMLAFSSCFSTCTS--------------------EAEH

QIPERYGNEQFNLQMQELP-----------------ILNAETQGSKPDYCSFYFSMWHFK

RQICSKSLYFDLSQN---------------------------------------------

--NIPW-----LNASTFR--GM---D------RVACVDLSY--NY--ISQTLNGHQ----

----FSHLSK-----------------------------LSYLN---------------M

AYNRI-DLYSDK----------------------------------AFQEVSGTL-----

---KALDLSNN-----EFHFIMKGMGH----------------------QFTFLTHMSSL

--------------IILSL-ANNHI----------------------------------G

LRISNILTSASLKYLIFS------------------------------------------

-----GNRLDILWDSWRNQYINLFQGLTNL-TH----LDISEN----------QLKSLS-

-----------------------PEVIVN-------------------------------

------------------------------------------------------------

------------------------------------------------------------

-------------------------LPLSL---QVLRVD---FNMLT-------------

----YF------------------PWAN----ISVLQKLCY-LNLSS----NMLS---YL

P--NINFE---LRLTGLDLSHN--RLVAIPKVF-LSQ----AANLKNLNLNNN-QLKILD

V-QALPLP-----F-----------HKGCTFIPGGQHKNRSSCKLVLHA----NPFTCS-

--CVISG-----------------------------------------------F--AKF

LR-----ETDLD-------------VPHL-------TTQVHC---GFPE----SL---AG

VNVLS-----------------VDL-R-SCQE------IF--------------------

----------GGVAFLCTSLLTLAATCVPLLK-----HLYGW-DLWYLIQ---------I

LWT--GHRG----HTPANGNPTDT--------------QYDAFVVFDTSNKA--VRDW-I

YKEM--LVRLE------NRGRW---RF----QLCLE-ERDWIPGVS-CIENLHK-SVYSS

RKTVFVLTSPGGYSDASGIV-RQAFLLVQQ--RLLDEKV---DV----AVLVLLDFL---

-FPKFKYLQMRKRLCKKSVLSWP-----RNPR--VQPLFWNDLRVALV------------

----------------------S---------------------DN--------V--RAY

NKNVTESFF---------------------------------------------------

------------------------------------------------------

>TLR9_Hosa

------------------------------------------------------------

--------------------------MGFCRSALHPLSL---------------------

---------LVQAIMLAMTL----------------------------------------

------------------------------------ALGTLPAFLPCELQPHGLVNCN--

---------WLFLKSVPHFSM-------AAPRGNVTSLSLSS------------------

------------------NRIHHLHDS-DFAHL-P----SLRHLNLKWNCP---PVGLSP

MHFPCHMTIEPSTFLA----VPTLEELNL---SYNNIMT-------------VPALP---

-----------KSLISLSLSH--------------------------TNIL---MLD---

--------SAS---LAG-LHALRF---------------------LFMDGNCYYKNPCRQ

AL-----------------------------------------------EVAPGALLGLG

N--------------LTHLSLKYNNL-----TVV--PRNLPSSLEYLLLSYNRIVKLA--

----PEDLANLTALRVLDVGGNCRRCDHAPNPCMECPRHFPQLHPDTFSHLSRLEGLVLK

DSSLSWLN-ASWFRGLGNLRVLDLSEN-------------------------FLYKCITK

TKAFQGLTQLRKLNL----SFNYQKRVSF-AHLSLAPSFGSLVALKELDMHGIFFRSLDE

TTLRPLARLP--MLQT-----------LRLQMNFINQ-----------------------

---AQLGIFRAFPGLRYVDLSDNRISGASELTAT----------------------MGEA

DGGEKVWLQPGDLAPAPVD-------------------TPSSEDFRPNCSTLNFTL----

----------DLSRN---------------------------------------------

--NLVT-----VQPEMFA--QL---S------HLQCLRLSH--NC--ISQAVNGSQ----

----FLPLTG-----------------------------LQVLD---------------L

SHNKL-DLYHEH----------------------------------SFTELP-RL-----

---EALDLSYN-----SQPFGMQGVGH------------NF----------SFVAHLRTL

--------------RHLSL-AHNNI----------------------------------H

SQVSQQLCSTSLRALDFS------------------------------------------

------GNALGHMWAEGDLYLHFFQGLSGL-IW----LDLSQN----------RLHTLL-

-----------------------PQTLRN-------------------------------

------------------------------------------------------------

------------------------------------------------------------

-------------------------LPKSL---QVLRLR---DNYLA-------------

----FF------------------KWWS----LHFLPKLEV-LDLAG----NQLK---AL

T--NGSLPAG-TRLRRLDVSCN--SISFVAPGF-FSK----AKELRELNLSAN-ALKTVD

H-SWFG-P-----L-----------------------ASALQ-ILDVSA----NPLHCA-

--CGAA------------------------------------------------F--MDF

LL-----EVQAA-------------VPGL-------PSRVKC---GSPG----QL---QG

LSIFA-----------------QDL-R-LCLD------EA--------------------

---------LSWDCFALSLLAVALGLGVPML--H---HLCGW-DLWYCFH---------L

CLA--WLPW--RGRQSGRDEDA-------------L--PYDAFVVFDKTQSA--VADW-V

YNEL--RGQLE------ECRG----RWAL--RLCLE-ERDWLPGKT-LFENLWA-SVYGS

RKTLFVLAHT---DRVSGLL-RASFLLAQQ--RLLEDRK---DV----VVLVILSP---D

GRRS-RYVRLRQRLCRQSVLLWP-----HQPS--GQRSFWAQLGMALT------------

---------------------------------------------------------RDN

HHFYNRNFCQGPTAE---------------------------------------------

------------------------------------------------------

>TLR9_Mumu

------------------------------------------------------------

--------------------------MVLRRRTLHPLSL---------------------

---------LVQAAVLAETL----------------------------------------

------------------------------------ALGTLPAFLPCELKPHGLVDCN--

---------WLFLKSVPRFSA-------AASCSNITRLSLIS------------------

------------------NRIHHLHNS-DFVHL-S----NLRQLNLKWNCP---PTGLSP

LHFSCHMTIEPRTFLA----MRTLEELNL---SYNGITT-------------VPRLP---

-----------SSLVNLSLSH--------------------------TNIL---VLD---

--------ANS---LAG-LYSLRV---------------------LFMDGNCYYKNPCTG

AV-----------------------------------------------KVTPGALLGLS

N--------------LTHLSLKYNNL-----TKV--PRQLPPSLEYLLVSYNLIVKLG--

----PEDLANLTSLRVLDVGGNCRRCDHAPNPCIECGQKSLHLHPETFHHLSHLEGLVLK

DSSLHTLN-SSWFQGLVNLSVLDLSEN-------------------------FLYESITH

TNAFQNLTRLRKLNL----SFNYRKKVSF-ARLHLASSFKNLVSLQELNMNGIFFRLLNK

YTLRWLADLP--KLHT-----------LHLQMNFINQ-----------------------

---AQLSIFGTFRALRFVDLSDNRISGPSTLSEAT---------------------PEEA

DDAEQEELLSADPHPAPLS-------------------TPASKNFMDRCKNFKFTM----

----------DLSRN---------------------------------------------

--NLVT-----IKPEMFV--NL---S------RLQCLSLSH--NS--IAQAVNGSQ----

----FLPLTN-----------------------------LQVLD---------------L

SHNKL-DLYHWK----------------------------------SFSELP-QL-----

---QALDLSYN-----SQPFSMKGIGH------------NF----------SFVTHLSML

--------------QSLSL-AHNDI----------------------------------H

TRVSSHLNSNSVRFLDFS------------------------------------------

------GNGMGRMWDEGGLYLHFFQGLSGL-LK----LDLSQN----------NLHILR-

-----------------------PQNLDN-------------------------------

------------------------------------------------------------

------------------------------------------------------------

-------------------------LPKSL---KLLSLR---DNYLS-------------

----FF------------------NWTS----LSFLPNLEV-LDLAG----NQLK---AL

T--NGTLPNG-TLLQKLDVSSN--SIVSVVPAF-FAL----AVELKEVNLSHN-ILKTVD

R-SWFG-P-----I-----------------------VMNLT-VLDVRS----NPLHCA-

--CGAA------------------------------------------------F--VDL

LL-----EVQTK-------------VPGL-------ANGVKC---GSPG----QL---QG

RSIFA-----------------QDL-R-LCLD------EV--------------------

---------LSWDCFGLSLLAVAVGMVVPIL--H---HLCGW-DVWYCFH---------L

CLA--WLPL--LARSRRSAQTL----------------PYDAFVVFDKAQSA--VADW-V

YNEL--RVRLE------ERRGRR--AL----RLCLE-DRDWLPGQT-LFENLWA-SIYGS

RKTLFVLAHT---DRVSGLL-RTSFLLAQQ--RLLEDRK---DV----VVLVILRPDA-H

RS---RYVRLRQRLCRQSVLFWP-----QQPN--GQGGFWAQLSTALT------------

----------------------R---------------------DN--------R--HFY

NQNFCRGPTAE-------------------------------------------------

------------------------------------------------------

>TLR5a_Dare

------------------------------------------------------------

------------------------------------------------------------

-------MAATYTLFLMLLF----------------------------------------

------------------------------------IWTPTVKSTSVCSTDGYFAFCM-D

R--GL--------------QEV------PKISTYITNVDLSK-N----------RIA---

------------------E----LNET-SFSHL-E----ALEVLILMHQTP---------

-----GLVIRRNSFMR----LSNLTSLQL---DYNHHLR-------------IDAGA---

-------FNGLSDLKNLTLTQ--------------------------CGLD---DSL---

--------LSG-NFLKP-LMSLEM---------------------LDLSRNN-IRRVQPA

SF-----------------------------------------------------FLNMR

R--------------FHVLDLTFNKV------KS--I--CEEDLLNFQGKHFTLLRLS--

------------------------------------------------------------

------------SITLQDMNEYWLGWE---------------------------------

-KCGNPFKNTSITTL----DLSGNGFNV-------------------------------D

MAKRFFDAIS--STKIQS---------LIISNTYRIG-----------------------

-----------------------------------------KSSGDNSKDPD----KSTF

SGLKSSGIKSFDLSNSSIF----SL-------------TYSVF----SYLSDLEQI----

----------TLAES---------------------------------------------

--QINK-----IENNAFL--GM---T------NLLKLNLSK--NF---LGYIDFRT----

----FQNLKG-----------------------------LEVLD---------------L

SYNHI-WRLGSQ----------------------------------SFQGLP-NL-----

---LSLNLTGN---------SLGHVYT------------FAT--------------LPKL

--------------EKLYLGDNNIQ-------------------------------YVYE

IPNIA----KHLKTLDLQ------------------------------------------

----------FNQIASMSELYTILDEFPQI-EE----VVFRGN----------QLLYCP-

-----------------------QDNHEV-------------------------------

------------------------------------------------------------

------------------------------------------------------------

-------------------------LSQNI---QVLDLS---FAGLQ-------------

----VI----WSEG---------KCLNV----FDDLHQLEV-LHLSS----NLLQ---SL

P--KDIFKDL-TSLLILDLSFN--SLKYLPTDV-FPK------TLQVLHLDYN-SIYSVD

P-NLFS---------------------------------TLS-YLSLMN----NDFRCD-

--CDLKD-----------------------------------------------F--QTW

LN--------QT-------------NITF----VHSIEDVTC---ASPE----DQ---YM

VPVVS---------------------S-SIQC------ED--------------------

EEEERRTEKLRLVLFISCTVLIILFSASTIV------YISRR-GVIFKIY----------

-----KKLI--GKFDNKPQEEP-DA-----NG---F--LYDVYLCFSS---R--DMKW-V

ERAL--LKRLD---SQFSEHN----TL----RCCFE-ERDFIPGED-HLTNMRN-AIQHS

RKTICVVSKH---FLKDGWC-LETFTLAQC--RMLVELK---DI----LVVLVVGNIP-Q

YRLL-KYEQLRSFIENRSYLVWP-----DDGQ--DLEWFYDQLLHKII------------

----------------------R---------------------NT--------K--VKQ

TNIKEKDKVDENNPEAADVQADTAV-----------------------------------

------------------------------------------------------

>TLR5b_Dare

------------------------------------------------------------

------------------------------MIRAHRMGY---------------------

---------TFILILFGLCL----------------------------------------

------------------------------------NTEVVKSTSECSVIGYN-AICI-N

R--GL--------------HQV------PELPAHVNYVDLSL-N----------SIA---

------------------E----LNET-SFSRL-Q----DLQFLKVEQQTP---------

-----GLVIRNNTFRG----LSSLIILKL---DYNQFLQ-------------LETGA---

-------FNGLANLEVLTLTQ--------------------------CNLD---GAV---

--------LSG-NFFKP-LTSLEM---------------------LVLRDNN-IKKIQPA

SF-----------------------------------------------------FLNMR

R--------------FHVLDLTFNKV------KS--I--CEEDLLNFQGKHFTLLRLS--

------------------------------------------------------------

------------SITLQDMNEYWLG-----------------------------------

---------WEKCGN----PFKNTS-------------------ITTLDLSGNGF-KE-S

MAKRFFDAIA----GTKI--QS-----LILSNSYNMG-----------------------

----------------------------------------SSFGHTNFKDPD----NFTF

KGLEASGVKTCDLSKSKIF----AL-------------LKSVF----SHFTDLEQL----

----------TLAQN---------------------------------------------

--EINK-----IDDNAFW--GL---T------HLLKLNLSQ--NF---LGSIDSRM----

----FENLDK-----------------------------LEVLD---------------L

SYNHI-RALGDQ----------------------------------SFLGLP-NL-----

---RKLNLTGN---------AVESVHT------------F-------------AA-LPNL

--------------NKLYL-GKNRIS------------------------------SVSS

LPNIA----HNLSTLDLE------------------------------------------

----------FNKLHALSDLYTILREFPQI-EN----IFLQGN----------TFSSCY-

-----------------------NQKQIV-------------------------------

------------------------------------------------------------

------------------------------------------------------------

-------------------------LSDKL---QLLHLG---LSSMQ-------------

----LI----WSEG---------KCLNV----FADLHQLQQ-LSLTA----NGLQ---SL

P--KDIFKDL-TSLFFLDLSFN--SLKYLPTDV-FPK------SLQILNLDYN-SIYSVD

P-NLFS---------------------------------TLS-YLSLMN----NDFRCD-

--CDLKD-----------------------------------------------F--QTW

LN--------QT-------------NVTF----VHPIEDVTC---ASPE----DQ---YM

VPVVK---------------------S-SIQC------EN--------------------

EEEERRTEKLRLVLFIVCTVLIILFTASTIV------YISRR-GVIFKMY----------

-----KKLI--GELVDGKREEP-DP-----DR---F--LYDVYLCFSS---K--DMKW-V

ERAL--LKRLD---SQFSEHN----TL----RCCFE-ERDFIPGED-HLTNMRS-AIQNS

RKTICVVSEH---FLKDGWC-LETFTLAQK--RMQAELE---DI----LVVLVVGNIP-Q

YRLL-KYKQVRSFIENRSYLVWP-----DDCQ--DLEWFYDQLLHKIR------------

----------------------K--------------------DIK--------I--NQT

TKEIKREEANFNTNTAV-------------------------------------------

------------------------------------------------------

>TLR5_Xetr

------------------------------------------------------------

-------------------------MPGMDSCNYRVTLF---------------------

---------YKLIAIIGGSA----------------------------------------

------------------------------------LAFGQIPKCTTTNRIAYFFYCN-L

T-------------------HM------PPVNSDTLVLDLSF-N----------YLS---

------------------E----VNST-FFPRL-L----QLVDLNLGSQQT---------

----ERLIVKKDSFRN----TPSLVKLDL---AYNQLLI-------------LDSDG---

-------LAGLSQLKILFLYY--------------------------NKLN---GSI---

--------LEN-DYFKD-LMSLEY---------------------VDLSSNE-ITYLRPN

PL-----------------------------------------------------FYHLY

S--------------LDIINLRHNQI------SS--I--CAGDLHSFERKYFTIMELS--

---------------------------------------------------------HNR

FNKWEMLGSDRCGNPFRNIEFDTLM-----------------------------------

---LSGTGFDVDKMQ----KFCGALNGTK------MMQLKLDSHGMGAGFGYNHI-K--D

PDNSTFVGLV--NSNLQI---------LDLSTGNIFS-----------------------

------------------------------------------------------------

-------------------------------------LKPYTF----GNLTVLKLL----

----------NLAEN---------------------------------------------

--KINR-----IENKAFC--GL---N------SLITLNLSY--NL---LGELYDYS----

----FEGLPD-----------------------------LTTID---------------L

QQNHI-GAIQNN----------------------------------AFKSLE-KL-----

---NYLDLRGN---------AMKKVTF------------FEA--------------LIPI

--------------TYIFF-GGNKLKSID---------------------------SSYV

YSTFLDLSENDLNDLGGL------------------------------------------

---------------------FKILQYPLL-QY----VILKRN----------RLSVCY-

-----------------------SHFSIS-------------------------------

------------------------------------------------------------

------------------------------------------------------------

-------------------------KNNSL---LHLDLS---DNMIN-------------

----LI-------------WDNGQCLNV----FSNLSLLGL-LKLNN----NFLR---YL

P--DGIFNGL-ESLQTLNLSSN--LLTHLMPGV-FPT------NLDTVDLSKN-QLYSPS

P-KVFL---------------------------------SVK-KLDLTH----NQYICD-

--CFLLD-----------------------------------------------F--IMW

LN----ETNAT--------------LLGS-------PYDTYC---MYPP----NF---LF

QPLHI-----------------LTV-E-NCDD------DK--------------------

-----VLMPLMFSLFVLTSTTILVLMSTVVT--Y---THYRG-FCFATYK----------

-----RILG--LIIGNEKKEEA-ADA---------C--KYDAYLCYSS---K--DFQW-V

QDAF--LRNLD---TQYCDRN----RF----RLCFE-ERDFIPGED-HIVNIRD-AIWNS

KKTICIVTKQ---FLKDGWC-VEALNYAQS--RYFTELK---DV----LIMVVVGSLS-Q

YQFM-KYQPIRAYVKRAQYLRWP-----EDIQ--DVEWFLGRLSYQIL------------

------------------------------------------------------K-ENTV

EKKPNQASRKPFNHELQNIETIS-------------------------------------

------------------------------------------------------

>TLR5_Hosa

------------------------------------------------------------

------------------------------------------------------------

-----MGDHLDLLLGVVLMA----------------------------------------

------------------------------------GPVFGIPSCSFDGRIAFYRFCN-L

T-------------------QV------PQVLNTTERLLLSF-N----------YIR---

------------------T----VTAS-SFPFL-E----QLQLLELGSQYT---------

-----PLTIDKEAFRN----LPNLRILDL---GSSKIYF-------------LHPDA---

-------FQGLFHLFELRLYF--------------------------CGLS---DAV---

--------LKD-GYFRN-LKALTR---------------------LDLSKNQ-IRSLYLH

P-----------------------------------------------------SFGKLN

S--------------LKSIDFSSNQI------FL--V--CEHELEPLQGKTLSFFSLA--

-----------------------------------------------------------A

NSLYSRVS-VDWGKCMNPFRNMVLE-----------------------------------

ILDVSGNGWTVDITG----NFSNAISKSQ------AFSLILAHHIMGAGFGFHNI-K--D

PDQNTFAGLA--RSSVRH---------LDLSHGFVFS-----------------------

------------------------------------------------------------

-------------------------------------LNSRVF----ETLKDLKVL----

----------NLAYN---------------------------------------------

--KINK-----IADEAFY--GL---D------NLQVLNLSY--NL---LGELYSSN----

----FYGLPK-----------------------------VAYID---------------L

QKNHI-AIIQDQ----------------------------------TFKFLE-KL-----

---QTLDLRDN---------ALTTIHF-----------------------------IPSI

--------------PDIFL-SGNKL-----------------------------------

---------VTLPKINLT------------------------------------------

----ANLIHLSENRLENLDILYFLLRVPHL-QI----LILNQN----------RFSSCS-

-----------------------GDQTPS-------------------------------

------------------------------------------------------------

------------------------------------------------------------

-------------------------ENPSL---EQLFLG---ENMLQ-------------

----LA----WETE---------LCWDV----FEGLSHLQV-LYLNH----NYLN---SL

P--PGVFSHL-TALRGLSLNSN--RLTVLSHND-LPA------NLEILDISRN-QLLAPN

P-DVFV---------------------------------SLS-VLDITH----NKFICE-

--CELST-----------------------------------------------F--INW

LN--------HT-------------NVTI----AGPPADIYC---VYPD----SF---SG

VSLFS-----------------LST-E-GCDE------EE--------------------

-----VLKSLKFSLFIVCTVTLTLFLMTILT--V---TKFRG-FCFICYK----------

-----TAQR--LVFKDHPQGTE-PDM---------Y--KYDAYLCFSS---K--DFTW-V

QNAL--LKHLD---TQYSDQN----RF----NLCFE-ERDFVPGEN-RIANIQD-AIWNS

RKIVCLVSRH---FLRDGWC-LEAFSYAQG--RCLSDLN---SA----LIMVVVGSLS-Q

YQLM-KHQSIRGFVQKQQYLRWP-----EDLQ--DVGWFLHKLSQQIL------------

----------------------------------------------------KKEKEKKK

DNNIPLQTVATIS-----------------------------------------------

------------------------------------------------------

>TLR5_Mumu

------------------------------------------------------------

---------------------MDAEFPHAPHFSRIMACQ---------------------

---------LDLLIGVIFMA----------------------------------------

------------------------------------SPVLVISPCSSDGRIAFFRGCN--

------------LTQIPWILNT-----------TTERLLLSF------------------

------------------NYISMVVAT-SFPLL-E----RLQLLELGTQYA---------

-----NLTIGPGAFRN----LPNLRILDL---GQSQIEV-------------LNRDA---

-------FQGLPHLLELRLFS--------------------------CGLS---SAV---

--------LSD-GYFRN-LYSLAR---------------------LDLSGNQ-IHSLRLH

S-----------------------------------------------------SFRELN

S--------------LSDVNFAFNQI----------FTICEDELEPLQGKTLSFFGLK--

------------------------------------------------------------

LTKLFSRVSVGWETCRNPFRGVRLE-----------------------------------

TLDLSENGWTVDITR----NFSNIIQGSQ---------ISSLILKHHIMGPGFGFQNIRD

PDQSTFASLA--RSSVLQ---------LDLSHGFIFS-----------------------

------------------------------------------------------------

-------------------------------------LNPRLF----GTLKDLKML----

----------NLAFN---------------------------------------------

--KINK-----IGENAFY--GL---D------SLQVLNLSY--NL---LGELYNSN----

----FYGLPR-----------------------------VAYVD---------------L

QRNHI-GIIQDQ----------------------------------TFRLLK-TL-----

---QTLDLRDN---------ALKAIGF-----------------------------IPSI

--------------QMVLL-GGNKL-----------------------------------

---------VHLPHIHFT------------------------------------------

----ANFLELSENRLENLSDLYFLLRVPQL-QF----LILNQN----------RLSSCK-

-----------------------AAHTPS-------------------------------

------------------------------------------------------------

------------------------------------------------------------

-------------------------ENPSL---EQLFLT---ENMLQ-------------

----LA-------------WETGLCWDV----FQGLSRLQI-LYLSN----NYLN---FL

P--PGIFNDL-VALRMLSLSAN--KLTVLSPGS-LPA------NLEILDISRN-QLFSPD

P-ALFS---------------------------------SLR-VLDITH----NEFVCN-

--CELST-----------------------------------------------F--ISW

LN-----QTNVT-------------LFGS-------PADVYC---MYPN----SL---LG

GSLYN-----------------IST-E-DCDE------EE--------------------

-----AMRSLKFSLFILCTVTLTLFLVITLVVIK---FR----GICFLCY-------KTI

QKL--VFKD--KVWSLEPGA---------------Y--RYDAYFCFSS---K--DFEW-A

QNAL--LKHLD---AHYSSRN----RL----RLCFE-ERDFIPGEN-HISNIQA-AVWGS

RKTVCLVSRH---FLKDGWC-LEAFRYAQS--RSLSDLK---SI----LIVVVVGSLS-Q

YQLM-RHETIRGFLQKQQYLRWP-----EDLQ--DVGWFLDKLSGCIL------------

---------------------KE--------------------EKG--------K--KRS

SSIQLRTIATIS------------------------------------------------

------------------------------------------------------

>TLR5_Chpi

------------------------------------------------------------

---------------------MQSCLSSWLFNYHLQHLP---------------------

---------VYSKSNITMLH----------------------------------------

------------------------------------HLVFLLGMSLVTKEIFAFTSCY-S

D-GQIARYYFCNLTEVP---PV------PN---NTVILWLNF-N----------KIR---

------------------Q----VNAS-SFPLL-E----QLQILEIGTQLV---------

----SSVTIGKAAFRN----LPNLRNLDL---GDNKILH-------------LDPDA---

-------FVKLSNVQILRLYH--------------------------NSLE---ESI---

--------LEE-DYLRD-MISLEY---------------------LDLSGNK-IKSLRPH

RL-----------------------------------------------------FYRLK

S--------------LQIVDLKNNRI------PI--L--CEGNLDSFQGKFFTLFILS--

-----------------------------------------------------------L

NKLYYPIS-MDWAKCGNPFKNIALD-----------------------------------

TLDLGGNGWGVDIIQ----HFCTAVNGTS------IVFLKLSHHIMGPGFGFKNL-K--D

PDQDTFAGLA--RSGVRL---------LDISHGFIFS-----------------------

------------------------------------------------------------

-------------------------------------LNPYVF----QSLGDLELL----

----------NLHNN---------------------------------------------

--KINQ-----IQKQAFF--GL---G------NLGTLNLSY--NI---LGELYDYT----

----FEGLQN-----------------------------VMHID---------------L

QQNHI-GVIAGN----------------------------------SFRDLR-RL-----

---KLVDLRDN---------AIKTLPS-----------------------------FPAM

--------------ITLHL-SDNKL-------------------------------LSVR

NQSIN----ATILVLERN------------------------------------------

-------------RLDNLGDLYILLQVPDV-KY----ILLRQN----------RLSYCV-

---------------------KSVDVIEN-------------------------------

------------------------------------------------------------

------------------------------------------------------------

---------------------------NQL---VYLDLG---ENMLK-------------

----LV----WDRG---------LCLDV----FRALSKLEV-LHLNN----NYLT---TL

P--QDIFSGL-TSLNRLNLASN--LLSYLSPGV-FPE------SLKTLNMSEN-QLLSPA

L-ELFM---------------------------------TLS-ILDITN----NRFFCD-

--CS--------------------------------------------------L--NTW

TA----RLNQTN-------------VTLA-----GSENDTYC---ILPP----FL---TR

VPLSS-----------------VAL-D-GCNE------DE--------------------

-----LQKPLQFSLFIFTSVTLIMFLTAVII--F---SHFRG-TCFVWYK----------

-----TIKG--AMLKERKQAID-T------SA---Y--QYDAYLCYSN---R--DFEW-V

QNSL--IKHLD---SQYSEKN----RF----TLCFE-ERDFLPGEE-QITNIRD-AIWNS

RKTICIVTRQ---FLKDGWC-VEAFNFAQS--RFFCDLQ---DV----LIMVVVGSLS-Q

YQLM-KYKPIRVFVQRSQYMQWP-----EDHQ--DVDWFLNNLSHQIL------------

---------------------------------------------------------KEK

KVKKKSSVIEMQTVRTIS------------------------------------------

------------------------------------------------------

>TLR5_Gaga

------------------------------------------------------------

------------------------------------------------------------

---------MMLHQRLIIVFGIALA-----------------------------------

------------------------------------GDICASRSCYSEDQVSMYNSCNLT

G--------------------------VPPVPKDTAKLFLTY------------------

------------------NYIRQVTAT-SFPLL-E----DLFLLEIGTQRV---------

----FPLYIGKEAFRN----LPNLRVLDL---GFNNILL-------------LDLDS---

-------FAGLQRLTILRLFQ--------------------------NNLGDS-------

--------ILEERYFQD-LRSLEE---------------------LDLSGNQ-ITKLHPH

P-----------------------------------------------------LFYNLT

I--------------LKAVNLKFNKI----------SNLCESNLTSFQGKHFSFFSLS--

---------------------------------------------------------TNT

LYRTDKMIWAKCPNPFRNITFNSLDVS------------------------ENGWSTETV

QYFCTAIKGTQINYL----SFRSHTMG-----------------------SGFGFNNLKN

PDTDTFTGLA--RSDLHL---------LDISNGFIFS-----------------------

------------------------------------------------------------

-------------------------------------LNSLIF----ESLRNLEFL----

----------NLFRN---------------------------------------------

--KINQ-----IQKQAFF--GL---E------NLEILNLSS--NL---LGELYDYT----

----FEGLHS-----------------------------IMYID---------------L

QQNHI-GMIGEK----------------------------------SFSNLV-NL-----

---KIIDLRDN---------AIKKLPS-----------------------------FPHL

--------------TSAFL-SDNKL-----------------------------------

--MSVAHTAIVATHIELE------------------------------------------

-----------RNWLANLGDLYVLFQVPGV-QY----LLLKQN----------RFSYCV-

---------------------KHVDAIEN-------------------------------

------------------------------------------------------------

------------------------------------------------------------

---------------------------NQL---IYMDLG---ENMLQ-------------

----LV-------------WERGLCLDV----FRTLSKLQV-LHLNN----NYLS---AL

P--QEIFNGL-TSLKRLNLASN--LLSHLSLRV-FPQ------SLINLNLSGN-QLFSPK

P-EVFM---------------------------------TLS-ILDITH----NKYVCD-

--CALKS-----------------------------------------------L--LVW

LN-----ETNVT-------------LAGS-------ESDRYC---VYPP----AL---AG

VPVSF-----------------LTY-D-DCDE------DE--------------------

-----LQQTLRFSVFVFLSVTLLMFLMSTII------FTRCR-GICFVWY----------

KTITKTLIG---SHPPAADTSE-------------Y--MYDAYLCYSK---N--DFEW-V

QNSL--LKHLD------SQYFDKN-RF----TLCFE-ERDFLPGEE-HINNIRD-AIWKS

RKTICVVTRQ---FLKDGWC-VEAFNFAQS--RYFSDLK---EV----LIMVVVGSLS-Q

YQLM-KHKPIRIFLQRSRYLRWP-----EDYQ--DIGWFLDNLSSQIL------------

--------------------------------------------KE--------K--KVQ

RNVSGIELQTIATVSH--------------------------------------------

------------------------------------------------------

>Crgi_TLR_2

------------------------------------------------------------

---------------------------------MKMHTT---------------------

---------PVLLVGICLLQAGV-------------------------------------

------------------------------------AWADTCPAYCFCNKLLTSVNCEGK

R-----------LMKIP-----------TDLPKTVEKLYLQH------------------

------------------NEIADLEPN-SLCGL-S----ELQELYLQNNKL---------

------SFIKSLTFTG--TCVPNLKVIRL---DNNRISS-------------LEENA---

-------FFNMTNLNITYFTN--------------------------NVIT---HIN---

--------PRS---FVE-CFKMSF---------------------LHLGQNY-LDHIPAI

SF--------------------------------------------------------LT

G--------------LQQLSIQGNKV-KNATFPT--SYENCTLLSTIGLSTNFIENLT--

------------------------------------------------------------

---------KETFQSLQNCPVRKLELS---------------------------------

RNKITDISKEAFLPLTKLVSLTISQNHLTAPKLKIGLEGLKSSSLSSLNIARLQLGG--Q

LPSSTFALLN--GTVLKQ---------LLMSNNKINQ-----------------------

------------------------------------------------------------

-------------------------------------LPSRAF----ATLKRLEQV----

----------DLKGC---------------------------------------------

--KIQT-----IANDTFA--GL---H------FLTNLNLAD--NY---LDKVPTNL----

----PSS--------------------------------LNILY---------------L

NGNQI-IALGEN----------------------------------SFVNLV-SL-----

---KNLYLGAN---------KISEVNK------------L-----------AFNG-LVSL

--------------QKLHL-VSNSI---------------------------------SS

LAAELFAPFGRLISLELN------------------------------------------

----------KNNLKTVQNSPDIFSSMTSL-LY----LSLADN----------GCSIMP-

-----------------------LSSFKH-------------------------------

------------------------------------------------------------

------------------------------------------------------------

--------------------------LQSL---KYLLLD---GNNLG-------------

----DL-------------IGSDNSGTL----FAGLHKLET-LSLSK----NFLH---NL

P--TSMFRDL-SSLKTLTMQGN--RISGWNNGL-FKQ----TSALKTLDLSDN-SISLVN

S-TSLE-D-----L---------------------SQNQNFQ-MLNLSN----NPFACT-

--CDLR--------------------------------------------W---F--RDW

VN-----QTKVN-------------VANV--------ENYVC---NSPD----AW---KG

KPFLS-----------------FDR-T-KINC---VWFNL--------------------

----------YFVIGVSVASSVAMLIFCVFI------YRKRW-WILYRCY-----RLKNS

CMA--PRSR--AGYQPINSEHG-------------W--KYDAYLSYAD---E--DYQW-I

LENL--LPGID------SGELSPEETFNGEFRLYFN-DRDSVPGSS-MISNISD-NIEMS

RKVIIVLTKK---YLSSAQH-TFEIDLAVM--LKLKDII---DD----IIVINVLGVP-Y

QSIP---KSLQRKVSRDEFLLWE-----DEVN--ARRVFKQRLIEALT------------

--------------------------------------------------------SKRK

TREVIV------------------------------------------------------

------------------------------------------------------

>Crgi_TLR_3

------------------------------------------------------------

---------------------------------MTLAML---------------------

---------LSLLLLLVDVA----------------------------------------

------------------------------------WTNTCPAKCFCNRVLTS-VNCE-G

K----------RLMEIP-----------AGLPKSVQKLYLRF------------------

------------------NEIADLEPN-SLCGL-P----ELQELYLQNNKL---------

------SILKSWTFNG--TCLPNLKVIRL---DNNRISS-------------LEENA---

-------FFNLTSLNLTYFTN--------------------------NYIT---FIH---

--------PRS---FVE-CLKMSF---------------------LHLGQNY-LDHIP--

------------------------------------------------------AISLLP

G--------------LQQFSIQGNKV-KNATFPD--TYENSKELATIGLSSNMIENLT--

------------------------------------------------------------

---------KESFQNLQNCPVRKLELS-------------------------RNKITDIS

KEAFLPLTKLVSLTI----SSNFLTASKL------------------------------Q

IGLEGLKSSS--LSSLNIAR-------LQLGGQLPSS-----------------------

----------------------------------------------------------TF

ALLNGTVLKQLLMSDNKIN----QL-------------PSRAF----ASLRRLEQI----

----------DLKGC---------------------------------------------

--KIQT-----IANDTFA--GL---D------TLTNLNLEN--NF---LNKVPTNL----

----PST--------------------------------LNILY---------------L

NRNQI-VALGEN----------------------------------AFVNLV-SL-----

---KNLYLDSN---------KISEVNK------------L-----------AFNG-LVRL

--------------QKLHL-VSNSI---------------------------------SS

LAAELFAPFGQLISLDLS------------------------------------------

----------NNNLKAIQNSPDIFSSMTSL-TS----LSLAEN----------GCSSLP-

-----------------------LQSFNH-------------------------------

------------------------------------------------------------

------------------------------------------------------------

--------------------------LQSL---KHLKFD---DNNLG-------------

----GL-------------IGSDNIGTL----FAGLHKLET-LSLSK----NFLH---NL

P--ISIFKDL-SSLQTLTMKSN--RISGWNNGL-FKQ----TSALRSLDLSDN-SISLVN

S-SSLA-D-----L---------------------SQNSNFQ-MLNLSN----NPLACT-

--CDLR--------------------------------------------W---F--RDW

VN----QTRVNI-------------ANVG---------NYVC---NSPN----VW---KG

KPFLS-----------------FDR-T-KINC---VWFNL--------------------

----------YFVVGVSIASGLAVLVFCVII------YKKRW-WILYRCY---------R

LKNCCVVSE--ARYQPINYQDG-------------QELVFDAYISYAD---D--DYKW-V

LEQL--LPDID------SGELSPGEPFKGEFKLYFH-DRDSVPGSS-MISSISD-NIEMS

RKVIIVLTEK---YLSSARH-KFEIDLAVM--LKSQGVI---DD----IIVINVCGVS-F

ACIP---KSLQRKVSKDEFLLWK-----DDVD--AIWLFKQRLKAELK------------

----------------------------------------------------------RM

KDVTEVIA----------------------------------------------------

------------------------------------------------------

>Ptfl_TLR_16

------------------------------------------------------------

------------------------------------------------------------

---------MNTLYLTAQ------------------------------------------

------------------------------------SLNKRTTRCHQCRPDTNTTSCR-L

Q-YPP--------LTTIP-QGI------NP---KTAVLNLGT-N----------QIS---

------------------I----LKSN-QFTSL-P----HLRKLNIENNAM---------

------EEIQDFAFTN----LKKLQVLNL---QNNKISN-------------LTRYT---

-------FSGLESLELLNLHG--------------------------SNIR---ILA---

--------NNS---FQP-LGKLKT---------------------LHLSHCT-IKSIEPD

------------------------------------------------------AFHGLS

E--------------LQQLYLEDNKL-TIF--PS--Y--CLSHTPSLLNLNLNMNFLG--

-----------------------------------------DVPQDICGYTPMLKHVDIS

NNPVKILNFGNSFQNCTHFTLLTAQ--------------------------NQLSHIDEN

SFKSFSNHVMDSLEI----SFGEISGQPF-KLFEQIRELKIQNLLSLYEKSHTSL-S--I

AMENVLHAFE--GVKIVQIMIKT----MTTHRTVGLH-----------------------

----------------------------------------------------------NT

TFRSLADVQLEDLTIMQSP----VT------------IDDNTF----QWFPQLNRL----

----------TLSKT---------------------------------------------

--SLTF-----IPDNAFR--GL---S------YLIQLDLSA--NK---LLTIPTPA----

----LLAFSN-----------------------------LQILD---------------L

SLNSI-PSISVG----------------------------------MLSTVD-TL-----

---RAFHLKSN---------RVDMYNL------------E-------------MAVLSNQ

--------------SRLERLELSNF-------------------------------WTSF

PNVSSTKVLNNVTNIDFG------------------------------------------

-----STYNLSYRLYRYLIRCYLAVIFSNL-SH----LDISGA----------GPVTLP-

----------------------KRPLDCK-------------------------------

------------------------------------------------------------

------------------------------------------------------------

--------------------------LPHL---ATLKMN---VIDLQ-------------

----SQ----------------TLWCSL----FQAFPNLKT-LELTK----NGIN---SI

S--THCFSNV-TSLTHLDLSTN--EIATLNPSI-WNG--L-N-DLITLDLRHN-AIQMVN

T-TSFA-G-----L------------------------TSLK-YLYLGE----NPFACT-

--CDTL--------------------------------------------W---F--YSW

LM--------ENPRVLIFQDIVDEEEMYW-------LQDYEC---FSPN----DH---AG

VYLVD-----------------FDI-N-ALHC------KS--------------------

----------HMPVYVITTAGILLSLIMIFAVIW---RKFKW-NIKYKFF---------L

FKLKTGYVG--QGYQVVDGTNE-ER-------------RDQIMVSYGD---D--DYHW-V

RHEM--MPRFE------ERD-----RF----CLCIK-DRDYLPGVA-IVANITE-CVHKS

DKVVIVLSES---FVDDQFC-IFELEVALQ--RLFDELR---DV----IIMVQLGPIP-E

QKLP-RLIRL--LKCRKKCLRWT-----NDEV--GQEMFWRQLKVELE------------

-------------------------------------------------------KDSRL

DHRTQLNLLNLG------------------------------------------------

------------------------------------------------------

>Ptfl_TLR_26

------------------------------------------------------------

------------------------------------------------------------

------------------------------------------------------------

------------------------------------------------------------

---------------------------------MYLSLNLNRLT----------------

-----------------------QVPQ-RLCKYTP----FLQQLSIPFNPI---------

-----ETLYFGEEFNK----CSHLTLLGM-----GHMHS---------------------

-------TINGSSFQPFNISL--------------------------NNLEVFYSYL---

--------NTSGTPYKS-FRNINY---------------------LRWQGPTPISGFDET

NA----------------------------------------------------SALLST

A--------------LQEAFYTFDDA-------------NITTFDLYELTQSNVINLH--

------------------------------------------------------------

-------------------TYFDVRW----------------------------------

-----------------------------------------------------------H

IHSTTFQGLA--KGPLQE---------LKIRRTRLTL-----------------------

------------------------------------------------------------

--------------------------------------EDFSF----KWMPQLKKL----

----------TLSDA---------------------------------------------

--SLTT-----IPNKAFY--GL---S------NLWYLDLGI--NT---LTAIPTTA----

----LSALSS-----------------------------LQFLD---------------L

SRNQL-HSLGAG----------------------------------ALRGLT-SL-----

---TNFQYSND---------HASLRHL------------YM----------SFLTDVPSL

--------------ERLKLTGCDSL----------------------------------H

TDVANVQPMKNLTIADFA------------------------------------------

-------RTRTVGLWVYRYLCYLPTTYPQL-EY----LDISGA----------SGISFP-

--------------------DKPLGCTLS-------------------------------

------------------------------------------------------------

------------------------------------------------------------

-------------------------HVNTLKMDRIIMKTYETDPNPY-------------

----LL------------------KCFL----FQSFPNLKI-LQLSK----ASLN---QI

D--SGCFSNV-SSLVYLDLSHN--QIATITPSI-WEN----LSNLVTLDLRSN-NIQTVN

S-TTFA-D-----L------------------------PALK-QLYLGG----NPFACT-

--CDIR--------------------------------------------W---F--YSW

LM----ENPGRL---LFQQRSQYSREIYW-------LQDYQC---FSPS----KY---EG

VYLVD-----------------FDI-S-TMHC------QS--------------------

----------KLPLILGTVSAVLAIVIVSSILVW---QKFKW-NIKYKLF---------L

LKLKLGVLQ--NGYQPLEGKNV-PK-------------SNQIMVSYAD---E--DYHW-V

RHKM--MTRFE------EGD-----RF----KLCIK-DRDYIPGVA-IATNIDE-CVHAS

DKVVFILSDD---FIEDQFC-IFELEMALQ--RLFDELR---DV----IILVELHPVP-E

DKLP-RLIRL--LKCRKKCFQWT-----DDTL--GQEMFWTQLKLEIE------------

----------------------K---------------------DS--------V--LDH

RVHLNLQELT--------------------------------------------------

------------------------------------------------------

>Pate_TLR_1

------------------------------------------------------------

------------------------------------------------------------

-------MGLECSFLMCFIV----------------------------------------

------------------------------------ISFCIAEGALLITDECRVYDRS-D

EFGKAAFCTQFISNLTSYSLPL-----------NTTRLQIYRTY----------------

------------------N----LSQTLPLAPNLN----MLTTLELPNDNL---------

------VNVSSGMFEG----LYNLTVLSL---ANNRILK-------------LPEGL---

-------FKNNLYISRIDLTR--------------------------NYLL---SFE---

--------ACT-VALKN-LKYLTD---------------------INLSGNKLLRNIFQT

ET----------------------------------------------------DVLGEL

T--------------IQKLDISDCAI----------EVIEIAAFENFHSLSFLDISMN--

---------------------------------------------------------PLS

EKAMKNLSQGLNLSALKEFRATDMKFT---------------------------------

------------------------------------------------------------

---------------------------AYFSNSFLNK-----------------------

------------------------------------------------------------

-----------------------------------------------LKWTNIEKL----

----------DLSDN---------------------------------------------

--KFTS-----FPRGHFP--------------HLKELIINN--CV---INVLMNGS----

----ISDMPR-----------------------------LEVLT---------------I

QKHQISHIGN------------------------------------IFHNNR-NL-----

---ISLDLSEY---------VTFTIVS------------RESATVDMTIEDYSFKYLKNL

--------------ETLIL-RKTPL--------------------------------RGV

LRRFMLFGLTNLTKLDFF------------------------------------------

------------NCAFESIQENAFETLSSL-RS----LDLSFN----------IIFELP-

-----------------------DKAFYG-------------------------------

------------------------------------------------------------

------------------------------------------------------------

--------------------------LKNL---KNLDLS---QNRLT-------------

----FL-----------------KSSEL----LNHTPKLEL-LNLNR----NKIQ---SL

S--IKMFSKL-HSIEMIQVSEN--QIQPWNKKL-FQN----NPNLTIFIFAEN-YVNYFT

R-EMLQ-D-----I------------------------SYLT-HVDFSK----NPFDCSF

--CSTVE-----------------------------------------------L--QSW

MN-----DTNVT-------------ISNL----QRELHSYDC---YSPE----SL---KE

QSLMD-----------------VNLSDLELKC----VPYI--------------------

---------FDFIMFAYSLISCCIFITFIVVAVW---YKWKW-NIRYIAF----------

-----RLRTRTKRFKENVKRF-----------------EFDAFVSYCE---K--DLPW-V

INQL--IPAIE------ENDP----NI----VLCLL-DRDLCAGNS-IFDSINS-AVEQS

RTTILVLSNA---YMNSNWN-VFETQIAQS--KLFEDMR---GG----LILIFLEPIQ-K

LEIT---KNLRYVIKTRTCLQWT-----KNAT--GQKLFWERLKMAIK------------

---------------------------------------------------------KPE

DKGISTHIT---------------------------------------------------

------------------------------------------------------

>TLR20b_Dare

------------------------------------------------------------

-----------------------MKIIVELKMLLLFGFV---------------------

---------ILFLKTSCVCS----------------------------------------

------------------------------------WLNEKCRVYSDGEEFPVKASSA-S

K-LNITVCRSVTDIKEDL-QGL------PA---NLLNLFVHMDG----------------

------------------GCHGVLAPN-SFSSF-A----SLEQLTISGCF----------

------SEIPPEAFNG----LTNVTSLTISYNFSENCSK-------------VALD----

-------FSHLPSLTSLSISD--------------------------YSLS---LLA---

--------SNV---FET-IPLLQN---------------------LILANVC-LRDMSEV

LC----------------------------------------------------RLSKVK

T--------------LKHFTLKEFLLKRLQYPNC--SVFNTSDISTEFNIEEVNLHFA--

--------------------------------------TVEHVDEGALKVFGKLSRFVFS

VSSTDFLR-DLSLIGVHKIKTLDFKVD---------------------------LLNVAD

LCTAAKLYGINSIQV----KYKTTNFWPTHTNISGGCKNIKDVMLDTILYP--ANLLDVN

CVFQIFSNLT--IISIYK---------HVLRSDDFQT-----------------------

--------------------------------------------------------LCAS

FPQTVKHLSAMDLRLKKVD----TI-------------VSHQF----MCFPNLETL----

----------IFTSS---------------------------------------------

--KIVV-----IEDFAFI--GL---N------KLKELNLRK--NK---ISSIHRHT----

----FSGLHE-----------------------------LLVLD---------------L

QENPI-IYIEPK----------------------------------SFGHFT-NL-----

---SSLLLGDL-------------------------------------------NFPPNM

--------------SLIKLHLSDIF-------------------------------GGIP

SNLSNVFISSGLRPMHLM---------------------------------------IGS

NTTLNNGLNLHIKGQYVIVEDCNSLLLTSV-VK----LQIHAA----------YMSCEN-

------------------------DFIGK-------------------------------

------------------------------------------------------------

------------------------------------------------------------

-------------------------YVPSV---VSLEFQSMFSDNIG-------------

----DL--------------------SV----INQLFHLKT-LKLRN----IEFT---NQ

PNTGIMFHNL-TKLETLILSNC--RIFFLDGSL-TKD----LKALTKLLLSAKHTVNIL-

--QSFVEH-----L------------------------VHLE-YIHIDS----IDLYCS-

--CDNA--------------------------------------------W---L--FSW

VK----DNRKVE-------------VVVS----NPSMQNLQC---FIGN----EF---DQ

LNFVS-----------------YVK-E-NCLF------DL--------------------

----------DFVLFTSTSVFLCIFIVVVLM------YNFVGQYLKPFYH----------

-----IANG--WFREALRMKEK-QQ----------Y--RYDAFVSYSG---K--DEHW-V

IEEL--LPNLE------QRGP----PFL---RLCLH-SRDFQLGHD-IVENITD-SIYAS

RRTLCLVSRN---YLNSNWC-SLEMQLATY--RLQVEHR---DI----LILVFLETIP-S

CLLS-SHHRLARLVKTRTYLDCP-----QEPE--MHDAFWDRLWCKLS------------

---------------------------------------------------------SNK

AN----------------------------------------------------------

------------------------------------------------------

>TLR20d_Dare

------------------------------------------------------------

--------------------------MAMLKRVLLFALL---------------------

---------LLFLKTYFVCS----------------------------------------

------------------------------------WLAGKCFVYGDVKEFPLTTYCP-G

N-INGADCKHVTDIKEDL-RGL------PS---SLQSVCIQMDG----------------

------------------GSDGVLAPN-SFSSF-A----SLKQLTIAGCF----------

------SQIPPEAFNG----LTNVTSLSISSSNSEKCCE-------------VALD----

-------FSRLPSLTRLFISY--------------------------YDLS---LFK---

--------LNV---FDK-IPHLEE---------------------LHLGNVC-LNNISEV

LC----------------------------------------------------RLANVK

S--------------LKQFNLDGVLH-KLQHSNC--SVFNTSNISTEFNIEKVNLTLT--

--------------------------------------KVEHVDEGALKTFGKLFFFQFF

VSHTDFLR-DLSLIGVHKIRTLDFKVD---------------------------VLNVDD

LCVAAKLYSVERMFV----SYEMINLSVTPTNMSDGCEYIMSIALSNDISVKIVDLLDVY

SLFQIFSNLT--TVTIQY---------HILRSNDFLS-----------------------

--------------------------------------------------------LCAS

FPQAVKQLSVMILKNNRID----KI-------------VSHQF----MCFVNLKTL----

----------KLVMS---------------------------------------------

--KISV-----FEDFAFI--GL---N------KLKELNLHS--NK---ISSIHRHT----

----FSGLHE-----------------------------LRVLD---------------L

QENPI-IYIEPK----------------------------------SFGHFT-NL-----

---SSLLLGDL-------------------------------------------NFPPNM

--------------SLIKLHLSDIF-------------------------------GGIP

SNLSNVFISSGLRPMHLV---------------------------------------IGS

NTTLNNGLNLHIKGQYVIVEDCNSLLLTSV-VT----LQIQAA----------YMICEN-

------------------------EFIGK-------------------------------

------------------------------------------------------------

------------------------------------------------------------

-------------------------YVPSV---VSLEFQSMFADNIG-------------

----DL--------------------SV----INQLVHLKT-LKLEN----IDLT---NQ

PNTGIMFHNL-TKLEKMILMNC--KIFFLDKSV-TKD----LKALTSLVLIPKEAVNII-

--QNFMEQ-----P------------------------THLK-YLHFQC----LDLYCS-

--CDNT--------------------------------------------W---L--VSW

IR----DNGKVE-------------VVMS----NPSMEDLRC---LTDD----EV---DH

LNFIS-----------------YAK-E-NCSI------DL--------------------

----------DFVFFSCSSVFLCIFIVVVLM------YKFVGQYFKPFYH----------

-----IASG--WFREALRMKEK-QQ----------Y--RYDAFVSYSG---K--DEHW-V

IEEL--LPNLE------QRGP----PFL---RLCLH-SRDFQLGHD-IVENITD-SIYAS

RRTLCLVSRN---YLNSNWC-SVEMQLATY--RLQVEHR---DI----LILVFLETIP-S

RLLS-SHHRLARLVKTRTYLDWP-----QEPE--MHEAFWDRLWCKLS------------

---------------------------------------------------------SNK

AK----------------------------------------------------------

------------------------------------------------------

>TLR20a_Dare

------------------------------------------------------------

-----------------------MKIIEELKMGLFFAFF---------------------

---------ILFLKTSYVCS----------------------------------------

------------------------------------WLAGKCHIESDGEEFSFSTSSP-R

K-RNTTVCRSVTDMKEDL-RGL------PA---NLQNLIVQTDL----------------

------------------GYHGVLAPN-SFLRF-G----SLENLEIVGCL----------

------SEVPPEAFNG----LTNVTTLTLSC--SQQCTE-------------VAFD----

-------FSRLTSLTSLSLSD--------------------------YSLS---LLP---

--------SNV---FEK-IPQLRW---------------------LHLCSEC-LKDLSEV

LC----------------------------------------------------RLKNVK

S--------------LKYLGLGNTMLTRLQYPNC--SVFNVSEISTKFNIERADLLLG--

--------------------------------------KLEHVDEGALKVFGKLSRLYFT

VSSKNFLR-DLSLIGVHQISVIIATVD---------------------------VLNVDD

LCVAAKLYSVKSVDV----DYDTINLSLT-SKISDGCKQIGYIMLENNIFGKTVNLLDVN

SLFQIFSNLT--SVTIDK---------HVLRSNDFQS-----------------------

--------------------------------------------------------LCAS

FPQTVKKISDMVLRIIRID----KI-------------VSHQF----MCFVNLKTL----

----------KLAMS---------------------------------------------

--KFSV-----IEDFAFI--GL---N------KLKELNLHS--NK---ISSIHQHT----

----FSGLHE-----------------------------LRVLD---------------L

NENPL-FHIEPE----------------------------------SFRHLI-NL-----

---RTLLLGDL-------------------------------------------NFPPNM

--------------SLIKLHLSDIF-------------------------------REIP

RNLSNVFISSGLRPMHLV---------------------------------------IGS

NTTLNNGLNLHIKGQYVIVEDCNSLLLTSV-VT----LQINAA----------YMICEN-

------------------------EFIGK-------------------------------

------------------------------------------------------------

------------------------------------------------------------

-------------------------YVPSV---VSLKFESMFSDNIG-------------

----DL--------------------SV----INQLVHLKT-LKLEN----IDLT---NQ

PNTGIMFHNL-TKLETLILANC--RLLFLDGSL-TKD----LKALTTLVLLPKDTVNIL-

--QTFAVH-----L------------------------IQLE-FVCFYR----LGLYCS-

--CDNA--------------------------------------------W---L--VSW

IR----DNRKVE-------------VDMS----NPSMHDLQC---FFGN----EF---DQ

LNFVS-----------------YAK-E-NCSF------DL--------------------

----------DFVFFACSSVFLCIFIVVVLM------YKFVGQYFKPFYH----------

-----IANG--WFREALRMKEK-QQ----------Y--RYDAFVSYSG---K--DEHW-V

IEEL--LPNLE------QRGP----PFL---RLCLH-SRDFQLGHD-IVENITD-SIYAS

RRTLCLVSRN---YLNSNWC-SLEMQLATY--RLQVEHR---DI----LILVFLENIP-S

RLLS-SHHRLARLVKTRTYLDWP-----QEPE--MHDAFWDRLWCKLS------------

---------------------------------------------------------SNK

AN----------------------------------------------------------

------------------------------------------------------

>TLR20c_Dare

------------------------------------------------------------

-----------------------MKIPEKLKMVLLFALF---------------------

---------ILCPKTCYVCS----------------------------------------

------------------------------------WLAEKCHFFSDVEGLNIASFYH-G

K-TSIAECLSVTDIKEDL-RGL------PT---NLLNLLVQMDL----------------

------------------HFHGVLAPN-SFSRF-G----SLENLKIAGCF----------

------SEIPPEAFNG----LTNVTSLTITALDSKNCCE-------------VALD----

-------FSHLPSLTSLFISH--------------------------HDLS---LLA---

--------LNV---FEK-IPHLQW---------------------LYLDSVC-LKDLSDV

LC----------------------------------------------------RLANVK

L--------------LKLFSLDDWNI-RLQYQNC--SVFNTTDISTEFSIETVDLLLG--

--------------------------------------KLEHIDEGAFKPFGKLSLLHFS

VSNTDFLE-DLSLIGVHQISKISAIVD---------------------------VLNVDD

LCVAAKLYSVKSVDV----YYKTINLSLT-SKGSVGCKEIGYITLENDISREIVNLLDVN

SVFQIFSNLT--SAAIYR---------HVLRSNDFQS-----------------------

--------------------------------------------------------LCAS

YPQNVKQLINMVLQTIRID----KI-------------VSHQF----MCFVNLKTL----

----------KLVMS---------------------------------------------

--KISV-----FEDFAFI--GL---N------KLKELNLHR--NK---ISSIHRHT----

----FSGLHE-----------------------------LRVLD---------------L

QENPI-IYIEPK----------------------------------SFGHFT-NL-----

---SSFLLGDL-------------------------------------------NFPPNM

--------------SLIKLHLSDIF-------------------------------GVIP

YNLSNVFISSGLRPMHLV---------------------------------------IGS

NTTLNNGLNLHIKGQYVIVEDCNSLLLTSV-FT----LQIQAA----------YMICEN-

------------------------EFIGK-------------------------------

------------------------------------------------------------

------------------------------------------------------------

-------------------------YVPSV---VSLEFQSMFSDNIG-------------

----DL--------------------SV----INQLVHLKT-LKLEK----LDLT---NL

PNMDIMFHNL-TKLETLILANC--KLFFLDGSL-TKD----LKALTKLGLIPKDTVNIL-

--QTFVDH-----L------------------------TQLR-FVYLED----LDLYCS-

--CDNA--------------------------------------------W---L--VSW

IR----DNRKVE-------------VVMS----NPSMQDLKC---LTDN----EV---DH

ISFVS-----------------YVT-E-NCSF------DL--------------------

----------DFVFFACSSVFLCIFIVVVLM------YKFVGQYFKPFYH----------

-----IASG--WFREAFRMKEK-QQ----------Y--RYDAFVSYSS---K--DEHW-V

IEEL--LPNLE------QRGP----PFL---RLCLH-SRDFQLGHD-IVENITD-SIYAS

RRTLCLVSRN---YLNSNWC-SVEMQLATY--RLQVEHR---DI----LILVFLETIP-S

RLLS-SHHRLARLVKTRTYLDWP-----QESK--MHEAFWDRLWCKLS------------

------------------------------------------------------------

-SNKAN------------------------------------------------------

------------------------------------------------------

>TLR19a_Dare

------------------------------------------------------------

-------------------------MFAFTTNSNFLRFF---------------------

---------WRFALGLSICNVVLTRI----------------------------------

------------------------------------TKRCITIEEHLLKQMPWTPHCL-H

Y-PGKGPYANCNITDLS--TDL------SQVGFEVRTLCVFG------------DIT---

-----------------------SIPAKAFSHL-P----SLEVLHIDGIRL---------

------ERVQAGAFEG----LPNLKYLSM--LFSDELYR----------LVRIDNRS---

-------FAGLNNLEELSLTG--------------------------LMLL---------

--------NGSSGIFDP-LVSLIR---------------------LDIVRTC-AQDLGEI

FC---------------------C------------------------------ISNGMT

R--------------LRHLNVEDSEI------ST--IENKGCPGGSMTWPLTALSGVQ--

-------------------------------------------------------NLYLI

GNNIKYIQ-ANSLIIFQNLSSLFLEFE------------------------------GKS

LGSIWESGVGKVNDL----TLKGKVLKKYSTNFKDLCHLVSSLYSQSLSLVYTSID---R

LTAEDLKDCG--TKLTKLLIQNSKIDNLDFRFWTSKL-----------------------

-------------EMQALQMAYMKLTDAP---------------------------FCFV

ANSTMWSLTSLDLTGNSIT----NI-------------EGDQF----ACMPFLEQL----

----------YLSQN---------------------------------------------

--AIKT-----LQLHAFR--GL---P------RLKILQLDS--NK---IWQLSAND----

----FKNLRA-----------------------------LEVL----------------L

INDNIIETIETG----------------------------------TFWDQR--------

---ELCELSFG---------RLEYVYE------------LH-------LENIFFEFPPKL

--------------QRLSIDAHYGT-------------------------------NIYI

GNASPPNGTFALELNGER------------------------------------------

----------------LGFVGCDSDVLMAV-RE----LKVNCT----------YFLCKD-

------------------------SFMAP-------------------------------

------------------------------------------------------------

------------------------------------------------------------

-------------------------YFLNL---ESLEIS---GGAER-------------

----AP-----------------LNYAT----INNLHHLKH-LKLAR----LNFP---NY

TESRSAFWNL-TQLQTLVVVNC--HLSFLTKSM-FRD----LTSLQLLRLYSD-SPLILT

D-GVFG-V-----L------------------------PVLK-AFILDR----VDFQCS-

--CENG--------------------------------------------W---L--LDW

AD----STEKVQ-------------VIYL--------QKQQC-----------VW---HY

QKLNF-----------------LATMEKLCQT------DA--------------------

----------QYICYVATASSISLLLSAAVG--Y---QFARW-PSLVLFL-----RLKGW

ME---RRFG--RRWNRRRRRME-DDYGEIEEM------QYDAFVSFCS---Q--DEAW-V

LGEM--ASRLE-----EQGNP----RL----RLCLH-NRDFEVGKD-IMDNITD-SIHNS

QCTVCLISRR---YLRSDWC-GLEMRVATH--RQLEEQK---HR----LILIFLQHIS-P

FELS-AFHRLAKLVRSRTYLDWP-----EEEG--DREHFWDRLRRNIA------------

---------------------------------------------------------EDS

EAS---------------------------------------------------------

------------------------------------------------------

>TLR19b_Dare

------------------------------------------------------------

-------------------------MFAFATNSNFLRFF---------------------

---------WRIALGLSICNVVLTRI----------------------------------

------------------------------------TKRCITIEEHLLKQMPWTPHCL-H

Y-PGKGPYANCNITDLS--TDL------SQVGFEVRTLCVFG------------DIT---

-----------------------SIPAKAFSHL-P----SLEVLHIDGIRL---------

------ERVQAGAFEG----LPNLKYLSM--LFSDDLYR----------LVKMDNRS---

-------FAGLNNLEELSLTG--------------------------LMLL---------

--------NGSSGIFDP-LVSLIR---------------------LDIVRTC-AQDLGEI

FC---------------------C------------------------------ISNGMT

R--------------LRHLNVEDSEI------ST--IENKGCAGGSMTWPLTALSGVQ--

-------------------------------------------------------NLYLI

GNNIKSIQ-ANSLNIFQNLSSLFLEFE------------------------------GKS

LGSIWESGVGKVNDL----TLKGKVLKKYSTNFKDLCHLVSSLYSQSLSLVYTSID---R

LTAEDLKDCG--TKLTKLLIQNSKIDNLDFRFWTSKL-----------------------

-------------EMQALQMAYMKLTDAP---------------------------FCFV

ANSTMWSLTSLDLTGNSIT----NI-------------DGDQF----ACMPFLEQL----

----------YLSQN---------------------------------------------

--AIKT-----LQLHAFR--GL---L------RLKILQLDS--NK---ISQLSAND----

----FKNLHA-----------------------------LEVL----------------L

INDNIIETIETG----------------------------------TFWDQR--------

---ELRELSFG---------RLEYVYE------------LH-------LENIFFEFPPKL

--------------QRLSIDAHYGT-------------------------------HIYI

GNASPPNGTFALELNGEK------------------------------------------

----------------LSFADCKSDVLMAV-RE----LKVNCT----------FFLCEN-

------------------------LFMAP-------------------------------

------------------------------------------------------------

------------------------------------------------------------

-------------------------YFLNL---ESFEIS---GGAER-------------

----AP-----------------LNYAT----INNLHHLKH-LKLAG----LNFP---NY

TESRSAFWNL-TQLQTLVMVNC--YLSFLTKSM-FRD----LTSLQLLRLYSD-SPLILT

D-GVFG-V-----L------------------------PVLK-AFILDR----VDFQCS-

--CENG--------------------------------------------W---L--LDW

AD----STEKVQ-------------VIYL--------QKQQC-----------VW---HY

QKLNF-----------------LATMEKLCQT------DA--------------------

----------QYICYVATASSISLLLSAAVG--Y---RFARW-PSLVLFF-----RLKGW

ME---RRFG--RRWNRRRRRME-DDYGEIEEM------QYDAFVSFCS---Q--DEAW-V

LGEM--APRLE-----DQGNP----RL----RLCLH-NRDFEVGKD-IMDNITD-SIHNS

QCTVCLISRR---YLRSDWC-GLEMRVATH--RQLEEQK---HR----LILIFLQHIS-P

FELS-AFHRLAKLVRSRTYLDWP-----EDEG--DREHFWDRLRRNIA------------

---------------------------------------------------------EDS

EAS---------------------------------------------------------

------------------------------------------------------

>Drme_Toll9

------------------------------------------------------------

---------------------------------MCPKYI---------------------

---------WDVIVLVCLFLGNVREA----------------------------------

------------------------------------YTEFSIQDGLIIEPDSATTSSEEA

E-------------------------EVSKERTDLKSLMLKY------------------

-----------------------ESDD-------G----NSCLLDLIKDEV---------

----IWWQFPNGTLRDSTKKYAHKLYLDL---SHGNLKD--------------DSDL---

-------FREAKLSRKVTIWR--------------------------TEVF---------

------------------SAAFNT---------------------LTAAPFRTLYSMRES

------------------------------------------------------------

---------------LKLLSLRGNNF----------AELIPDAEDFARFVNESRLEAS--

------------------------------------------------------------

---------NSVPHHCELLLLHNTT-----------------------------------

-------------------DLYDRECYLY---------FNNNTNMGQSITTGRNYTNFIK

VLKDRFDQHGSSS--------------QSIAWATFPK-----------------------

------------------------------------------------------------

-------------------------------------------------MPRLVEL----

----------DISNC---------------------------------------------

--SIEY-----VSKEAFR--NV---S------NLRRLFMSD--NK---IMTISHDT----

----FYYVQG-----------------------------VQYLD---------------L

SFTNF-----------------------------------------LTYSYQLQL-----

---PTLEMALS---------LIYGLKI------------QQ----------NVFKYLPEL

--------------IYLDL-SHSKM--------------------------------TRN

SAVAFAHLGDKLKFLSLC------------------------------------------

-------------YTAIPMVSSTIFKNTVL-EG----LDLSGN----------PYLSYN-

---------------------IIDDAFDG-------------------------------

------------------------------------------------------------

------------------------------------------------------------

-------------------------IANTL---KYLYFE---RSNIK-------------

----DL------------------EW------SKSLKNLQV-LGLAG----NNIN---AL

T--PAMFQSL-ESLEILDLSSN--HVGNWYRSA-FHN----NSALRVLNLRSN-TINMLS

N-EMLKDF------------------------------ERLD-YLSLGD----NDFICD-

--CHLRAVVEVAAANNKDADCSYRLLNYSQNAVGEEVISLAESLIIDRKLWQSRY--IPW

LQRSYSNIREFN-------------RANHIIKLRFSSEDYMVAKCSAAQ--PYHLGDLDG

DLTLKFQLLDYEASQYYCFNNTDQLQVDELNCQIRSMSDL--------------------

------AEELHHVTNTVIAVMGSLVGACILGFII---YLKRW-HIHYYYS---SLKSAAL

LSS--ASKESVNKFTNISQRDPSA--------------VYDIFISYCQ---N--DRTW-V

LNEL--LPNVE------ETG-----DV----SICLH-ERDFQIGVT-ILDNIIS-CMDRS

YSLMLIISSK---FLLSHWC-QFEMYLAQH--RIFEVSK---EH----LILVFLEDIP-R

RKRP-KTLQY--LMDVKTYIKWP-----TAKE--DRKLFWKRLKRSLE------------

----------------------V---------------------IG--------I--NSR

EISV--------------------------------------------------------

------------------------------------------------------

>TLR15_Gaga

------------------------------------------------------------

------------------------------------------------------------

---------MRILIGSLY------------------------------------------

------------------------------------FYFISFLFSKVNGFLTQRTSPV-S

S-FPFYNYSYLNLSSVSQ-AQA------PK---TARALNFSY------------------

------------------NAIEKITKR-DFEGF-H----VLEVLDLSHNHI---------

------KDIEPGAFEN----LLSLVSVDLSFNDKNLLVS-----GLAPHLKLIPTSG---

-------ASGPSQIYMYFQKS--------------------------AEAALEPSAP---

--------AELLPHLED-PPNPGN---------------------VNPRFRQRRTEENKT

SP----------------------------------------PAATLRPDLCGAPINGLL

D--------------LSRTKLSNEELTAKLDADL-----CQAQLGTVLEFNISHSDLE--

------------------------------------------------------------

---MDLLSLFILFLPMKDIQSVDAS-----------------------------------

---YNRITINNIDVE----AICHFPFSNF---------------------------SFLN

ISNNPINSLE--TVCLPESITV-----IDLSFTNIST-----------------------

--------------------------------------------------------IPAN

FAKKLSKLERMYVQGNQLI----YT---------VRPENPSATPRPPPGTVQISAI----

----------SLVRN---------------------------------------------

-----------QAGTPIE--SL---P-----ESVKHLKVSN--CS---IVELPEWF----

----ANRMQE-----------------------------LLFLD---------------L

SSNRIS----------------------------------------MLPDLPISL-----

---QQLDISNS---------DIKIIPP------------R------------FKS-LSNL

--------------TVFNI-----------------------------------------

---------QNNKLTEMH------------------------------------------

-----------------------PEYFPSTLTT----CDISKN----------KLKVLS-

------------------------------------------------------------

------------------------------------------------------------

------------------------------------------------------------

-------------------------LTKALENLESLNVS---GNLIT-------------

----RL--------------------EP----ACQLPSLTN-LDSSH----NLIS---EL

P--DHLGQSL-LMLKHFNLSGN--KISFLQRGS-LPA------SLEELDISDN-AITTIV

Q-DTFG-Q-----L------------------------TSLS-VLTVQG----KHFFCN-

--CDLY--------------------------------------------W---F--VNI

YI----RNPHLQ-------------INGK--------DDLRC---SFPP----DR---RG

SLVKS-----------------SNL-T-LLHC------SL--------------------

----------GIQMAITACMAILVVLVLTGLCWR---FDGLW-YVRMGWY---------W

CMA--KRRQ----YKKRPENK-----------------PFDAFISYSE---H--DADW-T

KEHL--LKKLE------TD------GF----KICYH-ERDFKPGHP-VLGNIFY-CIENS

HKVLFVLSPS---FVNSCWC-QYELYFAEH--RVLDENQ---DS----LIMVVLEDLP-P

DSVPQKFSKLRKLLKRKTYLKWS-----PEEH--KQKIFWHQLAAVLK------------

---------------------------------------------------------TTN

EPLVRAENGPNEDVIEME------------------------------------------

------------------------------------------------------

>Sako_TLR_5

------------------------------------------------------------

------------------------------------------------------------

------------------------------------------------------------

--------------------------------------MPSITTVLNLEDNSITTDGF-K

F------------PKLP----------------KLHYLTLAG------------------

------------------NPIDDIKRT-SFKTI-P----NLKTLSMYFTEI---------

------RHIEEGAFSE----LKMLQSLTL---SNIIIQH-------------IPSKT---

-------FQGLERLEFLDLSS--------------------------TYILE--SIA---

--------VDA---FSD-LHHLKY---------------------LDLHQNA-LTVIDTA

------------------------------------------------------VFQHLS

T--------------LQYLDLSNNKLSEIPDNIC--R--YLPTLQTLVLSNNNKLSSF--

---------------------------------------------AIRETCANLTNIQLD

STNITIVN-QEDFDAVSGHTLDGVS-----------------------------------

---FYQTPLQEIKVG----TFAGFQINRFSFGHKNSDFVNVLRGLTDCNMNQLAI----S

GLEDNFPSLS--TSTFHVA--------METTLGETLQ-----------------------

------------------------------------------------------------

---------QLTISFNHIK----AV-------------EDFAF----IAFPNLHEL----

----------NLTLN---------------------------------------------

--HIKT-----LGKDAFT--NL---V------FLQTLNLKS--NF---ISQIPDGI----

----FDNLRN-----------------------------LRYLN---------------L

YDNQL-AFLNKG----------------------------------VFDNLL-KL-----

---QEFVFGGQ---------QVSMIDA------------NP---------TVFLH-LSNL

--------------HTISI-TENII---------------------------------ND

ELKLPMSSLTKLKRLNFK------------------------------------------

--------------KISALQNDILHGCENGTSL----EDIEAD---------ESLWFVD-

---------------------HIPSINDC-------------------------------

------------------------------------------------------------

------------------------------------------------------------

--------------------------LLSV---KRMSIS---GVDLM-------------

----RGWKVCFWDL-----------RPG----IYLMQNLEE-LILSH----DQIS---DL

G--DNCFELM-NKLIRLNLSNN--KIQMLRSGL-WKG----LERLDMLDLRHN-AITRIE

Q-SAFQ-T-----L------------------------PSLK-QLYLGD----NPFVCD-

--CDLR--------------------------------------------W---F--QKQ

LS----TNIWQL------RPTEYQNMIYW-------LQDYQC---KLPE----TL---HG

TYVTD-----------------YKP-S-DFAC----------------------------

----------SLMAVAGLIAASTMTLFAITVVVL---RRYRY-CIRYRWF---------R

FKL--RYFG----YRALEGEDE-E-----------Y--LYDAYITHSE---DIQDIDW-I

CDHF--MPRLE------NEF-----NF----RITFR-HRDYIGGTN-RIENSRE-SLQRS

RHTVFVMTEK---ILESGIF-MFELQFAYQ--KLIEDKK---DL----IILVELEEIP-N

AELP-DLLHL--LFCSKDKIKWT-----ENEQ--GQMLFWEKIRHQIQ------------

---------------------------------------------------------SDN

RVDARK------------------------------------------------------

------------------------------------------------------

>TLR12_Xetr

------------------------------------------------------------

------------------------------------------------------------

---------MLL------------------------------------------------

------------------------------------RDPMFATRCSLYKNATSLALCG-N

I------------TNIT--ADI------SDVPSTINILCLSGSN----------------

------------------T----FIPEGTFANF-K----RIKYLFMKMPLV---------

-------RIYPNAFKG----LESLESLAISFLGHSKCNN-----------LSIPTDA---

-------FSNLYYLRDLSIEG--------------------------FKLV---------

--------KSMLIRLPF-STSLYS---------------------LSLPTNC-ITDLFHV

FH----------------------------------------------------IFQNLI

F--------------IHNLDVGSNKIERVSLQHD--LNISLPNITELVLEKNPLILIQ--

-----------------------------------------------QNALARFGIHLLN

LGFTKLNLEDILNAGVENLKSLSLTSA--------------------CRRAINKTHTLCS

IAQRFQLKSLSAPVN----RFSGIYTKDF----------LGCSSLEFLNLNHNHIHIVDP

DLLYTLPKLK--ELHLSYSLLHMNLCPLSYQDNFTSS-----------------------

------------------------------------------------------------

-------VEALYFIGNRIT----VL-------------KNKQF----FCMTKLTDL----

----------LLARN---------------------------------------------

--GIEH-----IEESAFW--GL---N------DLRVLDLRN--NR---LTSLQISS----

----LSGLSN-----------------------------LMELY---------------L

QNNVM-ESMQRY----------------------------------SLKWQT-KL-----

---RLVKIGFV---------SVSVSFE------------L----------------FPNT

--------------ETLDIQTTGKY----------------------------------I

TVFMNETAASSLKSLSVN------------------------------------------

---------GSHVELDIKCGHQFFSTLREL-------KVINNN----------GLISCS-

------------------HYGYQLEHFEN-------------------------------

------------------------------------------------------------

------------------------------------------------------------

--------------------------LENI----QYHFT---GAPLT-------------

----RN--------------------VN----FSNIANLRS-LEVEN----LATALDPNV

P-PNDLFHKL-HKLEVLKLFNC--GFKYMTTAL-LKD----MRSLRVLVMKNQ-MILAVD

P-GLQN-T-----L------------------------SPLE-FIYFID----VTFQCD-

--CDNS--------------------------------------------W---F--VTW

AL----EDKHTF-------------VSGIC--------SLFC---LQLN---------KK

FNLVN-----------------FSE-S-SCRQ------EI--------------------

----------DFMLFVITFTSLLLFIVLTMLCNI---FNSDIIQLFYVFQ----------

-----IWLK--KLRGKTNNPIA-------------Y--EYDAFVSYCS---R--DQGW-V

VNYL--VPNLE-----EKGRN----TI----KLCLH-NRDFLVGKD-IVDNIMD-SIYKS

RKTICLISYN---YLQSDWC-SLEMRMATY--KLLAEKN---DD----LILVFLESIS-G

YHLS-AYHRLARIVRKKTYIDWP-----QEEN--EQAEFWERLRMTVL------------

---------------------------------------------------------ETK

ED----------------------------------------------------------

------------------------------------------------------

>Ptfl_TLR_1

------------------------------------------------------------

------------------------------------------------------------

------------------------------------------------------------

-----------------------------------------------------MLDCS-R

K--NL--------TGIP--KHL------PAERNTIAALWLNG-N----------NIT---

------------------A----VNQD-LLENF-D----RLTVLWLESNMI---------

------RQLTSNAFAK----VPYLQHLHL---QGNEIQS-------------LNNDV---

-------FQGMQNLEILDIRK--------------------------NPLE---ELP---

--------DKT---FAE-LRNLHD---------------------LQLSMCY-LPELRSH

DL-----------------------------------------------------LFGLE

N--------------LQNLTLELCNI------SL--V--RHEVFGKVPNLKRLSLKGN--

------------------------------------------------------------

--KLHTID-SVIFRSLSKLKFLDLS---------------------------GNLLTQIP

QRLCDYTPLLADVSF----SFNPLHEIKF-------GKDYQKCSLRNIHLVSVTN-KTDL

LSNDSFIGIA--NSPLNK---------LDFHDN-EVS-----------------------

--YWRSEVFNSFTNLSFLDLSSIQVDYFTENRAGMST-----------------------

VGVQRSKVHTLRVSNNTIH----GI-----FFFFKLSFSAVAFATQAVNTSFLKEL----

----------DISRN---------------------------------------------

--QLSE-----LENNTFV--DF---S------SLEVLDISH--NL---ITHIEVAA----

----FSGLYN-----------------------------LLKLS---------------L

KDNNLWTIIPQT----------------------------------FNVQPNFPL-----

---TSLDLSEN---------VISYWQE------------K-----------AFTG-LKNL

--------------RTLNLGDQGKIRILPQNFDDLPSLEELDIFGN----------TWSD

QFHLPLCKMKNLTFLNIS------------------------------------------

----QAWVHSKYVFPLDDMSVKCPGYVSPL-KK----LDFKAS----------FVDNCK-

---------------------CVENYFSR-------------------------------

------------------------------------------------------------

------------------------------------------------------------

----------------------NWRAFQNL---ERLFISFMIHYDPD-------------

----YF----PWKI------------------FTKLSKLKV-LKLKG----NHIE---KI

P--PDIFANC-TNLTYLDLSLN--TIASLDSIP-CNG--L-H-NLETLDLQQN-AITTID

S-KFFE-Q-----L------------------------PKLK-KLNLNG----NKYDCS-

--CSFKS-----------------------------------------------T--DEF

LT----SSKAKSMISLTSNSHHFDDRPYW-------FQDYAC---DTPD----RL---RG

KTIAD-----------------FNPYN-TLLC------EP--------------------

----------SIIFTIMLVLFIFLVTVSVLL--E---RIFRY-EIRYLWF---------T

TKL--KFNC--QQYEPLVDDQT-------------Y--EYDVNISNSD---E--DNDW-V

ETNL--LPYLE------TT-L----HL----RVHFK-ERDMKPGTR-KPRYIADLIYKNS

RKTLFVISES---FIANGMC-MQELYTAFQ--KLFDNHM---NV----MVFVNLDKIP-D

KKRP---RILRFPLCQKRVNKWW--ECTSNER--WKKVFWETLKQHIS------------

-------------------------------------------------------EGSHV

NHITQIH-----------------------------------------------------

------------------------------------------------------

>Ptfl_TLR_24

------------------------------------------------------------

-----------------------------MENQQQRVAV---------------------

---------IVLVVMASAGY----------------------------------------

------------------------------------YCDYTSQPCHQHFYNTTILDCSRQ

N-----------LAGIP--KHL------PAERNTIMALWLNG------------------

------------------NNITVVNQD-LLENF-N----RLTVLWLESNMI---------

------RQLTSHAFAK----VTNLQHLHL---QDNEIQS-------------LNNDV---

-------FWGMYNLEILDIRK--------------------------NPLE---DLQ---

--------DRT---FAE-LTNLHE---------------------LQLSMCYLPELLSSN

------------------------------------------------------LLFGLE

N--------------LQNLTLELCN-----------------------------------

------------------------------------------------------------

------------------------------------------------------------

------------------------------------------------------------

---------------------------ISLIRREVFG-----------------------

------------------------------------------------------------

------------------------------------------------------------

------------------------------------------------------------

--KVPN-----LKRLSLK--GL---K------NLRTLNLGD--QG---KIKLLPQN----

----FDDLPS-----------------------------LEELD---------------I

FGNTWSDQSHL-----------------------------------PLCKMK-NL-----

---TFLNISQA---------------------------------------WMHSKYVFPL

--------------DDMSVKCPGYV-----------------------------------

---------SPLKKLDFK------------------------------------------

--------------------AYFVDNCKCVENY----FSRNWR-----------------

------------------------------------------------------------

------------------------------------------------------------

------------------------------------------------------------

-------------------------AFQNL---ERLNIRI--HYDPD-------------

----YF------------------PWSI----FTKLSKLKV-LKLKD----NRIE---KI

P--PDIFANC-TNLTHLDLSLN--TIASLDSIP-CKV----LHNLETLELQQN-AIATIE

S-KFFE-Q-----L------------------------PKLK-KLYLNG----NKYNCS-

--CSFKS-----------------------------------------------T--DEF

LT----SSKAKSMISLTSNSHHFDDRPYW-------FQDYAC---DAPD----RL---RG

KTIAD-----------------FNPNTLVCEP------ST--------------------

---------TEMAIFESLYSLGILIGLIPIFCIYQTLRIFRY-ELWYFWF-----TTELK

FNC--HRNC--HRNERLVEDQT-------------Y--EYDVNISNSD---E--DNDW-V

ETNL--LPHLE------TTL-----HL----RVHFK-ERDMQPRTW-KQTYIDDLINNNS

RKTLFVISES---FVANGKC-MQELYTAFK--KLFNKHM---NV----MVFVNLDKIP-E

KKQP-RILRLQ-LCQRRVNKWWE---CTSNER--RKKVFWETLKQHIS------------

----------------------E----------------------G--------C--HVN

HINQIH------------------------------------------------------

------------------------------------------------------

>TLR1_Dare

------------------------------------------------------------

------------------------------------------------------------

---------MKPSSGWWLVS----------------------------------------

------------------------------------VYLTCFHPSLIPAIQRIIVNYS-S

Q----------NLSSVP-----------DDLKPSTEDLDLSL-N----------HIQ---

------------------S----LNCR-DFNTT-P----RLRFLNLSWNIL---------

------ENIDRDTFTS----TPALEMLDL---SHNGLQN-------------LSEQPY--

-------LLHLGCLELLDLSS--------------------------NRFS---AMA---

--------LGE--EFSM-LKRLQW---------------------LGLSAKSI-------

------------------------------------------------------SIQDFT

H--------------ISNLTLRTLFI-NADGLLT--Y--EGNSLDDVHAEKAVIALSS--

------------------------------------------------------------

-TNVDIAIANDVFARFKEVEFTKVDGK------------------------------MEV

VQQMRSRALMRTVRL----EISNVKTTWE-F-----------------------------

---------------------------LTSSVNTILS-----------------------

------------------------------------------------------------

-----------------------------------------------STIRELSLT----

----------DLT-----------------------------------------------

---LTE-----MKDGANQ-SST---H------ILESFSTKRASVT---TFIFDQKM----

----LYDFFI---------------NTP-----------ARKVS---------------L

TESPI-IFMT------------------------------------CPGTIS-KI-----

---QELDLSDC---------ALTEKIF------------SV-------NPETECGTLVNL

--------------TRLVL-RGNNL-----------------------------------

---------KHLSPLTSR------------------------------------------

-----------------------INLMDSL-QY----IDLSQN----------TLTYSE-

-----------------------NQGRCF-------------------------------

------------------------------------------------------------

------------------------------------------------------------

-------------------------WPPRV---LHVDLS---RNGFD-------------

----EV--------------------VF----KCLPDSVQV-LNLRH----NRVS---TV

P---ADIHTF-DTLQVIDLTFN--RLLDLPTCRSFP-------SLQKLLIRSN-SIHSPV

P-GSLK-T-----C------------------------QHLQ-DLDLSH----NPFICT-

--CALRD-----------------------------------------------F--ASL

IN----AQGIKT---------FKSTLKHW-------PDGYRC---SYPE----SW---SN

STLED-----------------FYL-P-EISC------NA--------------------

----------WILAITILIPTISLIVAVSLLCNR---LDIPW-YVRMMWK---------W

TRA--KHYS--ITSQLKEEDVE-RL-------------HFHAFVSYSQ---K--NAGW-V

KSQF--LPKLE------GDC-----GL----RMCHH-ERDFIPGKT-VVQNILR-CIEQS

RRCVFVLSSH---FVQSEWC-HYELYFANH--QKLTRGM---DS----ILLILLEPLP-L

YLIPSKYYQLKTMMSRRTYLEWP-----QEGA--KQKLFWANLRAALQ------------

---------------------------------------------------------AEL

PNTPDREEE---------------------------------------------------

------------------------------------------------------

>TLR1L.1_Xetr

------------------------------------------------------------

----------------------------MRVHAAEYNLY---------------------

---------FLFAIIIYILA----------------------------------------

------------------------------------QFESNKALIIQRKLHKCFSSCP-S

V-CTL--------------------------------LDFTKEN-----------IT---

------------------H----LKTS-DFNCF-L----QLRLLNLSHNLI---------

------GELDCSVFKF----NPSLEYLDI---SNNRLHT-------------IKCHS---

-------LQYIKNIKRMDLSY--------------------------NNFK---TMD---

--------FCS--EFTA-LSQLKH---------------------LGLSSKKIQKDSFIN

------------------------------------------------------------

---------------IASMDLDFVFL-GIENEIE--Y--ENGSLQFLNTNKLHINLQP--

------------------------------------------------------------

--------------------NLSLA-----------------------------------

SSYLLSDALDTSMTL----EVSGAQC---------------------------------D

VHCDYFTKSF-----------------FTINKNSKVV-----------------------

------------------------------------------------------------

----NLIISNSTMPGNEML----KI---------------IPP----IWDSSVEHL----

----------HIK-----------------------------------------------

--MFRL-----IKEFKYV--KM---DFTHHSIKSFTLEYFT--ND---VFFFQGTH----

----PFNIFA----------------------EML----VENVT---------------F

SNAGM-IHFFCP----------------------------------PVPSIV--------

---RFLNLPSN---------RITDEIF------------LN------------CSSLNEL

--------------ELLNL-QNNKL-----------------------------------

---------EQMSKISSM------------------------------------------

-----------------------TLKMKNL-KH----LDLSKN----------GLHIDN-

------------------------EKQCN-------------------------------

------------------------------------------------------------

------------------------------------------------------------

-------------------------WMDSL---VFLNLS---ESGLT-------------

----NS----VFGC--------------------LPINLQI-LDLSK----NQIS---SI

P---IEVKNF-VSLKELHLASN--RLTDIPDCHNIGN------NLGVLKVDEN-FINLPS

K-EFLQ-N-----C------------------------EYVQ-YLSAGQ----NPFQCN-

--CDLRE-----------------------------------------------FVKMGV

MF-------PKR-------------LVGW-------PESYKC---ADPE----NV---KG

IFLQD-----------------FHL-S-EISC------NT--------------------

----------SLFLGVVLGTIFVVSIIVVSACFY---FDVPW-YIRMLFR---------W

FRT--KHRL--RNVNQQDIQND-------------K--LFHAFISYSQ---E--DSDW-V

KNML--LPNLE------RKDG----SI----KICHH-ERHFIPGKA-IIENIID-CIEKS

FKSIFVLSPN---FIQSDWC-HYELYFAQH--TLFGKNS---NN----LILILLDPIP-Q

YSIPNKYNKLRSIMKHRTYLEWP-----KEKG--KHALFWANLREAIH------------

------------------------------------------------------VNLSIK

EEDMADPEITIT------------------------------------------------

------------------------------------------------------

>TLR1L.2_Xetr

------------------------------------------------------------

------------------------------------------------------------

---------MLSIYILV-------------------------------------------

------------------------------------QSESSKASFIHRRLPRGLSNCP--

--------------------------------SADTQLDFTK------------------

------------------RNITQLQTS-DFSCF-L----DLKFLNLSYNSI---------

------EELDCSVFQF----NPSLEYLDI---SNNRLHT-------------IQCQS---

-------LQYIKHIKHLDLSY--------------------------NNFK---QMH---

--------FCK--EFTA-LSQLKH---------------------LGLSAEQIQTN----

------------------------------------------------------SFLNIA

PMQ------------LEYVFLGMEDL------TE--Y--ENGSLQFLNTNKLLINLPT--

------------------------------------------------------------

--NLNLASSYLLSDALNTSTTLEVSG----------------------------------

-------------------AQCDVHCDYFTKSFSTITKNSKVVNL--------------I

ISNGAMPGNE--IFKI-----------MPPIWDSSIE-----------------------

------------------------------------------------------------

-----------------------------------------------HLYIKIFRL----

-------------QN---------------------------------------------

--ELKY-----VKMDFLH-HSI---K------SLTVEDLTNEVVS---FRDTHPLQ----

----MFAEML-----------------------------VENIT---------------F

SRAELYFFFCP-----------------------------------PAPSIF--------

---RVINLPSN---------RLTDDIF------------QN------------CANLKEL

--------------ELLNL-QNNKL-----------------------------------

---------EQMSKISSM------------------------------------------

-----------------------TLTMPNL-KH----LDLSRN----------GLHIDN-

------------------------EKQCK-------------------------------

------------------------------------------------------------

------------------------------------------------------------

-------------------------WMDSL---VFLNLS---ESGLT-------------

------------------------NSVF----GCLPINLQI-LDLSK----NQIS---SI

P---IEVKNF-VSLKELHLASN--RLTDIPDCHNIGN------NLGVLKVDEN-LINLPS

K-EFLQ-N-----C------------------------EYVK-YLSAGK----NPFQCN-

--CDLRE-----------------------------------------------FVKMGV

MF-------PKR-------------LIGW-------PESYKC---ADPE----NL---RG

IFLQD-----------------FYL-P-EISC------NI--------------------

----------SMLLGVVLGTIFILSIIVVSACFY---FDVPW-YIRMLFR---------W

FRT--KHRL--RNVNQQDIQND-------------K--LFHAFISYSQ---E--DSDW-V

KNML--LPNLE------RKDG----SI----KICHH-ERHFIPGKA-IIENIID-CIEKS

FKSIFVLSPN---FIQSDWC-HYELYFAQH--TLFGKNS---NN----LILILLDPIP-Q

YLIPNKYNKLRSIMKHRTYLEWP-----KEKG--KHALFWANLREAIH------------

---------------------------------------------------------VNL

SIKEEDMADPEVRT----------------------------------------------

------------------------------------------------------

>TLR1_Rhty

------------------------------------------------------------

------------------------------------------------------------

--------MVNILFFFVWIL----------------------------------------

------------------------------------MFQPCHSLCFPVVNDIVRNYSS-S

S------------LSTVP-KNL------PN---LTNILDLSQ-N----------NIT---

------------------E----IHMQ-DFAPL-H----QLKYLNLSSNKI---------

------SNLAPAFFRF----NQKLECLDL---SRNQLMN-------------VECDF---

-------LHNVTSLKYLDISE--------------------------NNFL---SLT---

--------LGK--AFSF-LQDLEY---------------------LSIGSRKTIKFRKDD

LK----------------------------------------------------EISGKQ

---------------LQEVSIKLKML------SE--Y--DPSALTILRTAKLHIVLPS--

------------------------------------------------------------

---------IKSFHFLKNVLDDAFN--------------------------TSDILTLSN

FECCQNCNHLSVDCC----QVCRKISKRM-SQCCSDCSHYIES----------------F

KVLGKYSRVQ--NLSLQH---------LTVDWETFAK-----------------------

------------------------------------------------------------

-----------------IL----KI----------------------VWDSSVEKL----

----------SVSNM---------------------------------------------

--KICQ-----VRQHLSW-FFI---Q-----KKLKTFTLRKVNIL---PFYFSQAI----

----IYDVFE---------------ELK-----------VENLT---------------I

HESGMIFMTC------------------------------------PKKGNTYKL-----

-----IDISDN---------SFTSDFF------------F-----------QGCRTLKHL

--------------ETFIL-KQNRF-----------------------------------

-----------VQLFEVS------------------------------------------

---------------------NMTTYMTSL-KH----LDVSQN----------HLILDE-

------------------------IRICP-------------------------------

------------------------------------------------------------

------------------------------------------------------------

-------------------------WTNSL---TKLNLS---SNRLT-------------

----DS--------------------VF----TCLPSNLEI-LDLQK----NNIY---TV

P---KVLKNL-NNLKELYLGAN--KLASPPDCNKFR-------NLEILFVEAN-SFHEPS

S-IFLH-S-----C------------------------QRLT-VLNAAS----NPFTCT-

--CNLRD-----------------------------------------------F---TT

MN----KNTHIE-------------MIGW-------PKSYRC---AYPD----IL---KG

TLLKD-----------------FHL-S-EVTC------SP--------------------

----------TLLLVIVLGTMFVLAILIGLMCHF---LDLPW-YLRMTWQ---------W

TQM--KRRT--MKTDSYQLSEN-------------L--AYHAFVSYSQ---H--DYSW-V

KEQL--LSNLE------ER------NL----RICHH-ERDFIPGKG-IIENIIN-CIEKS

YKSIFVLSPN---FIQSEWC-HYELYFAQH--QVLSEQT---EN----LILIVLEPIP-Q

YLIPSKYYKLKSLMAKKTYLEWP-----NDKN--KQRLFWANLQAAIG------------

------------------------------------------------------V-SLMA

PRQDIVSVHND-------------------------------------------------

------------------------------------------------------

>TLR1_Xetr

------------------------------------------------------------

------------------------------------------------------------

---------MAAIFFLLI------------------------------------------

------------------------------------YLSSSGQCVRQEIFESHIANYS-N

K-FLF---------AVP-----------KNLSSLTTVLDISF-N----------SIA---

------------------T----METS-EFNYI-F----DLQVLNASYNKM---------

------KYLNSSIFKF----NTQLQYLDF---SHNVLRN-------------ISGTF---

----------PSHIQHLDISF--------------------------NNFQ---TLS---

--------VCS--GFGN-MVLLEY---------------------LGLGADQILKS----

------------------------------------------------------DFEGIV

HLH------------LKEVFIELNSL-------------NDYENGSLLLLNTKKLTLD--

------------------------------------------------------------

---------FGVLSNEDRYNVLFDA---------------------------------VN

TTSVLELSHLQRWNI----HIDPKKYIFN------------------------------I

VQNSKVTDLT--MRHVNVEWKM-----IVLALQYIWH-----------------------

------------------------------------------------------------

-----------------------------------------------SSVETLTFY----

----------DFS-----------------------------------------------

--LKGL-----IAKTSFD-YSN---T------SVKAVNVQGVNVG---VFQFDQSS----

----VYRVFS---------------NMN-----------IENLT---------------L

NFASLLFMVC------------------------------------PLNSS--TF-----

---QSIDFSHN---------ALTDDLF------------KD------------CFTLINL

--------------KRLKL-RKNKL-----------------------------------

---------EKIYKLSAM------------------------------------------

-----------------------TENMPSL-EY----LDASHN----------LLAYDE-

-------------------------EDCH-------------------------------

------------------------------------------------------------

------------------------------------------------------------

-------------------------WSQNI---VELNLA---SNFLT-------------

----AS--------------------IF----KCLPISIKI-LILES----NDIT---HV

P---SGVTHL-DDLVELNLSFN--RLGDLPDCS------NIS-SLSLLGVEGN-QILYPS

I-ESIE-S-----C------------------------SRVK-HISAGQ----NPFQCN-

--CE--------------------------------------------------L--RRF

IN----QEKEAPGT-----------LIGW-------PEAYVC---KYPD----HL---RG

TMLKD-----------------FYI-S-EITC------NV--------------------

----------FILIPVIILPIIISIALIIGLCKY---LDGPW-FLKMIWQ---------Y

TRT--KQRT--RTSKQGYQSLQ-RD----------F--DFNAFISYSE---H--DASW-V

KNIF--LPSIE------RSND----CI----RICQH-ERNFIPGKS-IIENIIN-CIEKS

YKSIFILSPN---FVQSEWC-HYELYFAHH--KLYTENN---DN----LILILLEPIP-Q

YLIPSKYYKLKTLMAQRTYLEWP-----SEKS--KHGLFWANLRAAIS------------

----------------------I---------------------DL--------T--HAE

SEIPYSISSENPCSISSENPHSISSENPYSISSDNPCLGTSENPCSISSENPYSISSENP

C-----------------------------------------------------

>TLR10_Hosa

------------------------------------------------------------

------------------------------------------------------------

---------MRLIRNIYIFCSI--------------------------------------

------------------------------------VMTAEGDAPELPEERELMTNCS-N

M--SL--------RKVP--ADL------TP---ATTTLDLSY-N----------LLF---

------------------Q----LQSS-DFHSV-S----KLRVLILCHNRI---------

------QQLDLKTFEF----NKELRYLDL---SNNRLKS-------------VT------

-------WYLLAGLRYLDLSF--------------------------NDFD---TMP---

--------ICE--EAGN-MSHLEI---------------------LGLSGAKIQKSDFQK

------------------------------------------------------------

---------------IAHLHLNTVFL-GFRTLPH--Y--EEGSLPILNTTKLHIVLPM--

------------------------------------------------------------

---------------------------------------------------------DTN

FWVLLRDGIKTSKIL----EMTNIDGKSQ------------------------------F

VSYEMQRNLS-----------------LENAKTSVLL-----------------------

------------------------------------------------------------

-------LNKVDLLWDDLF----LI---------------LQF----VWHTSVEHF----

----------QIR-----------------------------------------------

--NVTFGGKAYLDHNSFD--YS---N-----TVMRTIKLEH--VH---FRVFYIQQ----

----DKIYLL-----------------------------LTKMD---------------I

ENLTISNAQMPH-------------------------------M--LFPNYPTKF-----

---QYLNFANN---------ILTDELF------------KR------------TIQLPHL

--------------KTLIL-NGNKL-----------------------------------

------------ETLSLV------------------------------------------

---------------------SCFANNTPL-EH----LDLSQN----------LLQHKN-

------------------------DENCS-------------------------------

------------------------------------------------------------

------------------------------------------------------------

-------------------------WPETV---VNMNLS---YNKLS-------------

----DS--------------------VF----RCLPKSIQI-LDLNN----NQIQ---TV

P--KETIHLM--ALRELNIAFN--FLTDLPGCSHFS-------RLSVLNIEMN-FILSPS

L-DFVQ-S-----C------------------------QEVK-TLNAGR----NPFRCT-

--CE--------------------------------------------------L--KNF

IQ----LETYSEVM-----------MVGW-------SDSYTC---EYPL----NL---RG

TRLKD-----------------VHL-H-ELSC------NT--------------------

----------ALLIVTIVVIMLVLGLAVAFCCLH---FDLPW-YLRMLGQ-----CTQTW

HRV----------RKTTQEQLK--------RN---V--RFHAFISYSE---H--DSLW-V

KNEL--IPNLE------KEDG----SI----LICLY-ESYFDPGKS-ISENIVS-FIEKS

YKSIFVLSPN---FVQNEWC-HYEFYFAHH--NLFHENS---DH----IILILLEPIP-F

YCIPTRYHKLKALLEKKAYLEWP-----KDRR--KCGLFWANLRAAIN------------

------------------------------------------------------VNVLAT

REMYELQTFTELNEESRGSTISLMRTDCL-------------------------------

------------------------------------------------------

>TLR1_Hosa

------------------------------------------------------------

------------------------------------------------------------

---------MTSIFHFAIIF----------------------------------------

------------------------------------MLILQIRIQLSEESEFL-VDRS-K

N--GL--------IHVP--KDL---------SQKTTILNISQ------------------

------------------NYISELWTS-DILSL-S----KLRILIISHNRI---------

------QYLDISVFKF----NQELEYLDL---SHNKLVK-------------ISCHP---

----------TVNLKHLDLSF--------------------------NAFD---ALP---

--------ICK--EFGN-MSQLKF---------------------LGLSTTHLEKSSV--

------------------------------------------------------LPIAHL

N--------------ISKVLLVLGE------TYG--EKEDPEGLQDFNTESLHIVFPT--

------------------------------------------------------------

---------------NKEFHF---------------------------------------

---ILDVSVKTVANL----ELSNIKCVLE------------------------------D

NKCSYFLSIL-----------------AKLQTNPKLS-----------------------

----------------------------------------------------------NL

TLNNIETTWNSFIRILQL-----------------------------VWHTTVWYF----

----------SIS-----------------------------------------------

--NVKL-----QGQLDFR-DFDYSGT------SLKALSIHQVVSD---VFGFPQSY----

----IYEIFS----NMN----------------------IKNFT---------------V

SGTRM-VHML------------------------------------CPSKIS-PF-----

---LHLDFSNN---------LLTDTVF------------EN------------CGHLTEL

--------------ETLIL-QMNQL-----------------------------------

---------KELSKIAEM------------------------------------------

-----------------------TTQMKSL-QQ----LDISQN----------SVSYDE-

-----------------------KKGDCS-------------------------------

------------------------------------------------------------

------------------------------------------------------------

-------------------------WTKSL---LSLNMS---SNILT-------------

------------------------DTIF----RCLPPRIKV-LDLHS----NKIK---SI

P---KQVVKL-EALQELNVAFN--SLTDLPGCGSFS-------SLSVLIIDHN-SVSHPS

A-DFFQ-S-----C------------------------QKMR-SIKAGD----NPFQCT-

--CELGE-----------------------------------------------F--VKN

ID----QVSSEV-------------LEGW-------PDSYKC---DYPE----SY---RG

TLLKD-----------------FHM-S-ELSC------NI--------------------

----------TLLIVTIVATMLVLAVTVTSLCSY---LDLPW-YLRMVCQ---------W

TQT--RRRA--RNIPLEELQRN-------------L--QFHAFISYSG---H--DSFW-V

KNEL--LPNLE------KE------GM----QICLH-ERNFVPGKS-IVENIIT-CIEKS

YKSIFVLSPN---FVQSEWC-HYELYFAHH--NLFHEGS---NS----LILILLEPIP-Q

YSIPSSYHKLKSLMARRTYLEWP-----KEKS--KRGLFWANLRAAIN------------

---------------------------------------------------------IKL

TEQAKK------------------------------------------------------

------------------------------------------------------

>TLR1_Mumu

------------------------------------------------------------

------------------------------------------------------------

---------MTKPNSL--------------------------------------------

------------------------------------IFYCIIVLGLTLMKIQLSEECE--

---LIIKRPNANLTRVPKDLPL-----------QTTTLDLSQ------------------

------------------NNISELQTS-DILSL-S----KLRVLIMSYNRL---------

------QYLNISVFKF----NTELEYLDL---SHNELKV-------------ILCHP---

----------TVSLKHLDLSF--------------------------NAFD---ALP---

--------ICK--EFGN-MSQLQF---------------------LGLSGSRVQSSSVQL

------------------------------------------------------------

---------------IAHLNISKVLL---VLGDAYGEKEDPESLRHVSTETLHIVFPS--

------------------------------------------------------------

---------------------------------------------------------KRE

FRFLLDVSVSTTIGL----ELSNIKCVLE--------DQGCSYFLRALSKLGKNL----K

LSNLTLNN-------------------VETTWNSFIN-----------------------

------------------------------------------------------------

--------------------------------------ILQIVWHTPVKYFSISNV----

----------KLQGQ---------------------------------------------

-----------LAFRMFNYSDT----------SLKALSIHQVVTD---VFSFPQSY----

----IYSIFANMN--------------------------IQNFT---------------M

SGTHMVHMLCPS----------------------------------QVSPF---------

---LHVDFTDN---------LLTDMVF------------KD------------CRNLVRL

--------------KTLSL-QKNQL-----------------------------------

---------KNLENIILT------------------------------------------

-----------------------SAKMTSL-QK----LDISQN----------SLRYSD-

-----------------------GGIPCA-------------------------------

------------------------------------------------------------

------------------------------------------------------------

-------------------------WTQSL---LVLNLS---SNMLT-------------

------------------------GSVF----RCLPPKVKV-LDLHN----NRIM---SI

P---KDVTHL-QALQELNVASN--SLTDLPGCGAFSS-------LSVLVIDHN-SVSHPS

E-DFFQ-S-----C------------------------QNIR-SLTAGN----NPFQCT-

--CELRD-------------------------------------------F---VKNIGW

VA-------REV-------------VEGW-------PDSYRC---DYPE----SS---RG

TALRD-----------------FHM-S-PLSC------DT--------------------

----------VLLTVTIGATMLVLAVTGAFLCLY---FDLPW-YVRMLCQ---------W

TQT--RHRARHIPLEELQRNL-----------------QFHAFVSYSG---H--DSAW-V

KNEL--LPNLE------KDDI----------QICLH-ERNFVPGKS-IVENIIN-FIEKS

YKSIFVLSPH---FIQSEWC-HYELYFAHH--NLFHEGS---DN----LILILLAPIP-Q

YSIPTNYHKLKTLMSRRTYLEWP-----TEKN--KHGLFWANLRASIN------------

--------------------------------------------------------VKLV

NQAEGTCYTQQ-------------------------------------------------

------------------------------------------------------

>TLR6_Hosa

------------------------------------------------------------

-------------------------------MTKDKEPI---------------------

---------VKSFHFVCLMI----------------------------------------

------------------------------------IIVGTRIQFSDGNEFAVDKSKR-G

L------------IHVP--KDL---------PLKTKVLDMSQ------------------

------------------NYIAELQVS-DMSFL-S----ELTVLRLSHNRI---------

------QLLDLSVFKF----NQDLEYLDL---SHNQLQK-------------ISCHP---

----------IVSFRHLDLSF--------------------------NDFK---ALP---

--------ICK--EFGN-LSQLNF---------------------LGLSAMK-LQKLDLL

PI-------------------------------------------------------AHL

H--------------LSYILLDLRN------YYI--KENETESLQILNAKTLHLV-FH--

------------------------------------------------------------

---------------PTSLFAIQVNIS---------------------------------

---VNTLGCLQLTNI----KLNDDNC---------------------------------Q

VFIKFLSELT--RGSTL----------LNFTLN-HIE-----------------------

-----------------------------------------------------------T

TWKCLVRVFQFLWP---------------------------------KPVEYLNIY----

----------NLT-----------------------------------------------

--IIES-----IREEDFT-YSK---T------TLKALTIEHITNQ---VFLFSQTA----

----LYTVFS----EMN----------------------IMMLT---------------I

SDTPFIHMLC--------------------------------------PHAPSTF-----

---KFLNFTQN---------VFTDSIF------------EK------------CSTLVKL

--------------ETLIL-QKNGL-----------------------------------

---------KDLFKVGLM------------------------------------------

-----------------------TKDMPSL-EI----LDVSWN----------SLESGR-

-----------------------HKENCT-------------------------------

------------------------------------------------------------

------------------------------------------------------------

-------------------------WVESI---VVLNLS---SNMLT-------------

------------------------DSVF----RCLPPRIKV-LDLHS----NKIK---SV

P---KQVVKL-EALQELNVAFN--SLTDLPGCGSFS-------SLSVLIIDHN-SVSHPS

A-DFFQ-S-----C------------------------QKMR-SIKAGD----NPFQCT-

--CELRE-----------------------------------------------F--VKN

ID----QVSSEV-------------LEGW-------PDSYKC---DYPE----SY---RG

SPLKD-----------------FHM-S-ELSC------NI--------------------

----------TLLIVTIGATMLVLAVTVTSLCIY---LDLPW-YLRMVCQ---------W

TQT--RRRA--RNIPLEELQRN-------------L--QFHAFISYSE---H--DSAW-V

KSEL--VPYLE------KE------DI----QICLH-ERNFVPGKS-IVENIIN-CIEKS

YKSIFVLSPN---FVQSEWC-HYELYFAHH--NLFHEGS---NN----LILILLEPIP-Q

NSIPNKYHKLKALMTQRTYLQWP-----KEKS--KRGLFWANIRAAFN------------

---------------------------------------------------------MKL

TLVTENNDVKS-------------------------------------------------

------------------------------------------------------

>TLR6_Mumu

------------------------------------------------------------

--------------------MVKSLWDSLCNMSQDRKPI---------------------

---------VGSFHFVCAL-----------------------------------------

------------------------------------ALIVGSMTPFSNELESM-VDYS-N

R--NL--------THVP--KDL---------PPRTKALSLSQ------------------

------------------NSISELRMP-DISFL-S----ELRVLRLSHNRI---------

------RSLDFHVFLF----NQDLEYLDV---SHNRLQN-------------ISCCP---

----------MASLRHLDLSF--------------------------NDFD---VLP---

--------VCK--EFGN-LTKLTF---------------------LGLSAAK-FRQLDLL

PV-------------------------------------------------------AHL

H--------------LSCILLDLVS------YHI--KGGETESLQIPNTTVLHLV-FH--

------------------------------------------------------------

---------------PNSLFSVQVNMS---------------------------------

---VNALGHLQLSNI----KLNDENC---------------------------------Q

RLMTFLSELT--RGPTL----------LNVTLQ-HIE-----------------------

-----------------------------------------------------------T

TWKCSVKLFQFFWP---------------------------------RPVEYLNIY----

----------NLT-----------------------------------------------

---ITER----IDREEFT-YSE---T------ALKSLMIEH--VK---NQVFLFSK----

----EALYSV---FAEMN---------------------IKMLS---------------I

SDTPF-IHMV------------------------------------CPPSPS-SF-----

---TFLNFTQN---------VFTDSVF------------Q-----------GCST-LKRL

--------------QTLIL-QRNGL-----------------------------------

---------KNFFKVALM------------------------------------------

-----------------------TKNMSSL-ET----LDVSLN----------SLNSHA-

-----------------------YDRTCA-------------------------------

------------------------------------------------------------

------------------------------------------------------------

-------------------------WAESI---LVLNLS---SNMLT-------------

------------------------GSVF----RCLPPKVKV-LDLHN----NRIM---SI

P---KDVTHL-QALQELNVASN--SLTDLPGCGAFS-------SLSVLVIDHN-SVSHPS

E-DFFQ-S-----C------------------------QNIR-SLTAGN----NPFQCT-

--CELRD------------------------------------------F----VKNIGW

VA-------REV-------------VEGW-------PDSYRC---DYPE----SS---KG

TALRD-----------------FHM-S-PLSC------DT--------------------

----------VLLTVTIGATMLVLAVTGAFLCLY---FDLPW-YVRMLCQ---------W

TQT--RHRA--RHIPLEELQRN-------------L--QFHAFVSYSE---H--DSAW-V

KNEL--LPNLE------KD------DI----RVCLH-ERNFVPGKS-IVENIIN-FIEKS

YKAIFVLSPH---FIQSEWC-HYELYFAHH--NLFHEGS---DN----LILILLEPIL-Q

NNIPSRYHKLRALMAQRTYLEWP-----TEKG--KRGLFWANLRASFI------------

---------------------------------------------------------MKL

ALVNEDDVKT--------------------------------------------------

------------------------------------------------------

>TLR1A_Gaga

------------------------------------------------------------

------------------------------------------------------------

---------MGSLTSIYVFACV--------------------------------------

------------------------------------FLSILWNNIQPTVENKITANYSGH

L-----------LTEVP-----------KNIPVHTHILDLSH------------------

------------------NSISEITNF-RFTSL-S----DLQVLNLSHNLI---------

------TELDFSAFMF----NQDLEYLDL---SHNNIWT-------------AYCQL---

----------LARLRHLDLSF--------------------------NKFT---VLP---

--------ICQ--EFGI-MFHLEY---------------------LGLSAMMIRRSDF--

------------------------------------------------------RYVAHL

Q--------------LDTVFLTLEDF----------SLYEPLSLTALNTRSLHIVFAT--

------------------------------------------------------------

---NQNFNFSLLYDGMSTSEKLKIVNL---------------------------------

-------------------RYTLSHKDFP------------------------------S

PSLELQKKIKTTDLTLDT---------VDLEWTVILQ-----------------------

----------------------------------------------------------IF

LLVWDSSVEHLTV-------------------------RNLIFRGPVVELTEYKHV----

----------PLLRS---------------------------------------------

-----------LEQLLSL--GS----------SMKALTLERVRNK---LYYFNQEI----

----LYRQFSEMN--------------------------IDSLT---------------I

HDACMPHMLCPK----------------------------------KRSSF---------

---QYINFSRN---------ALTDELF------------------------QNCDTLANL

--------------KILIL-HRNKF-----------------------------------

---------ESLSKVSFM------------------------------------------

-----------------------TSRMKSL-RY----LDMSSN----------LLRNSR-

-----------------------AEGRCQ-------------------------------

------------------------------------------------------------

------------------------------------------------------------

-------------------------WADSL---AELDLS---SNQLT-------------

------------------------EAVF----ECLPANINK-VDLQN----NQIA---NV

P---KGIAEL-HSLQELNLASN--RLADLPGCRAFTG-------LEILNIERN-LILTPS

A-DFFETC------------------------------PSVK-ELQAGQ----NPFKCS-

--CELQD-----------------------------------------------F--LRL

ER-----QSGGK-------------LSGW-------PEAYVC---KYPE----DL---SG

TQLED-----------------FHL-T-ELAC------NT--------------------

----------TLLLVTALLLTLVLVAVVAFPCIY---LDVPW-YVRMLWQ---------W

TQT--KRRA----WHDCPEERE-------------TALQFHAFISYSE---R--DSLW-V

KNEL--IPNLE------KGEG----CI----QLCQH-ERNFIPGKS-IVENIIN-CIEKS

YKSIFVLSPN---FVQSEWC-HYELYFAHH--RLFSENS---NS----LILILLEPIP-S

YVIPARYHKLKALMAKRTYLEWP-----KERS--KHALFWANLRAVVN------------

---------------------IK-------------------------------L-PTSF

ETDEEQSDVTSTSSITQCLIK---------------------------------------

------------------------------------------------------

>TLR1B_Gaga

------------------------------------------------------------

------------------------------------------------------------

------------------------------------------------------------

-----------------------------------------MTKNMRYLRNCFIYNCL--

------------------------------------------------------------

-----------------------FVFT-FWDNI------GLAKKNELFASV---------

----------PNNFLE--DGLDKKNMSFPHSYANNQHYK-------------ADYGW---

-------VVIENTTESLSLSE----------------------------------IA---

--------DDN---VRK-LITLLS------------------------------------

------------------------------------------------------KFRKGS

R--------------LRNLTLTNMSV----------------------------------

------------------------------------------------------------

------------------------------------------------------------

-------DWKDIIKV---------------------------------------------

---------------------------LQVVWHSSIE-----------------------

------------------------------------------------------------

----------------------------------------------YFNINNLTQL----

-------------GN---------------------------------------------

-----------VVSTRFD-YSK---T------SMKAFAVNKVLITDLYFSQDDIYN----

----IFANMN-----------------------------IAALT---------------I

AESELIHMLC------------------------------------PSSDSP--L-----

---RYINFSKN---------DLTDLLF------------QN------------CDKLIQL

--------------ETFIL-HRNKF-----------------------------------

---------ESLSKVSFM------------------------------------------

-----------------------TSRMKSL-RY----LDMSSN----------LLRNSR-

-----------------------AEGRCQ-------------------------------

------------------------------------------------------------

------------------------------------------------------------

-------------------------WADSL---AELDLS---SNQLT-------------

------------------------EAVF----ECLPANINK-VDLQN----NQIA---SV

P---KGITEL-HSLQELNLASN--RLADLPGCRAFTG-------LEILNIERN-LILTPS

A-DFFE-T-----C------------------------PSVK-ELQAGQ----NPFKCS-

--CELQD-----------------------------------------------F--LRL

ER-----QSGGK-------------LSSW-------PEAYVC---KYPE----DL---SG

TQLKD-----------------FHL-T-ELAC------NT--------------------

----------TLLLVTALLLTLVLVAVVAFLCIY---LDVPW-YVRMLWQ---------W

TQT--KRRA----WHDCPEERE-------------TALQFHAFISYSE---R--DSLW-V

KNEL--IPNLE------KGEG----CI----QLCQH-ERNFIPGKS-IVENIIN-CIEKS

YKSIFVLSPN---FVQSEWC-HYELYFAHH--KLFSENS---NS----LILILLEPIP-P

YVIPARYHKLKALMAKRTYLEWP-----KERS--KHALFWANLRAAIS------------

--------------------------------------------IN--------L--SVA

DEQNRTEV----------------------------------------------------

------------------------------------------------------

>TLR1.1_Chpi

------------------------------------------------------------

------------------------------------------------------------

------------------------------------------------------------

-----------------------------------------MAENRRPLTNFFFCSCV-F

T------------LTLWN-NIK------PS---DENEFIANYSS----------SLL---

------------------E---------DHSDY------ETKSLPLSHTKR---------

-----------LQFAS----------------ADNKINH---------------PSW---

-------DVEHNTSDSLVLSN----------------------------------IP---

--------ENG---ISD-LIQLLS------------------------------------

------------------------------------------------------KFNKTS

G--------------LKNLALNNIT-----------------------------------

------------------------------------------------------------

---------TSWVNFIKILQIVW-------------------------------------

-----------------------------------------------------------H

TSTEYFSIFK-----------------VKLMSN---------------------------

------------------------------------------------------------

------------------------------------------------------------

------------------------------------------------------------

-----------INKQSFN--YD---G-----TSLKAVIISNVSIQ---IFYFSQDD----

----LYCIFS----------------------EMN----IIALT---------------I

SDSKIIHMLC--------------------------------------PSKRSQF-----

---RFLNFSNN---------DLTDMVF------------QG------------CNNLDLL

--------------ETLIL-QRNQL-----------------------------------

---------KKLSKVSSM------------------------------------------

-----------------------TNKMKLL-KH----LDISRN----------MLYYDE-

-----------------------NENHCH-------------------------------

------------------------------------------------------------

------------------------------------------------------------

-------------------------WVETL---AKLNLS---SNKLT-------------

----DS----VFGC--------------------LPINVQI-LDLQN----NQIT---TV

T---KDITEL-KALKELNIAFN--RLTELPGCS-HYR------GLELLNIEEN-SILTPS

S-DFFH-S-----C------------------------QNIR-ELRGGH----NPFQCS-

--CELRD-----------------------------------------------F--VNF

EK----KSGGR--------------LVGW-------PESYVC---EYPD----GL---KG

TQLKD-----------------FQL-S-ELSC------NT--------------------

----------TLLLVIALVVTVVVVAVTSFLCIY---FDVMW-YLKMMWQ---------W

TQT--KRRV----RKSHPEDLQ-SI----------L--QFHAFISYSE---R--DSLW-V

KNHL--IPNLE------KEDG----SV----QICLH-ERNFIPGKS-IVENIIN-CIEKS

HKSIFVLSPN---FVQSEWC-HYELSFAHH--KLFSESS---NS----LILILLEPIP-Q

YLIPARYHKLKALMAKRTYLEWP-----KEKS--KHGLFWANLKVAIN------------

------------------------------------------------------I--NLP

ISANVV------------------------------------------------------

------------------------------------------------------

>TLR1.2_Chpi

------------------------------------------------------------

------------------------------MGSPTNIFL---------------------

---------FACIFTFTLWN----------------------------------------

------------------------------------NIQLSVENEFTAHYSSSFLACVPK

S-----------------------------QSRHTTMLDLSQ------------------

------------------KNISELHIS-DFSSL-P----ELQVLNLSHNLI---------

------RELDFNVFKF----NEKLECLDL---SHNNLWN-------------MCCQT---

----------LARLRHLDLSF--------------------------NKFK---TLP---

--------ICQ--EFGN-MLNLEY---------------------LGLSATMIRKS----

------------------------------------------------------DFRGIT

HLQ------------LHTVFLTLEDL------SH--Y--ESKSLTVLNTKNLHIVFPI--

------------------------------------------------------------

---NKNFSFPLLYDGMNTSEKLELS-----------------------------------

-------------------NIRYNST---------------------------------D

IPFPPFAPLK--FKTLNLRFNN-----VDLSWSIFAR-----------------------

----------------------------------------------------------IF

MIVWYTPVEYFTVKNLTFS----------------------------GSIQSMTNT----

----------DFS-----------------------------------------------

--HLYN-----WAQSNHS--GN---S-------MKALILEHIRTK---VFYFSQDI----

----LYKAFSDMN--------------------------IENLT---------------I

SDAYMPHMLCP-----------------------------------SHISLF--------

---QYLDFSYN---------ALTDEVF------------KN------------CDTLTHL

--------------KMLIL-QRNQL-----------------------------------

---------ENLSKVSSM------------------------------------------

-----------------------TSKMKSL-KH----LDISRN----------LLYYDE-

-----------------------NENHCH-------------------------------

------------------------------------------------------------

------------------------------------------------------------

-------------------------WVETL---AKLNLS---SNKLT-------------

------------------------DSVF----GCLPINAQI-LDLQN----NQIT---TV

T---KDITEL-KALKELNIAFN--RLTELPGCSHYRG-------LEFLNIEEN-SILTPS

S-DFFH-S-----C------------------------QNIR-ELRGGH----NPFQCS-

--CELRD-----------------------------------------------F--VNF

EK-----KSDGR-------------LVGW-------PESYVC---EYPD----GL---KG

TQLKD-----------------FQL-S-ELSC------NT--------------------

----------TLLLVIALVVTVVVVAVTSFLCIY---FDVMW-YLKMMWQ---------W

TQT--KRRV--RKSHPEDLQSI-------------L--QFHAFISYSE---R--DSLW-V

KNHL--IPNLE------KEDG----SV----QICLH-ERNFIPGKS--------------

-----IMCKK-----------LNRFTMELH--GINQITI---------LLIVFMIVNP--

------------------------------------------------------------

------------------------------------------------------------

------------------------------------------------------------

------------------------------------------------------

>TLR27_Rhty

------------------------------------------------------------

------------------------------------------------------------

---------MTPLRAIAILT----------------------------------------

------------------------------------CALFMPIYTMPKMIDTVLVNCS-G

I-GYPHSPCFLTP--------------------DVEVLDLSYNN-----------IT---

------------------M----IQQQ-NFQNL-N----KLRKLFLQFNHI---------

------TAIEPGSFAK----NSELRYLDL---SNNLLQD-------------ISVLP---

-------FNHLQSLTHLDITN--------------------------NMFD---TAD---

--------FGA--EINK-LQKLHS---------------------LRFGNTR-LSSLNSG

SL----------------------------------------------------SVLQGI

P--------------LKDVYLITGDL-QAF---------EPGTFNALQNVEKLSLDLQ--

---------------------------------------------------------FGQ

DGKLLINIFQDIPMSTTTLEILNSD----------------------------FVKKASN

VDFFFPLKKLNISTL----VAHNITIDD-------------------------------S

LTTYLLNSVL--GSNI-----------KELVLDTIIM-----------------------

------------------------------------------------------------

-----------------------------------------------DGIGNWDVI----

----------PIP-----------------------------------------------

-----------SDQINLR--------------KLQILNVEN-PNF---YHFYSLEY----

----LIDIFA---------------Q-------------LTELM---------------I

LKGNLFYVPC------------------------------------QISEIMTYL-----

---RHLDISFN---------LLQEFSF------------IP---------GKCSAPFPNL

--------------NTLIA-NNNKF-----------------------------------

---------SNLPKFSTL------------------------------------------

-----------------------LSKMEKL-SS----INASHN----------DLVLQK-

------------------------GLTCT-------------------------------

------------------------------------------------------------

------------------------------------------------------------

-------------------------WPQTL---KCINFS---HNNLE-------------

----GN--------------------VF----GCLPASLQS-LDLSY----NSIT---AV

P----NLKGL-RNLREIFLTEN--FIASFPEIP-VGY------SLKVLHIDQN-KITDIN

I-RFLQ-S-----L-------------------------DLA-ELKFSN----NPLECF-

--CAIQS-----------------------------------------------V--SNY

VQ----QRRVV--------------ILDW-------PDRYRC---ESPR----KF---KG

QIIQA-----------------LKF-S-PIEC------KI--------------------

----------PLFVGVLLACVALLVGVCIVLCIK---FNITW-YCRTLWL----------

-----WLKA--KKSLDANLVEK-N-----------F--EYNAFVSYSE---Y--DSAW-V

KNKL--LIQLE------NNEP----PY----RICIH-ERDFKPGKP-IINNIID-CISKS

YKTIFILSKH---FVQSEWC-HYEFFFAHQ--QIFDDKK---DS----LILLLLEPIP-K

NSIPDRFCKLRKLMNRNTYLEWP-----QNEF--QQGFFWKRLKAVLN------------

----------------------L---------------------DF--------H--SCS

SRVTLQGVIQEPDEIPDRV-----------------------------------------

------------------------------------------------------

>TLR2_Dare

------------------------------------------------------------

--------------------------------MRLVGTM---------------------

---------TAIILIMFILA----------------------------------------

------------------------------------QGLECSQTCKCDQMYFCNCSSN-N

L-HQV-----------------------PTVPSDVLGLDLSF------------------

------------------NQIESINMT-DLSSY-N----ELIILNLHKNKL---------

------RHIHRDAFKS----QHNLEVLDL---SLNNLNN-------------LSPSW---

-------FHKLKSLQQLNLVG--------------------------NPYS---TVG---

--------PAP--IFKS-LVNLRT---------------------LHLGSPS-LRELHKN

------------------------------------------------------GLDVLT

H--------------LDEMTFFGSNL-RSY---------ENGSLKAARPIGSVSLSLQ--

------------------------------------------------------------

----------NLFESDPELVSKVLQ-----------------------------------

-DVSHPETLLIIKDV----TMKTNTSTEPFKMVKEGGTKSLTFQNS-------------S

TTDQALTSF------------------LEFMDGSPLS-----------------------

------------------------------------------------------------

-FIGLEDIQFVGIGEWQKA----KY----------------------THHDSLRTA----

------------------------------------------------------------

--YLRN-----IEIEGFF--GF---S------SMIELGFLL--KH---------------

---------------------------------------FHNVS---------------V

INATV-FVIPKE----------------------------------TTFLLK-NL-----

---EYMDLSQN---------LLTDLTI------------QP-------TLYTGSGAYQNL

--------------NMLNV-SQNVL-----------------------------------

---------KSLGLMSRL------------------------------------------

-----------------------VTNLKKL-KY----LDLSYN----------SFVSMP-

-------------------------EKCS-------------------------------

------------------------------------------------------------

------------------------------------------------------------

-------------------------WPVTL---RFLNLS---STKLS-------------

----TL---------------------T----PCLPSSLTV-LDLSE----NDLK-----

-----AFKQRFPHLTTLILTGN--RLMKLPDGK---L----FPSLNTLLIQRN-ALRMFN

Q-SSLR-S-----F------------------------KTLL-YLEAGA----NNFVCS-

--CK--------------------------------------------------F--VSF

FK----KDVED--------------LITL----QDGRQNYVC---NTPF----TL---RG

NAIDS-----------------VRL-S-VFEC------YM--------------------

----------IPAVSVLCFGIITALGLVVLTCHK---LHVIW-YLQMTKA---------W

IQ---AKRK--PAVGRLPEEL-----------------RYDAFVSYSQ---H--DAEW-V

EEIL--VAELE------DTQP----SF----SLCLH-KRDFRPGRW-IVDNIID-SIEKS

YRTLFVLSEH---FVSSEWC-RYELDFSHF--RIMDEHN---DS----AVLVLLEPIK-K

ETIPKRFCKLRKIMNSRTYLEWP-----EDED--KRDEFWSNLRAALQ------------

----------------------R---------------------DE--------C-----

------------------------------------------------------------

------------------------------------------------------

>TLR2.1_Rhty

------------------------------------------------------------

-----------------------------MRSPSLLILL---------------------

---------VDCIFVRAAFL----------------------------------------

------------------------------------EGNTDPSDICQKCNSHNFCNCSSV

KLENV-----------------------PRVVENVLGFDLSH------------------

------------------NKISQIKDT-DFITY-V----KLKRLLLQSNRI---------

------HSISEQALQH----NTDLEYLDL---SNNLLTH-------------LSPHW---

-------FEHLSKLLYLNILG--------------------------NNYT---DLG---

--------SGR--IFSN-LTRLRW---------------------LEFGSPS-LSVLKKG

------------------------------------------------------DFVGVT

H--------------LDEFIVTAEKL------QV--Y--EKGSFSSFSSISHATLSLP--

------------------------------------------------------------

---------YTFLNKPSQAQQIFVD-----------------------------------

--LSEFTTHMELRNV----AFPD------------------------------------K

KDNQPFLPVQ--NSSLRK---------LSFRNTFLTE-----------------------

------------------------------------------------------------

----------------------------------------------NTVINFLNSM----

------------------------------------------------------------

--KNTKVSELVVKDSELS--GV---GQWYGIDNLKTNSLDT--IL---LSNISIKH----

----FYLFHD-----------------------------LSHIS-------DLFQSIKNA

TFTKLTMFLMPC----------------------------------TVSKKLENM-----

---EYLDLTDN---------LLSDRSL------------DE---------TVCSGSWRSL

--------------HYLIL-RKNLF-----------------------------------

---------QSLARTSTK------------------------------------------

-----------------------LTTLPKL-IH----LDLSQN----------RFSVIK-

-------------------------TSCK-------------------------------

------------------------------------------------------------

------------------------------------------------------------

-------------------------WSEKL---QLLNLS---SCDIK-------------

----NI---------------------E----ECVPPNVEV-LDLSN----NIIS---SF

A------VNL-PSLKELNLSNN--KFKSLPGDG-YLP------KMEILKISSN-KLTSLS

A-EEIK-T-----F------------------------QKLQ-FLEAGK----NNYICS-

--CE--------------------------------------------------F--LFY

MN----NGKTVQ-------------LLDR-------AEDYIC---DSPL----FL---RG

KMVQN-----------------TKR-S-FFDC------HK--------------------

----------TLSLALLCVGTILTVAIVVVMCYK---YHVFW-YIQMTWA----------

-----WLKA--KRKPKKVKKNN-------------I--CYDAFVSYSE---M--DSEW-V

ENLL--VSELE------SAHP----PL----TLCLH-KRDFIPGKW-IIDNIIE-SIEKS

RKTLFVLSQH---FVQSEWC-KYELDYTHF--RLFDEND---DS----AILVLLEMIP-K

ETIPQRFCKLRKLMNTKTYLEWP-----QDEV--EQQIFWFNLRIALQ------------

-----------------------------------------------------------G

DNITSL------------------------------------------------------

------------------------------------------------------

>TLR2.2_Rhty

------------------------------------------------------------

----------------------------MRSPSLLILLV---------------------

---------DCIFVRAAFLEG---------------------------------------

------------------------------------NTDPSDICQKCNSHNFCNCSSV-K

L------------------ENV------PRVAENVLGFDLSH------------------

------------------NKISQIKDT-DFITY-V----KLKRLLLQSNRI---------

------HSISEQALQH----NTDLEYLDL---SNNLLTR-------------LSPHW---

-------FEHLSKLQYLNILG--------------------------NNYT---DLG---

--------SGR--IFSN-LTRLRW---------------------LEFGSPS-LSVLKKG

------------------------------------------------------DFVGVT

H--------------LDEFIVTAEKL------QV--Y--EKGSFSSFSSISHATLSLP--

------------------------------------------------------------

---------YTFLNKPSQAQQIFVD-----------------------------------

--LSEFTTHMELRNV----AFPDKKDSQPFL----------------------------P

VQNSSLRKLS--FRN------------TFLTENTVIN-----------------------

----------------------------------------------------------FL

NSVKNTKVSELVVKDSELS----GV----------------------GQWYGIDNL----

------------------------------------------------------------

--KTNS-----LDTILLS--NI---S-------IKHFYLFHDLSH---ISDLFQS-----

---------------------------------------IKNAT---------------F

TKLTM-FLMPCT----------------------------------VSKKLE-NM-----

---EYLDLTDN---------LLSDRSL------------DE---------TVCSGSWRSL

--------------HYLIL-RKNLF-----------------------------------

---------QSLARTSTK------------------------------------------

-----------------------LTTLPKL-IH----LDLSQN----------RFSVIK-

-------------------------TSCK-------------------------------

------------------------------------------------------------

------------------------------------------------------------

-------------------------WSEKL---QFLNLS---SCDIK-------------

----NI---------------------E----ECVPPNVEV-LDLSN----NIIS---SF

A------VNL-PSLKELNLSNN--KFKSLPGDG-YLP------KMEILKISSN-KLTSLS

A-EEIK-T-----F------------------------QKLQ-FLEAGK----NNYICS-

--CE--------------------------------------------------F--LFY

MN----KGKTVQ-------------LLDQ-------AEDYIC---DSPL----IL---RG

KMVQD-----------------TKR-S-FFDC------HK--------------------

----------TLSLALLCVGTILTVAIVVVMCYK---YHVFW-YIQMTWA----------

-----WLKA--KRKPKKVKNNN-------------I--CYDAFVSYSE---M--DSEW-V

ENLL--VSELE------SAHP----PL----TLCLH-KRDFIPGKW-IIDNIIE-SIEKS

RKTLFVLSQH---FVQSEWC-KYELDYTHF--RLFDEND---DS----AILVLLEMIP-K

ETIPQRFCKLRKLMNTKTYLEWP-----QDEV--EQQNFWLSLKVALK------------

---------------------------------------------------------EES

NETVAK------------------------------------------------------

------------------------------------------------------

>TLR2.1_Xetr

------------------------------------------------------------

------------------------------------------------------------

--------MMQSISLSCLILSLV-------------------------------------

------------------------------------VTVLSKGNTDCPCDAAHFCHCS--

---------SMHWEAIP-----------SGLPRDVRGLNLSS------------------

------------------NAIQIVTET-DLQPY-D----QLQTLLLQYNAI---------

------HTINDGSFQP----LGNLEELDL---SYNNLSH-------------LSSAW---

-------FRNLYNLKHLNLLG--------------------------NQYL---TLG---

--------NNS--LFAS-LSSLRS---------------------LQFGNQD-FSAIQKL

------------------------------------------------------HFESLE

R--------------LNILEIKAAQL------GL--Y--EEGSLAAIKQINHAVLTVN--

------------------------------------------------------------

---------------IQNLRALLND-----------------------------------

--FVHSVTLLEMKDM----QFGDAADVKA----------------------------MVI

LNDTSIKYLV--FRNCII---------TDQSASRLLE-----------------------

------------------------------------------------------------

IFQTYRNITDFILDDSTLY-----------------------------GTGTASPV----

------------------------------------------------------------

-----------IGDDPFS-VTT---A------IINKLHIPK--FF---LFSDLQGI----

----YKLASN-----------------------------FKNIA---------------C

IDSKV-FLMPC-----------------------------------AFSRSFSSL-----

---QYLDLSGN---------LLSDNLL------------AS-----SACAFEGGGAWPIL

--------------QTLNV-SRNLL-----------------------------------

---------TSLRRLAQI------------------------------------------

-----------------------TSGLKYL-TN----LDVSQN----------NFGELS-

------------------------TSTCQ-------------------------------

------------------------------------------------------------

------------------------------------------------------------

-------------------------WPKSL---KYLNIS---SSQMS-------------

----NI--------------------------TCIPPTLQI-LDVSS----NYLT-----

-----VFTIKMPNLTELYISNN--RLSKLPEGMYFPR-------LFMLSIDRN-KLNDFY

Q-SDLDLF------------------------------PQLT-TLDGRD----NNYLCS-

--CQ--------------------------------------------------F--LSF

MH-----SHTIA-------------LVGW-------PDDYIC---DSPS----SV---RG

KRIQD-----------------ANL-P-PLVC------HK--------------------

----------TLIVTLSCILLIALVAAIAALCHF---LHVVW-YAKMTWA----------

-----WLKA--KRKPLKNCDRE-------------I--CYNGFVSYSE---R--DSEW-V

ENMM--VPKLE------NAVP----PM----KLCLH-KRDFVPGKW-IIDNIID-AMEKS

YKTVFVLSEH---FVRSEWC-KYELEFSHF--RLFDENN---DS----AILILLEPIE-N

ETVPKRFCKLRKLMNTKTYLEWP-----TDEE--QQEVFWDNLKTALQ------------

---------------------------------------------------------SEY

------------------------------------------------------------

------------------------------------------------------

>TLR2.2_Xetr

------------------------------------------------------------

------------------------------------------------------------

-------MYIYSICIIYLLA----------------------------------------

------------------------------------IAELSKGNTDCPCDAAHFCHCS--

---------SMHWEAIP-----------SGLPRDVRGLNLSS------------------

------------------NAIQIVTET-DLKPY-D----QLQTLLLQYNAI---------

------HTINDGSFQP----LGNLEELDL---SYNNLVL-------------LSSVW---

-------FRNLNKLKHLNLLG--------------------------NQYI---TLG---

--------NDS--LFAS-LSSLSS---------------------LQFGNQN-FDAIQKQ

------------------------------------------------------SFEGLE

R--------------LNTLEINAAQL------TL--Y--EEGSLGRMKQIYHTILRVN--

------------------------------------------------------------

---------------LTLLPALLKD-----------------------------------

--LVHSVTLLEITDT----EFST------------------------------------P

ADMQAFEVLA--YTSVKQ---------FIFRNCTIVD-----------------------

------------------------------------------------------------

-------------------------------------VSGYRFVEIIQTYRNITDV----

----------VMKNC---------------------------------------------

--RLLGRG---LDCPNLK-RTT---S------SVTTIIINNIEVSLYYIFSDLSSL----

----HQIIPQ-----------------------------FTYVT---------------V

TDSPV-YLIPCQ----------------------------------FSQSFT-SL-----

---QYLDVSGG---------KLDNTYL------------GS-----SVCYHEGGGACPKL

--------------QTLNV-SRNLL-----------------------------------

---------TSVPKVAQF------------------------------------------

-----------------------LSGLNYL-TN----LDISQN----------KFSELS-

------------------------TSKCQ-------------------------------

------------------------------------------------------------

------------------------------------------------------------

-------------------------WPKNL---KYLNIS---NNQIN-------------

----II---------------------T----TCIPPTLQI-LDVSS----NYLT---VF

A------IEM-PNLTELYISNN--RLSKLPEGM-YFT------SLVLLSISRN-DLNGFG

Q-SDLELF------------------------------SNRT-ILDARA----NNYKCS-

--CQ--------------------------------------------------F--LDF

IR-----SNTAV-------------LVGW-------PDNYKC---ASPA----SV---KD

VQIQD-----------------ANL-P-PLVC------HK--------------------

----------TLIVTLSCILLIALVAAIVALCHF---LHVVW-YAKMTWA----------

-----WLKA--KRKPLKNCDRE-------------I--CFDGFVSYSE---R--DSEW-V

ENMM--VPKLE------NATL----AM----KLCLH-KRDFVPGKW-IIDNIID-AMEKS

YKTVFVLSEH---FVRSEWC-KYELEFSHF--RLFDENN---DS----AILILLDPIE-K

ETVPKRFCKLRKLMNTKTYLEWP-----TDEE--QQEVFWDNLKTALQ------------

------------------------------------------------------------

PADYIN------------------------------------------------------

------------------------------------------------------

>TLR2_Hosa

------------------------------------------------------------

------------------------------------------------------------

---------MPHTLWMVW------------------------------------------

------------------------------------VLGVIISLSKEESSNQASLSCD-R

N--GICKGSSGSLNSIP-----------SGLTEAVKSLDLSN------------------

------------------NRITYISNS-DLQRC-V----NLQALVLTSNGI---------

------NTIEEDSFSS----LGSLEHLDL---SYNYLSN-------------LSSSW---

-------FKPLSSLTFLNLLG--------------------------NPYK---TLG---

--------ETS--LFSH-LTKLQI---------------------LRVGNMDTFTKIQRK

------------------------------------------------------DFAGLT

F--------------LEELEIDASDL------QS--Y--EPKSLKSIQNVSHLILHMK--

------------------------------------------------------------

----------------QHILLLEIFVD------------------------------VTS

SVECLELRDTDLDTF----HFSELSTGET-----------------NSLIKKFTFRNVKI

TDESLFQVMK--LLNQISGL-------LELEFDDCTL-----------------------

------------------------------------------------------------

-----------------------------------------------NGVGNFRAS----

----------DNDRV---------------------------------------------

-----------IDPGKVE--TL---T-------IRRLHIPR--FY---LFYDLSTL----

----YSLTER-----------------------------VKRIT---------------V

ENSKV-FLVPCL----------------------------------LSQHLK-SL-----

---EYLDLSEN---------LMVEEYL------------KN---------SACEDAWPSL

--------------QTLIL-RQNHL-----------------------------------

---------ASLEKTGET------------------------------------------

-----------------------LLTLKNL-TN----IDISKN----------SFHSMP-

-------------------------ETCQ-------------------------------

------------------------------------------------------------

------------------------------------------------------------

-------------------------WPEKM---KYLNLS---STRIH-------------

----SV---------------------T----GCIPKTLEI-LDVSN----NNLN---LF

S------LNL-PQLKELYISRN--KLMTLPDAS-LLP------MLLVLKISRN-AITTFS

K-EQLD-S-----F------------------------HTLK-TLEAGG----NNFICS-

--CEFLS-----------------------------------------------F--TQE

QQ-----ALAKV-------------LIDW-------PANYLC---DSPS----HV---RG

QQVQD-----------------VRL-S-VSEC------HR--------------------

----------TALVSGMCCALFLLILLTGVLCHR---FHGLW-YMKMMWA----------

-----WLQA--KRKPRKAPSRN-------------I--CYDAFVSYSE---R--DAYW-V

ENLM--VQELE------NFNP----PF----KLCLH-KRDFIPGKW-IIDNIID-SIEKS

HKTVFVLSEN---FVKSEWC-KYELDFSHF--RLFDENN---DA----AILILLEPIE-K

KAIPQRFCKLRKIMNTKTYLEWP-----MDEA--QREGFWVNLRAAIK------------

------------------------------------------------------S-----

------------------------------------------------------------

------------------------------------------------------

>TLR2_Mumu

------------------------------------------------------------

------------------------------------------------------------

---------MLRALWLFWIL----------------------------------------

------------------------------------VAITVLFSKRCSAQESLSCDAS--

---GVCDGRSRSFTSIPSGLTA-----------AMKSLDLSF------------------

------------------NKITYIGHG-DLRAC-A----NLQVLMLKSSRI---------

------NTIEGDAFYS----LGSLEHLDL---SDNHLSS-------------LSSSW---

-------FGPLSSLKYLNLMG--------------------------NPYQ---TLG---

--------VTS--LFPN-LTNLQT---------------------LRIGNVETFSEIRRI

------------------------------------------------------DFAGLT

S--------------LNELEIKALSL------RN--Y--QSQSLKSIRDIHHLTLHLS--

------------------------------------------------------------

---ESAFLLEIFADILSSVRYLELR-----------------------------------

--------DTNLARF----QFSPLPVDEV------------SSPMKKLAFRGS------V

LTDESFNELL--KLLRYI---------LELSEVEFDD-----------------------

------------------------------------------------------------

--CTLNGLGDFNPSESDVV----------------------------SELGKVETV----

------------------------------------------------------------

--------------------------------TIRRLHIPQ--FY---LFYDLSTV----

----YSLLEK-----------------------------VKRIT---------------V

ENSKV-FLVPCS----------------------------------FSQHLK-SL-----

---EFLDLSEN---------LMVEEYL------------KN---------SACKGAWPSL

--------------QTLVL-SQNHL-----------------------------------

---------RSMQKTGEI------------------------------------------

-----------------------LLTLKNL-TS----LDISRN----------TFHPMP-

-------------------------DSCQ-------------------------------

------------------------------------------------------------

------------------------------------------------------------

-------------------------WPEKM---RFLNLS---STGIR-------------

----VV---------------------K----TCIPQTLEV-LDVSN----NNLD-----

-----SFSLFLPRLQELYISRN--KLKTLPDASLFPV-------LLVMKIREN-AVSTFS

K-DQLG-S-----F------------------------PKLE-TLEAGD----NHFVCS-

--CELLS-----------------------------------------------FTMETP

AL-------AQI-------------LVDW-------PDSYLC---DSPP----RL---HG

HRLQD-----------------ARP-S-VLEC------HQ--------------------

----------AALVSGVCCALLLLILLVGALCHH---FHGLW-YLRMMWA----------

-----WLQA--KRKPKKAPCRD-------------V--CYDAFVSYSE---Q--DSHW-V

ENLM--VQQLE------NSDP----PF----KLCLH-KRDFVPGKW-IIDNIID-SIEKS

HKTVFVLSEN---FVRSEWC-KYELDFSHF--RLFDENN---DA----AILVLLEPIE-R

KAIPQRFCKLRKIMNTKTYLEWP-----LDEG--QQEVFWVNLRTAIK------------

------------------------------------------------------S-----

------------------------------------------------------------

------------------------------------------------------

>TLR2.1_Chpi

------------------------------------------------------------

-------------------------------MSNQLWKG---------------------

---------WVIYMVITANL----------------------------------------

------------------------------------SEEKTTMQMCPSCDATHFCDCS--

---------SMNLSTIPSGLTT-----------DIMGLNLSY------------------

------------------NSIKYVRETDLLVGV------NLRVLLLQFNQI---------

------WTIDKESFIF----LGKLEHLDL---SNNKLTH-------------LSPIW---

-------FRHLFSLQHLNIQG--------------------------NLYT---TLG---

--------ENP--LFSN-LKNLRY---------------------LHLGNNNSFSAIRKQ

------------------------------------------------------DFDGIT

V--------------LEQLEIDGQRL-------------RQYESGSLITVNINHIIIN--

------------------------------------------------------------

---------------INDVQVLSVMVE---------------------------------

-DFIHSVICLELRHI----AFNTANESSL------------------LEPMSHSVMEKFV

LKNVLFTDAS-----------------IDKMLNILVH-----------------------

------------------------------------------------------------

----AEQLLELELDNSILQ----------------------------GTGHWHEPI----

------------------------------------------------------------

--KIKR-----RSPMEVV--------------TIQRLTIEK--FY---LFSDLSSL----

----KNLVGN-----------------------------ITKIT---------------V

VNTKV-FLVPCN----------------------------------ISKHFS-SL-----

---LYLDLSEN---------LLADPNL------------EH---------SSCDGAWPLL

--------------QTFNL-SQNTL-----------------------------------

---------GDLQMTARS------------------------------------------

-----------------------LSHLKHL-TH----LDISRN----------NFGEIP-

-------------------------ESCQ-------------------------------

------------------------------------------------------------

------------------------------------------------------------

-------------------------WPENL---KYLNIS---GTQIP-------------

----KL---------------------T----TCIPQTLEV-LDVSS----NNLD---DF

K------LKL-PRLKELYVSKN--KLKTLPDAP-FIP------NLIALRISRN-KLTSFS

K-EEFG-S-----F------------------------RKME-TLDAGD----NNFICS-

--CEFLS-----------------------------------------------F--IQY

QE-----GIANV-------------LANW-------PENYIC---DSPS----SV---RG

QQVKA-----------------ARL-S-LFEC------HR--------------------

----------TLAVSLICILVVLVILLTVILGYK---LHVIW-YMKMTWA----------

-----WLQA--KRKPKKSHNHD-------------F--CYDAFVSYSE---R--DSEW-V

ENLM--VQELE------NAIP----PF----KLCLH-KRDFVPGKW-IIDNIID-SIEKS

HKTLFVLSEH---FVQSEWC-KYELEFSHF--RLFDEHN---DA----AILILLEPIQ-E

ETIPKRFCKLRKIMNTKTYLEWP-----LDEG--QQQIFWFNLKIALK------------

------------------------------------------------------Y-----

------------------------------------------------------------

------------------------------------------------------

>TLR2.2_Chpi

------------------------------------------------------------

------------------------MLNQRKPNTKQIWRV---------------------

---------WIFYMAISTNR----------------------------------------

------------------------------------SEEKATTQVCPSCDATRFCDCS-S

M----------DLSTIPSGLTT-----------DVMGLNLSY------------------

------------------NSIDHVRETDLKLGL------NLRVLQLQSNQI---------

------RTIDKESFIF----LGKLEHLDL---SNNKLTH-------------LSPIW---

-------FRHLFSLQQLNIQG--------------------------NLYT---TLG---

--------ENP--LFSN-LKNLRY---------------------LHLGNNNSFSAIRKQ

------------------------------------------------------DFDGIT

I--------------LEQLEIDGQRL------RQ--Y--ESGSLTTVNSINHIIININ--

------------------------------------------------------------

-----------------DVQVLSVMLE---------------------------------

-DLINSAICVELRNI----AFNTANESSLLE----------------------------P

MSHSVMEKLV--LKNVLF---------TDASVIRVLN-----------------------

------------------------------------------------------------

ILRHAKQVLELEVDDSVLQ----GTG---------------------KWHGQIE------

------------------------------------------------------------

-----------VNGESAI--EV---I------TVQRLAIEQ--FY---LFSELSGV----

----ENLLGN-----------------------------ITKTT---------------V

INTNV-FLVPCN----------------------------------ISKHFS-SL-----

---LYLDLSEN---------LLADPTL------------EH---------SSCDGAWPLL

--------------QTFNL-SQNSL-----------------------------------

---------DDLEMTGRS------------------------------------------

-----------------------LSHLKHL-TH----LDISRN----------NFGEIP-

-------------------------ESCQ-------------------------------

------------------------------------------------------------

------------------------------------------------------------

-------------------------WPENL---KYLNIS---STQIP-------------

----KL---------------------T----TCIPQTLEV-LDVSS----NNLD---DF

R------LKL-PRLKELYVSKN--KLKTLPNAP-FIP------NLIALRISRN-KLTSFS

K-EEFG-S-----F------------------------RKME-TLDAGD----NNFICS-

--CEFLS-----------------------------------------------F--IQY

QE-----GIANV-------------LANW-------PENYIC---DSPS----SV---RG

QQVKA-----------------ARL-S-LFEC------HR--------------------

----------TLAVSLICILVVLVILLIVILGYK---LHVIW-YMKMTWA----------

-----WLQA--KRKPKKSHNHD-------------F--CYDAFVSYSE---R--DSEW-V

ENLM--VQELE------NAIP----PF----KLCLH-KRDFVPGKW-ITDNIID-SIEKS

HKTVFVLSEH---FVQSEWC-KYELEFSHF--RLFDEHN---DA----AILILLEPIQ-E

QTIPKRFCKLRKIMNTKTYLEWP-----LDEN--QQQIFWNNLKTALK------------

----------------------------------------------------------SY

DVI---------------------------------------------------------

------------------------------------------------------

>TLR2A_Gaga

------------------------------------------------------------

----------------------MFNQSKQKPTMKLMWQA---------------------

---------WLIYTALAAHL----------------------------------------

------------------------------------PEEQALRQACLSCDATQSCNCS--

---------FMGLDFIP-----------PGLTGKITVLNLAH------------------

------------------NRIKLIRTHDLQKAV------NLRTLLLQSNQI---------

------SSIDEDSFGS----QGKLELLDL---SNNSLAH-------------LSPVW---

-------FGPLFSLQHLRIQG--------------------------NSYS---DLG---

--------ESS--PFSS-LRNLSS---------------------LHLGNPQ-FSIIRQG

------------------------------------------------------NFEGIV

F--------------LNTLRIDGDNL------SQ--Y--EPGSLKSIRKINHMIISIR--

-------------------------------------------------------RIDVF

SAVIRDLLHSAIWLEVREIKLDIEN-----------------------------------

----EKLVQNSTLPL----TIQKLTFTGA------------------------------S

FTDKYISQIA--VLLKEI---------RSLRELEAID-----------------------

------------------------------------------------------------

-----------------------------------------------CVLEGKGAW----

------------------------------------------------------------

--DMTE-----IARSKQS--------------SIETLSITN-------MTILDFYL----

----FFDLEG----------------------IETQVGKLKRLS---------------I

ASSKV-FMVPCR----------------------------------LARYFS-SL-----

---LYLDFHDN---------LLVNNRL------------GE---------TICEDAWPSL

--------------QTLNL-SKNSL-----------------------------------

---------KSLKQAARY------------------------------------------

-----------------------ISNLHKL-IN----LDISEN----------NFGEIP-

-------------------------DMCE-------------------------------

------------------------------------------------------------

------------------------------------------------------------

-------------------------WPENL---KYLNLS---STQIP-------------

----KL---------------------T----TCIPSTLEV-LDVSA----NNLQ---DF

G------LQL-PFLKELYLTKN--HLKTLPEATDIPN-------LVAMSISRN-KLNSFS

K-EEFE-S-----F------------------------KQME-LLDASA----NNFICS-

--CEFLS-----------------------------------------------F--IHH

EA-----GIAQV-------------LVGW-------PESYIC---DSPL----TV---RG

AQVGS-----------------VQL-S-LMEC------HR--------------------

----------SLLVSLICTLVFLFILILVVVGYK---YHAVW-YMRMTWA----------

-----WLQA--KRKPKRAPTKD-------------I--CYDAFVSYSE---N--DSNW-V

ENIM--VQQLE------QACP----PF----RLCLH-KRDFVPGKW-IVDNIID-SIEKS

HKTLFVLSEH---FVQSEWC-KYELDFSHF--RLFDENN---DV----AILILLEPIQ-S

QAIP-RFCKLRKIMNTKTYLEWP-----PDEE--QQQMFWENLKAALK------------

------------------------------------------------------S-----

------------------------------------------------------------

------------------------------------------------------

>TLR2B_Gaga

------------------------------------------------------------

---------------------------------MHTWKM---------------------

---------WAICTALAAYL----------------------------------------

------------------------------------PEEQALRQACLSCDATQSCNCS--

---------FMGLDFIP-----------PGLTGKITVLNLAH------------------

------------------NRIKVIRTHDLQKAV------NLRTLLLQSNQI---------

------SSIDEDSFGS----QGKLELLDL---SNNSLAH-------------LSPVW---

-------FGPLFSLQHLRIQG--------------------------NSYSD--------

--------LGESSPFSS-LRNLSS---------------------LHLGNPQ-FSIIRQG

------------------------------------------------------NFEGIV

F--------------LNTLRIDGDNL----------SQYEPGSLKSIRKINHMIISIR--

-------------------------------------------------------RIDVF

SAVIRDLLHSAIWLDVRKLAFSVPE-----------------------------------

-----KIQLLRIMSS----------------------SFAKKISLKQCLFTDATVPEIVS

ILEGMPKLME-----------------VEMKDCTLLG-----------------------

------------------------------------------------------------

---------------------------------------------TGKWYKQIHA-----

------------------------------------------------------------

-----------NQSQSLR--IL----------TIENLSIEE--FY---LFTDLQSV----

----LDLLSL-----------------------------FRKVT---------------V

ENTKV-FLVPCK----------------------------------LSQHLL-SL-----

---EYLDLSAN---------LLGDQSL------------EH---------SACQGAWPSL

--------------QTLNL-SQNSL-----------------------------------

---------SDLKMTGKS------------------------------------------

-----------------------LFHLRNL-NL----LDISEN----------NFGEIP-

-------------------------DVCE-------------------------------

------------------------------------------------------------

------------------------------------------------------------

-------------------------WPENL---KYLNLS---STQIP-------------

----KL---------------------T----TCIPSTLEV-LDVSA----NNLQ---DF

G------LQL-PFLKELYLTKN--HLKTLPEATDIPN-------LVAMSISRN-KLNSFS

K------E-----------------------------------ELTVRG-----------

--AQ--------------------------------------------------------

----------------------------------------------------------VG

SVQLS-----------------------LMEC----------------------------

---------HRSLVSLICTLVFLFILILVVVGYK---YHAVW-YMRMTWA----------

-----WLQA--KRKPKRAPTKD-------------I--CYDAFVSYSE---N--DSNW-V

ENIM--VQQLE------QACP----PF----RLCLH-KRDFVPGKW-IVDNIID-SIEKS

HKTLFVLSEH---FVQSEWC-KYELDFSHF--RLFDENN---DV----AILILLEPIQ-S

QAIPKRFCKLRKIMNTKTYLEWP-----PDEE--QQQMFWENLKAALK------------

------------------------------------------------------S-----

------------------------------------------------------------

------------------------------------------------------

>Pema_putative_TLR1_2.2

------------------------------------------------------------

------------------------------------------------------------

---------MAGAAWSHGTS----------------------------------------

------------------------------------IFNGCQIRGVR-------ADCS-R

L--RL--------QSVP--EYL------PV---TITSLDLSY-N----------EIS---

------------------S----IQRE-HLSAL-L----QLKSLNVAFNQI---------

------SSIDPWVFLN----NSALEYIDL---SNNRLPG-------------AWDDS---

-------FAVLKSVRSLDVRN--------------------------NSYN---SLH---

--------IPG--SFTS-LKTLYH---------------------LKVGGAN-VTEMYQ-

------------------------------------------------------SVSKTM

E--------------LEHLSIVTGDL------TI--Y--TRGSLSAFHSLKSVTLALT--

------------------------------------------------------------

------------------------------------------------------------

-----MHTNIELLTD----IICDVSCN-----------------SVELQLEFLNF-TDYS

RDVNPFQCIS----KPQSMVEV-----LTISHTCVED-----------------------

------------------------------------------------------------

-----------SAAKFLVN----EV----------------------IESTKLRCL----

----------NLR-----------------------------------------------

--HIEY-----EGYGYFA--LK---DSLKSAPNVRHIFIEN--FN---IKVFNSFY----

----ALLNLK---------------P------FLIN---VQSLV---------------L

NFVHL-YYFPCE----------------------------------VLSGLA-SI-----

---VTLDISGN---------LLTEYTS------------FL---------TCSNVRLPYL

--------------MELIF-RKNHL-----------------------------------

---------THIQNVGAM------------------------------------------

-----------------------LISCPSL-KL----IDVSEN----------QIVSEW-

------------------------SSNCK-------------------------------

------------------------------------------------------------

------------------------------------------------------------

-------------------------WPSNL---QVFNIS---SNLLT-------------

----DD----VFDC--------------------LPTSLQS-LDVSN----NKIQ---NV

N--NKLMKF--KNLKELHLSNN--KINTIPVEL-LQA--L-P-WLKVLTIAGN-ILWTVE

P-SVLH-H-----L------------------------GNLT-LLDMRG----NPFYCT-

--CNIRH-----------------------------------------------F--VTF

CE----NSSPLR-------------IEGW-------PAEYHC---SNPE----NE---VG

KQLSS-----------------VSY-P-TLYC------DT--------------------

----------TMKSVIACVTTFVCTALLVGLCWY---LDALW-YVRMTWA---------W

LQA--KRRN----FLADPDENS----------------VYDAFVSYSQ---Q--DAAW-V

MQQL--MPELE-----SHSVP----PF----RLCVH-ERDFIPGRH-IMDNIID-CIELS

RKTLFIISQS---FVESEWC-HYELYFAQQ--RLIESRD---DA----LVLVLLEPIP-H

NSVPSRFCRLRCLMERKTYLEWP-----AHRG--KQALFWANLRATLG------------

---------------------------------------------------------RQK

QQEPKNEAIALAPP----------------------------------------------

------------------------------------------------------

>Pema_putative_TLR1_2.1

------------------------------------------------------------

------------------------------------------------------------

---------MSVALLVTIILMTRA------------------------------------

------------------------------------AWSHGAPISSGCLIRGDKADCS-R

L----------GLHYMPKELPV-----------TIASLDMSY------------------

------------------NQISSIQRE-HLSAL-L----RLKTLNVAFNQI---------

------SSIDPLAFSN----NFALQDVDL---SNNRLPG-------------AWDDS---

-------FAVLKCVRSLDVRN--------------------------NSYI---SFNI--

--------PGS---FRS-LKALHH---------------------LKVGGLN-VTEIHQ-

------------------------------------------------------NVSETM

K--------------LEHLSIMTGDL------TI--Y--TPGSLSAFRSLQSVTLALT--

------------------------------------------------------------

-----------LRTNIALLTDIICD-----------------------------------

-------VSGNSVEL----HVQFVDFSEYRQNVDVFKCISNSGTIEVFNISHS------Q

VTDNTITGML-----------------NNIMKSQRII-----------------------

------------------------------------------------------------

---------SFNLRQIGFD----GI-------------GDWQVPLLPSPVISLQSF----

------------------------------------------------------------

--LVDG-----IANPNFF--GF---S------TMTNLHFFL--YY---------------

---------------------------------------LKYLT---------------I

RNSNLFYMPC------------------------------------KITFLCQNL-----

---HFLDLSNN---------LLTENTA------------FA---------QCQNIQLKNL

--------------NTLRL-HKNYM-----------------------------------

---------SSLSAVGAM------------------------------------------

-----------------------VNNITSL-TL----LDISAN----------QIHSDQ-

------------------------KSGCM-------------------------------

------------------------------------------------------------

------------------------------------------------------------

-------------------------WPMNL---QVLNAS---SNFLT-------------

----DE---------------------T----FDCLPTSLYSIDVSN----NRIQ---KV

S--KRLLEF--KHLHELYLSKN--KLNFFPVEL-AHG----IPSLKVLTLANN-TLLGMK

L-TDLD-S-----L------------------------GNLT-LLDMRG----NPFYCT-

--CNILH-----------------------------------------------F--VTF

CE----NSSPLR-------------IEGW-------PADYQC---SNPE----KE---VG

KQLSS-----------------VSF-P-ILYC------DT--------------------

----------TLKIVIACVTTFVCTALLVGLCWY---LDALW-YLRMTWA---------W

LQA--KRRS----FLSDPDGNT----------------AYDAFVSYSQ---H--DAVW-V

MEKL--MPELE-----SHSVP----PF----RLCVH-ERDFVPGRF-IMDNIID-CIEQS

RKTLFVLSRS---FVESEWC-HYELYFAQQ--RLMESRD---DA----LVLVLLEPIP-R

DSVPSRFCRLRRLMGRKTYLEWP-----AEQG--KQVLFWANLRATLG------------

------------------------------------------------------E-----

------------------------------------------------------------

------------------------------------------------------

>Pema_putative_TLR1_2.3

------------------------------------------------------------

------------------------------------------------------------

------------------------------------------------------------

------------------------------------------------------------

----------------------------------MSYNQISS------------------

-----------------------IQRE-HLSAL-S----HLKSLNVAFNQI---------

------LSIDSLAFSN----NSALEDIDL---SNNRLPG-------------AWDDS---

-------FALLKSVRYLDVSN--------------------------NSYI---YLD---

--------IPGSFTLLKTLHHLKV---------------------GGLNVTKIQQSISET

ME----------------------------------------------------------

---------------LEHLSIVTGDL----------SIYTPGSLSAFRSLQSVTLALI--

------------------------------------------------------------

-----------LRTNISLLTGIICDVS---------------------------------

---------GNSVEL----QLDFLDFTNY------------------------------S

GDVNPFECVS--KQHSI----------LEIFNVSHLQ-----------------------

-----------------------------------------------------------V

TIPTIVFLFTSIIRSNKLV-----------------------------------------

----------AVNMR---------------------------------------------

--HIYFNGLSAFPWKILPSANI----------SVRHLLIEGIYNPNFHGIFYVKNI----

----NALSSN-----------------------------LESLV---------------V

TDASL-ISLGCD----------------------------------IVNNFG-NL-----

---YLLDISNN---------KLNENSV------------CG---------KIHSANLDKL

--------------NKLIL-RKNIF-----------------------------------

---------TSLDTVGAM------------------------------------------

-----------------------LKNLLSL-TS----LDVSEN----------RLRKNG-

------------------------ESGCE-------------------------------

------------------------------------------------------------

------------------------------------------------------------

-------------------------WPLSL---KFLNVS---RNFLK-------------

------------------------GNAF----NCLPTSLHS-LDVSN----NKIQ---NI

D---FKLMKF-EKLKELYLSNN--KLSTFPVEL-PKA----LPWLKVLTIAGN-TLLTMD

P-SVLH-G-----L------------------------ANFT-LLDMRG----NPFYCT-

--CDIRH-----------------------------------------------F--ITF

CE----NSSPLR-------------IEGW-------PADYQC---SNPE----DA---IG

KQLSS-----------------LSF-P-TLYC------HT--------------------

----------TLKIVIACVTTFVCTALLVGLCWY---LDALW-YVRMMWA---------W

LQA--KRRN----FLSDSDENA----------------VYDAFVSYSQ---Y--DAIW-V

MEKL--LPELE-----SHSVP----PF----RLCVH-ERDFVPGRV-IMDNIID-CIEQS

RKTLFVISRS---FVESEWC-HYELYFAQQ--RLMESRD---GA----LVLVLLEPIP-R

DSVPSRFCRLRRLMARKTYLEWP-----VEQS--KQALFWANLRATLS------------

----------------------C--------------------QRE--------Q--EPQ

NLEIVV------------------------------------------------------

------------------------------------------------------

>Pema_putative_TLR14_18.2

------------------------------------------------------------

------------------------------------------------------------

---------MAGWPGMFIAA----------------------------------------

------------------------------------AVLLCLMHPGPWVRGEAFHQCS-V

V-GDVADCSRRGLTAVP--ARL------PP---SIAQLDLSH-N----------RIE---

------------------S----LSAN-DFSHV-P----LLRVLNLAFNCI---------

------RDIHPGALAH----TALLQHLDL---YHNELLE-------------IPAEA---

-------VGNLRLLQVLNISM--------------------------NNYT---SFS---

--------LGG--AFAN-LHSLRS---------------------LTIGTTR-TDVLNTS

------------------------------------------------------DFMALQ

N--------------VSVTHLNVHTG-SPL--IK--F--EPGVLAPFKMLQSFRMNFT--

------------------------------------------------------------

------------------------------------------------------------

--VDDDPVIFSKVLL----DLNKTKVSEF------------------------------Q

TDRVLLNPVK--NTSIDLF--------YGLEKCSLLR-----------------------

------------------------------------------------------------

----NFTLVAANLTDHEIT----SL-------------LKNIYLSQITSVEITNSS----

----------YTDKN---------------------------------------------

--VVVF-----FNVENVT--KL---S------PLEKVTINQ--IF---HLNMTYPK----

----FAINFT----------------------LFPS---FSKLK---------------I

SHTGMNKVEC------------------------------------FFMKLK-FI-----

---TWLDFSSN---------LLDEEGL------------WW-------TNCKYTIILPRA

--------------TELYI-SNNKF-----------------------------------

---------TDLQIISSM------------------------------------------

-----------------------VSLMPSI-KL----LDVGYN----------YITDID-

--------------------------DCS-------------------------------

------------------------------------------------------------

------------------------------------------------------------

-------------------------WPPTL---ETLILR---NNDIS-------------

----KD--------------------SR----ICTSPQLKV-LDLSY----TRME---SV

P--YYILDDA-KSLRELYLTGN--NIHYIQPEI-QSS------SLQVLHINYN-TLGIIT

K-GTFQ-L-----L------------------------PKIR-ALKLGN----NLFYCM-

--CDLY--------------------------------------------W---F--RQT

FD--------KSL------------LVDW-------PKDYVC---SYPE----NL---AE

KTMDC-----------------FNP-S-IVSC------DK--------------------

----------RITIGLSVVITAMVVALVLGLGYY---CDALW-YIKMGWI---------W

VGA--KRRG----YNRVTSGEA-PP----------F--EYNAFISYSH---M--DSDW-V

EGTL--VPKLE------RSGS----NL----KLCMH-ERDFTPGEW-VVDNIIR-CIEGS

SKTLFVLSTN---FVKSEWC-HYELYFAQH--RMLEQRQ---DS----LVLVLLESLP-K

SSLPNKFCRLRRLLNRKTYLEWP-----AEES--KRAIFWASLQAILQ------------

---------------------------------------------------------TTS

NPTNPVT-----------------------------------------------------

------------------------------------------------------

>Pema_putative_TLR14_18.1

------------------------------------------------------------

-------------------------------MLMCTPVI---------------------

---------LCLMGFIKASL----------------------------------------

------------------------------------REMNDPASHCHIKDEGRTADCS-H

R--GL--------THVP--RGF------SA---DITRLDLSY-N----------NIK---

------------------A----LEVG-DFSST-P----NLQVLTLAFNHI---------

------QKIHPQAMAT----LKRLYYLDL---CQNNLSE-------------KPGEA---

-------LGSLPNLQVLNISM--------------------------NNYT---SFA---

--------LGV--DFSR-MSNLRD---------------------LTIGTSK-TTTLNAS

DF----------------------------------------------------QALESV

P--------------LKYLNLNTGSP-L----WV--Y--ETGALTHLRSLEKLNTNVS--

------------------------------------------------------------

---VDKDP-LVLSKMLTDLNNTNIS-----------------------------------

-----ELEIFRILNN----PLK-------------------------------------N

ASIDFFKGLG--SSNLLKN--------MTLIEANFTD-----------------------

------------------------------------------------------------

--------------------------------------KEVSNLLKNIYLSELTML----

----------VFKNS---------------------------------------------

--SYTD-----HYPVIFN--GV---KNVTKRSSLKKIVIDS--IF---HLNMTYPI----

----FIVNMT----------------------LFPN---ISQLK---------------I

SNTGMNKVDC------------------------------------FFMKMK-AI-----

---KQLDFSRN---------LLNEGGL------------WW-------DSCNYTVILPEA

--------------TELII-SHNYF-----------------------------------

---------KDLKKISEM------------------------------------------

-----------------------VSLMPRI-NS----LDVSYN----------GINYIE-

--------------------------ECR-------------------------------

------------------------------------------------------------

------------------------------------------------------------

-------------------------WPSSL---ERLILR---NNEIS-------------

----KE--------------------SV----ICTSANLKF-LDLSY----TRLE---SF

P--NYILTNA-TSLQELHLTGN--LIKYISSDV-KSQ------SLQVLYVDYN-AVGIIG

Q-GTFQ-H-----L------------------------QKIK-SITLGN----NPFYCM-

--CDLY--------------------------------------------W---F--RQR

FN--------KTL------------LNGW-------PNDYTC---SYPE----YL---SG

KEMDY-----------------FNP-N-ILNC------DR--------------------

----------IIAISVSVVVTVVVIALVIGIGHY---FDAIW-YIRMGFI---------W

VSA--KRRR----YKIISGEED-LP----------F--QYHAFISYSH---L--DSEW-V

QNTL--VPTLE------HSNP----DL----RLCIH-ERDFTPGHW-IVDNIIQ-CIEKS

SKTLFVLSRN---FVNSEWC-HYELYFAQH--RMVEENQ---DS----LVLLLLEPLP-K

GSLPSKFCKLRRLLSNKTYLEWP-----TEER--KRAVFWTSLNAVLQ------------

---------------------------------------------------------SRP

PP----------------------------------------------------------

------------------------------------------------------

>Pema_putative_TLR14_18.3

------------------------------------------------------------

------------------------------------------------------------

--------MEQLLKYAVVVF----------------------------------------

------------------------------------CTAGPWAGVGISGTKAKNCCCR-D

N-GDY---SQCDLAAVP--RAL------PA---TVTVLDLSH-N----------RIG---

------------------A----IRLG-DFSST-P----KLEVLILAFNRI---------

------RTIEAGALRD----VPLVRYLDL---YQNELPA-------------VPEEA---

-------LIDLPNLQTLNISM--------------------------NNYT---SFA---

--------LGG--AFSR-MSQLQS---------------------LSIGTSH-TSELHAS

DL----------------------------------------------------QALSSI

P--------------LKHLWLNTGSP-L----KS--Y--EQGALANLAHLEKLFTNVS--

------------------------------------------------------------

-----------IDDDLSILSKMLLDLG---------------------------------

---TTRISEITIMNI----LINQVH----------------------------------N

QSIDLFSGLA----YCPLLKNV-----MLISAN---------------------------

------------------------------------------------------------

----LTNLDIVSLLKN-------------------------------IYLSEVTSV----

----------TLTNS---------------------------------------------

--SYTD-----FDNVVFP--NL---P-TERKAALETVIIDG--IF---HMPMTYPL----

----FYIPFS----------------------LFPN---FSKFK---------------L

SNTGLNKVDC------------------------------------LFMTFPGKI-----

-----LDFSNN---------LLVEDGM------------WL-------PKCPQKDNLINT

--------------THLII-SHNRF-----------------------------------

---------SDLNTIAQK------------------------------------------

-----------------------VVVMPNI-KS----LDLSYN----------SINHIQ-

--------------------------ECS-------------------------------

------------------------------------------------------------

------------------------------------------------------------

------------------------AWPSTL---ETLILR---NNDIS-------------

----KD--------------------SK----ICISEYLQV-LDLSY----TRLE---TF

T--QNILNDG-KSLRELYLSGN--NIKYILPEL-YSP------SLQVLYVDNN-GVGIIG

E-NTFR-G-----L------------------------ENIK-TLYLGN----NPYYCF-

--CDLY--------------------------------------------W---F--QQT

FE-------KQL-------------LKNW-------PNEYRC---SYPN----EL---SN

RTLSD-----------------LNL-S-IITC------DK--------------------

----------RIIISMSVGITAFIIILILGLGYY---FDAIW-YIRMGTV---------W

ISA--KRRR----MQQGAGGP--------------F--QYHAFISYSQ---L--DSDW-V

ENTL--VPTLE------SSNP----DL----KLCIH-ERDFMPGEW-IVDNIIQ-CIEHS

NKTLFILSRN---FVNSEWC-HYELYFAQH--RVLEQRQ---DS----LVLLLLEPLP-R

NSVPSKFCRLRKLLNRKTYLEWP-----AEEG--KRSMFWASLRAVLQ------------

---------------------SD-------------------------------H-EPSN

PNSNHRSISTCNSNPIRNPYV---------------------------------------

------------------------------------------------------

>Pema_putative_TLR14_18.4

------------------------------------------------------------

--------------------------------NRNLLVF---------------------

---------ATTLLCVVQLPQA--------------------------------------

------------------------------------STQNLDNPYCSYDDHQQTADCS-D

R----------DFTAVP-----------QGLPATVTVLDLSH------------------

------------------NRIGAIRLG-DFSST-P----KLEVLILAFNRI---------

-----GTTIEAGALRD----VPLVRYLDL---YQNELPA-------------VPEGA---

-------LIDLPNLQTLNISM--------------------------NNYT---SFA---

--------LGG--AFSR-MSRLQS---------------------LSIGTSH-TSELKAA

DL----------------------------------------------------QALSNI

S--------------LTYLGLNTGS-------PL--LRYESGSLVYFRSLKTFFMNFS--

------------------------------------------------------------

---------VDGNPSIFSKVLIDLN-----------------------------------

---TSQTTQLETYQI----LIEPLKNRSF-------------------------------

---ELFHGLS--YCQSLRE--------IIIVNANFSD-----------------------

------------------------------------------------------------

--------------------------------------SEATNFIKNMYLSNLKMV----

----------VFTNS---------------------------------------------

--SYND-----KGPVVFL--GI---KGVNKTAPLKRLVIDG--VF---HLNMTYPL----

----FLINFT----------------------LFPN---LTEFK---------------I

SNTGMNKVAC------------------------------------LFLKIA-AI-----

---TWLDFSRN---------LLDETGL------------WW-------ESCNYTVILPNA

--------------EYMDI-SHNHF-----------------------------------

---------RHLKIISSM------------------------------------------

-----------------------VSLMPKI-KS----LDLSYN----------YINDIY-

--------------------------ECS-------------------------------

------------------------------------------------------------

------------------------------------------------------------

-------------------------WPNTL---QKLNLR---NNDIT-------------

----KS--------------------SK----ICTSKYLQA-LDLSY----TRLE---TF

P--YNILAEA-TSLKELYLSGN--NIRHINPQI-GSA------SLQVLYMDDN-ALGIIR

K-GTFE-N-----L------------------------QAAR-SLNLKN----NPFYCF-

--CDLY--------------------------------------------W---F--RKK

FN-------KSL-------------LMDW-------PNGYTC---SYPD----YL---AD

SPLEV-----------------FSP-S-YVAC------DR--------------------

----------NIIIILSVVITACVIGITAGLSYH---LDAVW-YIRMGWI---------W

VSA--KRRR----YNRLNDGETST-----------F--QYHAFISYSQ---L--DSDW-V

ENTL--VPTLE------SSNP----DL----KLCIH-ERDFTPGEW-IVDNIIQ-CIEHS

NKTLFILSRN---FVNSEWC-HYELYFAQH--RVLEQRQ---DS----LVLLLLEPLP-R

NSVPSKFCRLRKLLNRKTYLEWP-----AEEG--KRSMFWASLRALLQ------------

----------------------S--------------------DNR--------P--LES

SPDPDA------------------------------------------------------

------------------------------------------------------

>TLR18_Dare

------------------------------------------------------------

----------------------------------MLVPL---------------------

---------VLILSLQAVLH----------------------------------------

------------------------------------IKHAKAKQSCTIAFDKLSADCK-G

L--RL--------DSVPT-TQL------PD---SLEELDLSF-N----------TIH---

------------------V----IRKQ-DFVKL-T----YLRVLKLNFNNI---------

------SLVLDDAFQG----NLLLEELNL---FNNSLTE-------------IPFKA---

-------LEPLTNLKVLEMS---------------------------NNLY---SQA---

--------SLG-AAFLN-FNQLKV---------------------LSIGGPL-VSSLGSR

DI----------------------------------------------------YVLKNI

S--------------LDKFAVKTGT-----GFRD--Y--EPGYFKSLSTKHLWFDIAF--

------------------------------------------------------------

---------------DKNPDLLPKM-----------------------------------

---LKDLANKTFDVL----RFRNLFEFQY-Y----------------------------T

GKRDIFYGLQ--YVNAQT---------LTFYRGKFNE-----------------------

------------------------------------------------------------

------EVMRMALKNVEI-----------------------------SPIKALELL----

----------LID-----------------------------------------------

-----------FARSQNR--TQ---GSSVKNLSLDRLVLSDISNP---DIMRFDWS----

----FTWLNH-----------------------------VRKFI---------------V

WNVNF-NSVPCD----------------------------------SWPEMK-SV-----

---ELLDISNN---------QLLDSYI------------YN-------PLCDTRNTLHKL

--------------DTFNV-SHNRL-----------------------------------

---------TSLSDLASL------------------------------------------

-----------------------TKDFVKL-TT----IDMSHN----------QLQYLG-

------------------------NGACD-------------------------------

------------------------------------------------------------

------------------------------------------------------------

-------------------------WRKTI---TKVIGN---HNSLT-------------

----SD--------------------SF----KCLPTSVNF-LDLSY----SSLD---QL

D--MDYFNKA-SELTELLLSGN--KIKFIPSG--WKN----R-YLRTLALDGN-SFGLVS

M-KSFK-D-----M------------------------LSLR-VLTAGN----NPYHCT-

--CDLYT-----------------------------------------------F--IQE

TT----SKGKVT-------------ITDW-------PNNYKC---YHPE----RL---LN

TMVSK-----------------YSP-G-HLAC------DI--------------------

----------TLVIIISVSTTAAVVLVIMLLCYI---FHVPW-YVKATYQ---------I

IRA--KYRA----HKEGLGQGV-D-----------Y--AFHAFISYSH---S--DADW-V

RNHL--LPCLE------NVKP----PY----RLCIH-ERDFIPGKW-IIDNIIE-NIENS

RKVIFVLSHN---FVNSEWC-NYELYFAQQ--RAIGKTF---SD----VILVVKEPID-P

TSLPSKFCKLKRMLNTKTYLEWP-----QQPT--EQNFFWIQLRSVLG------------

----------------------K---------------------PN--------S--IRP

RTISRHSRLSSARSVSLIEAPQIQDPEGPDEEDHQNSPQPSNKCQLTCIEVA--------

------------------------------------------------------

>TLR14_Chpi

------------------------------------------------------------

-----------------------------------MRPL---------------------

---------AGMCIALCVVI----------------------------------------

------------------------------------VGLADGADPCLVTADNKVATCK-G

Q--NL--------NRVP--QHL------PR---TLLRLDLSY-N----------RLK---

------------------E----IAAG-DFSAL-T----QLQSLDLGYNNI---------

------SRISADAFAS----NVLLEELSL---FNNSLRW-------------IPSPA---

-------LKPLRKLRRLEMS---------------------------NNLY---LRS---

--------TLD-EVFST-LRNLQE---------------------FSMGGSL-IQTVGKW

DF----------------------------------------------------LPLKNI

A--------------LQKFALKTASS-L----LK--Y--QNGAFSVLNTTALWCDIAL--

------------------------------------------------------------

---------------DKNPKALPMI-----------------------------------

---LRDLRGKPLQYL----RFRNLFEFTY-Y----------------------------T

EPADLFSGLA--EVQASK---------LVFYRGKFNE-----------------------

------------------------------------------------------------

---NLLRLALLNIQKSRIR----DL-----------------------------------

----------SLM-----------------------------------------------

--SIDF-----ARSSQWN--RS---ETGITNLTLGSLLLQDISNP---DILRFDWT----

----FTWFSG-----------------------------VANLS---------------I

LNVNF-NFVPCD----------------------------------TWDEMR-NV-----

---VTLDVSNN---------RLKDAYI------------YN-------QGCNYQDVLPKL

--------------ERFLM-AKNEL-----------------------------------

---------TSLGIVAKL------------------------------------------

-----------------------TANWPRL-TH----INASHN----------HIGGLK-

------------------------ETACQ-------------------------------

------------------------------------------------------------

------------------------------------------------------------

-------------------------WNPGL---VWLALD---HNTVT-------------

----ME--------------------IF----KCLPITLHY-LDLSY----SELD---RL

E--MSYFVRC-QDLQELKLSGN--KIKFIPSE--WRC----P-SLRILAMDGN-SFGVIS

E-GSFV-N-----M------------------------PELT-SLKAGN----NPYHCT-

--CDLYG-----------------------------------------------F--LQE

TR----RKGKLT-------------LLDW-------PEGWTC---YHPE----SL---LD

MGVAS-----------------YTP-G-LTEC------DV--------------------

----------RVVVAISVSITAVVIIATMVLCWK---FDVLW-YLQATY-----------

-----RIIR--SKYRARHAQPA--------RA---Y--AYHAFISYSC---S--DADW-V

RQEL--LQRLE------SSSP----PY----RICIH-ERDFTPGKW-IIDNIIE-NIESS

YKVIFVLSRS---FVDSEWC-NYELYFAHQ--RAVGLGY---ED----VILVVKEAID-P

QSLPNKFCKLRKMLSTKTYLEWP-----SEPS--RQPFFWIQLRNVLG------------

------------------------------------------------------KPGAAE

PGHDRVSLASVELGSGEVVSSPAEEETAIDAVASPAS-----------------------

------------------------------------------------------

>TLR14.2_Xetr

------------------------------------------------------------

----------------------------MEMFRRYNEPV---------------------

---------FYCFLLLALTK----------------------------------------

------------------------------------GIWSQNSCQVDEKQKYANCQGQ-N

L------------NDVP--KDL------PV---TLEVLDLSC-N----------WIS---

------------------Q----IRVD-DFSSY-T----NLQALNLSFNNI---------

------STIDNSSFAS----NTQLRNLTL---FNNGLTE-------------MPSTL---

-------LEPLLLLEVLDLS---------------------------DNLY---NYS---

--------TLG-KVFKT-LANLRS---------------------LSIGGPF-VSKVLRG

DF----------------------------------------------------VPIKNI

S--------------LQKFELKTRSS-L----RF--Y--QTGAFSVLNTDVLVLVMTL--

------------------------------------------------------------

---------------DTNPKVLPMI-----------------------------------

---LKDLAGKSLDIL----SFGNLFEHNY-Y----------------------------A

GPTNPFSSLP--DINVRE---------LIFNRG-KVN-----------------------

------------------------------------------------------------

-----QKLLQLILETIQTS----SI----------------------QDLSLLSV-----

----------DFDYS---------------------------------------------

--DVRT-----TVDVKMD--NL---F-------LRSLVIKDATNP---DILTFDQT----

----FTWFSK-----------------------------VSNLY---------------I

INVNF-NFAQCD----------------------------------TWSQMK-NV-----

---ERLDIRNN---------LLLSSDL------------YN--------PSCKYGELPKL

--------------HTFTA-ANNNL-----------------------------------

---------QILKPISLL------------------------------------------

-----------------------TANWPKL-SS----LDLSSN----------YIGS---

-----------------------EDENCS-------------------------------

------------------------------------------------------------

------------------------------------------------------------

-------------------------WTPNI---TTFILK---NNLLT-------------

----PG--------------------VF----TCLPTTVRY-LDLSN----SRLE---SL

D--MDYFSNA-TNLKTLVLSYN--KLTSISSN--WSN----P-FLQVLFLDNN-IISIID

K-GSFN-N-----L------------------------PQLR-TLTAGN----NPYHCT-

--CDLYS-----------------------------------------------F--FSD

VL----GKNKIT-------------ISDW-------PQSYCC---YHPQ----QL---RN

TRVDI-----------------YTP-G-SIEC------NV--------------------

----------GLLVAITVSTTAVVVIACMIMCWR---LDAPW-YFRMMCH---------I

VKS--KYRS-----KKANDSRE-------------Y--NYHAFISYSY---S--DADW-V

RGEL--LYRLE------SCSP----PY----RVCIH-ERDFLPGRW-IIDNIID-NIETS

RKTIFVLSHN---FVNSEWC-NYELYFAHQ--RAIGHSF---ED----VILVVKENVT-L

KDLPKRFHKLRKMLRKKTYLEWP-----SEPS--KQHFFWIQLKNILG------------

------------------------------------------------------S-PSTA

GALEPCPASEVPSITYSKMNLPGS------------------------------------

------------------------------------------------------

>TLR14.4_Xetr

------------------------------------------------------------

--------------QVPFLQNLFFPNRCEDPGRVSAQQK---------------------

---------VHVCFLLLNLL----------------------------------------

------------------------------------KWTSCQNPCQRDSTNRY-ANCQ-G

Q--DL--------VEVP--QDL------PV---TLQSLDLSY-N----------RLF---

------------------H----IRYE-DFSSY-T----NLRALNLSFNNI---------

------STIECGSFAS----NTLLRNLTL---FNNSLTE-------------MPSTL---

-------FEPLLLLEFLDVS---------------------------NNLY---NYS---

--------TLG-KVFEN-LVNLQN---------------------LAIGGPL-VSKVLKD

DF----------------------------------------------------APIKNR

S--------------LKKFSLKTMS-----KLGF--Y--EPGALEVLNTRVLWLDISL--

------------------------------------------------------------

---------------DTNAQALPLI-----------------------------------

---LKDLAGKTIDSL----RFRRLFESSY-Y----------------------------T

DTMDLFCGLV--DINIRE---------LIFFRGMFTE-----------------------

------------------------------------------------------------

------NLLHQALHSIQKS----TI-------------QDLLL----LSVKFDRSL----

----------NTN-----------------------------------------------

-----------NTHILFD--RL---Y-------LNSLVIRDIAGP---HIFKYDWT----

----FTWFSK-----------------------------VRNLQ---------------L

SRVHL-GSLPCI----------------------------------AWRQMG-NI-----

---ECLDVSDN---------RLIGSNL------------YN--------PSCWDGGLPKL

--------------DTFIA-ANNNL-----------------------------------

---------QSLRLISLL------------------------------------------

-----------------------TAKWPKL-TK----LDLSSN----------DLGVYN-

-------------------------EVCT-------------------------------

------------------------------------------------------------

------------------------------------------------------------

-------------------------WIPNI---TTLILK---GNTLK-------------

----MC----VFQC--------------------LPTSVEL-LDLSY----SQLE---QL

D--LNYFNRA-TNLKELVLSHN--KLNFISSD--WRS----P-NLQVLHLEGN-SISLID

K-GTFK-D-----L------------------------PSLR-RLTAGN----NPYDCT-

--CDLYA-----------------------------------------------F--FST

LQ----NNDEML-------------LADW-------PYDYQC---FHPQ----HL---RD

TDVED-----------------YTP-W-RVEC------DV--------------------

----------SLVITISVSTTAAIIILCMLVCWR---FDVPW-YLRMTW-----------

-----RIVK--SKYRSKKSNKS--------RE---Y--NYHAFISYSY---S--DADW-V

RGEL--LYRLE------SCSP----PY----RVCIH-ERDFLPGRW-IIDNIIE-NIENS

RKIIFVLSSN---FINSEWC-NYELYFAHQ--RAIGHSL---ED----IILVVKEKVT-M

EDLPKRFHKLRKMLRTKTYLEWP-----SEPS--KQHFFWIQLKSILG------------

------------------------------------------------------DASVSL

AGQEGLSVVNEAVVGPVFYLYHI-------------------------------------

------------------------------------------------------

>TLR14.1_Xetr

------------------------------------------------------------

--------------------------------MEKLSHH---------------------

---------ERLVILCILAL----------------------------------------

------------------------------------GLVERAQGQTPCKTDGIYANCK-G

R--SL--------AAVP--KDL------PT---TLVELDLSY-N----------RLS---

------------------H----IQFD-DFASF-T----HLRALNLSYNNI---------

------SAIETGSFAS----NVLLTNLTL---FNNSLTE-------------MPSAL---

-------FEPLRFLQFLDIS---------------------------NNFY---NCA---

--------TLG-AEFSM-LENLRN---------------------LSIGGPL-VSKVLKG

DF----------------------------------------------------APIKNI

S--------------LQRFSLKTMSS-L----WL--Y--EKGAFSDLNTQSLWLDIAL--

------------------------------------------------------------

---------------DTNPQALPRM-----------------------------------

---LKDLAGKAFSSL----RFRNLFEFTY-Y----------------------------T

DAMDVFFGLA--DIFIKE---------LTFYRG-KFN-----------------------

------------------------------------------------------------

-----ENLLRLTLKNVEKS----NI-------------QDLFL----LSIDFARSL----

----------STN-----------------------------------------------

-----------RTDIRIN--DL---A-------LRRLVIKDVTNP---DILRFDWT----

----FTWFNK-----------------------------VSHLD---------------I

INVNF-NFVPCD----------------------------------AWSQMV-NV-----

---ERLDISNN---------RLLASNL------------YN--------LLCQYSELPNL

--------------HTFIA-SDNNM-----------------------------------

---------RSLYTLSLL------------------------------------------

-----------------------TLTWPKL-AT----LDLTSN----------YLGALD-

-------------------------EVCT-------------------------------

------------------------------------------------------------

------------------------------------------------------------

-------------------------WTPQI---TKLILK---DNTLK-------------

----VG----VFKC--------------------LPVTVEH-LDMSN----SLLE---RL

D--MDYFNRA-TKLKVLILSQN--KLKFISRDW------KCP-NLQVLGLEGN-SFSVID

K-GSFK-D-----L------------------------PELR-RLTAGD----NPYHCT-

--CDLYA-------------------------------------------F---F--TET

LT----ERRVS--------------LADW-------PEEYNC---YHPP----HL---LD

TKVEF-----------------YNP-G-RVEC------DV--------------------

----------RLVVAISVSTTAVVVMLCMLLCWR---FDVPW-YLRTTCS---------I

VQS--KYRS-----RSFHDSRD-------------Y--NYHAFISYSH---S--DADW-V

RGEL--LYRLE------SCSP----PY----RVCIH-ERDFLPGRW-IIDNIIE-NIESS

RKIIFVLSRN---FVNSEWC-NYELYFAHQ--RAIGHAF---ED----VILVVKEKVT-M

EDLPKRFQKLRKMMRTKTYLEWP-----SEQN--RQHFFWIQLKSILG------------

---------------------------------------------------------KAN

PPVTSQETLSVVSETVAYGTCSVSETPSVPLGKVTLPSS---------------------

------------------------------------------------------

>TLR14.3_Xetr

------------------------------------------------------------

------------------------------MNPVLQNGQ---------------------

---------LGLWCVLVLGL----------------------------------------

------------------------------------VRELWCQTPCLVDESRRFVSCSGR

N-----------LVEIPKNFSV-----------TLEELDMSF------------------

------------------NRIFQIKSD-DFSAY-T----NLRALNLSYNQI---------

------ATIENGSFNS----NTQLRSLTL---FNNSLTE-------------MPSAL---

-------LEPLHLLEFLDMSN--------------------------NFYN---------

--------KSTLGDVFQTLVNLQT---------------------LSIGGPL-VSKVQKD

DF----------------------------------------------------------

---------------VPIRNIGLQKF----------ALKTMSSLTLYEEGAFSVLNTH--

------------------------------------------------------------

---------VLWFDIALDTNPQALLLI---------------------------------

---LKDLKGKSFDVL----RFRNLFEITY-----------------------------YT

DTVDIFSWLP--SISTRE---------LVFYRGKFNE-----------------------

------------------------------------------------------------

----------------------------------------NLLWIMLENIQRSSIL----

----------DLSLL---------------------------------------------

--SVDFSRSHSANKTNVSIDDL----------RLRTLRVKDVTNP---DILRFDWT----

----FTWFRK-----------------------------ISNLY---------------I

INVNF-NSVPCD----------------------------------AWSEMS-NL-----

---EKLDMSTD---------ELVDTYL------------YN--------PWCQDVALPTT

--------------DTFIL-AYNNL-----------------------------------

---------QSLRMLSLL------------------------------------------

-----------------------TAKWPKL-AT----LDLRSN----------SLGSND-

-------------------------EMCT-------------------------------

------------------------------------------------------------

------------------------------------------------------------

-------------------------WTPSI---RTVILK---DNMLK-------------

------------------------VGVF----QCLPTTVEF-LDLSH----SQLE---QL

D--MDYFNKA-TNLKQLILSHN--KIKFISSE--WKS----P-NLQVLALEDN-SFGVIN

V-GSFK-D-----L------------------------PKLR-NLTAGD----NPYGCT-

--CDLYR-----------------------------------------------F--FSQ

IR----EEGRIV-------------LADW-------PQAYKC---YSPP----DL---LD

TKVEF-----------------YNP-G-KVQC------DV--------------------

----------RLVVAISVSTTAVVVILSMLLCWR---FDVLW-YVQTMFA---------I

VQS--KYRS-----RNMGNTKE-------------Y--LYHAFISYSH---S--DADW-V

RGEL--LHQLE------SCSP----PY----RVCIH-ERDFLPGRW-IIDNIIE-NIENS

RKIIFVLSRN---FVNSEWC-NYELYFAHQ--RAVGHAL---ED----VILVVKEKVT-M

EDLPKRFQKLRKLLRTKTYLEWP-----LEHT--RQHFFWIQLRSILG------------

---------------------------------------------------------KVS

SPVIGQDDLLVDNGAAASWDGPASEAHHEDEIYETLIPQ---------------------

------------------------------------------------------

>Ptfl_TLR_5

------------------------------------------------------------

-------------------------MAWKLHGRVDVLAV---------------------

---------LLLVITKTNSQ----------------------------------------

------------------------------------QQTFKYCQSMTSTRVGSRFDCS-S

L----------QLANVP-----------SYLPHSVTELNMDT-N----------YLQ---

------------------D----LGEE-PFIHL-P----QLRKLVLSNSDI---------

------SVLSEKNFQG----LGNLRVLDL---SMNRIRS-------------IECET---

-------FSDLSRLEVLDLSGAVTTPDTSCCYKWEMLDKRYRRLQSGQQTQGALFLN---

--------KCT---WSG-LSNLKK---------------------LILQNAK-IDEKSLL

PK----------------------------------------------------TFSALN

G--------------LKLLDLSSNRL-TNL---------KAGVFSGLNKLETLILNRC--

--------------------------------------NVKTIENGTFSTLHNLKYLNLM

FSNLTDFS-SISYLPKQRLVTLHLG---------------------------GPFHSIFF

DIDYFSLASLKLSPD----TVTCHSKKFN-IKFAEQTFQRKVSLDELYVWFDLIH----R

INVSSFEIFD--HIKLLQLRCSVENCDLAAFASFILK---HSTVSAITMPTSTYPLQICD

KYNLTESFPVVKFTIERPCIETERQFYVTSDHDFSCFKKLKELNLSHIDMVY----FTQH

TFASLEKLQFLNLSYNSLN----RL-------------EDYAF----NKTASLVNL----

----------DLSHN---------------------------------------------

--NVFH-----LIEETFI--GL---A------NLRILNLSY--NS---IQDLSVHT----

----FQNLKS-----------------------------LEYLY---------------L

DNNNL-RTIPSN----------------------------------AFKGLA-KL-----

---REIDLKEN---------YIQTVGD------------G-----------AFSE-LPSL

--------------MFLYL-RKNRL--------------------------------KEW

QPAFDDFSETSLQTLDIG-----------------------------ENLLHCSDRCVAI

LGNCSRLRIIDLSTVYANPTSFKPFELPNI-AE----MTLTKANVLPNKILAPKLKKLD-

-----------VSHSDLRLHAGENNFILR-------------------------------

------------------------------------------------------------

------------------------------------------------------------

-------------------------VEAAV---EELRLS---GNSIN-------------

----SL--------LCTDCSGICSRISL----LANMTSLRV-LDLSS----CDIS---VL

C--EDYFPNQ-PLLEKLNLSSN--KLTYLTADV-FNA----NTKLCTLDLSSN-RLSVID

I-SAFT-K-----F------------------------ANLR-QFYFGN----NPLSCS-

--SRAES-----------------------------------------------L--QIW

MN------DVTN-------------NITFTRDNSFPWQTYIC---KSPG----SL---AG

TPILI-----------------VDF-H-KVNW----------------------------

----------YIIATIIGSVLIICAIACVLIKFT---FKYRW-SIRYRRY---------L

YKL--RHKS--Q-YQRLDPKRD-RS----------I--RYDAYVSFSH---K--DGEW-V

EQELMKLEQVE------EQHR----RM----KFCIC-HRDFLGGTY-IVKNISN-AMRQS

RKVIFIVSEN---FVSSKWC-LFEIEYATQ--LLLETNE---DN----VLFIILDNVS-R

RKMP-DILNL--LMNTRIYLEWP---GDDPDKERERETFWQKLEMSLQ------------

--------------------------------------------------------THAV

D-----------------------------------------------------------

------------------------------------------------------

>Ptfl_TLR_19

------------------------------------------------------------

------------------------------------------------------------

---------MEETLVRP-------------------------------------------

------------------------------------DTLALTLGSVSGTSVGYISDSPFQ

TLVNLKTLVMVSLNEFPWKRTE-----------MPEPSSMQI------------------

-----------------------ITKG-WFRGL-K----HLQYLNLSMNFI---------

------QGIQDGAFEG----MGALRKLCL---QWNDLAT-------------IERET---

-------FIGLENLQMLNLRV--------------------------NGIS---------

--------VIREGALDN-LKELRE---------------------LNLAGNF-LRQVGRD

------------------------------------------------------VFMNLK

C--------------LTILNLSHNQL------KSIPAVSCTSGLQSLILSGNLIGTAE--

------------------------------------------------------------

--------IPRTMSSCLTLKILDLSSN-----------------------RINYLVKGDF

HWLNRSSAGVRLKGN----HLYGISANSFTPGQRLTFMSFELSVRTSSSL---------Q

ENLKAFEDVK--VKEID----------LSIISNNQLE-----------------------

--------------------------------------------------------FDNG

TFQALQNSDVSAFTMADCS--------------WAHHFGDFVF----SSMATLTEL----

----------VFDVE---------------------------------------------

--NCAAS----FSASTLA--GL---Y------NLENLNFLD-------LTYFLSDG----

------ILET-----------------------------VSKYP---------------L

KKLSFHLRYPVG----------------------------------ALEEMS-RL-----

---EKLNLSYA---------FMSYPSS------------LS---------YGYLRGLKAV

--------------RELIL-SHNFI---------------------------------GS

IDNDTFCDLVSVEYFDFS------------------------------------------

--------YNPLELKGLTVQTENCTNFDKL-TV----MNLNNT----------AVQLEN-

----------------------SDQSLHS-------------------------------

------------------------------------------------------------

------------------------------------------------------------

-------------------------FLVSLPSLKELHLSHILQGSSA-------------

----SN-------YYDCTVRTLTSGLTI----FRNLMALEY-LGLSE----NCLV---SM

H--ERSFQDL-PNLKHLDLGKN--YLKTLSGSL-WVG----LERLEILDLSNN-HIQVIN

R-TTFQ-G-----L------------------------SRLK-KLDLSG----NPLKCN-

--CDMQ--------------------------------------------W---F--RKW

ID-----MKTVM-------------LTSF-------PNNYTC---ELLT----DH---TN

INLID-----------------FNP-D-MLDC----------------------------

----------SREVFQIVTLSV---------------------SERYNYE----------

-----KLQN--------------------------F--EFDAFISYTY---D--DFPW-V

RNQL--MRTLE------ENKLDG--KF----KLCVK-DRDFLVGED-IPVNAIN-AVTNS

NKSVFVLSEN---FVRDDWC-MFELKVALQ--QLIDHHR---DV----IVLVSLGDIP-D

NKMP-LLLRM--LRAEKKTLTWT-----DDER--GQIIFWDDMRNKLR------------

------------------------------------------------------K-SSAF

DHRFEV------------------------------------------------------

------------------------------------------------------

>Stpu_TLR_84

------------------------------------------------------------

------------------------------------------------------------

---------SLLSTRLLVKDVSASL-----------------------------------

------------------------------------SPESTRRCQLKNTSLGSKATCT--

---------HLDLKSVP-----------LDLPSNTVMLDLSF------------------

------------------NKITSLFNS-SFAYV-P----DITTLGLASNVL---------

------SKIENGAFKP----LPHLRELSL---RQNRLAS-------------LPPGL---

-------FWTNNFLSILILCR--------------------------NRLL---------

--------SFPSDALPW-SNSIKK---------------------LELSTNN-ISFIDSH

------------------------------------------------------DLKPLQ

NCS------------LERLSLENNAFNSLPLN----IFSYLSTVTFLNLSGNNFTTFH--

-----------------------------------------------------------T

STVLGRTVITNLYINFCKIHEIIPLNK-----------------------------SHVS

LGEQGNIFKLSLTAN----IISYVPDFAF-------------WGFNQTKILSFHRSQVAS

LSNRSFCGLD----HLIQ---------LDLSYNRLAT-----------------------

--------------------------------------------------------LSLD

MFSCNNMLQQLKLNGNNIA---------------------RLFIGEEWGISLLNHL----

----------DLAYN---------------------------------------------

--NIKD-----IESNRNN-VTF---P------SVEYIDLSF--NK---FTGIRRLM----

----LWGFTN-----------------------------LKILN---------------M

SNNDISDPYSPK----------------------------------LFKNLR-LL-----

---QELYLTNE---------HAQGINE-------------------------AFRYLGAL

--------------RVLNL-SFAPL---------------------------------KL

TSISQFTNTSSLTRLIMC------------------------------------------

-----ENSLRSTDMYHVQTKSSLFHGLDSL-ER----LALRQN----------KLDMLA-

-----------------------PGTFNP-------------------------------

------------------------------------------------------------

------------------------------------------------------------

--------------------------LKKL---KILDLS---QSSIT-------------

----VL------------------SCGV----FDSLTALRS-LDLCH----NEIK---KI

P--ECLLRTQ-HHLAVLFLSNN--ELETIPRTL-FNE----TTSLHSLFIQQN-KIATIE

PKTMFP--------------------------------TNMTMKLDAYG----NPFSCT-

--CHLS--------------------------------------------W---F--VKW

LR-----SGNVK-------------LLNR--------EKTFC---SLTS--IREE---VN

SPILS-----------------FNP---DQYC------GT--------------------

----------DIVMITGVSFSVVLIVVICLVA-----YRKRR-WLNYKIF---------L

LKL--AICG----YEEINQDFDAQD----------Y--EYQLNIMYNE---D--DEEW-V

DRIM--KPMLQ------ERFP----HLR---KVIFG-DNDLNIGMF-YINALHY-AAEIS

FKTVLLISYN---SVDDAWF-LTKLRIALE--HINDTRL---DK----VILIFIEDIQ-D

EDLP--YLVRLFMSKNKPYMLWT-----ADED--GQKLFWAQFEKSMR------------

------------------------------------------------------A-NRAF

NSVIPV------------------------------------------------------

------------------------------------------------------

>Stpu_TLR_76

------------------------------------------------------------

------------------------------------------------------------

------------------------------------------------------------

------------------------------------------------------------

------------------------------------------------------------

----------------------------------S----LLNHLDLAYNNI---------

------KDIESNRNNV---TFSSVEYIDL---SFNKFTG---------------------

-------------IRRLMLWG---------------------------------------

------------------FTNLKI------------------------------------

------------------------------------------------------------

------------------------------------------------------------

------------------------------------------------------------

------------------------------------------------------------

------------------------------------------------------------

---------------------------LNMSNN---------------------------

------------------------------------------------------------

------------------------------------------------------------

----------DISTP---------------------------------------------

-----------YSPKSFK--NL---R------LLQELYLTN--EH---AQGINEA-----

----FRYLGA-----------------------------LRVLN---------------L

SFAPL-----------------------------------------KLTSIS--------

---QFTNTSSL-------------------------------------------------

--------------TRLIM-CENSL-----------------------------------

---------RSTDMYHVQ------------------------------------------

------------------TKSSLFRGLDSL-ER----LALRQN----------KLDMLA-

-----------------------PGTFNP-------------------------------

------------------------------------------------------------

------------------------------------------------------------

--------------------------LKKL---KILDLS---QSSIT-------------

----VL------------------SCGV----FDSLTALRS-LDLRH----NEIK---KI

P--ECLLQTQ-HHLAVLFLSNN--ELETIPRTL-FNE----TTSLHSLFIQQN-KIATIE

PKTMFP--------------------------------TNMTMKLDAYG----NPFSCT-

--CHLS--------------------------------------------W---F--VKW

LR-----SGNVK-------------LLNR--------EKTFC---SLTS--IREE---VN

SPILS-----------------FNP---DQYC------GT--------------------

----------DIVMITGVSFSVVLIVVICLVA-----YRKRR-WLNYKIF---------L

LKL--AICG----YEEINQDFDAQD----------Y--EYQLNIMYNE---D--DEEW-V

DRIM--KPMLQ------ERFP----HVQ---KVIFG-DNDLNIGMF-YINALHY-AAEIS

FKTVLLISYN---SVDDAWF-LTKLRIALE--HINDTRL---DK----VILIFIEDIQ-D

EDLP--YLVRLFMSKNKPYMLWT-----ADED--GQKLFWAQFEKSMR------------

------------------------------------------------------T-NRAF

NSVIPV------------------------------------------------------

------------------------------------------------------

>Stpu_TLR_78

------------------------------------------------------------

-----------------------MMAVNAHAISFVVLCG---------------------

---------FTLLYGVVMNH----------------------------------------

------------------------------------TSITLNHTCSENSSDHS-ANCS-Y

R--GL--------TSVP--RNL------SH---DLRSLKVSD-N----------NIS---

------------------M----LLDQ-SFVNY-K----QLETLDASYNSI---------

------YLIENETFHA----LLLLNVLML---NHNNISA-------------LPISL---

-------LEKNCHLSSLILNHNIL---------------KGIPRIFGNNLQTT-KFA---

--------EDG-EDACG-CKNLSR---------------------FDLSLNK-VRSLVQE

DF----------------------------------------------------VALQNC

S--------------FKRFNLNYNDI------KN--L--SRAVFTDLPAVNLLINYIS--

------------------------------------------------------------

---LAKFH-AESFLGNKAIVKATIT-----------------------------------

-----RSGITSIVPM----NTSEIPRHLF-PG------------IIELYLSRNEL-V--T

IPKYALDGFE----KLQV---------LDIGFN-HLA-----------------------

---------------------------------------------------S----LHNE

SFCGLKSLVNLKLEVNKIK----SL-------------PRGSF----ACAEKLESI----

----------DLSHN---------------------------------------------

--DLAV-----LDPQWFD--GS---H------RLSTLTFYQ--SN---IDEIKTIP----

----WNATN------------------------------LQTLI---------------L

ANNNL-NSVNKN----------------------------------TLLGLK-NL-----

---KKLDFTRNYKPLKISVDAFEETSS------------LEKIIMTDLVKFIMTGCFSNM

--------------HQLVFLDMSYLSS-----------------------------HLEI

NSCDQFSHTSALRTLNLS------------------------------------------

-----KTKIKAEDLVQFKSNRSLFSGLVSL-RT----LKLSHN----------FFDDFH-

---------------------HVPNAFTT-------------------------------

------------------------------------------------------------

------------------------------------------------------------

--------------------------LWNL---HELDLA---DCRIH-------------

----KI------------------DSGI----FRNLTSLAY-LSLAI----NYIR---IV

P--EKAFQDL-QNLRSLKLQYN--SITVIEKKL-FSK--T-D-SLQYLSLHGN-QISTID

P-FTLI-------------------------------PPSLK-VLIIAR----NPFTCT-

--CQFA--------------------------------------------W---F--REW

LD------KVNT-------------TIYR-------RNETRC---SSTS--FKLL---KD

QTIWS-----------------FHP---KDYC------GV--------------------

----------NIYLIVGVSLAIVTVLSLSVI--V---YLKRW-WLNHKLF---------L

LKL--AIIG----YQEIIENQG-P------ED---Y--EYQLNLMFRE---D--DEWW-I

NDCM--KPFLQ------GRMP----HLE---HIVFG-DSGLHPGSF-YLNAIYD-VIENS

YKTVLLLSNQ---SVEDTWF-MTKLRMAVE--HMNDTKL---EK----IILIFLEDID-D

DHLP--YLVRLLLSRNKPYLLWV----DDDED--GQEFFWAKFEKSMR------------

------------------------------------------------------S-NREM

NNVIPV------------------------------------------------------

------------------------------------------------------

>Stpu_TLR_98

------------------------------------------------------------

------------------------------------------------------------

------------------------------------------------------------

------------------------------------------------------------

------------------------------------------------------------

-----------------------MGKT------------HADVLDIGRNQL---------

------SSLYNGSFCG----LKSLIDLNL---SVNKITS-------------LPRGS---

-------FACAEKLESINLS----------------------------------------

------------------LNDIAV------------------------------------

------------------------------------------------------------

------------------------------------------------------------

------------------------------------------------------------

------------------------------------------------------------

------------------------------------------------------------

------------------------------------------------------------

------------------------------------------------------------

------------------------------------------------------------

------------------------------------------------------------

-----------LDPQWFD--GN---H------CLSTLRLYQ--SN---IHEIKTVP----

----WNATN------------------------------LQTLN---------------L

ANNNL-------------------------------------------------------

-----ISVNRN-------------------------------------------------

------------------------------------------------------------

------------------------------------------------------------

----------------------TFVGLRNL-KF----LDLSRN-----------------

------------------------------------------------------------

------------------------------------------------------------

------------------------------------------------------------

------------------------------------------HNPLD-------------

----IS-------------------VDA----FEETSSLEK-IIMKD------LV---KF

T--MTAFQDL-QKLRNLNLASN--YITVIEKQL-FSR----TSSLQYLYLQNN-QISTID

S-FTLL-P------------------------------TSLK-VLIIAN----NPFTCT-

--CQLA--------------------------------------------W---F--REW

LD------KVNT-------------TIYQ-------RNETRC---SSTS--FKSL---NN

QTIWS-----------------FHP---EDYC------GV--------------------

----------NIYLIVGVSLAIVTVLSLSVL--V---YQKRW-WLNHKRF---------L

LKL--AIVG----YQEIIENQGPED----------Y--EYQLNLMFRE---D--DEWW-I

NDCM--KPFLQ------GRMP----HLE---HIVFG-DSGLHPGSF-YLNAIYD-VIENS

YKTVLLLSNQ---SVEDTWF-MTKLRMAVE--HMNDTKL---EK----IILIFLEDID-D

DHLP--YLVRLLLSRNKPYLLWV----DDDED--AQEFFWAKFEKSMR------------

------------------------------------------------------A-NREM

NNVIPV------------------------------------------------------

------------------------------------------------------

>Stpu_TLR_90

------------------------------------------------------------

------------------------------------------------------------

------------------------------------------------------------

-------------------------------------------------MAQITMDSL-T

R-TLL----------------------------ISMAIVLGC------------------

-----------------------IVQK-VRVGM------PISSQQPNYTCP---------

--------------------VRDLQIAKC---AHLNLTS-------------IPQDL---

----------PHGLLVLSIRQ--------------------------NQLT---ELI---

--------NMS---FMN-YNQLEE---------------------LYAGHNF--------

------------------------------------------------------------

------------------------------------------------------------

------------------------------------------------------------

------------------------------------------------------------

------------------------------------------------------------

------------------------------------------------------------

------------------------------------------------------------

------------------------------------------------------------

------------------------------------------------------------

---IAF-----IDSGTFE--TL---P------QLQVVRLDY--NL---ITFLPIYF----

----LQKNAL-----------------------------LYIIS---------------L

SHNKI-----RF----------------------------------ALRGLE-KL-----

---QVLSLGEN---------RISNINN------------E-----------SFCG-LHSL

--------------LDLKL-----------------------------------------

---------YDNELNSIS------------------------------------------

--------------------RALFACASNL-QK----IDLSRN----------KLAALD-

-----------------------PQWFDG-------------------------------

------------------------------------------------------------

------------------------------------------------------------

--------------------------SRYL---RNLILY---KCGIS-------------

----RI-------------------TVG----PWNATNLQT-LVLNK----NNLG---FL

H--HYTFTGL-AKLKTLDVSGNANSLKLSEDALTSVG------SLELLIMTDLGKFTMKA

S-NTFM-P-----S-------------------------SLI-RFNIAY----NPLTCD-

--CQ-LA-------------------------------------------W---F--RQW

LN------EAEG-------------NIDL-----APKNQIRC---SSSS--LKVL---VN

QIIWS-----------------FHP---NEYC------GI--------------------

----------NTMIIVSACFAPILVLTLGIL--V---YLNRW-WINYKLY---------L

LKL--AIVG----YHEITEDRT-P------ND---Y--EFQLNLMFHD---G--DEWW-V

DDCM--KPFLE------QRMP----HLE---RVIFG-DADLHPGSF-YLNAIYD-VIENS

HKTILLLSNQ---SVDDTWY-MTKLRMAVE--HMNDTKL---EK----VILIFLEDID-D

DHLP--YLVRLLLSRNKPYLLWT-----EDEE--GQEVFWAKVQKSMR------------

------------------------------------------------------S-NRQM

NNVIPV------------------------------------------------------

------------------------------------------------------

>Stpu_TLR_50

------------------------------------------------------------

------------------------------------------------------------

------------------------------------------------------------

-----------------------------------------------------------K

Y-----------------------------------------------------------

-----------------------IHPA-NISSMRKENVPLIKDLNLDLNIL---------

------KHIPGFALRG----LEKLQVLSL---GGNRISN-------------INNES---

-------FCGLHALVNISLYG--------------------------NRIQ---SLP---

--------RAS---FAC-ASNLKK---------------------IDLSRNN--------

------------------------------------------------------------

---------------LVTLD----------------------------------------

------------------------------------------------------------

---------PQWFDGSFFLRNLVIDQS---------------------------------

-----------------------------------------------------------G

IRSITFRPWE--VTNLQT---------LVLTKNFIKT-----------------------

------------------------------------------------------------

------------------------------------------------------------

------------------------------------------------------------

-----------LYHETFT--GL---E------KLKALNLSGNANR----LRILGDA----

----LTSLGS-----------------------------LELFD---------------M

SHSNELTMKG------------------------------------SFRNMQ-NL-----

---LYLDMSYS---------LLKMSSI------------D-----------QFTN-TSAL

--------------RVLNM-SGSHL-----------------------------------

---------KAEDLVDIQ------------------------------------------

------------------TGTSLFSGLVSL-HI----LKLRKN----------SLDNLH-

---------------------NIPGIFTP-------------------------------

------------------------------------------------------------

------------------------------------------------------------

--------------------------LGSL---VELDLT---SCRIL-------------

----QV------------------ASGT----FANLTTLLV-LRLPQ----NQLT---SI

S--KDAFHGL-HNLQVLQLQYN--SITFIDQEL-FWG----TNELVQLYLQNN-HISTVA

S-NTFM-P------------------------------STLI-RLNIAK----NPLSCH-

--CELT--------------------------------------------W---F--RQW

LN------EVEG-------------KINF-----DPENETLC---SSSS--LKPL---VN

QIIWS-----------------FHP---FEYC------RV--------------------

----------NAMIIVSACFAPILVLTLGIL--V---YLNRW-WINYKLY---------L

LKL--AIVG----YHEITEDRTPED----------Y--EYQLNLMFHE---D--DEWW-V

NDCM--KPFLE------QRMP----HLE---RVIFG-DADLHPGLF-YLNAIYD-VIENS

HKTILLLSNQ---SVDDAWY-MTKLRMAVE--HMNDTKL---EK----VILIFLEDID-N

DHLP--YLVRLLLSRNKPYLLWT-----EDEE--GQEIFWAKVQKSMR------------

------------------------------------------------------S-NRQM

NNVIPV------------------------------------------------------

------------------------------------------------------

>Stpu_TLR_51

------------------------------------------------------------

------------------------------------------------------------

------------------------------------------------------------

------------------------------------------------------------

---------------------------------------VTF------------------

-----------------------IQSK-AFGHL-P----KLGTLNMNNIKL---------

------ATFDVRYFMG----HVEIERLLI---DSSGIK----------------------

-------YIHPANISSMRKEN---------------------------------------

------------------VPLIKD---------------------LNLNLNI-LKHIPGF

------------------------------------------------------ALRGLE

K--------------LQVLSLGGNR-----------------------------------

------------------------------------------------------------

------------------------------------------------------------

------------------------------------------------------I-S--N

INNESFCGLH----ALVN---------INLYGN-RIQ-----------------------

------------------------------------------------------------

------------------------------------SLPRASF----ACASNLKKI----

----------DLSRN---------------------------------------------

--NLVT-----LNPQWFD--GS---L------FLRSLVIDQ--SG---IRSITFRP----

----WEVTN------------------------------LQTLV---------------L

TKNFI-KTLYHE----------------------------------TFTGLE-NL-----

---KALNLSGN-------AKRLRILGD------------AL------------TS-VGSL

--------------ELFDMSHSNEF-----------------------------------

------------------------------------------------------------

----------------------TMKGLVSL-RI----LKLRQN----------SLNTTH-

---------------------ALPGIFTP-------------------------------

------------------------------------------------------------

------------------------------------------------------------

--------------------------LRNL---VELDLT---SCCIK-------------

----QV----ASRT------------------FANLTTLLQ-LSLQD----NDLT---SI

P--KDAFQGL-QNLQVLRLQNN--LIKFIHQGL-FMG--T-N-ELEQLYLQNN-HISTVA

S-NTFM-P-----S-------------------------SLI-RFNIAY----NPLTCD-

--CQ-LA-------------------------------------------W---F--RQW

LN------EVEG-------------KIDL-----APKNQTRC---SSSS--LKVL---VN

QIIWS-----------------FHP---DEYC------GI--------------------

----------NTMIIVSACFAPILVLTLGIL--V---YLNRW-WINYKLY---------L

LKL--AIVG----YHEITEDRN-P------ED---Y--EFQLNLMFHD---D--DEWW-V

NDCM--KPFLE------QRMP----HLE---RVIFG-DADLHPGSF-YLNAIYD-VIENS

HKTILLLSNQ---SVDDTWY-MTKLRMTVE--HMNDTKL---EK----VILIFLEDID-D

DHLP--YLVRLLLSRNKPYLLWT-----EDEE--GQEVFWAKVQKSMR------------

------------------------------------------------------S-NRQM

NNVIPV------------------------------------------------------

------------------------------------------------------

>Stpu_TLR_5

------------------------------------------------------------

------------------------------------------------------------

------------------------------------------------------------

------------------------------------------------------------

------------------------------------------------------------

----------------------------------E----QVTFLENSFCQ----------

--------------------LHTLLILDL---SNAPIQV---------------------

------------------------------------------------------SLT---

--------STE--QFSN-MSSLSE---------------------LRMEKAQ--------

------------------------------------------------------------

---------------LEDTDLYDEV-----------------------------------

------------------------------------------------------------

------------------------------------------------------------

------------------------------------------------------------

------------------------------------------------------------

------------------------------------------------------------

------------------------------------------------------------

------------------------------------------------------------

-----------KHQSLFT--GL---F------SLRKLRIKD--NY---------------

---------------------------------------LHDLD----------------

------------------------------------------------------------

------------------------------------------------------------

------------------------------------------------------------

------------------------------------------------------------

--------------------VRVFQNLSQL-VY----LDMTNS----------RIHTLR-

------------------------------------------------------------

------------------------------------------------------------

------------------------------------------------------------

---------------------------SGL------------------------------

--------------------------------FSPLSSLRY-LYIGE----NNLG---EV

P--GDIFNGL-FRLNVLTFQNN--ILSSLDPKT-FAQ--T-L-RLTDLYLPGN-QISTIK

P-GTVL-P-----G------------------------NTSL-RFDISK----NPFSCT-

--CS-LA-------------------------------------------W---F--RQW

LD--------SA-------------DIDF-----KHADQTLC---SGTS--LKGL---SK

QPILS-----------------FHP---DDHC------GV--------------------

----------NIFLIAGISFTGIFLFFITLL--A---YNRRW-WLNHKLF---------L

LKL--AVVG----YKEMAEDFD-A------DN---Y--EFHLNLMFLE---E--EEEW-V

DRVM--KPALE------ERFP----HLQ---NIIYG-DKDLHLGMF-YINAIND-ALDNS

FKTVLLISNQ---SIRDAWC-MTKLRMALE--HLNETGL---DK----IILIFLEDIE-D

ENLP--YLVRLFMSRNKPYMLWT-----DDED--GQELFWAQFEKSMR------------

------------------------------------------------------A-NKAI

NNAIPL------------------------------------------------------

------------------------------------------------------

>Stpu_TLR_63

------------------------------------------------------------

------------------------------------------------------------

------------------------------------------------------------

------------------------------------------------------------

--------------------LL------PP------------------------------

------------------------DNASDYHTPYP----SILKLDMRRNSI---------

------ENVPPGSFWG----FIWLKMLLL---SYNKISS-------------LSNES---

-------FCLLTSLLELDVSS--------------------------NKLV---SLP---

--------PET---FAC-LPNLTT---------------------LDVSNNL-LPNISPQ

------------------------------------------------------SFDGMP

L--------------IRSISLSGNRI----------------------------------

------------------------------------------------------------

------------------------------------------------------------

------------------------------------------------------------

---------------------------TDLNIGGRL------------------------

------------------------------------------------------------

-----------------------------------------------WTLGTLKAL----

----------DISNN---------------------------------------------

--YIPL-----IPRGKFK--GL---I------NLQVLDVSN--NQ---LTSYSEYA----

----FTDLVL-----------------------------LREQH---------------L

SNEKVVLLKD------------------------------------TFKQLR-TL-----

---LYLDLSYT---------DIQVSQS------------SI---------EQFYN-MTCL

--------------KDLNL-ESATL-----------------------------------

---------RDTDLYNGI------------------------------------------

------------------NNRSLFSGLFSL-KK----VHLNDN----------YLVSLD-

-----------------------ERVFHN-------------------------------

------------------------------------------------------------

------------------------------------------------------------

--------------------------LSKV---YYLDLS---KSRKQ-------------

----VL------------------RPGV----FGPLSFLRV-LYLRE----NKLV---EM

T--GDIFHGL-YLLNAVYIADN--MLSGLEPTT-FAQ--A-P-RLTVLSLSGN-QISTVE

R-GTVL-P-----A------------------------NTSL-RLDISR----NPFTCT-

--CT-LT-------------------------------------------W---F--RKW

LQ-----------------------LADI---DLQHPEKTLC---SKTS--LKGL---VN

QPIMA-----------------FHP---EDHC------GA--------------------

----------NIVLITVLTFTGVVLVMISML--A---YNKRW-WLNHKFF---------L

LKL--AVIG----YEEMAEDFN-A------DN---Y--RHHLNLMFEE---A--EQEW-V

DRVM--RPFLE------ERMP----HLQ---NIIYG-DEDLHLGMY-YIPALYD-AIDNS

FKTVLLLRNQ---SVNDGWT-MTKLRMALE--HLNDSGL---DK----VILIFVEDIE-D

ENMP--YLVRLFLSRNKPYMLWT-----DDED--GQELFWAQFEKSMR------------

------------------------------------------------------A-NKAI

NNAIPL------------------------------------------------------

------------------------------------------------------

>Stpu_TLR_6

------------------------------------------------------------

------------------------------------------------------------

------------------------------------------------------------

------------------------------------------------------------

------------------------------------------------------------

------------------------------------------------------------

--------------------------------DTD-----------------LYNGI---

------------------------------------------------------------

------------------------------------------------------------

------------------------------------------------------------

------------------------------------------------------------

------------------------------------------------------------

------------------------------------------------------------

------------------------------------------------------------

------------------------------------------------------------

------------------------------------------------------------

------------------------------------------------------------

------------------------------------------------------------

-----------NNRSLFS--GL---F------SLKKLRLND--NY---------------

---------------------------------------LVSLD----------------

------------------------------------------------------------

------------------------------------------------------------

------------------------------------------------------------

------------------------------------------------------------

--------------------KRVFHNLSNV-YY----LDMSKS----------RIQVLR-

------------------------------------------------------------

------------------------------------------------------------

------------------------------------------------------------

-----------------------------L------------------------------

----GV--------------------------FSPLSSLKF-LYLRE----NKLV---EM

A--GDIFHGL-YQLRVVNIPDN--ILRGLKPTT-FAQ----APRLTDLSLSGN-QISTIG

R-GTVL-P------------------------------ANTSLRLDISR----NPFTCT-

--CSLT--------------------------------------------W---F--RQW

LQ-----SADID-------------LKHA--------EKTLC---SKTS--LQGV---VN

QPIMA-----------------FHP---EDHC------GA--------------------

----------DIVLITVLTLTGVVLVMISMLA-----YNKRW-WLNHKLF---------L

LKL--AAIG----YEEMAEDFNADN----------Y--RHHLNLMFEE---A--EQEW-V

DRVM--RPALE------ERMP----HLQ---NIIFG-DEGLHLGMY-YIPALYD-AIDNS

FKTVLLLSNQ---SVNDAWT-MTKLRMALE--HLNDSGL---DK----VILIFVEDIE-D

ENLP--YLVRLFLSRNKPYMLWT-----DDED--GQELFWAQFEKSMR------------

------------------------------------------------------A-NKAI

NNAIPL------------------------------------------------------

------------------------------------------------------

>Stpu_TLR_44

------------------------------------------------------------

------------------------------------------------------------

------------------------------------------------------------

------------------------------------------------QLHTVLLLCI--

------------------------------------------------------------

---------------------------------------SNSLIEISQDSI---------

-----------KQFVN----MTHLHELRM---EKAQLTG---------------------

-----------------------------------------------SHLY---------

------------------------------------------------------------

------------------------------------------------------------

------------------------------------------------------------

------------------------------------------------------------

------------------------------------------------------------

------------------------------------------------------------

------------------------------------------------------------

------------------------------------------------------------

------------------------------------------------------------

----------DVVNN---------------------------------------------

-------------QSLFT--GL---L------ALKRLRLKD--NY---------------

---------------------------------------LHSLD----------------

------------------------------------------------------------

------------------------------------------------------------

------------------------------------------------------------

------------------------------------------------------------

--------------------SRVFYNLSQL-YH----LDMTNS----------RIQVLR-

------------------------------------------------------------

------------------------------------------------------------

------------------------------------------------------------

------------------------------------------------------------

-------------------------PEV----FHPLSSLAQ-LYLSD----NKLV---EI

A--GDTFHGL-SLLKVLYLQNN--SIRGLEATT-FAQ----NPRLKNLFLPGN-QISIIK

P-GTVL-P------------------------------SNISLRLDVSR----NPLTCT-

--CSLS--------------------------------------------W---F--RQW

LD--------SA-------------DINF-----ERADQTLC---SGTS--LKEL---AN

KPILS-----------------FNP---EDHC------GV--------------------

----------NIILIVVVSFSGVLVGMMAMLA-----YNKRW-WLNHKLF---------L

LKL--AIIG----YEEMAEDFNAGN----------Y--LHHLNLMFEE---A--EEEW-V

NQVM--KPALE------ERLP----HLQ---NIIYG-DEDLHLGMY-YINALYD-AIDNS

FKTVLLLSNQ---SVNDAWT-MTKLRMALE--HVNDTGL---DK----VILIFVEDIE-D

DNMP--YLVRLFLSRNKPYMLWT-----DDED--QQELFWVQFEKSTR------------

------------------------------------------------------A-NKAI

NNTIPL------------------------------------------------------

------------------------------------------------------

>Stpu_TLR_99

------------------------------------------------------------

------------------------------------------------------------

------------------------------------------------------------

------------------------------------------------------------

------------------------------------------------------------

------------------------------------------------------------

--------------------MTHLYELRM---EKAQLTG---------------------

-----------------------------------------------SHLY---------

------------------------------------------------------------

------------------------------------------------------------

------------------------------------------------------------

------------------------------------------------------------

------------------------------------------------------------

------------------------------------------------------------

------------------------------------------------------------

------------------------------------------------------------

------------------------------------------------------------

----------DVVNN---------------------------------------------

-------------QSLFT--GL---H------ALKRLRLKD--NY---------------

---------------------------------------LHSLD----------------

------------------------------------------------------------

------------------------------------------------------------

------------------------------------------------------------

------------------------------------------------------------

--------------------SRVFYNLSKL-YY----LDMTNS----------NIHVLR-

------------------------------------------------------------

------------------------------------------------------------

------------------------------------------------------------

---------------------------SGV------------------------------

--------------------------------FYPLSSLAV-LRLSG----NKLA---EI

A--GDTFHGL-SHLSFLNLQNN--GLRGLEVTT-FAQ----NPKLKTLLLPGN-QISIIK

PETVFP--------------------------------SNISLHLDVSR----NPFACT-

--CSLI--------------------------------------------W---F--RQW

LH-----SANID-------------LKHA--------DQTLC---SGTS--LKEF---VN

KPILS-----------------FHP---EDHC------GV--------------------

----------NVVLIVVLSFLGVLVGVMAMLV-----YNKRW-WLNHKIF---------L

LKL--AIVG----YIEMEEDFNAGN----------Y--RHHLNLMFHE---T--EEEW-V

NQVM--KPALE------ERLP----HLQ---NIIYG-DEDLHLGMY-YINALYD-AIDNS

FKTVLLLSNQ---SVNNAWT-MTKLRMALE--HINDTGL---DK----VILIFVEDIE-D

DNLP--YLVRLFLSRNRPYMLWT-----EDED--RQELFWAQFEKSTR------------

------------------------------------------------------A-NRAI

NNAIPL------------------------------------------------------

------------------------------------------------------

>Stpu_TLR_33

------------------------------------------------------------

------------------------------------------------------------

------------------------------------------------------------

------------------------------------------------------------

------------------------------------------------------------

-----------------------MSTN---------------------------------

------VEILNQVFSG----LKNLIKLNI---SSSKIAS---------------------

------------------------------------------------------------

------------------LGSINQ------------------------------------

------------------------------------------------------------

------------------------------------------------------------

------------------------------------------------------------

------------------------------------------------------------

------------------------------------------------------------

------------------------------------------------------------

------------------------------------------------------------

------------------------------------------------------------

------------------------------------------------------------

----------------FT--NT---T------SLRELSMRK--NN---------------

---------------------------------------LKSQD----------------

----------------------------------------------LFDNVN-QL-----

------------------------------------------------------------

------------------------------------------------------------

------------------------------------------------------------

---------------------SLFGGLISL-RK----LNLREN----------YLTGLE-

-----------------------PGVFSP-------------------------------

------------------------------------------------------------

------------------------------------------------------------

--------------------------LTKL---SSLDLS---QGNIV-------------

----IL------------------KPGL----FKGMTSLRS-LYLDN----NCIM---TT

S--ANLFSGL-DNLGSLFLRNN--KLRILDKYL-FGA----TPNLRYLHLSSN-KLSQVQ

S-DTFF-P------------------------------TNKSLLIDVSN----NPFSCT-

--CELS--------------------------------------------W---F--RSW

LD--------EI-------------NTRF-----VNPDKTLC---SKTS--FKAV---ID

MPILS-----------------FDP---AYFC------GI--------------------

----------NIILITSLFFFACIVIYVCVL--V---YHKCW-WLKHKFL---------L

LKL--AIAG----SGKIMEDFKEDN----------Y--DFHLNLMFHD---A--EEGW-V

DRVL--RPVLE------KRFP----HLQ---NIIYG-DRDLRVEMF-YINAIYD-AIENS

FKTVLLMSNR---SMYDIWC-MTKLRLALE--HINDTGL---DK----VILIFVEDIE-D

DDLP--YLVTLFLSKNNPHMWWT-----DDED--EQELFWAQFQRSMR------------

------------------------------------------------------A-NRAI

INAIPL------------------------------------------------------

------------------------------------------------------

>Stpu_TLR_48

------------------------------------------------------------

------------------------------------------------------------

------------------------------------------------------------

------------------------------------------------------------

------------------------------------------------------------

-----------------------MSEN------------KITSLVV--------------

-----------DSFCG----LDSLTHLDI---SHNLIVS-------------LHPGM---

-------FSCNLQLQELFISY--------------------------NNIA---------

------------------------------------------------------------

------------------------------------------------------------

------------------------------------------------------------

------------------------------------------------------------

------------------------------------------------------------

-----------------------------------------------------------V

LNTMSYQGLS----LRK----------LNISNN-VMK-----------------------

------------------------------------------------------------

------------------------------------NPHHVQY----WDNPSLAVI----

----------DISNN---------------------------------------------

--QITR-----INQDLFR--GV---L-----GNLHILILSN--NK---IGSFSPVT----

----FSNVPS-----------------------------LQKLY---------------L

KNEVGQRLNG------------------------------------VFSNMT-NL-----

---IKLDLSFT---------RTTFTSI------------R-----------QFTE-TRSL

--------------KRLQM-SHTEL-----------------------------------

---------KAPDLFDNQ------------------------------------------

------------------TQSSFFDGLVSL-ER----LNLSGN----------LLSNMS-

-----------------------PGVFRS-------------------------------

------------------------------------------------------------

------------------------------------------------------------

--------------------------LTKL---KVLDLS---QASIV-------------

----IL----KPEL------------------FKGLVSLTS-LNLNE----NNIL---NT

S--AQVFSGL-DNLGSLYFENS--KLEFIDPDT-FLK--T-P-NLRSLFLSGN-RLTKVQ

N-DTFF-P-----T----------------------SINHTL-TIDVSA----NPFSCT-

--CE-LS-------------------------------------------W---F--ITW

LH--------ES-------------NINL-----KHPNQTIC---SRTS--IKEV---VN

LRILM-----------------FDP---ADFCFVNSVDSV--------------------

----------NILLIVALSFFGVMVGFVSIL--A---YSKRW-WLNHKLF---------L

LKL--AIIG----YQEMAEEFD-E------DN---Y--EFHLNLMFHE---A--EEEW-V

DQVL--KPGLE------ERLP----HLQ---NIIYG-DKDLHLGMY-YVNAIFD-AIDNS

FKTVLLISNQ---SIDDPWC-MTKLRMSLE--HLNDTGL---DK----VILIFLEDID-D

DHLP--YLVRLFMSRNKPYMLWT-----ENED--GQELFWAQFEKSMR------------

------------------------------------------------------A-NRAI

NNIIPV------------------------------------------------------

------------------------------------------------------

>Stpu_TLR_40

------------------------------------------------------------

----------------------------MIAMESIGLLW---------------------

---------ITCIIAYSLMM----------------------------------------

--------------------DSIGHTGADAA-----DLRGVPCHYETTSEGLK-ATCS-H

R--NL--------TAVP--TNL------TN---DIIVLDLTH-N----------QLT---

------------------K----LTNT-SFSSL-P----HLRYLYLNSNNM---------

------STIEPGAFQA----LPELYSIML---TNNNFTS-------------LPTTI---

-------FSKNLKLELVDLTA--------------------------NRFV---SFP---

--------GNA---LNS-VPSLTE---------------------LRLDQNF-MSSLHFT

GW-------------------------------------------------------RSR

N--------------MTLLLLKTNHF------SS--F--HEDDFLPLKDTRIDLITFF--

------------------------------------NNKITSLQDGLFQHLEGVREMRLT

ANQIRNFS-LHSFLGMSSLETLHMATN-------------------LISAIEPLAPLPNQ

TNLMPNLTSLDLQGN----RIPSIPPRAF----------WGLGNLIRLDIHQSRI-K--T

LQNDSFEGLN----SLEI---------LDLTGN-HLS-----------------------

--YVTKDMFLFSPRLQSLVLPSNWFTELSPKQ----------------------------

-FGDIASLTTLNLARCRIT----DL----------------ILQRGGWNLRNLNFL----

----------DISHN---------------------------------------------

--RLMR-----INKNSFY--GM---A------NLTTLDISN--NR------LLTTI----

----ENGAFA----------------------SIGR---LQILS---------------L

SHLSYLGQLHS-----------------------------------PFTNLN-EL-----

---TILDMSYT---------SVALSYE------------L------------FIG-LSNL

--------------RKLSM-RGSGL-----------------------------------

---------TASSFWDSH------------------------------------------

-----------------HEDLPVLSALSTL-ER----LYIKGN----------KLNKLK-

-----------------------PGTFQG-------------------------------

------------------------------------------------------------

------------------------------------------------------------

--------------------------LQNL---RHLEMD---DSEIS-------------

----SL------------------NEDI----FMNLTSLQS-LFINE----NHIA---EL

T--SRHLADL-SSLFGVSIKSN--EIKGLASDV-FAN--N-P-HLSYLYISHN-HLTTVK

E-GTVL-P-----R---------------------------Q-TLDVSN----NPFSCN-

--CEFT--------------------------------------------W---F--INW

IN--------EA-------------EVSI-----IHPDQTNC---SSGS--LAPF---KN

QPILA-----------------FDP---TEVC------GT--------------------

----------KVWVYIITTFVIVTCIVVCVV--A---YQRRW-LINYKLF----------

-----HLTL--VFLGRPDDHDG--------RERLDY--EYDINLAFDD---D--DEQW-V

RGIL--KPGFE------ERLP----DFD---RIVCG-DDDLPLGMY-YIEAITE-VFEQS

YKSIMVVSNR---AVDNHSF-ISKLRLAVD--QMNEVEL---EK----VILIFKEDIP-D

GRLP--YLVRLFLSKNKPYYRWS-----EDKY--RQRIMWEKLVRELG------------

------------------------------------------------------Y-NKKM

NDILPI------------------------------------------------------

------------------------------------------------------

>Stpu_TLR_75

------------------------------------------------------------

------------------------MTQWMIAMESIVILW---------------------

---------ICCMLASSLTM----------------------------------------

--------------------DSIGHTGVDAA-----DLRGVPCHYDITSEGLK-ATCS-H

R--YL--------TAVP--TNL------TH---DITVLDLTQ-N----------QLN---

------------------K----LDNT-SFTSL-P----HLRYLYLQSNNM---------

------STIESGAFQS----LPELYLISL---LNNNFTS-------------LPTNI---

-------FSKNQKLQIVDLTA--------------------------NQFV---SFP---

--------GNA---LDS-VSSLTQ---------------------LRFDENF-LSRLNFT

GW-------------------------------------------------------RSR

N--------------MTSFVLNDNRF------SS--F--HEDDFLPLKETRIDQISFT--

------------------------------------KNNLTSLQNGLFQHLEGVREMSLT

SNHIHNFS-LHSFLGMSSLETLYVDTN-------------------VILAIKPLAPLLNQ

TNVMPNLTYLDLQGN----RIPSIPPRAF----------WGLGNLIRLDIHQSRI-K--T

LQNDTFQGLE----SLEI---------LDLTGN-HLS-----------------------

--YVTKDMFVFSPRLQSLILSSNWFTELSPKQ----------------------------

----IGDIASLTSLNLARC----GI-------------TDFRTQSRGWNLRNLKSL----

----------DISYN---------------------------------------------

--RLVR-----IDKNSFY--GM---P------NLTTLDISN--NR------LLTTI----

----ENGAFA----------------------SIGR---LQSLS---------------L

SHLSYLGQLHS-----------------------------------PFTNLN-EL-----

---TILDMSYT---------SVALSYE------------L------------FTG-LSNL

--------------RRLSM-RGSGL-----------------------------------

---------TASSFWDSH------------------------------------------

-----------------TEDLPVLSALPTL-ER----LYLKGN----------KLDRLK-

-----------------------PGTFQG-------------------------------

------------------------------------------------------------

------------------------------------------------------------

--------------------------LQNL---HNLEMD---NSDIT-------------

----SL----NEDV------------------FLNLTSLEY-LFIDE----NHIA---EL

T--SRHLTDL-SSLVGVHIKSN--EIKGLASDV-FSN--N-P-HLSYLYISHN-HLTTVK

E-GTVL-P-----R---------------------------Q-TLDVSN----NPFSCN-

--CE-LT-------------------------------------------W---F--INW

IN--------KA-------------EVSI-----IHPDQTNC---SDVS--LAPF---KN

QPILA-----------------FDP---TEVC------GP--------------------

----------KVWVYIITIFVIVTCIMVCVV--A---YQRRW-LINYKLF---------H

LKL--VFLG----RRDDHDGRE-R------LD---Y--EYDINLAFDD---D--DEQW-V

RGIL--KPGLE------ERLP----DFN---RIVCG-DDDLPLGMY-YIEAITE-VVEQS

YKSILIVSNR---AVENHSF-ISKLRLAVD--QMNEVEL---EK----VILIFKEDIP-D

GRLP--YLVRLFLSKNKPYFRWS-----KDKY--GKKIMWENLVRELG------------

------------------------------------------------------Y-NKKM

NDILPI------------------------------------------------------

------------------------------------------------------

>Stpu_TLR_12

------------------------------------------------------------

------------------------------------------------------------

------------------------------------------------------------

------------------------------------------------------------

-------------------------------------------------------LI---

------------------G----VARS------------RIKWLYIEHVDQ---------

------DIITVDFFRP--LYNSRLIYLEL---NVVHLNL-------------QNATP---

-------FSNLNQLIELTLEG--------------------------TDIP---TLE---

--------PEY---FLG-MNKLEI---------------------LHLEHNH--------

------------------------------------------------------------

---------------INQINPNNSF-----------------------------------

------------------------------------------------------------

------------------------------------------------------------

------------------------------------------------------------

------------------------------------------------------------

------------------------------------------------------------

-----------------------------------------------WSTPHVREI----

----------YLGYN---------------------------------------------

--ELKF-----LSRTAFQ--GL---D------NLFTLDITFNTNF---MELVINQY----

----TGGLFN-----------------------------LRYLV---------------V

SNNVI-----RD----------------------------------FLVDAP--------

---YLISLTSS-------------------------------------------------

------------------------------------------------------------

---------ASGDYFRSG------------------------------------------

---------------------ITFQDTPSL-QW----LDLSYS----------NIRSQL-

----------------LWNSLTSTSLFDG-------------------------------

------------------------------------------------------------

------------------------------------------------------------

--------------------------LDNL---AHLELE---GNPIS-------------

----EL------------------LQGM----FRGLFALEF-LDLSD----CEVS---SI

Q--SNVFRGL-SSLRTLSLGGN--KLQRLPFNL-LTFDNN-T-SLETLVLAGN-KFTYFN

Y-SSFE-P-----L---------------------MFTKNLS--IDISQ----NELICN-

--CDIS--------------------------------------------W---L--VKW

LD--------GQ-------------VNVL------NADNTVC---STASATLSSL---RG

KPLLT-----------------FIP---SDLC------GP--------------------

----------NIVLICSTSLAVIVFAATLLL--I---YHFRW-FVRYKLY---------L

LKL--AVIG----YNEIIDARD-H------GD---F--EFDLNIMFME---D--DEHW-V

QEHL--RPVLE------ERLP----NFN---RNAIG-DDDLIPGMY-YFDAVFY-VIEKS

FKTVLLLSRA---AFQDNWF-MKKFRIAFE--QVNDARM---EN----IVVVFLEDIQ-D

AELP--FLVRLYLSERRTYLWWM-----EDER--GQEYFWNELILTLQ------------

------------------------------------------------------RDNVRW

NIMVPPE-----------------------------------------------------

------------------------------------------------------

>Stpu_TLR_32

------------------------------------------------------------

---------------------MMALKECSVLSSLTMLVV---------------------

---------YFLLVFPCLI-----------------------------------------

------------------------------------TIHEDALPTSKLTVESSIEVCSQD

PKLKEAWCDNRRLTSIP-----------QDLAEDIELLSTQY------------------

------------------NNVKALLNS-SFVRY-P----LITTLDLRSNDI---------

------RTLDHTAFYP----LRDLMNLFM---SFNPHLV-------------LPDTG---

------LFRWASKLSILDLSN--------------------------SNMI---------

--------SLPNDTLKW-SKKLER---------------------LQLSRNQ--------

------------------------------------------------------------

---------------FAFINISSCGM-----------------VKNVHLEGNQLAHLS--

------------------------------------------------------------

---------TEFFNLVCDIDFLVLVEN---------------------------------

--PIKSIDPNVIASL----NVRELLIGNSPLTMKVFRNLFMGISCSEIEFVTIIGSNLTA

FPVDFFDSFC--NCPLSF---------LSFYTVGLKA-----------------------

--------------------------------------------------------LSPY

LFSNLTQLDKLSLSSNSIV-----------------TIEPDFF----EGMQDLRIL----

----------KLQSN---------------------------------------------

--NIQQ-----INPYNQT-WNI----------KVQELQLYN--NL---LIEISQSS----

----FLGLKY-----------------------------LTLLD---------------L

SRNKGLTFLEFT----------------------------------AFTGLE-SI-----

---QKIVLSEC---------NIHHLLL----------------------------ETPSL

--------------TSLVL---NNI-------------------------------IERD

YGWDPRESVKHLRSLIYL------------------------------------------

----NLGAMDLGVYDLWFMNVSLFDGMDNL-TT----LYLSKN---------TDLGSYS-

-------------------WGMLPGSFQK-------------------------------

------------------------------------------------------------

------------------------------------------------------------

--------------------------LTTL---QNLNLD---DCNIL-------------

----SL------------------HSHL----FHDLVSLRM-LSLTG----NRIQ---QL

R--YDMLTEL-RQLTRIYLDRN--LLSYLDETI-FSN----NLRLEYLSLADN-KLTRLN

Q-STFR-P-----I-----------------------KNSLS-SLDISE----NPLLCN-

--CDLK--------------------------------------------W---L--LDW

QK----RSPNLT-------------MQHT--------TITIC---SSAS--LAPL---RE

KPLRD-----------------FDP---SNLC------RL--------------------

----------SSTIPCFISLAVICMVVIAVLV-----HHHRW-HLRYKLF---------L

LKL--ALVG----YKEVRDARNHND----------Y--EFDVNVICYD---D--DEEW-I

RDHL--RPALE------EKLP----QFQ---RNVFG-DEDVVPGMH-YLDAVYH-AVTRS

YKTLIVLSRA---AVRDRWF-MLKLRIAMD--HVSDTRT---EF----VVVVFLEDIP-D

DEIP--FMARLYLNDGRPYLYWT-----DDVR--GQEYFWNKLAKNLT------------

------------------------------------------------------I-NLKT

DDLIPNE-----------------------------------------------------

------------------------------------------------------

>Stpu_TLR_97

------------------------------------------------------------

-----------------------MMALKECSVLSSLTTL---------------------

---------VVSFLLVFPCLQ---------------------------------------

------------------------------------TFYGDALPASKLTVGISNQNCYQD

PKLKEALCDNRKLASIP-----------QDLAEDIELLLMQN------------------

------------------NNVRDILNS-SFVKY-P----LITTLDLSFNDI---------

------RALDHTAFYP----LRDLRDLYM---SFNPHLV-------------LPDKG---

------LFRWASKLSILDLSN--------------------------SNMI---------

--------SLPNDTLRW-STKLEK---------------------LQLSRNK--------

------------------------------------------------------------

---------------FAFINISSCGA-----------------VKYVQLDVNQLAHLD--

------------------------------------------------------------

---------AESFNLVCDIDVLLLVEN---------------------------------

--PIRSVDPNVIASL----NVRVLMIGNSPLTLKVFRNLFMGISCSEIEFVSITGSNLTA

IPVEFFDPLC--NCPLSF---------LDLQTVGLKA-----------------------

--------------------------------------------------------LSKD

VFSNLTQLDKLFLSSNSIV-----------------TIEPDFF----EGIQDLRIL----

----------ELQSN---------------------------------------------

--NIQH-----INTYNQT-WNI----------KLQKLNLYN--NL---LTEISQSA----

----FLGLKD-----------------------------LTLLD---------------L

SMNKRLTFLEFT----------------------------------AFTGLE-SI-----

---QKIVLSKC---------NIHHLLL------------E-------------TPALTSL

--------------TLNYIIERDAY----------------------------------W

APKESLKHLRSLIYLNLG------------------------------------------

-------ATHFSVFDMWFLNGSLFDGMDNL-TS----LDLSRN---------TDFGSYS-

-------------------SGLVPAVFQN-------------------------------

------------------------------------------------------------

------------------------------------------------------------

--------------------------LTTL---QNLNLD---DCDIP-------------

----SL------------------HSHL----FDDLVSLHT-LSLKG----NRIQ---QL

R--YDILTEL-RQLTRIELDRN--YLSYLDETI-FSN----NLRLKYLSLADN-KLTRLN

Q-STFK-P-----I-----------------------EKSLS-SLDISK----NPILCN-

--CDLK--------------------------------------------W---L--LEW

QR--------RS-------------LINL-----TEQSRTIC---SSAS--LTPL---RG

KPLRD-----------------FDP---RNLC------RL--------------------

----------SSTIPCLISLAVICMVVIAVLV-----HHHRW-HLRYKLF---------L

LKL--ALVG----YKEVQDARNHND----------Y--EFDVNVICYD---D--DEEW-I

RDHF--RPALE------EKLP----QFQ---RNVFG-DEDLVPGMH-YLDAVDH-AVTRS

YKTVLVLSRA---AVRDRWF-MLKLRIAMD--HVSDTRT---EF----VVVVFLEDIP-D

DEIP--FMARLYLNDGRPYLHWT-----DDVR--GQEYFWNKLAKNLT------------

------------------------------------------------------I-NLKI

DDLIPNE-----------------------------------------------------

------------------------------------------------------

>Stpu_TLR_25

------------------------------------------------------------

-----------------------------VLPDTGLFRL---------------------

---------ARNLTLVHLSNT---------------------------------------

------------------------------------YLKWLPSDTLKWTTRLEDIVCNNN

K---------------------------------LSIINISACG----------------

---------------------------------------TIKNAEFSRNQI---------

------KDLTVEAFSF----VCHTTTLVL---ERNPIKS-------------VDPKV---

--------IASLHVRQLRLGI--------------------------YPLN---------

---------------------------------------------LEVVRNVFIGVAHSE

------------------------------------------------------------

---------------IEELSVKNARL----------KAFPKDFFDPLMDSSLSVLDLS--

------------------------------------------------------------

-DNHVYMLYPLVFSNLTKLDQLNFSNS---------------------------------

--------------------------------------------------------RIDS

IEPNFFDGMG--EIKV-----------LNFNHNHIRH-----------------------

------------------------------------------------------------

-----------------------------------------INPRNQTWNIGVLEL----

----------HLSTN---------------------------------------------

--RLTV-----IYQSAFL--GL---E------NLTFIDLRF--NN---LLVFELTD----

----STDLRR-----------------------------IQTID---------------L

SLCRISRL--------------------------------------QLESL--TL-----

---KSLNLGHT---------RIPLKPS------------------------KSFKHLPFL

--------------EHLDL-SSTGL-----------------------------------

---------GKLNVWDAT------------------------------------------

------------------VNISLFDELFNL-TT----LDLRNN----------HLLGRE-

-------------------GAFTAGVFRQ-------------------------------

------------------------------------------------------------

------------------------------------------------------------

--------------------------LSAL---QELNLQ---DCDVA-------------

----TV------------------NPNV----FKGLGSLQK-LNLAG----NRIK---RL

P--YSAFNGL-EQVTIINLDTN--NVAYLEDRI-FLN----NPKLTSISLASN-NLTHLK

L-STFQ-P-----I-----------------------ISSLS-SLDLSM----NPIVCN-

--CDLK--------------------------------------------W---L--PSF

LH------RQLM-------------LTNA--------DETIC---SESS--LDPL---RA

KPLLQ-----------------FDP---DELC------KR--------------------

----------SIVLFYSLPLATICLIVIIVLV-----HHYRW-KVKYKIF---------L

LKL--AALG----YNELRDARDHSD----------F--EFDLNIIFYD---D--DEEW-I

REHL--RPALK------AQLP----QFQ---RNVFG-DEDLVVGMH-YLDSVDY-VVSHS

YKTIILLSRA---AVHDRWF-MLKLRTAMD--HVSDTQT---EF----VVVVFLEDIP-D

EEIP--FLARLYLSDGRPYLYWT-----DDVR--GHGYFWHGLTKYLT------------

------------------------------------------------------I-NLRT

NDWIPNE-----------------------------------------------------

------------------------------------------------------

>Stpu_TLR_10

------------------------------------------------------------

------------------------------------------------------------

------------------------------------------------------------

------------------------------------------------------------

------------------------------------------------------------

----------------------------------------LTTLNLSENPH---------

--------------------------------LGNG----------------LPPFI---

------------------------------------------------------------

------------------FRKLSV------------------------------------

------------------------------------------------------------

---------------LQELRIEFCYI----------------------------------

------------------------------------------------------------

------------------------------------------------------------

------------------------------------------------------------

------------------------------------------------------------

------------------------------------------------------------

------------------------------------------------------------

------------------------------------------------------------

-------------------------------------------SH---------------

---------------------------------------FHHLT----------------

------------------------------------------------------------

------------------------------------------------------------

------------------------------------------------------------

------------------------------------------------------------

------------------------------------------------------------

------------------------------------------------------------

------------------------------------------------------------

------------------------------------------------------------

------------------------------------------------------------

--------------------------------FLGLISLQK-LSLRG----NNIY---QL

H--QDLLQGL-VQVRRMHLQGN--FLSYLDEAI-FSN----NAKLTTLSLANN-KFTSFN

Q-STFQ-P-----I-----------------------KLSLS-SIDLSN----NPINCN-

--CDLK--------------------------------------------W---L--LDW

LR------GPIQ-------------LLNE--------DETIC---SLAS--LDSV---GE

KPLLD-----------------FDP---NELC------RL--------------------

----------NIVLICVLSLAAICFVVVVGFV-----YHNRW-QIRFKLY---------L

LKL--AVLG----YNELRDARDHND----------Y--EFDLNIIFYD---D--DEHW-I

REQL--QPVYE------ERLP----QFE---RNVFG-DDDLVPGMH-YLDSVDY-VVSRS

YKTVIVLSRA---AVRDRWF-MLKLRIAMD--HVSDTQT---EF----VVVVFLEEIP-D

DEIP--FLARLYLSDGRPYFHWP-----NEER--GQEYFWNELVKSLT------------

------------------------------------------------------I-NLRT

NDLIPNE-----------------------------------------------------

------------------------------------------------------

>Stpu_TLR_47

------------------------------------------------------------

------------------------------------------------------------

------------------------------------------------------------

------------------------------------VLTIEELRNIFIGMSRSLIEIF--

--------------------------------TLTNGSDLSV------------------

-----------------------VPKD-LFDALRDC---SLRILDLSMNQL---------

------RAPYRRVFSK----LTSLRQFHL---RGNGIVT-------------IEPDF---

-------FEGMDNLRVLDLKS--------------------------NHIK---------

--------DINTDNVSW-TIDLIE---------------------LHLSGNA-LTTIRES

------------------------------------------------------AFRGLH

N--------------LTLLDLRSNEL----------------------------------

------------------------------------------------------------

------------------------------------------------------------

------------------------------------------------------------

---------------------------LKI------------------------------

------------------------------------------------------------

------------------------------------------------------------

------------------------------------------------------------

-----------VWATSFL--EL---K------SIQTIDLSG--CMIRPFQVMIPSS----

---------------------------------------VTSLF---------------L

NYMVI------------------------------------------------PL-----

---NFVNLTTS------------------------------------------FKHLQLL

--------------EHLEMKESDLM-----------------------------------

--------NNNLWNPDVN------------------------------------------

--------------------VSLFDGLFNL-KT----LDLSIN----------QLYIHL-

----------------DVKGMLPPWILRQ-------------------------------

------------------------------------------------------------

------------------------------------------------------------

--------------------------LSTL---QYLNLR---TCFFS-------------

----NI------------------HPQA----FIALKSLRV-LILSY----NQMH---QL

P--VDLFKPL-DQVTSIDLANN--FLTDLDGAI-FLN----NPLLETLLLSNN-KLIRLE

Q-NAFK-P-----L-----------------------YSSLV-FIDLSM----NPIDCN-

--CDLS--------------------------------------------W---F--LDW

LN------GPLR-------------LTFL--EDDFDDDKTIC---SSAS--LEPL---RG

KHLID-----------------FDP---REFC------SI--------------------

----------NITLVCLPTLAIICLIFIVALV-----YHYRW-PLRYRLF---------L

VKL--AAVG----YEEMRDARDHND----------Y--QYDLNVIFYD---E--DEEW-I

REHL--RPALA------ERLP----QFQ---RNVFG-DEDLVLGMH-YLESVDY-VVSHS

YKTVVVLSSA---AVLDRWF-ILKFRTAMD--HVSDTLT---EF----VVVVFLEDIP-D

DEMP--FLARLYLSDGRPYIHWT-----EDVR--GHEYFFDKLTKSLT------------

------------------------------------------------------I-NLRT

NDRIPNV-----------------------------------------------------

------------------------------------------------------

>Stpu_TLR_15

------------------------------------------------------------

--------------MYRPIIVSALKQESVIMAFKHPLLI---------------------

---------LHLVISLTQAL----------------------------------------

------------------------------------NGVAALVPSRSLHLDTSLQGCDED

LERKKVFCGDRGFISVP-----------QHLPEDTEGLYLDS------------------

------------------NNIRSLLNS-SFRRY-P----LITDLNLFFNDL---------

------TVIEFRSFTP----LKNLTHLDI---SGNLFMV-------------LPCSF---

-------FRWARKLSLLYVSE--------------------------SILR---SFP---

--------NDI---LKW-SPNLNT---------------------VDLSNNW--------

------------------------------------------------------------

---------------LTVINISSCGTARH-------------------------------

------------------------------------------------------------

------------------------------------------------------------

------------------------------------------------------------

---------------------------VDLSYNRIHN-----------------------

------------------------------------------------------------

------------------------------------------------------------

------------------------------------------------------------

-----------LTEDAFK--FV---C------NSDTLDLKV--NT---IRSVDPNV----

----ITSLN------------------------------VRSLI---------------I

GWRVL-----------------------------------------NIEELR-NI-----

----FIGISRS-------------------------------------------------

------------------------------------------------------------

---------SVIENFTFT------------------------------------------

------------------------NPINSV-TV--------------------DTEDLP-

-----------------------PGVLNQ-------------------------------

------------------------------------------------------------

------------------------------------------------------------

--------------------------LSAL---QNLSLE---DCDLS-------------

----NL------------------HPQA----FTGLKSLRV-LILRN----NKLL---QL

H--VDLFKPL-DLVTSIDLVDN--VLMDLDGPI-FLS----NRKLKTLLLSNN-RLTRLE

Q-YTFK-P-----L-----------------------YSSLL-FIDLSM----NPIDCN-

--CGLS--------------------------------------------W---F--PKW

LS-----GPLNL-------------IEDN---------ITIC---SSDS--LEPL---RG

EYLID-----------------FDP---RELC------SI--------------------

----------NIALVCLPTLAIICLIFIVAL--V---YHYRW-PLRYRLF---------L

VKL--AVLG----YKEMRDARGHID----------Y--EFDLNVIFYD---D--DEEW-I

REHL--RPALA------ERLP----QFQ---RNVFG-DADLVLGMH-YLDSVDY-VVSHS

YKTIIVLSSA---AVLDRWF-ILKFRTAMD--HVSDTLT---EF----VVVVFLEDIP-D

AEMS--FLARLYLDDGRPYIHWT-----EDVR--GHEYFWDKLTKILT------------

------------------------------------------------------I-NLRT

NDRIPNE-----------------------------------------------------

------------------------------------------------------

>Stpu_TLR_74

------------------------------------------------------------

------------------------------------------------------------

------------------------------------------------------------

------------------------------------------------------------

------------------------------------------------------------

----------------------------------M-------------------------

------FLLPTTSFKN----VELLERLEM---IHSRLLI---------------------

-----------------------------------------------GNLW---------

--------NPR-------------------------------------------------

------------------------------------------------------------

------------------------------------------------------------

------------------------------------------------------------

------------------------------------------------------------

------------------------------------------------------------

------------------------------------------------------------

------------------------------------------------------------

------------------------------------------------------------

------------------------------------------------------------

-----------KNVSLFD--GL---F------NITALDLSK--NP---------------

------------------------------------------------------------

----------------------------------------------IYSATR--------

---NTEDLPPG-------------------------------------------------

------------------------------------------------------------
[truncated: 241,871 more chars]
