# Supplementary material for: Evolutionary History of the Toll-Like Receptor Gene Family across Vertebrates
Source: Genome Biol Evol. 2019 Dec 4;12(1):3615–34. doi: 10.1093/gbe/evz266 (PMC6946030; doi:10.1093/gbe/evz266)
Supplement: evz266_Supplementary_Data [file evz266_supplementary_data.zip › Supplementary text S2.docx]

Supplementary text S2. Vertebrate TLR amino acid alignments.

>TLR15_Alsi

------------------------------------------------------------

------------------------------------------------------------

------------------------------------------------------------

------------------------------------------------------------

------------------------------------------------------------

------------------------------------------------------------

---------------MGI------L--I--------------------------------

--------------------------------L---R--F--Y--L------IA----F-

---LFNGADGFQ----------------------------------------T--QR---

-TSY-MYG----------------------------FKFSNYS-----------------

-----------Y------LN--LS-S-------IH----E-A-QA-----P-------K-

T--SRVL-------NFSHN-------V---I--E-----------KITRRDFEG--F-VA

L-----------EVLDLS-YNQ------------------I-QD--IEP--GAFENLLGL

VSVNLS------F--------ND-Q-LH-----------------------RI---P-YL

------A-PHL---TF---LQTGETS-GI---PQH-NIYFE------RS---S---EA--

----AL-ESF------------V-------------------------------------

-----------SAEKQRYPE-DSH-GLVNGHSKFSRTVQD-LQGVDENRTVFPSS----T

LRHKICGKPINGVLDLSKSN-LS----EE--E------------LTE---KMD----ASH

CK-GHLD-----SV--------VELNISH------------------------NNLE---

-IDL--LS----L----FIL---------------LLPM----ENAQ----SID---A--

S--YN---K-I-T---ISN-------IE----LGKI-------C--DFPIRRLLF----V

NIS----------NNPLNSLDTVC---LP-S----------T-IKIIDL--SYT-NI-N-

-HI-PK--NFHKKLFN---------------------LE---R---IYV--QGN-----Q

-----------------F-I---Y--T-------V--NS-DDS----------G-----K

NV----S---------K--SQ----PGTVRIAA-LSL-----------------------

-------------------------------------------------VNTREGTPI--

-------------------------------ESLPE------------------------

---------------------------K--VKY-------LKMSNCSIVEL-PEWF--AR

TMKK--LLFLDLSNNP--------I-S-K------L--------------PDLP--SSLQ

HLDLSNSDIKIIPPSFKS--L-SN-LTVFKIQSNK-------------I--T-DF-----

-SPAYFL--LT-LTEYDVSK-N-K--------------------------L-K---V---

--------LN----------L-N--ENL------------------R-------------

--KAEFLNISGNV--I----T-QI--------------DTTSPLS---------------

ALTNL----------DGS-------HNLI----SE--L-----PDH-----FA--E-FLP

----------------------------------------------------------V-

---------------------LKYFNLSGNKISFL-QPGS--LP-ESLVELDISNNAITT

IVEE---------T-FG------------HLTK-------------LNVLTVQ-GKHFFC

NCDL--Y-WF----V--N--------------T----Y-------IH-----------SP

-------Q-----------LL--------IN-G-----RESLRC-SFP-PD----KR---

-GALVEKS--NLTLVH-C-SFGI-----QMAIT--ACPAILIMSVITSL----C--WHFD

G-PW-YIRMG---WY---W-----CM----A--K------------------R-------

-------------RQY---------------E----KR----P---ESK---A----YDA

FISYSENDA---SWTKENLLEKL----ETK---G--------------------------

---F---KICYHERDFK----------------P--GH-PVLGNIF-YCIENSHKVLFVL

SP------SFV-----------NSC-------WCQY-ELYFAEHRVLN--E-N-QDSLIM

VVLE--D-LPPNSV----------------------------------------------

--PQKFSKLRK-LLKRKTYLKWS----PE-------EQKQK-------IFWHQLTAVL--

--KTS------------ND-----------------------------------------

-------------------PIVLKAE-------NG-----LN--QDTYEMECH-------

-----------------------------------------------------------

>TLR15_Gagan

------------------------------------------------------------

------------------------------------------------------------

------------------------------------------------------------

------------------------------------------------------------

------------------------------------------------------------

------------------------------------------------------------

---------------MGI------L--IRV--L---------RF----------------

---------------------------------------Y--F--I------AF----L-

--------------------------------------------FNGANGFQT--QR---

-TSY-MYG----------------------------FTFSNYS-----------------

-----------Y------LN--LS-S-------IH----E-A-QA-----P-------K-

T--SKVL-------NFSHN-------I---I--E-----------KITRRDFEG--F-VA

L-----------EVLDLS-YNQ------------------I-QD--IEP--GSFENLLSL

VSVNLS------F--------NDQL-HR-----------------------IP---YLAP

------HLTFL---QT---GGTSGIP-QH---NIY-FERSS-----------E---AA--

----LE-SFV------------S-------------------------------------

-------------AEEQQYP-E-G-LYGLV-----------------NGHSKFSR---TV

RDLQEVDENRT---VLLSPP-LR---------------------HKI---CGK----PIN

GV-LDLS-----KS---NLSEEELTEKMDASHCKDHL---------------DSVVE---

-LNI--SH----N----NLE--IDLLSLFIL-LLPMENA----QSID----A-----S--

Y--NK-----ITI---SNI-------ELG---KICD-------F--PI--HRLLF----V

NIS----------NNPLNSLDTVC---LP-S----------T-IKIIDL--SYT-NI-N-

-HI-PK--NFHEKLFN--------------------------------L--ERI-----Y

-----------------V-Q---G--N-------Q--FI-YTV------NSDDS-----G

KN----V---SEPKPGTVRIAT---LSFVNTREGTPI-----------------------

------------------------------------------------------------

-------------------------------ESLPE------------------------

---------------------------N--VKY-------LKMSNCSIVEL-PEWF--AR

TMKR--LLFLDLSNNP--------I-------------------------SKLP------

----------------DL----PSSLQHLDLSNSD-------------I--K-----IIP

-PSFKSL--SN-LTVFKIQS-N-K--------ITDFSPEYFLLTL-----T-E---YDVS

KN-----KLK----------V-L--NLNEN----------L-----R-------------

--KAEFLNISGNV--I----T-----------------QIDTTSP------LS-------

ALTNV----------DGS-------HNSI----SE--L-----PDH-----FA--K-FLP

----------------------------------------------------------V-

---------------------LKYFNLSGNKISFL-QPGSLP---ESLVELDISNNAITT

IVEE---------T-FG------------HLTK-------------LNVLTVQ-GKHFFC

NCDL--Y-WF----V--N--------------T----Y---IH--SP-------------

-------Q-----------LL--------IN-G-----RENLRC-SFP-PD----KR---

-GALVEKS--NLTLVH-C-SFGI-------QMAITACAAILIMSVITSL----C--WHFD

G-PW-YIRMG---WY---W-----CM----A--K--------------------------

-----------R-RQY---------------E----KR----P---ENK---T----YDA

FISYSENDA---SWTKENLLEKL----ETK---G--------------------------

-F-----KICYHERDFK----------------P--GH-PVLGNIF-YCIENSHKVLFVL

SP------SFV-----------NSC-------WCQY-ELYFAEHRVLN--E-N-QDSLIM

VVLE--D-LPPNSV----------------------------------------------

--PQKFSKLRK-LLKRKTYLKWS----PE-------EQKQK-------IFWHQLTAVL--

--KTS------------ND-----P-------IVLK------------------------

------------------------AE-------NG-----LNQDTYEMECH---------

-----------------------------------------------------------

>TLR15_Tagu

------------------------------------------------------------

------------------------------------------------------------

------------------------------------------------------------

------------------------------------------------------------

------------------------------------------------------------

------------------------------------------------------------

---------------MRILPGYLHFYFIPF--L---------------------------

---------------------------------------LSGARGF------LM------

-------------------------------------------------------WR--T

-PPA-F-------------------------------LVYNYS-----------------

-----------Y------SN--LS-S-------VS----E-A-QA-----P-------K-

T--ARAL-------NFSHN-------V---I--E-----------KVTKGELEG--F-DW

L-----------EVLDFS-YNQ------------------I-RA--VEP--GVFQSLLSL

VSVDLS------F--------NDEK-LL-----------------------LS---D---

------LPSHLRLSPAGKAPRSLQIS-RN---SGR-SSGVA------LQ---P---SAPA

EEPSHSGVLS------------L-------------------------------------

-------------LQILSPRLR-R-STGNLLRRG-------------ERNTTALPMVTPE

PTLCGTPINGTL--NLSHSN-LT----QD--E-------------------------LVL

KLDEDLC-----QA------------------------------------HLRRILE---

-LDI--SH----N----NVE--MDLLSLFSL-FFPMENT----LSID----A-----S--

S--NK-----------LTINILNPESFC----NFPS--------------HQLLF----L

NIS----------NNPINSLDRLC---LP------------SSIKEIDL--SFT-NI-S-

-QI-PL--DFAKKLFN--------------------------------L--EKM-----Y

-----------------VQG---N--H-------F---IYTAFSESGNTLPTCV---PPP

GT----V---H--------LNA---LSLVRNKAGTPV-----------------------

------------------------------------------------------------

-------------------------------ESLPE------------------------

---------------------------K--VKH-------LGMSNCSIVEL-PEWF--AD

TVEE--LLFLDLSSNH--------I-------------------------SVFP------

----------------NF--P-PS-LQHLDISSND-------------I--K-----VIS

-SSLKSL--SN-LKIFRIQN-N-K--------IMGIHTEFFPSALKKCDFS-K--NKVKV

LS---LTSAL------------------------------------E-------------

--KLEHLNISGNL--I----T-----------------RLEPAGH------LP-------

ALTNL----------DSS-------HNLI----PE--L-----PDG-----FG--V-SLP

----------------------------------------------------------G-

---------------------LKYFNLSGNKISFL-QRGSLP---ASLVELDISDNAITT

IVEA---------T-FS------------PLTS-------------LRLLTVQ-GDHFFC

TCDL--Y-WF----V--N--------------V----Y---LHE-PQ-------------

-------L-----------EI--------RG-------REAMRC-SFP-SE----RR---

-GSPVGSS--RLTLLR-C-SLGV-------QLAVTAGAASLAVLALTAL----C--WRLD

G-PW-YIRMG---WY---W-----CM----A-----------------------------

-------------KRK--------------------QYEKRPE---NKL--------FDA

FISYSEHDA---DWTKENLLKTL----END----------------------------G-

-F-----RICYHERDFK----------------P--GH-PVLGNIF-YCIENSHKVLFVL

SP------SFV-----------NSC-------WCQY-ELYFAEHRVLN--E-N-LDSLIM

IVLE--D-LPSHSV----------------------------------------------

--PQKFSKLRK-LLRRKTYLKWS----LE-------EHKQK-------MFWHQLKAVL--

--KTT------------NE-----------------------------------------

------------------------PL-------VR-----AENRSALEMRELE-------

-----------------------------------------------------------

>TLR15_Coli

------------------------------------------------------------

------------------------------------------------------------

------------------------------------------------------------

------------------------------------------------------------

------------------------------------------------------------

------------------------------------------------------------

--------------MKPL------IGSFHF--Y---------------------------

------------------------------------------FISF------LL--SRA-

-------------------------------------------------------KG--L

-PAL-R-----------------------APTASVSPFYTYCY-----------------

------------------LN--LS-S-------VA----E-A-QA-----P-------K-

T--ARAL-------NFSHN-------L---I--K-----------KITKRDLEG--F-DA

L-----------EVLDLS-HNQ------------------I-RD--IEP--GAFERLPSL

LSVNLS------F--------ND---KS-----------------------LL---A-AG

------LPPPLKLNPAREASGLLQLY-EY-------FDRSS-----------E---AA--

----LEPPAP------------A-------------------------------------

-------------EERPSVP-K-P-LPARL-----------------RRGTGGVP----P

GAEGNVALSPN-------PD-PC--------A-------------------------APV

SGRLDLS-----HR---PLSEDELAAMLDEDRCLARL---------------ESILE---

-LDM--SH----S----GLQ--MDLLSLFAL-FLPMKNV----RSID----A-----S--

H--NK-----------LTMNVLDASSFC----KFPS--------------SKLLF----L

NIS----------NNPINSLDTLC---VP------------STIQVIDL--SFT-NI-S-

-QI-PR--NFAKKMTN---------------------LE---H-----L--YAQ-----G

------------N----H----------------F---I-YTVRPETPSVAAEY---PPG

SV--------------H--INA---ISLVWNEAGTPI-----------------------

------------------------------------------------------------

-------------------------------ESLPR------------------------

---------------------------R--VKH-------LQMSNCSIVEL-PEWF--AS

TMQE--LLFLDLSSNR--------I-------------------------SALP------

----------------EL----PASLRHLDLSNSD-------------I--K-----IIP

-PRFKSA--AN-LTVFNIQN-N-K--------ITDMHPEYFPLALTECDIS-K--NKLTV

LS---LTGAL------------------------------------E-------------

--KLEFLNVSGNL--I----A-----------------PPAPLPP---------------

-PPTL----------APS-------HNLI----AE--L-----PDH-----FG--E-SLP

----------------------------------------------------------A-

---------------------LKYFNLSGNKISFL-QRGSLP---ASLLELDISNNAITT

IVED---------T-FG------------PLTS-------------LSVLTVQ-GKHFFC

NCDL--Y-WF----V--N--------------V----Y---MRR-PG-------------

-------L-----------RI--------RG-------REALRC-SFP-PR----RR---

-GWLVELL----------------------------------ELLLAAL----C--RRFH

G-LW-YVRMG---WY---W-----CM----A-KR------------------R-Q-----

-----------YEKRP--------------------------E---NKL--------FDA

FVSYSEQDA---GWTKEYLLEKL----EAD----------------------------G-

-F-----RICYHERDFK----------------P--GH-PVLGNIF-YCIENSHKVLFVL

SP------SFV-----------NSC-------WCQY-ELYFAEHRVLN--E-N-QDSLIM

IVLE--E-LPADSV----------------------------------------------

--PQRFSKLRK-LLKRKTYLKWS----PQ-------EHKQK-------MFWRQLEAVL--

--KTT------------NE-----------------------------------------

------------------------PL-------VR-----AENGSAGDAYEME-------

-----------------------------------------------------------

>TLR15_Meun

------------------------------------------------------------

------------------------------------------------------------

------------------------------------------------------------

------------------------------------------------------------

------------------------------------------------------------

------------------------------------------------------------

---------------------------MRI--L---------------------------

---------------------------------------IGSLHFY------SI--SFF-

----------------------------------------------------LSGAN--G

-FLT-E-----------------------GTPTAYTFPFYNYS-----------------

-----------Y------LN--LS-S-------IS----E-A-GA-----P-------R-

T--ARAL-------NLSHN-------A---I--E-----------KVTKRDLEG--F-ST

L-----------EVLDLS-YNR------------------I-KD--IEP--GAFESLPSL

TAVDLS------F--------ND---KE-----------------------LV---V---

--------SGL--------PAHVKLTPTT-------KPSEAAPEPSAPP---E---EL--

----FHAAIL------------P-------------------------------------

-------------DPGPRLGWSTA-NVPRRGEETTVSSSRGGGNGTVAPTASPRPSSCGE

PVNGTLDLSNS---KLSEEE-LM---------------------------LKL---DPDL

CQ-AQLD----------------------------------------------HIVE---

-LNI--SH----S----ALE--MDLLSLFLL-FLPIKNM----QSVD----A-----S--

Y--NK-----------LTINILDLDSICD---FPSE----------KLWFVNLSH-----

--------------NPINSLNALC---LP------------TTIRVIDL--SFT-NI-S-

-QI-PR--NFARKMLN---------------------LE---H-----M--YVQ-----G

------------N----H----------------F---I-YTV-----QPQQPN---PLA

KPAHGCF---H--------ISA---LSLVRNQAGTPI-----------------------

------------------------------------------------------------

-------------------------------ESLPK------------------------

---------------------------N--MKH-------LEMSNCSIVEL-PEWF--AG

TMNA--LLYLDLSSNR--------I-------------------------AALP------

----------------DL--P-PS-LQHLDISSSD-------------I--K-----VIP

-PGFRSM--AN-LTVFVIHN-N-K--------VTEMHPEYFPLTLTKCDIS-K--NKLSL

LP---LTEAL------------------------------------Q-------------

--NLQHLNVSGNL--I----TRLE--------------ATSLLP----------------

ALTNL----------DSS-------HNLI----AE--L-----PDD-----FG--A-SLP

----------------------------------------------------------M-

---------------------LKHFNLSGNKISFL-QRGALP---ASLLELDISDNAITT

IVED---------T-FG------------RLTQ-------------LSLLTVQ-GKHFFC

NCDL--Y-WF----V--N--------------V----Y---IHS-PQ-------------

-------L-----------HI--------RG-------KESLIC-SFP-PD----RR---

-GQLVESS--HLTLLH-C-SLGI-------QMAITACAAILLVLVLTGL----C--WRFD

G-LW-YVRMG---WY---W-----CM----A-KR--------------------------

-------------KQY--------------------------E---KRPE-NK--P-FDA

FISYSEPDA---EWTKENLLGKL----ERD----------------------------G-

-F-----KICYHERDFK----------------P--GH-PVLGNIF-YCIENSHKVIFVL

SP------SFV-----------SSC-------WCQY-ELYFAEHRVLN--E-N-QDSLIM

IVLE--E-LPPHSV----------------------------------------------

--PQKFSKLRK-LLKRKTYLKWS----PE-------EHKQR-------MFWRQLEAVL--

--RTT------------NE-----------------------------------------

------------------------PL-------LR-----AENGPAQDACEMEGCTEV--

-----------------------------------------------------------

>TLR15_Tigu

------------------------------------------------------------

------------------------------------------------------------

------------------------------------------------------------

------------------------------------------------------------

------------------------------------------------------------

------------------------------------------------------------

------------MRILIVGRLHFYLISLLF--S---------------------------

-------------------------------ILFSTANGL-----L------TW------

-------------------------------------------------------RS--S

-RMF-F-------------------------------QFYDYS-----------------

-----------Y------SN--FS-S-------VS----E-A-QA-----P-------Q-

T--ARVL-------NLSHN-------M---I--E-----------NITKRDFEG--F-DA

L-----------EVLDLA-YNQ------------------L-KY--VEP--GVFENLLNL

VSVNFS------F--------ND---KQ-----------------------LD---V---

--------LGL--------ASRLKLLPTN-----EASRILQLSKYFEIS---S---EP--

----AQESPV------------S-------------------------------------

-------------AQELQRS-E-G-PADQL-----------------PVNARVRRSNGDS

QRAEKNVTVSPIAGLM--TN-VC--------G-------------------------TPI

NGILDLS-----ER---KLSEEELQEKLQADLCQAQL---------------DSVLE---

-LNI--SH----N----NLE--IDLLSLFVL-FLPMKNV----QSID----A-----S--

Y--NK-----------ITINNIDVEAICH--------------F--PF--HNLFF----L

NIS----------NNPMNRLDTVC---LP------------STIKVIDL--SFT-NI-S-

-QI-PK--NFAKKLFN--------------------------------L--EKM-----Y

-----------------VEG---N--H-------F---I-YTV-----SPENAD---ANQ

KPKPGTV---R--------INA---ISLVRNEAGTPI-----------------------

------------------------------------------------------------

-------------------------------ESLPE------------------------

---------------------------K--VKH-------LKMSNCSIVEL-PEWF--AG

RMTK--LMLLDLSSNP--------V-------------------------STFP------

----------------HL----PTSMQHLDISNSD-------------I--K-----VIP

-PSFKFL--SN-LTIFNVQN-N-K--------ITDMPLEYFPLTLTKCDIS-K--NKLQV

LS---LTETL------------------------------------R-------------

--KLEYLNVSGNQ--I----T-----------------TLEPRKQ------LS-------

AITNL----------DGS-------HNLI----AE--L-----PDH-----FG--E-ILP

----------------------------------------------------------T-

---------------------LKYLNLSGNKISFL-QSGSLP---VSLIELDISNNAITT

IVED---------A-FG------------QLTS-------------LSVLTVP-GKHFFC

NCDL--Y-WF----VNVY--------------IHNPYL----------------------

-------------------QI--------IG-------RENLRC-SFP-PD----RR---

-GLLVEHS--NLTLLH-C-SLGV-------QMAITACVAVLIVLVLTGL----C--WRFD

G-LW-YVRMG---WY---W-----CM----A-----------------------------

-------------KRK--------------------EYERRPE---NKL--------YDA

FISYSNEDA---DWTKENLLEKL----EAK----------------------------G-

-F-----KVCYHERDFI----------------P--GH-PVLGNIF-YCIENSHKVIFVL

SP------SFV-----------NSC-------WCQY-ELYFAEHRVLN--E-N-QDSLIM

IVLE--E-LPFNSV----------------------------------------------

--PQKFSKLRK-LLKRKTYLKWS----PE-------QAKQK-------VFWHQLTAVL--

--KTA------------NEPLVVRAENGAAHNTYEME-----------------------

------------------------------------------------------------

-----------------------------------------------------------

>TLR15_Rham

------------------------------------------------------------

------------------------------------------------------------

------------------------------------------------------------

------------------------------------------------------------

------------------------------------------------------------

------------------------------------------------------------

-----------MRILLGK------LHFYLMSFL---------------------------

---------------------------------------FGRVDGF------LT------

-------------------------------------------------------WRTSG

MYAF---------------------------------QFYNYS-----------------

-----------Y------LN--LS-S-------VS------E-QA-----P-------K-

M--ARVL-------NFSHN-------M---I--E-----------KITKRDFEG--F-DA

L-----------EVLDLA-YNQ------------------I-KD--IES--GALENLPSL

ISVNFS------F--------ND---KK-----------------------LD-------

------V-SGL---TSHKLLPSGEVS-GVLQLYKY-FETPS-----------E---TA--

----LESSVS--------------------------------------------------

-------------AKGLKHSEG---PSGRL-----------------HANPRFRRSKQPP

RAGKNVTVSPT-------AE-LK----PN---------------FCG----------TPI

NGILDLS-----MSKLSEEELMKILNPDLCQAQLDSI-----------------------

-LEL-------------NISHSNLEMDLLSL-FILFLPM----ENVQ----------SID

ASYNK-----------ITINNIDVEAICH--------------F--KF--HSFFF----L

NIS----------NNPMNNLDTIC---LP------------STIKVIDL--SFT-NI-S-

-QIPQNFAKKLFNLET--------------------------------MYVQGN------

------------H---------------------F---IYTASPENTNENQKLA------

PG--------------TVRISA---ISLVRNEAGTPI-----------------------

------------------------------------------------------------

-------------------------------ESLPK------------------------

---------------------------K--VKH-------LKMSNCSIVEL-PEWF--AG

TMKE--LLLLDLSSNP--------V-------------------------STFP------

----------------YL----PISLQHLDLSNSD-------------I--K-----VIP

-PRFKSL--FN-LTIFNIQN-N-K---------ITDMPLEYFPLTLTKCDISKNKLQVLS

LT-----------------------ETL------------------K-------------

--KLEYLNVSGNL--I----S-----------------KLEPTGQ------FS-------

AITNL----------DSS-------HNLI----AE--L-----PDH-----FG--K-SLP

----------------------------------------------------------A-

---------------------LKYLNLSGNKISFL-QSGSLP---ASLIELDISNNAITT

IVED---------T-FG------------QLTS-------------LSVLTVE-GRHFFC

NCDL--Y-WF----VNIY-------------------I-------HN-------------

-------------------PF--------LHIN-----GRENLC-SFP-PE----RR---

-GLLVESS--NLTLLH-C-SLGI-------QMAITACVAILVVLVLTGL----C--WHFD

G-LW-YVRMG---WY---W-----CM----A-----------------------------

------KRKQYEKRPE------------------------------NKL--------YDA

FISYSEHDA---DWTKENLLEKL----ELK----------------------------G-

-F-----KICYHERDFK----------------P--GH-PVLGNIF-YCIENSHKVLFVL

SP------SFV-----------NSC-------WCQY-ELYFAEHRVLN--E-N-QDSLIM

IVLE--D-LPFNSV----------------------------------------------

--PQKFSKLRK-LLKRKTYLKWS----PE-------QHKQK-------IFWHQLTAVL--

--KTT------------NEPLVLRAEHGSTQDMYEME-----------------------

------------------------------------------------------------

-----------------------------------------------------------

>TLR15_Drno

------------------------------------------------------------

------------------------------------------------------------

------------------------------------------------------------

------------------------------------------------------------

------------------------------------------------------------

------------------------------------------------------------

------------------------RMRILIGKL---------------------------

-------------------------------HFYLISFLFSRANGF------LT------

-------------------------------------------------------WRTSR

VYVS---------------------------------QFYNYS-----------------

-----------Y------LN--LS-S-------VS----E-A-QA-----P-------K-

M--ARVL-------NFSHN-------M---I--E-----------KITKRDFEG--F-HA

L-----------EVLDLA-YNQ------------------I-QH--IEP--GVFENLLSL

VSVNLS------F--------ND---KK-----------------------LD---A-SG

------LASHLKLLPTVEAPGVLQLY-KY-------FETSP-----------E---AV--

----LESSVS--------------------------------------------------

-------------AKELQHSEG-PSEQLNV-----------------NPSFRRSK---RD

PQRAEKNVTVSPTAGL--KPNLC--------A-------------------------TPI

NGILDLS-----KR---KLSEEELTEKLDADLCQAQL---------------ESVLV---

-LNI-SHS---------NLE--MDLLSLFIL-FLPMKNI----QSID----------A--

---SY-----------NKI-------------TINN-------I--DVEACHFPF----H

SLF-----FLNISNNPMNYLDTIC---LP------------STIKVIDL--SFT-NI-S-

-QIPQNFAKKLFNLEN--------------------------------MYVQGN------

------------H---------------------F---IYTASPENTNANQKLA------

PG--------------TVRINA---ISLVRNDAGTPI-----------------------

------------------------------------------------------------

-------------------------------ESLPE------------------------

---------------------------K--VKQ-------LKMSNCSIVEL-PEWF--AG

TMKE--LLLLDLSSNP--------V-------------------------SMFP------

----------------HL----PTSLQHLDISNSD-------------I----KI----P

-PSFKSL--SN-LTIFNIQN-N-K---------ITDMPLEYFPLTLSKCDISKNKLQVLS

LT-----------------------ETL------------------K-------------

--KLEYLNVSGNL-------------------------ITKLEPT-----QLS-------

AITNL----------DSS-------HNLI----AE--L-----PDH-----FG--K-SLP

----------------------------------------------------------T-

---------------------LKYLNLSGNKISFL-QNGSLP---ASLIELDISNNAITT

IVED---------T-FG------------RLTS-------------LSVLTVQ-GKHFFC

NCDL--Y-WFVNIYMHNT--------------Y----L----------------------

-------------------QI--------NG-------RENLRC-SFP-PD----RR---

-GSLVENS--NLTLLY-C-SLGV-------QMAITACVAILIVLALTGL----C--WHFD

G-LW-YVRMG---WY---W-----CM----A-----------------------------

------KRKQYEKRPE------------------------------NKP--------YDA

FISYSEHDA---NWTKENLLEKL----EAK----------------------------E-

-F-----KICYHERDFK----------------P--GH-PVLGNIF-YCIENSHKVLFVL

SP------SFV-----------NSC-------WCQY-ELYFAEHRVLN--E-N-QDSLIM

IVLE--D-LPFNSV----------------------------------------------

--PQKFSKLRK-LLKRKTYLKWS----PE-------QHKQK-------VFWHRLTAVL--

--KTT------------NEPLVLRAENGSAQDMYEME-----------------------

------------------------------------------------------------

-----------------------------------------------------------

>TLR15_Apau

------------------------------------------------------------

------------------------------------------------------------

------------------------------------------------------------

------------------------------------------------------------

------------------------------------------------------------

------------------------------------------------------------

------------------------MRILIGKLH---------------------------

-------------------------------FYLISFLFSRANGFL------AW--RTS-

----------------------------------------------------RMYAF---

-------------------------------------QFYNYS-----------------

-----------Y------LN--LS-S-------VS----E-A-QA-----P-------K-

M--ARVL-------NFSHN-------M---I--E-----------KIAKRDFEG--F-DA

L-----------EVLDLA-YNQ------------------I-KD--IEP--GVFENLLSL

VSVNFS------F--------ND---KK-----------------------LD---V-SG

------LASGL---KL---LPTIEVS-GGLQLYKY-FETSS-----------E---TA--

----LESSVS--------------------------------------------------

-------------AKELRLSEG-PADQLSV-----------------NPRFRRSK---RD

PRRAEKNVTVSPTAGLR-PN-FC--------G-------------------------TPI

NGILDLS-----KS---KLSEEELMEKLDADLCQVQL---------------DSILE---

-LNI-SRS---------NLE--MDLLSLFIL-FLPMKNV----RSID----A-----S--

Y--NK-----------ITINNIDVEAICH--------------F--PF--HNLFF----L

NIS----------NNPMNNLDTVC---LP------------STIKVIDL--SFT-NI-S-

-QIPKNFAEKLFNLEN--------------------------------MYVQGN------

------------H---------------------F---I-YTV-----SPENTNANQKPA

PG--------------TVRINA---ISLVRNEAGTPI-----------------------

------------------------------------------------------------

-------------------------------ESLPG------------------------

---------------------------K--VKH-------LKMSNCSIVEL-PEWF--AG

TMKE--LLLLDLSSNP--------V-------------------------STFP------

----------------HL----PISLQYLDISNSD-------------I--K-----VIP

-PSFKSL--SN-LTIFNIQN-N-K---------ITEMPLEYFPLTLTKCDVSKNKLQVLS

LT-----------------------ETL------------------K-------------

--KLEYLNVSGNL--I----T-----------------TLEPTRQ------LS-------

AITNL----------DSS-------HNLI----AE--L-----PDH-----FG--K-SLP

----------------------------------------------------------T-

---------------------LKYLNLSGNKISFL-QSGSLP---ASLIELDISNNAITT

IVED---------T-FG------------QLTS-------------LSVLTVQ-GKHFFC

NCDL--Y-WFVNIYIRNP--------------Y----L----------------------

-------------------QI--------NG-------RENLRC-SFP-PD----RR---

-GSLVESS--NLTLLR-C-SLGI-------QMAITACVAILIVLLLTGL----C--WHFD

G-VW-YLRMG---WY---W-----CM----A-KR--------------------------

-------------KQY------------------------------EKRPENK--P-YDA

FISYSEHDA---NWTKENLLEKL----ETK----------------------------G-

-F-----KICYHERDFK----------------P--GH-PVLGNIF-YCIENSHKVLFVL

SP------SFV-----------NSC-------WCQY-ELYFAEHRVLN--E-N-QDSLIM

IVLE--D-LPFNSV----------------------------------------------

--PQKFSKLRK-LLKRKTYLKWS----PE-------QHKQK-------VFWHQLTAVL--

--KTT------------NEPFVLRAENGSTEDMYEME-----------------------

------------------------------------------------------------

-----------------------------------------------------------

>TLR15_Stca

------------------------------------------------------------

------------------------------------------------------------

------------------------------------------------------------

------------------------------------------------------------

------------------------------------------------------------

------------------------------------------------------------

------------------------MRILIGKLY---------------------------

-------------------------------FYLISFSFSRTNGFL------TW--RTS-

----------------------------------------------------RAYAF---

-------------------------------------QFYNYS-----------------

-----------Y------LN--LS-S-------VS----EAH-G------P-------K-

T--ARVL-------NFSHN-------V---I--E-----------KIAKRDFEG--F-DA

L-----------EVLDLA-YNQ------------------I-KD--VEP--GAFENLLSL

VSVNFS------F--------ND---KK-----------------------LD-------

------V-TGLA--SH---LKLLPTGEGSGVLQLYKYFETS------SE-------TA--

----LESSVS--------------------------------------------------

-------------AKELQRSKGPSDRLNVN-----------------PKFRRSIQ----D

PRRAEKNVTVS---PT--AG-LK----PN---------------LCG----------TPI

NGILDLS-----KS---KLSEEDLTEKLDAQLCQAQL---------------DSILE---

-LNI-SHS---------SLE--MDLLSLFIL-FLPMKNV----QSID----A-----S--

Y--NK-----ITI---DNI-------------DVEA-------I------CHFTF----H

NFF-----FLNISNNPLNNLDTIC---LP------------STIKVIDL--SFT-NI-S-

-QVPQNFAKKLFNLEN--------------------------------MYVQGN------

------------H---------------------F---IYTVSPENTNANQKFA------

PG--------------TVHINA---ISLVRNEAGTPI-----------------------

------------------------------------------------------------

-------------------------------ESLPE------------------------

---------------------------K--VKH-------LKMSNCSIVEL-PEWF--AG

TMKE--LRLLDLSSNP--------V-------------------------STFP------

----------------HL----PISLQHLDISNSD-------------I--K-----VIP

-PSFKSL--SN-LTIFNIQN-N-K---------ITDMPLEYFPLALTKCDISKNKLQVLS

LT-----------------------ATL------------------K-------------

--KLDYLNVSGNL--I----T-----------------KLEPTSQ------LS-------

AITNL----------DSS-------HNLI----SE--L-----PDH-----FG--R-SLP

----------------------------------------------------------T-

---------------------LKYLNLSGNKISFL-QSGSLP---ASLIELDISNNAITT

IVED---------T-FG------------QLTS-------------LSVLTAQ-GKHFFC

NCDL--Y-WF----V--N-------------------I-------YI-------------

-------RNPSL-------QI--------NG-------RENLRC-SFP-PD----RR---

-GSLVENS--NLTLLH-C-SLGI-------QMAITACVAILIVLVLTGL----C--WHFD

G-LW-YVRMG---WY---W-----CM----S-----------------------------

------KRKQYEKRPE------------------------------NKL--------YDA

FISYSEHDA---NWTKENLLEKL----EAK----------------------------G-

-F-----KICYHERDFK----------------P--GH-PVLGNIF-YCIENSHKVLFVL

SP------SFV-----------NSC-------WCQY-ELYFAEHRVLN--E-N-QDSLIM

IVLE--D-LPFNSV----------------------------------------------

--PHKFSKLRK-LLKRKTYLKWS----PE-------QHRQK-------VFWHQLTAVL--

--KTT------------NEPLVLRAENGSTEDMYEME-----------------------

------------------------------------------------------------

-----------------------------------------------------------

>TLR15_Gaga

------------------------------------------------------------

------------------------------------------------------------

------------------------------------------------------------

------------------------------------------------------------

------------------------------------------------------------

-------------------MVPIGRKVSCCKKENPATHPHSPSCPAASTTKAAAWPGSFR

GCSRAARRVGTGNRRMRI------L--I-G--S---------------------------

--------------------------------L---Y--F--Y--F------IS----F-

---LFSKVNGFL----------------------------------------T--QR---

-TSP-VSS----------------------------FPFYNYS-----------------

-----------Y------LN--LS-S-------VS----Q-A-QA-----P-------K-

T--ARAL-------NFSYN-------A---I--E-----------KITKRDFEG--F-HV

L-----------EVLDLS-HNH------------------I-KD--IEP--GAFENLLSL

VSVDLS------F--------ND-K-NL-----------------------LV---S-GL

------A-PHL---KL---IPTSGAS-GP---SQI-YMYFQ------KS---A---EA--

----AL-EPS------------A-------------------------------------

-----------PAELLPHLE-DPP-NPGNVNPRFRQ------RRTEENKTSPPAA----T

LRPDLCGAPINGLLDLSRTK-LS----NE--E------------LTA---KLD----ADL

CQ-AQLG-----TV--------LEFNISH------------------------SDLE---

-MDL--LS----L----FIL---------------FLPM----KDIQ----SVD---A--

S--YN---R-I-T---INN-------ID----VEAI-------C--HFPFSNFSF----L

NIS----------NNPINSLETVC---LP-E----------S-ITVIDL--SFT-NI-S-

-TI-PA--NFAKKLSK---------------------LE---R---MYV--QGN-----Q

-----------------L-I---Y--T-------V--RP-ENP----------S-----A

TP----R------------PP----PGTVQISA-ISL-----------------------

-------------------------------------------------VRNQAGTPI--

-------------------------------ESLPE------------------------

---------------------------S--VKH-------LKVSNCSIVEL-PEWF--AN

RMQE--LLFLDLSSNR--------I-S-M------L--------------PDLP--ISLQ

QLDISNSDIKIIPPRFKS--L-SN-LTVFNIQNNK-------------L--T-EM-----

-HPEYFP--ST-LTTCDISK-N-K--------------------------L-K---V---

--------LS----------L-T--KAL------------------E-------------

--NLESLNVSGNL--I----T-RL--------------EPACQLP---------------

SLTNL----------DSS-------HNLI----SE--L-----PDH-----LG--Q-SLL

----------------------------------------------------------M-

---------------------LKHFNLSGNKISFL-QRGS--LP-ASLEELDISDNAITT

IVQD---------T-FG------------QLTS-------------LSVLTVQ-GKHFFC

NCDL--Y-WF----V--N--------------I----Y-------IR-----------NP

-------H-----------LQ--------IN-G-----KDDLRC-SFP-PD----RR---

-GSLVKSS--NLTLLH-C-SLGI-----QMAIT--ACMAILVVLVLTGL----C--WRFD

G-LW-YVRMG---WY---W-----CM----A--K------------------R-------

-------------RQY---------------K----KR----P---ENK---P----FDA

FISYSEHDA---DWTKEHLLKKL----ETD---G--------------------------

---F---KICYHERDFK----------------P--GH-PVLGNIF-YCIENSHKVLFVL

SP------SFV-----------NSC-------WCQY-ELYFAEHRVLD--E-N-QDSLIM

VVLE--D-LPPDSV----------------------------------------------

--PQKFSKLRK-LLKRKTYLKWS----PE-------EHKQK-------IFWHQLAAVL--

--KTT------------NE-----------------------------------------

-------------------P-LVRAE-------NG-----PN--EDVIEME---------

-----------------------------------------------------------

>TLR15_Cost

------------------------------------------------------------

------------------------------------------------------------

------------------------------------------------------------

------------------------------------------------------------

------------------------------------------------------------

------------------------------------------------------------

---------------------------MRI--L---------------------------

-------------------------------IGSLHFYFMPFL---------LS------

-------------------------------------------------------RA--N

-GSL-T-----------------------QTPAAYASPVYNYS-----------------

-----------Y------LN--LS-S-------VS----E-A-RA-----P-------K-

T--ARAL-------NFSHN-------V---I--G-----------SITKRDLEG--F-VL

L-----------QVLDLS-YNQ------------------I-KD--IEP--GAFESLPCL

ASVDLS------F--------NS---KE-----------------------LV---V-SD

------LPAPLKLSPTSEASGALQLY-KH---CGNSSEQPHSGGPSVLP-------NV--

-------NPR--------------------------------------------------

-------------PRHLPQR-A-E-RNSTV-----------------SPPATPRPHFCGE

PVNGTLDLSHS---KLTEEE-LM---------------------------QKL---DPDL

CQ-AQLD----------------------------------------------SILE---

-LDI--SH----T----DLQ--MDLLSLFVL-FLPMKSL----RSID----A-----S--

C--NR-----------LTINILDVEAICS--------------F--PL--SKLLF----L

NIS----------HNPINSLDTLC---LP------------STIRVIDL--SFT-NV-S-

-QI-PS--NFAKKLWN---------------------LE---T-----M--YTQ-----G

------------N----H----------------F---IYTARAEGTGAAPKPG---PGF

VH-----------------IDA---ISFVRNQAGTPI-----------------------

------------------------------------------------------------

-------------------------------QSLPK------------------------

---------------------------K--VKR-------LKMSNCSIVEL-PEWF--AG

TMAE--LLFLDLSTNR--------I-------------------------SVLP------

----------------DL----PSSLQHLDISNSD-------------I--K-----TIP

-PTFKSL--SN-LTTFNIQN-N-E---------VTALQPEYFPGALTRCDL-N-------

-------RNK------------L--SVL------------PLTRAVG-------------

--KLRYLSVSGNL--I----T-----------------RLEPSGP------LP-------

ALVHL----------DSS-------HNLI----SE--L-----PDH-----FG--K-SLP

----------------------------------------------------------G-

---------------------LRYLNLSGNKISFL-QRGSLP---ASLVELDISNNAITT

IVED---------T-FG------------PLTS-------------LSVLTVE-GKHFFC

NCDL--Y-WF----V--N--------------V----Y---MHS-PH-------------

-------L-----------QI--------NG-------KGKLRC-SFP-PA----RR---

-GSLVESS--NLTLLH-C-SLGI-------QMAITASVAILVVLVLTGL----C--WRFD

G-PW-YVRMG---WY---W-----CM----A-KR--------------------------

-------------KQY--------------------------E---KRPE-DK--P-FDA

FISYSEQDA---NWTKEILLRKL----ETD----------------------------G-

-F-----KICYHERDFK----------------P--GH-PVLGNIF-YCIENSHKVLFVL

SP------SFV-----------NSC-------WCQY-ELYFAEHRVLN--E-N-QDSLIM

IVLE--D-LPLNSV----------------------------------------------

--PQKFSKLRK-LMKRKTYLKWS----PE-------EHKQK-------LFWHQLAAVL--

--KTT------------NE-----------------------------------------

------------------------AL-------VR-----AENGCAQDMYEME-------

-----------------------------------------------------------

>TLR15_Burh

------------------------------------------------------------

------------------------------------------------------------

------------------------------------------------------------

------------------------------------------------------------

------------------------------------------------------------

------------------------------------------------------------

-----------MRILTGS---------LCF--F---------------------------

------------------------------------------FISF------LF--NST-

-------------------------------------------------------NG--L

-PTL-G----------------------TPTAYAFPFFNYSCL-----------------

-----------N----------LS-S-------VA----E-A-RA-----P-------K-

T--ARAL-------NLSHN-------A---I--E-----------KITKRDLEG--F-DA

L-----------QVLDLS-YNQ------------------I-KD--IEP--GAFESLLSL

VSVNLS------F--------NH---EN-----------------------LL---V-SG

------LPPHLELLPSSKASGSLQLS-KY-------FDKSSEAAASSEE---L---PH--

----WGGPLA--------------------------------------------------

-------------LQDVRPRLRRS-TEGLLR----------------EGEKNGTPSPTAT

VRPDLCGVPINGILNLSHSK-LS----EE--E-------------------------LML

KLDSDLC-----QA------------------------------------QLEGILE---

-LDI--SH----S----DLE--MDLLSLFVL-FLPMKNM----RSVD----A-----S--

H--NK-----------LTINVLDVGSICN--------------F--PF--SQLLF----V

NIS----------HNPINSLDTLC---LP------------STIKVIDL--SFT-NI-S-

-RL-PR--IFATRLPH--------------------------------V--EQV-----Y

-----------------VQG---N--H-------F---I-YTA------HQGGN---NSL

QN----C---P--------LGV---VCISSISF---------------------------

-------------------------------------------------VRNDAGTPI--

-------------------------------ERLPS------------------------

T-----------------------------VKQ-------LKMSNCSIVEL-PEWF--AN

TMNQ--LLVLDLSSNQ--------I-------------------------SLLP------

----------------DL--P-PS-LQHLDISNSD-------------I--E-----IIP

-PRFKSV--SS-LTVFNIQN-N-K--------VTDMHPEYFPLNLTKCDIS-K--NKLKL

LS---LTDTL------------------------------------E-------------

--NLEYLNVSGNL--I----T-----------------RLEPASH------LS-------

ALTNL----------DSS-------HNLV----SE--L-----PDH-----FG--K-SLP

----------------------------------------------------------M-

---------------------LKYFNLSGNKISFL-QRGSLP---ASLVELDISDNAITT

IVED---------T-FG------------QLTS-------------LSVLTVQ-GKHFFC

NCDL--Y-WF----V--N--------------V----Y-------IH-------------

-------K---------------------PQLWING--KENLRC-SFP-PQ----RR---

-GSLVARS--NLTLLH-C-SLGI-------QMAITACVATLVVLVLTGL----C--WRFD

G-LW-YVRMG---WY---W-----CM----A-----------------------------

-------------KRK--------------------QYEKRPE---NKL--------FDA

FISYSEHDA---NWTKENLLEKL----ERD----------------------------G-

-F-----KICYHERDFK----------------P--GH-PILGNIF-YCIENSHKVLFVL

SP------SFV-----------NSC-------WCQY-ELYFAEHRVLN--E-N-QDSLIM

IVLE--D-LPPHSV----------------------------------------------

--PQKFSKLRK-LLKRKTYLKWS----PE-------EHKQK-------MFWRQLAAVL--

--KTT------------NE-----------------------------------------

------------------------PL-------LR-----AEDGAAQEAYEME-------

-----------------------------------------------------------

>TLR15_Menu

------------------------------------------------------------

------------------------------------------------------------

------------------------------------------------------------

------------------------------------------------------------

------------------------------------------------------------

------------------------------------------------------------

---------------------------MRI--L---------------------------

---------------------------------------FGSLCFC------FI--SFL-

----------------------------------------------------LSRAN--G

-FLT-L-----------------------RTPTAYTFPVYNYS-----------------

-----------Y------LN--LS-S-------VA----E-A-QA-----P-------K-

T--ARAL-------NFSHN-------V---I--E-----------KITKRDLEG--F-DV

L-----------EVLDLS-YNQ------------------I-KE--VEP--GVFESLPSL

VSVNLS------F--------ND---EK-----------------------LL---V-ST

------LAPPLKLLPAGKASGSLQLS-ED-------SGTPS-----------G---AA--

----LG-HAA------------S-------------------------------------

------AEGGPSVLQDLSPRQG-R-STENLLR---------------RAEPQGTASPPAT

PKRDLCGAPADGTLDLSHSQ-LS----EE--D-------------------------LNL

KLDPKLC-----QA------------------------------------QLHGILH---

-LNI--SH----S----GLE--MDLLSLFIL-FLPMENV----QSVD----A-----S--

Y--NK-----------ITINMENVDPICK--------------F--PF--KNLLF----L

NIS----------NNPINSLDTLC---LP------------STIKVIDL--SFT-NI-S-

-QI-PQ--NFATKLFN---------------------LE---N-----M--YVQ-----G

------------N----H----------------F---I-YTV-----RPPEVN---EAP

RPPPGTV---R--------INA---ISFVRNQAGTPI-----------------------

------------------------------------------------------------

-------------------------------ESLPK------------------------

---------------------------K--VRH-------LKMSNCSIVEL-PEWF--AG

TMQE--LLFLDLGSNR--------I-------------------------SVLP------

----------------DL--P-PS-LQHLDISNSD-------------I--K-----IIP

-PRFKSV--SN-LTIFSIQN-N-K--------ITDMHPEHFPLTLTKCDIS-K--NRLNV

LL---LTDAL------------------------------------E-------------

--KLEYLNVSRNL--I----T-----------------RLEASSH------LS-------

ALANL----------DSS-------HNLI----SE--L-----PDH-----FG--K-ALP

----------------------------------------------------------V-

---------------------LKHFNLSGNKISFL-QRGCLP---ASLVELDISDNAITT

IVED---------T-FG------------QLTS-------------LSVLTAQ-GKHFFC

NCDL--Y-WF----V--N--------------V----Y---MRS-PR-------------

-------L-----------RI--------RG-------KGALRC-SFP-PE----RR---

-GSLVESS--NLTLLR-C-SLGA-------QMAVTACVAVLVATGVTGL----C--WRLD

G-LW-YVRMG---WY---W-----CM----A-KR--------------------------

-------------KQY--------------------------E---KRPE-NK--P-FDA

FVSYSEHDA---EWTKENLLQRL----EMD----------------------------G-

-F-----KICYHERDFK----------------P--GH-PVLGNIF-YCIENSHKVLFVL

SP------SFV-----------NSC-------WCQY-ELYFAEHRVLN--E-N-QDSLIM

IVLE--D-LPPNSV----------------------------------------------

--PQKFSKLRK-LLKRKTYLKWS----PE-------EHKQK-------MFWRQLEAVL--

--KTT------------NE-----------------------------------------

------------------------PL-------VR-----AENGSAQDIYEMQ-------

-----------------------------------------------------------

>TLR15_Euhe

------------------------------------------------------------

------------------------------------------------------------

------------------------------------------------------------

------------------------------------------------------------

------------------------------------------------------------

------------------------------------------------------------

---------------MRI------L--I-G--S---------------------------

--------------------------------L---C--F--Y--F------IS----S-

---LLGRANGFL----------------------------------------P--LR---

-AAT-ALA----------------------------FPFYDYS-----------------

-----------Y------LN--LS-S-------IS----E-A-RA-----P-------K-

T--ARAL-------NFSHN-------V---I--E-----------KITRRDMEG--Y-EA

L-----------EVLDLS-YNR------------------I-KD--VER--GAFESLLGL

VSVNLS------F--------ND-E-KL-----------------------LV---P-AL

------P-PAL---KL---SPTGKAS-GS---WRL-YKDSD------KS---S---EA--

----AL-EPS------------A-------------------------------------

-----------SAGELPRSG-GPS-VPQKVSPRLRRSIGNLLRRGEKTVTVSPAA----T

SQPDLCEAPINGILDLSNSK-IT----ED--E------------LML---KLD----PDL

CQ-AQLD-----GI--------VEFNISH------------------------SDLE---

-MEL--LS----L----FVL---------------FLPM----KNLR----SVD---G--

S--HN---K-L-K---INV-------LD----VGAI-------C--KFPSSKLLF----V

NIS----------NNPINSLDTLC---LP-S----------T-IKVIDL--SFT-GI-S-

-QI-PQ--NFATKMPD---------------------LE---N---MYV--QGN-----H

-----------------F-I---Y--T-------A--HP-EAS----------N-----A

AP----R------------LP----PGTVHINA-ISF-----------------------

-------------------------------------------------VRNQAGTPI--

-------------------------------ESLPR------------------------

---------------------------K--VKR-------LKMANCSIVEL-PEWF--AD

TMEE--LLFLDLSSNR--------I-S-V------L--------------PDLP--PSLQ

HLDLSNSDIKTIPPGFRS--V-SN-LTIFNIQNNK-------------I--V-DM-----

-HPEYLP--LT-LTKCDISK-N-R--------------------------L-K---A---

--------LP----------L-T--GAP------------------E-------------

--KLEYLNVSGNL--I----T-SL--------------EPASHLP---------------

ALANL----------DGS-------HNLI-----------------------------LP

----------------------------------------------------------M-

---------------------LKYFNLSGNKISFL-QRGS--LP-ASLIELDISDNAITT

IVED---------T-FS------------PLTS-------------LSVLTVQ-GRHFFC

NCDL--Y-WF----V--N--------------V----Y-------SR-----------QP

-------H-----------VR--------IN-G-----KGNLQC-SFP-PD----RR---

-GSSVEST--NLTLLR-C-SLGI-----QMAIT--ACVAILVVLVLTGL----C--WRFD

G-PW-YVRMG---WY---W-----CM----A--K------------------R-------

-------------KQY---------------K----ER----P---ENK---P----FDA

FISFSERDA---KWTEEHLLEKL----ERD---G--------------------------

---F---RICYHERDFK----------------P--GH-PILGNIF-YCIENSHKVLFVL

SP------SFV-----------NSC-------WCQY-ELYFAEHRVLN--E-N-EDSLIM

IVLE--D-LPPNSV----------------------------------------------

--PQKFSKLRK-LLKRKTYLKWS----PE-------EHKQK-------MFWHQLTAVL--

--KTT------------NE-----------------------------------------

-------------------P-LVRTE-------NG-----SA--QATLEME---------

-----------------------------------------------------------

>TLR15_Ptgu

------------------------------------------------------------

------------------------------------------------------------

------------------------------------------------------------

------------------------------------------------------------

------------------------------------------------------------

------------------------------------------------------------

---------------MKILTGSLHFHFISF--L---------------------------

---------------------------------------FSRANGF------LT----L-

-------------------------------------------------------RT--P

-TTD-A------------------------------SPFYNYS-----------------

-----------Y------LN--LS-S-------IS----E-A-QA-----P-------K-

M--ARAL-------NFSHN-------I---M--E-----------KITKQDLEG--F-DA

L-----------EVLDLS-YNR------------------I-KD--IEP--GAFETLPSL

VSVNLS------F--------ND---NE-----------------------LL---V-LG

------LPAHLKPAPTSEASGSFQLF-KY-------FDNSS-----------EAAPEP--

----YT----------------S-------------------------------------

-------------PEGLPHSGGPS-VLQNV-----------------NLELKRNTENPLP

RAEEKVTVSPT---ATLQPN-FC--------G-------------------------API

NGILDLS-----NS---KLTQEELEAKLDADLCQPQM---------------NNILE---

-LDI--SH----SELEMDLLSLSMLFWP-------MKNV----RSVN----A-----S--

Y--NK-----------ITINILDAEAICT--------------F--PF--SKLLF----L

NIS----------NNPINSLNTLC---LP------------STIKVLDL--SFT-NV-S-

-QI-PK--NFAKKMIN--------------------------------L--ETM-----Y

-----------------VEG---N--H-------F---I-YTI------HPEIT------

SA----A---PQPPPGTVHINA---ISFVRNQAGTPI-----------------------

------------------------------------------------------------

-------------------------------ESLPK------------------------

---------------------------K--LKH-------LQMSNCSIVEL-PEWF--AG

TMQE--LLFLDLSSNR--------I-------------------------SMLP------

----------------DL----PASLQHLDISNSD-------------I--K-----TIP

-PTFKSL--SN-LTMFNIQN-N-K--------ITDMHPEHFPLSLTKCDIS-R--NKLNV

LS---LTDTL------------------------------------V-------------

--KLEYLNVSGNL--I----T-----------------RVEPTGH------LS-------

ALANL----------DSS-------HNLI----SE--L-----PD------LF--GKSLP

----------------------------------------------------------M-

---------------------LKYFNLSGNKISFL-QHGSLP---TSLIELDISDNAITT

IVED---------T-FG------------PLTS-------------LSVLTVQ-GKHFFC

NCDL--Y-WF----V--N--------------V----Y---IHK-PH-------------

-------L-----------QI--------NG-------KENLRC-SFP-PD----RR---

-GSLVESS--NLTLLR-C-SLGI-------QMAITACVAVLVVLLLTGL----C--WWFD

G-LW-YVRMG---WF---W-----CM----A-KR--------------------------

-----------KQYKE--------------------------R---PEN---K--P-FDA

FISYSEQDA---KWTKENLLEKL----EAD----------------------------G-

-F-----KICYHERDFK----------------P--GH-PILGNIF-YCIENSHKVIFVL

SP------SFV-----------NSC-------WCQY-ELYFAEHRVLN--E-N-QDSLIM

IVLE--D-LPPNSV----------------------------------------------

--PQKFSKLRK-LLKRKTYLKWS----PE-------EHRQK-------MFWHQLAAVL--

--KTT------------NE-----------------------------------------

------------------------PL-------VR-----AENGSARDMYEMDEIQEHAE

VVPGNSTVK--------------------------------------------------

>TLR15_Apvi

------------------------------------------------------------

------------------------------------------------------------

------------------------------------------------------------

------------------------------------------------------------

------------------------------------------------------------

------------------------------------------------------------

---------------MRILTESLHFYLISF--L---------------------------

---------------------------------------FSGANGF------LT----P-

-------------------------------------------------------RT--P

-TAY-A------------------------------FPFCNYS-----------------

-----------Y------LN--LS-S-------VS----E-A-QA-----P-------K-

T--ARAL-------NFSHN-------L---I--E-----------KITKRDLEG--F-DA

L-----------EVLDLS-YNL------------------I-KD--VES--GALESLFSL

VSVNLS------F--------NDKK-LH-----------------------VS---DLPP

------YLKLL---PTSKASGSLQLY-KY-------FEKSS-----------E---AA--

----LE-PLA------------S-------------------------------------

-------------AKKLPHSRGRP-LLQNV-----------------NSRLRRSTENLLR

KAEKNATVAPR---ATLRPN-FC--------G-------------------------API

NGKLDLS-----YS---KLSEEELEIKLNPNFCQAEL---------------DSILE---

-LDI--SH----S----DLE--MDLLSLFIL-FLPMKNV----QWID----A-----S--

H--NK-----------LKINDQDVEAICK--------------F--PF--SMFFF----L

NIS----------NNPINSLDNLC---LP------------STIRVIDL--SFT-NI-S-

-QI-PQ--NFARKLFN---------------------LE---N-----M--YVQ-----G

------------N----H----------------F---IYTAR-----PEASQN---LSS

GA----V---T--------INA---ISLVRNEAGTPI-----------------------

------------------------------------------------------------

-------------------------------ESLPR------------------------

---------------------------K--VKH-------LKMSNCSIVEL-PEWF--AD

TMEE--LLFLDLSSNP--------I-------------------------SVLP------

----------------HL----PTTLQHLDISNSD-------------I--K-----TIP

-PSFKSI--SN-LTIFNIQN-N-K--------ITDMHPEYFPLTLTKCDIS-K--NKLNM

LS---LTDVL------------------------------------E-------------

--KLEYLNISGNM--I----T-----------------RLEPTSN------LS-------

ALTNL----------DGS-------HNQI----SE--L-----PD------YF--GKSLS

----------------------------------------------------------T-

---------------------LKYFNLSGNKISFL-QRGSLP---ASLIELDISDNAIST

IVED---------T-FG------------QLTS-------------LRVLTLQ-GKHFFC

NCDL--Y-WF----V--N--------------V----Y---TRN-PH-------------

-------L-----------QI--------NG-------KGNLKC-SFP-PH----RR---

-GSLVESS--NLTLLH-C-SLGI-------QMAITACIAILVALALAGL----C--WRFD

G-LW-YVRMG---WY---W-----CM----A-KR--------------------------

-------------KQY--------------------------E---KRPE-NK--P-FDA

FISYSEHDA---NWTKENLLQKL----EME----------------------------G-

-F-----KICYHERDFK----------------P--GH-PVLGNIF-YCIENSHKVLFVL

SP------SFV-----------NSC-------WCQY-ELYFAEHRVLN--E-N-QDSLIM

IVLE--D-LPPDSV----------------------------------------------

--PQKFSKLRK-LLKRKTYLKWS----PE-------EHKQK-------MFWRQLAAVL--

--KTT------------NE-----------------------------------------

------------------------PL-------VK-----AENGAAQDMYEMK-------

-----------------------------------------------------------

>TLR15_Pipu

------------------------------------------------------------

------------------------------------------------------------

------------------------------------------------------------

------------------------------------------------------------

------------------------------------------------------------

------------------------------------------------------------

---------------------------MRI--L---------------------------

---------------------------------------VGSLHLC------FI--SFL-

--------------------------------------------FSRPNGFLR--TP--T

-ACA-L-------------------------------LAYNYS-----------------

-----------Y------LN--LS-S-------VP----E-A-QA-----P-------K-

M--ARAL-------NFSHN-------L---I--E-----------KITKRDLEG--F-EA

L-----------EVLDLS-YNQ------------------I-KE--IEP--GAFESLLSL

ISVNLS------F--------ND---KK-----------------------LL---V-SG

------IPSSLKLLATSKALGSLQLY-KY-------FDKSS-----------G---AA--

----LE-PAK--------------------------------------------------

--------------ELPHSG-GPS-GLHNV-----------------HPRLRRST----E

NLLRRGEQTEA---DSPAGT-LQ----PN---------------LCA----------API

NGMLNLS-----HR---KLSEEELMLKLDSDLCQAQL---------------DSILE---

-LDI--SH----C----DLE--MDLLSLFTL-VLPMTNV----QYID----A-----S--

C--NK-----------LTINILDVEAICK--------------F--PL--SKLLF----L

NIS----------NNPINSLDTLC---LP------------STIKVIDL--SFT-NI-S-

-QI-PP--NFATKLFN---------------------LE---H-----M--YVQ-----G

------------N----H----------------F---IYTARPEVTEEVPKFS---PGT

VH-----------------INA---ISFVRNQAGTPI-----------------------

------------------------------------------------------------

-------------------------------ESLPK------------------------

---------------------------K--LKH-------LKVSNCSIVEL-PEWF--AD

TMGE--LLLLDLSSNR--------I-------------------------SVLP------

----------------DL--P-PS-LQHLDVSNSD-------------I--K-----MIP

-PGFKSA--SN-LTIFNIQN-N-K-----------------ITDMHPE--------YL--

----------------------------------------P-----L-------------

--TLTKCDISKNK--L----N-----------------LLLLTDA------LE-------

KLEHL----------NVS-------GNLI----TR--L----EPTS-----HLSVLTNLD

----------------------------------------------------------S-

---------------------SHKLISEGNKISFL-QRGSLP---TSLVELDISDNAITT

VVED---------T-FG------------QLSS-------------LSVLTVQ-GKHFFC

NCDL--Y-WF----V--N--------------V----Y---IRK-PQ-------------

-------L-----------QI--------NG-------KGNLRC-SFP-PE----RR---

-GSLVESS--NLTLLH-C-SLGI-------QMAITACAAILVVLVLTGL----C--WRFD

G-LW-YMKMG---WY---W-----CM----A-KR--------------------------

-------------KQY--------------------------E---KRPE-NK--P-FDA

FVSYSEHDA---NWTKQNLLEKL----EMD----------------------------G-

-F-----KICYHERDFK----------------P--GH-PVLGNIF-YCIENSHKVLFVL

SP------SFV-----------NSC-------WCQY-ELYFAEHRVLN--E-N-QDSLIM

IVLE--D-LPPNSV----------------------------------------------

--PQKFSKLRK-LLKRKTYLKWS----PE-------EHKQK-------MFWRQLAAVL--

--KTT------------NE-----------------------------------------

------------------------PL-------MR-----TENGSTQEAYEME-------

-----------------------------------------------------------

>TLR15_Anpl

------------------------------------------------------------

------------------------------------------------------------

------------------------------------------------------------

------------------------------------------------------------

------------------------------------------------------------

------------------------------------------------------------

-----------MGILIRS------LHFYFT--S---------------------------

-------------------------------FLLSRANGF-----L------TW------

-------------------------------------------------------RT--T

-ESA-F-------------------------------PFYNYS-----------------

-----------Y------LN--LS-S-------VS----E-A-QA-----P-------K-

M--ARAL-------NFSHN-------I---I--E-----------KITKRDFEG--F-DA

L-----------EVLDLS-YNQ------------------I-KD--IEP--GTFEILLSL

VSVNLS------F--------ND---KK-----------------------LL---V---

--------SGL--------APHLKLL-AT-------SRASDKSTEAALE---P---PASA

EELPHSGSPP------------D-------------------------------------

-------------LQKINLR-LRR-STRHLR----------------RAEENTMVSPTAT

LRPNRCGLPINGTLDLSKSK-LS--------E-------------------------EEL

TEKLDPDLCQAQLD---------------------------------------GVLE---

-LDI--SH----N----DLE--MDLLSLFIL-FLPMKNV----QSID----A-----S--

Y--NN-----------ITINNIDVEAICR--------------F--PF--SNLSF----V

NIS----------NNPLNNLETIC---LPPT------------ITVIDL--SFT-NI-S-

-VI-PQ--NFAKKLFN---------------------LE---N-----M--YVQ-----G

------------N----H----------------F---IYTVHPKPNNTAPKFA---PGT

VH-----------------ISA---ISLVRNQAGTPI-----------------------

------------------------------------------------------------

-------------------------------ESLPE------------------------

---------------------------K--VKH-------LKISNCSIVEL-PEWF--AN

RMQE--LLFLDLSSNR--------I-------------------------SMLP------

----------------DL----PISLQHLDISNSD-------------I--K-----IIP

-PSFKSL--PN-LTVFNIQN-N-K--------VTDMHPEYFPLTLTKCDIS-K--NKLSV

LS---LTKTI------------------------------------G-------------

--KLEFLNVSRNL--I----T-----------------RLEPTSQ------LR-------

LLTNL----------DGS-------HNLI----SE--L-----PDH-----FG--K-SLP

----------------------------------------------------------M-

---------------------LKYFNLSGNKISFL-QRGSLP---VSLMELDISDNAITT

IAED---------T-FG------------QLTS-------------LSILTVQ-GKHFFC

NCDL--Y-WF----V--N--------------V----Y---VHT-PH-------------

-------L-----------QI--------NG-------KENLRC-SFP-PD----RR---

-GSLVEGS--NLTLLH-C-SLGI-------QMAITACVAVLVVLVLTGL----C--WRFD

G-LW-YVKMG---WY---W-----CM----A-KR--------------------------

-----------KQYKK--------------------------R---PEN---K--P-FDA

FISYSEHDA---NWMKENLLVRL----ETD----------------------------G-

-F-----KICYHERDFK----------------P--GH-PVLGNIF-NCIENSHKVLFVL

SP------SFV-----------NSC-------WCQY-ELYFAEHQVLN--E-N-QDSLIM

IVLE--D-LPPNSI----------------------------------------------

--PQKFSKLRK-LLKRKTYLKWS----PE-------EHKQK-------IFWHQLAAVL--

--KTT------------NE-----------------------------------------

------------------------PF-------IV-----RAENGPTQDMYEMK------

-----------------------------------------------------------

>TLR15_Capu

------------------------------------------------------------

------------------------------------------------------------

------------------------------------------------------------

------------------------------------------------------------

------------------------------------------------------------

------------------------------------------------------------

-----------MRILIGS------LHCYFV--S---------------------------

-------------------------------FLLSRANTF-----L------IL------

-------------------------------------------------------RT--P

-TAH-A------------------------------SLVYNYS-----------------

-----------Y------LN--LS-S-------VL----E-A-QA-----P-------K-

I--ARAL-------NFSHN-------A---I--E-----------KITKRDLEG--F-EA

L-----------EVLDLS-YNQ------------------I-KD--VEP--GVFESLLSL

VSVNLS------F--------ND---KK-----------------------LL---L-SG

------LPPHLKLLPASKASGSLELS-KY-------FDKSS-----------E---AA--

----LE-PSA------------S-------------------------------------

-------------AKELSRS-GGP-SV--------------------LPDVKLRR----R

RNTENLLRRAEKNVTV--SP-TA----TSGPN------------LCA----------API

NGTLNLS-----NS---KLSAEELTLKLDQDLCQAQL---------------DGILE---

-LDI--SH----S----DLE--MDLLSLFSL-VLPMKNV----QSVD----A-----S--

Y--NK-----LTI---NIL-------------NVEG-------L------FKFPF----S

KLW---FLNISNNPINSLDTLWLP-----------------STIKVIDL--SFT-NI-S-

-QI-PQ--NFAKKFIN---------------------LE---N-----M--YVQ-----G

------------N----H----------------F---I-YTV------HPEIT---NAA

PK----F---C--------PGT---VHINAISF---------------------------

-------------------------------------------------VRNQAGTPI--

-------------------------------ESLPK------------------------

---------------------------K--VKH-------LKMSNCSIVEL-PEWF--VD

TMEE--LLFLDLSSNR--------I-------------------------SVLP------

----------------DL----PIALQHLDISNND-------------I--K-----IIP

-SSFKSA--SN-LTTLNIQN-N-K--------ITDMHPEYFPLTLTRCDIS-K--NKLNF

LS---LTDAL------------------------------------E-------------

--KLDYLNVSGNL--I----T-----------------RLEAGSH------LS-------

ALTNL----------DSS-------HNLI----AE--L-----PDH-----FG--K-SLA

----------------------------------------------------------T-

---------------------LKYFNLSGNKISFL-QHGALP---PSLIELDISDNAITT

IVED---------T-FG------------RLTS-------------LSVLTVR-GKHFFC

NCDL--Y-WF----V--N--------------V----Y---IHH-PR-------------

-------L-----------QI--------NG-------RGSLRC-SFP-PD----RR---

-GSLVESS--NLTLLH-C-SLGI-------QMAITAGVAILVVLVLTGL----C--WWFD

G-LW-YLRMG---WY---W-----CV----A-KR--------------------------

-------------KQY--------------------------E---KKPE-NK--P-FDA

FVSYSEEDA---NWTKEHLLEKL----EAD----------------------------G-

-F-----KICYHERDFK----------------P--GH-PVLGNIF-YCIENSHKVLFVL

SP------NFV-----------NSC-------WCQY-ELYFAEHRVLN--E-N-QDSLIM

IVLE--D-LPPNSV----------------------------------------------

--PQKFSKLRK-LLKRKTYLKWS----PE-------ERKQK-------MFWHQLAAAL--

--KTT------------NE-----------------------------------------

------------------------PL-------VR-----AQKGSAQDVCEME-------

-----------------------------------------------------------

>TLR15_Fach

------------------------------------------------------------

------------------------------------------------------------

------------------------------------------------------------

------------------------------------------------------------

------------------------------------------------------------

------------------------------------------------------------

---------------MRI------V--I-G--S---------------------------

--------------------------------L---H--F--Y--F------TS----F-

---LLSGANGFL----------------------------------------T--LR---

-TPT-AYA----------------------------FPVYNYS-----------------

-----------Y------LN--LS-S-------VS----E-A-EA-----P-------K-

A--AKAL-------NFSHN-------V---I--E-----------KITKRDLEG--F-DT

L-----------EVLDLS-YNQ------------------I-KD--IEP--GAFEGLLSL

VSVDLS------F--------ND-Q-RL------------------------V---S-RL

------P-PHL---KL---LPTSKAS-GS---LQL-YTNSD------KP---S---EA--

----AL-QPP------------A-------------------------------------

-----------SAGGLPAPG-GPP-VLPNGNLRLRRSAGNLLRRAEKNATVSPTA----T

VRPGFCGEPINGTLDLSHSK-LS----EE--E------------LML---MLD----PDL

CQ-AQLD-----RI--------LELDISH------------------------SELQ---

-MDL--LS----L----FML---------------FLPM----KNVR----SVD---V--

S--YN---K-L-T---INI-------LD----VEAI-------C--SFPFSKLLF----L

NFS----------NNPINSLDTLC---LP-P----------T-IKIIDL--SFT-NI-S-

-QI-PQ--NFAKKMPH---------------------LE---N---MYV--QGN-----H

-----------------F-I---Y--T-------V--HP-AIT----------N-----A

VP----Q------------PC----PGTVHINA-ISF-----------------------

-------------------------------------------------VRNQAGTPI--

-------------------------------ESLPK------------------------

---------------------------K--AKH-------LKMSNCSIVEL-PEWF--AS

TMEE--LLFLDLSSNR--------I-S-V------L--------------PDLP--TSLQ

HLDVSNSDIKIIPPSFKS--V-SN-LTILNIQNNK-------------I--T-DM-----

-HPEYFP--PT-LTKCDISK-N-K--------------------------L-N---T---

--------LS----------L-S--DAL------------------E-------------

--KLKYLNVSGNL--I----A-RL--------------EPASHLS---------------

ALTNL----------DSS-------HNLI----SE--L-----PDH-----FG--K-SLS

----------------------------------------------------------T-

---------------------LKYFNLSGNKISFL-QRGS--LP-ASLIELDISDNAITT

IVEG---------T-FG------------QLTS-------------LSVLTVQ-GKHFFC

NCDL--Y-WF----V--N--------------V----Y-------LH-----------KP

-------H-----------LQ--------IN-G-----RGHLRC-SFP-PD----RR---

-GSSVESS--NLTLLR-C-SLGI-----QMAVT--ACVATLVVLVVTGL----C--WRFD

G-LW-YLRMG---WY---W-----CM----A--K------------------R-------

-------------KQY---------------E----KR----P---ENK---P----FDA

FISYSEHDA---NWTKENLLGKL----EAD---G--------------------------

---F---KICYHERDFK----------------P--GH-PVLGNIF-YCIENSHKVLFVL

SP------SFV-----------NSC-------WCQY-ELYFAEHRVLN--E-N-QDSLIM

VVLD--D-LPPNSV----------------------------------------------

--PQKFSKLRK-LLKRKTYLKWS----PE-------EHKQK-------MFWHQLTAVL--

--KTT------------ND-----------------------------------------

-------------------Q-LVRAE-------NG-----ST--QDMYEME---------

-----------------------------------------------------------

>TLR15_Cuca

------------------------------------------------------------

------------------------------------------------------------

------------------------------------------------------------

------------------------------------------------------------

------------------------------------------------------------

------------------------------------------------------------

---------------MRILIGSLHFYFISF--L---------------------------

---------------------------------------FSRANGF------LT----L-

-------------------------------------------------------RT--P

-AAY-T------------------------------FPAYNYS-----------------

-----------Y------LN--LS-S-------VS----E-A-QA-----P-------K-

A--ARAL-------NFSHN-------V---I--E-----------KITKRDMEG--F-DV

L-----------EVLDLS-YNQ------------------I-KD--IEP--GVFESLLSL

VSVNLS------F--------ND---QK-----------------------LL---V-SG

------LPPHLKLLPTSQASRTLLLY-KH-------FDTSS-----------E---AA--

----LEPSAS------------A-------------------------------------

-------------KELLHSG-SPS-VLQNV-----------------NPRFRQGTEDLLR

RGEKNPTVSPT-------AN-LT----PD---------------FCG----------IPI

NGILDLS-----NR---KLSEEELTLKLDEDLCQAQM---------------DSILE---

-LDI--SH----S----DLE--MDLLSLFIL-FLPLKNV----QSID----A-----S--

H--NK-----------LTINTEDVEAICK--------------F--PF--SKLLF----L

NIS----------NNPINSLNTLC---LPPT------------IKVIDL--SFT-NL-S-

-QI-PW--NFAKKMIN--------------------------------L--EKM-----Y

-----------------VQG---N--H-------F---I-YTV----------------R

PQ----I---T--------NET---QNLPPGTVRINA-----------------------

-------------------------------------------------ISFVRNEAG--

-------------------------------TPIES------------------------

-------------------------LPK-KVKH-------LKMSNCSIVEL-PEWF--AG

TMEE--LLSLDLSSNR--------I-------------------------SVLP------

----------------EL----PSSLQLLDISNSD-------------I--K-----IIP

-PGFKSV--SN-LTTLNIQN-N-K--------ITDMHPEYFPLTLTKCDIS-K--NKLNM

LS---LTDAL------------------------------------E-------------

--KFEFLNVSGNL--I----T-----------------RLEPASR------LS-------

VLTNL----------DSS-------HNLI----SE--L-----PDH-----FG--K-SLP

----------------------------------------------------------M-

---------------------LKYFNLSGNKISFL-QRGSLP---ASLIELDISDNAITT

IVED---------T-FG------------LLTS-------------LSVLNVQ-GKHFFC

NCDL--Y-WF----VNVY--------------FHNPHL----------------------

-------------------QI--------NG-------KEDLRC-SFP-PD----RR---

-GLLVESS--NLTLLR-C-SLGL-------QMAITACVAILVVLVLTGL----C--WRFD

G-LW-YVKMG---WY---W-----CM----A-KR--------------------------

-------------KQY--------------------------E---KRPE-NK--P-FDA

FISYSEQDA---NWTKKNLLEKL----EAD----------------------------G-

-F-----KICYHERDFK----------------P--GH-PVLGNIF-YCIENSHKVLFVL

SP------SFV-----------NSC-------WCQY-ELYFAEHRVLN--E-N-QDSLIM

IVLE--D-LPPNSV----------------------------------------------

--PQKFSKLRK-LLKRKTYLKWS----PE-------EHKQK-------MFWLQLAAVL--

--RTT------------NE-----------------------------------------

------------------------PL-------VR-----AENGATQDVYEME-------

-----------------------------------------------------------

>TLR15_Stoc

------------------------------------------------------------

------------------------------------------------------------

------------------------------------------------------------

------------------------------------------------------------

------------------------------------------------------------

------------------------------------------------------------

------------------------MGVFLR--V---------------------------

-----------------FINIEPIIMMDFTFLFSRMRILVGSFHFY------LI--SFL-

--------------------------------------------LSRANGFLT--LR--T

-PAY-V------------------------------FPFYNYS-----------------

-----------Y------LN--LS-S-------LS----E-A-QA-----P-------K-

T--ARAL-------NFSHN-------I---I--E-----------KITRRDLEG--F-DV

L-----------EVLDLS-YNR------------------I-KD--IEP--GAFETLLSL

ISVNLS------F--------ND---KK-----------------------LL---V-SG

------LPPHLKLLPSSKASGSLQLY-EY-------FEKSS-----------E---AA--

----LESSAS------------A-------------------------------------

-------------EELPRSG-GPS-VLQNV-----------------NSRLRRSTGNLLP

RAEKNVTVSPT---ATSGPD-FC--------T-------------------------API

NGVLNLS-----HS---------------------------------------KLSE---

-EEL--TL---------KLD--PDLCQVQLD-STLELDI--SHSDLE----M-----D--

----------L-----LSL-------FIL---FLPM-------K--NVQSIDASY----N

KLT-----------INILDVEAIC--KFP--------------FSKLWF---------L-

-NI-SN--NPINSLDT---------------------LC---------L--PSTIKVIDL

------------S----F-----T--N-----------V-SQI------PPNFA-----K

KM----F---N--------LEH---MYVQGNHFIYTV-----------------------

-------------------------RPDAVPTVP-PGT-----------VRINAISLV--

-------------------------------RNQAG-------------------TPIES

L------------------------PKR--VKY-------LKMSNCSIVEL-PEWF--VG

TMEE--LIFLDLSSNW--------I-------------------------SVLP------

----------------DL----PTSLQHLDISNSD-------------I--K-----IIP

-PRFKSL--SN-LTIFNIQN-N-K---------ITAMHPEYFPSTLTKCDI-S--KNKLN

ML---LLTDA------------------------------P-----G-------------

--KLEYLNVSGNL--I----T-----------------RLEPGSH------LS-------

ALATL----------DSS-------HNLI----SE--L-----PDH-----FG--K-SLP

----------------------------------------------------------M-

---------------------LKYFNLSGNKISFL-QRGSLP---ASLVELDISDNAITT

IVED---------T-FG------------QLTS-------------LSVLSTQ-GKHFFC

NCDL--Y-WF----T--N--------------V----Y---IHD-PR-------------

-------V-----------QI--------IG-------KENLRC-SFP-PD----RR---

-GSLVGSS--NLTLLR-C-SLGI-------QMAITACAAILVVLTFTGL----C--WRFD

G-LW-YVRMG---WY---W-----CM----A-KR--------------------------

-------------KQY--------------------------E---KRPE-NK--P-FDA

FISYSEQDA---NWTKENLLRKL----ETD----------------------------G-

-F-----KICYHERDFK----------------P--GH-PILGNIF-YCIENSHKVLFVL

SP------SFV-----------NSC-------WCQY-ELYFAEHRVLN--E-N-QDSLIM

IVLE--D-LPPNSV----------------------------------------------

--PQKFSKLRK-LLKRKTYLKWS----PE-------EHKQK-------MFWHQLAAVL--

--KTT------------NE-----------------------------------------

------------------------PL-------VR-----AQNGPAQDVYEME-------

-----------------------------------------------------------

>TLR15_Meuni

------------------------------------------------------------

------------------------------------------------------------

------------------------------------------------------------

------------------------------------------------------------

------------------------------------------------------------

------------------------------------------------------------

---------------MKI------L--I-G--S---------------------------

--------------------------------L---H--F--Y--F------MS----V-

---LFSRANGSL----------------------------------------T--LR---

-TPS-AYA----------------------------FPFCNYT-----------------

-----------Y------LN--LS-S-------VS----E-A-QA-----P-------K-

T--ARAL-------NFSHN-------V---I--E-----------KITKRDLEG--F-DV

L-----------EVLDLS-YNQ------------------I-KD--IEP--GAFERLFSL

VSVNLS------F--------ND-K-KL-----------------------LV---P-GL

------P-PHP---KL---LPPSQAS-GS---LQL-YTYFD------KS---S---EA--

----AP-EPS------------A-------------------------------------

-----------SAKELPHLG-GPS-VLQNVNARLRRNTENLLRRAEKNTTASPAA----T

SRPDFCGAPVNGTLDLSNSK-LS----EE--E------------LSA---KLD----ANL

CQ-VQLE-----GI--------VELNISH------------------------SDLE---

-MDL--LS----L----FIL---------------FLPM----ENVQ----SID---A--

S--YN---K-L-T---INI-------QD----VEAI-------C--NFPLSKLSF----L

NIS----------NNPINSLDTLC---LP-S----------T-IQIIDL--SFT-NI-S-

-QI-PP--NFARKMMN---------------------LE---H---MYV--QGN-----H

-----------------F-I---Y--T-------V--RP-QIT----------N-----A

VA----K------------PP----PGTVHINA-ISF-----------------------

-------------------------------------------------VRNLNGTPI--

-------------------------------ESLPN------------------------

---------------------------K--LKH-------LKMSNCSIVEL-PEWF--VG

TMQE--LLFLDLSSNR--------I-S-V------L--------------PDLP--TSLQ

HLDISNSDIKIIPPSFKS--V-SN-LTIFNIQNNK-------------I--T-DM-----

-HPEYFP--LT-LTKCDISK-N-K--------------------------L-N---T---

--------LS----------L-T--DAL------------------G-------------

--KLEHLNVSGNL--I----T-RL--------------EPASYLS---------------

ALANL----------DSS-------RNLI----SE--L-----PDH-----FG--K-ALP

----------------------------------------------------------M-

---------------------LKYFNLSGNKISFL-QRGS--LP-ASLIELDISDNAITT

IVED---------T-FG------------QLTS-------------LSVLTVQ-GKHFFC

NCDL--Y-WF----V--N--------------V----Y-------MH-----------HP

-------H-----------LR--------IN-G-----KGNLRC-SFP-PD----RR---

-GSLVESS--RLTLLH-C-SLGI-----QMAIT--ACVAVLVVLVLTGL----C--WRFD

G-LW-YVRMG---WY---W-----CM----A--K------------------R-------

-------------KQY---------------E----KR----P---ENK---P----FDA

FVSYSEQDA---NWTKEHLLERL----ETD---G--------------------------

---F---KICYHERDFK----------------P--GH-PVLGNIF-YCIENSHKVLFVL

SP------SFV-----------NSC-------WCQY-ELYFAEHRVLN--E-N-QDSLIM

IVLE--D-LPPNSV----------------------------------------------

--PQKFSKLRK-LLKRKTYLKWS----PE-------EHKQK-------MFWHQLAAVL--

--KTT------------NE-----------------------------------------

-------------------P-LVKAE-------SG-----CA--LDMHEMV---------

-----------------------------------------------------------

>TLR15_Chma

------------------------------------------------------------

------------------------------------------------------------

------------------------------------------------------------

------------------------------------------------------------

------------------------------------------------------------

------------------------------------------------------------

---------------------------MRI--L---------------------------

-------------------------------IGSLHFYLISFL--L------SR--GNG-

----------------------------------------------------FLTPR--T

-PAA-HAV-----------------------------PFYNYS-----------------

-----------Y------LN--LS-S-------VS----E-A-QA-----P-------K-

M--ARTL-------DFSHN-------N---I--E-----------KITKRDLEG--F-DV

L-----------EVLDLS-HNQ------------------I-QD--IEP--GALASLLSL

VSVNLS------F--------ND---KK-----------------------LL---V-SD

------LPPHL---KL---LPTSEAS-GT---LQL-YRYLVKSPEAALE---P---SA--

----SAEELP------------R-------------------------------------

-------QGGPPVLQSVHPR-HRR-GTENLLRRGEKNTTI-------SPTVTLKPDLCGE

PVNGTLDLSNS---KLSEEE-LM---------------------------LKL---DPDL

CQ-AQLD----------------------------------------------GILE---

-LNI--SH----S----DLE--MDLLSLFML-FLPMTNV----RSID----A-----S--

Y--NR-----------LTINILDVEAIC----KFPS--------------SKLLF----L

NIS----------NNPINSLDTLC---LP------------STIKVIDL--SFT-NI-S-

-QI-PQ--NFANKMMN---------------------LE---N-----M--YVQ-----G

------------N----H----------------F---I-YTV------HPQIT---NAA

PK----P---P--------PGS---VHISAISF---------------------------

-------------------------------------------------VRNQAGTPV--

-------------------------------ESLPT------------------------

---------------------------K--VKH-------LKMSNCSIVEL-PEWF--AD

RMEE--LLVLDLSSNR--------I-------------------------SVLP------

----------------DL----PTSLQHLDISNSD-------------I--K-----VIP

-PSFKSV--SN-LTIFNIQN-N-K--------ITDMHPEYFPLTLTKCDIS-K--NKLNR

LS---LTDAL------------------------------------E-------------

--KLESLNVSGNL--I----T-----------------RLEPASH------LS-------

ALTNL----------DSS-------HNLI----SE--L-----PDH-----FG--K-SLP

----------------------------------------------------------M-

---------------------LKYFNLSGNKISFL-QRGSLP---ASLIELDISDNAITT

IVED---------T-FG------------LLTS-------------LSVLTVQ-GKHFFC

NCDL--Y-WF----V--N--------------V----Y---IHQ-PH-------------

-------L-----------QI--------NG-------KGNLRC-SFP-PE----RR---

-GLLVDSS--NLTLLH-C-SLGI-------QMAITACVAVLVVLVLTGL----C--WRFD

G-LW-YVRMG---WY---W-----CM----A-KR--------------------------

-------------KQY--------------------------E---KRPE-NK--P-FDA

FISYSEQDA---NWTKENLLKKL----EKD----------------------------G-

-F-----KICYHERDFK----------------P--GH-PVLGNIF-YCIENSHKVLFVL

SP------SFV-----------NSC-------WCQY-ELYFAEHRVLN--E-N-QDSLIM

IVLE--D-LPPNSV----------------------------------------------

--PQKFSKLRK-LLKRKTYLKWS----PE-------EHKQK-------MFWHQLAAVL--

--KTT------------NE-----------------------------------------

------------------------PL-------VR-----AENGSAQEMYELE-------

-----------------------------------------------------------

>TLR15_Bare

------------------------------------------------------------

------------------------------------------------------------

------------------------------------------------------------

------------------------------------------------------------

------------------------------------------------------------

------------------------------------------------------------

---------------MRILIGSLHFYFISF--L---------------------------

---------------------------------------FSRANGF------LT----L-

-------------------------------------------------------RT--P

-TAY-A------------------------------FPFYNYS-----------------

-----------Y------LN--LS-S-------VS----E-A-QA-----P-------K-

T--ARAL-------NFSHN-------V---I--E-----------KITKRDLEG--F-DV

L-----------EVLDLS-YNQ------------------I-KD--IEP--GAFESLLSL

VSVNLS------F--------ND---KK-----------------------LL---V---

--------PGL---PA---HLKLSLA-SEASGSLQLYKYFD------RS---P---EA--

----ASEPSA------------P-------------------------------------

-------------AEELPHA-GRP-SVLRNA----------------HPRLRRSTENLLR

RAEKNVTVSPT---TTLRPN-FC--------G-------------------------VPI

NGTLALS-----NS---KLSEEELRLKLDPDVCQAQL---------------DGILE---

-LDI--SH----N----DLE--MDLLLLFIL-FLPMKNM----QSVD----A-----S--

Y--NK-----------LTINIQDVEAICN--------------F--PF--SNFLF----L

NIS----------NNPINSLDTLC---LP------------STIKVIDL--SFT-NI-S-

-RI-PP--NFAKKMIN---------------------LE---N-----M--YVQ-----G

------------N----H----------------F---I-YTV------RPDVP------

NA-----------------VQM---FPPGTVRI-NAI-----------------------

--------------------------------------------------SFVRDEGG--

-------------------------------TPIKS------------------------

-------------------------LPN-KVKY-------LKMSNCSIVEL-PEWF--AG

TMGE--LLFLDLSSNR--------I-------------------------SVLP------

----------------DL----PTSLQHLDLSNSD-------------I--R-----AIP

-PSFKSV--SN-LTIFNIQN-N-K--------VTDMHPEHFPLTLTKCDIS-K--NKLNT

LS---LTDAL------------------------------------E-------------

--KLEYLNVSGNL--I----A-----------------RLEPSSH------LS-------

ALTNL----------DSS-------HNLI----SE--L-----PDR-----FG--K-SLP

----------------------------------------------------------T-

---------------------LRYFNLSGNKISFL-QRGSLP---ASLIELDISNNAITT

IVED---------T-FG------------QLTS-------------LSVLTVQ-GKHFFC

NCDL--Y-WF----V--N--------------V----Y---IHH-PR-------------

-------L-----------QI--------NG-------KGSLKC-SFP-PE----RR---

-GSLVESS--DLTLLR-C-SLGI-------QMAVTACVAILVVLVLTGL----C--WRFD

G-LW-YVRMG---WY---W-----CM----A-KR--------------------------

-------------KQY--------------------------E---KRPE-NK--P-FDA

FISYSEQDA---NWTKENLLKKL----ETD----------------------------G-

-F-----KICYHERDFK----------------P--GH-PVLGNIF-YCIENSHKVLFVL

SQ------SFV-----------NSC-------WCQY-ELYFAEHRVLN--E-N-QDSLIM

IVLE--D-LPPNSV----------------------------------------------

--PQKFSKLRK-LLKRKTYLKWS----PE-------EHKQK-------MFWHQLAAVL--

--KTT------------NE-----------------------------------------

------------------------PL-------VR-----AENGSAQDMYEME-------

-----------------------------------------------------------

>TLR15_Taer

------------------------------------------------------------

------------------------------------------------------------

------------------------------------------------------------

------------------------------------------------------------

------------------------------------------------------------

------------------------------------------------------------

---------------MRI------L--I-G--S---------------------------

--------------------------------L---H--F--Y--F------IS----F-

---LFSRTNGFL----------------------------------------T--LR---

-TPT-AHA----------------------------FPFYNYS-----------------

-----------Y------LN--LS-S-------VS----E-A-QA-----P-------K-

R--ARAL-------NFSHN-------V---I--E-----------KITQRDLEG--F-DT

L-----------EVLDLS-YNR------------------I-KD--VEP--GAFERLLSL

VSLNLS------F--------ND-K-KL-----------------------LV---L-GL

------P-PHL---KL---LPTSEAS-GS---LQL-YKYFD------ES---S---EA--

----AP-EPS------------A-------------------------------------

-----------SAEELPHSR-GSS-VLQNINLRLRRSTEN-LQRAEKNVTVSPTT----T

LKSNFCRAPINGILDLSNRK-LS----EA--E------------LGL---KLD----PDH

CQ-AQLD-----GI--------LELDISH------------------------SDLE---

-MDL--LS----L----FIL---------------FLPM----TNLQ----SVD---A--

S--SN---K-I-T---INT-------QD----VEAI-------C--KFPFSKLVF----L

NIS----------NNPINSLDTLC---LP-S----------T-IKVIDL--SFT-NI-S-

-QI-PQ--NFAKKMMN---------------------LE---N---MYV--HGN-----H

-----------------F-I---Y--T-------V--RP-AIT----------N-----A

PA----K------------PP----SGGVHINA-ISF-----------------------

-------------------------------------------------VRNQAGTPI--

-------------------------------ESLPK------------------------

---------------------------K--VKR-------LEASNCSIVEL-PEWF--AG

RMEE--LLFLDLSSNR--------I-S-V------L--------------PDLP--TSLQ

HLDISNSDIKIIPPRFKS--L-CN-LTIFTIQNNK-------------I--T-DM-----

-HPEYFP--LT-LTRCDISK-N-K--------------------------L-N---T---

--------LS----------L-T--DTL------------------E-------------

--KLEYLNVSGNL--I----S-RL--------------EAASHLS---------------

ALAIL----------DSS-------HNLI----SE--L-----PDH-----FG--K-SLP

----------------------------------------------------------A-

---------------------LKYFNLSGNKISFL-QRGS--LP-ASLTELDISDNAITT

IVED---------T-FG------------QLTS-------------LSVLTVQ-GKHFFC

NCDL--Y-WF----V--N--------------V----Y-------IR-----------NP

-------R-----------LR--------IN-G-----KGNLRC-SFP-PD----RR---

-GSLVESS--NLTLLR-C-SLGI-----QMAIT--ACVAILVVLVLTGL----C--WRFD

G-LW-YVRMG---WY---W-----CM----A--K------------------R-------

-------------KQY---------------E----KR----P---ENK---P----FDA

FVSYSEQDA---NWTKENLLQKL----ETD---G--------------------------

---F---KICYHERDFK----------------P--GH-PVLGNIF-YCIENSHKVLFVL

SP------SFV-----------NSC-------WCQY-ELYFAEHRVLN--E-N-QDSLIM

IVLE--D-LPPNSV----------------------------------------------

--PQKFSKLRK-LLKRKTYLRWS----PE-------EHKQK-------MFWRQLAAVL--

--KTT------------NE-----------------------------------------

-------------------P-LVRAE-------NG-----PA--QDTYEME---------

-----------------------------------------------------------

>TLR15_Caca

------------------------------------------------------------

------------------------------------------------------------

------------------------------------------------------------

------------------------------------------------------------

------------------------------------------------------------

------------------------------------------------------------

-----------MRILIGS------LHFYFI--S---------------------------

-------------------------------FLVSRTNGF-----L------TL------

-------------------------------------------------------RR--P

-TAH-A------------------------------FPFYNYS-----------------

-----------Y------LN--LS-S-------VS----E-A-QA-----P-------K-

T--AKAL-------NFSHN-------I---I--E-----------KITKRDLEG--F-DA

L-----------EVLDLS-YNQ------------------I-MD--IEP--GVFENLLSL

VSVNLS------F--------ND---KK-----------------------L--------

------FVSGLP--PH---LKVLPTS-KASGSLQL-YKYFDKSSEAALE---P---SASA

EELPHSGGPS------------V-------------------------------------

-------------LQNVNLR-LRR-STDNLQ----------------REEKNITVSPTTT

LSPDLCGAPTNGILNLSNSK-LS----EE--E-------------------------LML

KLDPDMC-----QD------------------------------------QLDGMLE---

-LDI--SH----N----DLE--MDLLSLFIL-FLPLKNV----QSID----A-----S--

Y--NK-----------LTINVQDVEAICK--------------F--PF--NKLFF----L

NIS----------NNPINSLDTLC---LP------------STIKVIDL--SFT-NI-S-

-QI-PQ--NFAKKLVN---------------------LE---N-----M--YVQ-----G

------------N----H----------------F---I-YTV------RPEIT------

NA-----------------VPN---LPPGTVRI-NAI-----------------------

--------------------------------------------------SFVRNQAG--

-------------------------------TPIES------------------------

-------------------------LPK-KVKH-------LKMSNCSIVEL-PEWF--AG

KMEE--LLFLDLSSNR----------------------------------ISVL------

----------------PV--L-PTSLQHLDISSSD-------------I--K-----IIP

-PSFKAL--SN-LTIFNIQN-N-K--------ITDMHPEYFPLTLTKCDIS-K--NKLNM

LS---LTDAM------------------------------------E-------------

--KLKYLNVSGNL--I----T-----------------RLEPNSQ------LS-------

ALVNL----------DSS-------HNLI----SE--L-----PDH-----FG--K-SLP

----------------------------------------------------------M-

---------------------LKYFNLSGNKISFL-QRGSLP---ASLIELDISDNAITT

IVED---------T-FG------------QLMS-------------LSVLTVQ-GKHFFC

NCDL--Y-WF----V--N--------------V----Y---IHN-PH-------------

-------L-----------QI--------NG-------KRNLRC-SFP-PD----RR---

-GSLVESS--NLTLLH-C-SLGI-------QMAITACVAILVVLVLTFL----C--WRFD

G-LW-YVRMG---WY---W-----CM----A-KR--------------------------

-------------KQY--------------------------E---KRPE-DK--P-FDA

FISYSEQDA---NWTKENLLQKL----ETD----------------------------G-

-F-----KICYHERDFK----------------P--GH-PVLGNIF-YCIENSHKVLFVL

SP------SFV-----------NSC-------WCQY-ELYFAEHRVLN--E-N-QDSLIM

IVLE--D-LPPNSV----------------------------------------------

--PQKFSKLRK-LLKRKTYLKWS----PE-------EHKQK-------MFWHQLAAVL--

--KTT------------NE-----------------------------------------

------------------------PL-------VR-----AENGSAHDMYEME-------

-----------------------------------------------------------

>TLR15_Phru

------------------------------------------------------------

------------------------------------------------------------

------------------------------------------------------------

------------------------------------------------------------

------------------------------------------------------------

------------------------------------------------------------

-----------MRILIGS------LHFYFI--S---------------------------

-------------------------------FLVSRANGF-----L------TL------

-------------------------------------------------------RT--P

-TAY-A------------------------------FPSYNYS-----------------

-----------Y------LN--LS-S-------IS----E-A-QA-----P-------K-

T--ARAL-------NFSHN-------V---I--E-----------KITKRDLEG--F-EA

L-----------EVLDLS-YNQ------------------I-ED--IEP--GVFESLLSL

VSVNLS------F--------ND---KK-----------------------LL---V-PS

------LPPHLKLLPTSKASGSLRLY-KY-------FDKSS---------------EA--

----AREPSA------------S-------------------------------------

-------------AEELPHS-GGP-SVLP------------------HVNPRLRRSTENL

LRRAEKNATAS---PI--AT-LR----PD---------------------FCG---APRN

GT-LDLS-----NS---KLSEEELMLKLDPDLCQAQL---------------DGILQ---

-LDI--SH----S----DLE--MDLLSLFVL-FLPMKNV----RSID----A-----S--

Y--NK-----LTINILDAE-------------AICH-------F--PF--SKLLF----L

NIS----------NNPINSLDTLC---LP------------STIKVIDL--SFT-NI-S-

-QI-PQ--NFAKKLFN---------------------LE---N-----M--YVQ-----G

------------N----H----------------F---I-YTV------RPEIT---SEA

PK----F---P--------PGT---VHINAISF---------------------------

-------------------------------------------------VRNQAGTPI--

-------------------------------ESLPQ------------------------

---------------------------K--VKH-------LRMSNCSIVEL-PEWF--AG

TMEE--LLFLDLSGNR--------I-------------------------SVLP------

----------------DL----PTSLRHLDISNSD-------------I--K-----TIP

-PSFKSI--SN-LTVFKIQN-N-K--------ITDMHPEYFPLTLTKCDIS-K--NKLNM

LS---LTEAL------------------------------------E-------------

--KLEYLNVSGNL--I----S-----------------RLEPASH------LP-------

ALANL----------DSS-------HNLI----SE--L-----PDH-----FG--K-SLP

----------------------------------------------------------T-

---------------------LKYFNLSGNKISFL-QRGSLP---ASLVELDISDNAITT

IVED---------T-FG------------QLTS-------------LSVLTVQ-GKHFFC

NCDL--Y-WF----V--N--------------V----Y---THH-PR-------------

-------L-----------QI--------NG-------RGDLRC-SFP-PD----RR---

-GSLVESS--DLTLLR-C-SLGI-------QMAVTACVATLAVLALTGL----C--WRFD

G-LW-YVRMG---WY---W-----CM----A-----------------------------

-------------KRK--------------------QYEKRPE---NKL--------FDA

FVSYSEQDA---NWTKEYLLEKL----END----------------------------G-

-F-----KICYHERDFK----------------P--GH-PVLGNIF-YCIENSHKVLFVL

SP------SFV-----------NSC-------WCQY-ELYFAEHRVLN--E-N-QDSLIM

IVLE--D-LPPNSV----------------------------------------------

--PQKFSKLRK-LLKRKTYLKWS----PE-------EHKQK-------MFWHQLAAVL--

--KTT------------NE-----------------------------------------

------------------------PL-------VR-----TENGAAQDV-----------

-----------------------------------------------------------

>TLR15_Nini

------------------------------------------------------------

------------------------------------------------------------

------------------------------------------------------------

------------------------------------------------------------

------------------------------------------------------------

------------------------------------------------------------

-----------MRILIGS------LHFYFI--S---------------------------

-------------------------------VLFSRANGF-----L------AW------

-------------------------------------------------------RT--P

-TAY-A------------------------------FPFYNYS-----------------

-----------Y------LN--LS-S-------VP----E-A-QA-----P-------K-

T--ARAL-------NFSHN-------I---I--E-----------KITKRDLEG--F-DM

L-----------EVLDLS-YNQ------------------I-KD--IEP--GAFERLLSL

VSVNLS------F--------ND---KK-----------------------LL---L---

--------SGLP--PH---LKLLPASEASGSLQLSKYADKS-----------P---AA--

----AREPSA------------S-------------------------------------

-------------AEELPRA-GGP-SVLH------------------NVNLRLRRSTENL

LRRAEKNVTAS---PA--PT-VR----PD---------------FCG----------APV

NGILDLS-----NS---KLSEEELTLKLDPDNCQAQL---------------DAILE---

-LDI--SH----S----DLE--MDLLSLFVL-FLPMKNV----QSID----A-----S--

Y--NK-----------LTINVLDVEAICN--------------F--PF--SKVLF----L

NIS----------NNPINSLDTLC---LP------------STIKVIDL--SFT-NI-S-

-QI-PQ--NFATKMLN---------------------LE---N-----M--YVQ-----G

------------N----H----------------F---I-YTV------RPEIT---NAV

QK----F---P--------PGS---VHINAISF---------------------------

-------------------------------------------------VRNQAGTPI--

-------------------------------ESLPK------------------------

---------------------------K--VKQ-------LKMSNCSIVEL-PEWF--AG

TMEE--LLFLDLSSNR--------I-------------------------SVLP------

----------------NL----PASLQHLDISNSD-------------I--K-----IIP

-PGFKSV--SN-LTIFNIQN-N-K--------ITDMHPEYFPLTLTKCDIS-K--NKLSM

LS---LTDAL------------------------------------E-------------

--NLEYLNVSGNL--I----T-----------------RLEPASH------LS-------

ALANL----------DSS-------HNLI----SE--L-----PDH-----FG--K-SLP

----------------------------------------------------------M-

---------------------LKYFNLSGNKISFL-QRGALP---ASLIELDISDNAITT

IVED---------T-FG------------QLTS-------------LSVLTVQ-GKHFFC

NCDL--Y-WF----V--N--------------V----Y---IHD-PR-------------

-------L-----------QI--------NG-------KGNLRC-SFP-LE----RR---

-GSLVERS--NLTLLH-C-SLGI-------QMAITACVAILVVLVLTGL----C--WRFD

G-LW-YVRMG---WY---W-----CM----A-KR--------------------------

-------------KQY--------------------------E---KRPE-NK--P-FDA

FVSYSEQDA---NWTKESLLEKL----ETD----------------------------G-

-F-----KICYHERDFK----------------P--GH-PVLGNIF-YCIENSHKVLFVL

SP------SFV-----------NSC-------WCQY-ELYFAEHRVLN--E-N-QDSLIM

IVLE--D-LPPNSV----------------------------------------------

--PQKFSKLRK-LLKSKTYLKWS----PE-------EHKQK-------MFWHQLAAIL--

--KTT------------NE-----------------------------------------

------------------------PL-------VR-----AEDRPAQDTYEME-------

-----------------------------------------------------------

>TLR15_Gast

------------------------------------------------------------

------------------------------------------------------------

------------------------------------------------------------

------------------------------------------------------------

------------------------------------------------------------

------------------------------------------------------------

---------------------------MMI--L---------------------------

-------------------------------IGSLHFYFISSL--LSRANGFLT----L-

-------------------------------------------------------RT--P

-TAH-V------------------------------FPFYNYS-----------------

-----------Y------LN--LS-S-------LA----E-A-QA-----P-------K-

T--ARAL-------NFSHN-------V---I--E-----------KITKKDLEG--F-DA

L-----------EVLDLS-YNR------------------I-KD--IEP--GAFERLLSL

VSVNLS------F--------NDKT-LL-----------------------VP---G---

------FPPHLKLLPTSEASGSLQLS-KY-------FDKSP-----------EAAPEP--

-------SAS------------A-------------------------------------

-------------EELPHPG-GPS-ALQNV-----------------NLQLRQSTENLLR

RAEKNIMVSPT---ATLKSD-FCRAPINGTLD------------LSNSKLSEE---ELML

KLDPDLC-----QA------------------------------------QLDSILE---

-LDI--SH----S----DLE--MDLLSLFVL-FLPMKNL----QSID----A-----S--

Y--NK-----------LTINILDVEGFCN--------------F--SF--SKLLF----L

NIS----------NNPINSLDTLC---LP------------STIKVIDL--SFT-NI-S-

-QI-PH--NFAKKMFN---------------------LE---N-----M--YVQ-----G

------------N----H----------------F---I-YTV------RPEVT---NAV

PK----F---P--------PGT---VHINAISF---------------------------

-------------------------------------------------VRNQAGTPI--

-------------------------------ESLPK------------------------

---------------------------K--VKH-------LKMSNCSIVEL-PEWF--AG

TMEE--LLFLDLSSNR--------I-------------------------SVLP------

----------------DL----PTSLQHLDLSNSD-------------I--K-----IVP

-PSFKSV--SN-LTVFNIQN-N-K--------ITDMHPEYFPLTLTKCDIS-K--NKLNM

LS---LTDAL------------------------------------E-------------

--KLEYLNVSGNL--I----T-----------------RLEPGSR------LS-------

ALANL----------DSS-------HNLI----SE--L-----PDD-----LG--K-SLP

----------------------------------------------------------M-

---------------------LKYFNLSGNKISFL-QRGSLP---ASLIELDISDNAITT

IVED---------T-FG------------QLTS-------------LSVLTVQ-GKHFFC

NCDL--Y-WF----V--N--------------V----Y---IHH-PH-------------

-------L-----------QI--------NG-------KGNLRC-SFP-PD----RR---

-GSLVESS--NLTLLH-C-SLGI-------QMAITACVAILVVLVLTGL----C--WRFD

G-LW-YVRMG---WY---W-----CM----A-KR--------------------------

-------------KQY--------------------------E---KRPE-NK--P-FDA

FVSYSEQDA---NWTKENLLEKL----ETD----------------------------G-

-F-----RICYHERDFK----------------P--GH-PVLGNIF-YCIENSHKVLFVL

SP------SFV-----------NSC-------WCQY-ELYFAEHRVLN--E-N-QDSLIM

IVLE--D-LPPNSV----------------------------------------------

--PQKFSKLRK-LLKRKTYLKWS----PE-------EHKQK-------MFWHQLAAVL--

--KTT------------NK-----------------------------------------

------------------------PL-------VR-----AEKGSAEDMYEME-------

-----------------------------------------------------------

>TLR15_Ledi

------------------------------------------------------------

------------------------------------------------------------

------------------------------------------------------------

------------------------------------------------------------

------------------------------------------------------------

------------------------------------------------------------

---------------MRILIGSFRFYFISF--L---------------------------

---------------------------------------FSRANGF------LT----L-

-------------------------------------------------------RR--P

-TAY-A------------------------------FPVCNYS-----------------

-----------Y------LN--LS-S-------VS----E-A-QA-----P-------K-

T--ARVL-------NFSHN-------I---I--E-----------KITKKDLEG--F-DA

L-----------EVLDLS-YNQ------------------I-KD--IEP--GAFESLLSL

VSVNLS------S--------ND---RK-----------------------LL---V-SG

------LPPHLKLLPTSKASGSLQLS-KY-------FDKSS------EA---A---PE--

----FS-ASA--------------------------------------------------

-------------EELPHSG-GPS-VLQNV-----------------NPRLRRSTVNLLR

RAERNVTVSPT-------AT-LR----PNPCG-------------------------API

NGILNLS-----NS---QLSEEELMLKLDPDLCQAQL---------------DSILE---

-LDI--SH----S----DLE--MDLLSLFVL-FLPMKNV----QSID----A-----S--

Y--NK-----------LTINILDVEAICN--------------F--PF--TTLLF----L

NIS----------NNPINSLDTLC---LPPT------------IKVIDL--SFT-NI-S-

-QI-PQ--NFAKKMFD---------------------LE---N-----M--YVQ-----G

------------N----H----------------F---I-YTV------RPEIT---NGV

SK----I---P--------PGT---VHINAISF---------------------------

-------------------------------------------------VRNQAGTPI--

-------------------------------ESLPK------------------------

---------------------------K--VKH-------LKMSNCSIVEL-PEWF--AD

TMEE--LLFLDLSSNR--------I-------------------------SVLP------

----------------DL----PASLQHLDISNSD-------------I--K-----VIP

-PSFKSV--SN-LTIFNIQN-N-K--------ITDVHPEYFPLTLTKCDIS-K--NKLNM

LS---LTDAL------------------------------------E-------------

--KLEYLNVSGNL--I----T-----------------RLKPASH------LS-------

ALANL----------DSS-------HNLI----SE--L-----PDH-----FG--K-SLP

----------------------------------------------------------T-

---------------------LKYFNLSGNKISFL-QRGSLP---ASLIELDISNNAITT

IVEN---------T-FG------------QLTS-------------LSVLTVQ-GKHFFC

NCDL--Y-WF----V--N--------------V----Y---IRN-PR-------------

-------L-----------QI--------NG-------KENLRC-SFP-PD----RR---

-GSLVESS--NLTLLH-C-SLGI-------QMAITACVAILVVLVLTGL----C--WRFD

G-LW-YVRMG---WY---W-----CM----A-KR--------------------------

-------------KQY--------------------------E---KRPE-NK--P-FDA

FISYSEHDA---TWTKENLLEKL----ETD----------------------------G-

-F-----KICYHERDFK----------------P--GH-PVLGNIF-YCIENSHKVLFVL

SP------SFV-----------NSC-------WCQY-ELYFAEHRVLN--E-N-QDSLIM

IVLE--D-LPPNSL----------------------------------------------

--PQKFSKLRK-LLKRKTYLKWS----PE-------EHKQK-------MFWRQLAAVL--

--QTT------------NE-----------------------------------------

------------------------PL-------VR-----AENGPAQDVYEME-------

-----------------------------------------------------------

>TLR15_Haal

------------------------------------------------------------

------------------------------------------------------------

------------------------------------------------------------

------------------------------------------------------------

------------------------------------------------------------

------------------------------------------------------------

---------------MRILTRTLHFYFISF--L---------------------------

---------------------------------------LSRANGF------LT----L-

-------------------------------------------------------RT--P

-TAY-T------------------------------FPFYNYS-----------------

-----------Y------LN--LS-S-------VS----E-A-QA-----P-------K-

M--ARAL-------NFSHN-------V---I--E-----------KITKRDLEG--F-DA

L-----------EVLDLS-YNQ------------------I-KD--IEP--GAFESLPSL

VSVNLS------F--------NKNL--------------------------LV---P-GL

------P-PHLKLLPTSKASGSLRLS-KY-------FDKPS-----------E---AA--

----LEPSVS------------A-------------------------------------

-------------EELPHLG-GPS-ILQNV-----------------NPRLRRSTENLLR

RPEKNVTVSPT---ATLKPD-FC--------G-------------------------API

NGILDLS-----NS---KLSEEELMLKLDPDLCQIQL---------------DGILE---

-LDI--SH----S----DLE--MDLLSLFVL-FIPMKNL----QSVD----A-----S--

Y--NK-----------LTINILDVEAICN--------------F--PF--SKLLF----L

NIS----------NNPINSLDTLC---LP------------STIKVIDL--SFT-NI-S-

-QI-PP--NFAKKMSD---------------------LQ---N-----M--YVQ-----G

------------N----H----------------F---I-YTV------RPETT---NAA

PK----F---P--------PGT---VHINAISF---------------------------

-------------------------------------------------VRNQAGTPI--

-------------------------------ESLPK------------------------

---------------------------K--VKH-------LKMSNCSIVEL-PEWF--AG

TMEE--LLFLDLSSNR----------------------------------ISVL------

----------------PV--L-PASLQHLDISNSD-------------L--K-----LIP

-PSFKSV--SN-LTIFNIQN-N-K--------ITDMHPEYFPSTLTKCDIS-K--NKLNM

LS---LTDAL------------------------------------E-------------

--KLEHLNVSGNL--I----T-----------------RLEPASH------LS-------

ALANL----------DSS-------HNLI----SE--L-----PDH-----FG--K-SLP

----------------------------------------------------------I-

---------------------LKYFNLSGNKISFL-QRGSLP---ASLIELDISDNAITT

IVED---------T-FG------------QLTS-------------LSVLTVQ-GKHFFC

NCDL--Y-WF----V--N--------------I----Y---IHN-PH-------------

-------L-----------QI--------NG-------KGNLRC-SFP-PD----RR---

-GSLVESS--NLTLLH-C-SLGI-------QMAITACVAILVVLVLTGL----C--WQFD

G-LW-YMRMG---WY---W-----CM----A-KR--------------------------

-------------KQY--------------------------E---KRPE-NK--P-FDV

FISYSEEDA---NWTKEHLLEKL----ETD----------------------------G-

-F-----KICYHERDFK----------------P--GH-PVLGNIF-YCIENSHKVLFVL

SP------SFV-----------NSC-------WCQY-ELYFAEHRVLN--E-N-QDSLIM

IVLE--D-LPPNSV----------------------------------------------

--PQKFSKLRK-LLKRKTYLKWS----PE-------EHKQK-------MFWHQLAAVL--

--KTT------------NE-----------------------------------------

------------------------PL-------VR-----AENGSAQDMYEME-------

-----------------------------------------------------------

>TLR15_Apfo

------------------------------------------------------------

------------------------------------------------------------

------------------------------------------------------------

------------------------------------------------------------

------------------------------------------------------------

------------------------------------------------------------

---------------MRILTGSLHFYFISF--L---------------------------

---------------------------------------FSRANGF------LT----P-

-------------------------------------------------------RT--P

-TAY-A------------------------------FPFYNYS-----------------

-----------Y------LN--LS-S-------VS----E-A-QA-----P-------K-

T--ARAL-------NFSHN-------V---I--E-----------KITKRDLEG--F-DT

L-----------EVLDLS-YNQ------------------I-KD--IEP--GAFERLLSL

VSVNLS------F--------NA---KK-----------------------LL---V-SG

------LPPHL---KL---LPTSEAS-GSLQLCRY-FDGSP-----------E---AA--

----LE-PSA------------S-------------------------------------

-------------IEELPHS-GGP-SVPQ------------------NVNLRLRRSTENL

LRRAEKNVTVS---PT--AT-LR----PN---------------FCG----------API

NGILDLS-----NS---KLSEEELTLKLDPDLCQAQL---------------DSILE---

-LDI--SH----S----DLE--MDLLSLFVL-FLPMKNV----QSID----A-----S--

Y--NK-----LTINALDAE-------------AICS-------F--PF--SKLLF----L

NIS----------NNPINSLDTLC---LP------------STIKVIDL--SFT-NI-S-

-QI-PQ--NFAKKMFN---------------------LE---N-----M--YVQ-----G

------------N----H----------------F---I-YTV------RPEIT---NAV

PK----F---P--------PGT---VRINAISF---------------------------

-------------------------------------------------VRNQAGTPI--

-------------------------------KSLPK------------------------

---------------------------K--VKR-------LKMSNCSIVEL-PEWF--AD

TVEE--LLFLDLSSNR--------I-------------------------SVLP------

----------------DL----PTSLKHLDISNSD-------------I--K-----IIP

-PNFKSV--SN-LTIFSIQN-N-K--------IVDMHPEYFPLTLTKCDIS-K--NKLNT

LS---LTDAL------------------------------------E-------------

--KLKYLNVSGNL--I----T-----------------RLEPTSH------LS-------

ALANL----------DSS-------HNLI----SE--L-----PDH-----FG--K-SLP

----------------------------------------------------------T-

---------------------LKYFNLSGNKISFL-QRGALP---GSLVELDISDNAITT

IVED---------T-FG------------QLTS-------------LSVLTVQ-GKHFFC

NCDL--Y-WF----V--N--------------T----Y---IHN-PH-------------

-------L-----------QI--------NG-------KGNLRC-SFP-PD----RR---

-GSLVESS--NLTLLR-C-SLGI-------QMAITACAAILVVFLLTGL----C--WRFD

G-LW-YVRMG---WY---W-----CM----A-----------------------------

-------------KRK--------------------QYEKRPE---EKL--------FDA

FVSYSEQDA---NWTKENLLEKL----ETD----------------------------G-

-F-----KICYHERDFK----------------P--GH-PVLGNIF-YCIENSHKVLFVL

SP------SFV-----------NSC-------WCQY-ELYFAEHRVLN--E-N-QDSLIM

IVLE--D-LPPNSV----------------------------------------------

--PQKFSKLRK-LLKRKTYLKWS----PE-------EHKQK-------VFWHQLAAVL--

--KTT------------NE-----------------------------------------

------------------------PL-------VR-----AENGSARDMYEME-------

-----------------------------------------------------------

>TLR15_Fugl

------------------------------------------------------------

------------------------------------------------------------

------------------------------------------------------------

------------------------------------------------------------

------------------------------------------------------------

------------------------------------------------------------

------------------------MRILIGSLH---------------------------

---------------------------------------FYFISFL------FS----R-

----------------------------------------------------ANGFLTLR

TPTAYA------------------------------FPFYNYS-----------------

-----------Y------LN--LS-S-------VS----K-A-QA-----P-------K-

T--ARAL-------NFSHN-------V---I--E-----------KITKRDLEG--F-DM

L-----------EVLDLS-YNQ------------------I-KD--IEP--GAFERLLSL

VSVNLS------F--------ND---KK-----------------------LL---V-SG

------LPPHLKLSPASEASGSLQLY-KY-------FDKSP-----------E---AA--

----LE-PSA------------S-------------------------------------

-------------AEELPHS-GGPSVLQNV-----------------HLRPRRSTEHLLR

RAEKNVTVSPT---AALRPN-FC--------G-------------------------API

NGILDLS-----NSKLCEEELTLKLDPNLCQAQLE------------------SILE---

-LDI-------------SHTDLEMDLLSLSVLFLPMKNV----QSVD----A-----S--

Y--NK-----LTINVLDAE-------------AICN-------F--PF--SKLLF----L

NIS----------NNPINSLDTLC---LP------------STIKVIDL--SFT-NI-S-

-QIPQNFAKKMFNLEN--------------------------------MYVQGN------

------------D---------------------FIYTVRPEI------TNAVTKFPPGT

VH-----------------INA---ISFVRNQAGTPI-----------------------

------------------------------------------------------------

-------------------------------ESLPK------------------------

---------------------------K--VKH-------LKMSNCSIVEL-PEWF--AG

TMEE--LLFLDLSSNR--------I-------------------------AVLP------

----------------DL----PTSLRHLDISNSD-------------I--K-----IIP

-PSFKSV--SN-LTIFNIQN-N-K---------ITDMHPEYFPLTLTKCDISKNKLNLLS

LT-----------------------DAL------------------Q-------------

--KLEYLNVSGNL--I----T-----------------RLEAASH------LS-------

ALTNL----------DSS-------HNLI----SE--L-----PDR-----FG--E-SLP

----------------------------------------------------------M-

---------------------LKYFNLSGNKISFL-QRGSLP---ASLIELDISNNAITT

IVED---------T-FG------------QLTS-------------LSVLTVQ-GKHFFC

NCDL--Y-WF----V--N-------------------V-------YI-------------

-------HHPHL-------QI--------NG-------KGHLRC-SFP-PD----RR---

-GSLVESS--NLTLLR-C-SLGI-------QMAITACVAILVVLALTGL----C--WRFD

G-LW-YVRMG---WY---W-----CM----A-----------------------------

------KRKQYEKRPE------------------------------NKL--------FDA

FVSYSEQDA---HWTKENLLEKL----ETD----------------------------G-

-F-----RICYHERDFK----------------P--GH-PVLGNIF-YCIENSHKVLFVL

SP------SFV-----------NSC-------WCQY-ELYFAEHRVLN--E-N-QDSLIM

IVLE--D-LPPNSV----------------------------------------------

--PQKFSKLRK-LLKRKTYLKWS----PE-------EHKQK-------MFWRQLAAVL--

--KTT------------NEPLVRAENGSAQDMYEME------------------------

------------------------------------------------------------

-----------------------------------------------------------

>TLR15_Cacr

------------------------------------------------------------

------------------------------------------------------------

------------------------------------------------------------

------------------------------------------------------------

------------------------------------------------------------

------------------------------------------------------------

---------MRILI-GS----------LHF--Y---------FI----------------

-------------------------------SF---LFSR--A--N------GF----L-

----------------------------------------------------T--LR---

-TPT-AYA----------------------------FPFYNYS-----------------

-----------Y------LN--LS-S-------VS----E-A-QA-----P-------K-

L--ARAL-------NFSHN-------I---I--E-----------KITKRDLEG--F-DA

L-----------EVLDLS-YNQ------------------I-KD--IEP--GAFESLLSL

VSVNLS------F--------ND-K-KL-----------------------LV---S-SL

------P-PHL---KL---LPTSKAS-GS---LQL-YKYFD------KSLEAA-------

----LEPSAS------------A-------------------------------------

-------------EELPHSG-GPS-VLQNVNPRLRRSTGNLLRRAEKNVTVTPTA----T

LRPNFCGAPINGILDLSNSK-LS----EE--E------------LTF---KLD----PDL

CQ-AQLD-----GI--------LELNISH------------------------SDLE---

-MDL--LS----L----FVL---------------FLPM----KNVQ----SID---A--

S--YN---K-L-T---INI-------LD----VEAI-------C--NFPFSKLLF----L

NIS----------NNPINNLDTLC---LP-P----------T-IKVIDL--SFT-NI-S-

-QI-PQ--NFAKKMLN---------------------LE---N---MYV--QGN-----H

-----------------F-I---Y--T-------V--RP-EDT----------N-----A

AP----K------------LP----PGTVRINA-ISF-----------------------

-------------------------------------------------VRNQAGTPI--

-------------------------------ESLPK------------------------

---------------------------T--VKH-------LKMSNCSIVEL-PEWF--AG

TMKE--LLFLDLSSNR--------I-------------------------SVLP------

----------------DL----PTSLQHLDISNSD-------------I--K-----IIP

-SSFKSF--SN-LTIFNIQN-N-K--------ITDM---HPEYFP-----L-A--LTKCD

IS-----KNK----------L-S---V------L-SLTDAL-----E-------------

--KLEFLNVSGNL--I----T-----------------RLEPTSHLS-------------

ALANL----------DSS-------HNLI----SE--L-----PDH-----FG--K-SLP

----------------------------------------------------------M-

---------------------LKYFNLSGNKISFL-QHGS--LP-ASLIELDISDNAITT

IVED---------T-FG------------QLTS-------------LSVLTVQ-GKHFFC

NCDL--Y-WF----V--N--------------A----Y---IH--NP-------------

-------R----L------QI--------NG-------KGDLRC-SFP-PD----RR---

-GWLVESS--NLTLLR-C-SLGI-----QMAVT--ACVAILVVLVLTGL----C--WRFD

G-LW-YVRMG---WY---W-----CM----A--K--------------------------

------R------KQY---------------E----KR----P---ENK---P----FDA

FISYSEYDA---NWTKENLLEKL----ETD---G-F------------------------

-------KICYHERDFK----------------P--GH-PVLGNIF-YCIENSHKVIFVL

SP------SFV-----------NSC-------WCQY-ELYFAEHRVLN--E-N-QDSLIM

IVLE--D-LPPNSL----------------------------------------------

--PQKFSKLRK-LLKRKTYLKWS----PE-------EHKQK-------MFWHQLAAVL--

--KTT------------NE-----------------------------------------

------------------------PL-------VR-----AENGSA--QDIYEMK-----

-----------------------------------------------------------

>TLR15_Opho

------------------------------------------------------------

------------------------------------------------------------

------------------------------------------------------------

------------------------------------------------------------

------------------------------------------------------------

------------------------------------------------------------

---------MRILI-GS----------LHF--Y---------FI----------------

-------------------------------SF---FFSR--A--N------GF----L-

----------------------------------------------------T--LR---

-TPA-AYA----------------------------FPFYNYS-----------------

-----------Y------LN--LS-S-------VS----E-A-QA-----P-------K-

T--ARAL-------NFSHN-------I---I--E-----------KISKRDLEG--F-DA

L-----------EVLDLS-HNR------------------I-KN--IEP--GAFESLLSL

VSVNLS------F--------NH-K-EL-----------------------LA---S-GL

------P-PYL---KL---LPTSKAS-GS---LQL-YKYFD------KSSEGA-------

----LEPPAS------------A-------------------------------------

-------------EELPHSG-GPS-VLQNVNPRLRRSTEHLLRRAEKNVTVSPEA----T

SRSNFCGAPINGTLDLSNSK-LS----EE--E------------LTL---KLD----PDL

CQ-AQLE-----GI--------LELDISH------------------------SDLE---

-MDL--LS----L----FVL---------------VLPM----KNVQ----SID---A--

S--YN---K-L-T---INI-------LD----VEAI-------C--NFPFSKLLF----L

NIS----------NNPINSLDTLC---LP-S----------T-IKVIDL--SFT-NL-S-

-QI-PQ--NFAKKMIN---------------------LE---N---MYV--QGN-----H

-----------------F-I---Y--T-------V--RP-EIG----------N-----G

VS----K------------FP----PGTVHINA-ISF-----------------------

-------------------------------------------------VRNEAGTPI--

-------------------------------QSLPQ------------------------

---------------------------K--VRH-------LKMSNCSIVEL-PEWF--AG

TMEE--LLFLDLSSNR--------I-------------------------SVLP------

----------------DL----PTSLQHLDISNSD-------------I--K-----IIP

-PSFKSV--SN-LTVFNIQN-N-K--------IMDM---HPEYFP-----L-T--LTKCD

IS-----KNK----------L-N---M------L-SLTDAL-----E-------------

--KLEYLNVSGNL--I----T-----------------RLEPASHLS-------------

ALANL----------DSS-------HNLI----SE--L-----PDH-----FG--K-SLP

----------------------------------------------------------M-

---------------------LKYFNLSGNKISFL-QRGA--LP-ASLIELDISDNAITT

IVED---------T-FG------------QLTS-------------LSVLTVQ-GKHFFC

NCDL--Y-WF----V--N--------------V----Y---IR--NP-------------

-------H----L------QI--------NG-------KGNLRC-SFP-PD----RR---

-GSLVESS--NLTLLH-C-SLGI-----QMAIT--ACVAILVVLVLTGL----C--WRFD

G-LW-YMRMG---WY---W-----CM----A--K--------------------------

------R------KQY---------------E----KR----P---ENK---P----FDA

FISYSEQDA---KWTKENLLEKL----ETD---G-F------------------------

-------KICYHERDFK----------------P--GH-PVLGNIF-YCIENSHKVLFVL

SP------SFV-----------NSC-------WCQY-ELYFAEHRVLN--E-N-QDSLIM

IVLE--D-LPPNSV----------------------------------------------

--PQKFSKLRK-LLKRKTYLKWS----PE-------EHKQK-------MFWHQLAAVL--

--KTT------------NE-----------------------------------------

------------------------PL-------VR-----AENGSA--QDMYEME-----

-----------------------------------------------------------

>TLR15_Phle

------------------------------------------------------------

------------------------------------------------------------

------------------------------------------------------------

------------------------------------------------------------

------------------------------------------------------------

------------------------------------------------------------

-----------MRILIGS------LHFYFISFL---------------------------

---------------------------------------FSRANGF------LT----L-

-------------------------------------------------------RT---

-PAAHV------------------------------FPFYNYS-----------------

-----------Y------LN--LS-S-------VS----E-A-QA-----P-------K-

T--ARAL-------NFSHN-------V---I--E-----------KITKRDLEG--F-DA

L-----------EVLDLS-YNR------------------I-KD--VEP--GAFESLLSL

VSANLS------F--------ND---KK-----------------------LL-------

------V-SGL--------PPHLKLL-PTGALQLYKYFDKS------SE-------AA--

----LE-PSA------------S-------------------------------------

-------------AQELRHS-GGPSVLQNG-----------------NPRLRRSTENLLR

RAEKNVTVSPA---ATLRPN-FC--------G-------------------------API

NGILDLS-----NSKLSEEELTLKLDPDLCQAQLDGI-----------------------

-LEL-------------DVSHSELEMDLLSL-FVLFLPM----KNLH----------SVD

ASYNN-----------LTINILDVEAICN--------------F--PF--SKLLF----L

NIS----------NNPINSLDTLC---LP------------STIKVIDL--SFT-NI-S-

-QIPQNFAQKMFNLEN--------------------------------MYVQGN------

------------H---------------------F---I-YTV------RPAIT------

NA-----------------VPK---IPPGTVHI-NAI-----------------------

--------------------------------------------------SFVRNEAG--

-------------------------------TPIES------------------------

-------------------------LPK-KVKH-------LKMSNCSIVEL-PEWF--AG

TMEE--LLFLDLSSNR--------I-------------------------SVLP------

----------------DL----PTSLQHLDISNSD-------------I--K-----IIP

-PSFKSV--SN-LTIFNIQN-N-K---------ITDLHPEYFPLTLTKCDISKNRLNLLS

LT-----------------------DAL------------------E-------------

--KLEYLNVSGNL--I----T-----------------RLEPASH------LA-------

ALANL----------DSS-------HNLI----SE--L-----PDH-----FE--K-SLP

----------------------------------------------------------M-

---------------------LKYFNLSGNKISFL-QRGSLP---ASLIELDISDNAITT

IVED---------T-FG------------QLTS-------------LSVLTVQ-GKHFFC

NCDL--Y-WF----V--N-------------------V-------YI-------------

-------HDPHL-------QI--------NG-------KGNLRC-SFP-PE----RR---

-GSLVESS--NLTLLR-C-SLGI-------QMAITACVAILVVLVLTGL----C--WRFD

G-LW-YVRMG---WY---W-----CM----A-KR--------------------------

-------------KQY------------------------------EKRPENK--P-FDA

FISYSEQDA---NWTKENLLEKL----ETD----------------------------G-

-F-----KICYHERDFK----------------P--GH-PVLGNIF-YCIENSHKVLFVL

SP------SFV-----------NSC-------WCQY-ELYFAEHRVLN--E-N-QDSLIM

IVLE--D-LPPNSV----------------------------------------------

--PQKFSKLRK-LLKRKTYLKWS----PE-------EHKQR-------MFWHQLAAVL--

--KTT------------NEPLVRAENGSAQDMYEME------------------------

------------------------------------------------------------

-----------------------------------------------------------

>TLR15_Geja

------------------------------------------------------------

------------------------------------------------------------

------------------------------------------------------------

------------------------------------------------------------

------------------------------------------------------------

------------------------------------------------------------

------------------------MGIFTRGLY---------------------------

------------------------------------------FYFI------IF----F-

----------------------------------------------------CNGKGEFK

HPGDEI---------------------------FVSTTTSNCS-----------------

-------------------------S-------VP----E-S-QT-----P---------

---RRVI-------NVFHV-----------I--K-----------YLTKRKGAV--H-SD

P-----------KGVNSS-HNQ------------------N-FQ--VESTAVSFNNLPQE

MSDQNS------H--------QK-L-SQ----------------------------T---

------F-KTT---DS---PKTEESL-WD-------TTGPP------MPSEKQ---QGDK

RLLNIN-PML------------K-------------------------------------

-------------QSALGHK-RPDKQETSF-----------------LPSALMGENGCGV

LAKGMLDLSNR---KLSDSK-LK----------------------------------EKM

DS-WHCR-----AT---------------------------------------VDKI---

-RDL-NAS---HN----DLN--MDLKALISL-LLKMKNV----HSID----V-----S--

Y--NK-----ITNSGTHRT-------------TICN-------L--EP--SNLLF----L

NLS----------HNPLRTLDNLC---LP------------STLKSIDL--SFT-SI-N-

-KIPKEFSGEFFHLEE---------------------MY---------IQGNHF-----I

----------------------------------YKPQL-YGS-----------------

GF----F------------LQG---LTAKPVIFRPEV-----------------------

-------------------------------------------------ISHIDVPET--

--------------------------------PIQS------------------------

-------------------------LPH-QVKH-------LQISNCSIVEL-PGWF--TN

TMAN--LLFLDLSNNP--------L-------------------------NSFP------

----------------QL----PASLQHLDLNNTN----------------------TES

VIDISHF--SN-LTVLHIQN-N-K--------MTGRLPSEHLPFALKELDVSKNKLHMFP

LQ-----------------------EAQ------------------Q-------------

--KLESLNVSGNL--I----TELY--------------LNTSFP----------------

SLTNL----------DVS-------HNMI----TE--L-----SDH-----TG--E-FLP

----------------------------------------------------------A-

---------------------LKYFNLSGNKISFL-QPGSLP---QSLLELDISNNAITI

IMKE---------T-FN------------NLQN-------------LQLLTVQ-GKNFFC

NCDL--Y-WF----VNTY-------------------L-------SS-------------

-------T--HV-------QV--------NG-------REDMLC-SYP-PN----KW---

-GLLVEHS--NLTLLN-C-SLGL-------QMGITACVAILVISTIMTL----C--WHFD

G-PW-YIKMG---WY---W-----CM----AKRK--------------------------

------QYEKRPQHKP-----------------------------------------YDA

FLSYSEDDA---PWTKATLLEKL----ETS----------------------------G-

-F-----KVCYHERDFK----------------P--GH-PVLGNIF-YCIENSHKVLFVL

SP------SFV-----------NSC-------WCQY-ELYFAEHRVLN--E-N-QDSLIM

VVLE--D-LPTNSI----------------------------------------------

--PQKFCKLRK-LLKSKTYLKWS----PE-------EHKQK-------LFWHQLTAVL--

--KTV------------NEPMA--------------------------------------

------------------------------------------------------------

-----------------------------------------------------------

>TLR15_Anca

------------------------------------------------------------

------------------------------------------------------------

------------------------------------------------------------

------------------------------------------------------------

------------------------------------------------------------

------------------------------------------------------------

---------------------------MGT--F---------------------------

----------------------------------------------------IH------

-------------------------------------------------------SL--Y

-FCL-I--------------------------------ILSCH-----------------

-----------GESEFQNSEAQIY---------VS----K----------P-------S-

G-------------HHTFN-------Y------S-----------SVTKQQTLQ----QS

L-----------GSLQST----------------------M-KN--VNK--KMTDRSKTL

ESLNPL------Q--------NR-S-SI-----------------------LN---STDY

------QVDELYTQKH---TEIMEVMDSS-------KDQPLLNARMSLE---PRKHNL--

----HEEPVL------------K-------------------------------------

-------------LKAVDHRKH-N-DTP-------------------SPSVAMKPEGCGI

SMNGILDLSNR---NLSEKE-LR----AT---------------IESGPCWAT---LDKI

QV-LNAS-----HN-----------------------------------NLEGDLII---

-LIL--LF---LN----------------------MKNV----RAID----L-----S--

C--NN-----LTF---NAM-------CAE---EIQD-------L--EE--SKLIF----L

NLS----------HNPLKTLSDLC---LP------------QSLKGIDL--SFT-QI-D-

-RI-PQ--EFAILFSN--------------------------------M--EEI-----Y

-----------------LQG---N--Q-------F---V-YTV-----------------

KT-----------------LQS---VLIGDVGT-SSV-----------------------

--------------------------------------------------SYVDLPKH--

-------------------------------SLIES------------------------

-------------------------LPH-RVKH-------LVLSNCSIVEL-PEWF--AQ

KVGQ--LLFLDLSNNP--------M-------------------------NSFP------

----------------GL----PTTLQRLDLSNSN-------------I-----------

-KAMANLKFISNLTVVNIPN-N-K--------IEDISPKHVPYSLEEFDIS-K--NKIRR

MP---FLGAH------------------------------------S-------------

--KLKSLNISGNV--I------MQL-------------NVNTSHP---------------

SLSNL----------DAS-------HNLI----TE--L-----HDE-----MG--T-FLP

----------------------------------------------------------E-

---------------------LKFLNLSGNKISFL-QPGSLP---ESLLELDISNNAITI

IMEE---------T-FG------------RLRN-------------LRVLMAQ-GKHFFC

NCDL--Y-WF----A-NT--------------Y----L---ASP-NV-------------

-------------------QI--------HG-------REALKC-SFP-LQ----KR---

-GLLVENS--NLTILY-C-SLGL-------QMGITAIVAAMFMTVITVL----C--WRFH

G-PW-YIKMG---WY---W-----CM----A-KR--------------------------

-------------KQY--------------------------Q---KSPE-DK--L-YDA

FVSYSENDA---PWTKEILLKNL----EAN----------------------------N-

-Y-----RVCYHERDFL----------------P--GH-PVLGNIF-HCIENSHKVLFVL

SP------SFV-----------NSC-------WCQY-ELYFAEHRVLN--E-N-QDSLIM

IVLE--D-LPPNSV----------------------------------------------

--PQKFSKLRK-LLKRKTYLKWS----SE-------EHKQK-------LFWCQLNAVL--

--KTT------------NE-----------------------------------------

------------------------PM-------VL-----DETIELH-------------

-----------------------------------------------------------

>TLR15_Pybi

------------------------------------------------------------

------------------------------------------------------------

------------------------------------------------------------

------------------------------------------------------------

------------------------------------------------------------

------------------------------------------------------------

-----------MTLPPMPAEKQQGADGLLN--L---------------------------

------------------------------------------SSVL------KW--NVL-

--------------------------------------------GNERHNEKG--TR--F

-PSS-V-------------------------------LQINAS-----------------

------------------CG-------------IP----ANG-TL---------------

D--LLNK-------KLTEN-------E---L--R-----------EKMDPKLCKATM-DK

I-----------KKFNAS-HNN------------------METD--IATLISVFLEMKNV

YLTDVS------Y--------NK---IS-----------------------IN---AKSA

--------KES---HN---LEHSNLLFLN-------LSHNP------LK---T-------

----LQ-TLT---------LPRS-------------------------------------

-------------LKSIDLS-F-T-QINQI-----------------PKEFGAMF-----

FQIEEIFLQGN---HF---I-YK----------------------------------PET

VI----------SN----------------------------------------------

-FNP--VH---------TMR-----------------------KEKH----------S--

---SR---------------------------------------------SAISY-----

-------------------------------------------IDLPEH----T-SI-E-

-SLPTK--ATRQRTSN---------------------SS---------T--EEL-----L

------------E------G--------------F-----QDT-----------------

-------------------MNKCIFLDLSNNLL---------------------------

-------------------------------------------------HKSPYLSNS--

------------------------------------------------------------

------------------------------LRC-------FDLSNSNIQGIANPNL--FP

N-----LTVFKIRNNK--------I----------E--------------NISP------

----------------AY--L-PDSLEECDVSDNK-------------I----RD-----

-FPFQGA--EQSLKSLNLSR-N--------------------------------------

------------------------------------------------------------

--LIMQLNLNTSY--L--------------------------------------------

SLTNL----------DVS-------HNLI----TN--L-------------RYHMGKFLP

----------------------------------------------------------E-

---------------------LKYLNLSENKIFFL-QSGSLP---KSLVELDISNNVILI

IMKE---------M-FH------------HLTN-------------LKILTVQ-GKYFFC

NCEL--Y-WF----V-ST--------------Y----L---ANP-QV-------------

-------------------QI--------NG-------RERLFC-GFP-PK----KR---

-GLLVENS--HLTMLY-C-SLRL-------QIGITASVAILIMSIITLL----C--WYFD

G-PW-YVKMG---WY---W-----CK----A-----------------------------

-------------KRK--------------------QYEKRPE---HKI--------YDA

FLSYSENDA---SWTKEILLEKL----EAN----------------------------G-

-F-----KICYHERDFM----------------P--GH-PVLGNIF-YCIENSHKVLFVL

SP------SFV-----------HSC-------WCQY-ELYFAEHRVLS--E-N-QDSLIM

VVLE--D-LPPNSI----------------------------------------------

--PQKFSKLRK-LLKRKTYLKWS----PE-------EHKQK-------LFWHQLSAVL--

--KTM------------NE-----------------------------------------

------------------------PV-------LI-----TQRKSCTRKHR---------

-----------------------------------------------------------

>TLR15_Prmu

------------------------------------------------------------

------------------------------------------------------------

------------------------------------------------------------

------------------------------------------------------------

------------------------------------------------------------

------------------------------------------------------------

------------------------MGFFIK--T---------------------------

----------------------------------------------------LH--FYL-

-------------------------------------------------------II--L

-FCS-G------------------------------DEQFQHS-----------------

-----------D------NKSLLSKS-------SPSSFPE-S-QI-----P---------

---RRGL-------SFLHN-----------V--R-----------TKTAKKKAG--L-NI

L-----------NILNMR-HNY------SFQVDPTIVLSNI-QP--QHRSSHKFSQTDGI

INDSLT---------------DPSL-RS---------------------MTLS---P-KP

AEKQQEAESLLNLNSV---LKWNVLGDEQYNKKETRFSSSTLQISASCGIPAN---GT--

----FD------------------------------------------------------

-------------LSNRKLT-E-N-ELRGKM----------------DPIFCKAT----L

DQIKKFNASLN---NL-----------ET--D-------------------------IAT

LIPLFLE-----MK---------------------------------------SVYL---

-IDV--SY----NKIFINTKSAKDSYNLQHN-NLLFLNL--SHNPLK----------T--

----------L-----KNL-------------ILPR----------GLKSIDLSF----T

QIN--------------QIPKEFI--AIL------------SQIEEVFL--QGN-PF---

----IY--NAEALMSN--------------------------------F--NPV-----L

------------T----I-----R--N-------Q-----KHF-----------------

------F---NGG------ISY---TNFPQSTIHQIM-----------------------

-------------------------------------------------LHCFTEELP--

-------------------------------EGFQN------------------------

-------------------------TKN--PHI------FLDLSNKSIHNF-------HY

LPKS--LRHFDLSNCN--------L-Q--------G--------------KASP------

----------------NL--F-PN-LTVFKIQNNK-------------I--K--------

-KISPYL--PDSLEECDLSK-N-N--------------------------I-R------N

FP---FHGAE------------------------------------Q-------------

--SLKLLNLSRNL--L----K-----------------QLNVNTS------YL-------

SLNNL----------DVS-------HNLI----TNL-L----GHMG-----TF-----LP

----------------------------------------------------------E-

---------------------LKYLNLSENKIFFL-QPGSLP---KSLVELDISNNAIAI

LMKD---------M-FL------------HLTN-------------LKILTLQ-GEHFFC

NCEL--Y-WF----A--N--------------T----Y---LAN-PQ-------------

-------M-----------QI--------NG-------QERLFC-GFP-KK----KR---

-GLMLQNS--HLTMLY-C-SLGL-------QIGITVVTVILFMSVISVL----C--WHFD

V-PW-YLKMG---WY---W-----CK----A-----------------------------

-------------KRK--------------------QYEKRPE---HKI--------YDA

FISYSENDA---SWTKETLLKNL----EAS----------------------------G-

-F-----KVCYHERDFM----------------P--GH-PVLGNIF-YCIENSHKVLFVL

SP------SFV-----------HSC-------WCQY-ELYFAEHRVLT--E-N-QDSLIM

VVLE--D-LPANSI----------------------------------------------

--PKKFSKLRK-LLQRKTYLKWS----PE-------EHKQK-------LFWHQLSTVL--

--KTM------------NE-----------------------------------------

------------------------PV-------LR-----TQRKSCIRKQTDSTE-----

-----------------------------------------------------------

>TLR15_Thsi

------------------------------------------------------------

------------------------------------------------------------

------------------------------------------------------------

------------------------------------------------------------

------------------------------------------------------------

------------------------------------------------------------

------------------------MGFFIK--T---------------------------

----------------------------------------------------LH--FYL-

-------------------------------------------------------II--L

-FCG-G------------------------------NEQFQHS-----------------

-----------D------NKSLLSKS-------SP----SPS-LL-----P-------E-

SQIPKGL-------NFLHNVTAKSTKKKVRLNIR-----------NILNKQHND--S-FQ

VD----------PTIVLSNSQP--------------------QY--TSS--HKVSQTDEI

INVSLT---------------SQSL-RG---------------------MTLS---PMPP

KKQQKAL-KSV---LK---WNVLGDEQNNAKGTRFPYSTLL------IS-----------

----AGCGIP------------A-------------------------------------

-------------NGTFDLS-N-R-NLTENEL---------------RGKMDPIFCKATL

DQIKKFNASFN---KL---E-ID----------------------------------IAI

LIPLFLE-----MK---------------------------------------SVYL---

-IDV--SY----NKIFINTTSAKENHNLEHN-NLLYLNL--SHNPLK----------T--

----------L-----TNL-------------TLPR----------GLKSIDLSF----T

QIN--------------QIPKEFV--AMF------------SQIEEVFL--QGN-QF---

-------------TYN---------------------TE---------V--VTS------

------------N---------------------F-----NPV-----------------

HK-----------------IKNDEVFFHRSPHI-ERL-----------------------

-------------------------------------------------LEEETHQIK--

-------------------------------LNCFT-------------------EVFPE

G------------------------FPN--------------ITKEHIVLHFRINFMHYF

HYLPKSLRRVDLSNCN--------L-------------------------QGIA------

--------------SPNL--F-PN-LTVFKIQNNK-------------M-----------

-KKISYL--PDSLEECDVSK-N-K--------------------------L-Q--TFP--

-------FNR---------------TEQ--------------------------------

--SLKLLNLSRNH--L----K-----------------ELNVSTS------YL-------

FLNNL----------DVS-------YNLI----TKL-L----GDMA---------K-CTP

----------------------------------------------------------E-

---------------------LKYLNLSENKICFL-QPGIFP---KSLVELDISNNAIAI

LMKE---------T-FL------------PLTN-------------LKILTVQ-GKHYFC

NCEL--Y-WF----ANTY--------------FANPQI----------------------

-------------------QI--------NG-------QESFFC-GFP-RK----MR---

-GLLLQNN--QLTMLR-C-SLGL-------QIGITVIVVILIMSIISIL----C--WQFD

G-PW-HLKMG---WY---W-----CK----A-----------------------------

-------------KRK--------------------QYEKRPE---HKI--------YDA

FISYSENDA---SWTKETLLKNL----EAR----------------------------G-

-F-----KVCYHERDFM----------------P--GH-PVLGNIF-YCIENSHKVLFVL

SP------RFV-----------HSC-------WCQY-ELYFAEHRVLT--E-K-QDSLIM

VILE--D-LPANSI----------------------------------------------

--PKKFSKLRK-LLKRKTYLKWS----PE-------EHKQK-------LFWHQLTAVL--

--KTT------------ND-----------------------------------------

------------------------PV-------LK-----TQRKSCIRKQTDET------

-----------------------------------------------------------

>TLR15_Opha

------------------------------------------------------------

------------------------------------------------------------

------------------------------------------------------------

------------------------------------------------------------

------------------------------------------------------------

------------------------------------------------------------

-----------MTLSPMPAEKQQKTGGLLN------------------------------

---------------------------------------LNSVLKW------NV------

--------------------------------------------LGDEQYKVKGTRF---

-PSSTL------------------------------HFSAGCG-----------------

---------------------------------IP----ANG-TF---------------

D--LSNR-------KLTKN-------E---L--R-----------GKIDPIFCKATL-DQ

I-----------KKFNAS-LNK------------------LETD--IATLIPLFLEMKNV

YLIDVS------Y--------NKIF--------------------------INTKSARES

------HNLEH---NN---LQFLNLS-HN-----P-LKTLT-----------S-------

----LTLPRG--------------------------------------------------

-------------LKSIDLS-F-T-QISQI-----------------PKEFVTMF-----

SHIEEVFLQGN---QF---------------K-------------------------YNT

EA-VAFN-----FN----------------------------------------------

-----------------PVRRIRNDESVFNG----AISF----TKLP----------Q--

S--TP-----------IES-------------LLEK----------AT--HQIML-----

-----------------------------------------------------N-CF-T-

-ET---------EFQN---------------------IT---------------------

------------K---------------------------KHI-----------------

------S------------------LDLSNKFM---------------------------

-------------------------------------------------HNFHYLPKS--

------------------------------------------------------------

------------------------------LHR-------VDLSNCNLQGIARPNL--FP

N-----VTMLKIQNNK--------I----------K--------------KIYL------

----------------------PDSLEECDVSRNK----------------------ILR

-FTFHRE--GQNLKVLNLSR-N--------------------------------------

------------------------------------------------------------

--LLKQLNVNNSY--L--------------------------------------------

SLYNL----------DVS-------YNLI----TD--L-----LGD-----MG--T-LMP

----------------------------------------------------------E-

---------------------LKYLNLSENKIFFL-QPGFFP---KSLVELDISNNAIAI

LMKE---------T-FV------------HLTN-------------LKILTVQ-GKHFFC

NCEL--Y-WFANTYLANP---------------------------QM-------------

-------------------QI--------NG-------RESLFC-GFP-KK----KR---

-GLLLQNN--SLTMLH-C-CLGL-------QIGITVIVVILIMSIVSVL----C--WHFD

G-PW-YLKMG---WY---W-----CK----A--K--------------------------

-------------RKQ--------------------------Y---EKRPEHK--I-YDA

FISYSENDA---SWTKETLLKNL----EGR----------------------------G-

-F-----KVCYHERDFM----------------P--GH-PVLGNIF-YCIENSHKVLFVL

SP------RFV-----------HSC-------WCQY-ELYFAEHRVLT--E-N-QDSLIM

VVLE--D-LPTNSI----------------------------------------------

--PKKFSKLRK-LLKRKTYLKWS----PE-------EHKQK-------LFWHQLTAVL--

--KTM------------NEPVLRTQRKSCIRKQTDE------------------------

---------------------------------TE-------------------------

-----------------------------------------------------------

>TLR14_Orrh

------------------------------------------------------------

------------------------------------------------------------

------------------------------------------------------------

------------------------------------------------------------

------------------------------------------------------------

------------------------------------------------------------

------------------------M--FPP--H---------------------------

--------------------------------G---S--C--L--W------IC----F-

---LI---VN-------------------------------------LAEAKC--II---

--NL-S------------------------------TKTADCH-----------------

-----------G------KE--FL-E-------VP----D---DL-----P-------N-

D--LQIL-------DLSYN-------G---L--T-----------RIKTGDFIF--F-SD

L-----------RELNLS-YNN------------------I-SS--IDN--DSFVSNTLL

GYLSLF------N--------NS-L-TE-----------------------MP---S-QV

------L-EPL---MY---LEYLDMS-NN-----F-YNSST------LE---D---V---

----FK-TFV------------H-------------------------------------

-------------LKYLSIG-G-P-MIKKIL----------------KDDFVPIK----D

LDLTRFSLKTQS--SL--EE-YE----VG--A------------YNV----------VNT

TY-LWFD-----IA--------LDGNPSN-------L----------------PLIL---

-NDL--KG----K----SFK---------------SIRF----RNLF----E-----F--

T--YY---T-K-D---VDL-------FT----GLKE-------I--YV--DELIF----Y

RGK----------------FNEYL---LQ-L----------I-LNSVQQ----S-NI-S-

-DL---------SLLA---------------------VD---------F--ARS-----P

------------N----S-----N--N-------T--DD-D------------S-----D

GL----S------------LQN---LVIKDVTN-PDI-----------------------

-------------------------------------------------LRF---DWT--

-------------------------------FTWFS------------------------

---------------------------N--IVN-------LHIINVNFNFV-PCDA--WL

HMRK--VETLNISENR--------L-Q--------D--------------SFLH--NP--

-----KCQ--------TG--L-PT-IEMFNASRNL-------------L--C-SL-GMIS

-GLTTQW--PK-LSHLDLSY-N-K--------------------------I-G---T---

--------RK------------E--SCT-W----------I-----A-------------

--NITHLYLHHNV--L----T-VD--------------ISQCLPQ---------------

TLEYL----------DMS-------HSQL----ER--L-----NLT-----YF--N-LAT

----------------------------------------------------------N-

---------------------LRELYLHHNKIKFI-PAGW--ES-FRLEFLTLQSNSFGI

IDRG---------S-FR------------DLPQ-------------LRELSAG-NNPYYC

NCEL--Y-AF----F--K--------------E----A-------IP----------NKK

-------L-----------LI--------AD-W-----PDSYYC-YHP-LD----LL---

-DTKIEYF--NPGQLQ-C-DVGL-----VVAIT--VSVTAMIVIICMML----C--WRFD

V-PW-YLRAT---CN---I-----MH----S--K------------------Y------R

S-K---N----S------------------------AD----N---REF---I----YHA

FISYSYLDA---DWVRGVLLPRL----ENG---T--------------PP----------

---Y---RLCIHERDFL----------------P--GR-WIIDNII-ENIENSRKVIFVL

SR------SFV-----------NSE-------WCNY-ELYFAHQRNIG--H-A-FNDVIL

VVKE--N-VTLEDL----------------------------------------------

--PKRFCKLRK-MLKTKTYLEWP----SE-------QNRQP-------FFWVQLKTIL--

--GRA------------SQ-----------------------------------------

-------------------T-ING-Q-------DN-----FSVISESP------VDRLSD

SSVVSSTTN----------------------QTHTFPAS--------------------

>TLR14_Crfi

------------------------------------------------------------

------------------------------------------------------------

------------------------------------------------------------

------------------------------------------------------------

------------------------------------------------------------

------------------------------------------------------------

------------------------M--FYY--S---------------------------

--------------------------------G---R--L--A--L------PL----I-

---LL---FGVV-----------------K-----------------LCKSVC--NK---

--SP-D------------------------------GKHVNCK-----------------

-----------G------AK--LG-H-------VP----Q---DL-----S-------K-

Y--LEYL-------DLSYN-------N---I--R-----------ELNPSMFFR--Y-RY

L-----------DTLNLS-YNN------------------I-SR--IEN--GSFKNNLLL

RNLSLF------N--------NS-L-TD-----------------------IP---V-SS

------L-EPL---AR---LQILDLS-NN-----F-YRFIT------LG---K---G---

----FS-KLM------------N-------------------------------------

-------------LQYLSIG-G-P-IVSSVL----------------RHDFVALK----N

ISLQKFALKTKS--GL--DY-YE----PG--A------------FSV----------LNT

EA-LWLD-----IA--------LDSNAIL-------L----------------FDML---

-KDL--KG----K----KLT---------------TLRF----RNLF----E-----F--

S--YY---E-L-V---LDI-------FF----FLPA-------I--NV--KNLVF----F

RGK----------------FNENL---LR-L----------V-LQNVQN----S-NI-K-

-NL---------LLLS---------------------VD---------F--ARS-----L

------------N----A-----N--K-------T--DA-I------------I-----N

NL----A------------LQN---LVIKDVNN-PDI-----------------------

-------------------------------------------------LRF---DWT--

-------------------------------FTWFR------------------------

---------------------------K--VTQ-------LSIINVNFNFV-PCDA--WA

EMTN--VVTLNISGNR--------L-I--------S--------------SYLS--DI--

-----LCKP-------NV--L-PN-VEMFNVSNNQ-------------I--D-SL-KTIS

-LLTADW--PN-LTQVDLTN-N-V--------------------------I-G---S---

--------HN------------E--SCN-W----------T-----T-------------

--SIKILILRYNI--L----T-QN--------------VFWCLPI---------------

TLEYL----------DMS-------NCQL----ER--L-----DMS-----YF--D-QAT

----------------------------------------------------------N-

---------------------LTELILRNNKIKFI-PTGW--QN-PNLQVLSLERNSFGV

IDQG---------S-FQ------------DLLQ-------------LRMLTAG-NNPYHC

TCDL--Y-GF----F--T--------------E----T-------LT----------DES

-------L-----------SL--------PD-W-----PDKYYC-HYP-LD----LL---

-DTKVEFF--SPGKLE-C-DIRL-----VVAIS--VSVTAVVVIVCMLL----C--YRFD

V-PW-YLRAT---CQ---I-----IQ----S--K------------------Y------R

S-K---K----S------------------------RE----T---KEY---A----YHA

FISYSYSDA---DWVRGVLLPHL----EDS---N--------------SS----------

---F---RVCIHERDFL----------------P--GK-WIIDNII-DNIENSRKIIFVL

SH------NFV-----------NSE-------WCNY-ELYFAHQRAVG--H-A-FEDIIL

LVKE--N-VRMEDL----------------------------------------------

--PKRFCKLRK-LLSTKTYLEWP----LE-------ETRQP-------FFWVQLKSIL--

--GKG------------SK-----------------------------------------

-------------------S-LLG-H-------DN-----IS-----V------NVAVDS

SSEGFIPSK----------------------CNQDLPSS--------------------

>TLR14_Drci

------------------------------------------------------------

------------------------------------------------------------

------------------------------------------------------------

------------------------------------------------------------

------------------------------------------------------------

------------------------------------------------------------

------------------------M--FHL--N---------------------------

--------------------------------G---R--F--A--L------LL----V-

---VL---CGVV-----------------K-----------------LCESAC--VI---

--QL-D------------------------------GRYVNCK-----------------

-----------G------EN--LF-S-------VP----Q---NL-----S-------E-

D--LEYL-------DLSYN-------K---I--T-----------EVDVTSFIR--Y-PY

L-----------HTLNLS-YNN------------------I-SH--IEN--GSFEVNLLL

RNLSLF------N--------NS-L-TE-----------------------IP---A-S-

------L-APL---VS---LKILDLS-NN-----F-YRFPT------LG---E---E---

----FN-KLV------------E-------------------------------------

-------------LQDLSIG-G-P-LVSVVQ----------------RNDLAVLQ----S

IHLQKFSLKSKS--SL--DY-YE----PG--A------------FSV----------LDT

EV-LWLD-----VA--------LDSNPDL-------L----------------TDIL---

-NDL--NG----K----NLT---------------TLRF----RNLF----E-----F--

S--YY---D-Q-A---KDI-------FS----LLPA-------I--NL--RDLVF----Y

RGK----------------FNENL---LR-L----------V-LQNIQV----S-KI-E-

-NL---------LLLS---------------------ID---------F--ARS-----V

------------N----T-----N--K-------T--DV-T------------I-----D

NL----D------------LQN---LVIQDVTN-PDI-----------------------

-------------------------------------------------LRF---DWT--

-------------------------------FTWFS------------------------

---------------------------K--VAH-------LSIINVNFNFV-PCDA--WS

QMTN--VVSLNISSNR--------L-R--------A--------------AYLF--NL--

-----LCKD-------NG--L-PN-IETFNVSNNE-------------V--R-SL-RTIS

-LLTAYW--PK-LTQLDLSD-N-A--------------------------I-G---S---

--------MA------------E--SCE-W----------R-----A-------------

--NIKTLILRNNI--L----L-ED--------------MFHCLPT---------------

TVEHL----------DMS-------NSQL----ER--L-----DMN-----YF--D-KAT

----------------------------------------------------------N-

---------------------LTELIVRNNKIKFI-PTDW--SS-PNLQVLALEGNSFGV

IDQG---------S-FQ------------NLRQ-------------LRELTAG-NNPYHC

TCDL--Y-AF----I--T--------------V----T-------LN----------EGS

-------L-----------SL--------PD-W-----PDNYYC-YHP-HQ----LL---

-DTRIEFF--TPGRLE-C-DLRL-----VVALS--VSVTAVVVFVCMLL----C--YKFD

V-PW-YLRAT---CQ---I-----IR----S--K------------------Y------R

S-R---K----D------------------------QE----A---REY---A----YHA

FISYSYSDA---DWVRGMLLPHL----ESA---T--------------PP----------

---Y---RICIHERDFL----------------P--GK-WIIDNII-ENIENSRKIIFVL

SR------NFV-----------NSE-------WCNY-ELYFAHQRAIG--H-A-FEDVIL

VVKE--N-VCMQDL----------------------------------------------

--PKRFCRLRK-MLSTKTYLEWP----VE-------ENRQP-------FFWVQLKSIL--

--GKG------------SI-----------------------------------------

-------------------R-SSG-H-------DN-----TSLVNEMV------NVTVES

SSEEPMTMTIK--------------------DNLKLPDS--------------------

>TLR14_Oopu

------------------------------------------------------------

------------------------------------------------------------

------------------------------------------------------------

------------------------------------------------------------

------------------------------------------------------------

------------------------------------------------------------

------------------------M--FLQ--N---------------------------

--------------------------------G---R--L--L--V------LF----L-

---FL---FGAI-----------------G-----------------LCESIC--EI---

--TL-S------------------------------GKHVNCK-----------------

-----------G------EK--LD-Y-------IP----Q---DL-----S-------E-

E--LEYL-------DFSYN-------I---L--T-----------HLVPGSLSR--Y-PY

L-----------HTLNLS-FNN------------------I-SR--IEN--GSFEVNFLL

RNLSLF------N--------NS-F-ME-----------------------IP---A-SS

------L-ESL---KG---LQILDVS-NN-----F-YRFPT------LS---K---E---

----FT-TLV------------N-------------------------------------

-------------LQELSIG-G-P-LVSSIL----------------RDDFRVLQ----N

ISLQKFSLKTKS--SL--EN-YE----PG--A------------FSE----------LNT

EV-LWLD-----IA--------LDGNSSL-------L----------------PYML---

-LDL--AQ----K----NLI---------------TLRF----RNLF----E-----F--

S--YY---E-G-T---LDI-------FS----FLPA-------I--GV--RDLVF----Y

RGK----------------FNENL---LR-Q----------V-LQNVQI----S-NI-E-

-NL---------LLLS---------------------ID---------F--ARS-----L

------------N----A-----N--K-------S--DG-G------------I-----T

NL----T------------LQN---LIIQDVTN-PDI-----------------------

-------------------------------------------------LRF---DWT--

-------------------------------FTWFS------------------------

---------------------------Q--VIH-------LSIINVNFNFV-PCDA--WG

EMAN--VMTLNISGNR--------L-F--------A--------------SYLF--NS--

-----LCTL-------NI--L-PN-IHTFNISNNQ-------------I--H-SL-KTIS

-RLTANW--PK-LTTVDMAN-N-F--------------------------I-G---S---

--------LN------------E--SCV-W----------Q-----N-------------

--NIKTLILTNNL--L----T-VD--------------VFQCLPI---------------

TLEYL----------DMS-------NSQL----DR--L-----HMK-----YF--L-QAT

----------------------------------------------------------N-

---------------------LTELILRNNKIKFI-PTGW--QS-PNLQVLSIEGNSFGV

IDQG---------S-FE------------NLNQ-------------LRSLTAG-NNPYHC

TCDL--H-AF----I--T--------------E----T-------LN----------EGS

-------L-----------TL--------SD-W-----PENYYC-YHP-LN----LL---

-DTRIEFF--SPGKLE-C-DVRL-----VVAAS--VSVTTFIIIICMLL----C--YRFD

V-PW-YLKAT---WQ---V-----IR----S--K------------------Y------R

S-K---K----D------------------------QE----T---REY---A----YHA

FISYSYSDA---DWVRGVLLPRL----ENS---N--------------PP----------

---Y---RVCIHERDFL----------------P--GR-WIIDNII-ENIENSCKIIFVL

SH------NFV-----------NSE-------WCNY-ELYFAHQRAIG--H-A-FEDVIL

VVKE--N-VSMEDL----------------------------------------------

--PKRFCRLRK-MLSTKTYLEWP----LE-------ENRQP-------FFWVQLKSIL--

--GKG------------SQ-----------------------------------------

-------------------S-ISG-Q-------EN-----ISLVTENI------TVVVDG

SSEESLTMTTK--------------------CNLNLPGS--------------------

>TLR14_Atgl

------------------------------------------------------------

------------------------------------------------------------

------------------------------------------------------------

------------------------------------------------------------

------------------------------------------------------------

------------------------------------------------------------

------------------------M--CHR--N---------------------------

--------------------------------V---R--L--V--L------YF----I-

---LL---FRVV-----------------K-----------------LCESVC--DI---

--SP-D------------------------------EKYVYCK-----------------

-----------G------EY--LN-Y-------VP----Q---DL-----S-------E-

D--LEYL-------DLSYN-------Q---I--T-----------ELNRGSFSR--Y-PY

L-----------DTLNLS-FNN------------------I-SH--IEN--GLFEFNLLL

RNLSLF------N--------NS-L-VE-----------------------IP---A-SS

------L-EHL---EG---LQVLDLS-NN-----F-YRSST------LG---E---E---

----FY-KLV------------N-------------------------------------

-------------LRVLSIG-G-P-LVSSVL----------------KDDFVVLQ----N

ISLQKFALKTKS--SL--NY-YE----PG--A------------FTV----------LNT

EV-LWMD-----IA--------LDSNANL-------L----------------IDML---

-KDL--AG----K----KLT---------------TLRF----RNLF----E-----F--

S--YY---E-H-V---LDI-------FS----FLPA-------I--HL--RDLIF----Y

RGK----------------FNENL---LR-L----------V-LQNVQI----S-NI-E-

-NL---------LLLS---------------------VD---------F--ARS-----L

------------S----A-----N--K-------T--DV-T------------I-----N

DL----G------------LQN---LVIQDVTN-PDI-----------------------

-------------------------------------------------LRF---DWT--

-------------------------------FTWFS------------------------

---------------------------K--VNH-------LSIINVNFNFV-PCDA--WG

QMTN--VVLLNISNNR--------L-F--------A--------------TYLY--NL--

-----LCKQ-------NV--L-PN-IETFNISNNR-------------I--D-SL-RTIS

-LLTAKW--PK-LTLLDLAS-N-A--------------------------I-G---S---

--------IK------------E--FCV-W----------N-----I-------------

--SIKILILKDNM--L----T-VD--------------VFQCLPV---------------

TLKYL----------DMS-------NSQL----ER--L-----DMS-----YF--D-QAT

----------------------------------------------------------N-

---------------------LTQLILRNNKIKFI-PTGW--YS-PNLQVLALEGNSFGV

IDQG---------S-FQ------------NLVQ-------------LRELTAG-NNPYHC

TCDL--N-AF----I--T--------------D----T-------LQ----------AGS

-------L-----------SL--------PD-W-----PDNYYC-YHP-PY----ML---

-DTRIESF--SPGKLE-C-DVRL-----VVAVS--VSVTAFVLIIGMLF----C--FKFD

I-PW-YLRAM---CQ---I-----IQ----S--K------------------Y------R

S-K---N----S------------------------EE----T---REY---A----FHA

FISYSYSDA---DWVRGVLLPRL----ENS---N--------------PP----------

---Y---RVCIHERDFL----------------P--GR-WIIDNII-ENIENSWKIIFVL

SH------NFV-----------NSE-------WCNY-ELYFAHQRAIG--H-A-FEDVIL

VVKE--N-ISMEDL----------------------------------------------

--PKRFCRLRK-LLSTKTYLEWP----LE-------ENRQP-------FFWVQLKSIL--

--GKG------------GK-----------------------------------------

-------------------S-LLG-L-------DN-----MSLMNETI------NVGVDS

NSEEPITMTTK--------------------SNLNLTPRS-------------------

>TLR14_Atze

------------------------------------------------------------

------------------------------------------------------------

------------------------------------------------------------

------------------------------------------------------------

------------------------------------------------------------

------------------------------------------------------------

---------------MCH------R--NVR--L---------VL----------------

-------------------------------YF---ILLF--R--V------VK----L-

----------------------------------------------------C--ES---

-VCD-I---------------------------SPDEKYVYCK-----------------

-----------G------EY--LN-Y-------VP----Q-D--L-----S-------E-

D--LEYL-------DLSYN-------Q---I--T-----------ELNRGSFSR--Y-PY

L-----------DTLNLS-FNN------------------I-SH--IEN--GLFEFNLLL

RNLSLF------N--------NS-L-VE-----------------------IP---A-SS

------L-EHL---EG---LQVLDLS-NN-----F-YRSST------LG---E---E---

----FY-KLV------------N-------------------------------------

-------------LRVLSIG-G-P-LVSSVL----------------KDDFVVLQ----N

ISLQKFALKTKS--SL--NY-YE----PG-----------------A---FTV----LNT

EV-LWMD-----IA--------LDSNANLL-----------------------IDML---

-KDL--AG----K----KLT---------------TLRF----RNLF----E-----F--

S--YY---E-H-V---LDI--------FS---FLPA-------I--HL--RDLIF----Y

RGK----------------FNENL---LR-L----------V-LQNVQI----S-NI-E-

-NL---------------------------------------------L--LLS-----V

------------D----F-A---R--S-----------L-SAN------KTDVT-----I

ND----L---G--------LQN---LVIQDVTN---------------------------

-------------------------------------------------PDILRFDWT--

-------------------------------FTWFS------------------------

---------------------------K--VNH-------LSIINVNFNFV-PCDA--WG

QMTN--VVLLNISNNR--------L-F-A------T--------------YLYN--LLCK

Q---------------NV--L-PN-IETFNISNNR-------------I--D-SL-RTIS

-LLTAKW--PK-LTLLDLAS-N-A--------------------------I-G---SI--

--------------------K-E--FCV-W----------N-----I-------------

--SIKILILKDNMLTV----D-----------------VFQCLPV---------------

TLKYL----------DMS-------NSQL----ER--L-----DMS-----YF--D-QAT

----------------------------------------------------------N-

---------------------LTQLILRNNKIKFI-PTGWYS---PNLQVLALEGNSFGV

IDQG---------S-FQ------------NLVQ-------------LRELTAG-NNPYHC

TCDL--N-AF----I--T--------------D----T-------LQ-------------

-------A-GSL-------SL--------PD-W-----PDNYYC-YHP-PY----ML---

-DTRIESF--SPGKLE-C-DVRL-----VVAVS--VSVTAFVLIIGMLF----C--FKFD

I-PW-YLRAM---CQ---I-----IQ----S--K--------------------------

------Y----R-SKN---------------S----EE----T---REY---A----FHA

FISYSYSDA---DWVRGVLLPRL----ENS---N--------------PP----------

-Y-----RVCIHERDFL----------------P--GR-WIIDNII-ENIENSWKIIFVL

SH------NFV-----------NSE-------WCNY-ELYFAHQRAIG----HAFEDVIL

VVKE--N-ISMEDL----------------------------------------------

--PKRFCRLRK-LLSTKTYLEWP----LE-------ENRQP-------FFWVQLKSIL--

--GKG------------GK-----S-------LLGL------------------------

-------------------DNMSLMN-------ET-----INVGVDSNSEEPITMTTKSN

LNLTPRS----------------------------------------------------

>TLR14_Rhma

------------------------------------------------------------

------------------------------------------------------------

------------------------------------------------------------

------------------------------------------------------------

------------------------------------------------------------

------------------------------------------------------------

------------------------M--CHR--S---------------------------

--------------------------------V---Y--L--V--L------RL----I-

---SF---FGVV-----------------K-----------------LCESVC--DT---

--SP-D------------------------------KKYVHCN-----------------

-----------G------LY--LN-D-------VP----Q---NL-----S-------E-

D--VEYL-------DLSYN-------K---I--T-----------ELKRGSFSR--Y-PY

L-----------DTLNLS-FNN------------------I-SY--IEN--GSFKTNLLL

RNLSLF------N--------NS-L-VE-----------------------IP---A-SS

------L-EHL---EG---LQILELS-NN-----F-YKFST------LG---K---E---

----FY-KLV------------N-------------------------------------

-------------LQVLSIG-G-P-LVSTVL----------------KDDFAVIQ----N

ISLQKFALKTKS--SL--DY-YE----QG--A------------FSV----------LNT

EV-LWLD-----IA--------LDSNASL-------L----------------NYML---

-RDL--AG----K----NLT---------------TLRF----RNLF----E-----F--

S--YY---E-E-V---LDI-------FF----FLPA-------I--NT--RDLIF----F

RGK----------------FNENL---LR-L----------V-LQNVQS----S-KI-Q-

-NL---------LLLS---------------------VD---------F--ARS-----V

------------S----D-----Q--T-------S--DE-A------------I-----K

DL----V------------LQN---LVIQDVTN-PDI-----------------------

-------------------------------------------------LRF---DWS--

-------------------------------FTWFR------------------------

---------------------------K--VAH-------LSIINVNFNFV-PCDA--WD

EMAN--VVSLNISNNR--------L-F--------S--------------TYLY--NL--

-----LCKQ-------NV--L-PN-IETFNVSLNR-------------I--D-SL-RTIS

-LLTAKW--PK-LTQLDLSS-N-A--------------------------I-G---S---

--------IK------------E--SCV-W----------N-----I-------------

--GIKILILQNNK--L----T-VD--------------VFPCLPV---------------

TLEYL----------DMS-------NSRL----ER--L-----DMS-----YF--D-QAT

----------------------------------------------------------N-

---------------------LTKLILRNNKIKFI-PTGW--NS-PNLQVLELEGNAFGV

IDQG---------S-FQ------------KLIR-------------LRELTAG-NNPYHC

TCDL--Y-AF----I--T--------------D----T-------LK----------EGS

-------L-----------LL--------PD-W-----PDNYYC-YHP-TY----ML---

-DTRIESF--SPGKLE-C-DVRL-----VVAVS--VSVTALVLIIGMLL----C--FKFD

I-PW-YLRAT---CQ---I-----IQ----S--K------------------Y------R

S-R---N----S------------------------QE----T---REY---A----FHA

FISYSYSDA---DWVRGVLLHHL----ENS---N--------------PP----------

---Y---RVCIHERDFL----------------P--GR-WIIDNII-ENIENSRKIIFVL

SH------NFV-----------NSE-------WCNY-ELYFAHQRAVG--H-S-FGDVIL

VVKE--N-IRMEDL----------------------------------------------

--PKRFCRLRK-LLSTKTYLEWP----FE-------ENRQP-------FFWIQLKSIL--

--GKG------------SK-----------------------------------------

-------------------S-LSG-P-------DN-----I-------------------

-----------------------------------------------------------

>TLR14_Buga

------------------------------------------------------------

------------------------------------------------------------

------------------------------------------------------------

------------------------------------------------------------

------------------------------------------------------------

------------------------------------------------------------

------------------------M--CHH--I---------------------------

--------------------------------V---Y--L--V--L------HL----I-

---LF---FGVV-----------------K-----------------LCESVC--DI---

--SP-D------------------------------NKYVYCK-----------------

-----------G------EY--LK-Y-------VP----Q---NL-----S-------E-

D--VEYL-------DLSYN-------K---I--T-----------ELNRGSFSR--Y-PY

L-----------NTLNLS-FNN------------------I-SH--IDN--GSFEANLLL

KNLSLF------N--------NS-L-VE-----------------------IP---A-SS

------L-EHL---KS---LQILDLS-NN-----F-YRFST------LG---K---E---

----FY-KLV------------N-------------------------------------

-------------LQVLSIG-G-P-LISRVL----------------KDDFAVIQ----N

ISLHKFALKTKS--SL--DY-YE----QG--A------------FSV----------LNT

EV-FWLD-----IA--------LDSNASM-------L----------------NDML---

-KDL--AG----K----KLT---------------TLRF----RNLF----E-----F--

S--YY---E-H-V---LDI-------FS----FLPA-------I--DI--RDLIF----F

RGK----------------FNENL---LR-L----------V-LKNVQS----S-KI-K-

-NL---------LLLS---------------------VD---------F--ARS-----V

------------R----D-----N--T-------L--NT-T------------I-----N

DL----V------------LQN---LVIQDVTN-PDI-----------------------

-------------------------------------------------LRF---DWT--

-------------------------------FTWFS------------------------

---------------------------K--VTY-------LSIINVNFNFV-PCDA--WG

QMAN--VVSLNISNNR--------L-F--------A--------------AYLY--NL--

-----QCKQ-------NV--L-PN-IETFNVSHNQ-------------I--D-SL-RTIS

-LLTAKW--PK-LTQLDLAN-N-A--------------------------I-G---S---

--------IK------------E--SCV-W----------N-----I-------------

--SIKILILRNNM--L----T-VD--------------VFQCLPV---------------

SLEYL----------DMS-------NSHL----ER--L-----DIS-----YF--D-QAT

----------------------------------------------------------N-

---------------------LTKLILRNNKIMFI-PTGW--NS-PNLQVLALEGNSFGV

IDQG---------S-FQ------------NLVR-------------LRELTAG-NNPYHC

TCDL--N-AF----I--T--------------D----T-------LK----------EGS

-------L-----------SL--------PD-W-----PEKYYC-YHP-IY----ML---

-DTRIESF--SPGRLE-C-DVRL-----VVAVS--VSVTAFVLIIGMLL----C--LKFD

I-PW-YLRAT---CQ---I-----IQ----S--K------------------Y------R

S-R---N----S------------------------QE----T---REY---A----FNA

FISYSYSDA---DWVRRVLLSHL----ENS---N--------------PP----------

---Y---RVCIHERDFL----------------P--GR-WIIDNII-ENIENSQKIIFVL

SH------NFV-----------NSE-------WCNY-ELYFAHQRAIG--H-S-FGDVIL

VVKE--N-MKMEDL----------------------------------------------

--PKRFCRLRK-LLSTKTYLEWP----LE-------ENRQP-------FFWFQLKSIL--

--GKG------------SK-----------------------------------------

-------------------S-LSV-L-------DN-----MSMINETT------DVGVDR

NSE--------------------------------------------------------

>TLR14_Rhsp

------------------------------------------------------------

------------------------------------------------------------

------------------------------------------------------------

------------------------------------------------------------

------------------------------------------------------------

------------------------------------------------------------

---------------MCH------R--NVY--L---------VL----------------

-------------------------------HL---ISFF--R--V------VK----L-

----------------------------------------------------C--ES---

-VCD-I---------------------------SPDKKYVYCK-----------------

-----------G------EY--LK-Y-------VP----Q-N--L-----S-------E-

D--VEYL-------DLSYN-------K---I--T-----------ELNRSSFSR--Y-PY

L-----------DTLNLS-FNN------------------I-SR--IEN--GSFEANLLL

RNLSLF------N--------NS-L-VE-----------------------IP---A-SS

------L-EHL---EG---LQILELS-NN---FYR-FSTLG------KE-----------

----FY-KLL------------N-------------------------------------

-------------LQVLSIG-G-P-LISRVL----------------KDDFAVIQ----N

ISLQKFALKTKS--SL--DY-YE----QG-----------------A---FSL----LNT

EV-LWLD-----IA--------LDSNASLL-----------------------NDML---

-KDL--VG----K----NLT---------------TLRF----RNLF----E--------

---FS---Y-YEH---VLD-------IFS---FLPA-------I--DI--RDLIF----F

RGK----------------FNENL---L-------------R-LVLKNV--QSS-KL-K-

-NL-LL--LSVDFARS---------------------VS---D---------NT-----L

------------D---------------------E--TI-NDL-----------------

------V------------LQN---LVIQDVTN---------------------------

-------------------------------------------------PDILRFDWT--

-------------------------------FTWFS------------------------

---------------------------K--VTY-------LSIINVNFNFV-PCDA--WG

QMAN--VVSLNISNNR--------L-F-S------T--------------YLYN--LLCK

Q---------------NV--L-PN-IKTFNVSHNR-------------I--D-SL-RTIS

-LLTAKW--PK-LTQLDLAS-N-A--------------------------I-G-------

--------SI----------K-E--SCV-W----------N-----I-------------

--SIKILILRNNM--L--TVD-----------------VFQCLPV---------------

TLEYL----------DMS-------DSHL----ER--L-----DMR-----YF--D-QAR

----------------------------------------------------------N-

---------------------LTKLILRNNKIKFI-PTGWNS---PNLQVLALEGNSFGV

IDQG---------S-FQ------------NLVR-------------LRELTAG-NNPYHC

TCDL--NAFI----T--D--------------T----L-------KE-------------

-------G-----------SL----SL--PD-W-----PDNYYC-YHP-TY----ML---

-DTRIESF--SPGKLE-C-DVRL-----VVAVS--VSVTAFVLIIGMLL----C--FKFN

I-PW-YLRAT---CQ---I-----IQ----S--K--------------------------

------Y----R-ARN---------------S----QE----T---REY---A----FHA

FISYSYSDA---DWVRGVLLSHL----ENS---N--------------PP----------

-Y-----RVCIHERDFL----------------P--GR-WIIDNII-ENIENSRKIIFVL

SH------NFV-----------NSE-------WCNY-ELYFAHQRAVG--H-S-FGDVIL

VVKE--N-IKMEDL----------------------------------------------

--PKRFCRLRK-LLSTKTYLEWP----LE-------ENRQP-------FFWFQLKSIL--

--GKG------------SR-----S-------LLGLDNM---------------------

------------------------------------------------------------

-----------------------------------------------------------

>TLR14_Live

------------------------------------------------------------

------------------------------------------------------------

------------------------------------------------------------

------------------------------------------------------------

------------------------------------------------------------

------------------------------------------------------------

------------------------M--FRC--D---------------------------

--------------------------------G---P--L--V--L------LL----L-

---LL---SAIV-----------------E-----------------LCESEC--NI---

--GS-N-G----------------------------NS-VYCK-----------------

-----------G------EN--LD-S-------VP----Q---NL-----S-------K-

D--LEYL-------DLSYN-------K---I--T-----------ELTQGSFSR--Y-PG

L-----------HTLDLR-FNN------------------I-SH--IEN--GTFRVNLLL

RNLSLF------N--------NS-L-KE-----------------------IP---A-SA

------L-EPL---KD---LQILDLS-NN-----L-YRFAT------LG---R---E---

----FN-KLV------------K-------------------------------------

-------------LQDLSIG-G-P-LVSRVL----------------RDDFAVLQ----N

ISLQKFSLKTKS--SL--GS-YE----SG--A------------FSV----------LDT

EV-LWLD-----IA--------LDNNAHL-------L----------------NHML---

-KDL--KG----K----KLT---------------TLRF----RNLF----E-----L--

S--YY---D-H-V---LDI-------FS----YLPA-------F--EL--KNLVF----Y

RGK----------------FNENL---LR-L----------V-LQNAQI----S-KI-E-

-NL---------LLLS---------------------VD---------F--ARS-----S

------------N----A-----N--K-------T--NV-T------------I-----N

NL----V------------LQN---LVMQDVTN-PDI-----------------------

-------------------------------------------------LRF---DWT--

-------------------------------FTWFS------------------------

---------------------------K--VNH-------LSIINVNFNYV-PCDA--WG

QMTN--VVTLNISGNR--------L-L--------S--------------GYLF--NL--

-----LCKN-------NV--L-PK-IETFNVSDNQ-------------V--D-SL-KTVS

-LLTANW--PK-LTKLDLAD-N-A--------------------------I-G---S---

--------IN------------E--SCV-W----------K-----A-------------

--NIKHLILRNNM--F----S-VD--------------VFQCLPR---------------

TLEYL----------DMS-------NSQL----ER--L-----DMS-----YF--E-KAT

----------------------------------------------------------N-

---------------------ITELILRNNKIKFI-PKGW--HS-PNLRVLALEGNSFGV

IDQG---------S-FQ------------NLHQ-------------LTRLTAG-NNPYHC

SCDL--H-AF----I--K--------------E----T-------LN----------EGS

-------L-----------SL--------SD-W-----PEDYYC-YHP-TD----LL---

-DTRIESF--KPGKLE-C-DVRL-----VVAVS--VSVTAFVVIVCMLL----C--YKFD

I-PW-YLRTT---CQ---I-----IQ----S--K------------------Y------R

A-R---N----S------------------------PE----T---REF---P----YHA

FISYSYSDA---DWVRRVLLPRL----ENS---N--------------PP----------

---Y---RICIHERDFL----------------P--GK-WIIDNII-ENIENSRKIIFVL

SH------NFV-----------NSE-------WCNY-ELYFAHQRAIG--H-A-FEDVIL

VVKE--N-VSMEDL----------------------------------------------

--PKRFFRLRK-LLSTKTYLEWP----LE-------ENRQP-------FFWVQLKSIM--

--GKG------------SR-----------------------------------------

-------------------S-LSG-Q-------DN-----VSVVNETV------SVGGDG

ISEEPVTVSRDERIE---------------TVELGFSTNF-------------------

>TLR14_Agca

------------------------------------------------------------

------------------------------------------------------------

------------------------------------------------------------

------------------------------------------------------------

------------------------------------------------------------

------------------------------------------------------------

------------------------M--FPR--D---------------------------

--------------------------------G---P--L--V--L------LL----I-

---FL---FRIL-------------------------------------ESSC--DR---

--RS-G-G----------------------------TSYVYLS-----------------

-----------G------EN--LQ-D-------VP----Q---NL-----S-------E-

D--LEYL-------DLSYN-------K---I--T-----------ELKRSSFSR--Y-RR

L-----------ETLNLS-YNN------------------I-SN--IEN--GSFQLNLFL

RNLSLF------N--------NS-L-LE-----------------------FP---A-SS

------L-KPL---KN---LKILDLS-NN-----F-YRFST------LG---K---E---

----FT-KLV------------N-------------------------------------

-------------LQELSIG-G-P-LVTTVL----------------RDDFAVLK----N

ISLQKFSLKTKS--SL--DS-YK----PG--A------------FSV----------LNT

KA-LWLD-----IA--------LDNNATL-------L----------------ALML---

-KDL--TG----K----NLT---------------TLRF----RNLF----E-----F--

S--YY---D-R-V---SDI-------FS----SLPD-------I--NI--TNLVF----Y

RGK----------------FNENL---LR-L----------V-LKNVQI----S-KI-Q-

-NL---------LLMS---------------------VD---------F--ARS-----P

------------D----A-----N--K-------T--NG-T------------I-----N

NL----V------------LDN---LVIQDVTN-PDI-----------------------

-------------------------------------------------LRF---DWT--

-------------------------------FTWFS------------------------

---------------------------K--VTH-------LSIINVNFNFV-PCDA--WG

QMTN--VVTLNISSNR--------L-V--------S--------------GYLY--NL--

-----LCKR-------NV--L-PN-IETFNVSNNL-------------V--D-SL-KTVS

-QLTAEW--PM-LTLVDLAD-N-T--------------------------I-G---S---

--------IN------------E--SCQ-W----------K-----A-------------

--HIQVLILRNNI--L----T-VD--------------VFHCLPT---------------

TLEYL----------DMS-------NSQL----DR--L-----DMG-----YF--N-RAT

----------------------------------------------------------N-

---------------------ITELILRNNKIKFI-PAGW--HS-PNLQVLALEGNSFGV

IDQG---------S-FQ------------NLPQ-------------LRTLTAG-NNPYYC

TCDL--N-AF----I--T--------------E----T-------LN----------EGS

-------L-----------SL--------SD-W-----PDQYYC-YHP-QH----LL---

-DTRIESF--RPGKLE-C-DMRL-----VVAVS--VSVTACVVFVCMML----C--YRFD

V-LW-YVRAT---CQ---I-----IQ----S--K------------------Y------R

A-R---K----N------------------------QE----S---REF---A----YHA

FISYSYSDA---DWVRGVLLPHL----ENA---N--------------PP----------

---Y---TICIHERDFL----------------P--GK-WIIDNII-ENIENSRKIIFVL

SH------NFV-----------NSE-------WCNY-ELYFAHQRAIG--H-A-FEDVIL

VVKE--N-VSMEDL----------------------------------------------

--PKRFHRLRR-LLNTKTYLEWP----LE-------ENRQA-------FFWVQLKSIL--

--GKG------------GK-----------------------------------------

-------------------S-ASG-V-------DN-----LSLVNETV------NVGAES

SSGEPITMTAD--------------------CSLQLPGS--------------------

>TLR14_Mifi

------------------------------------------------------------

------------------------------------------------------------

------------------------------------------------------------

------------------------------------------------------------

------------------------------------------------------------

------------------------------------------------------------

------------------------M--FLL--S---------------------------

--------------------------------G---R--F--L--P------LT----F-

---LLIVGLGAL-----------------P-----------------WCQPAC--VF---

--SS-D------------------------------GMYVTCK-----------------

-----------G------EN--LY-A-------VP----Q---NF-----S-------E-

D--LVYL-------DLSYN-------K---I--S-----------EIKPSDFSQ--Y-PK

L-----------DVINLS-YNN------------------I-SV--IQN--GTFQYNLFL

RNLSLF------N--------NC-L-AE-----------------------IP---S-SS

------L-ESL---DN---LKILDLS-NN-----F-YRHVT------LG---D---V---

----FY-RLV------------N-------------------------------------

-------------LQEFSIG-G-P-LVSEIL----------------KDDFASIQ----N

ITLKKFSLKSKS--SL--DY-YE----PG--A------------FSL----------LDT

QI-LWLD-----IA--------LDANATF-------L----------------IHML---

-KDL--EN----K----TVT---------------SIRF----RNLF----E-----N--

S--YY---S-M-D---DDI-------FF----NLQN-------I--NL--TDLSF----Y

RGK----------------FNEKL---LR-I----------I-LLNVQR----S-SI-I-

-NL---------YLYS---------------------ID---------F--ARS-----P

------------D----A--------V-------K--DG-S------------I-----N

DL----N------------LRN---LVIQDVTN-PDI-----------------------

-------------------------------------------------LRF---DWT--

-------------------------------FTWFS------------------------

---------------------------K--ITN-------LSIRNVNFNFV-PCDA--WG

QMTN--LVNLDISSNR--------L-L--------A--------------SYLY--NL--

-----LCKD-------IV--L-PN-IEVFNASDNE-------------M--R-SL-RTLS

-LLTAQW--PR-LTQLDLSS-N-N--------------------------L-G---S---

--------NN------------E--VCE-W----------K-----S-------------

--DIRVLILRFNI--L----L-YS--------------IFNCLPT---------------

TLEYL----------DLS-------NSNL----DR--L-----DMN-----YF--K-KTQ

----------------------------------------------------------N-

---------------------LQELILSNNKIKFI-PSGW--YT-PNLQVLALEGNSFGS

IDKG---------S-FH------------NLQH-------------LKILTAG-NNPYHC

TCDL--Y-TF----F--S--------------E----T-------LS----------AGS

-------P-----------TL--------PD-W-----PKNYYC-YHP-EQ----LL---

-DTRVASY--SPGKLE-C-NVGL-----VVAIS--VSITAVVVIAGMLL----C--WRFD

V-PW-YIRVT---FQ---I-----IR----S--K------------------Y------R

S-K---N----D------------------------KP----N---KDY---A----YHA

FISYSYFDA---EWVRGVLLPHL----ETA---N--------------PP----------

---Y---QICIHERDFQ----------------P--GR-WIIDNII-ENIENSRKIIFVL

SH------NFI-----------NSE-------WCNY-ELYFAHQRAIG--H-A-FEDVIL

VVKE--A-VSMEDL----------------------------------------------

--PKRFHRLRK-MLRTKTYLEWP----LE-------ENRQP-------FFWLQLKSIL--

--GKS------------NM-----------------------------------------

-------------------G-VSG-L-------DN-----VSVSVEGT------ISSMEG

SPDEVTDSL---------------------DVVATSEDTKL------------------

>TLR14_Raor

------------------------------------------------------------

------------------------------------------------------------

------------------------------------------------------------

------------------------------------------------------------

------------------------------------------------------------

------------------------------------------------------------

------------------------M--SQV--T---------------------------

--------------------------------G---W--L--M--T------LM----V-

---L----IGTL-----------------T-----------------WCQADC--NI---

--ST-N------------------------------GTNVTCK-----------------

-----------G------QN--LK-Q-------VP----Q---NL-----S-------E-

S--LEYL-------DLSYN-------E---I--S-----------EMRATDFMQ--Y-PN

L-----------QTLNLR-YNN------------------I-SY--IEN--GTFQYNLLL

SNLSLY------Q--------NL-L-SE-----------------------IP---N-SP

------L-IDL---KN---LKILDVS-NN-----L-YERVT------LG---D---V---

----FG-TLV------------Y-------------------------------------

-------------LTDLSIG-G-P-LVSGIF----------------KHDFASIE----N

ISLDRFSLKSKS--SL--DY-YE----PG--A------------FSV----------LNT

RN-LWLD-----IA--------VDTNATQ-------L----------------SDML---

-KDL--TN----K----SLT---------------SLNF----INLF----E-----F--

S--YY---V-G-N---KDL-------FS----YLPS-------I--DL--RDLAF----Y

RGK----------------FNENL---LY-L----------L-LMNVQI----S-SV-K-

-NL---------YLFS---------------------LD---------F--ARS-----P

------------N----L-----D--I-------S--DV-A------------I-----D

NL----S------------LEN---LVIQDVIN-PDI-----------------------

-------------------------------------------------LGF---DLA--

-------------------------------VPWFG------------------------

---------------------------K--VAN-------LSVINMNFDQL-PCNI--WT

QLTN--LVMLNISGNR--------L-L--------A--------------AYLY--NM--

-----LCKT-------TV--L-PN-IEIFNASYNS-------------M--R-SL-KTIS

-LLTAQW--PK-LSYLDFTS-N-R--------------------------F-G---T---

--------LD------------E--FCV-W----------T-----S-------------

--NIKVLILRGNV--L----Q-YK--------------VFQCLPT---------------

TVEYL----------DLS-------HSQL----EQ--L-----NMS-----YF--N-LAT

----------------------------------------------------------N-

---------------------LTELNLSNNKIKSI-PYNW--QS-PSIQVLALQGNSFGI

IDKG---------S-FK------------NLPQ-------------LRNLTAG-SNPYYC

MCDL--Y-DF----F--S--------------D----V-------LE----------DRS

-------L-----------VL--------TD-W-----PDDYYC-YHP-QN----LM---

-ETKIDSF--RPGKLD-C-NVGL-----VVAIS--ASVTAFVIIVCMLL----G--WKFN

A-PW-YIKAT---CQ---I-----IK----S--R------------------Y------R

A-R---K----A------------------------DQ----V---RNY---D----YHA

FISYSCFDA---DWVRRVLLPQL----EDS---S--------------PP----------

---Y---KVCIHERDFQ----------------P--GK-WIIDNII-ENIEKSRKIIFVL

SH------NFI-----------NSE-------WCNY-ELYFAHQRAIG--H-A-FADVIL

VVKD--S-VGMEDL----------------------------------------------

--PKRFHRLRK-LLRTKTYLEWP----EE-------GSRQP-------FFWVQLKNIL--

--GRD------------SL-----------------------------------------

-------------------S-VMG-Q-------DN-----LTYL----------------

-----------------------------------------------------------

>TLR14.2_Raca

------------------------------------------------------------

------------------------------------------------------------

------------------------------------------------------------

------------------------------------------------------------

------------------------------------------------------------

------------------------------------------------------------

------------------------M--SQF--T---------------------------

--------------------------------G---W--L--M--T------LM----V-

---L----IGTL-----------------T-----------------RCKANC--NI---

--ST-D------------------------------GKRVNCK-----------------

-----------G------QY--LE-Q-------IP----Q---NL-----S-------E-

S--LEYL-------DISYN-------R---I--S-----------EIRADNFIQ--Y-PN

L-----------QMLNLR-FNN------------------I-SY--IEN--GTFQYNLLL

TNLSLS------N--------NL-L-SE-----------------------IP---N-SS

------L-MGL---KN---LQILDLS-NN-----L-YQRVT------LG---D---V---

----FS-TLV------------N-------------------------------------

-------------LTDLSIG-G-P-RISGIF----------------KGDFASIK----N

LSLNRFALKSKS--SL--DY-YD----PG--A------------FSV----------LNT

KN-LWLD-----IA--------VDTNATL-------L----------------SDML---

-KDL--TN----K----SLT---------------SLNF----INLF----E-----F--

S--YY---V-D-N---KDI-------FS----YLPS-------I--DL--RDLVF----Y

QGE----------------FSENL---LY-L----------L-LMNVQI----S-SV-K-

-NL---------YLFS---------------------LD---------F--ALS-----P

------------N----L-----N--I-------S--DV-A------------I-----D

NL----H------------LEN---LVIQDVIN-PDI-----------------------

-------------------------------------------------LGF---DLT--

-------------------------------FTWFS------------------------

---------------------------K--IVN-------LSVINMNFNHV-PCNA--WA

QMTN--LVVLNVSSNR--------L-L--------A--------------TYLY--NL--

-----LCKN-------TV--L-PN-IEIFNASYNA-------------M--H-SL-KTIS

-LLTAQW--PK-LTYLDLTS-N-K--------------------------F-G---T---

--------LN------------E--FCV-W----------T-----A-------------

--NIKVLILRGNV--L----Q-YE--------------VFQCLPT---------------

TVENL----------DLS-------NSQL----EQ--L-----NMS-----YF--N-LAT

----------------------------------------------------------N-

---------------------LTELNLSNNKIKFI-PYNW--QS-PSIQVLALEGNSFGI

IDKG---------S-FK------------YLPQ-------------LKNLMAG-NNPYYC

MCDL--Y-DF----F--T--------------D----V-------LD----------EGS

-------L-----------VL--------TD-W-----PDDYYC-YHP-QN----LM---

-GTRIDHF--HPGKLD-C-NVGL-----VVAIS--VSVTAFVIIVCMLL----G--WKFN

A-PW-YIKAT---CQ---I-----IK----S--R------------------Y------R

S-Q---K----A------------------------DQ----T---RNY---D----YHA

FISYSCFDA---DWVRGVLLPQL----ENS---N--------------PA----------

---Y---KVCIHERDFQ----------------P--GK-WIIDNII-ENIEQSRKVIFIL

SH------NFI-----------NSE-------WCNY-ELYFAHQRAIG--H-A-FADVIL

VVKD--S-VGMEDL----------------------------------------------

--PKRFHRLRK-LLRTKTYLEWP----KE-------DSRQP-------FFWVQLKNIL--

--GKD------------SL-----------------------------------------

-------------------S-IMG-Q-------DN-----LT------------------

-----------------------------------------------------------

>TLR14.3_Raca

------------------------------------------------------------

------------------------------------------------------------

------------------------------------------------------------

------------------------------------------------------------

------------------------------------------------------------

------------------------------------------------------------

-----------MSQVTGW------L--MTL--M---------VL----------------

---------------------------------------I--E--T------LT----R-

----------------------------------------------------C--QA---

-NCN-I---------------------------STDGKRVTCK-----------------

-----------G------QN--LK-Q-------VP----Q-N--L-----S-------E-

S--VEYL-------DISFN-------E---I--S-----------EMRATDFMQ--Y-PN

V-----------QTLNLS-YNN------------------I-SY--IEN--RTFQYNLLL

SNLSLF------H--------NL-L-SE-----------------------IP---N-SP

------L-IDL---KN---LQILDLS-NN-----L-YKRIT------LG-------DI--

----FS-TLR------------N-------------------------------------

-------------LTDLSMG-G-P-MVSSIF----------------KHDFASIE----N

ISLDRFSLKSKS--SL--GY-YE----PG-----------------A---FSV----LNT

QN-LWLD-----IA--------VDTNATLL-----------------------SDML---

-KDL--TN----K----SLM-----------------SL----NFIN----L-----F--

EF-SY---Y-VGN---KDI--------FS---YLPS-------I--DL--RDLVF----Y

RGK----------------FNENL---L-------------Y-LLLMNV--QIS-SV-K-

-NL---------YLFS---------------------LD---------F--ARS-----P

------------N----L-N---I--S-------Y-------------------------

------V---A--------IDH---LHLENLMI-QDV-----------------------

-------------------------------------------------INPDILGFD--

-------------------------------LTFPW------------------------

-------------------------FSK--VTN-------LSVINMNFQQV-PCKV--WA

QLTN--LVVLNLSGNR--------L-M--------A------AYLYN-L-LCKN------

----------------TL--L-PN-IEIFNASYNA-------------M--R-SL-KTIS

-LLTAQW--PK-LTYLDFTS-N-K--------------------------F-G---RL--

--------------------N-E--FCV-W----------T-----S-------------

--NIKVLILRGNV--L----Q-YE--------------VFQCLPT---------------

TVEYL----------DLS-------HSQL----EQ--L-----NIS-----YF--N-LAT

----------------------------------------------------------N-

---------------------LTELNLSNNKIKFI-PYNWRS---PSIQVLALEGNSFGI

IDKG---------S-FK------------YLPQ-------------LKNLMAG-NNPYYC

MCDL--YDFF----T--D--------------V----L-------ED-------------

-------G-----------SL----VL--TD-W-----PNDYYC-YHP-QN----LM---

-GTRIDHF--RPGKFD-C-NVGL-----VVAISASVTAFFIIVCMLLG--------WKFN

A-PW-YLKAT---CQ---I-----IK----S--R--------------------------

------Y----R-SKK---------------A----DQ----T---RNY---D----YHA

FISYSCFDA---DWVRRVLLPQL----ENS---N--------------PA----------

-Y-----KVCIHERDFQ----------------P--GK-WIIDNII-ENIEKSRKIIFIL

SH------NFI-----------NSE-------WCNY-ELYFAHQRAIG----HAFADVIL

VVKD--S-VGMEDL----------------------------------------------

--PKRFHRLRK-LLRTKTYLEWP----EE-------DSRQP-------FFWVQLKNIL--

--GKD------------SL-----------------------------------------

---------------------SIMGQ-------DN-----LT------------------

-----------------------------------------------------------

>TLR14.1_Raca

------------------------------------------------------------

------------------------------------------------------------

------------------------------------------------------------

------------------------------------------------------------

------------------------------------------------------------

------------------------------------------------------------

-------FSEKEDADCLD------M--FLS--T---------------------------

--------------------------------G---W--L--M--T------LI----V-

---L----IGAL-----------------T-----------------RCQSAC--DV---

--SA-D------------------------------GNTVTCK-----------------

-----------G------QN--LE-G-------VP----Q---NL-----S-------E-

N--LEYL-------DLSYN-------K---I--S-----------EIRADDFLR--Y-PK

L-----------QILNLS-YNN------------------I-SY--IEN--GTFQYNLFI

RNLSLF------N--------NS-L-RE-----------------------IP---H-SS

------L-ENL---RY---LQILDMS-NN-----F-YERAT------LE---D---V---

----FS-SLV------------N-------------------------------------

-------------LKDLSVG-G-P-LISNIL----------------KDDFASME----N

ISLIRFALKSKS--SL--DY-YE----PG--A------------FSF----------LNT

QN-FWLD-----IA--------IDKKATL-------L----------------GDML---

-KDL--TN----K----TFT---------------SLRF----RNLF----E-----L--

S--YY---V-G-I---EDI-------FS----NLPY-------I--GL--KNLVF----Y

RGK----------------FNENL---LR-L----------V-LQNVQR----A-SI-K-

-SL---------YLVS---------------------ID---------F--ARS-----P

------------N----A-----N--K-------S--EV-T------------I-----N

NL----Y------------LEN---LVIQDVTN-PDI-----------------------

-------------------------------------------------LRF---DWT--

-------------------------------FTWFN------------------------

---------------------------K--VTN-------LSIINVNFNYV-PCDA--WG

QMTN--VVKLDISGNR--------L-L--------A--------------TYLY--NL--

-----LCKD-------NI--L-PN-IEIFNASNNI-------------M--R-SL-KTIS

-LLTAQW--PK-LTYLDLSS-N-N--------------------------L-G---S---

--------RN------------E--SCV-W----------T-----S-------------

--NPKVLILRDNL--L----Q-YE--------------VFQCLPT---------------

TVEYL----------DLS-------NSHL----DR--L-----NMN-----YF--A-MAT

----------------------------------------------------------N-

---------------------LTQLILSNNKIKFI-TSSW--NN-PNLHVLALEGNSFGV

IDKG---------S-FK------------NLPQ-------------LKSLTAG-NNPYHC

TCDL--Y-AF----F--T--------------D----T-------LK----------EGS

-------L-----------TL--------DD-W-----PDDYYC-YHP-PR----LL---

-DTRIEYF--HPGLLE-C-NVGL-----MVAIS--VSVTAFVVIVCMLL----C--WRYD

A-PW-YIRTT---CQ---I-----IQ----M--K------------------Y------R

S-Q---K----A------------------------VQ----S---RIY---E----YHA

FISYSYSDA---DWVRGVLLPHL----ENS---N--------------PP----------

---Y---KVCIHERDFQ----------------P--GK-WIIDNII-ENIENSCKIIFIL

SH------NFV-----------NSE-------WCNY-ELYFAHQRAIG--H-A-FEDVIL

VVKE--S-VSMEDL----------------------------------------------

--PKRFSRLRK-ILRTKTYLEWP----VE-------ESRQP-------FFWVQLKSIL--

--GKG------------NL-----------------------------------------

-------------------S-LIG-Q-------ES-----FAL-NETA------TVGQES

SSEEM----------AIPTE----------DSNVNSSVNQT------------------

>TLR14_Rapi

------------------------------------------------------------

------------------------------------------------------------

------------------------------------------------------------

------------------------------------------------------------

------------------------------------------------------------

------------------------------------------------------------

-----------MFLSTGW------L--MTL--I---------VL----------------

---------------------------------------V--G--A------LT----R-

----------------------------------------------------C--QS---

-ACD-I---------------------------SSDGNTVTCK-----------------

-----------G------QS--LE-E-------VP----Q-H--L-----S-------E-

N--LQYL-------DLSYN-------K---I--S-----------EILADDFLR--Y-PK

L-----------QILNLS-YNN------------------I-SY--IEN--GTFQYNLLI

RNLSLF------N--------NS-L-RE-----------------------IP---H-SS

------L-ENL---KY---LQILDLS-NN-----F-YERAT------LE-------DV--

----FS-SLA------------N-------------------------------------

-------------LKDLSVG-G-P-LISDIL----------------KDDFASME----N

ISLVRFALKSKS--SL--DY-YE----PG-----------------A---FSF----LNT

QN-FWLD-----IA--------IDKNATLL-----------------------GDML---

-KDL--TN----K----TFN---------------SLRF----RNLF----E--------

---LS---Y-YVG---IED-------IFS---YLPY-------I--GL--RHLVF----Y

RGK----------------FNENL---L-------------R-LVLQNV--QRS-SI-K-

-NL---------------------------------------------Y--LLS-----I

------------D----F-A---R--S-----------------------PNAN-----K

SE----V---P--------INN---LYLENLVI-QDV-----------------------

-------TN----------------------------------------PDILRFDWT--

-------------------------------FTWFN------------------------

---------------------------K--VTN-------LSIINVNFNYV-PCDA--WG

QMTN--VVKLDISGNR--------L-L-A------T------NLH-----NPLC--KD--

----------------NI--L-PN-IEIFNASNNI-------------M--R-SL-KTIS

-LLTAQW--PK-LTYLDLSS-N-N--------------------------L-G-------

--------SR----------N-E--SCV-W----------T-----S-------------

--NLKVLILRDNL--L--QYE-----------------VFQCLPT---------------

KVEYL----------DLS-------NSHL----DR--L-----NMN-----YF--D-MAT

----------------------------------------------------------N-

---------------------LTQLILSNNKIKFI-TSSWNS---PNLHILALEGNSFGA

IDKG---------S-FK------------DLPQ-------------LKHLTAG-NNPYHC

TCDL--YAFF----T--D--------------S----L-------KE-------------

-------G-----------SL----IL--DD-W-----PDDYYC-YHP-PH----LL---

-DTRIEYF--HPGLLE-C-NVGL-----VVAIS--VSVTAFVVIVCMLL----C--WRYD

A-PW-YIRTT---CQ---I-----IQ----M--K--------------------------

------Y----R-SRK---------------A----DQ----S---RIY---E----YHA

FISYSYSDA---DWVRGVLLPHL----ENS---N--------------PP----------

-Y-----KVCIHERDFQ----------------P--GK-WIIDNII-ENIENSCKIIFIL

SH------NFV-----------NSE-------WCNY-ELYFAHQRAIG----HAFEDVIL

VVKE--S-VSMEDL----------------------------------------------

--PKRFHRLRK-ILRTKTYLEWP----VE-------ESRQP-------FFWVQLKSIL--

--GKG------------NS-----R-------LIGQ------------------------

-------------------ESLSLMN-------ET-----AIVGQESSSEKMVVPAEDSN

VNSSVNQT---------------------------------------------------

>TLR14.2_Napa

------------------------------------------------------------

------------------------------------------------------------

------------------------------------------------------------

------------------------------------------------------------

----------------------------MKERCDDLPSVSRSMTPHAHTLSWLSDAGRLR

TSSGREQLLSLNRQRLIIPACGLIQDLWAGSGGAATGKWRSWNVSHVDRLDLRSVVIGHA

WPMAENSFCEKEDVDCLD------M--FQY--I---------------------------

--------------------------------G---W--L--M--T------LK----V-

---L----TVAL-----------------P-----------------WCQGTC--GV---

--SA-D------------------------------GKIVTCK-----------------

-----------G------QD--LE-R-------VP----P---NL-----S-------E-

N--LVYL-------DLSYN-------N---I--L-----------KIGAEDFLQ--Y-PN

L-----------HTLNLS-FNN------------------I-SY--IEN--KTFQYNALI

RNLSLS------N--------NA-L-SE-----------------------IP---H-SS

------L-EDL---GD---LQILDLS-NN-----F-YKRAT------LG---E---V---

----FC-NLV------------N-------------------------------------

-------------LKELSVG-G-P-RVSGIF----------------KDDFAPII----N

ISLNKFALKSKS--SL--DF-YE----PG--A------------FLV----------LDT

QI-LWLD-----IA--------VDTNATL-------L----------------SDML---

-KDL--KN----K----SIS---------------SLRL----RNLF----G-----S--

S--CY---M-G-I---EDI-------FA----YLPY-------I--DL--RDLVF----Y

RGI----------------FNENL---LR-L----------V-LQNVQV----S-SV-Q-

-NL---------YLLS---------------------ID---------F--ASS-----P

------------N----P-----N--I-------W--NT-T------------I-----D

NL----N------------LDN---LVIQDVTN-PDI-----------------------

-------------------------------------------------LQF---DWT--

-------------------------------FTWFS------------------------

---------------------------K--VTN-------LSITNVNFNIL-PCDA--WR

QMTN--LVVLNISGNR--------L-L--------A--------------TYLY--KQ--

-----LCRN-------NM--F-SN-IEIFNASYND-------------M--R-SL-KTLS

-LLTAGW--PK-LIYLDLRS-N-H--------------------------F-G---V---

--------LD------------E--TCV-W----------T-----S-------------

--DIKVLILRDNL--L----Q-YE--------------VFRCLPT---------------

TVEHL----------DLS-------NSHL----DQ--L-----DMH-----YF--D-KAT

----------------------------------------------------------N-

---------------------LTELILSNNKIKFI-PSGW--RS-PNLQVLALEGNSFGV

IDQG---------S-FR------------DLPQ-------------LTHLTAG-NNPYHC

TCDL--Y-AF----I--T--------------D----T-------LK----------EGS

-------L-----------GL--------DD-W-----PDNYYC-YHP-PY----LL---

-NTRIEHF--SPGRLE-C-SVGL-----VVAIS--ASVTAFVVIVCMFL----C--WRFN

A-PW-YIRAT---CQ---I-----IK----T--K------------------Y------R

S-R---K----A------------------------KH----S---RDY---D----YHA

FISYSYSDA---DWVRGVLLRHL----ENS---D--------------PP----------

---Y---RVCIHERDFL----------------P--GK-WIIDNII-ENIENSQKIIFIL

SH------NFI-----------NSE-------WCNY-ELYFAHQRAIG--H-A-MEDVIL

VVKE--S-VSMEDL----------------------------------------------

--PKRFHRLRK-ILRTKTYLEWP----ME-------ESRQP-------FFWVQLKSIL--

--AKG------------RS-----------------------------------------

-------------------S-IKG-Q-------DN-----VSLIIENA------IVRKKS

SFKETATPTEATPTETTPTEATATETTPTDDSNVNNAETGQT-----------------

>TLR14_Mabe

------------------------------------------------------------

------------------------------------------------------------

------------------------------------------------------------

------------------------------------------------------------

------------------------------------------------------------

------------------------------------------------------------

------------------------M--FQS--N---------------------------

--------------------------------G---W--L--M--A------LI----V-

---L----IGAL-----------------P-----------------PCQLAC--DI---

--NT-D------------------------------GRRVSCK-----------------

-----------G------QN--LE-R-------VP----Q---NL-----S-------E-

S--LEYL-------DISYN-------K---I--T-----------EIRADDFLQ--Y-LN

L-----------QTLNLS-FNN------------------I-SY--VEN--GTFQNNLLM

SNLSLF------N--------NS-L-QE-----------------------IP---N-SA

------L-EDL---RY---LQILDLS-NN-----F-YERAT------LG---D---V---

----FG-TLV------------N-------------------------------------

-------------LKDLSIG-G-P-LVSSIL----------------KDDFASIQ----N

ISLNRFALKSKS--SL--DY-YE----PG--A------------FSL----------LNT

QI-LWLD-----IA--------VDKEATL-------L----------------GDML---

-KDL--TN----K----SLT---------------SLRF----RNLF----E-----L--

S--YY---A-G-I---QDI-------FS----YLPC-------I--DL--RDLVF----Y

RGK----------------FNENL---LR-L----------I-LQNVQT----S-SI-K-

-NL---------YLLS---------------------ID---------F--ARS-----P

------------N----A-----N--K-------S--DI-R------------I-----N

DL----N------------LYS---LLIQDVTN-PDI-----------------------

-------------------------------------------------LRF---DWT--

-------------------------------FTWFN------------------------

---------------------------K--VIN-------LNIINVNFNYV-PCDA--WG

QMTN--VVMLNISSYR--------L-L--------A--------------SYLY--NL--

-----QCIG-------NI--L-PN-IKIFNASNND-------------M--R-SL-KTLS

-LLTAQW--PK-LTHLDLSS-N-N--------------------------L-G---S---

--------CN------------E--SCV-W----------M-----A-------------

--NIKVLILRDNF--L----Q-YE--------------VFLCLPT---------------

SVEYL----------DLS-------HSQL----ER--L-----NMR-----YF--D-MAT

----------------------------------------------------------N-

---------------------LTELILSNNKIKFI-PSGW--HS-PKLQVLALEGNSFGV

IDQG---------S-FH------------DLSQ-------------LTHLRAG-NNPYHC

TCDL--Y-AF----I--T--------------D----T-------LE----------ERS

-------L-----------VL--------DD-W-----PDDYYC-YHP-QY----LL---

-DTRIEHF--SPGRLE-C-NVGL-----VVAIS--VSVTAFVVIVGMLL----C--WRFD

V-PW-YIRAT---CQ---I-----IQ----M--K------------------Y------R

S-R---K----A------------------------DQ----S---RNY---D----YHA

FISYSYSDA---DWVRGILLPHL----ENS---E--------------PP----------

---Y---RVCIHERDFQ----------------P--GK-WIIDNII-ENIENSNKIIFIL

SH------SFV-----------NSE-------WCNY-ELYFAHQRSIG--H-A-FEDVIL

VVKE--S-VSMEDL----------------------------------------------

--PKRFHRLRK-ILRTKTYLEWP----ME-------ESRQP-------FFWVQLKSIL--

--GKD------------SL-----------------------------------------

-------------------S-VTG-Q-------DN-----LSLMNETT------IVGQES

SAKEV----------STPI-----------DSNVNISENQT------------------

>TLR14.1_Napa

------------------------------------------------------------

------------------------------------------------------------

------------------------------------------------------------

------------------------------------------------------------

------------------------------------------------------------

------------------------------------------------------------

------------------------M--FQS--I---------------------------

--------------------------------G---W--L--M--T------LI----V-

---L----TGAL-----------------P-----------------RCQGAC--GV---

--ST-D------------------------------GKSVTCK-----------------

-----------G------QN--LK-Q-------VP----Q---NL-----S-------E-

S--LVYL-------DLSYN-------K---I--S-----------AIGADDFFQ--Y-PK

L-----------HTLNLS-YNN------------------I-SY--VDN--RTFQYNVLI

RNLSLF------N--------NS-L-SE-----------------------IP---H-SS

------L-EDL---GD---LQIFDLS-NN-----L-YKRVT------LE---D---V---

----FG-TLV------------K-------------------------------------

-------------LKDLSVG-G-P-LVSGIF----------------KDDFASIQ----N

INLIRFALKSKS--SL--DY-YE----PG--A------------FSF----------LDT

QN-LWLD-----IA--------VDKNATL-------F----------------GAML---

-KDL--TN----K----SFT---------------SLRF----RNLF----E-----L--

S--YY---A-G-I---EDI-------FS----YLPY-------I--DV--RGLVF----Y

RGK----------------FNENL---LR-L----------V-LQNVQV----S-SI-R-

-NL---------YLVS---------------------ID---------F--ARS-----P

------------N----A-----N--K-------S--EV-I------------I-----D

NL----H------------LDN---LVIQDVTN-PDI-----------------------

-------------------------------------------------LRF---DWT--

-------------------------------FTWFN------------------------

---------------------------K--VTS-------LSIINVNFNFV-PCDA--WG

QMSN--VVVLNVSGNR--------L-L--------A--------------SYLF--NL--

-----LCSD-------HI--L-PN-IKIFNASDNV-------------M--R-SL-KTVS

-LLTARW--PK-LTHLDLRS-N-N--------------------------L-G---S---

--------LD------------E--TCV-W----------T-----S-------------

--NIKVLILKDNL--L----R-YE--------------VFRCLPT---------------

TVEHL----------DLS-------NSQL----ER--L-----NMH-----YF--D-MAT

----------------------------------------------------------N-

---------------------LTKLILSNNKIKFI-PSGW--RS-PNLQVLALEGNSFGV

IDQG---------S-FR------------DLPQ-------------LTHLTAG-NNPYHC

TCDL--Y-AF----I--T--------------D----T-------LK----------EGS

-------L-----------GL--------DD-W-----PDNYYC-YHP-PY----LL---

-DTRIEHF--SPGRLE-C-SVGL-----VVAIS--ASVTAFVVIVCMLL----C--WRFD

V-PW-YIRAT---CQ---I-----IQ----M--K------------------Y------R

S-R---K----A------------------------KH----S---RDY---D----YHA

FISYSYSDA---DWVRGVLLRHL----ENS---D--------------PP----------

---Y---RVCIHERDFL----------------P--GK-WIIDNII-ENIENSQKIIFIL

SH------NFI-----------NSE-------WCNY-ELYFAHQRAIG--H-A-FEDVIL

VVKE--S-VSMEDL----------------------------------------------

--PKRFHRLRK-ILRTKTYLEWP----ME-------ESRQP-------FFWVQLKSIL--

--GKG------------SS-----------------------------------------

-------------------S-IKG-Q-------DN-----VSLVNETV------IRGQES

SSEEMSTPTEATPTETTPTE----------DSDVNTPENQTFSCIL-------------

>TLR14_Qubo

------------------------------------------------------------

------------------------------------------------------------

------------------------------------------------------------

------------------------------------------------------------

------------------------------------------------------------

------------------------------------------------------------

------------------------M--FQS--I---------------------------

--------------------------------G---W--L--M--T------LT----V-

---L----IGAL-----------------P-----------------RCQGAC--GV---

--CN-D------------------------------GRIVTCK-----------------

-----------G------QN--LK-Q-------VP----Q---NL-----S-------E-

N--VEYL-------DLSYN-------K---I--S-----------AIRAEDFFK--Y-PN

L-----------HTLNLS-YNN------------------I-SY--VGN--GTFQYNALI

RNLSLF------N--------NS-L-RE-----------------------IP---H-SS

------L-EDL---GD---LQILDLS-NN-----F-YERAA------LE---D---V---

----FG-TLV------------N-------------------------------------

-------------LKDLSVG-G-P-LVSSIL----------------KDDFASIQ----N

ISLNRFALKSKS--SL--DY-YE----PG--A------------FSF----------LDT

QN-LWLD-----IA--------VDKNATL-------L----------------SAML---

-KDL--TN----K----SFT---------------SLRF----RNLF----E-----L--

S--YY---V-G-I---EDI-------FS----YLPS-------I--DL--RGLVF----Y

RGK----------------FNENL---LR-L----------V-LQNVQV----S-SI-R-

-NL---------YLVS---------------------ID---------F--ARS-----P

------------N----A-----N--K-------S--DV-T------------I-----D

NL----N------------LDN---LVIQDVTN-PDI-----------------------

-------------------------------------------------LRF---DWT--

-------------------------------FTWFN------------------------

---------------------------N--VVS-------LSIINVNFNFV-PCDA--WG

QMTN--VVVLNISGNR--------L-L--------A--------------TYLF--NL--

-----LCRD-------NI--L-PN-IEIFNASDNV-------------M--R-SL-KTIS

-LLTAQW--PK-LTYLDLRS-N-N--------------------------L-G---S---

--------LN------------E--SCV-W----------T-----S-------------

--NIKVLILKDNL--L----Q-YE--------------VFHCLPT---------------

TVEYL----------DLS-------NSQL----ER--L-----NMH-----YF--D-TAT

----------------------------------------------------------N-

---------------------LTELILSNNKLKFI-PSGW--RS-PNLRVLALEGNSFGV

IDQG---------S-FR------------DLPQ-------------LTHLTAG-NNPYHC

TCDL--Y-AF----I--T--------------D----T-------LE----------ERS

-------L-----------VL--------GD-W-----PDNYYC-YYP-PY----LL---

-DTRIEHF--RPGRLE-C-SVGL-----VVAIS--ASVTAFVVIVCMLL----C--WRFD

V-PW-YIRAT---CQ---I-----IQ----M--K------------------Y------R

SRR---K----A------------------------KH----S---RDY---D----YHA

FISYSYSDA---DWVRGVLLRHL----ENS---D--------------PP----------

---Y---RVCIHERDFL----------------P--GR-WIIDNII-ENIENSRKIIFIL

SH------NFV-----------NSE-------WCNY-ELYFAHQRAIG--H-A-YEDVIL

VVKE--S-VSMEDL----------------------------------------------

--PKRFHRLRK-ILRTKTYLEWP----ME-------ESRQP-------FFWVQLKSIL--

--GKG------------SS-----------------------------------------

-------------------S-LKG-Q-------DN-----VSLVHETV------IGGQES

NSEEM----------LTPTE----------DSNVNTTENQTCSYIL-------------

>TLR14.4_Xetr

------------------------------------------------------------

------------------------------------------------------------

------------------------------------------------------------

------------------------------------------------------------

------------------------------------------------------------

------------------------------------------------------------

----------------QV------P--FLQ--N---------LFFPNRCEDPGRVS----

-------------------------------AQ---Q--K--V--H------VC----F-

---LL---LNLL-----------------K---------------WTSCQNPC--QR---

--DS-T------------------------------NRYANCQ-----------------

-----------G------QD--LV-E-------VP----Q---DL-----P-------V-

T--LQSL-------DLSYN-------R---L--F-----------HIRYEDFSS--Y-TN

L-----------RALNLS-FNN------------------I-ST--IEC--GSFASNTLL

RNLTLF------N--------NS-L-TE-----------------------MP---S-TL

------F-EPL---LL---LEFLDVS-NN-----L-YNYST------LG---K---V---

----FE-NLV------------N-------------------------------------

-------------LQNLAIG-G-P-LVSKVL----------------KDDFAPIK----N

RSLKKFSLKTMS--KL--GF-YE----PG--A------------LEV----------LNT

RV-LWLD-----IS--------LDTNAQA-------L----------------PLIL---

-KDL--AG----K----TID---------------SLRF----RRLF----E-----S--

S--YY---T-D-T---MDL-------FC----GLVD-------I--NI--RELIF----F

RGM----------------FTENL---LH-Q----------A-LHSIQK----S-TI-Q-

-DL---------LLLS---------------------VK---------F--DRS-----L

------------N----T-----N--N-------T--HI-L------------F-----D

RL----Y------------LNS---LVIRDIAG-PHI-----------------------

-------------------------------------------------FKY---DWT--

-------------------------------FTWFS------------------------

---------------------------K--VRN-------LQLSRVHLGSL-PCIA--WR

QMGN--IECLDVSDNR--------L-I--------G--------------SNLY--NP--

-----SCWD-------GG--L-PK-LDTFIAANNN-------------L--Q-SL-RLIS

-LLTAKW--PK-LTKLDLSS-N-D--------------------------L-G---V---

--------YN------------E--VCT-W----------I-----P-------------

--NITTLILKGNT--L----K-MC--------------VFQCLPT---------------

SVELL----------DLS-------YSQL----EQ--L-----DLN-----YF--N-RAT

----------------------------------------------------------N-

---------------------LKELVLSHNKLNFI-SSDW--RS-PNLQVLHLEGNSISL

IDKG---------T-FK------------DLPS-------------LRRLTAG-NNPYDC

TCDL--Y-AF----F--S--------------T----L-------QN----------NDE

-------M-----------LL--------AD-W-----PYDYQC-FHP-QH----LR---

-DTDVEDY--TPWRVE-C-DVSL-----VITIS--VSTTAAIIILCMLV----C--WRFD

V-PW-YLRMT---WR---I-----VK----S--K------------------Y------R

S-K---K----S------------------------NK----S---REY---N----YHA

FISYSYSDA---DWVRGELLYRL----ESC---S--------------PP----------

---Y---RVCIHERDFL----------------P--GR-WIIDNII-ENIENSRKIIFVL

SS------NFI-----------NSE-------WCNY-ELYFAHQRAIG--H-S-LEDIIL

VVKE--K-VTMEDL----------------------------------------------

--PKRFHKLRK-MLRTKTYLEWP----SE-------PSKQH-------FFWIQLKSIL--

--GDA------------SV-----------------------------------------

-------------------S-LAG-Q-------EG-----LSVVNEAV------VG----

------PVFYL--------------------YHI-------------------------

>TLR14.3_Xetr

------------------------------------------------------------

------------------------------------------------------------

------------------------------------------------------------

------------------------------------------------------------

------------------------------------------------------------

------------------------------------------------------------

----------------MN------P--VLQ--N---------------------------

--------------------------------G---Q--L--G--L------WC----V-

---LV---LGLV-----------------R---------------ELWCQTPC--LV---

--DE-S------------------------------RRFVSCS-----------------

-----------G------RN--LV-E-------IP----K---NF-----S-------V-

T--LEEL-------DMSFN-------R---I--F-----------QIKSDDFSA--Y-TN

L-----------RALNLS-YNQ------------------I-AT--IEN--GSFNSNTQL

RSLTLF------N--------NS-L-TE-----------------------MP---S-AL

------L-EPL---HL---LEFLDMS-NN-----F-YNKST------LG---D---V---

----FQ-TLV------------N-------------------------------------

-------------LQTLSIG-G-P-LVSKVQ----------------KDDFVPIR----N

IGLQKFALKTMS--SL--TL-YE----EG--A------------FSV----------LNT

HV-LWFD-----IA--------LDTNPQA-------L----------------LLIL---

-KDL--KG----K----SFD---------------VLRF----RNLF----E-----I--

T--YY---T-D-T---VDI-------FS----WLPS-------I--ST--RELVF----Y

RGK----------------FNENL---LW-I----------M-LENIQR----S-SI-L-

-DL---------SLLS---------------------VD---------F--SRS-----H

------------S----A-----N--K-------T--NV-S------------I-----D

DL----R------------LRT---LRVKDVTN-PDI-----------------------

-------------------------------------------------LRF---DWT--

-------------------------------FTWFR------------------------

---------------------------K--ISN-------LYIINVNFNSV-PCDA--WS

EMSN--LEKLDMSTDE--------L-V--------D--------------TYLY--NP--

-----WCQD-------VA--L-PT-TDTFILAYNN-------------L--Q-SL-RMLS

-LLTAKW--PK-LATLDLRS-N-S--------------------------L-G---S---

--------ND------------E--MCT-W----------T-----P-------------

--SIRTVILKDNM--L----K-VG--------------VFQCLPT---------------

TVEFL----------DLS-------HSQL----EQ--L-----DMD-----YF--N-KAT

----------------------------------------------------------N-

---------------------LKQLILSHNKIKFI-SSEW--KS-PNLQVLALEDNSFGV

INVG---------S-FK------------DLPK-------------LRNLTAG-DNPYGC

TCDL--Y-RF----F--S--------------Q----I-------RE----------EGR

-------I-----------VL--------AD-W-----PQAYKC-YSP-PD----LL---

-DTKVEFY--NPGKVQ-C-DVRL-----VVAIS--VSTTAVVVILSMLL----C--WRFD

V-LW-YVQTM---FA---I-----VQ----S--K------------------Y------R

S-R---N----M------------------------GN----T---KEY---L----YHA

FISYSHSDA---DWVRGELLHQL----ESC---S--------------PP----------

---Y---RVCIHERDFL----------------P--GR-WIIDNII-ENIENSRKIIFVL

SR------NFV-----------NSE-------WCNY-ELYFAHQRAVG--H-A-LEDVIL

VVKE--K-VTMEDL----------------------------------------------

--PKRFQKLRK-LLRTKTYLEWP----LE-------HTRQH-------FFWIQLRSIL--

--GKV------------SS-----------------------------------------

-------------------P-VIG-Q-------DD-----LLVDNGAA------ASWDGP

ASEAHHED-EI--------------------YETLIPQ---------------------

>TLR14.3_Xela

------------------------------------------------------------

------------------------------------------------------------

------------------------------------------------------------

------------------------------------------------------------

------------------------------------------------------------

------------------------------------------------------------

----------------MN------T--AQQ--N---------------------------

--------------------------------G---I--L--A--L------WC----F-

---LV---LGLV-----------------R---------------ESWCQTPC--LV---

--DE-S------------------------------QRYVSCS-----------------

-----------G------LN--LV-E-------IP----K---SL-----P-------T-

T--LEDL-------DLSFN-------R---L--F-----------QIKSADFST--F-TN

L-----------RALNLS-YNE------------------I-SS--IEN--SSFNSNTRL

RSLTLF------N--------NS-L-ME-----------------------MP---S-TL

------L-EPL---LH---LEFLDMS-NN-----F-YNEST------LG---E---V---

----FK-TLV------------N-------------------------------------

-------------LQILSIG-G-P-LISKVQ----------------KDDFVPLQ----N

ISLQKFALKTMS--SL--LV-YE----EG--A------------FSL----------LNT

RV-LWID-----IA--------LDNNAQA-------L----------------PPML---

-KDL--AG----K----SFT---------------SLRF----RNLF----E-----T--

N--YY---T-G-A---VDI-------FS----WLAY-------I--NT--TELVF----Y

RGK----------------FNENL---LM-L----------I-LVNIQK----S-RI-L-

-DL---------SLLS---------------------VD---------F--SRS-----Q

------------S----A-----N--K-------T--NV-S------------I-----D

DL----Y------------LRT---LLVKDVTN-PDI-----------------------

-------------------------------------------------LRF---DWT--

-------------------------------FTWFS------------------------

---------------------------K--VNN-------LSIINVNFNFV-PCDA--WD

EMCN--LKKLDMSCDK--------L-V--------D--------------AYLY--NP--

-----KCHN-------VV--L-PK-IETYILANNN-------------L--Q-SL-HLLS

-LLTAKW--PR-LATLDLRS-N-S--------------------------L-G---S---

--------YN------------E--TCT-W----------T-----P-------------

--SITTVILKDNV--L----N-VY--------------VFQCLPT---------------

TVEFL----------DLS-------HSQL----ER--L-----DMD-----YF--N-KAT

----------------------------------------------------------N-

---------------------LKELILSQNKIKFI-SSDW--KC-PNLQVLALEDNSFGV

IDKG---------S-FK------------DLPK-------------LRNLTAG-DNPYHC

TCDL--Y-AF----F--S--------------D----I-------QE----------ESR

-------I-----------VL--------FD-W-----PEAYYC-YHP-PE----LL---

-DTKVAFY--TPGRVQ-C-DVRL-----VVAIS--VSTTAVVVIITMLL----C--WRFD

V-P---------------------------------------------------------

------------------------------------------------------------

----CHSDA---DWVRGELLHRL----ESC---S--------------PP----------

---Y---RVCIHERDFV----------------P--GK-WIIDNII-ENIENSRKIIFVL

SH------NFV-----------NSE-------WCNY-ELYFAHQRAIG--H-A-LEDIIL

VVKE--K-VTMEDL----------------------------------------------

--PKRFQKLRK-LLRTKTYLEWP----LE-------HTRQH-------FFWIQLKSIL--

--GKV------------SP-----------------------------------------

-------------------S-VID-Q-------ED-----LLVANGGA------ASSDGP

ASEVYQEDAEL--------------------YEALIPQ---------------------

>TLR14_Xemu

------------------------------------------------------------

------------------------------------------------------------

------------------------------------------------------------

------------------------------------------------------------

------------------------------------------------------------

------------------------------------------------------------

-----------MNTTQQN------G--ILG--L---------WC----------------

-------------------------------LL---VLGL--V--E------ES----W-

----------------------------------------------------C--QN---

-PCL-V---------------------------DESQRYVSCS-----------------

-----------G------LD--LV-E-------IP----K-S--L-----P-------T-

T--LEEL-------DLSYN-------R---L--F-----------QIKSADFST--Y-TN

L-----------QALNLS-YNE------------------I-SS--IEN--NSFNSNTRL

RSLTLF------N--------NS-L-TE-----------------------MP---S-AL

------L-EPL---RH---LEFLDMS-NN-----F-YNEST------LG-------QV--

----FK-TLV------------K-------------------------------------

-------------LKILSIG-G-P-LISKVK----------------KDDFVPLQ----N

ISLQKFALKTMS--SL--MS-YE----EG-----------------A---FSL----LNT

RV-LWFD-----IA--------LDNNAQAL-----------------------PLIL---

-KDL--AG----K----SFT---------------SLRF----RNLF----E-----T--

N--YY---T-D-T---VDI-------FL----GLAY-------I--DT--TELVF----Y

RGK---------------------------F----------N-ENLLKL--ILE-NI-Q-

-KS---------SILD---------------------LS---L---LSV-----------

------------D----F-S---R--S-------Q--SA-NKT------KVSID-----D

LY-----------------LRT---LFVKDVTN---------------------------

-------------------------------------------------PDILRFDWT--

-------------------------------FTWFR------------------------

---------------------------K--VSS-------LSIINVNFNFV-PCDA--WD

QMYN--LKRLDMSSDK--------L-V--------D------AYLYN-R-LCHD------

----------------VV--L-PK-IETFILADNN-------------L--Q-SL-RLLS

-LLTAKW--PR-LATLDLRS-N-S--------------------------L-G-------

--------SY----------N-E--TCT-W----------T-----P-------------

--SITAIILKDNV--L------NVW-------------VFQCLPT---------------

TVEFL----------DLS-------HSEL----ER--L-----DMD-----YF--N-KTP

----------------------------------------------------------N-

---------------------LKELILSRNKIKFI-SSDWKC---PKLQVLALEDNSFGV

IDKG---------S-FK------------DLPN-------------LRNLTAG-DNPYHC

TCDL--YAFF----S--E--------------I----Q-------EE-------------

-------S-----------RI----VL--VD-W-----PKAYYC-YHP-PE----LL---

-DTKVAFY--TPGRVQ-C-DVRL-----VVAIS--VSTTAVVVIITMLL----C--WRFD

V-PW-YVQTL---FA---I-----VQ----S--R--------------------------

------Y----R-SKN---------------T----GD----S---KEY---L----YHA

FVSYSHSDA---DWVRGELLYRL----ESC---S--------------PP----------

-Y-----RVCIHERDFL----------------P--GK-WIIDNII-ENIENSRKIIFVL

SH------NFV-----------NSE-------WCNY-ELYFAHQRAVG----HALEDIIL

VVKE--K-VTMKDL----------------------------------------------

--PKRFHKLRK-LLRTKTYLEWP----LE-------HTRQH-------FFWIQLKSIL--

--GKV------------SP-----------------------------------------

-------------------SVIDQED-------LL-----VINGGTASSDGPASDAHKED

AELYEALIPQ-------------------------------------------------

>TLR14.1_Xetr

------------------------------------------------------------

------------------------------------------------------------

------------------------------------------------------------

------------------------------------------------------------

------------------------------------------------------------

------------------------------------------------------------

----------------ME------K--LSH--H---------------------------

--------------------------------E---R--L--V--I------LC----I-

---LA---LGLV-----------------E---------------RAQGQTPC--KT---

--DG---------------------------------IYANCK-----------------

-----------G------RS--LA-A-------VP----K---DL-----P-------T-

T--LVEL-------DLSYN-------R---L--S-----------HIQFDDFAS--F-TH

L-----------RALNLS-YNN------------------I-SA--IET--GSFASNVLL

TNLTLF------N--------NS-L-TE-----------------------MP---S-AL

------F-EPL---RF---LQFLDIS-NN-----F-YNCAT------LG---A---E---

----FS-MLE------------N-------------------------------------

-------------LRNLSIG-G-P-LVSKVL----------------KGDFAPIK----N

ISLQRFSLKTMS--SL--WL-YE----KG--A------------FSD----------LNT

QS-LWLD-----IA--------LDTNPQA-------L----------------PRML---

-KDL--AG----K----AFS---------------SLRF----RNLF----E-----F--

T--YY---T-D-A---MDV-------FF----GLAD-------I--FI--KELTF----Y

RGK----------------FNENL---LR-L----------T-LKNVEK----S-NI-Q-

-DL---------FLLS---------------------ID---------F--ARS-----L

------------S----T-----N--R-------T--DI-R------------I-----N

DL----A------------LRR---LVIKDVTN-PDI-----------------------

-------------------------------------------------LRF---DWT--

-------------------------------FTWFN------------------------

---------------------------K--VSH-------LDIINVNFNFV-PCDA--WS

QMVN--VERLDISNNR--------L-L--------A--------------SNLY--NL--

-----LCQY-------SE--L-PN-LHTFIASDNN-------------M--R-SL-YTLS

-LLTLTW--PK-LATLDLTS-N-Y--------------------------L-G---A---

--------LD------------E--VCT-W----------T-----P-------------

--QITKLILKDNT--L----K-VG--------------VFKCLPV---------------

TVEHL----------DMS-------NSLL----ER--L-----DMD-----YF--N-RAT

----------------------------------------------------------K-

---------------------LKVLILSQNKLKFI-SRDW--KC-PNLQVLGLEGNSFSV

IDKG---------S-FK------------DLPE-------------LRRLTAG-DNPYHC

TCDL--Y-AF----F--T--------------E----T-------LT----------ERR

-------V-----------SL--------AD-W-----PEEYNC-YHP-PH----LL---

-DTKVEFY--NPGRVE-C-DVRL-----VVAIS--VSTTAVVVMLCMLL----C--WRFD

V-PW-YLRTT---CS---I-----VQ----S--K------------------Y------R

S-R---S----F------------------------HD----S---RDY---N----YHA

FISYSHSDA---DWVRGELLYRL----ESC---S--------------PP----------

---Y---RVCIHERDFL----------------P--GR-WIIDNII-ENIESSRKIIFVL

SR------NFV-----------NSE-------WCNY-ELYFAHQRAIG--H-A-FEDVIL

VVKE--K-VTMEDL----------------------------------------------

--PKRFQKLRK-MMRTKTYLEWP----SE-------QNRQH-------FFWIQLKSIL--

--GKA------------NP-----------------------------------------

-------------------P-VTS-Q-------ET-----LSVVSETV------AYGTCS

VSET--PSVPL--------------------GKVTLPSS--------------------

>TLR14.1_Xela

------------------------------------------------------------

------------------------------------------------------------

------------------------------------------------------------

------------------------------------------------------------

------------------------------------------------------------

------------------------------------------------------------

----------------ME------T--LRH--H---------------------------

--------------------------------E---R--L--A--M------LC----M-

---LV---LGLV-----------------A---------------WAQCQTPC--EI---

--NA---------------------------------NYANCK-----------------

-----------G------RS--LA-T-------VP----K---EL-----P-------A-

T--LVEL-------DLSYN-------R---L--S-----------QIKLDDFAF--F-TN

L-----------RALNLS-YNN------------------I-SV--IEN--GSFASNALL

TNLTLF------N--------NS-L-TE-----------------------MP---S-AL

------F-EPL---KF---LQVLDIS-NN-----F-YNCPT------LG---A---E---

----FS-MLE------------N-------------------------------------

-------------LQSLSVG-G-P-LVSKVL----------------KDDFAPIK----N

ISLQKFSLKTMS--SL--GF-YE----QG--A------------FSV----------LNT

QT-LWLD-----IA--------LDTNPQA-------L----------------PRML---

-KDL--AG----K----SFD---------------SLRF----RNLF----E-----F--

T--YY---M-D-A---LDV-------FS----GLAD-------I--FI--GELTF----Y

RGK----------------FNENL---LR-L----------T-LKNMEK----S-SV-Q-

-DL---------LLLS---------------------ID---------Y--ARS-----L

------------S----T-----N--R-------T--SV-R------------I-----K

DL----G------------LRN---LVIKDVTN-PDI-----------------------

-------------------------------------------------LRF---DWT--

-------------------------------FTWFS------------------------

---------------------------R--VSH-------LYIINVNFNFV-PCDA--WS

QMVS--VEELDISNNR--------L-L--------A--------------SYLY--NL--

-----LCQY-------SE--L-PN-LHTFIAANNN-------------M--R-SL-YTIS

-LLTSTW--PK-LDTLDLTS-N-Y--------------------------L-G---A---

--------RD------------E--ICT-W----------T-----P-------------

--KITKLILKDNT--L----K-VE--------------VFKCLPV---------------

TVEHL----------DMS-------NSLL----ER--L-----DMD-----YF--N-RAM

----------------------------------------------------------N-

---------------------LKELILSQNKIKFI-SRDW--KC-PNLQVLALEGNSFGV

IDKG---------S-FK------------DLPE-------------LRRLTAG-DNPYHC

TCDL--Y-AF----F--T--------------E----T-------LN----------EHK

-------I-----------LL--------SD-W-----PGAYNC-YHP-PQ----LL---

-DTKVEFY--TPGRVE-C-DVRL-----VVAIS--VSTTAVVVILCMLL----C--WKFD

V-PW-YLRTT---CS---I-----VQ----S--K------------------Y------R

S-R---S----F------------------------HE----G---GEC---N----YHA

FISYSHSDA---DWVRGELLYRL----ESC---S--------------PP----------

---Y---RVCIHERDFL----------------P--GR-WIIDNII-ENIENSRKIIFVL

SH------NFV-----------NSE-------WCNY-ELYFAHQRAIG--H-A-FEDVIL

VVKE--K-VTMEDL----------------------------------------------

--PKRFHKLRK-MLRTKTYLEWP----SE-------QNRQP-------FFWIQLKSIL--

--GKA------------SP-----------------------------------------

-------------------V-VTT-Q-------AS-----LSVVSETV------AFGSCS

VSGA--PLVQL--------------------SKTSS-----------------------

>TLR14.2_Xela

------------------------------------------------------------

------------------------------------------------------------

------------------------------------------------------------

------------------------------------------------------------

------------------------------------------------------------

------------------------------------------------------------

--------------METL------P--RHE--R---------LL----------------

---------------------------------------M--F--C------IL----V-

--------------------------------------------LGLVEGTQC--QT---

-PCE-TNG-----------------------------FYANCK-----------------

-----------G------RS--LA-A-------VP----K-D--L-----P-------T-

A--LEEL-------DLSYN-------R---L--F-----------QIKIDDFSS--F-TN

L-----------RALNLS-YNN------------------I-SF--IEN--GSFASNTLL

INLTLF------N--------NS-L-TE-----------------------MP---S-AL

------F-EPL---QF---LQVLDIS-NN-----F-YSCPT------LG-------AE--

----FS-KLE------------N-------------------------------------

-------------LQSLSVG-G-P-LVSKVL----------------KGDFAPIK----N

ISLQKFSLKTMS--SL--GF-YE----KG-----------------A---FSV----LNT

QT-LWLD-----IA--------LDTNPQAL-----------------------PLIL---

-KDL--AG----K----SFN---------------SLRF----RNLF----------E--

F--TY---Y-MDA---MDV--------FS---GLAD-------I--FI--GELTF----Y

RGK----------------FNENL---L-------------R-LTLKNV--EKS-NV-Q-

-DL---------------------------------------------L--LLS-----I

------------DYARSL-S---T--N-------R--TS-VRI-----------------

KD----L---G--------LQN---LVIKDVTN---------------------------

-------------------------------------------------PDILRFDWT--

-------------------------------FTWFS------------------------

---------------------------R--VSH-------LYIINVNFNFV-PCDA--WS

QMVS--VERLDISSNR--------L-L-A------S------NLYNL-L-CQYS------

----------------EL----PN-LHTFIAANNN-------------M--R-SL-YTIS

-LLTSTW--PK-LDTLDLTS-N-Y--------------------------L-G-------

--------AR----------D-E--ICT-W----------T-----P-------------

--KITKLILKDNT--L--KVE-----------------VFKCLPV---------------

TVEHL----------DLS-------NSLL----ER--L-----DMD-----YF--N-RNT

----------------------------------------------------------N-

---------------------LKELILSQNKLKFI-SRDWKC---PNLQVLALEGNSFGV

IDTG---------S-FK------------DLPE-------------LRRLTAG-DNPYHC

TCDL--YAFF----T--E--------------T----L-------NE-------------

-------R-----------RI----IL--AD-W-----PQAYYC-YHP-PQ----LL---

-DTKVEFY--TPGQVE-C-DVRL-----VVAIS--VSITAVVVILCMLV----C--WRFD

I-PW-YLRTT---CS---I-----VQ----S--K--------------------------

------Y----R-SRS---------------F----RD----G---GEH---N----YHA

FISYSHSDA---DWVRAELLYRL----ENC---S--------------PP----------

-Y-----RICIHERDFL----------------P--GK-WIIDNII-ENIENSRKIIFVL

SH------NFV-----------NSE-------WCNY-ELYFAHQRAIG----HAFEDVIL

VVKE--K-VTMEDL----------------------------------------------

--PKRFHKLRK-MLRTKTYLEWP----SE-------QNRQH-------FFWIQLKSIL--

--GKV------------SP-----------------------------------------

-------------------PVTSQES-------LS-----VVSETVAFGSGTPSVELSKV

TLPCS------------------------------------------------------

>TLR14.2_Xetr

------------------------------------------------------------

------------------------------------------------------------

------------------------------------------------------------

------------------------------------------------------------

------------------------------------------------------------

------------------------------------------------------------

----------------ME------M--FRR--Y---------------------------

--------------------------------N---E--P--V--F------YC----F-

---LL---LALT-----------------K---------------GIWSQNSC--QV---

--DE-K------------------------------QKYANCQ-----------------

-----------G------QN--LN-D-------VP----K---DL-----P-------V-

T--LEVL-------DLSCN-------W---I--S-----------QIRVDDFSS--Y-TN

L-----------QALNLS-FNN------------------I-ST--IDN--SSFASNTQL

RNLTLF------N--------NG-L-TE-----------------------MP---S-TL

------L-EPL---LL---LEVLDLS-DN-----L-YNYST------LG---K---V---

----FK-TLA------------N-------------------------------------

-------------LRSLSIG-G-P-FVSKVL----------------RGDFVPIK----N

ISLQKFELKTRS--SL--RF-YQ----TG--A------------FSV----------LNT

DV-LVLV-----MT--------LDTNPKV-------L----------------PMIL---

-KDL--AG----K----SLD---------------ILSF----GNLF----E-----H--

N--YY---A-G-P---TNP-------FS----SLPD-------I--NV--RELIF----N

RGK----------------VNQKL---LQ-L----------I-LETIQT----S-SI-Q-

-DL---------SLLS---------------------VD---------F--DYS-----D

------------V----R-----T--T-------V--DV-K------------M-----D

NL----F------------LRS---LVIKDATN-PDI-----------------------

-------------------------------------------------LTF---DQT--

-------------------------------FTWFS------------------------

---------------------------K--VSN-------LYIINVNFNFA-QCDT--WS

QMKN--VERLDIRNNL--------L-L--------S--------------SDLY--NP--

-----SCKY-------GE--L-PK-LHTFTAANNN-------------L--Q-IL-KPIS

-LLTANW--PK-LSSLDLSS-N-Y--------------------------I-G---S---

--------ED------------E--NCS-W----------T-----P-------------

--NITTFILKNNL--L----T-PG--------------VFTCLPT---------------

TVRYL----------DLS-------NSRL----ES--L-----DMD-----YF--S-NAT

----------------------------------------------------------N-

---------------------LKTLVLSYNKLTSI-SSNW--SN-PFLQVLFLDNNIISI

IDKG---------S-FN------------NLPQ-------------LRTLTAG-NNPYHC

TCDL--Y-SF----F--S--------------D----V-------LG----------KNK

-------I-----------TI--------SD-W-----PQSYCC-YHP-QQ----LR---

-NTRVDIY--TPGSIE-C-NVGL-----LVAIT--VSTTAVVVIACMIM----C--WRLD

A-PW-YFRMM---CH---I-----VK----S--K------------------Y------R

S-K---K----A------------------------ND----S---REY---N----YHA

FISYSYSDA---DWVRGELLYRL----ESC---S--------------PP----------

---Y---RVCIHERDFL----------------P--GR-WIIDNII-DNIETSRKTIFVL

SH------NFV-----------NSE-------WCNY-ELYFAHQRAIG--H-S-FEDVIL

VVKE--N-VTLKDL----------------------------------------------

--PKRFHKLRK-MLRKKTYLEWP----SE-------PSKQH-------FFWIQLKNIL--

--G--------------SP-----------------------------------------

-------------------S-TAG------------------------------ALEPCP

ASEV--PSITY--------------------SKMNLPGS--------------------

>TLR14.5_Xela

------------------------------------------------------------

------------------------------------------------------------

------------------------------------------------------------

------------------------------------------------------------

------------------------------------------------------------

------------------------------------------------------------

----------------ME------M--FLH--Y---------------------------

--------------------------------G---E--L--V--W------YC----L-

---VL---LALA-----------------T---------------GKWCQNPC--QV---

--DK-E------------------------------QKYANCQ-----------------

-----------G------QN--LN-E-------VP----K---DL-----P-------I-

T--LEEL-------NLSCN-------W---L--Y-----------QITADDFSS--Y-TN

L-----------RALNVS-FNN------------------I-ST--IEN--NSFVSNTLL

RNLTLS------N--------NS-L-ME-----------------------MP---S-VL

------L-EPL---VL---LESLDLS-NN-----L-YNYST------LG---K---V---

----FK-TLV------------N-------------------------------------

-------------LQRLSIG-G-P-FVSKVL----------------KGDFVPIK----N

ISLQRFALKTKS--SL--KL-YQ----SR--A------------FSV----------LNT

KI-LILD-----IA--------LDKNAKA-------L----------------PLIL---

-KDL--AG----K----SLD---------------RLTF----ENLF----E-----Y--

T--YY---T-G-S---TNL-------FF----GLPD-------I--NI--RELVF----Y

GGK----------------VNDKL---LQ-L----------I-LESILI----S-RI-Q-

-NL---------LLLS---------------------VD---------F--DYT-----L

------------N----R-----S--K-------I--DV-K------------M-----D

KL----F------------LKN---LVIKDVMN-PDI-----------------------

-------------------------------------------------LTF---DRT--

-------------------------------FTWFG------------------------

---------------------------K--VRN-------LFIINVNFNFV-QCDA--WS

QMQN--VETLDISNNL--------L-L--------G--------------SYLY--NP--

-----LCKY-------SE--L-PK-LRKFTAANNN-------------L--K-YL-KPIS

-LLTANW--PK-LSSLDLSS-N-Y--------------------------L-G---S---

--------QY------------E--NCR-W----------T-----S-------------

--NITTLILKYNI--L----S-VG--------------VFTCLPT---------------

SVHYL----------DLS-------FSHL----ES--L-----NMD-----YF--K-NAT

----------------------------------------------------------N-

---------------------LKKLNLSYNKLTLI-SSEW--RH-PHLQVLFLDGNIFGV

IDKG---------S-FN------------NLPQ-------------LRTLTAG-DNPYHC

TCDL--Y-SF----F--S--------------D----I-------LS----------NSK

-------V-----------SL--------AD-W-----PQAYYC-YHP-QQ----LR---

-DTRVESY--TPGSVE-C-DVRL-----LVAIT--VSATAAVVIFCMIL----C--WRFD

A-PW-YFRMT---CH---I-----VK----S--K------------------Y------R

S-R---K----A------------------------NY----G---REY---L----YHA

FVSYSHSDA---DWVRGELLYRL----ESC---S--------------PP----------

---Y---RVCIHERDFL----------------P--GK-WIIDNII-DNIENSRKTIFVL

SH------NFV-----------NSE-------WCNY-ELYFAHQRAIG--H-S-FEDVIL

VVKE--N-VTLKDL----------------------------------------------

--PKRFYKLRK-MLRRKTYLEWP----SE-------PSKQH-------FFWIQLKNIL--

--G--------------SP-----------------------------------------

-------------------S-NGG-Q-------DV-----LSVMNEPV------APEPCP

VSEV--PLTTY--------------------SKMNLPGN--------------------

>TLR14.4_Xela

------------------------------------------------------------

------------------------------------------------------------

------------------------------------------------------------

------------------------------------------------------------

------------------------------------------------------------

------------------------------------------------------------

MSRLDPLDHGSEDVKDLE------M--FRH--Y---------------------------

--------------------------------G---E--P--L--L------YC----L-

---LL---LALV-----------------K---------------GTWSQNPC--QV---

--DH-E------------------------------QKYANCQ-----------------

-----------G------LN--LN-E-------VP----E---DL-----P-------I-

T--LEEL-------DLSCN-------W---L--Y-----------QIKFDDFSS--Y-TN

L-----------RALNLS-FNN------------------I-ST--IEN--DSFALNTLL

RNLTLF------N--------NS-L-TE-----------------------VP---S-VL

------L-EPL---LL---LESLDLS-NN-----L-YNYST------LG---K---V---

----FK-TLV------------N-------------------------------------

-------------LQSLSIG-G-P-FVSKVL----------------KGDFVPIK----N

ISLQTFSLKTKC--SL--VL-YQ----SG--A------------FSV----------LNT

NI-LTLD-----IA--------LDTNAKA-------L----------------LLIL---

-KDL--AG----K----SLD---------------RISF----WNLF----E-----Y--

T--YY---A-G-P---TNL-------FS----GLPD-------I--NI--RELVF----Y

GGK----------------VNEKL---LQ-L----------I-LESIQT----S-SI-Q-

-NL---------LLLS---------------------ID---------F--DYT-----L

------------D----R-----S--K-------V--DV-K------------M-----D

NL----F------------LKN---LVIKDVKN-PDI-----------------------

-------------------------------------------------LTF---DRT--

-------------------------------FTWFG------------------------

---------------------------R--VRN-------LYIINVNFNFV-QCDA--WS

QMEN--VERLDISNNL--------L-L--------G--------------SYLY--NP--

-----LCKY-------SE--L-PK-LRTFTAKNNN-------------L--Q-SL-KPIS

-LLTASW--PK-LATLDLSS-N-Y--------------------------L-G---S---

--------QY------------E--DCR-W----------T-----P-------------

--NITTLILKYNI--L----S-VG--------------VFTCLPT---------------

TVQYL----------DLS-------YSHL----EW--L-----NMD-----YF--N-NAT

----------------------------------------------------------N-

---------------------LKKLILSYNKLTLI-SSDW--RN-PHLQVLSLDGNIFSV

IDKG---------S-FN------------NLPQ-------------LRTLTAG-DNPYHC

TCDL--Y-AF----F--S--------------D----I-------LS----------NSK

-------V-----------SL--------AD-W-----PQAYYC-YHP-QQ----LR---

-GTRVNSY--TPGSVE-C-DVRL-----LVAIT--VSTTAVVVIFCMIL----C--WRFD

A-PW-YFRMT---CH---I-----VK----S--K------------------Y------R

S-R---K----A------------------------ND----G---KEY---L----YHA

FVSYCHSDA---DWVRGELLYRL----ESC---S--------------PP----------

---Y---RVCIHERDFL----------------P--GR-WIIDNII-DNIENSRKTIFVL

SH------NFV-----------NSE-------WCNY-ELYFAHQRAIS--H-S-FEDVIL

VVKE--N-VTLKDL----------------------------------------------

--PKRFYKLRK-MLRKKTYLEWP----SE-------LSKQH-------FFWIQLKNIL--

--G--------------SP-----------------------------------------

-------------------S-NGG-Q-------EG-----LSVVDETV------APEPCP

VSEI--PPIIY--------------------SKMNLPGS--------------------

>TLR14_Xean

------------------------------------------------------------

------------------------------------------------------------

------------------------------------------------------------

------------------------------------------------------------

------------------------------------------------------------

------------------------------------------------------------

----------MFRHYGEP------V--LYC--L---------LL----------------

---------------------------------------L--A--L------VK----G-

----------------------------------------------------TWSQN---

-SCQ-V---------------------------DQEQKYANCQ-----------------

-----------G------LN--LN-E-------VP----E-D--L-----P-------I-

T--LEEL-------DLSCN-------W---L--Y-----------QIKFDDFSS--Y-TN

L-----------RALNLS-FNN------------------I-ST--IEN--DSFALNTLL

RNLTLF------N--------NS-L-TE-----------------------VP---S-VL

------L-EPL---LL---LESLDLS-NN---LYN-YSTLG---------------NV--

----FK-TLV------------N-------------------------------------

-------------LQSLSIG-G-P-FVSKVQ----------------KVDFVPIK----N

ISLQTFSLKTKS--SL--VL-YQ----SG---------------AFS---VLN----TNS

LT-LDIT-----LD--------TNAKAL-------------------------PLML---

-KDL--AG----K----SLD---------------RISF----WNLF----E--------

---YT---Y-YAG---PIN-------LFS---GLPD-------I--DI--RELVF----Y

GGK----------------VNEKL---L-------------Q-LILESI--QTS-SI-Q-

-NL---------------------------------------------L--LTS-----I

------------D----F-D---Y--T-----------L-DRS------KVDVK-----M

DN----L---F--------LKN---LIIKDVKN---------------------------

-------------------------------------------------PDILTFDRT--

-------------------------------FTWFG------------------------

---------------------------R--VQN-------LYIINVNFNFV-QCDA--WS

QMEN--VERLDISNNL--------L-L-G------S------YIYNP-L-CKYS------

----------------EL----PK-LRTFTAANNN-------------L--Q-SL-KPIS

-LLTASW--PK-LATLDLSS-N-Y--------------------------L-G-------

--------SQ----------Y-E--DCR-W----------T-----P-------------

--NITTFILKYNI--L--SVG-----------------VFTCLPT---------------

TVQYL----------DLS-------YSHL----ES--L-----DMD-----YF--N-NAT

----------------------------------------------------------N-

---------------------LKKLVLSYNKLTFI-SSDWRN---PHLQVLSLDGNIFSL

IDKG---------S-FN------------NLPQ-------------LRTLTAG-DNPYHC

TCDL--YAFF----S--D--------------I------------QS-------------

-------N-SRV-------SL--------SD-W-----PQAYYC-YYP-QQ----LR---

-GTRVSSY--NPGSVE-C-DVRL-----LVAIT--VSTTAAVVIFCMIL----C--WRFD

A-PW-YFRMT---CH---I-----VK----S--K--------------------------

------Y----R-SRK---------------A----NY----G---KEY---L----YHA

FVSYSHSDA---DWVRGELLYRL----ESC---S--------------PP----------

-Y-----RVCIHERDFL----------------P--GK-WIIDNII-ENIENSRKIIFVL

SH------NFV-----------NSE-------WCNY-ELYFAHQRAIG----HAFEDIIL

VVKE--K-VTMEDL----------------------------------------------

--PKRFHKLRK-MLRTKTYLEWP----SE-------QNRQH-------FFWIQLKSIL--

--GKV------------SP-----------------------------------------

-------------------SVTSQES-------LS-----VVSEAVAFGSGTPSVELSKV

TLPCS------------------------------------------------------

>TLR18_Dare

------------------------------------------------------------

------------------------------------------------------------

------------------------------------------------------------

------------------------------------------------------------

------------------------------------------------------------

------------------------------------------------------------

------------------------------------------------------------

---------------------------------------M--L--V------PL----V-

---LI---LSLQ-----------------A----------VLHIKHAKAKQSC--TI---

--AF-D------------------------------KLSADCK-----------------

-----------G------LR--LD-S-------VP----T-T-QL-----P-------D-

S--LEEL-------DLSFN-------T---I--H-----------VIRKQDFVK--L-TY

L-----------RVLKLN-FNN------------------I-SL--VLD--DAFQGNLLL

EELNLF------N--------NS-L-TE-----------------------IP---F-KA

------L-EPL---TN---LKVLEMS-NN-----L-YSQAS------LG---A---A---

----FL-NFN------------Q-------------------------------------

-------------LKVLSIG-G-P-LVSSLG----------------SRDIYVLK----N

ISLDKFAVKTGT--GF--RD-YE----PG--Y------------FKS----------LST

KH-LWFD-----IA--------FDKNPDL-------L----------------PKML---

-KDL--AN----K----TFD---------------VLRF----RNLF----E-----F--

Q--YY---T-G-K---RDI-------FY----GLQY-------V--NA--QTLTF----Y

RGK----------------FNEEV---MR-M----------A-LKNVEI----S-PI-K-

-AL---------ELLL---------------------ID---------F--ARS-----Q

--------------------------N-------R--TQ-GSS----------V-----K

NL----S------------LDR---LVLSDISN-PDI-----------------------

-------------------------------------------------MRF---DWS--

-------------------------------FTWLN------------------------

---------------------------H--VRK-------FIVWNVNFNSV-PCDS--WP

EMKS--VELLDISNNQ--------L-L--------D--------------SYIY--NP--

-----LCDT----R--NT--L-HK-LDTFNVSHNR-------------L--T-SL-SDLA

-SLTKDF--VK-LTTIDMSH-N-Q--------------------------L-Q---YL--

--------GN------------G--ACD-W----------R-----K-------------

--TITKVIGNHNS--L----T-SD--------------SFKCLPT---------------

SVNFL----------DLS-------YSSL----DQ--L-----DMD-----YF--N-KAS

----------------------------------------------------------E-

---------------------LTELLLSGNKIKFI-PSGW--KN-RYLRTLALDGNSFGL

VSMK---------S-FK------------DMLS-------------LRVLTAG-NNPYHC

TCDL--Y-TF----I--Q--------------E----T-------TS----------KGK

-------V-----------TI--------TD-W-----PNNYKC-YHP-ER----LL---

-NTMVSKY--SPGHLA-C-DITL-----VIIIS--VSTTAAVVLVIMLL----C--YIFH

V-PW-YVKAT---YQ---I-----IR----A--K------------------Y------R

A-H---K----E-GL---------------------GQ----G---VDY---A----FHA

FISYSHSDA---DWVRNHLLPCL----ENV---K--------------PP----------

---Y---RLCIHERDFI----------------P--GK-WIIDNII-ENIENSRKVIFVL

SH------NFV-----------NSE-------WCNY-ELYFAQQRAIG--K-T-FSDVIL

VVKE--P-IDPTSL----------------------------------------------

--PSKFCKLKR-MLNTKTYLEWP----QQ-------PTEQN-------FFWIQLRSVL--

--GKP------------NS-----------------------------------------

-------------------I-RPR-T-------IS-----RHSRLSSAR-----SVSLIE

APQIQDPE---GPDEEDHQNSPQPSNKCQLTCIEVA-----------------------

>TLR18.1_Cyca

------------------------------------------------------------

------------------------------------------------------------

------------------------------------------------------------

------------------------------------------------------------

------------------------------------------------------------

------------------------------------------------------------

------------------------------------------------------------

---------------------------------------M--F--V------LL----A-

---LL---LSLQ-----------------A----------VVHIKPAKVNQSC--TI---

--TP-D------------------------------RRSADCK-----------------

-----------G------LR--LD-H-------VP----V-T-NL-----P-------V-

S--LEEL-------DLSFN-------T---I--Q-----------VISKQDLFK--L-SH

L-----------RVLKLN-FNN------------------I-SL--IVD--DAFQGNIFL

ENLNLF------N--------NS-L-TE-----------------------IP---F-KA

------L-EPL---TN---LKVLDMS-NN-----L-YTRAS------LD---A---S---

----FW-NFP------------Q-------------------------------------

-------------LKVLSIG-G-P-LVSSLG----------------SHDIFVLK----N

ISLDKFAIKTGT--GF--KS-YE----PG--Y------------FSN----------LNT

KN-LWFD-----IA--------FDKKPDM-------L----------------PMIL---

-KDL--AN----K----TFD---------------VLRF----RNLF----E-----F--

Q--YY---T-G-K---HDI-------FN----GLQN-------V--KT--QLLIF----Y

RGK----------------FNEGV---MR-M----------A-LVNLEI----S-SI-K-

-GL---------ELLF---------------------ID---------F--ARS-----Q

--------------------------N-------R--TQ-GSS----------I-----T

NL----S------------LEK---LVLSDISN-PDI-----------------------

-------------------------------------------------MRF---DWS--

-------------------------------FTWLN------------------------

---------------------------H--VRQ-------FIVWNVNFNSV-PCDS--WP

EMKS--FELLDISNNQ--------L-R--------D--------------SYIY--NP--

-----LCDT----R--NA--L-HK-LEIFNVSRNR-------------L--T-SL-SDLA

-SLTKDF--VK-LTTIDMSH-N-Q--------------------------L-Q---YM--

--------GN------------H--DCS-W----------R-----P-------------

--TITKVIAHHNS--L----M-SD--------------SFKCLPT---------------

SVSFL----------DLS-------YSSL----DQ--L-----DMD-----YF--N-KAS

----------------------------------------------------------N-

---------------------LTELLLSGNKIKFI-PSGW--RN-PYLRMLALDGNSFGL

VSMT---------S-FK------------DMPS-------------LRILTAG-NNPYHC

TCDL--Y-TF----I--Q--------------E----T-------TS----------NGK

-------V-----------TI--------TD-W-----PKNYKC-YHP-ER----FL---

-NTMVSQY--FPGHLT-C-NITL-----IIIIC--VSTTAVVVLALTLL----C--YIFH

V-PW-YIKAT---YQ---I-----IR----A--R------------------Y------R

A-H---K----E-GS---------------------GQ----A---VDY---T----FHA

FISYSHVDA---DWVRNQLLPCL----ENS---K--------------PP----------

---Y---RLCIHERDFT----------------P--GK-WIIDNII-DNIEDSRKVIFVL

SR------NFV-----------NSE-------WCNY-ELYFAQQRAIG--K-T-FSDVIL

IVKE--P-IDPTSL----------------------------------------------

--PSKFCKLKR-MLNTKTYLEWP----QQ-------PTEQS-------FFWVQLRSVL--

--GKP------------NI-----------------------------------------

-------------------I-RQR-T-------TS-----LHNRLSSVR-----SVSLID

LPQDWSSA---GTDEDGHQISDEPSNRCQQTLENLLEGHE-------------------

>TLR18.2_Cyca

------------------------------------------------------------

------------------------------------------------------------

------------------------------------------------------------

------------------------------------------------------------

------------------------------------------------------------

------------------------------------------------------------

------------------------------------------------------------

---------------------------------------M--F--V------SM----A-

---IL---LSLQ-----------------A----------VVHIKPAKAKLPC--TI---

--TS-D------------------------------KLSADCK-----------------

-----------G------LR--LD-H-------VP----I-T-NL-----P-------A-

S--LEEL-------DLSFN-------T---I--Q-----------VISKQDFFK--L-SH

L-----------RVLNLN-YNN------------------I-TL--IVD--DAFQGNIFL

ENLNLF------N--------NS-L-TE-----------------------IP---F-KA

------L-EPL---KN---LKVLEMS-NN-----L-YTQAS------LD---A---S---

----FL-KFT------------E-------------------------------------

-------------LKVLSIG-G-S-LVSILG----------------SHDIYVLK----N

ISLDKFAIKTGS--GF--KK-YE----PG--Y------------FSY----------LNT

KN-LWLD-----VA--------FDKKPDI-------L----------------PMIL---

-KDL--AN----K----TFD---------------VLRF----RNLF----E-----F--

Q--YY---T-D-K---QDI-------FY----GLRY-------V--NT--QLLTF----Y

RGK----------------FNEEV---MR-M----------A-LVNVEI----S-SI-K-

-GL---------ELLF---------------------ID---------F--ARS-----Q

--------------------------N-------R--TQ-GSS----------V-----T

KL----T------------LEK---LVLSDISN-PDI-----------------------

-------------------------------------------------MRF---DWS--

-------------------------------FTWLN------------------------

---------------------------H--VRQ-------FIVWNVNFNSV-PCDS--WQ

EMKS--VELLDISNNQ--------L-R--------D--------------SYIY--NP--

-----LCDT----T--NT--L-NK-LETFNVSRNR-------------L--T-SL-SDLA

-SLTKDF--VK-LTTIDMSH-N-Q--------------------------L-Q---YM--

--------GN------------H--VCY-W----------R-----Q-------------

--TITKVIAHHNI--L----T-SD--------------SFKCLPT---------------

SVSFL----------DLS-------YSSL----DQ--L-----DMD-----YF--N-KAS

----------------------------------------------------------N-

---------------------LTELLLSGNKIKFI-PSGW--RN-PYLRTLALDGNSFGL

VSMT---------S-FK------------DMPS-------------LRVLTAG-NNPYHC

ICDL--Y-TF----I--Q--------------E----T-------TN----------KGM

-------V-----------SI--------TD-W-----PDNYKC-YHP-EH----LL---

-NTMVSQY--FPGHLT-C-NITL-----IIIIS--VSTTAVVVFGLMLL----C--YIFH

V-PW-YIKAT---YQ---I-----IR----A--K------------------Y------R

A-H---K----E-GS---------------------GQ----D---VDY---T----FHA

FISYSHVDA---DWVRNQLLPCL----ENS---K--------------PP----------

---Y---RLCIHERDFT----------------P--GK-WIIDNII-ENIEGSRKVIFVL

SR------NFV-----------NSE-------WCNY-ELYFAQQRAIG--K-T-FSDVIL

ILKE--P-IDPTSL----------------------------------------------

--PSKYCKLKK-MLNTKTYLEWP----QQ-------PTEQS-------FFWVQLRSVL--

--GKP------------NI-----------------------------------------

-------------------T-RQR-T-------AS-----RHSRLSSMR-----SVSMIE

LPQDQSSADTKGTDEEGHQINDQPSNRCQQTLRELA-----------------------

>TLR18_Leoc

------------------------------------------------------------

------------------------------------------------------------

------------------------------------------------------------

------------------------------------------------------------

------------------------------------------------------------

------------------------------------------------------------

---------------------------MSS--S---------------------------

--------------------------------L---R--V--T--A------LA----N-

---LL---LCVW-----------------L---------TLVTVPATAGQFPC--PA---

--SD--------------------------------GVFANCK-----------------

-----------G------AN--LE-W-------VP------A-GL-----P-------L-

S--VEVL-------DLSYN-------R---L--R-----------AIRRADFAH--L-SR

L-----------RDLRLQ-YNN------------------I-SH--IEG--GSFLFNALL

EHLNIF------N--------NS-L-RA-----------------------IP---H-KA

------L-QPL---VK---LRSLDMS-NN-----L-YAQAT------LD---D---V---

----FS-AFT------------S-------------------------------------

-------------LESLSLG-G-P-LVQNLS----------------RGDFRALQ----K

TRLVKFAIKAGT--SL--VH-YE----PG--T------------LVG----------IQT

RE-MWLD-----IA--------LDDRPEI-------L----------------PNIL---

-RDF--AG----S----SFA---------------CLRF----RNLF----E-----F--

K--YY---T-G-T---EDI-------FL----GLRA-------I--KT--NKLVF----Y

RGK----------------FNENL---LR-M----------A-LLNIQG----S-PI-K-

-GL---------ELVA---------------------VD---------F--ARS-----P

------------S----F-----V--D-------N--GT-GSS----------V-----T

DL----S------------LDE---LVLSDISN-PDI-----------------------

-------------------------------------------------LRF---DWR--

-------------------------------FTWFS------------------------

---------------------------K--VRN-------LTIRNVNFNFV-PCDA--WD

ELRN--VEVMTIANNR--------L-R--------D--------------NFLY--NQ--

-----RCTY----K--DS--M-PS-LRTFDASANE-------------L--T-SL-STLT

-ALMGEW--RQ-LRVLDLSY-N-K--------------------------L-G---SLE-

----------------------E--SCT-W----------R-----Q-------------

--NITKMILHHNT--F----D-RS--------------VLDCLPT---------------

TLEYL----------DLS-------YSSL----DQ--L-----DMT-----YF--Q-KAT

----------------------------------------------------------S-

---------------------LRVLLLSDNKIKFI-PSGW--SS-PSLWSLVVDGNSFGL

INTG---------S-FH------------NMPS-------------LALLRAG-NNPYHC

TCDL--H-HF----F--Q--------------E----T-------LA----------RGT

-------V-----------NI--------TD-W-----PDDYIC-YHP-EP----LR---

-MTSVDRF--SPGRLA-C-DVRL-----VVAIS--VVITAIVVLVIVLL----C--YAFD

V-PW-YAKAT---YQ---I-----LR----A--R------------------Y------R

A-R---Q----E-GDL---------------L----G---------RDF---A----YHA

FVSYSHSDA---EWVRDQLLLRL----ESC---E--------------PP----------

---Y---RVCIHERDFM----------------P--GR-WIIDNII-ENIENSRKVIFVL

SH------HFV-----------NSE-------WCNY-ELYFAQQRAIG--K-T-FQDVIL

VVKE--A-IDPDSL----------------------------------------------

--PSKYCRLKK-MLSTKTYLEWP----EE-------TQRRA-------FFWAQLCSVL--

--GKP------------TL-----------------------------------------

-------------------R-V--------------------------------------

-----------------------------------------------------------

>TLR18_Clha

------------------------------------------------------------

------------------------------------------------------------

------------------------------------------------------------

------------------------------------------------------------

------------------------------------------------------------

------------------------------------------------------------

------------------------------------------------------------

---------------------------------------M--L--G------FT----L-

---TV---AAIV-----------------T---SVLNVHAYISIQQTELKHLC--SV---

--SD-D------------------------------QRRADCR-----------------

-----------G------QH--LD-R-------VP----K-E-NL-----P-------V-

P--LEEV-------DLSYN-------S---L--Q-----------RIHSTDFSH--L-PH

L-----------RVLNLE-YNN------------------I-SA--IDN--DAFTFNSLL

EDLNIF------N--------NS-L-AV-----------------------VP---N-KA

------L-TNL---TR---LRNLEMS-NN-----F-FGEVA------LG---D---V---

----FS-TFS------------G-------------------------------------

-------------LQELSVG-G-P-FVRELK----------------KSNLLALK----N

ISLFKFAFKSGS--SL--LK-YE----PG--S------------LEC----------LKT

NN-MWLD-----IA--------MDNIPNV-------L----------------NAML---

-NDL--AS----K----SFY---------------ALRF----RNLF----E-----F--

L--YY---T-G-E---NDI-------FQ----GLRH-------I--RA--QQLIF----H

RGK----------------FNENL---LR-M----------A-LINIQQ----S-PI-K-

-AL---------GLFF---------------------ID---------F--ARS-----P

------------T----F-----V--D-------S--GA-GSS----------V-----T

NL----A------------LDR---LVLSDISN-PDI-----------------------

-------------------------------------------------LRF---DWR--

-------------------------------FTWFN------------------------

---------------------------K--IRR-------LSIWNINFNTV-PCDA--WL

EMGA--MEVLDVSNNQ--------L-K--------D--------------HYLI--NR--

-----RCDY----A--GT--M-PV-LHTFNVSFNQ-------------L--T-RL-SDFA

-SFAGEF--KR-LQVLDVSH-N-Q--------------------------L-Q---TV--

--------DA------------S--ECN-W----------K-----Q-------------

--NISKLIAHHNA--L----V-VT--------------SLRCLPT---------------

TVLYL----------DLS-------FCNL----DQ--L-----DTN-----YF--L-KAI

----------------------------------------------------------N-

---------------------LRELLLSGNKIKFI-PFGW--KS-PNLQSLSLDGNSFGL

ISMN---------S-FK------------DMPS-------------LSTLRAG-NNPYHC

ICDL--H-TF----I--Q--------------K----T-------TS----------DRR

-------V-----------NI--------TD-W-----PGNYKC-YYP-EH----LV---

-NTMMADF--FPGRVA-C-DTRL-----VILIS--VITTAFVVFSIVLM----C--YFFN

V-PW-YTKAT---FQ---I-----IR----A--K------------------Y------R

A-N---K----E-GT---------------------RP----E---QDY---D----YHA

FISYSHSDA---EWVRNQLLPCL----ESS---R--------------PP----------

---Y---RVCIHERDFT----------------P--GK-WIIDNII-ENIENSLKVIVVL

SQ------HFV-----------DSE-------WCNY-ELYFAQQRAIG--K-T-FSDVIL

VVKE--P-IDPNSL----------------------------------------------

--PSKYCKLKK-MLSTKTYLEWP----QQ-------PKQQV-------FFWAQLKSVL--

--GRP------------SP-----------------------------------------

-------------------T-QHRPD-------SR-----HGTPTGSVV-----SVTELA

QLE--------------------------------------------------------

>TLR18_Asme

------------------------------------------------------------

------------------------------------------------------------

------------------------------------------------------------

------------------------------------------------------------

------------------------------------------------------------

------------------------------------------------------------

------------------------------------------------------------

---------------------------------------M--F--T------FL----S-

---LV---FGFQ-----------------A----------TLLLLPDRSEQSC--LI---

--SA-D------------------------------QRAANCQ-----------------

-----------G------FR--LQ-R-------VP----V-E-DL-----P-------P-

S--LEVL-------DLSYN-------M---L--Q-----------IIQNPDFAG--L-PL

L-----------RVLSLQ-FNN------------------I-SL--IED--EAFRDNPLL

EDVNIF------N--------NS-L-TT-----------------------IP---S-KA

------L-APL---SR---LQNLDMS-NN-----L-YTEAT------LD---D---T---

----VL-NFK------------N-------------------------------------

-------------LKVLSMG-G-P-YVSILK----------------TGDLNALQ----N

VSLEKFAIKTGS--SL--NV-YE----PG--Y------------LKN----------IQT

KN-MWLD-----VA--------IDNRSSA-------L----------------PLIL---

-KDL--AN----K----TFS---------------VLRF----RNLF----E-----F--

K--YY---T-D-K---EDI-------FD----GLKF-------I--NC--QQLIF----Y

RGK----------------FNEEL---LR-M----------A-LVNLQK----S-SI-K-

-FL---------GLLF---------------------ID---------F--ARS-----Q

------------T----F-----V--H-------T--DQ-ASS----------V-----T

NL----Q------------LEM---LVLSDISN-PDI-----------------------

-------------------------------------------------LRF---DWR--

-------------------------------FTWLN------------------------

---------------------------K--IRH-------LRVHNVNFNSV-PCDA--WE

EMNG--VEILDVSKNQ--------L-E--------D--------------RFLF--NQ--

-----QCDY----R--ET--M-PS-LHTFNLSTNQ-------------L--T-SL-SSFA

-SLAGEF--KH-LQTIDISY-N-Q--------------------------L-G---SE--

--------GN------------N--VCS-W----------K-----Q-------------

--NITTVIAHHNI--F----V-ID--------------SFKCLPT---------------

TVLYL----------DLS-------YCNL----DQ--L-----DMH-----YF--E-KAA

----------------------------------------------------------G-

---------------------LKEIYLSGNKIKFI-PSGW--RS-PNLNSLALDGNSFGI

ISMN---------S-FK------------GMPS-------------LEQLRAG-NNPYHC

TCDL--H-TF----I--Q--------------E----T-------TV----------KGK

-------V-----------QI--------TD-W-----PENYRC-YYP-AD----LL---

-NTMVSKY--FPGHVA-C-DIRL-----VIVIS--VVTTAVFVLAIMLM----C--YLFN

I-PW-YAKAT---YQ---I-----IR----A--K------------------Y------R

A-H---K----D-GA---------------------GM----S---AEY---T----FHA

FISYSHSDA---DWVRDKLLPCL----ENC---K--------------PS----------

---Y---RICIHERDFM----------------P--GK-WIIDNII-DNIENSHKVIFVL

SR------HFV-----------NSE-------WCNY-ELYFAQQRAIG--K-N-LRDVIL

VVKE--P-IDPKSL----------------------------------------------

--PSKYCKLKK-MLNTKTYLEWP----EQ-------PKQQA-------FFWAQLRSVM--

--GKP------------NL-----------------------------------------

-------------------L-KQN-S-------VI-----ENRRVSKLS-----RASVIE

LPEVKPQITAGTEELPERNTENCSLRMPLEQLA--------------------------

>TLR18_Icpu

------------------------------------------------------------

------------------------------------------------------------

------------------------------------------------------------

------------------------------------------------------------

------------------------------------------------------------

---------------------------------------------------MLRPCQHPL

IMFIHHTNNNPCCYISTQ------I--QMA--D---------------------------

--------------------------------V---R--V--F--T------LF----L-

---LV---FGFQ-----------------A----------SLLILPFQAEQSC--LI---

--SA-D------------------------------QRTADCR-----------------

-----------G------QR--LE-Q-------VP----V-E-EL-----P-------S-

S--LEKL-------DLSYN-------I---L--Q-----------VIRNDDFIE--L-PL

L-----------TVLWLK-FNN------------------I-SL--IED--EAFHKNLLL

EEVNMY------N--------NS-L-TT-----------------------IP---S-KA

------L-APL---SR---LKILEMA-NN-----L-YTEAT------LD---D---V---

----FL-NFK------------S-------------------------------------

-------------LKLLSLG-G-P-VLSTLK----------------KGDLDVLA----N

IHLERIAIRTGS--SL--DV-YD----PG--Y------------LKN----------IQT

AN-LWLD-----IA--------IDNRTYA-------L----------------PLIL---

-KDI--EN----K----TFE---------------AIRF----RNLF----A-----F--

M--YY---T-D-T---EDI-------FY----GLQN-------I--NS--QLLTF----H

HGK----------------FNEDL---LR-M----------A-LMNLAK----S-EI-K-

-AL---------GLFF---------------------VD---------F--TLS-----Q

------------T----F-----V--N-------S--EE-ESS----------L-----T

NL----A------------LDM---LVLSDISN-PDI-----------------------

-------------------------------------------------LRF---DWS--

-------------------------------FTWLN------------------------

---------------------------K--IRH-------LRINNVNFNIV-PCDA--WE

EMRG--VEILDVSKNN--------L-K--------D--------------NFLF--TQ--

-----QCDY----T--DI--M-SA-LHTFNLSNNK-------------L--T-SL-SSFA

-SLAGEF--SN-LQVIDISH-N-Q--------------------------L-G---FK--

--------GN------------S--PCS-W----------K-----Q-------------

--NITTVIANHNS--M----V-ID--------------SFRCLPT---------------

TVLYL----------DLS-------YCNL----DQ--L-----DVN-----YF--R-KAI

----------------------------------------------------------N-

---------------------LKEVYLSGNKIKFI-PSDW--KS-PSLKSLALDGNSFGI

ISMR---------S-FK------------GMPN-------------LEQLRAG-NNPYYC

TCEL--H-NF----I--Q--------------E----T-------AI----------KGN

-------V-----------NI--------TD-W-----PENYRC-YHP-ED----LL---

-NTMVSHY--LPGQIV-C-DIRL-----VIATS--VVTTAGAVLALMLI----C--YFFN

I-PW-YAKAT---YQ---I-----LR----A--K------------------Y------R

S-H---K----E-GL---------------------KL----S---MDY---T----FHA

FISYSHSDA---DWVRDELLPCL----ENC---K--------------PP----------

---Y---RLCIHERDFM----------------P--GK-WIIDNII-ENIENSWKVIFVL

SR------HFV-----------NSE-------WCNY-ELYFAQQRAMG--MGK-TSDVIL

VVKE--P-IDPNSL----------------------------------------------

--PSKYCKLKK-MLSNKTYLEWP----EQ-------PKHQA-------FFWEQLRSVM--

--GKP------------KL-----------------------------------------

-------------------T-RQF-S-------SS-----MRRCSTRMN-----TVSVIE

LVHENQKSADSGPKI----DEASEIKLQIEGKA--------------------------

>TLR18_Eslu

------------------------------------------------------------

------------------------------------------------------------

------------------------------------------------------------

------------------------------------------------------------

------------------------------------------------------------

------------------------------------------------------------

---MHIGPFFITKSKRYM------H--IMI--L---------------------------

--------------------------------L---G--F--A--W------FV----L-

---PH---HSIY-----------------A---LQT--------ASNDVRNPC--QI---

--YD-S------------------------------GRFAKCL-----------------

-----------G------QQ--LD-H-------VP----W-N-LL-----P-------S-

T--LESL-------DLSYN-------E---I--Q-----------AIYADDFSR--L-HS

L-----------RVLELQ-FNN------------------I-SV--IED--HAFHNNSLL

ENLNIF------N--------NS-L-EE-----------------------VP---A-TA

------L-QPL---YN---LRRLFMS-NN-----L-YTKAK------LA---D---KV--

----FS-SFT------------H-------------------------------------

-------------LQILSMG-G-P-LVKGLR----------------KGDFQALK----N

ISLQEFNIKCSS--NI--SY-YE----PG--S------------LKA----------IQT

SS-MGFD-----IA--------IDQKPHF-------L----------------PLML---

-QDL--AS----K----TFS---------------QIQF----RNLF----E-----F--

M--YY---M-Q-D---DDI-------FR----NLPD-------I--NV--HQLIF----H

RGK----------------FNENL---LR-M----------A-LLNLQV----T-PI-K-

-HL---------TFQY---------------------ID---------F--ARS-----P

------------N----F-----V--D-------S--GL-NSS----------I-----K

DL----A------------LDR---LDLWHISN-PDI-----------------------

-------------------------------------------------LRF---DWR--

-------------------------------FTWFN------------------------

---------------------------K--IRL-------LSIQNVNFNSV-PCDA--WL

EMGG--MEVLDISNNR--------L-Q--------N--------------NFIF--NQ--

-----RCNY----R--GN--M-PA-LFIFNVSTNQ-------------L--T-SL-RDLA

-SLTSKF--KQ-LQVMDVSN-N-L--------------------------L-G---SAK-

--------ES------------H--NCY-W----------L-----H-------------

--NITQLIAHHNH--F----E-SG--------------ALKCLPT---------------

TVQFL----------DLS-------YCDL----DQ--L-----DMA-----YF--Q-QTT

----------------------------------------------------------T-

---------------------LKELWLNGNKIKFI-PSGW--KS-HSLQSLALDGNSFGI

ISIG---------S-FQ------------DMPK-------------LSRLTAG-NNPYHC

TCDL--H-AF----I--Q--------------D----T-------IS----------KRR

-------I-----------NI--------TD-W-----PKNYKC-YHP-EA----LL---

-NTAVAHF--FPGNVA-C-DIRL-----VIVIC--VATTAVVVLVLVFI----C--YIFN

I-PW-YTKAT---YQ---I-----LR----A--K------------------Y------R

A-H---Q----Q-GAY--------------------GG----S---QVY---E----YHA

FISYSHPDA---DWVRDQLLPCL----ENN---K--------------PP----------

---Y---RLCIHERDFM----------------P--GK-WIIDNII-ENIESSRKVIFVL

SH------HFV-----------NSE-------WCNY-ELYFAQQRAIG--K-T-FNDVIL

VVKE--P-IDPESL----------------------------------------------

--PSKYCKLKK-MLNTKTYLEWP----QQ-------DKQQT-------FFWAQLKSVL--

--GKP------------TL-----------------------------------------

-------------------I-RQHAA-------SG-----RGSMVSSVE-----TVSVIE

LSLEERIPAKYMPKLKSEAELPKVDPEDAVFL---------------------------

>TLR18.1_Sasa

------------------------------------------------------------

------------------------------------------------------------

------------------------------------------------------------

------------------------------------------------------------

------------------------------------------------------------

------------------------------------------------------------

---------------MCM------H--TMI--L---------------------------

--------------------------------L---G--F--V--W------FS----P-

---PL---LVIL-----------------A---SPTP-------ASNEKRGPC--QI---

--YN-S------------------------------GRSANCL-----------------

-----------G------QR--LD-H-------VP----W-K-HL-----P-------S-

T--LERI-------DLSYN-------E---L--H-----------AIRVNYFSR--L-PH

L-----------RVLELQ-FNN------------------I-SV--IED--RAFHNNPLL

EHLNIF------N--------NS-L-EE-----------------------IP---A-NA

------L-QDL---FN---LRELLMS-NN-----L-YTQAT------LS---A---DV--

----FS-RLT------------H-------------------------------------

-------------LKALSMG-G-P-MVKGLR----------------KGDFQALR----N

IQLNDFAIKTSS--NI--SY-YE----PG--S------------LKV----------IQT

GR-MGFD-----MA--------IDQKPNF-------L----------------PLIL---

-RDL--AN----K----TFS---------------LIQF----RNLF----E-----F--

M--YY---L-G-D---EDI-------FR----GLQD-------I--KV--DSLIF----H

RGK----------------FNENL---MR-M----------A-LLNLQV----T-PL-K-

-RL---------TLQY---------------------ID---------F--ARS-----P

------------N----F-----V--D-------N--GL-ASS----------I-----K

DL----A------------LSR---LNLWHISN-PDI-----------------------

-------------------------------------------------MRF---DWR--

-------------------------------FTWFN------------------------

---------------------------K--VRL-------LSIQNVNFNSV-PCDA--WL

NMED--MEVLDISNNR--------L-P--------D--------------NILF--NQ--

-----RCDY----R--GT--M-PA-LRSFNVSTNQ-------------L--T-SL-QDLA

-SLTREF--KQ-LKVLDVSH-N-M--------------------------L-G---SAE-

--------ES------------R--NCN-W----------T-----Q-------------

--NITQVIAHHNR--F----K-SG--------------ALQCLPT---------------

TVQFL----------DLS-------YCDL----DQ--L-----DMA-----YF--R-QTI

----------------------------------------------------------S-

---------------------LKELLLNGNKIKFI-PSGW--RS-RSLQSLALDGNSFGL

ISMG---------Y-FQ------------DMPQ-------------LSRLTAG-NNPFHC

TCDL--H-AF----I--Q--------------E----T-------IS----------KGK

-------V-----------NI--------TD-W-----PENYKC-YHP-EA----QL---

-NTAVANF--FPGHVA-C-DVRL-----VIIIC--VATTAAVVLVLMLI----C--YIFN

V-PW-YTKAT---YQ---I-----LR----A--K------------------Y------R

A-H---Q----E-GTS--------------------GK----S---QIY---A----YHA

FISYSHPDA---DWVRDQLLPCL----ENS---K--------------PP----------

---Y---RLCIHERDFM----------------P--GK-WIIDNII-ENIESSEKVIFVL

SH------HFV-----------NSD-------WCNY-ELYFAQQRAIG--K-T-FSDVIL

VVKE--P-IDPESL----------------------------------------------

--PSKYCKLKK-MLNTKTYLEWP----QQ-------VTQQT-------FFWAQLRSCL--

--GKP------------TV-----------------------------------------

-------------------T-REQAL-------SG-----RSSSVSSVG-----VVPVIE

LPLEDGKPAIAMPNLDREAGLHKVVPQEIKNLSARSAEFYGQRPIPVAVA---------

>TLR18.2_Sasa

------------------------------------------------------------

------------------------------------------------------------

------------------------------------------------------------

------------------------------------------------------------

------------------------------------------------------------

------------------------------------------------------------

-------------MCMHT------M--ILL--G---------FV----------------

-------------------------------WF---SPPL--L--V------AL----A-

--------------------------------------------SPTPASNDE--RG---

-PCQ-I---------------------------YNSGRSANCL-----------------

-----------G------QR--LD-H-------VP----W-K-HF-----P-------S-

T--LESI-------DLSYN-------E---I--H-----------AIRVNYFSR--L-PH

L-----------RVLKLQ-FNN------------------I-SV--IED--RAFHNNPLL

EHLNIF------N--------NS-L-EE-----------------------IP---A-NA

------L-QDL---FN---LRELLMS-NN-----L-YTQAT------LS---A---EV--

----FS-RLT------------Q-------------------------------------

-------------LKALSMG-G-P-LVKGLR----------------KGDFQALR----N

IQLQDFAIKTSS--NI--SY-YE----PG-----------------S---LKV----IQT

GR-MGFD-----MA--------IDQKPNFL-----------------------PLIL---

-RDL--AN----K----TFS---------------LIQF----RNLF----------E--

F--MY---Y-M-G---DED-------IFR---GLQD-------I--KV--DSLIF----H

RGK------------FNEKLMRMA---LF------------N-LQVTPL--KSL-TL-Q-

-YI---------DFAR---------------------SP---N-----F--VDN-----G

-----------------L-G---S--S-----------I-KDL-----------------

------A------------LSR---LNLWHISN---------------------------

-------------------------------------------------PDILRFDWR--

-------------------------------FTWFN------------------------

---------------------------K--VRL-------LSIQNVNFNSV-PCDA--WL

NMED--MEVLDISNNR--------L-Q-D------N------ILFNQ-R-CDYR------

----------------GT--M-PT-LRSFNVSTNQ-------------L--T-SL-QDLA

-SLTREF--KQ-LKVLDVSH-N-M--------------------------L-G---SA--

--------EE----------S-R--NCN-W----------M-----Q-------------

--NITQVIAHHNRFKS----G-----------------ALQCLPT---------------

TVQFL----------DLS-------YCDL----DQ--L-----DMA-----YF--QQTIS

------------------------------------------------------------

---------------------LKELLLSENKIKFI-PSGWRS---HSLQSLALDGNSFGL

ISMG---------S-FQ------------DMPQ-------------LSRLTAG-NNPFHC

TCDL--H-AF----L--Q--------------D----T-------IS-------------

-------K-GKV-------NI--------TD-W-----PENYKC-YHP-EA----LL---

-NTAVANF--FPGHVA-C-DIRL-----IIIIC--VATTAAVVLVLMLI----C--YIFN

V-PW-YIKAT---YQ---I-----LR----A--K--------------------------

--Y-RAH----Q-EGA---------------S----GK----S---QIY---V----YHA

FISYSHPDA---DWVRDQLLPCL----ENS---K--------------PP----------

-Y-----RLCIHERDFM----------------P--GK-WIIDNII-ENIESSEKVIFVL

SH------HFV-----------NSD-------WCNY-ELYFAQQRAIG--K-T-FSDVIL

VVKE--P-IDPESL----------------------------------------------

--PSKYCKLKK-MLNTKTYLEWP----QQ-------AKQQT-------FFWAQLRSCL--

--GKP------------TV-----T-------REQALSGRSSSVSSVGAVPVIELPLEDG

KPAIAMPNLDREAELHKVVPQEIEKL-------SV-----RSPEFYGQRPIPVALASDKI

SQACSL-----------------------------------------------------

>TLR18_Pema

------------------------------------------------------------

------------------------------------------------------------

------------------------------------------------------------

------------------------------------------------------------

------------------------------------------------------------

------------------------------------------------------------

-----------------------------M--I---------------------------

--------------------------------W---T--A--F--L------LC----A-

---LF---SRTR-----------------T---SPTP------PPSALTSSPC--LI---

--SS-S------------------------------GLFADCL-----------------

-----------G------QQ--LD-S-------VP----W-H-QL-----P-------T-

T--LETI-------DLSYN-------K---L--T-----------AIHAEDFSQ--F-PN

L-----------RVLLLT-FNN------------------I-SH--IDD--NAFLHNPIL

ENLNIF------N--------NS-L-HE-----------------------IP---A-KA

------L-QPL---SN---LKILFMS-NN-----L-YKSAK------LA---E---S---

----FA-NFA------------K-------------------------------------

-------------LQVLSLG-G-H-LVMGLK----------------KGDFEPLR----N

ISLQTFAIRCSS--NL--SY-YE----RG--S------------LEV----------IQT

KQ-MGFD-----MA--------IDTQPNA-------L----------------LDML---

-GDL--AN----K----SFT---------------DVKF----RNLF----E-----F--

T--YY---T-G-V---EDI-------FQ----GLVN-------V--TA--HRLIF----Y

RGK----------------FNENL---LR-M----------L------Q----T-PI-K-

-RL---------RLQY---------------------ID---------F--ARS-----P

------------S----F-----S--D-------S--GA-ESS----------I-----T

DL----K------------LDR---LDLWYISN-PDI-----------------------

-------------------------------------------------LRF---DWR--

-------------------------------FTWLN------------------------

---------------------------G--IRQ-------LSIQHVYLNFA-PCDA--WL

EMRD--VEILDVSNNR--------L-R--------D--------------EYIY--NR--

-----RCVY----K--GT--L-PN-LRTFNLTNNE-------------L--T-SL-RDLS

-SLTRYF--NR-MVVMDVSN-N-K--------------------------L-G---SAA-

--------YS------------S--GCI-W----------H-----Q-------------

--NITRFIAHHNE--F----V-SE--------------ALLCLPT---------------

TVEYL----------DLS-------YCNL----DQ--L-----EMT-----FF--E-KAT

----------------------------------------------------------N-

---------------------LKELLLSGNKIKYI-PPSW--KS-PSLQSLELDGNSFGV

ITID---------S-FQ------------EMPL-------------LSSLKGG-NNPYHC

TCEL--H-AF----V--E--------------N----T-------MS----------KEK

-------V-----------NL--------TD-W-----PWNYHC-YHP-QP----LL---

-NTLISKY--FPGRVA-C-DIRL-----VILIC--VATTAVVIIILMVI----C--YIFD

L-PW-YTKAT---YQ---I-----IR----A--K------------------Y------R

A-Y---Q----E-STT--------------------EG----V---VLF---T----YHA

FISYSHSDA---EWVKEQLLPAL----EGS---K--------------DP----------

---Y---RLCIHERDFM----------------P--GK-WIIDNII-ENIENSRKVIFVL

SR------NFV-----------NSE-------WCNY-ELYFAQQRAMG--K-S-FTDVIL

VVKE--P-INPGSL----------------------------------------------

--PNKYCKLRK-MLSTKTYLEWP----QQ-------VNQQA-------FFWAQLRSVL--

--GRP------------SP-----------------------------------------

-------------------V-RSR-S-------FS-----LRSLSLRSR-----LRSEEN

RPLESNGGLSVAVVGEPRSTEQN------R-----GEEEVNQRQMQLPVA---------

>TLR18_Meae

------------------------------------------------------------

------------------------------------------------------------

------------------------------------------------------------

------------------------------------------------------------

------------------------------------------------------------

------------------------------------------------------------

-----------------------------M--F---------------------------

--------------------------------W---T--L--L--S------RS----V-

---LI---CGLV-----------------TSHSLTTP----TVPTTISKEGPC--RI---

--YN-S------------------------------GRSADCL-----------------

-----------G------RQ--LD-R-------IP----W-R-QF-----P-------Y-

T--LEKV-------DLSYN-------K---L--Q-----------AVYAEDFQN--L-PH

L-----------QVLQLQ-FNN------------------L-SH--IDE--DAFKHNPIL

ESLNIF------N--------NS-L-RE-----------------------IP---A-PA

------L-ISL---MN---LQKLDMS-NN-----L-YESAT------LV---E---G---

----FS-KLV------------K-------------------------------------

-------------LKVLSMG-G-P-LVMGLK----------------KGDFQPLK----N

IKLQGFAIKCTS--NL--SF-YE----LG--S------------LEV----------IQT

QQ-IGFD-----MA--------IDQRPQA-------L----------------PLML---

-SDI--AN----K----TFT---------------AIQF----RNLF----E-----F--

M--YF---S-G-K---EDI-------FF----NLRY-------V--KA--YQLIF----H

RGK----------------FNENL---LR-M----------A-LMNIQE----A-NIVK-

-RL---------RLQY---------------------ID---------F--ARS-----P

------------T----F-----E--D-------S--GA-GSS----------I-----T

NL----K------------LDG---LDLWYISN-PDV-----------------------

-------------------------------------------------LRF---DWR--

-------------------------------FTWFN------------------------

---------------------------K--VKQ-------LSLQYVYFNSV-PCDA--WA

EMGS--AEMLDVSNNR--------L-L--------D--------------SVLY--NK--

-----RCDY----T--GS--V-PH-VRIFNVSTNS-------------L--V-SL-KEMS

-YLTQEF--KR-LEVLDLSH-N-M--------------------------L-G---SLE-

--------GS------------R--DCV-W----------K-----P-------------

--NITRLIAHHNQ--F----V-SE--------------ALQCLPT---------------

TMQFL----------DLS-------SCDL----GQ--L-----DMT-----FF--D-QTT

----------------------------------------------------------Q-

---------------------LTELLLSGNKIKFI-PPHW--RS-AYLRSLALDGNSFGV

ISVE---------S-FQ------------EMPL-------------LSQLSAG-NNPYHC

TCEL--H-AF----I--Q--------------Q----T-------QS----------QGK

-------V-----------NL--------ME-W-----PENYKC-YHP-EP----FL---

-NTIIANY--LPAHVA-C-DIRL-----VVVIS--VAVTATAVMILMLI----C--YIFN

I-PW-YAKAT---YQ---I-----VR----A--K------------------Y------R

A-H---K----E-RAA--------------------GE----G---EVF---V----YHA

FISYCHSDG---DWVRDQLLPCL----ENN---R--------------DP----------

---Y---RLCIHERDFM----------------P--GK-WIIDNII-DNIENSRKVIFVL

SR------HFV-----------NSE-------WCNY-ELYFAQQRAMG--K-T-FSDVIL

VVME--P-IDPHSL----------------------------------------------

--PSKYCKLKK-MLSTKTYLEWP----QQ-------SKHQP-------FFWAQLKSVL--

--GKP------------SL-----------------------------------------

-------------------T-RGR-A----------------------------------

-----------------------------------------------------------

>TLR18_Taru

------------------------------------------------------------

------------------------------------------------------------

------------------------------------------------------------

------------------------------------------------------------

------------------------------------------------------------

------------------------------------------------------------

-----------------------------M--I---------------------------

--------------------------------W---T--F--V--H------FI----A-

---LA---VGVL-----------------A---STTP---SPPSTTKTVTGFC--RV---

--FN-S------------------------------GRSADCL-----------------

-----------G------MQ--LS-S-------VP----W-R-QF-----P-------P-

S--LEDI-------DLSYN-------R---L--Q-----------VINAEDFAL--F-PR

L-----------RSLNLK-YNN------------------I-SR--IDS--DAFKNNPLL

EILDIF------N--------NS-L-GE-----------------------IP---V-AA

------L-SPL---LN---LKKLYMS-NN-----L-YKRAA------LA---E---T---

----FS-TFV------------R-------------------------------------

-------------LQTLSMG-G-P-LVEGLK----------------KGDFQPLR----K

LRLQEFAIKCSS--NL--RY-YE----AG--S------------LEV----------VQT

QK-LGFD-----MA--------IDQRPSA-------L----------------VDML---

-RDI--AN----K----TFI---------------AIQF----RNLF----E-----F--

R--YY---T-R-V---QDI-------FQ----GLKH-------V--AA--YQLIF----H

RGK----------------FNENL---LR-M----------A-LLNLE-------AI-K-

-RL---------RFQY---------------------ID---------F--ARS-----P

------------T----F-----V--D-------N--RA-GSS----------I-----T

DL----V------------LDK---LDLWYISN-PDV-----------------------

-------------------------------------------------LRF---DWR--

-------------------------------FTWFN------------------------

---------------------------N--IRS-------LSIQYVYFNSV-PCDS--WA

EMKQ--VKVLDVSNNR--------L-T--------D--------------TYIF--NQ--

-----LCNY----K--GA--A-PN-LRLFNMSNNE-------------L--T-SL-KDLS

-LLTKEF--QQ-LQELDLSR-N-K--------------------------L-G---SAA-

--------ES------------R--NCI-W----------Q-----K-------------

--SITRFIVHHNN--F----E-SS--------------ALHCLPT---------------

SVEFL----------DLS-------FCDL----DQ--L-----DMN-----YF--S-KTS

----------------------------------------------------------N-

---------------------LQELHLSGNKIKFI-PSKW--AS-PSLQSLSLDGNSFGL

IGTE---------S-FQ------------DMPR-------------LSHLSAG-NNPYHC

TCEL--H-AF----V--Q--------------E----T-------IT----------EGK

-------V-----------NL--------TD-W-----PWNYKC-YHP-EP----LL---

-NTVISQY--LPGKVA-C-DIRL-----VIVIC--VAATTFVVLILVLI----C--YIFD

L-PW-YTKAT---FQ---I-----IR----A--K------------------Y------R

A-H---K----E-KAA--------------------GE----E---GPF---T----YHA

FISYSHSDA---DWVRDQLLPCL----ENN---N--------------NP----------

---Y---RLCIHERDFT----------------P--GR-WIIDNII-ENIENSRKVIFVL

SR------HFV-----------NSE-------WCNY-ELYFAQQRAMG--K-T-FSDVIL

VVKE--P-IDPNSL----------------------------------------------

--PSKYCKLKK-MLSTKTYLEWP----QQ-------VNQQA-------FFWAQLRSVL--

--GRP------------TA-----------------------------------------

-------------------V-TRG-R-------QS-----VRSRTSSA------SISVIG

PLVDERNPEM---DEDRGTEPNY------EVIE-NSLEVSHQRQIPMVAV---------

>TLR18_Hico

------------------------------------------------------------

------------------------------------------------------------

------------------------------------------------------------

------------------------------------------------------------

------------------------------------------------------------

------------------------------------------------------------

-----------------------------M--L---------------------------

--------------------------------W---S--F--I--V------LG----A-

---VF---NAGH-----------------T---SPTQR--PSTRSGAMEGRPC--RF---

--SN-A------------------------------GRTADCL-----------------

-----------G------GQ--LE-S-------VP----W-R-YF-----P-------S-

T--LEDI-------DLSYN-------K---L--Q-----------EIHSEDFRR--L-PR

L-----------RVLNLQ-YNN------------------I-SR--IDD--AAFGNNNLL

EHLNIF------N--------NS-L-KE-----------------------IP---A-SV

------L-TSL---LN---LKYLYMS-NN-----L-YTRAT------LS---E---G---

----FS-RLT------------K-------------------------------------

-------------LKVLSLG-G-P-LVEGLK----------------REDFRPLM----K

IKLQGFAIKCST--NL--SY-YE----PG--S------------LRI----------VQT

EQ-MGFD-----VA--------IDQRPGT-------L----------------LHML---

-QDL--AN----K----SFS---------------ALQF----RNLF----E-----F--

T--YY---T-G-D---EDI-------FQ----GLGD-------I--TV--YQLVF----H

RGK----------------FNENL---LR-M----------A-LTNLQV----A-PI-K-

-RV---------RFQY---------------------ID---------F--ARS-----Q

------------K----F-----T--D-------S--GA-KSS----------I-----T

DL----K------------LEK---LDLWYISN-PDI-----------------------

-------------------------------------------------LRF---DWR--

-------------------------------FTWFN------------------------

---------------------------K--VKQ-------LSIGYVYFNSV-PCDA--WV

EMSG--VEILDVSNNR--------L-R--------D--------------TYIF--NQ--

-----LCDY----R--GS--M-TS-LHTFNMSTNE-------------L--T-SL-KDLS

-ALTRQF--HR-LQVLDFSH-N-Q--------------------------L-G---SVE-

--------SS------------R--GCI-W----------Q-----T-------------

--NITTVIAHHNQ--F----T-TE--------------ALACLPT---------------

TVHFL----------DLS-------SCNL----DQ--L-----NVT-----YF--E-KAI

----------------------------------------------------------N-

---------------------LKELLLSDNKIKFI-PSRW--ES-PSLFRLALDGNSFGL

ISKE---------S-FQ------------HMPG-------------LSHLRAG-NNPYHC

TCEL--H-AF----I--E--------------D----T-------------------KGR

-------V-----------NL--------TD-W-----PENYKC-YHP-EQ----FL---

-NTVISKY--LPSEVA-C-DVRL-----VIIIS--VATTTAVLLILMLI----C--YIFD

L-PW-YTKAT---YQ---I-----IR----A--K------------------Y------R

A-H---K----E-RAA--------------------GE----A---GPF---A----YHA

FISYSHWDA---DWVREQLLPCL----ENN---R--------------NP----------

---Y---RLCIHERDFM----------------P--GR-WIIDNII-ENIESSHKVIFVL

SR------HFV-----------NSE-------WCNY-ELYFAQQRAMG--K-T-FSDVIL

VVKE--P-IDPGSL----------------------------------------------

--PSKYCKLKK-MLSTKTYLEWP----QQ-------ANQQA-------FFWAQLRSVL--

--GKP------------T------------------------------------------

-------------------------R-------HS-----TRSRNSSIG-----GHSL--

--KEPTGPEMADPDGGAEYV---------------KDEMFNGRQIPVVA----------

>TLR18_Orla

------------------------------------------------------------

------------------------------------------------------------

------------------------------------------------------------

------------------------------------------------------------

------------------------------------------------------------

------------------------------------------------------------

-----------------------------M--I---------------------------

--------------------------------C---A--V--L--M------FS----G-

---LL---MGTL-----------------S---SPT----VFPTSSSTEKKPC--RI---

--YN-N------------------------------GRSADCR-----------------

-----------G------QQ--LY-R-------VP----P-R-HL-----P-------P-

T--LEDI-------DLSYN-------E---I--N-----------AVHASDFQH--L-PR

L-----------QTLQLQ-YNN------------------I-SH--IDN--NAFQNNTLL

EHLDIF------N--------NS-L-LE-----------------------IP---T-VA

------L-EPL---FN---LKKLFMS-NN-----L-YKHAT------LA---D---S---

----FS-KFV------------K-------------------------------------

-------------LQILSMG-G-P-LVMGLK----------------KKDFYPLK----S

IKLHTFAIKCSS--NL--SY-YE----PE--S------------LKV----------VQT

TI-MGFD-----MA--------VDQLPSA-------L----------------HNML---

-HDL--AN----K----SFQ---------------VIQF----RNLF----E-----F--

T--YY---L-G-K---DDI-------FQ----GLRN-------V--KS--QQLIF----H

RGK----------------FNENL---LR-M----------A-LMNLQV----T-PI-K-

-RL---------RLQY---------------------ID---------F--ARS-----P

------------T----F-----V--D-------S--GV-GSS----------I-----T

NL----A------------LDN---LDLWYISN-PDI-----------------------

-------------------------------------------------LRF---DWR--

-------------------------------FTWFK------------------------

---------------------------K--IKH-------LSIQHVYFNSV-PCDS--WS

EMEG--LEILDVSNNR--------L-Q--------N--------------DYVF--NQ--

-----RCDY----S--GT--M-PN-LHTFNMSTND-------------L--T-SL-KDLS

-SLTREF--SR-MQVFDLSN-N-K--------------------------L-G---STQ-

--------ER------------E--DCI-W----------K-----Q-------------

--NITHFIAHHNP--L----G-SN--------------AFNCLPT---------------

TVHYL----------DLS-------YCNL----DQ--L-----EMT-----YF--R-KAT

----------------------------------------------------------N-

---------------------LKHLLLSGNKIKFI-PSKW--ES-LSLQFLALDGNSFGL

ISKE---------S-FQ------------HMPQ-------------LSELSAG-NNPYHC

TCEL--H-AF----V--E--------------D----T-------LS----------EGK

-------I-----------NL--------TD-W-----PLNYRC-YHP-EP----FL---

-NTFISKY--FPGKVA-C-DIRL-----VITIS--VVVTAAVIMVLVLI----C--YIFD

L-PW-YTKAT---YQ---I-----IR----A--K------------------Y------R

A-H---K----E-KAV--------------------GE----M---ETF---T----YHA

FISYSHSDA---DWVRDQLLPCL----ENN---R--------------NP----------

---Y---RLCIHERDFM----------------P--GK-WIIDNII-ENIESSRKIIFVL

SR------HFV-----------NSE-------WCNY-ELYFAQQRAMG--K-T-FSDVIL

VVKE--P-IDPSSL----------------------------------------------

--PSKYCKLKK-MLSTKTYLEWP----QQ-------PNQQQ-------FFWAQLRSVL--

--GKP------------TE-----------------------------------------

-------------------T-QDR-P-------HS-----IRKRNSE-G-----RISVIG

PPVEKNMPKEDMPDADSK---DL------EFDEGNGIELSNLRQIPIMVL---------

>TLR18_Cyro

------------------------------------------------------------

------------------------------------------------------------

------------------------------------------------------------

------------------------------------------------------------

------------------------------------------------------------

------------------------------------------------------------

-----------------------------M--F---------------------------

--------------------------------L---K--L--M--S------LS----T-

---LM---FGLA-----------------SPSPSTTP---SPFTPNSTEGGLC--RI---

--SN-L------------------------------GRSADCL-----------------

-----------G------RQ--LD-S-------VP----W-R-HL-----P-------S-

T--LENI-------DLSYN-------R---L--Q-----------AISAVDFHH--L-PR

L-----------QILELQ-YNN------------------I-SH--IDN--DAFKNNPFL

EHLNIF------N--------NS-L-QE-----------------------IP---A-AA

------L-MPL---VN---LKILYMS-NN-----L-YKHAT------LA---E---G---

----FS-KFD------------K-------------------------------------

-------------LKVLSMG-G-S-LVMGLK----------------KGDFQPLK----N

IKLQGFAIKCSS--NL--SY-YE----PG--S------------LEV----------IQT

SQ-IGFD-----IA--------IDQRPNA-------L----------------PLLL---

-RDI--AN----K----TFS---------------VIQF----RNLF----E-----F--

M--YY---M-G-D---EDI-------FQ----GLKY-------V--TA--YQLIF----H

RGK----------------FNENL---LR-M----------T-LMNLEE----A-QI-R-

-NL---------RLQY---------------------ID---------F--ARS-----P

------------T----F-----V--D-------S--GA-GSS----------I-----T

NL----A------------LER---LDLWYISN-PDV-----------------------

-------------------------------------------------LRF---DWR--

-------------------------------FTWLN------------------------

---------------------------Q--IQK-------LSIQYVYFTSV-PCDA--WV

EMGG--VEVLDASNNR--------L-V--------D--------------SVLF--NK--

-----RCNY----K--GT--M-QN-LHTFNLSTNE-------------L--T-SL-KDTS

-YLTKEF--QQ-LQVLDLSN-N-K--------------------------L-G---SAK-

--------GS------------R--DCV-W----------R-----Q-------------

--NITKLIAHHNE--F----T-TE--------------ALQCLPT---------------

TVRVL----------DLS-------YCEL----DQ--L-----DMT-----YF--E-KSL

----------------------------------------------------------N-

---------------------LKELLLSGNKIKFI-PSRW--RS-ASLQSLALDGNSFGV

ISVE---------S-FR------------DMPQ-------------LSRLSAG-NNPYHC

TCEL--H-AF----I--Q--------------D----T-------LS----------KGK

-------V-----------NL--------TE-W-----PEDYRC-YHP-QP----FL---

-NSVISNY--FPGRVA-C-DIRL-----VILIS--VATTAVVVMILMLL----C--YIFN

I-PW-YTKAM---YQ---I-----VR----A--K------------------Y------R

A-H---K----E-NAA--------------------GE----A---QIF---A----YHA

FISYSHSDA---DWVRDQLLPCL----END---R--------------DP----------

---Y---RLCIHERDFM----------------P--GK-WIIDNII-ENIESSRKVMFVL

SR------NFV-----------NSE-------WCNY-ELYFAQQRAMG--K-T-FSDVIL

VVKE--P-IDPDSL----------------------------------------------

--PSKYCKLKK-MLNTKTYLEWP----QQ-------PNQQV-------FFWAQLKSVL--

--GKP------------SL-----------------------------------------

-------------------T-GGR-M-------GS-----IRSAKSNR------------

-----------------------------------------------------------

>TLR18_Poja

------------------------------------------------------------

------------------------------------------------------------

------------------------------------------------------------

------------------------------------------------------------

------------------------------------------------------------

------------------------------------------------------------

-----------------------------M--I---------------------------

--------------------------------W---K--L--M--N------LS----A-

---LL---AGTL-----------------ASPTTPTP---SSSPSAVTEEHVC--RI---

--YN-L------------------------------GRSADCL-----------------

-----------G------RQ--LD-Y-------IP----W-R-QF-----P-------S-

T--LEDI-------DLSYN-------K---L--Q-----------AVRADDLLN--L-PE

L-----------RILQLQ-FNN------------------I-SY--IDD--DAFKQNLLL

EHLDIF------N--------NS-L-QE-----------------------IP---A-TA

------L-RPL---SN---LQKLYMS-NN-----L-YKHAT------LA---D---G---

----FS-KFN------------K-------------------------------------

-------------LQVLSMG-G-P-LVMGLK----------------KDDFLPLK----N

IRLHGFAIKCSS--NL--SY-YE----PG--S------------LAV----------IQT

SQ-MGFD-----IA--------IDQRPNS-------L----------------PLML---

-RDL--AN----K----TFS---------------VIQF----RRLF----E-----F--

M--YY---L-G-D---EDI-------FQ----GLKD-------I--SA--SQLIF----H

RGK----------------FNENL---MR-M----------A-LMNLQI----A-PI-K-

-RV---------RLQY---------------------ID---------F--TRS-----P

------------T----F-----V--D-------S--GS-GSS----------I-----T

DL----E------------LDK---LDLWYISN-PDI-----------------------

-------------------------------------------------LRF---DWR--

-------------------------------FTWFN------------------------

---------------------------K--IKE-------LSIQYVFFNSV-PCDS--WA

EMGG--VELLDVSNNR--------L-L--------D--------------SFLF--NQ--

-----LCDY----K--GR--M-QR-LRVFNLSNNA-------------L--S-SL-KDIS

-SLTRDF--RQ-LRVIDVAN-N-K--------------------------L-G---SAE-

--------ES------------R--DCV-W----------T-----Q-------------

--NITRVIAHHNQ--F----V-SE--------------ALHCLPT---------------

TVQFL----------DLA-------YCGL----DQ--L-----DMT-----YF--E-KTT

----------------------------------------------------------D-

---------------------LKELLLNGNKIKFI-PSRW--KS-LSLQSLALDGNSFGL

ISME---------S-FQ------------DMPR-------------LTRLRAG-NNPYHC

TCDL--H-AF----I--Q--------------D----T-------TS----------KGK

-------V-----------NL--------TD-W-----PENYRC-YHP-EA----LL---

-NTVIYRY--LPSRVA-C-DIRL-----VIIIC--VATTALVLMILMLI----C--YIFD

V-PW-YTKAT---YQ---I-----VR----A--K------------------Y------R

A-H---K----E-RAA---------------G----GE----A---EVF---T----YHA

FISYSHSDA---DWVRDQLLPCL----ENN---K--------------NP----------

---Y---RLCIHERDFM----------------P--GK-WIIDNII-ENIESSQKVIFIL

SH------HFV-----------NSE-------WCNY-ELYFAQQRAMG--K-T-FNDVIL

VVKE--P-IDPGSL----------------------------------------------

--PSKYCKLKK-MLSTKTYLEWP----QQ-------VKQQA-------FFWAQLKSVL--

--GKP------------TL-----------------------------------------

-------------------T-RER-M-------DS-----TRSKASSVG-----AVSVIE

LPLEDGRPERAAADVDGEAELDE------VIER--NDELFNLKPIPVAVA---------

>TLR18_Labe

------------------------------------------------------------

------------------------------------------------------------

------------------------------------------------------------

------------------------------------------------------------

------------------------------------------------------------

------------------------------------------------------------

-----------------------------M--I---------------------------

--------------------------------W---K--W--I--Y------LT----A-

---LL---PGAL-----------------T---SPTP---PSRPNTTAVEGPC--RI---

--YN-S------------------------------GRSADCL-----------------

-----------G------RQ--LE-S-------VP----W-T-QF-----P-------P-

T--LEEI-------DLSSN-------K---L--Q-----------AILANDFLR--L-PR

L-----------RVLLLK-FNN------------------I-SH--IDS--EAFKNNKLL

EHLDIF------N--------NS-L-RE-----------------------IP---A-TA

------L-TPL---LN---LKQLMMS-NN-----L-YEHAT------LD---E---S---

----FS-KLV------------H-------------------------------------

-------------LKVLSMG-G-P-LVMGLK----------------KADFQPLK----N

MRLHGFAIKCSS--NL--SY-YE----PG--S------------LEV----------VQT

MQ-MGFD-----MA--------LDQRSVA-------L----------------HHML---

-RDL--AN----K----TFS---------------VIQF----RNLF----E-----F--

T--YY---M-G-T---DDI-------FQ----GLRY-------V--TA--QQLVF----H

RGK----------------FNENL---LR-M----------A-LMNLEV----A-PI-R-

-NL---------RLQY---------------------ID---------F--ARS-----P

------------T----F-----I--D-------N--GG-DSS----------I-----T

DL----A------------LDE---LDLWYISN-PDV-----------------------

-------------------------------------------------LRF---DWR--

-------------------------------FTWFN------------------------

---------------------------K--IKH-------LSIQYVNFNSV-PCDA--WA

EMAA--VEIMDISNNR--------L-Q--------D--------------DYIF--NQ--

-----RCQY----K--GA--M-PN-LHTFTLSTND-------------L--T-SL-RDLS

-FLTREF--QK-LQELDVSN-N-R--------------------------L-G---AAG-

--------KS------------R--DCV-W----------Q-----K-------------

--SITRFIAHHNR--F----E-SE--------------SLRCLPT---------------

TVQYL----------DLS-------HCDL----DQ--L-----DIT-----YF--E-KAN

----------------------------------------------------------S-

---------------------LKELLLSGNKIKFI-PFQW--GS-PSLQSLALDGNSFGV

ISKA---------S-FR------------DMPR-------------LSHLRAG-NNPFHC

TCEL--H-AF----V--E--------------D----T-------TS----------KGK

-------I-----------NL--------TD-W-----PENYKC-YHP-EA----FL---

-NTVVSKY--LPGRVA-C-DIRL-----VIIIC--VATTMLVIFILMLM----C--YMFD

L-PW-YTKAT---FQ---I-----IR----A--K------------------Y------R

A-H---K----E-KAA--------------------GE----A---GSF---T----YHA

FISYSHSDA---DWVRDQLLPCL----ESD---K--------------NP----------

---Y---RLCIHERDFM----------------P--GR-WIIDNII-DNIESSRKVIFVL

SR------HFV-----------NSE-------WCNY-ELYFAQQRAMG--K-T-FNDVIL

VVKE--P-LDP-SL----------------------------------------------

--PSKYCKLKK-MLSTKTYLEWP----QQ-------VSQQP-------FFWAQLKSVL--

--GKP------------TV-----------------------------------------

-------------------T-REG-R-------HS-----VRSIASSVI-----GSEVVD

RKAEEAQLN---------AEPKN------KINKMNNYELSNQTHIPAVAF---------

>TLR18_Xima

------------------------------------------------------------

------------------------------------------------------------

------------------------------------------------------------

------------------------------------------------------------

------------------------------------------------------------

------------------------------------------------------------

----------------MI-GE--LI--SV---G---------------------------

-------------------------------VLLGAVSSQ--F--SPDP---SS--PST-

----------------------------------------------------FISDE--G

-PCR-I------------------------YNF---GLSADCQ-----------------

-----------G------RK--LD-F-------VP----W-R-QL-----P-------V-

T--LESI-------DLSYN-------G---L--H-----------VISAADFQN--F-PE

L-----------RILLLQ-YNN------------------I-SH--IAN--ETFKNNPLL

EHLNIF------N--------NS-L-HE-----------------------IP---A-SP

------L-TSL---LN---LKELSMS-NN-----L-YRHAT------LA---D---S---

----FS-KLV------------R-------------------------------------

-------------LKVLSLG-G-P-LVMGLK----------------KNDFQPLK----N

IQLQAFAIKCSS--NL--SY-YE----PG--S------------LEV---IHT---KQ-M

GFDMAID-----QR---------------------------------PNAL--HHML---

-RDL--AN----K----TLS---------------VIQF----RNLF----------E--

F--TY---Y-MGD---EDI-------FL----NLKD-------I--RA--FQLIF----H

RGK---------------FNENLL---KM------------A-LLNLQV----T-PI-K-

-RL---------RLQY---------------------ID---F---ARS--PTF-----V

------------D----S-G---A--G-------S--SI-TKL-----------------

S------------------LDN---LDLWYISN-PDV-----------------------

-------------------------------------------------LRF---DWR--

-------------------------------FTWFK------------------------

---------------------------R--IKQ-------LSIQYVYFSSV-PCDS--WA

EMEG--VELLDVSNNR--------L-N-D------D------VFYNK-R-CNYK------

----------------GS--M-PN-LLTFNLSTNE-------------L--T-SL-EDLS

-SLIKHF--KK-LQVLDLSN-N-K--------------------------L-G---SA-E

--------ES------------R--HCV-W----------T-----Q-------------

--NITRFIAHHNY----FSSE-----------------TFHCLPT---------------

TVSYL----------DLS-------DCNM----DQ--L-----DLT-----YF--K-KAT

----------------------------------------------------------N-

---------------------LKILLLSGNKIKFI-PSKW--ES-PSLQFLALDGNSFGL

ISKA---------S-FH------------HMPQ-------------LSQLTAG-NNPYHC

TCEL--H-SF----VQDT--------------I------------AI-------------

-------G-----------KV----NL--TD-W-----PLNYRC-YHP-EP----FL---

-NTSISKY--FPGQVA-C-DIRL-----VIIIS--VATTAVVILIFVLI----C--YIFD

L-PW-YTRAT---YQ---I-----IQ----A-----------------------------

------K---YRAHKE----------------NLA-GG----V---GNF---N----YHA

FISYSHSDA---DWVRDQLLPCL----ENN--------------------KNP-------

-Y-----RLCIHERDFM----------------P--GK-WIIDNII-ENIESSRKVIFVL

SR------HFV-----------NSE-------WCNY-ELYFAQQRAMG--K-T-FSDVIL

VLKE--P-IDPSSL----------------------------------------------

--PSKYCKLKK-MLSTKTYLEWP----QQ-------VNQQP-------FFWAQLRSVL--

--GKP------------TM-------------------------------T-REG-TNSI

RSRNSSE-----------------------------------------------------

-----------------------------------------------------------

>TLR18_Pafr

------------------------------------------------------------

------------------------------------------------------------

------------------------------------------------------------

------------------------------------------------------------

------------------------------------------------------------

------------------------------------------------------------

----------------MI-WK--LV--SLS--T---------------------------

-------------------------------LLLGVLT-------SPTPTP-SS--PPT-

----------------------------------------------------TSTEA--G

-PCR-I------------------------YNS---GRSADCL-----------------

-----------G------KQ--LD-D-------VP----W-R-QL-----P-------F-

T--LQKI-------DLSYN-------K---L--Q-----------AVHANDFLH--L-PH

L-----------RVLLLP-FNN------------------I-SH--IDD--DAFKNTPLL

EHLDIF------N--------NS-L-QE-----------------------IP---A-TA

------L-KPL---SN---LKQLYMS-NN-----L-YEHAT------LA---G---D---

----FS-KFV------------Q-------------------------------------

-------------LQVLSMG-G-P-LVMGLK----------------RGDFQPLK----N

IKLQGFAIKCSS--NL--SY-YE----PG--S------------LEV---IQT---NQ-M

GFDMAID-----QR---------------------------------PDAL--PLML---

-RDL--AH----K----TFI---------------AIQF----RNLF----------E--

F--MY---Y-MGA---DDI-------FQ----GLRD-------I--TV--RQLIF----H

RGK---------------FNENLL---RM------------A-LTNLEN----T-KI-K-

-QL---------RLQY---------------------ID---F---ARS--PTF-----I

------------D----S-G---A--G-------S--SI-TDL-----------------

S------------------LDK---LELWYISN-PDV-----------------------

-------------------------------------------------LRF---DWR--

-------------------------------FTWFN------------------------

---------------------------K--IRH-------LSIKYVYFNSV-PCDA--WV

EMRG--VEFLDVSNNR--------L-E-D------S------VLYNK-L-CSYK------

----------------GT--M-PV-LRTFNLSTND-------------L--A-SL-KDLS

-SLTEEF--QQ-LQVLDISN-N-K--------------------------L-G---SA-E

--------KS------------R--DCI-W----------R-----Q-------------

--NITRVIAHHNQ----FVSE-----------------SLQCLPT---------------

TMLYL----------DLS-------NCDL----DQ--L-----DMS-----YF--Q-KTT

----------------------------------------------------------N-

---------------------LKELLLSGNKIKFI-PSQW--KS-PSLQLLALDGNSFGL

ISMQ---------S-FQ------------DMPQ-------------LSRLRAG-NNPYHC

TCDL--H-IF----IQDT--------------M------------SK-------------

-------G-----------KV----NL--TD-W-----PESYKC-YHP-EA----FL---

-NTVISKY--LPSRVA-C-DIRL-----VIIIS--VTTTAAVVLILMLV----C--YLCD

V-PW-YAKAT---YQ---I-----IR----A-----------------------------

------K---YRAHKE----------------KVA-GE----P---EIF---A----YHA

FISYSHSDA---DWVRDQLLPCL----ENN--------------------RNP-------

-Y-----RLCIHERDFM----------------P--GK-WIIDNII-ENIESSRKIIFVL

SR------HFV-----------NSE-------WCNY-ELYFAQQRAMG--K-T-FSDVIL

VVKE--P-IDPQSL----------------------------------------------

--PSKYCKLKK-MLSTKTYLEWP----QQ-------VKQQA-------FFWAQLKSVL--

--GKP------------TL-------------------------------T-RER-AHSA

TSNISSVG-----AVSVIELPLEDKN-------PE-----IAMTKTAGEAEVIQRNAEL-

------YRPILVADT--------------------------------------------

>TLR18_Noco

------------------------------------------------------------

------------------------------------------------------------

------------------------------------------------------------

------------------------------------------------------------

------------------------------------------------------------

------------------------------------------------------------

----------------MM-WR--WV--HLS--A---------------------------

-------------------------------LLLGALA-------SPTP---SS--QRT-

----------------------------------------------------TNADG--E

-SCR-I------------------------YRS---GRSADCL-----------------

-----------G------RQ--YE-N-------IP----W-R-QF-----P-------S-

T--LEEI-------DLSYN-------K---L--Q-----------AVRVDDFLR--L-PQ

L-----------HTLELQ-YNN------------------I-SH--IDK--DAFKNNMLL

EHLNIF------N--------NS-L-EE-----------------------IP---A-AA

------L-STL---LN---LKKLYMS-NN-----F-YKSAA------LA---H---S---

----FS-KFV------------K-------------------------------------

-------------LKVLSLG-G-P-LVMGLK----------------KADFQPLK----N

ITLQEFAIKCSS--HL--SY-YE----PG--S------------LEV---IQT---RD-M

GFDMAID-----QL---------------------------------PRAL--PYML---

-QDI--AN----K----TFR---------------AIQF----RNIF----------E--

F--MY---Y-MGD---EDI-------FK----GLKD-------I--TA--QQLVF----H

RGK---------------FNENLL---SM------------A-LMNLQD----A-PI-K-

-RL---------RLQY---------------------ID---F---ARS--PTF-----V

------------D----S-G---A--G-------S--SI-NDL-----------------

A------------------LDK---LDLWYISN-PDI-----------------------

-------------------------------------------------LRF---DWS--

-------------------------------FTWFN------------------------

---------------------------K--VKE-------LSIQHVYFNSV-PCDA--WV

DMEV--VEILDASNNR--------L-N-N------E------FLFNK-R-CDYR------

----------------GA--V-PN-LHTFNMSRNV-------------L--T-SL-RDLS

-MLTKEF--KQ-LHVLDLSF-N-Q--------------------------M-G---SA-E

--------KS------------Q--DCV-W----------K-----I-------------

--NITRIIAHHNP----FVSE-----------------ALQCLPT---------------

TVQYL----------DLS-------YCDL----DQ--L-----DMM-----YF--E-KAT

----------------------------------------------------------N-

---------------------LKELLLSGNKIKYI-PSKW--KS-QSLQSLSLDGNSFGL

ISKA---------S-FQ------------DMPQ-------------LSQLRAG-NNPFHC

TCEL--H-AF----VQDA--------------I------------SK-------------

-------A-----------KV----NL--TD-W-----PWNYRC-YHP-EA----LL---

-NTVISKF--FPNQVA-C-DTRL-----VIIIC--VVTTAAVILILMLI----C--YIFD

L-PW-YTKAT---YQ---I-----IR----A-----------------------------

------K---YRYHKE----------------KAA-GE----L---EDC---T----YHA

FISYSHSDA---DWVREQLLPSL----ENN--------------------KNP-------

-Y-----RLCIHERDFM----------------P--GK-WIIDNII-DNIESSRKVIFVL

SR------HFV-----------NSE-------WCNY-ELYFTQQRAMG--K-T-FGDVIL

VMME--P-MDARSI----------------------------------------------

--PSKYCRLKK-MLSTKTYLEWP----QQ-------VNLQA-------FFWAQLRSVL--

--GKP------------TM-------------------------------T-REE-RHSV

KSRTLSVG-----EVSVIGPLIEDTR-------PE-----GATPNADKEAVPKDEIIKRN

NCELSNQRKIPPVAL--------------------------------------------

>TLR18_Myja

------------------------------------------------------------

------------------------------------------------------------

------------------------------------------------------------

------------------------------------------------------------

------------------------------------------------------------

------------------------------------------------------------

----------------MI-WR--LM--SFS--A---------------------------

-------------------------------LLAGALT-------STAP---AP--SPA-

----------------------------------------------------TNTEG--G

-PCR-I------------------------HNA---GRNADCL-----------------

-----------G------RQ--LD-H-------VP----W-A-QL-----P-------S-

T--LEEI-------DLSYN-------K---L--R-----------AVSVNDFRR--L-PQ

L-----------RVLQLQ-YNN------------------I-SH--IDD--DAFTYNPFL

EHLDIF------N--------NS-L-EE-----------------------IP---A-TA

------L-KPL---SN---LKKLLMS-NN-----L-YKNAT------LA---A---D---

----FS-KFT------------K-------------------------------------

-------------LQVLSMG-G-P-LVMGLK----------------KGDFDPLR----N

IKLQGFAIKCSS--NL--LY-YE----PG--S------------LKV---IQT---WN-M

GFDMAID-----QK---------------------------------PNTL--PHML---

-NDL--AN----K----TFS---------------AIQF----RNLF----------E--

F--MY---Y-TGD---EDI-------FQ----GLEN-------I--TA--HQLIF----H

RGK---------------FNENLL---RM------------A-LTNLQV----T-PI-K-

-RL---------TLQY---------------------ID---F---ARS--PTF-----V

------------D----S-G---A--G-------S--SI-TNL-----------------

E------------------LDK---LDLWYVAN-PDV-----------------------

-------------------------------------------------LRF---DWR--

-------------------------------FTWFN------------------------

---------------------------K--IRQ-------LSIRYVYFNSV-PCDA--WV

EMEG--VQVLDVSNNR--------L-E-N------S------FIFNQ-R-CEYK------

----------------GT--M-PN-LHTFNVSTNT-------------L--T-SL-KDLS

-ALTREF--QQ-LSVLDCSN-N-N--------------------------F-G---SA-K

--------ES------------R--DCV-W----------K-----Q-------------

--NISRVIAHHNQ----FVSE-----------------ALHCLPT---------------

TVEYL----------DLS-------YCSL----DQ--L-----DMT-----YF--E-KAT

----------------------------------------------------------N-

---------------------LKELLLSGNKIKFI-PSHW--KS-PSLQTLALDGNSFGL

ISME---------S-FQ------------DMPQ-------------LSRLRAG-NNPYHC

TCEL--H-AF----IEDT--------------V------------ST-------------

-------G-----------KV----NL--TD-W-----PENFRC-YHP-EA----FL---

-NTVIANY--LPGRVA-C-DIRL-----VIVIC--VATTAAVILILMVI----C--YIFD

V-PW-YTKAT---YQ---I-----IR----A-----------------------------

------K---YRAHKE----------------KMT-GE----A---EVF---V----YHA

FISYSHSDA---EWVRDQLLPTL----ENN--------------------RNP-------

-Y-----RLCIHERDFM----------------P--GK-WIIDNII-ENIENSRKVIFVL

SR------HFV-----------NSE-------WCNY-ELYFAQQRAMG--K-T-FSDVIL

VVKE--P-IDPNSL----------------------------------------------

--PSKYCKLKK-MLSTKTYLEWP----QQ-------VKQQA-------FFWEQLRSVL--

--GKP------------VQ-------------------------------T-RER-LHSA

KSRTSSV-----------------------------------------------------

-----------------------------------------------------------

>TLR18_Anst

------------------------------------------------------------

------------------------------------------------------------

------------------------------------------------------------

------------------------------------------------------------

------------------------------------------------------------

------------------------------------------------------------

----------------ML-WK--SL--HFC--S---------------------------

-------------------------------LLLGVVA-------SPTP---YS--QPT-

----------------------------------------------------AITDR--G

-ICN-V------------------------FNA---GRSADCL-----------------

-----------G------RQ--LD-S-------VP----W-R-QL-----P-------S-

T--LEDI-------DLTYN-------K---L--Q-----------AVHVDDFQS--F-PW

L-----------RSLQLQ-FNN------------------I-SS--IAD--DAFKNNKQL

EILNIF------N--------NS-L-QE-----------------------IP---T-KA

------L-TPL---LN---LKELYMS-NN-----L-YKHAT------LA---N---S---

----FS-KFA------------K-------------------------------------

-------------LQVLSMG-G-P-QVMGLK----------------KGDFQALK----N

IRLYGFAIKCSS--NL--SY-YE----PG--S------------LEV---IQA---VQ-M

GFDMAVD-----QR---------------------------------PEAL--LHML---

-RDI--AN----K----TFS---------------VIQF----RNLF----------E--

F--MY---Y-MGR---EDI-------FS----GLEY-------V--TA--HQLIF----H

RGK---------------FNENLL---RM------------A-LMNLQI----A-PI-K-

-RL---------RLQY---------------------ID---F---ARS--PTF-----V

------------D----S-E---T--S-------S--SI-TDL-----------------

E------------------LDN---LDLWYISN-PDV-----------------------

-------------------------------------------------LRF---DWR--

-------------------------------FTWFN------------------------

---------------------------K--IKE-------LSIQYVYFNSV-PCDS--WV

EMEG--VQFLDVSNNR--------L-E-N------E------YLFNQ-L-CDYK------

----------------GR--M-PN-LHTFKMNNNE-------------L--T-SL-KDLS

-SLTREF--QQ-LKVLDFSN-N-K--------------------------L-G---SA-E

--------ES------------R--NCV-W----------Q-----K-------------

--NIKRLIAHHNQ----FVGE-----------------ALHCLPT---------------

TVQFL----------DLS-------YCDL----DY--L-----DMT-----YF--E-KAT

----------------------------------------------------------E-

---------------------LTELFLSGNKIKFI-PSEW--KS-PSLQLLTLDGNSFGL

VSKE---------S-FE------------DMPQ-------------LSHLRAG-NNPYHC

TCEL--H-AF----VQDT--------------I------------SE-------------

-------G-----------KI----NL--TD-W-----PGNYRC-YHP-EA----FL---

-NTVIAKY--FPSQVA-C-DIRL-----VIIIS--VATTAAVILILMLI----C--YIFD

L-PW-YTKAT---YQ---I-----IR----A-----------------------------

------K---YRAHKE----------------KAA-GE----S---CAY---T----YHA

FISYSHSDA---DWVRDQLLPCL----ENS--------------------KNP-------

-Y-----RLCIHERDFM----------------P--GR-WIIDNII-DNIENSRKVIFVL

SR------HFV-----------NSE-------WCNY-ELYFAQQRAMG--K-T-FSDVIL

VVKE--P-IDPGSL----------------------------------------------

--PSKYCKLKK-MLSTKTYLEWP----QQ-------VNQQP-------FFWAQLKGVL--

--GKP-------------------------------------------------------

------------------------------------------------------------

-----------------------------------------------------------

>TLR18_Ante

------------------------------------------------------------

------------------------------------------------------------

------------------------------------------------------------

------------------------------------------------------------

------------------------------------------------------------

------------------------------------------------------------

----------------MI-WK--LI--NLC--V---------------------------

-------------------------------LLSRALT-------SPTP---SS--PPI-

------------------------------------------------------TEE--R

-PCH-I------------------------YNF---GRSADCL-----------------

-----------G------RQ--LY-S-------VP----R-R-QF-----P-------S-

T--LEQI-------DFSYN-------R---L--Q-----------AVHVDDFAH--L-PQ

L-----------RILQLQ-YNN------------------I-SH--IDN--NAFKNNALL

EHLDIF------N--------NS-L-QE-----------------------IP---A-TA

------L-TPL---LN---LKKLYMS-NN-----L-YKHAT------LA---D---S---

----FS-KFL------------K-------------------------------------

-------------LQLLSMG-G-P-LVMGLK----------------KADFQPLK----N

MKLQSFAIKCSS--NL--SY-YE----PG--S------------LEV---IQT---MQ-M

GFDMAID-----QL---------------------------------PNAL--LYML---

-RDL--AN----K----TFS---------------FIQF----RNLF----------E--

F--TY---Y-MGK---EDI-------FE----GLKY-------I--TA--SQLTF----H

RGK---------------FNENLL---RM------------A-LMNLQI----A-PI-K-

-ML---------RLQY---------------------ID---F---ARS--PTF-----V

------------D----S-G---A--G-------S--SI-TDL-----------------

A------------------LNK---LDLWYISN-PDV-----------------------

-------------------------------------------------LRF---DWR--

-------------------------------FTWFN------------------------

---------------------------K--IRE-------LSIQNVYFNSV-PCDA--WV

EMEG--VEFLDVSNNR--------L-K-D------E------FIFNQ-L-CNYK------

----------------GS--M-PN-LRTFNTSTND-------------L--T-SL-KVLS

-LLTKEF--KQ-LQELDFSN-N-Q--------------------------L-G---SA-I

--------NS------------Q--NCV-W----------H-----Q-------------

--NITRVIAHHNQ----FVSE-----------------ALHCLPT---------------

TVHYL----------DLS-------YCNL----DQ--L-----DIT-----YF--E-KTT

----------------------------------------------------------N-

---------------------LKKLLLSGNKIKFI-PSKW--KS-PSLQSLALDGNSFGL

VSMA---------S-FQ------------DMPQ-------------LSQLSAG-NNPYHC

TCEL--H-AF----IEDT--------------I------------SK-------------

-------G-----------KV----NL--TD-W-----PQNYRC-YHP-EA----FL---

-NTFVSKY--FPGKVA-C-DIRL-----VIIIC--VATTAAVILILMLI----C--YIFD

L-PW-YTKAT---YQ---I-----IR----A-----------------------------

------K---YRAHKE----------------KSA-VE----S---GTF---T----YHA

FISYSHSDA---DWVRDQLLPCL----ENN--------------------RNP-------

-Y-----RLCIHERDFM----------------P--GK-WIIDNII-ENIESSRKVIFVL

SR------SFV-----------NSE-------WCNY-ELYFAQQKAMG--K-T-FSDVIL

VVKE--P-IDPRSL----------------------------------------------

--PSKYCRLKK-MLSTKTYLEWP----QQ-------VNQQA-------FFWLQLKSVL--

--GKP------------TM-------------------------------T-CEE-TQSL

RNRTSSVG-----GISVIAYPREDET-------PD-----AVTPNVNKEEEAINE-TNGN

NDNLSNQRPIPLVAV--------------------------------------------

>TLR18_Amoc

------------------------------------------------------------

------------------------------------------------------------

------------------------------------------------------------

------------------------------------------------------------

------------------------------------------------------------

------------------------------------------------------------

----------------ML-WK--LI--PLY--A---------------------------

-------------------------------LLLGVLT-------SPTP---SS--LPT-

----------------------------------------------------VNTEQ--R

-LCH-I------------------------YNS---GRTADCL-----------------

-----------G------RQ--LE-N-------VP----W-R-QF-----P-------S-

T--LEEI-------DLSYN-------K---L--Q-----------AVSADDFLR--L-PQ

L-----------RILQLR-YNN------------------I-SH--IDD--DAFKNNALL

EHLDIF------N--------NS-L-QE-----------------------IP---A-TA

------L-TPL---LN---LKILYMS-NN-----L-YKRAA------LA---D---S---

----FS-KFV------------K-------------------------------------

-------------LQELSMG-G-P-LVMGLK----------------KGDFQPLM----N

ISLQGFAIKCSS--NL--SY-YE----PG--S------------LEV---IQT---KR-M

GFDMAID-----QQ---------------------------------PSAL--LHML---

-HDL--AN----K----TFI---------------VIQF----RNLF----------E--

F--MY---Y-MGQ---EDI-------FL----GLKD-------I--TA--HQLIF----H

RGK---------------FNENLL---RM------------A-LMNLQV----A-PI-Q-

-RL---------RLQY---------------------ID---F---ARS--PTF-----V

------------D----S-G---A--G-------S--SI-TDL-----------------

T------------------LDN---LDLWYISN-PDV-----------------------

-------------------------------------------------LRF---DWR--

-------------------------------FTWFN------------------------

---------------------------K--IKR-------LSIRYVYFNTV-PCDA--WV

EMEG--VQFLDVSNNR--------L-K-N------E------FIFNR-R-CDYK------

----------------GT--M-PN-LHTFNMSTND-------------L--T-SL-KDMS

-SLTREF--QQ-LQVLDFSN-N-K--------------------------L-G---SA-E

--------ES------------R--DCV-W----------K-----Q-------------

--NITRLIAHHNQ----FVSE-----------------ALSCLPT---------------

TVQYL----------DLS-------YCNL----DQ--L-----GMT-----YF--E-KAT

----------------------------------------------------------N-

---------------------LRTLLLSGNKIKFI-PPKW--ES-PSLQSLALDGNSFGL

ISKE---------S-FQ------------DMPQ-------------LSCLMAG-NNPYHC

TCEL--R-AF----IEDT--------------L------------SK-------------

-------G-----------KV----NL--TD-W-----PWNYKC-YHP-EA----LL---

-NTVIAKY--FPGKVA-C-DIRL-----VIIIC--VATTSAVILILMLI----C--YIFD

L-AW-YTKAT---YQ---I-----IR----A-----------------------------

------K---YRVHKE----------------KAA-GD----L---ETF---T----YHA

FISYSHSDA---DWVRDELLPCL----EKN--------------------RNP-------

-Y-----RLCIHERDFM----------------P--GK-WIIDNII-DNIESSRKVIFVL

SR------HFV-----------DSE-------WCNY-ELYFAQQRAMG--K-S-FSDVIL

VVKE--P-IDPSSL----------------------------------------------

--PSKYCKLKK-MLSTKTYLEWP----QQ-------VNQQP-------FFWAQLKSVL--

--GKP------------KM-------------------------------T-QNR-TYSV

KSRTSSVG-----GISVIRPPTGERA-------SE-----EEDCKVADKTDIY-------

-----------------------------------------------------------

>TLR18_Thal

------------------------------------------------------------

------------------------------------------------------------

------------------------------------------------------------

------------------------------------------------------------

------------------------------------------------------------

------------------------------------------------------------

----------------MI-VR--LI--NFS--A---------------------------

-------------------------------LLLGTLT-------SPTPSP-SS--PPT-

----------------------------------------------------NNNEE--G

-PCR-I------------------------DKS---GHFADCL-----------------

-----------G------RQ--LG-S-------VP----W-K-QF-----P-------S-

T--LEKI-------DLSYN-------R---L--Q-----------AIHADDFLH--L-PQ

L-----------RILLLQ-YNN------------------I-SR--IDN--DAFKNNTLL

ENLNIF------N--------NS-L-QE-----------------------IP---A-TA

------L-TPL---LN---LRELSMS-NN-----L-YKHAT------LA---D---S---

----FS-KFV------------K-------------------------------------

-------------LQSLSMG-G-P-LVMGLK----------------KEDFQPLK----N

IRLQGFAIKCSS--NL--SY-YE----PG--S------------LQV---IQT---QQ-M

GFDMAID-----QQ---------------------------------PNAL--RDML---

-HDL--AN----K----TFG---------------VIQF----RNLF----------E--

F--MY---Y-MGE---EDI-------FQ----GLKG-------I--KA--HQIIF----H

RGK---------------FNENLL---RM------------A-LLNLEV----A-PV-K-

-RL---------RLQY---------------------ID---F---ARS--PKF-----I

------------D----S-G---A--G-------S--SI-RDL-----------------

A------------------LDR---LDLWYISN-PDV-----------------------

-------------------------------------------------LRF---DWR--

-------------------------------FTWLN------------------------

---------------------------K--IKE-------LSIQYVYFNSV-PCDA--WV

EMEG--VKFLDVSNNR--------L-K-D------E------YVFNQ-L-CDYK------

----------------GT--M-PN-IHTFNMSSNE-------------L--A-SL-EDLS

-ALTREF--QQ-LQVLDLSN-N-K--------------------------L-G---TA-K

--------HS------------Q--DCV-W----------Q-----Q-------------

--NITRFIAHHNT----FESE-----------------ALRCLPT---------------

TVHYL----------DLS-------HCDL----DQ--L-----DMT-----YF--E-KAT

----------------------------------------------------------N-

---------------------LKELLLSGNKIKFI-PSKW--ES-PSLQSLALDGNSFGL

ISKE---------S-FQ------------DMPQ-------------LSRLRAG-NNPYHC

TCEL--H-AF----VQDT--------------M------------SK-------------

-------R-----------KV----NL--TD-W-----PWNYRC-YHP-EA----LL---

-NTIISKY--FPGQVA-C-DIRL-----VIIIC--VATTAAVILILMLI----C--YIFD

L-PW-YTKAT---YQ---I-----IR----A-----------------------------

------K---YRAHKE----------------KAA-GE----P---GTF---T----YHA

FISYSHSDA---DWVRDELLPCL----ENN--------------------RNP-------

-Y-----QLCIHERDFM----------------P--GK-WIIDNII-ENIESSRKVIFVL

SR------NFV-----------NSE-------WCNY-ELYFAQQRAMG--K-T-FSDIIL

VVKE--P-IDPSSL----------------------------------------------

--PSKYCKLKK-MLSTKTYLEWP----QH-------VNQQA-------FFWEQLRSVL--

--GKP------------AM-------------------------------T-RER-AHSV

KSRTSSVR-----SISLM---MEDQR-------PE-----IAIPYMDKEAEPQKEIVKRD

NDHLANHRQIPVVAF--------------------------------------------

>TLR18_Maar

------------------------------------------------------------

------------------------------------------------------------

------------------------------------------------------------

------------------------------------------------------------

------------------------------------------------------------

------------------------------------------------------------

----------------MI-WK--LI--HFS--A---------------------------

-------------------------------LLLGVVT-------SPAP---SS--SPT-

----------------------------------------------------THTEE--G

-PCR-I------------------------YNS---GRSADCV-----------------

-----------G------RQ--LS-S-------VP----W-R-HF-----P-------S-

T--LEDI-------DLSYN-------K---I--Q-----------AVHADDFPR--F-PQ

L-----------RTLQLQ-YNN------------------I-SH--IDN--DAFKNNTRL

EHLDIF------N--------NS-L-QE-----------------------IP---A-AA

------L-TPL---LN---LKMLSMS-NN-----L-YKHAT------LV---D---S---

----FS-TFV------------K-------------------------------------

-------------LQVLSMG-G-P-LVMGLK----------------KGDFEPLR----N

IKLQSFAIKCSS--NL--SY-YE----PG--S------------LEV---IQT---RN-L

GFDMAID-----QQ---------------------------------PYAL--HHML---

-RDL--TN----K----TFS---------------VIQF----RNLF----------E--

F--MY---Y-MGT---EDI-------FQ----GLKD-------I--TV--FQLIF----H

RGK---------------FNENLL---RM------------A-LMNLQV----T-PI-N-

-RL---------RLQY---------------------ID---F---ARS--PTF-----V

------------D----D-G---A--E-------S--SI-KNL-----------------

A------------------LDK---LDLWYISN-PDI-----------------------

-------------------------------------------------LRF---DWR--

-------------------------------FTWFN------------------------

---------------------------K--IKE-------LSIQYVYFNSV-PCDA--WV

EMEG--VELLDVSNNR--------L-K-N------E------VIFSQ-H-CDYK------

----------------GS--V-PN-LHTFNISTND-------------L--T-SL-KDLS

-LLTKEF--QQ-LQVLDFSS-N-Q--------------------------L-G---SA-K

--------TS------------Q--DCV-W----------Q-----Q-------------

--NITRLIAHHNQ----FQSE-----------------ALNCLPT---------------

TVHYL----------DLS-------YCNL----DQ--L-----DMA-----YF--K-KAT

----------------------------------------------------------N-

---------------------LRELLLSGNKIKFI-PTKW--GS-PSLQSLALDGNSFGL

ISMA---------T-FQ------------DMPQ-------------LSQLRAG-NNPYHC

TCEL--H-AF----IQDT--------------I------------SK-------------

-------G-----------KV----NL--TD-W-----PSDYRC-YHP-EA----FL---

-NTVISKY--FPGQVA-C-DIRL-----VIVIS--VATTAAVILILMLI----C--YIFD

L-PW-YTKAT---YQ---I-----IR----A-----------------------------

------K---YRAHKE----------------KAA-GE----V---QEF---A----YHA

FISYSHFDA---DWVRDQLLPCL----ENN--------------------RNP-------

-Y-----RLCIHERDFM----------------P--GR-WIIDNII-ENIESSRKVIFVL

SR------QFV-----------NSE-------WCNY-ELYFAQQRAMG--K-T-FSDVIL

VVKE--P-IDPRSL----------------------------------------------

--PSKYCKLKK-MLSTKTYLEWP----QQ-------VNQQA-------FFWAQLKSVL--

--GKP------------TI-------------------------------T-AER----M

HSRTSSVS-----GLSVTGPPKENEI-------LE-----VLTPNVDEEAEHKHDNIRRN

NDELLNQRQIPMVAF--------------------------------------------

>TLR18_Spau

------------------------------------------------------------

------------------------------------------------------------

------------------------------------------------------------

------------------------------------------------------------

------------------------------------------------------------

------------------------------------------------------------

----------------MM-WK--LV--YFS--A---------------------------

-------------------------------LLLGALS-------SPTP---SS--TPT-

----------------------------------------------------INTDE--G

-PCL-I------------------------YNS---GRSADCL-----------------

-----------G------KQ--LE-S-------VP----W-R-QF-----P-------S-

T--LEDI-------DLSYN-------K---L--Q-----------AVRANDFLH--L-SQ

L-----------RTLKLQ-YNN------------------I-SH--IDN--VAFKNNKLL

EHLNIF------N--------NS-L-QE-----------------------IP---A-AA

------L-TPL---LN---LKELYMS-NN-----L-YKHAT------LA---D---S---

----FS-KFV------------K-------------------------------------

-------------LQVLSMG-G-P-LVMGLK----------------KGDFQPLK----N

IRLQSFAIKCSS--NL--SY-YE----PG--S------------LAV---IQT---RQ-M

GFDMAID-----QR---------------------------------PNAL--RHML---

-RDL--AN----K----TFI---------------TIQF----RNLF----------E--

F--MY---Y-MGD---EDI-------FQ----GLKD-------I--TA--YQLIF----H

RGK---------------FNENLL---KM------------A-LMNLQV----A-SI-R-

-RL---------RLQY---------------------ID---F---ARS--PMF-----V

------------D----S-G---A--G-------S--SI-TEL-----------------

A------------------LDK---LDLWYISN-PDV-----------------------

-------------------------------------------------LRF---DWR--

-------------------------------FTWFK------------------------

---------------------------K--IKE-------LSIQYVYFNSV-PCDA--WV

EMEG--VEIIDISNNR--------L-K-N------E------FIFNQ-R-CDYK------

----------------GA--M-PN-LHTFDINTNE-------------L--T-SL-KDLS

-SLTREF--KQ-LRVLDISN-N-K--------------------------L-G---SA-E

--------DS------------Q--DCV-W----------Q-----N-------------

--NITRLIAHHNQ----FVSE-----------------SLRCLPT---------------

TVHYL----------DLS-------YCNL----DQ--L-----DMV-----YF--E-KAT

----------------------------------------------------------N-

---------------------LKELLLSGNKIKFI-PSKW--ES-PSLQSLTLDGNSFGL

ISKA---------S-FQ------------DMPQ-------------LSQLRAG-NNPYHC

TCEL--H-AF----VQDT--------------L------------SK-------------

-------G-----------KV----NL--TD-W-----PWNYRC-YHP-EA----LL---

-NTVISKY--FPGHVA-C-DIRL-----VIIIC--VATTAAVILIMMVI----C--YIFD

L-PW-YTKAT---YQ---I-----IR----A-----------------------------

------K---YRAHKE----------------KVA-GE----P---EPF---T----YHA

FISYSHSDA---DWVRDQLLPSL----ENS--------------------RNP-------

-Y-----RLCIHERDFM----------------P--GR-WIIDNII-DNIENSRKVIFVL

SR------HFV-----------NSE-------WCNY-ELYFAQQRAMG--K-T-FNDVIL

VVKE--P-IDPNSL----------------------------------------------

--PSKYCKLKK-MLSTKTYLEWP----HQ-------VNQQA-------FFWAQLKSVL--

--GKP------------TM-------------------------------T-REV-RQSV

KSRTSSEG-----GVSVILLPMEDRR-------PE-----EEAPNLDKEAEPNNKIIQKN

NDEQSNQRQIPVVAF--------------------------------------------

>TLR18_Sedu

------------------------------------------------------------

------------------------------------------------------------

------------------------------------------------------------

------------------------------------------------------------

------------------------------------------------------------

------------------------------------------------------------

----------------MI-WT--LI--NFS--A---------------------------

-------------------------------LLLGVLT-------SPTPSSDTT--NPN-

----------------------------------------------------PDTEE--G

-RCR-I------------------------YNS---GRSADCL-----------------

-----------G------RQ--LN-S-------VP----W-R-QF-----P-------S-

T--LQDV-------DLSYN-------K---L--Q-----------AVHADDFIH--L-PQ

L-----------RKLQLT-YNN------------------I-SH--IDN--DAFKNNPLL

EHLDIF------N--------NS-L-QE-----------------------IP---A-RA

------L-TPL---LN---LKQLFMS-NN-----L-YKHAT------LA---D---S---

----FS-KFV------------K-------------------------------------

-------------LQVLSMG-G-P-LVMGLK----------------KADFQPLK----N

IRLYGFAIKCSS--YL--NY-YE----PG--S------------LEV---IQT---KQ-M

GFDMAID-----QR---------------------------------PNAL--LHMI---

-HDL--AN----K----TFS---------------VIQF----RNLF----------E--

F--MY---Y-NGE---EDI-------FQ----GLKD-------V--TA--YQLIF----H

RGK---------------FNENLM---RM------------A-LMNLQV----A-PI-K-

-RL---------RLQY---------------------ID---F---ARS--PTF-----I

------------D----S-G---A--G-------S--SI-TDL-----------------

A------------------LDR---LDLWYISN-PDI-----------------------

-------------------------------------------------LRF---DWR--

-------------------------------FSWFN------------------------

---------------------------K--IKE-------LSIQYVYFNSA-PCDS--WL

EMEG--VEFLDVSNNR--------L-R-N------E------YIFNQ-R-CDYR------

----------------GT--M-PN-LHTFNTSNND-------------L--A-SL-KDLS

-LLIGEF--ER-LQVLDVSN-N-K--------------------------L-G---SA-E

--------NS------------R--DCV-W----------Q-----Q-------------

--NITKLIAHHNH----FVSV-----------------ALRCLPT---------------

TLHYL----------DLS-------YCDL----DQ--L-----DMT-----YF--E-QAT

----------------------------------------------------------N-

---------------------LKELLLSGNKIKFI-PSKW--ES-LSLQSLALDGNSFGL

ISKA---------S-FQ------------DMPQ-------------LSNLRAG-NNPYHC

TCEL--H-AF----VEDT--------------I------------SQ-------------

-------K-----------KV----NL--TD-W-----PWNYKC-YHP-EP----FL---

-NTAISKY--FPGQVA-C-DIRL-----VIIIC--VATTAAVILILMLI----C--YIFD

L-PW-YTKAT---YQ---I-----IR----A-----------------------------

------K---YRAHKE----------------KAA-GE----L---GTF---T----YHA

FISYSHPDA---DWVRDQLLPCL----EKN--------------------RNP-------

-Y-----RLCIHERDFM----------------P--GR-WIIDNII-ENIESSRKVIFVL

SR------HFV-----------NSE-------WCNY-ELYFAQQRAMG--K-T-FSDVIL

VVKE--P-IDPSSL----------------------------------------------

--PSKYCKLKK-MLSTKTYLEWP----QQ-------VNQQA-------FFWEQLRSVL--

--GKP------------TM-------------------------------T-RKR-DHSV

KSRTSSVG-----SISVIGLPNEDVG-------PD-----VAKNNENQEAEIK--LIKRN

NDELSNQRQIPVMAF--------------------------------------------

>TLR18_Scma

------------------------------------------------------------

------------------------------------------------------------

------------------------------------------------------------

------------------------------------------------------------

------------------------------------------------------------

------------------------------------------------------------

----------------MI-WR--LF--HFS--A---------------------------

-------------------------------LLAGALT-------SPTP---TT--APT-

----------------------------------------------------TDTED--G

-PCR-L------------------------YNS---GRSADCL-----------------

-----------G------RQ--LP-S-------VP----W-G-HF-----P-------S-

T--LEDI-------DLSYN-------K---L--R-----------AVHADDFRR--L-PR

L-----------RILQLQ-YNN------------------I-SI--IDN--DAFKNNPLL

EHVNIF------N--------NS-L-QE-----------------------IP---A-RA

------L-TPL---SN---LKQLYMS-NN-----L-YKHAT------LA---E---S---

----FS-KFV------------K-------------------------------------

-------------LEVLSMG-G-P-LVMGLK----------------KADFGPLK----N

IGLQGFAIKCSS--NL--SY-YE----PG--S------------LEV---IQT---KQ-M

GFDMAID-----QR---------------------------------PHAL--VHML---

-RDL--AN----K----TFS---------------VIQF----RNLF----------E--

F--MY---Y-MEQ---DDI-------FL----GLKD-------I--KA--YQLIF----H

RGK---------------FNENLL---RM------------A-LINLQV----T-PI-K-

-RL---------RLQY---------------------ID---F---ARS--PTF-----V

------------D----S-G---A--G-------S--SI-TDL-----------------

A------------------LDK---LDLWYISN-PDV-----------------------

-------------------------------------------------LRF---DWR--

-------------------------------FTWFN------------------------

---------------------------K--IKA-------LSIQYVYFNFV-PCDA--WI

EMEG--VELLDISNNR--------L-Q-S------D------YIFNQ-R-CDNR------

----------------GT--M-PN-LHTFNTSSND-------------L--T-SL-KDLS

-LLTREF--QR-LQVLDVSN-N-K--------------------------L-G---SV-E

--------NS------------W--ECV-W----------Q-----Q-------------

--NITRLIAHHNQ----FVNE-----------------ALHCLPT---------------

TVHYL----------DLS-------YCDL----DQ--L-----DLT-----YF--E-KAT

----------------------------------------------------------N-

---------------------LKELLLSGNKIKFI-PSKW--KG-LSLQSLALDGNSFGL

ISKA---------S-FE------------GMPQ-------------LSRLRAG-NNPYHC

TCEL--H-AF----VQDT--------------I------------SH-------------

-------G-----------KV----NL--TD-W-----PWNYRC-YHP-EA----LL---

-NTVISKY--FPGQVA-C-DIRL-----VIIIC--VATTAAVILILMLI----C--YIFD

L-PW-YTKAT---YQ---I-----IR----A-----------------------------

------K---YRAHKE----------------KAV-GG----S---GTF---T----YHA

FISYSHSDA---DWVRDQLLPSL----ENN--------------------RNP-------

-Y-----RLCIHERDFM----------------P--GR-WIIDNII-ENIESSRKVIFVL

SR------HFV-----------NSE-------WCNY-ELYFAQQRAMG--K-T-FSDVIL

VVME--P-IDPGSL----------------------------------------------

--PSKYCKLKK-MLSTKTYLEWP----QQ-------VNQQA-------FFWEQLKSVL--

--GKP------------AM-------------------------------T-REEGGHSV

KSRTSSVG-----GVAVIGHPQEDGG-------PK-------------------------

-----------------------------------------------------------

>TLR18_Mape

------------------------------------------------------------

------------------------------------------------------------

------------------------------------------------------------

------------------------------------------------------------

------------------------------------------------------------

------------------------------------------------------------

---------MIWKLVHFSALLIGAL--TSP--T---------------------------

----------------------------------------------------------P-

----------------------------------------------------SSAPT--T

-YTD-N----------------------GPCHIYNSGRSADCL-----------------

-----------G------RQ--LD-S-------VP----W-R-QF-----P-------S-

T--LEEI-------DLSYN-------K---L--Q-----------AVHADDFLR--L-PQ

L-----------RILHLK-YNN------------------I-SH--IDN--DVFKNNTLL

EHLDIF------N--------NS-L-QE-----------------------IP---A-TA

------L-TPL---LN---LKQLYMS-NN-----L-YKHAT------LA-------DS--

----FS-KFV------------K-------------------------------------

-------------LEVLSMG-G-P-LVMGLK----------------KADFQALK----N

IKLQGFAIKCSS--NL--SY-YE----PGSLE------------VIQTKQMGF---DMAI

DQLPN----------------------------------------------ALFHML---

-RDL--AN----K----SFS---------------VIQF----RNLF-------------

E--FT---Y-YMG---EED-------IFQ---GLKD-------I--TA--YQLIF----H

RGK-----------FNENLLRMAL---I-------------N-LQVTPI--KRV-RL-Q-

-YI---------DFAR--------------------------S---PTF--IDS----GA

------------G----S-S---I-----------------TN-----------------

LA-----------------LDK---LDLWYISN---------------------------

-------------------------------------------------PDVLRFDWR--

-------------------------------FTWFN------------------------

---------------------------K--IKE-------LSIQYVYFNSV-PCDA--WV

EMGG--VEILDVSNNR--------L-LDEFIFNQLC------DYK---------------

----------------GT--M-PN-LHTFNLTTNE-------------L--T-SL-KDLS

-SLTREF--QQ-LRVLDLSY-N-K--------------------------L-G-------

SA-----KNS------------R--DCV-W----------Q-----K-------------

--NITRLIAHHNQ--FESE-------------------ALHCLPT---------------

TVHYL----------DLS-------YCNL----DQ--L-----DMT-----YF--E-KAT

----------------------------------------------------------N-

---------------------LKELLLSGNKIKFI-PSKWKS---PSLQSLALDGNSFGL

ISKA---------S-FQ------------DMPQ-------------LSDLRAG-NNPYHC

TCEL--H-AF----V-----------------E------------D--------------

--------------------TMSKGKVNLTD-W-----PRNYKC-YHP-EP----FL---

-NTFISKY--LPGQVA-C-DIRL-------VIIISVATTAAVMLILVLI----C--YIFD

L-PW-YTKAT---YQ---I-----IR----A-----------------------------

------KYRA-----H---------------KEKAAGE----T---GTF---T----YHA

FISYSHSDA---DWVRDQLLPCL----ENN--------------------R--N--PY--

-------RLCIHERDFM----------------P--GR-WIIDNII-ENIESSRKVIFVL

SR------HFV-----------NSD-------WCNY-ELYFAQQRAMG--K-T-FSDVIL

VVKE--P-IDPSSL----------------------------------------------

--PSKYCKLKK-MLSTKTYLEWP----QQ-------VNQQA-------FFWAQLMSVL--

--GKP------------TMIRDRAH-------SVKSRTSSVGGVSVIGPPIEDRRPEVEK

PNADKEAESKNNIIKMNNYELSNERQ-------IP-----LAF-----------------

-----------------------------------------------------------

>TLR18_Anja

------------------------------------------------------------

------------------------------------------------------------

------------------------------------------------------------

------------------------------------------------------------

------------------------------------------------------------

------------------------------------------------------------

------------------------MIGLLL--L---------------------------

------------------------------------------SDQA------TL--TII-

----------------------------------------------------S--DK--S

-ICK-I---------------------------SSDQRLADCR-----------------

-----------G------RL--LA-E-------VP----V-K-DL-----P-------P-

S--LEEV-------DLSHN-------A---L--Q-----------VIHHDDFSH--L-YR

L-----------RVLQLQ-YNN------------------I-SL--IED--DAFRFNPLL

EYLDVF------N--------NS-L-RA-----------------------IP---T-EA

------L-KPA---SR---LRILKMS-NN-----F-YTEAT------LD-------NI--

----FA-SFT------------H-------------------------------------

-------------LKELSFG-G-P-FIQSLK----------------KGDFQALQ----K

IPLDTFAIKTAS--SL--QN-YE----PG--C------------LKV----------IQT

KN-LWFD-----VA--------LDQKPDIL-----------------------PVML---

-GDL--AN----K----TFA---------------LLRF----RNLF----------E--

F--KY-----YSG---EMN-------IFQ---GLAD-------I--KT--HQLIF----Y

RGK----------------FNENL--LRMAL----------INIQVSPI--RGL-GL-L-

-FI---------DFAR---------------------SP-------------TF-----V

------------D----------S--G-----------VGSSV-----------------

TD----L---A--------LDD---LVLWDISN---------------------------

-------------------------------------------------PDILRFDWR--

-------------------------------FTWFS------------------------

---------------------------K--VRR-------LSIKNVNFNFV-PCDA--WD

EMGM--IEVLDVSNNR--------L----------Q------DHYLYNRRCQYS------

----------------GA--M-RS-LHSFNCSTNT-------------L--S-SL-SDIA

-SLTTEF--WK-LKVLDLSY-N-Q--------------------------L-G-------

-------SKE------------G--ACT-W----------K-----S-------------

--NITRMILHHNNFVS----S-----------------TLSCLPT---------------

TVQYL----------DLS-------FCNL----DL--V-----DMD-----YF--K-QAT

----------------------------------------------------------N-

---------------------LKELLLSGNKIKFI-PSGWSN---PALLSLALDGNSFGL

ISMS---------S-FK------------NMPS-------------LSRLKAG-NNPYHC

TCDL--H-AF----I--Q--------------E----T---TAK-GK-------------

-------V-----------NI--------TD-W-----PDNYRC-YHP-EA----WL---

-NTVVANF--FPGRIA-C-DIRL-------VIVISVVTTTVVVLAVMVL----C--YVFN

V-PW-YAKAT---YQ---I-----LR----A-KY--------------------------

-------------RAH--------------------KEGEGVL---QTY---A----YHA

FISYSHSDA---DWVRDKLLPCL----ENSKPPY--------------------------

-------NLCIHERDFL----------------P--GK-WIIDNII-ENMENSRKVIFVL

SR------NFV-----------NSE-------WCNY-ELYFANQRAIG--K-T-FNDVIL

VVKE--P-IEPNSL----------------------------------------------

--PNKYCKLKK-MLSTKTYLEWP----HE-------AKHQV-------FFWAQLKSVL--

--GKP------------SI-----------------------------------------

------------------------PR-------DR-----ASSTRSPHSEVNVSMVELP-

-----------------------------------------------------------

>TLR18_Scfo

------------------------------------------------------------

------------------------------------------------------------

------------------------------------------------------------

------------------------------------------------------------

------------------------------------------------------------

------------------------------------------------------------

------------------------------------------------------------

---------------------------------------M--Y--K------LH----C-

---PV---VAMM-----------------TITLTDLLVSLCGSHQHPSGRYTC--NI---

--SL-E------------------------------KRFADCK-----------------

-----------G------LH--LT-Q-------MP----I---GL-----H-------S-

S--LEEL-------DLSSN-------A---L--Q-----------GIREGDLSN--L-TK

L-----------RVLRLQ-YNN------------------I-SV--IED--QAFQYNTLL

EDLNIF------N--------NS-L-RS-----------------------IP---A-KA

------L-EPL---SK---LRILDMS-NN-----F-YTEAT------LP---D---I---

----FS-TFT------------Q-------------------------------------

-------------LKILSMG-G-P-LIPSLK----------------KDDFWTLQ----N

ITLEKFAIKSAS--SL--LR-YE----LG--C------------LKF----------IRT

TR-MWFD-----VA--------LDQRPDA-------L----------------PLML---

-QDL--AN----K----SFT---------------ELRF----RNLF----E-----F--

T--YY---T-G-K---EDL-------LQ----GLKH-------I--RM--RQLVF----Y

RGK----------------FNENL---LR-M----------A-LVNLQN----S-SV-K-

-SL---------GLLF---------------------ID---------F--ARS-----P

------------T----F-----V--D-------S--GS-DSS----------V-----T

NL----V------------LDE---LFLWDISN-PDI-----------------------

-------------------------------------------------LRF---DWR--

-------------------------------FTWFS------------------------

---------------------------K--VRR-------LSIKNVNFNFV-PCDA--WN

EMKA--VEELDVSNNR--------L-Q--------D--------------EYIY--NK--

-----RCDY----K--KT--L-PV-LSTFNVSKNQ-------------L--T-KL-RDVV

-ALVGEF--KN-MRVIDLSY-N-Q--------------------------L-G---SSEI

TN-----LEN------------N--KCN-W----------K-----Q-------------

--NFTKVILHHNN--F----I-SS--------------DLSCLPI---------------

NVEYL----------DLS-------YCNL----DH--L-----DMD-----YF--E-RVS

----------------------------------------------------------N-

---------------------LKELLLSDNKIKFI-PSGW--SN-PTLQSLTLDGNSFGL

ISMG---------S-FK------------DMPN-------------LSNLKAG-NNPYHC

TCDL--H-TF----F--Q--------------E----T-------AV----------KGK

-------V-----------NI--------TD-W-----PENYRC-YHP-ES----FL---

-NLMVAKF--SPGSVA-C-DIRL-----VVIIS--VATTATVVLAIMLL----C--YVFN

V-PW-YMRAT---YQ---I-----LR----A--K------------------Y------R

A-Y---Q----E-GS---------------------GL----S---QEF---D----YHA

FISYSHSDA---EWVRDQLLTYL----ESS---N--------------PP----------

---Y---RICIHERDFM----------------P--GK-WIIDNII-ENIENSRKVIFVL

SR------HFV-----------NSE-------WCNY-ELYFAQQRAIG--K-T-FSDVIL

VVKE--P-IDPESL----------------------------------------------

--PNKYCKLKK-MLNTKTYLEWP----KE-------TKQQA-------FFWAQLKSVL--

--GKP------------TF-----------------------------------------

-------------------T-RDQ-A-------AS-----TRNRNSAA------AVSTIE

LPLEDELVSQSLPSEHSSTATMEGSVLSN------------------------------

>TLR18_Cami

------------------------------------------------------------

------------------------------------------------------------

------------------------------------------------------------

------------------------------------------------------------

------------------------------------------------------------

------------------------------------------------------------

------------------------------------------------------------

------------------------------------------------------------

------------------------------------------------------------

------------------------------------------------------------

------------------------------------------------------------

-------------------------------------------------MELDG--L-SQ

L-----------KVLSLK-YNR------------------I-SW--LHP--KAFASNRQL

EHLDLF------N--------NS-L-TA-----------------------IP---D-RV

------L-SPL---RS---LRRLDMS-NN-----L-YTSAK------LG---P---I---

----FS-QLP------------L-------------------------------------

-------------FRHLSMG-G-S-LIPAIL----------------VGDFKVLR----N

VSLEQFALKAQS--SL--PK-YQ----PG--A------------LAE----------VRT

ES-LWFD-----FA--------LDKNPSA-------L----------------PDIL---

-VDI--SR----N----QVA---------------SVRF----RNLF----E-----F--

T--YY---T-G-W---LDL-------FA----PLRQ-------T--RV--RNLSF----F

RGK----------------FNENL---LG-F----------I-LKNVEG----S-EV-R-

-NL---------SLQS---------------------VD---------F--ARS-----V

------------N----R-----S--A-------S--LP-N------------I-----E

DL----V------------LDW---LTLTEISN-PEV-----------------------

-------------------------------------------------LGF---NQR--

-------------------------------FSWFS------------------------

---------------------------K--VQA-------LVIKKINFNYV-PCPA--WN

AMKL--VRFLDISDNQ--------L-E--------N--------------SYIY--NK--

-----RCRY----Q--GT--M-AR-LEEFLLHHNA-------------I--K-SL-REVA

-LLTAEW--AS-LHTIDLSN-N-Q--------------------------I-G-------

--------RD------------E--TCE-W----------R-----Q-------------

--PIATLILHHNR--V----T-SA--------------IFKCLPT---------------

SLVHL----------DMS-------HSQL----ER--L-----ERA-----YF--E-MAV

----------------------------------------------------------N-

---------------------LTKLLLSGNRIKFI-PSGW--SS-PGLQTLAVDGNSFGV

ITKG---------S-FQ------------LMPS-------------LATLRAG-NNPYHC

TCDL--F-RF----V--Q--------------D----I-------HR----------RGL

-------L-----------RL--------AG-W-----PEDYIC-YHP-DR----YI---

-DTRVVDY--APGRLE-C-DVSL-----VVAIS--VSTTAVLVASSMVL----C--WRFD

A-LW-YIRAT---WQ---I-----IR----S--R------------------Y------R

A-E-----------A---------------------AA----S---KAF---A----YHA

FISYSHSDA---NWVRGELLARL----ESN---E--------------PP----------

---Y---RICIHERDFT----------------P--GK-WIIDNII-ENIENSRKVIFVL

SK------SFV-----------NSE-------WCNY-ELYFAHQRAIG--Q-A-FEDVVL

VVIE--A-IKPDSL----------------------------------------------

--PNKFCKLRK-MLDTKTYLEWP----PE-------ANRQP-------FFWAQLKNWF--

--TPS------------LF-----------------------------------------

-------------------V----------------------------------GMFLAQ

MPAKFATKPDYFRHS--------------------------------------------

>TLR14_Hyre

------------------------------------------------------------

------------------------------------------------------------

------------------------------------------------------------

------------------------------------------------------------

------------------------------------------------------------

------------------------------------------------------------

------------------------M--FQR--I---------------------------

--------------------------------G---R--L--L--L------LS----I-

---IM---TGGI-----------------R-------------VSRCLT--PC--TV---

--NE-E------------------------------HV-CLCS-----------------

-----------G------KN--LN-E-------VP----K---DL-----P-------I-

S--LEQL-------DLSHN-------K---I--M-----------TISKADFSS--Y-TN

L-----------KALSLA-YNS------------------I-YK--IEN--DSFASNLLL

EKLSLF------N--------NS-L-TE-----------------------IP---S-LA

------L-SPL---KN---LKELEMS-NN-----L-YTNST------LD---D---I---

----FK-TFQ------------N-------------------------------------

-------------LKVLSMG-G-T-EIKWVS----------------RDDFLPLQ----N

ISLHKFALKTAS--SP--EH-YE----KG--A------------FAV----------LDT

SS-LWCD-----FA--------LDMNANA-------L----------------LPIL---

-EDL--GG----K----SMK---------------LLRF----RNLF----G-----N--

S--YY---T-E-K---TDL-------FD----GLAN-------I--DV--NELTF----Y

RGK----------------FNENL---LR-H----------L-LLNVQK----S-NV-S-

-NL---------NLVA---------------------VD---------F--ARS-----L

------------N----S-----S--S-------S--TA-G------------I-----N

NL----T------------LNK---LLIQDVSN-PDI-----------------------

-------------------------------------------------LRF---DWT--

-------------------------------FTWFS------------------------

---------------------------N--VIN-------LFIINVNFNFV-PCDA--WG

EMKN--IVVLNMSNNR--------L-K--------D--------------EYVY--NP--

-----SCQ-----D--PY--L-PK-VKEFILTNNQ-------------I--T-SL-KIIS

-RLTFNW--PN-LRVLDLGY-N-S--------------------------I-I---SR--

--------ME---------------SCN-W----------G-----P-------------

--AINTLILHHNN--M----N-RS--------------VFQCLPT---------------

SLRHL----------DLS-------NCQL----DE--L-----DLN-----YF--R-QAT

----------------------------------------------------------N-

---------------------LCELRLSDNKIKFI-PSKW--ES-PNLEVLNVDGNSFGV

ISKG---------S-FD------------NMPK-------------LAKLRAG-NNPFHC

TCDL--A-EF----F--R--------------D----T--------R----------HKN

-------L-----------NV--------LD-W-----PQRYIC-YHP-EH----LL---

-HTRIEDY--DPSRLE-C-DITL-----VVAIS--VATTALLVIVSMML----C--WKFD

V-PW-YLKAT---WQ---I-----LQ----S--R------------------Y------R

S-Q---Q-------E---------------------IP----C---RTY---L----YHA

FVSYSYLDA---EWVREELLRRL----ENC---N--------------PP----------

---F---TICIHERDFL----------------P--GK-WIIDNII-ENIENSRKVIFVL

SR------SFV-----------NSD-------WCNY-ELYFAHQRCIG--H-A-FDDVIL

VVKE--D-INLDSL----------------------------------------------

--PNKFCKLRK-MLSTKTYLEWP----SD-------ESKQP-------FFWVQLKSVL--

--GKV------------TL-----------------------------------------

-------------------G-STD-N-------EG-----MSLAN---------QSAFIE

APVVSENSNDHVFTVNL---------------------------------P--------

>TLR14_Amme

------------------------------------------------------------

------------------------------------------------------------

------------------------------------------------------------

------------------------------------------------------------

------------------------------------------------------------

------------------------------------------------------------

------------------------AAHLAH--C---------------------------

------------------------------------------QVAY------LG----R-

----------------------------------------------------C--PN---

-PCT-VNE----------------------------NNVGLCS-----------------

-----------G------SR--LS-E-------VP------K-NL-----S-------T-

H--VVEL-------DLSHN-------E---I--R-----------KISKTDFLA--Y-TN

L-----------TVLNLA-FNK------------------I-FE--IEN--DSFASNLLL

KKLNLF------N--------NS-L-TE-----------------------IP---S-QA

------L-SPL---KE---LEELDMS-NN-----L-YVNST------LS-------DV--

----FY-SLK------------K-------------------------------------

-------------LAVLSMG-G-T-EIVWIS----------------KNDFLPLK----N

NILQKFALKTAS--SP--RH-YE----RG--A------------FAV----------LNT

DS-LWCD-----FA--------LDTNPNVL-----------------------LLIL---

-EDL--RG----K----SMT---------------LLRF----RNLF----A-----N--

S--YY-----------TDE-----GDLFS---GLAD-------I--GM--TQLVL----F

RGK----------------FNEHL-------------------LRLLLLNVQKS-NV-T-

-DL---------SLMS---------------------ID---------F--ARS-----F

------------N---------------------------TSA-----------------

PK----P---S--------IQN---LTLNKLLL-QDI-----------------------

-------SN----------------------------------------PDILRFDWT--

-------------------------------FTWLS------------------------

---------------------------K--VIS-------LFIINVNFNYI-PCDA--WR

EMQN--VVTLNISDNR--------L-L-----------------------NEFIYNPSCQ

N---------------PS--I-PL-LTQFNASKNL-------------I--T-SL-KTIS

-QMTFKW--PR-LKIIDLSY-N-K--------------------------I-G-------

-------SKN------------E--FCK-W----------G-----P-------------

--AITKFIMHHNI--I----KEE---------------VFQCLPT---------------

SLHFL----------DLS-------NTEL----DV--L-----QLD-----YF--N-RAT

----------------------------------------------------------N-

---------------------LSELWLSNNRIKFI-PSNWES---PNLRVLKLDGNSFGL

ISKG---------S-FD------------KMPN-------------LVQLNAG-NNPFHC

TCDL--S-MF----IQEN--------------LYNGNL----------------------

-------------------KF--------MK-W-----PNSYTC-YHP-PD----YL---

-HSKVESY--SPGMLE-C-DVTL-------IVIISITSTALLVIVSMLL----C--WKFD

I-PW-YVKAT---CQ---I-----IQ----S-----------------------------

------KYRSGQGIAP--------------------------K---SYI--------YHA

FVSYSSSDA---DWVRSQLLNRL----ENC------------------SPPY--------

-------SICIHERDFL----------------P--GK-WIIDNII-ENIEKSRKVIFVL

SH------SFV-----------NSE-------WCNY-ELYFAHHRCIG----HAFDDVIL

VVKE--N-VNLDSL----------------------------------------------

--PTKFCKLRK-MLSKKTYLEWP----SE-------TSKQP-------FFWMKLKNVL--

--GKD------SIQSTGQE-----------------------------------------

-------------------GMFLGYE-------ND-----FLELPVTSGNTLAAAHALNS

L----------------------------------------------------------

>TLR14_Limo

------------------------------------------------------------

------------------------------------------------------------

------------------------------------------------------------

------------------------------------------------------------

------------------------------------------------------------

------------------------------------------------------------

------------------------M--VQS--S---------------------------

--------------------------------G---R--W--L--A------LF----I-

---LM---TGVA-----------------N-------------VAQSQM--TC--TV---

--NE-N------------------------------NV-ALCS-----------------

-----------G------LS--LS-E-------VP----K---YL-----S-------T-

S--RVEL-------DLSYN-------K---L--Q-----------SISEEDFSA--Y-TN

L-----------TILNLA-FNN------------------I-FR--IDR--NSFASNLLL

RKLNIF------N--------NS-L-KE-----------------------IP---S-LA

------I-SPL---EH---LTELEMS-NN-----L-YLNST------LD---D---I---

----FY-TFR------------S-------------------------------------

-------------LTVLSMG-G-S-EITWIR----------------KNDFLPLQ----N

ISLQKFALKTAS--SP--MH-YD----KG--T------------FAV----------LNS

FS-LWCD-----FA--------LDSNPYV-------L----------------PLIL---

-GDL--RG----K----SIK---------------LLRL----RNLF----A-----H--

S--YY---T-D-D---MDL-------FN----GLAD-------T--DL--NELIL----Y

RGK----------------FNENL---LR-L----------L-LINVQI----S-NV-T-

-NL---------CLNA---------------------ID---------F--ARS-----L

------------N----L-----Y--T-------S--NV-K------------I-----S

NL----T------------LNK---LLLKDINN-PDI-----------------------

-------------------------------------------------LRF---DRT--

-------------------------------FTWFS------------------------

---------------------------K--VSN-------LYIINVNFRYV-PCDA--WD

EMKN--VIVLNISNNR--------L-T--------N--------------EFVH--NP--

-----TCQ-----N--PS--V-PK-LKCFNASSNK-------------I--T-SL-KIIS

-LLSLNW--PN-LRVVDLSH-N-G--------------------------I-G---SN--

--------NE---------------YCS-W----------G-----P-------------

--AITKLILHHNI--I----K-KI--------------IFQCLPT---------------

SLHYL----------DLS-------NSEL----DG--L-----DTD-----YF--I-KST

----------------------------------------------------------N-

---------------------LSELFLSNNRIKFI-PSTW--ES-PNLRFLAIDGNSFGV

ITTG---------S-FG------------SMPR-------------LAKLKAG-NNPYHC

TCEL--A-LF----I--E--------------E----T-------TQ----------YGD

-------L-----------EF--------AD-W-----PQNYKC-YHP-EY----LL---

-RSMIESY--NPGWLE-C-DVTL-----VVIIT--VTTTAVVVIISMLL----C--WKFD

V-PW-YIKAT---CQ---I-----IQ----S--R------------------H------R

S-R---K-------G---------------------IP----C---RTY---S----YHA

FVSYSSSDA---DWVRRELLYRL----EQC---N--------------PP----------

---L---SICIHERDFM----------------P--GK-WIIDNII-ENIEKSRKVIFVL

SR------SFV-----------DSE-------WCNY-ELYFAHQRCIG--H-A-FDDVIL

VVKE--K-VSLDSL----------------------------------------------

--PNKFCKLRK-MLSTKTYLEWP----SE-------PNKQA-------FFWVQLKNVL--

--GKC------------TL-----------------------------------------

-------------------E-SAD-Q-------QR-----ISWTN---------DSAMSE

LPVAL------------------------------------------------------

>TLR14_Sain

------------------------------------------------------------

------------------------------------------------------------

------------------------------------------------------------

------------------------------------------------------------

------------------------------------------------------------

------------------------------------------------------------

------------------------M--LQS--S---------------------------

--------------------------------G---R--W--L--A------LF----I-

---LM---AGVA-----------------H-------------IAQSQI--PC--TV---

--NE-N------------------------------NV-ALCS-----------------

-----------G------LN--LS-E-------AP----T---NL-----S-------T-

S--VVEL-------DLSFN-------K---L--W-----------SISKADFAA--Y-TN

L-----------TILNLG-YNS------------------I-FT--IER--DSFASNLLL

RKLNLF------N--------NS-L-TE-----------------------IP---S-LA

------I-SPL---KN---LKELEMS-NN-----L-YLNST------LG---D---V---

----FY-TFK------------S-------------------------------------

-------------LMVLSMG-G-S-EITWIR----------------KNDFLPLQ----N

ISLQKFALKTAS--SP--KH-YD----KG--A------------FAV----------LNT

FS-LWCD-----FA--------LDTNPYV-------L----------------PLIL---

-GDL--RG----K----SIK---------------LLRL----RNLF----A-----N--

S--YY---T-D-D---MDL-------FS----GLAD-------T--GV--KELIL----F

RGK----------------FNENL---LR-L----------L-LLNVQI----S-NV-T-

-DV---------GLIA---------------------ID---------F--ARS-----L

------------N----L-----S--A-------S--NV-G------------I-----S

NL----T------------LNK---LLLQDITN-PDI-----------------------

-------------------------------------------------LRF---DWT--

-------------------------------FTWFS------------------------

---------------------------K--VSN-------LHIININFNYV-PCDA--WD

EMKN--VIVLNISNNR--------L-T--------N--------------EFIY--NP--

-----KCQ-----N--PP--L-PK-LECFNASSNK-------------I--T-SL-KTIS

-LISLNW--PN-LQVVDLSH-N-G--------------------------I-G---SN--

--------NE---------------SCS-W----------G-----P-------------

--AIIKLILHHNI--I----K-QI--------------IFRCLPT---------------

SLHYL----------DLS-------NSEL----DG--L-----DTN-----YF--I-KST

----------------------------------------------------------N-

---------------------LSELFLSNNRIKFI-PSSW--ES-PNLLFLAVDGNSFGV

ITTG---------S-FD------------KMPK-------------LAKLKAG-NNPYHC

TCDL--A-MF----I--E--------------E----T-------MQ----------HGD

-------L-----------EF--------AD-W-----PQNYKC-YHP-EN----LL---

-RNVIESY--NPGRLE-C-DVTL-----VVIIS--VTSTAVVVILSMLL----C--WKFD

V-PW-YIKAT---CQ---I-----IQ----S--R------------------H------R

S-R---K-------G---------------------IP----C---RTY---S----YHA

FVSYSSLDA---DWVRRELLFRL----EQC---S--------------PP----------

---Y---SICIHERDFM----------------P--GK-WIIDNII-ENIEKSRKVIFVL

SR------SFV-----------DSE-------WCNY-ELYFAHQRCIG--H-A-FDDVIL

VVKE--N-VSLDSL----------------------------------------------

--PNKFCKLRK-MLSTKTYLEWP----SE-------TNKQA-------FFWVQLKNIL--

--GKC------------TL-----------------------------------------

-------------------E-SVD-Q-------ER-----ISWTN---------DSAMSE

LPVASGSTQVYTVNLH-------------------------------------------

>TLR14_Sasal

------------------------------------------------------------

------------------------------------------------------------

------------------------------------------------------------

------------------------------------------------------------

------------------------------------------------------------

------------------------------------------------------------

----------MLQSSGRW------L--ALF--I---------LM----------------

---------------------------------------A--G--V------AH----V-

----------------------------------------------------AQSQI---

-PCT-VNE----------------------------NNVALCS-----------------

-----------G------LS--LS-E-------VP----T-N--L-----S-------T-

S--VVEL-------DLSFN-------K---L--R-----------SISKADFAA--Y-TN

L-----------TILNLG-YNS------------------I-FT--IER--DSFASNLLL

RKLNLF------N--------NS-L-TE-----------------------IP---S-LA

------I-SPL---KN---LKELEMS-NN-----L-YLNST------LS-------DV--

----FY-TFK------------S-------------------------------------

-------------LMVLSMG-G-S-EITWIR----------------KNDFLPLQ----N

ISLQKFALKTAS--SP--KH-YD----KG-----------------A---FAV----LNT

FS-LWCD-----FA--------LDTNPYVL-----------------------PLIL---

-GDL--RG----K----SIK---------------LLRL----RNLF----A-----N--

S--YY-------T---DDMD------LFS---GLAD-------T--GV--KELIL----F

RGK----------------FNENL---L-------------R-LLLLNV--QIS-NV-T-

-DL---------GLIA---------------------ID---------F--ARS-----L

------------N----L-S---A--S-----------N-VGI-----------------

SN----L---T--------LNE---LLLQDITN---------------------------

-------------------------------------------------PDILRFDWT--

-------------------------------FTWFS------------------------

---------------------------K--VSN-------LHIINVNFNYV-PCDA--WD

EMKN--VIVLNISNNR--------L-T-N------E--------------FIYN--PTCQ

N---------------PP--L-PK-LECFNASSNK-------------I--T-SL-KTIS

-LISLKW--PN-LQVVDLSH-N-G--------------------------I-G-------

--------SN----------N-E--SCS-W----------G-----P-------------

--AIIKLILHHNI--I----T-KI--------------IFQCLPT---------------

SLHYL----------DLS-------NSEL----DG--L-----DTN-----YF--T-KTT

----------------------------------------------------------N-

---------------------LSELFLSNNRIKFI-PSSWES---PNLLFLAVDGNSFGV

ITTG---------S-FD------------KMPK-------------LAKLKAG-NNPYHC

TCDL--A-MF----I--E--------------E----T-------MQ-------------

-------H-GDL-------EF--------AD-W-----PQNYKC-YHP-EN----LL---

-RNLIESY--NPGRLE-C-DVTL-------VVIISVTSTAVVVILSMLL----C--WKFD

I-PW-YIKAT---CQ---I-----IQ----S--R--------------------------

------H----R-SRK---------------G----IP----C---RTY---S----YHA

FVSYSSLDA---DWVRRELLFRL----EQC---S--------------PP----------

-Y-----SICIHERDFM----------------P--GK-WIIDNII-ENIEKSRKVIFVL

SR------SFV-----------DSE-------WCNY-ELYFAHQRCIG----HAFDDVIL

VVKE--N-VSLDSL----------------------------------------------

--PNKFCKLRK-MLSTKTYLEWP----SE-------TNKQA-------FFWVQLKNIL--

--GKC------------TL-----E-------SVDQ------------------------

-------------------ERMSWTN-------DS-----AMSELPVASGSTQVYTVNLH

-----------------------------------------------------------

>TLR14_Plwa

------------------------------------------------------------

------------------------------------------------------------

------------------------------------------------------------

------------------------------------------------------------

------------------------------------------------------------

------------------------------------------------------------

------------------------M--IQS--S---------------------------

--------------------------------G---R--W--L--E------LF----I-

---LI---AGVS-----------------S-------------VALSYL--SC--TV---

--KE-N------------------------------NV-ALCL-----------------

-----------G------LS--LI-E-------VP----K---DL-----S-------T-

S--IVEL-------DLSYN-------K---L--Q-----------SISKDDFSA--Y-TN

L-----------TILNLA-FNT------------------I-FR--IDH--NSFASNLLL

RKLNLF------N--------NS-L-TE-----------------------IP---S-LA

------I-SPL---KN---LEELEMS-NN-----L-YLNST------LS---D---V---

----FY-TFK------------S-------------------------------------

-------------LTVLSMG-G-S-EITWIR----------------ENDFLPLQ----N

ISLQKFALKTAS--SP--KH-YD----KG--A------------FAV----------LNT

FS-LWCD-----FA--------LDTNPYV-------L----------------PLIL---

-GDL--QG----K----SIR---------------LLRL----RNLF----A-----N--

S--YY---T-D-D---MDL-------FN----GLAD-------T--GV--KELIL----F

RGK----------------FNENL---LR-L----------L-LTNVQI----S-NV-T-

-DL---------CLIA---------------------ID---------F--ARS-----L

------------N----L-----S--A-------S--NV-G------------I-----S

NL----T------------LNK---LLLQDISN-PDI-----------------------

-------------------------------------------------LRF---DWT--

-------------------------------FTWFS------------------------

---------------------------K--VSN-------LHIINVNFNYV-PCDA--WD

EMKN--VIVLNISNNR--------L-T--------N--------------EFVY--NP--

-----TCQ-----N--PS--V-PK-LECFNASSNK-------------I--T-SL-KIIS

-LISINW--PN-LRVVDLSH-N-G--------------------------I-G---SN--

--------DE---------------SCS-W----------G-----P-------------

--AITKLILHHNI--I----K-KV--------------IFQCLPT---------------

SLHYL----------DLS-------NSEL----DG--L-----DTN-----YF--T-KTT

----------------------------------------------------------N-

---------------------LSELLLSNNRIKFI-PTIW--KS-PNLRFLAVDGNSFGV

ITKG---------S-FN------------NMPK-------------LAKLKAG-NNPYHC

TCEL--A-MF----I--E--------------E----T-------MQ----------YGD

-------L-----------EF--------AD-W-----PQNYKC-YHP-EY----LL---

-RNTIESY--DPGRLE-C-DITL-----VVIIS--VTSTAVVVIISMLL----C--WKFD

V-PW-YIKAT---CQ---I-----IQ----S--R------------------H------R

S-R---K-------G---------------------IP----S---RTY---S----YHA

FVSYSSSDA---DWVRRELLYRL----EQC---N--------------PA----------

---F---SICIHERDFM----------------P--GK-WIIDNII-ENIEKSRKVIFVL

SR------SFV-----------DSE-------WCNY-ELYFAHQRFIG--H-A-FDDVIL

VVKE--N-VSMDSL----------------------------------------------

--PSKFCKLRK-MLSTKTYLEWP----SE-------PNKQA-------FFWVQLKNVL--

--GKC------------TL-----------------------------------------

-------------------E-SAV-Q-------QT-----ISWTN---------DTAVSE

LPVGS------------------------------------------------------

>TLR14_Tywe

------------------------------------------------------------

------------------------------------------------------------

------------------------------------------------------------

------------------------------------------------------------

------------------------------------------------------------

------------------------------------------------------------

--------------MVQI------S--WKW--L---------EL----------------

---------------------------------------F--I--L------IA----G-

--------VGNI----------------------------------------AQSDT---

-PCT-VNE----------------------------NNVALCF-----------------

-----------G------LS--LI-E-------AP----K-K--L-----S-------T-

S--MVEL-------DLSYN-------K---L--Q-----------SINKDDFSA--Y-TN

L-----------TILNLA-FNI------------------I-FR--IDH--NSFASNLLL

RKLNLF------N--------NS-L-KE-----------------------IP---S-LA

------I-SPL---KN---LVELEMS-NN-----L-YLNST------LS-------DV--

----FY-TFK------------S-------------------------------------

-------------LTVLSMG-G-S-GITWIR----------------KNDFLPLQ----N

ISLQKFALKTAS--SP--KH-YD----KG-----------------A---FAV----LNT

FS-LWCD-----FA--------LDTNPYVL-----------------------PLIL---

-GDL--QG----K----SIR---------------LLRL----RNLF----A--------

---NS---Y-YTD---DMD-------LFK---GLAD-------T--GV--KELIL----Y

RGK----------------FNE-------------------N-LLRLLL--TNV-QI-S-

-NV---------TDLC---------------------LI---A---IDF--ARS-----L

------------N---------------------S--SA-SNV-----------------

----------------G--ISN---LTLNKLLL-QDI-----------------------

-------SN----------------------------------------PDILRFDWT--

-------------------------------FTWFS------------------------

---------------------------K--VSN-------LHIINVNFNYV-PCDA--WD

EMKN--VIVLNISNNR--------L-T-N------E--------------FVYN--PTCQ

N---------------PS--A-PK-LECFNASCNK-------------I--T-SL-KTIS

-LLSLKW--PN-LRVVDLSN-N-G--------------------------I-G-------

--------SN----------D-E--SCS-W----------G-----P-------------

--AITKLILHHNI--I--KKV-----------------IFQCLPT---------------

SLHYL----------DLS-------NSEL----DG--L-----DTN-----YF--I-KTT

----------------------------------------------------------N-

---------------------LSELLLSNNRIKFI-PASWES---PSLQFLAVDGNSFGV

ITKG---------S-FH------------NMPK-------------LAKLKAG-NNPYHC

TCDL--A-MF----I--E--------------E----T-------MQ-------------

-------Y-GDL-------EF--------AD-W-----PQNYKC-YHP-EH----LL---

-RNMIESY--NPGRLE-C-DITL-------VVIISVTSTAVVVIISMLL----C--WKFD

V-PW-YIKAT---CQ---I-----IQ----S--R--------------------------

--H--------R-SRK---------------G----IP----S---RTY---S----YHA

FVSYSSSDA---DWVRRELLYRL----EQC---N--------------PT----------

-F-----SICIHERDFM----------------P--GK-WIIDNII-ENIEKSRKVIFVL

SR------SFV-----------DSE-------WCNY-ELYFAHQRFIG----HAFDDVIL

VVKE--N-VSMDSL----------------------------------------------

--PSKFCKLRK-MLSTKTYLEWP----SE-------PNKQA-------FFWVQLKNVL--

--GKC------------TL-----E-------SAVQ------------------------

---------------------QTISW-------TN-----DTAMSELPVGS---------

-----------------------------------------------------------

>TLR18_Lach

------------------------------------------------------------

------------------------------------------------------------

------------------------------------------------------------

------------------------------------------------------------

------------------------------------------------------------

------------------------------------------------------------

--------------MEMH------Q--STK--Q---------------------------

--------------------------------N---W--F--L--I------SI----L-

---WA---AQIA-----------------T-------------ASMIFKHGAC--EI---

--RG-G------------------------------D--ASCK-----------------

-----------G------QS--LF-Q-------VP----R---NL-----P-------P-

S--LERI-------DLSYN-------K---L--Y-----------IIKNNDFAH--L-TK

L-----------RHLYLQ-FNN------------------I-SK--IEE--GAFASNVLL

EELDLF------N--------NS-L-TE-----------------------IP---S-AV

------L-KPL---TN---LRKLQLS-NN-----F-YSYST------LS---D---V---

----FS-TLW------------N-------------------------------------

-------------LTELFMG-G-P-LIKTVR----------------SHDFVPLQ----N

ITLEKFTLKTAS--SL--MT-YE----QG--A------------MLR----------VNT

QV-LWFD-----IA--------LDENPEI-------L----------------PQIL---

-KDL--QN----K----CFQ---------------LLRF----RNLF----E-----F--

K--YY---R-G-T---VDL-------FA----GLAG-------I--HV--KQLFF----Y

RGK----------------FNENL---MR-F----------A-LLNVQN----S-TI-K-

-DL---------VFAF---------------------VD---------F--ARS-----V

------------N----S-----S--T-------S--DT-A------------I-----T

DL----T------------LDH---LVLTDISN-PDI-----------------------

-------------------------------------------------LRF---DWT--

-------------------------------FTWFG------------------------

---------------------------K--ITN-------LSIINVNFNTV-PCDV--WE

EMKN--VETMNITNNR--------L-Q--------D--------------DFLF--NQ--

-----KCHY----K--DT--M-PK-LKRLLVSQNK-------------L--V-SL-KVIS

-QLSKEW--HQ-LYFIDLSY-N-Q--------------------------I-----NL--

--------EN------------K--LCN-W----------G-----S-------------

--NITRLILHHNT--I----T-SK--------------SFECLPT---------------

TLHYL----------DLS-------YTQL----ER--L-----DTD-----YF--L-HAI

----------------------------------------------------------H-

---------------------LKELFLSGNKIKFI-PSDW--TS-PSLRSLAVDGNSFGV

LSKG---------S-FQ------------NMPQ-------------LISLKAG-NNPYHC

TCDL--Y-MF----V--E--------------N----T-------VQ----------TKK

-------L-----------QL--------VN-W-----PKDYIC-YHP-ES----LL---

-DTDIAAF--SPARVQ-C-DIGL-----VVAIS--ASVTAAVVIVCMVL----C--WRFD

V-PW-YLKAT---CQ---V-----IQ----S--K------------------Y------R

S-R---K-------M---------------------DS----S---RSY---T----YHA

FISYSHSDA---EWVREELVPRL----ESC---N--------------PP----------

---Y---KICIHERDFM----------------P--GR-WIIDNII-ENIENSHKVIFVL

SH------NFV-----------NSE-------WCNY-ELYFAHQRAIG--Q-G-FEDVIL

VVKE--T-INPNSL----------------------------------------------

--PNKFCKLRK-MLSTKTYLEWP----AE-------PNRQP-------FFWMQLKNVL--

--GKV------------CQ-----------------------------------------

-------------------G-SES-E-------DD-------------------TSNNAP

TGVSMDCKLSEIESGGS---------------------------------LPD------

>TLR14_Anca

------------------------------------------------------------

------------------------------------------------------------

------------------------------------------------------------

------------------------------------------------------------

------------------------------------------------------------

------------------------------------------------------------

--------------MPCA------W--GSR--V---------------------------

--------------------------------P---L--L--V--L------LL----L-

---LV---PHSS-----------------R-------------APVPS---GC--HV---

--PS-T------------------------------GR-ASCR-----------------

-----------G------QN--LI-R-------VP----P---DL-----P-------E-

T--LEAL-------DLSYN-------K---I--R-----------RISAGDFAA--L-TR

L-----------KSLDLR-YND------------------L-SY--VDD--DAFASNLLL

EQLDLF------N--------NS-L-LT-----------------------IP---S-NA

------L-KDL---KR---LKVLSMS-NN-----L-YPRSA------LD---G---V---

----FG-GLK------------D-------------------------------------

-------------LEELSMG-G-P-AILTVS----------------SEDFQPLA----D

IPLKKFALKTAS--SL--TE-YQ----PG--A------------FAT----------LTT

ES-LWCD-----IA--------LDRNPGA-------L----------------PQML---

-RDL--WG----K----PLR---------------YLRF----RNLF----E-----F--

T--YY---N-H-P---ADI-------FS----NLER-------V--DA--RELVF----F

RGK----------------FNENL---LR-L----------I-LLNVQK----T-TV-Q-

-DL---------SFIS---------------------ID---------F--ARS-----P

------------Q----R-----A--K-------P--DV-G------------I-----A

NL----T------------LRS---LVLKDISN-PDI-----------------------

-------------------------------------------------LRF---DWT--

-------------------------------FTWFS------------------------

---------------------------G--VSF-------LSVLNVNFNFV-PCDA--WK

EMRN--VAALNISNNR--------L-L--------D--------------EYIY--NE--

-----LCSY----V--DI--V-PK-LEEFNVSYNR-------------V--T-RL-RTVS

-RLTAAW--PR-LSVLDLSH-N-Q--------------------------I-G---ND--

--------DN------------S--PCR-W----------S-----P-------------

--TLVWLGLAYNA--V---TT-ME--------------IFRCLPI---------------

TLRFL----------DLS-------HSQL----ER--L-----ELS-----YF--E-AAV

----------------------------------------------------------D-

---------------------LRELRLSGNKLKFI-PTAW--KG-PSLEVLTVDGNSFGA

IGRG---------S-FA------------NMPR-------------LSRLQAG-NNPYHC

VCEL--H-SF----L--R--------------E----A-------LS----------KGK

-------L-----------TI--------TD-W-----PENWTC-YHP-ER----LL---

-DTPVADY--APWVTE-C-DVTV-----VVAIT--VSVTAAVVGAATLL----C--WRFD

V-PW-YLRAT---FR---I-----VR----S--K------------------Y------R

S-G---H----P-SG---------------------QP----S---RRF---T----YHA

FVSYSHSDA---DWVRKELLLRL----EAT---R--------------PP----------

---Y---RLCVHERDFT----------------P--GR-WVIDNIV-DSIERSRKVVFVL

SR------SFV-----------DSD-------WCNY-ELYFAHQRAVG--L-G-FEDVVL

VVKE--A-VDPQAL----------------------------------------------

--PRKFCKLRK-LLSAKTYLEWP----AE-------PGRQA-------FFWAQLAAVL--

--GKA------------EK-----------------------------------------

-------------------G-VQR-P-------E--------------------GEAAAG

QTPEGQPGGGGSSAVDA---------------------------------PAS------

>TLR14_Alsi

------------------------------------------------------------

------------------------------------------------------------

------------------------------------------------------------

------------------------------------------------------------

------------------------------------------------------------

------------------------------------------------------------

----------------ML------T--ALL--A---------------------------

--------------------------------N---A--C--M--T------LG----L-

---LL---TGLV-----------------G-------------GAGAL---PC--ST---

--LS-D------------------------------TT-MICK-----------------

-----------G------QN--LV-S-------IP----R---DL-----P-------S-

S--IMFL-------DLSYN-------R---L--Q-----------EVPTAAFSG--L-PQ

L-----------RQLDLG-FNN------------------L-SR--LAA--DAFVTNVRL

EKLRLF------N--------NS-L-QE-----------------------IP---S-LA

------L-KPL---KN---LKWLDMS-NN-----L-YPRAT------LD---G---V---

----FS-TLR------------S-------------------------------------

-------------LQELSMG-G-P-LIQVLG----------------QQDFQPLR----N

ITLNKFSLKTAS--SL--LA-YE----EG--A------------FQP----------LNT

TV-LWCD-----IA--------LDQNPKA-------L----------------PMML---

-RDL--RG----S----PVR---------------HLRF----RNLF----E-----F--

T--YY---T-Q-S---LDI-------FT----GLAE-------V--EA--QELVF----Y

RGK----------------FNENL---LR-L----------V-LLNVQK----S-RI-R-

-DL---------SLVA---------------------ID---------F--ARS-----P

------------Q----W-----N--R-------S--EA-G------------L-----L

RP----R------------LDH---LLLKDISN-PDI-----------------------

-------------------------------------------------LRF---DWT--

-------------------------------FTWFS------------------------

---------------------------Q--VTN-------LSIINVNFNFV-PCDA--WD

EMRS--VVFLDISRNR--------L-Q--------D--------------AYLY--NQ--

-----ACHY----Q--DI--M-PK-MEQFLLAHNE-------------L--T-KL-DILA

-MITVTW--PR-LTHINASY-N-S--------------------------L-G---AQ--

--------QG---------------SCR-W----------N-----P-------------

--GLVWLSLHHNL--V----T-TE--------------AFQCLPT---------------

TLRYL----------DLS-------DSQV----DR--L-----EMD-----YF--A-RST

----------------------------------------------------------E-

---------------------LQELRLSGNKIKFI-PSEW--RC-PSLRVLAIDGNSFGV

ISKG---------S-FA------------NMPQ-------------LSKLQAN-NNPYHC

TCDL--Y-SF----L--Q--------------E----T-------RE----------RGR

-------L-----------TL--------VG-W-----PQGWTC-YHP-EA----LL---

-DTAVAAY--APGLVE-C-DVRV-----VVAIS--VAVTAVVVVACMVL----C--WRFD

V-PW-YLRAT---FH---I-----VQ----S--R------------------Y------R

A-R---R-------A---------------------QP----A---RTY---A----YHA

FISYSYSDA---DWVRQELLQRL----ESA---Q--------------PP----------

---Y---RVCIHERDFT----------------P--GR-WIIDNII-ENIENSYKIIFVL

SH------SFI-----------DSE-------WCNY-ELYFAHQRAVG--L-G-YEDVIL

VVKE--A-IDPQSL----------------------------------------------

--PHKFCKLRK-MLGTKTYLEWP----AE-------PSRQP-------FFWMQLRSVL--

--GKP------------GV-----------------------------------------

-------------------I-DEA-Q-------ET-----GSLASIEIE-----LPAAAA

TLPE-EETAVDVTMV-----------------------------------PSS------

>TLR14_Sppu

------------------------------------------------------------

------------------------------------------------------------

------------------------------------------------------------

------------------------------------------------------------

------------------------------------------------------------

------------------------------------------------------------

------------------------M--SPL--P---------------------------

--------------------------------G---T--W--L--V------LC----V-

---TT---MGLA-----------------G-------------AVDAS---PC--RI---

--SE-D------------------------------LKAAECQ-----------------

-----------G------QN--LV-R-------VP----W---DL-----P-------H-

T--LQRL-------DLSYN-------K---L--Q-----------EISERDFSA--L-TQ

L-----------RGLDLG-YNN------------------L-SR--IAA--NSFASNVLL

EDLSLF------N--------NS-L-YQ-----------------------IP---S-QA

------L-KPL---KN---LKRLFMS-NN-----L-YPRST------LD---G---V---

----FS-TLR------------K-------------------------------------

-------------LQEVSMG-G-P-AIQEVG----------------AQDFFPLK----D

TPLQKFSLKTAS--SL--TK-YQ----QG--A------------FSL----------LNT

TA-LWFD-----IS--------LDKNAEA-------L----------------PVML---

-RDL--RG----K----PVR---------------YLRF----RNLF----E-----F--

T--YY---M-Q-S---MDL-------FS----GLAE-------V--EA--NELVF----Y

RGK----------------FNENL---LR-L----------A-LLNMQK----S-RI-Q-

-DL---------SLIA---------------------ID---------F--ARS-----P

------------E----W-----E--Q-------P--ET-G------------I-----T

NL----T------------LNR---LLLQDISN-PDI-----------------------

-------------------------------------------------LRF---DWT--

-------------------------------FTWFS------------------------

---------------------------G--ITY-------LFILNVNFNFV-PCDA--WT

EMRN--VVALDVSNNQ--------L-K--------D--------------NYIY--NQ--

-----ACHY----Q--DV--M-PK-LGLFNLSHNE-------------L--T-SL-GKVA

-KLTFSW--HG-LTQLDLSY-N-Q--------------------------I-G---SL--

--------KD---------------SCV-W----------G-----P-------------

--ALTWLALSNNV--V----T-IV--------------TFKCLPT---------------

TLHFL----------DLS-------YSQL----DR--L-----EMS-----YF--E-RAV

----------------------------------------------------------D-

---------------------LQELWLSGNKIKFI-PSEW--RC-PSLRALMVDGNSFGV

ISKG---------S-FI------------NMPQ-------------LTKLKAG-NNPYHC

TCDL--H-GF----L--Q--------------E----T-------LR----------QGK

-------L-----------TL--------LD-W-----PHGWTC-YHP-EF----LL---

-DTEVAAY--SPGVTE-C-DVRV-----VVAIS--VSVTAILVIACMVL----C--WKLD

L-LW-YIRAT---FR---I-----VR----S--K------------------Y------R

A-R---H-------A---------------------QP----S---RAF---V----YHA

FISYSCSDT---DWVRRELLQHL----ENS---N--------------PP----------

---Y---RICIHERDFT----------------P--GR-WIIDNII-ENIENSHKVIFVL

SH------SFV-----------DSD-------WCNY-ELYFAQQRAVG--L-G-FEDVVL

VVKE--T-IDPRTL----------------------------------------------

--PNKFFKLRK-MLSSKTYLEWP----SE-------PSRQP-------FFWMQLKSLL--

--GKV------------EV-----------------------------------------

-------------------I-ETD-R-------DT-----VSPLDTELW-----ASRTPE

GPVELDGPPVESVVVHV---------------------------------PPS------

>TLR14_Pesi

------------------------------------------------------------

------------------------------------------------------------

------------------------------------------------------------

------------------------------------------------------------

------------------------------------------------------------

------------------------------------------------------------

------------------------M--PPL--A---------------------------

--------------------------------G---T--C--L--A------LC----V-

---VM---VGLA-----------------V-------------RAD-----PC--LF---

--TA-D------------------------------NTTAMCK-----------------

-----------G------RN--LD-R-------VP----R---HL-----P-------R-

T--LLRL-------DLSYN-------R---L--Y-----------EISPGDFAE--L-TQ

L-----------QSLDLS-YNN------------------L-SH--IAA--GAFAANVLL

EELSLF------N--------NS-L-HQ-----------------------IP---A-LA

------L-KPL---RK---LRRLEMS-NN-----L-YCHST------LD---K---A---

----FS-TLR------------N-------------------------------------

-------------LQELSMG-G-P-LIQTVG----------------KGDFLPLK----E

IALQKFALKTAS--SL--LE-YQ----EG--A------------FSV----------LNT

TS-LWFD-----IA--------LDKNPNA-------L----------------PVIL---

-RDL--KG----K----PLL---------------YLRF----RNLF----E-----F--

T--YY---T-D-A---ADL-------FS----GLAE-------V--RA--EKLVF----Y

RGK----------------FNENL---LR-L----------P--------------I-R-

-DL---------SLMA---------------------ID---------F--ARS-----P

------------Q----W-----K--R-------P--EA-G------------I-----A

NL----T------------LDS---LLLQDISN-PDI-----------------------

-------------------------------------------------LRF---DWT--

-------------------------------FTWFS------------------------

---------------------------G--VTN-------LSILNVNFNFV-PCDA--WD

EMRN--VVALDVSNNR--------L-T--------D--------------AYIY--NQ--

-----RCDY----Q--DV--M-PK-LERFFVARNE-------------L--T-SL-SVLA

-KLTANW--PR-LTHINASH-N-Q--------------------------L-G---SL--

--------KE------------E--TCQ-W----------N-----S-------------

--GLVWLALDHNT--V----T-ME--------------IFKCLPI---------------

TLLYL----------DLS-------HSQL----DR--L-----EMD-----FF--D-RCR

----------------------------------------------------------H-

---------------------LQELKLSGNKIKFI-PSVW--KC-PSLRTLAVDGNSFGI

ISKG---------S-FV------------HMPQ-------------LASLQAG-NNPYHC

TCDL--Y-GF----L--Q--------------E----M-------WR----------KGK

-------L-----------TL--------KD-W-----PENWKC-YHP-ES----LL---

-DMRVADY--APGLTE-C-DVRV-----VVAIS--VSVTAVVIIAIMVL----C--WRFD

V-PW-YLHAT---FR---I-----IR----S--K------------------S------R

A-R---H-------A---------------------TP----A---RAY---A----YHA

FISYSWSDA---DWVRQELLRRL----ETS---T--------------PP----------

---Y---RICIHERDFT----------------P--GK-WIIDNII-ENIENSCKVIFVL

SR------SFV-----------DSE-------WCNY-ELYFAHQRAGG--L-G-YEDVIL

VVLE--S-IDPRSL----------------------------------------------

--PNKFCKLRK-MLSTKTYLEWP----SE-------PSRQP-------FFWIQLRNVL--

--GKP------------GA-----------------------------------------

-------------------I-DTS-Q-------DQ-----VSLTSMKGR-----SEVAVS

DLAE-EEIAIDADSSSVLHLLRIGVKALGLCWWDVKSVALVPGQLSSSPFPPD------

>TLR14_Chmy

------------------------------------------------------------

------------------------------------------------------------

------------------------------------------------------------

------------------------------------------------------------

------------------------------------------------------------

------------------------------------------------------------

------------------------M--RPL--A---------------------------

--------------------------------G---M--C--L--A------LC----L-

---VI---VGLA-----------------D-------------GAD-----PC--LV---

--SA-N------------------------------NEIATCK-----------------

-----------G------QN--LN-R-------VP----Q---HL-----P-------C-

T--LLRL-------DLSYN-------K---L--K-----------EITAGDFSA--L-TR

L-----------QSLDLG-YNN------------------I-SH--IVA--HAFASNVQL

EELSLF------N--------NS-L-RQ-----------------------IP---S-LA

------L-KPL---RK---LRRLDMS-NN-----F-YLHST------LD---E---V---

----FS-TLR------------N-------------------------------------

-------------LQEFSMG-G-S-LIQTVS----------------KWDFLPLR----D

IALQKFALKTAS--SL--LE-YQ----EG--A------------FSV----------LNT

TA-LWCD-----IA--------LDKNAQA-------L----------------PMML---

-RDL--RG----K----PLQ---------------YLRF----RNLF----E-----F--

T--YY---T-E-S---TDL-------FS----GLAE-------V--RA--SKLVF----Y

RGK----------------FNEKR---V--------------------------------

-----------------------------------------------------P-----S

------------Q----G-----T--R-------S--EA-G------------I-----A

NL----T------------LDS---LLLKDISN-PDI-----------------------

-------------------------------------------------LRF---DWT--

-------------------------------FTWFS------------------------

---------------------------G--VAN-------LSILNINFNFV-PCDT--WD

EMRN--VVTLDISNNR--------L-R--------D--------------AYIY--NQ--

-----GCNY----Q--DV--M-PK-LERFLMAKNE-------------L--T-SL-AVVS

-KLTANW--PR-LTHINASH-N-R--------------------------I-G---GL--

--------KE------------T--ACR-W----------N-----P-------------

--GLVWLALDHNT--V----T-ME--------------IFKCLPI---------------

TLHYL----------DLS-------SSEL----DR--L-----EMS-----YF--V-RCQ

----------------------------------------------------------D-

---------------------LQELKLSGNKIKFI-PSEW--RC-PSLRILAMDGNSFGV

ISKG---------S-FV------------NMPE-------------LTSLKAG-NNPYHC

TCDL--Y-GF----L--Q--------------E----T-------RR----------KGK

-------L-----------TL--------LD-W-----PGGWTC-FHP-ES----LL---

-DVGVAAY--TPGLME-C-DVRV-----VVAIS--VSVTAAVIIATMVL----C--WRFD

V-LW-YLQAT---YR---I-----VR----S--K------------------Y------R

A-R---H-------A---------------------HP----T---RAY---A----YHA

FISYSCSDA---DWVRQELLQRL----ESS---S--------------PP----------

---Y---RICIHERDFT----------------P--GK-WIIDNII-ENIESSYKIIFVL

SR------SFV-----------DSE-------WCNY-ELYFAHQRAVG--L-G-YEDVIL

VVKE--A-IDPQSL----------------------------------------------

--PHKFCKLRK-MLSTKTYLEWP----SE-------PSRQP-------FFWVQLRNVL--

--GKP------------GA-----------------------------------------

-------------------A-EPS-Q-------DR-----VSLASVELG-----SDESVN

NPAE-EETAIDTVAS-----------------------------------PAS------

>TLR14_Chpi

------------------------------------------------------------

------------------------------------------------------------

------------------------------------------------------------

------------------------------------------------------------

------------------------------------------------------------

------------------------------------------------------------

------------------------M--RPL--A---------------------------

--------------------------------G---M--C--I--A------LC----V-

---VI---VGLA-----------------D-------------GAD-----PC--LV---

--TA-D------------------------------NKVATCK-----------------

-----------G------QN--LN-R-------VP----Q---HL-----P-------R-

T--LLRL-------DLSYN-------R---L--K-----------EIAAGDFSA--L-TQ

L-----------QSLDLG-YNN------------------I-SR--ISA--DAFASNVLL

EELSLF------N--------NS-L-RW-----------------------IP---S-PA

------L-KPL---RK---LRRLEMS-NN-----L-YLRST------LD---E---V---

----FS-TLR------------N-------------------------------------

-------------LQEFSMG-G-S-LIQTVG----------------KWDFLPLK----N

IALQKFALKTAS--SL--LK-YQ----NG--A------------FSV----------LNT

TA-LWCD-----IA--------LDKNPKA-------L----------------PMIL---

-RDL--RG----K----PLQ---------------YLRF----RNLF----E-----F--

T--YY---T-E-P---ADL-------FS----GLAE-------V--QA--SKLVF----Y

RGK----------------FNENL---LR-L----------A-LLNIQK----S-RI-R-

-DL---------SLMS---------------------ID---------F--ARS-----S

------------Q----W-----N--R-------S--ET-G------------I-----T

NL----T------------LGS---LLLQDISN-PDI-----------------------

-------------------------------------------------LRF---DWT--

-------------------------------FTWFS------------------------

---------------------------G--VAN-------LSILNVNFNFV-PCDT--WD

EMRN--VVTLDVSNNR--------L-K--------D--------------AYIY--NQ--

-----GCNY----Q--DV--L-PK-LERFLMAKNE-------------L--T-SL-GIVA

-KLTANW--PR-LTHINASH-N-H--------------------------I-G---GL--

--------KE------------T--ACQ-W----------N-----P-------------

--GLVWLALDHNT--V----T-ME--------------IFKCLPI---------------

TLHYL----------DLS-------YSEL----DR--L-----EMS-----YF--V-RCQ

----------------------------------------------------------D-

---------------------LQELKLSGNKIKFI-PSEW--RC-PSLRILAMDGNSFGV

ISEG---------S-FV------------NMPE-------------LTSLKAG-NNPYHC

TCDL--Y-GF----L--Q--------------E----T-------RR----------KGK

-------L-----------TL--------LD-W-----PEGWTC-YHP-ES----LL---

-DMGVASY--TPGLTE-C-DVRV-----VVAIS--VSITAVVIIATMVL----C--WKFD

V-LW-YLQAT---YR---I-----IR----S--K------------------Y------R

A-R---H-------A---------------------QP----A---RAY---A----YHA

FISYSCSDA---DWVRQELLQRL----ESS---S--------------PP----------

---Y---RICIHERDFT----------------P--GK-WIIDNII-ENIESSYKVIFVL

SR------SFV-----------DSE-------WCNY-ELYFAHQRAVG--L-G-YEDVIL

VVKE--A-IDPQSL----------------------------------------------

--PNKFCKLRK-MLSTKTYLEWP----SE-------PSRQP-------FFWIQLRNVL--

--GKP------------GA-----------------------------------------

-------------------A-EPG-H-------DR-----VSLASVELG-----SGEVVS

SPAE-EETAIDAVAS-----------------------------------PAS------

>TLR14_Goag

------------------------------------------------------------

------------------------------------------------------------

------------------------------------------------------------

------------------------------------------------------------

------------------------------------------------------------

------------------------------------------------------------

--------------MCPL------A--GIC--I---------AL----------------

---------------------------------------C--V--V------IV----G-

----------------------------------------------------LAEGD--H

-PCL-V---------------------------TTDNKTATCK-----------------

-----------G------QN--LN-C-------VP----Q-H--L-----P-------R-

T--LLRL-------DLSYN-------R---L--K-----------EITSGDFSV--L-TQ

L-----------QSLDLG-YNN------------------I-SR--IAE--DAFTSNVLL

EELSLF------N--------NS-L-RR-----------------------IP---S-PA

------L-KPL---RK---LRRLEMS-NN-----L-YLCST------LD-------EV--

----FS-TLR------------N-------------------------------------

-------------LQEFSMG-G-S-LIQTVG----------------KWDFFPLK----D

IALQKFALKTAS--SL--LE-YQ----KG-----------------A---FSV----LNT

TA-LWCD-----MA--------LDKNPKAL-----------------------PMIL---

-RDL--RG----K----PLQ---------------YLRF----RNLF----E--------

---FT---Y-YTE---SAD-------VFS---GLAE-------V--RA--SQLVF----Y

RGK----------------FNENL---L-------------R-LALLNI--QKS-RI-R-

-DL---------SLMA---------------------ID---------F--ARS-----S

------------Q----W-N---R--S-----------E-AGI-----------------

AN----L---T--------LDS---LLLQDISN---------------------------

-------------------------------------------------PDILRFDWT--

-------------------------------FTWFS------------------------

---------------------------G--VAN-------LSILNVNFNFV-PCDT--WD

EMRN--VVTLDVSNNR--------L-K-D------A------YIYNQ-G-CNYQ------

----------------GF--M-PK-LERFLVAKNE-------------L--T-SL-GVVA

-KLTASW--PR-LTHINASH-N-H--------------------------I-G---GL--

---------K----------E-T--ACQ-W----------N-----P-------------

--GLVWLVLDHNSVTM----E-----------------IFKCLPI---------------

TLHYL----------DLS-------YSEL----DR--L-----EMS-----YF--V-RCQ

----------------------------------------------------------D-

---------------------LQELKLSGNKIKFI-PSEWRC---PSLRILAMDGNSFGV

ISEG---------S-FV------------NMPE-------------LTSLKAG-NNPYHC

TCDL--Y-GF----L--Q--------------E----T-------RR-------------

-------K-GKL-------TL--------LD-W-----PGDWTC-YHP-ES----LL---

-DMGVAAY--TPGLTE-C-DVRV-----VVAIS--VSVTAAVIIASMVL----C--WRFD

V-LW-YLQAT---YR---I-----VR----S--K--------------------------

------Y----R-ARH---------------A----HP----A---RAY---A----YHA

FISYSCSDA---DWVRQELLRRL----ESS---S--------------PP----------

-Y-----RICIHERDFT----------------P--GK-WIIDNII-ENIENSYKVIFVL

SR------SFV-----------DSE-------WCNY-ELYFAHQRAIG--L-G-YEDVIL

VVKE--A-IDPKSL----------------------------------------------

--PNKFCKLRK-MLSTKTYLEWP----SE-------PSRQP-------FFWIQLRNVL--

--GKP------------GA-----------------------------------------

-------------------AERSQDR-------VS-----LASVELGSDEVVNSPAEEET

AIDAVAGPAS-------------------------------------------------

>TLR14_Povi

------------------------------------------------------------

------------------------------------------------------------

------------------------------------------------------------

------------------------------------------------------------

------------------------------------------------------------

------------------------------------------------------------

----------------ME------L--AS-------------------------------

---------------------------------------F--V--M------LC----L-

---LF---LRAA-----------------E-------------QAEAF---VC--QI---

--FS-P------------------------------QR-ASCR-----------------

-----------G------QN--LV-K-------VP----S---NL-----S-------S-

A--LEFL-------DLSYN-------K---I--K-----------SVTSGDLSA--L-IR

L-----------KGLDLG-YNN------------------I-VS--VAR--DAFSANVLL

EQLILF------N--------NS-L-RE-----------------------IP---S-QA

------L-KPL---KN---LKVLSMS-NN-----L-YPRAT------LD---A---T---

----FG-ALK------------N-------------------------------------

-------------LEELSMG-G-P-AIATVG----------------REDFLPLE----A

VPLKKFALKTAS--SL--RE-YQ----RG--A------------FSK----------LNT

SA-LWCD-----IA--------LDNYAEA-------L----------------PLIL---

-RDL--RG----K----PLK---------------YLRF----RNLF----K-----F--

T--YY---T-D-A---TDL-------FS----SLAE-------V--DV--EELVF----F

RGK----------------FNENL---LR-L----------V-LQNVQK----S-PI-R-

-DL---------SFIA---------------------ID---------F--ARS-----P

------------E----W-----K--P-------T--EA-G------------V-----A

NL----T------------LRR---LVLKDISN-PDI-----------------------

-------------------------------------------------LRF---DWT--

-------------------------------FTWFA------------------------

---------------------------G--VTY-------LSILNVNFNFV-PCDA--WE

EMRN--LVALDVSNNR--------L-T--------D--------------EYIY--NE--

-----ACLY----Q--GI--V-PH-LEQFNLSYNA-------------L--A-RL-GTVA

-RLTAHW--PR-LSALDLRH-N-Q--------------------------I-G---SA--

--------ND------------L--PCT-W----------T-----R-------------

--SLVWLTLAYNA--V----T-TD--------------IFRCLPT---------------

TLSFL----------DLS-------HSQL----ER--L-----EMG-----YF--E-AAV

----------------------------------------------------------D-

---------------------LQELLLTGNKIKFI-PTEW--RC-PNLKLLAVDGNSFGV

IGRG---------S-FV------------NMPR-------------LTQLKAG-NNPYQC

VCDL--H-RF----L--Q--------------E----T-------FG----------NRK

-------L-----------TL--------LD-W-----PHGWTC-YHP-EP----LL---

-DTPVAAY--NPWVTE-C-DVTV-----VVAIA--VSITATVIVVCMVL----C--WRFD

L-PW-YLKAT---FQ---I-----VR----S--K------------------Y------R

A-G---R-------S---------------------QG----S---RPF---T----YHA

FISYSFSDA---EWVRRELLQKL----EAS---S--------------PP----------

---Y---HVCIHERDFT----------------P--GR-WIIDNII-DNIEKSHKVIFVL

SR------SFV-----------DSE-------WCNY-ELYFAHQRAVG--L-G-FEDVVL

VVKE--A-IDPQAL----------------------------------------------

--PHKFYKLRK-MLSAKTYLEWP----AE-------PTRQA-------FFWLQLTSVL--

--GKP------------AR-----------------------------------------

-------------------V-HME-P--------K-----DHPEHADPR-----PGGVAA

SHRETGSFTEGNSATDV---------------------------------PLEVRKSQT

>TLR14_Geja

------------------------------------------------------------

------------------------------------------------------------

------------------------------------------------------------

------------------------------------------------------------

------------------------------------------------------------

------------------------------------------------------------

----------------ME------M--PQL--T---------------------------

--------------------------------A---R--W--L--A------LL----M-

---VI---VGLE-----------------E-------------QSKAS---LC--YI---

--TE-D------------------------------KFKASCQ-----------------

-----------G------KS--LF-S-------VP----Q---DL-----P-------V-

T--LQLL-------DLSYN-------K---I--R-----------EITSGDFSS--F-TQ

L-----------KGLDLS-YNN------------------I-VF--IAN--DSFSSNILL

EQLNLF------N--------NS-L-GE-----------------------IP---S-QA

------F-KPL---RN---LRELFIS-NN-----L-YPHST------LD---G---V---

----FS-TLK------------K-------------------------------------

-------------LEVLSMG-G-P-AIQTVG----------------SQDFLSIK----E

IPLKQFALKTAF--NL--LE-YQ----TG--A------------FSK----------LNT

TA-LWCD-----IS--------LDKNTKA-------L----------------PLML---

-QDL--RG----K----PLR---------------YLRF----RKLF----E-----F--

T--YY---M-D-A---TDL-------FS----GLAE-------L--EA--EELVF----F

RGK----------------FNENL---LR-L----------A-LLNVQK----S-RI-Q-

-DL---------SFIA---------------------ID---------F--ARS-----P

------------E----W-----K--P-------P--EA-G------------I-----A

NL----T------------LRH---LVLQDINN-PDI-----------------------

-------------------------------------------------LRF---DWT--

-------------------------------FTWFA------------------------

---------------------------G--VTY-------LSILNVNFNVV-PCDA--WG

EMRN--VVALNVSRNR--------L-Q--------N--------------GYIY--NQ--

-----ACVY----Q--DI--L-PK-LEDFNMTRNE-------------L--T-SL-KTMA

-TLTSSW--AQ-LSKLDLSH-N-L--------------------------I-G---DL--

--------NE------------F--PCT-W----------T-----P-------------

--SLVWLGLAYNA--V----T-VE--------------IFKCLPT---------------

TLHFL----------DMS-------DSGL----ER--L-----DMG-----YF--E-LAV

----------------------------------------------------------D-

---------------------LQELLLSGNKIKFI-PTEW--RC-PNLRILHVDGNSFGA

ISKG---------S-FG------------NMPH-------------LAYLKAG-NNPYHC

VCDL--Y-GF----L--Q--------------E----T-------LR----------DGK

-------L-----------TL--------LD-W-----PNEWTC-YHP-ER----LL---

-DTSVAAY--APRVTE-C-DVTV-----VVAIA--VSITATVVIVCMVL----C--WKFD

I-PW-YLKAT---FR---I-----VR----S--K------------------Y------R

A-S---H-------S---------------------QP----S---KPF---V----YHA

FISYSCSDA---EWVRRELLQRL----EAS---T--------------PP----------

---Y---RVCIHERDFT----------------P--GR-WIIDNII-ENIENSRKVIFVL

SR------NFV-----------DSE-------WCNY-ELYFAHQRAVG--L-G-FEDVVL

VVKE--P-IDPQAL----------------------------------------------

--PNKFCKLRK-MLSTKTYLEWP----LE-------PSRQP-------FFWLQLKSVL--

--GKA------------EM-----------------------------------------

-------------------V-NVD-P-------SN-----GDPNNAVLDDTEPISDGVTD

STFELNDLSEENVTVDT---------------------------------QMS------

>TLR14_Pybi

------------------------------------------------------------

------------------------------------------------------------

------------------------------------------------------------

------------------------------------------------------------

------------------------------------------------------------

------------------------------------------------------------

----------------MG------P--SPF--A---------------------------

--------------------------------D---L--F--V--V------LR----L-

---VF---LGLA-----------------Q-------------HPTTS---PC--QI---

--VS-N------------------------------TI-APCR-----------------

-----------G------QN--LL-R-------VP----R---DL-----P-------R-

T--LEKL-------DLSYN-------K---I--E-----------HIGPADFSS--L-TQ

L-----------KELNLG-YNH------------------I-FS--IAN--DSFASNILL

EKLSLF------N--------NS-L-GE-----------------------IP---S-LA

------L-KPL---KK---LTALSIS-NN-----I-YHLSR------LD---G---V---

----FS-SLK------------N-------------------------------------

-------------LEEFSLG-G-P-VIYKVG----------------REDFVPLK----D

IPLKKFSLKTAS--SL--ME-YQ----QG--A------------FSK----------LST

RE-FWCD-----IA--------LDKNPEI-------L----------------RLML---

-RDL--KG----K----QLQ---------------YLRF----RNLF----E-----F--

T--YY---T-D-S---VDL-------FA----GLPE-------V--EV--EELVF----Y

RGK----------------FNEHL---LR-L----------V-LLNVQK----S-HV-R-

-DL---------SFIA---------------------ID---------F--ARS-----P

------------K----Q-----K--T-------P--EA-G------------I-----A

NL----T------------LRH---LLLKDISN-PDI-----------------------

-------------------------------------------------LRF---DWT--

-------------------------------FTWFA------------------------

---------------------------D--VTY-------LSILNVNFNFV-PCDA--WG

EMRN--VVALNVSGNR--------L-L--------D--------------EYIY--NE--

-----GCLY----Q--GI--M-PK-LEQFNLSSNE-------------L--T-HL-STVS

-KLTSSW--PR-LSKLDLSH-N-R--------------------------I-G---HS--

--------KD------------L--PCV-W----------S-----P-------------

--SLVWLDLAYNV--V----T-VE--------------IFECLPT---------------

RLQFL----------DLS-------YSQL----ES--L-----DMN-----YF--E-VAV

----------------------------------------------------------D-

---------------------LQELWLSGNKIKFI-PTKW--RC-PNLRVLAVDSNSFGV

ISKG---------S-FV------------NMPQ-------------LTQLKAG-NNPYHC

ICEL--Y-SF----L--Q--------------E----T-------LK----------NGK

-------L-----------QI--------VD-W-----PSGWIC-YHP-EP----LR---

-DTAVVAY--APRVTQ-C-DVMV-----VVAIA--VSITAIVVILTMVL----C--WWFN

V-PW-YLKAT---FQ---I-----LH----S--R------------------Y------R

A-S---R-------S---------------------QP----L---REF---V----YHA

FISYSCSDA---EWVRRELLQRL----EAS---T--------------PP----------

---Y---RVCIHERDFT----------------P--GR-WIIDNII-ENIENSRKIIFVL

SR------SFV-----------DSD-------WCNY-ELYFAHQRAVG--L-S-FEDVVL

VVKE--A-IDPQAL----------------------------------------------

--PNKFCKLRK-MLSKKTYLEWP----PE-------PNRQA-------FFWVQLTSVL--

--GKA------------EA-----------------------------------------

-------------------I-GRN-P--------K-----EDPVNSAWG-----TDGLAD

GSLEMDNAAHAD---DV---------------------------------PGC------

>TLR14_Prmu

------------------------------------------------------------

------------------------------------------------------------

------------------------------------------------------------

------------------------------------------------------------

------------------------------------------------------------

------------------------------------------------------------

------------------------MGPSPLADL---------------------------

---------------------------------------FLVLCLV------LL--GWA-

-------------------------------------------------------QRAVP

SPCQ-IVS----------------------------NTVAPCR-----------------

-----------G------QN--LL-Q-------VP------Q-DL-----P-------S-

T--LEKL-------DLSYN-------K---I--E-----------QIDSADFSS--L-TQ

L-----------KELDLG-YNH------------------I-FS--IAN--DSFASNILL

EKLSLF------N--------NS-L-GE-----------------------IP---S-LA

------L-TPL---KK---LTALSIS-NN-----L-YPLSR------LD-------GV--

----FS-SLK------------K-------------------------------------

-------------LEELSLG-G-P-VIYKVG----------------SEDFAPLK----D

LPLKKFFLKTAS--SL--TE-YQ----QG--A------------FSK----------LNT

TE-FWCD-----IA--------LDRNPEAL-----------------------PLMF---

-SDL-KGK---------QLQ---------------YLRF----RNLF----------EFT

YYTNS-----------MDL-------FA----SLPE-------V--EV--EKLVF----Y

RGK----------------FNENL-------------------LRLVLLNVQKS-RV-R-

-DL---------SFIA---------------------ID---------F--ARS-----P

------------K----WKP--------------PEAGI-ANL-----------------

------T------------LRH---LLLKDISN---------------------------
[truncated: 4,971,092 more chars]
