# Supplementary material for: Evolutionary History of the Toll-Like Receptor Gene Family across Vertebrates
Source: Genome Biol Evol. 2019 Dec 4;12(1):3615–34. doi: 10.1093/gbe/evz266 (PMC6946030; doi:10.1093/gbe/evz266)
Supplement: evz266_Supplementary_Data [file evz266_supplementary_data.zip › Suppl material legends.docx]

**Supplementary data**

Fig. S1. Schematic presentation of the structural comparison of single cysteine cluster TLRs (sccTLRs), multiple cysteine cluster TLRs (mccTLRs), and short or truncated TLR-like proteins. The figure is adapted from Leulier and Lemaitre (2008). The mccTLRs and sccTLRs share a prototypical structure with an extracellular domain (ECD) containing multiple leucine-rich motifs, a short transmembrane domain (TM), and an intracellular Toll/interleukin-1 receptor domain (TIR). The mccTLR ectodomain has two cysteine clusters at the C-terminus of the LRR (LRRCT); the sccTLR ectodomain has only one LRRCT domain (Brennan and Gilmore 2018).

Fig. S2. Syntenic comparison of vertebrate TLRs and surrounding genes. Transcription orientation is indicated by triangle direction.

Fig. S3. Putative patterns of TLR gene family evolution in vertebrates. TLR gene gain (red font) and loss (blue font) events are shown along the branches. Significant differences in the numbers of TLR genes for vertebrate species were detected between amphibian and fish and between amphibian and amniote (two-tailed Wilcoxon test, W = 189, *P* = 0.0103; W = 446, *P* = 5.56e-05). Asterisks indicate *P* value: * *P* < 0.05, ** *P* < 0.01.

Table S1. Metazoan species list and TLR homologs identified per species

Table S2. Vertebrate species list and TLR genes identified from public genome data

Table S3. Summary of amphibian transcriptomic data used for TLR identification and the TLR genes identifed

Table S4. Mean ω (d*N*/d*S*) values for full-length (FL), extracellular (ECD), transmembrane (TM) and intracellular (ICD) domain regions of each vertebrate TLR gene

Table S5. Positively selected sites across vertebrate TLR genes, as inferred by PAML M7/M8 and FUBAR methods

Table S6. Extracellular (ECD), transmembrane (TM) and intracellular (ICD) amino acid ranges of vertebrate reference TLR genes

Table S7. Positively selected sites from TLR genes across different vertebrate clades, as inferred by the branch-site model of the PAML method

Table S8. Phylogenetic model selection for different datasets of TLR protein sequence alignments

Supplementary text S1. Codon alignments for sccTLRs and mccTLRs of metazoan species

Supplementary text S2. Vertebrate TLR amino acid alignments

**Literature Cited**

Akaike H 1974. A new look at the statistical model identification. IEEE Transactions on Automatic Control 19: 716-723. doi: 10.1109/tac.1974.1100705

Alcaide M, Edwards SV 2011. Molecular evolution of the toll-like receptor multigene family in birds. Mol Biol Evol 28: 1703-1715. doi: 10.1093/molbev/msq351

Alexopoulou L, Holt AC, Medzhitov R, Flavell RA 2001. Recognition of double-stranded RNA and activation of NF-kappaB by Toll-like receptor 3. Nature 413: 732-738. doi: 10.1038/35099560

Altekar G, Dwarkadas S, Huelsenbeck JP, Ronquist F 2004. Parallel Metropolis coupled Markov chain Monte Carlo for Bayesian phylogenetic inference. Bioinformatics 20: 407-415. doi: 10.1093/bioinformatics/btg427

Altizer S, Bartel R, Han BA 2011. Animal migration and infectious disease risk. Science 331: 296-302. doi: 10.1126/science.1194694

Altschul SF, Gish W, Miller W, Myers EW, Lipman DJ 1990. Basic local alignment search tool. Journal of Molecular Biology 215: 403-410. doi: 10.1016/s0022-2836(05)80360-2

Andersen-Nissen E, Smith KD, Bonneau R, Strong RK, Aderem A 2007. A conserved surface on Toll-like receptor 5 recognizes bacterial flagellin. J Exp Med 204: 393-403. doi: 10.1084/jem.20061400

Anderson KV, Bokla L, Nüsslein-Volhard C 1985. Establishment of dorsal-ventral polarity in the drosophila embryo: The induction of polarity by the Toll gene product. Cell 42: 791-798. doi: 10.1016/0092-8674(85)90275-2

Areal H, Abrantes J, Esteves PJ 2011. Signatures of positive selection in Toll-like receptor (TLR) genes in mammals. BMC Evol Biol 11: 368. doi: 10.1186/1471-2148-11-368

Babik W, et al. 2014. Constraint and adaptation in newt toll-like receptor genes. Genome Biol Evol 7: 81-95. doi: 10.1093/gbe/evu266

Bainova H, et al. 2014. First evidence of independent pseudogenization of toll-like receptor 5 in passerine birds. Dev Comp Immunol 45: 151-155. doi: 10.1016/j.dci.2014.02.010

Barreiro LB, et al. 2009. Evolutionary dynamics of human Toll-like receptors and their different contributions to host defense. PLoS Genet 5: e1000562. doi: 10.1371/journal.pgen.1000562

Bell JK, Askins J, Hall PR, Davies DR, Segal DM 2006. The dsRNA binding site of human Toll-like receptor 3. Proc Natl Acad Sci U S A 103: 8792-8797. doi: 10.1073/pnas.0603245103

Benjamini Y, Drai D, Elmer G, Kafkafi N, Golani I 2001. Controlling the false discovery rate in behavior genetics research. Behavioural Brain Research 125: 279-284. doi: 10.1016/s0166-4328(01)00297-2

Benton MA, et al. 2016. Toll genes have an ancestral role in axis elongation. Curr Biol 26: 1609-1615. doi: 10.1016/j.cub.2016.04.055

Boyd AC, et al. 2012. TLR15 is unique to avian and reptilian lineages and recognizes a yeast-derived agonist. J Immunol 189: 4930-4938. doi: 10.4049/jimmunol.1101790

Brennan JJ, Gilmore TD 2018. Evolutionary Origins of Toll-like Receptor Signaling. Mol Biol Evol 35: 1576-1587. doi: 10.1093/molbev/msy050

Brownlie R, et al. 2009. Chicken TLR21 acts as a functional homologue to mammalian TLR9 in the recognition of CpG oligodeoxynucleotides. Mol Immunol 46: 3163-3170. doi: 10.1016/j.molimm.2009.06.002

Carroll R. 2009. The Rise of Amphibians: 365 Million Years of Evolution: Johns Hopkins University Press.

Chen S, Cheng A, Wang M 2013. Innate sensing of viruses by pattern recognition receptors in birds. Vet Res 44: 82. doi: 10.1186/1297-9716-44-82

Chojnacki S, Cowley A, Lee J, Foix A, Lopez R 2017. Programmatic access to bioinformatics tools from EMBL-EBI update: 2017. Nucleic Acids Res 45: W550-W553. doi: 10.1093/nar/gkx273

Chuang T, Ulevitch RJ 2001. Identification of hTLR10: a novel human Toll-like receptor preferentially expressed in immune cells. Biochim Biophys Acta 1518: 157-161.

Dannemann M, Andres AM, Kelso J 2016. Introgression of Neandertal- and Denisovan-like haplotypes contributes to adaptive variation in human Toll-like receptors. Am J Hum Genet 98: 22-33. doi: 10.1016/j.ajhg.2015.11.015

Deschamps M, et al. 2016. Genomic Signatures of Selective Pressures and Introgression from Archaic Hominins at Human Innate Immunity Genes. Am J Hum Genet 98: 5-21. doi: 10.1016/j.ajhg.2015.11.014

Diebold SS, Kaisho T, Hemmi H, Akira S, Reis e Sousa C 2004. Innate antiviral responses by means of TLR7-mediated recognition of single-stranded RNA. Science 303: 1529-1531. doi: 10.1126/science.1093616

Edgar RC 2004. MUSCLE: multiple sequence alignment with high accuracy and high throughput. Nucleic Acids Res 32: 1792-1797. doi: 10.1093/nar/gkh340

Enard D, Depaulis F, Roest Crollius H 2010. Human and non-human primate genomes share hotspots of positive selection. PLoS Genet 6: e1000840. doi: 10.1371/journal.pgen.1000840

Fageras Bottcher M, et al. 2004. A TLR4 polymorphism is associated with asthma and reduced lipopolysaccharide-induced interleukin-12(p70) responses in Swedish children. J Allergy Clin Immunol 114: 561-567. doi: 10.1016/j.jaci.2004.04.050

Ferrer-Admetlla A, et al. 2008. Balancing selection is the main force shaping the evolution of innate immunity genes. J Immunol 181: 1315-1322. doi: 10.4049/jimmunol.181.2.1315

Gauthier MEA, Du Pasquier L, Degnan BM 2010. The genome of the sponge Amphimedon queenslandica provides new perspectives into the origin of Toll-like and interleukin 1 receptor pathways. Evol Dev 12: 519-533. doi: 10.1111/j.1525-142X.2010.00436.x

Gouy M, Guindon S, Gascuel O 2010. SeaView version 4: A multiplatform graphical user interface for sequence alignment and phylogenetic tree building. Mol Biol Evol 27: 221-224. doi: 10.1093/molbev/msp259

Grueber CE, Wallis GP, Jamieson IG 2014. Episodic positive selection in the evolution of avian toll-like receptor innate immunity genes. PLoS One 9: e89632. doi: 10.1371/journal.pone.0089632

Grueber CE, Wallis GP, King TM, Jamieson IG 2012. Variation at innate immunity Toll-like receptor genes in a bottlenecked population of a New Zealand robin. PLoS One 7: e45011. doi: 10.1371/journal.pone.0045011

Haas BJ, et al. 2013. De novo transcript sequence reconstruction from RNA-seq using the Trinity platform for reference generation and analysis. Nat Protoc 8: 1494-1512. doi: 10.1038/nprot.2013.084

Hayashi F, et al. 2001. The innate immune response to bacterial flagellin is mediated by Toll-like receptor 5. Nature 410: 1099-1103. doi: 10.1038/35074106

Heidari M, Fitzgerald SD, Zhang H 2015. Immune Responses in Cecal Tonsils of Marek's Disease Virus-Infected Chickens. Avian Dis 59: 213-226. doi: 10.1637/10950-093014-Reg.1

Heil F, et al. 2004. Species-specific recognition of single-stranded RNA via toll-like receptor 7 and 8. Science 303: 1526-1529. doi: 10.1126/science.1093620

Hemmi H, et al. 2000. A Toll-like receptor recognizes bacterial DNA. Nature 408: 740-745. doi: 10.1038/35047123

Hentschel U, Piel J, Degnan SM, Taylor MW 2012. Genomic insights into the marine sponge microbiome. Nature Reviews Microbiology 10: 641-U675. doi: 10.1038/nrmicro2839

Hibino T, et al. 2006. The immune gene repertoire encoded in the purple sea urchin genome. Dev Biol 300: 349-365. doi: 10.1016/j.ydbio.2006.08.065

Holland P, Dehal P, Boore JL 2005. Two Rounds of Whole Genome Duplication in the Ancestral Vertebrate. PLoS Biol 3: e314. doi: 10.1371/journal.pbio.0030314

Huang S, et al. 2008. Genomic analysis of the immune gene repertoire of amphioxus reveals extraordinary innate complexity and diversity. Genome Res 18: 1112-1126. doi: 10.1101/gr.069674.107

Hwang SD, Kondo H, Hirono I, Aoki T 2011. Molecular cloning and characterization of Toll-like receptor 14 in Japanese flounder, Paralichthys olivaceus. Fish Shellfish Immunol 30: 425-429. doi: 10.1016/j.fsi.2010.08.005

Jetz W, Fine PV 2012. Global gradients in vertebrate diversity predicted by historical area-productivity dynamics and contemporary environment. PLoS Biol 10: e1001292. doi: 10.1371/journal.pbio.1001292

Ji J, et al. 2018a. Characterization of the TLR Family in Branchiostoma lanceolatum and Discovery of a Novel TLR22-Like Involved in dsRNA Recognition in Amphioxus. Front Immunol 9: 2525. doi: 10.3389/fimmu.2018.02525

Ji J, Rao Y, Wan Q, Liao Z, Su J 2018b. Teleost-Specific TLR19 Localizes to Endosome, Recognizes dsRNA, Recruits TRIF, Triggers both IFN and NF-kappaB Pathways, and Protects Cells from Grass Carp Reovirus Infection. J Immunol 200: 573-585. doi: 10.4049/jimmunol.1701149

Jin MS, et al. 2007. Crystal structure of the TLR1-TLR2 heterodimer induced by binding of a tri-acylated lipopeptide. Cell 130: 1071-1082. doi: 10.1016/j.cell.2007.09.008

Junpee A, Tencomnao T, Sanprasert V, Nuchprayoon S 2010. Association between Toll-like receptor 2 (TLR2) polymorphisms and asymptomatic bancroftian filariasis. Parasitol Res 107: 807-816. doi: 10.1007/s00436-010-1932-9

Kawai T, Akira S 2010. The role of pattern-recognition receptors in innate immunity: update on Toll-like receptors. Nat Immunol 11: 373-384. doi: 10.1038/ni.1863

Key FM, Teixeira JC, de Filippo C, Andres AM 2014. Advantageous diversity maintained by balancing selection in humans. Curr Opin Genet Dev 29: 45-51. doi: 10.1016/j.gde.2014.08.001

Kiechl S, et al. 2002. Toll-like receptor 4 polymorphisms and atherogenesis. N Engl J Med 347: 185-192. doi: 10.1056/NEJMoa012673

Kim HM, et al. 2007. Crystal structure of the TLR4-MD-2 complex with bound endotoxin antagonist Eritoran. Cell 130: 906-917. doi: 10.1016/j.cell.2007.08.002

Kloch A, et al. 2018. Signatures of balancing selection in toll-like receptor (TLRs) genes - novel insights from a free-living rodent. Sci Rep 8: 8361. doi: 10.1038/s41598-018-26672-2

Koblansky AA, et al. 2013. Recognition of profilin by Toll-like receptor 12 is critical for host resistance to Toxoplasma gondii. Immunity 38: 119-130. doi: 10.1016/j.immuni.2012.09.016

Laayouni H, et al. 2014. Convergent evolution in European and Rroma populations reveals pressure exerted by plague on Toll-like receptors. Proc Natl Acad Sci U S A 111: 2668-2673. doi: 10.1073/pnas.1317723111

Lanfear R, Frandsen PB, Wright AM, Senfeld T, Calcott B 2017. PartitionFinder 2: New Methods for Selecting Partitioned Models of Evolution for Molecular and Morphological Phylogenetic Analyses. Mol Biol Evol 34: 772-773. doi: 10.1093/molbev/msw260

Letunic I, Bork P 2018. 20 years of the SMART protein domain annotation resource. Nucleic Acids Res 46: D493-D496. doi: 10.1093/nar/gkx922

Letunic I, Bork P 2007. Interactive Tree Of Life (iTOL): an online tool for phylogenetic tree display and annotation. Bioinformatics 23: 127-128. doi: 10.1093/bioinformatics/btl529

Leulier F, Lemaitre B 2008. Toll-like receptors--taking an evolutionary approach. Nat Rev Genet 9: 165-178. doi: 10.1038/nrg2303

Li XD, Chen ZJ 2012. Sequence specific detection of bacterial 23S ribosomal RNA by TLR13. Elife 1: e00102. doi: 10.7554/eLife.00102

Li Y, et al. 2018. Teleost-specific TLR25 identified from Schizothorax prenanti may recognize bacterial/viral components and activate NF-kappaB and type I IFNs signaling pathways. Fish Shellfish Immunol 82: 361-370. doi: 10.1016/j.fsi.2018.08.007

Lin SC, Lo YC, Wu H 2010. Helical assembly in the MyD88-IRAK4-IRAK2 complex in TLR/IL-1R signalling. Nature 465: 885-890. doi: 10.1038/nature09121

Liu G, et al. 2017. Characterization of the peripheral blood transcriptome and adaptive evolution of the MHC I and TLR gene families in the wolf (Canis lupus). BMC Genomics 18: 584. doi: 10.1186/s12864-017-3983-0

Liu L, et al. 2008. Structural basis of toll-like receptor 3 signaling with double-stranded RNA. Science 320: 379-381. doi: 10.1126/science.1155406

Luo R, et al. 2012. SOAPdenovo2: an empirically improved memory-efficient short-read de novo assembler. Gigascience 1: 18. doi: 10.1186/2047-217X-1-18

Mukherjee S, Huda S, Sinha Babu SP 2019. Toll-like receptor polymorphism in host immune response to infectious diseases: A review. Scand J Immunol: e12771. doi: 10.1111/sji.12771

Murrell B, et al. 2013. FUBAR: a fast, unconstrained bayesian approximation for inferring selection. Mol Biol Evol 30: 1196-1205. doi: 10.1093/molbev/mst030

Nielsen R, Yang Z 1998. Likelihood models for detecting positively selected amino acid sites and applications to the HIV-1 envelope gene. Genetics 148: 929-936.

O'Connor EA, Cornwallis CK, Hasselquist D, Nilsson JA, Westerdahl H 2018. The evolution of immunity in relation to colonization and migration. Nat Ecol Evol 2: 841-849. doi: 10.1038/s41559-018-0509-3

Offord V, Coffey TJ, Werling D 2010. LRRfinder: a web application for the identification of leucine-rich repeats and an integrative Toll-like receptor database. Dev Comp Immunol 34: 1035-1041. doi: 10.1016/j.dci.2010.05.004

Ohto U, et al. 2015. Structural basis of CpG and inhibitory DNA recognition by Toll-like receptor 9. Nature 520: 702-705. doi: 10.1038/nature14138

Oldenburg M, et al. 2012. TLR13 recognizes bacterial 23S rRNA devoid of erythromycin resistance-forming modification. Science 337: 1111-1115. doi: 10.1126/science.1220363

Ozinsky A, et al. 2000. The repertoire for pattern recognition of pathogens by the innate immune system is defined by cooperation between toll-like receptors. Proc Natl Acad Sci U S A 97: 13766-13771. doi: 10.1073/pnas.250476497

Park BS, et al. 2009. The structural basis of lipopolysaccharide recognition by the TLR4-MD-2 complex. Nature 458: 1191-1195. doi: 10.1038/nature07830

Pietretti D, et al. 2014. Identification and functional characterization of nonmammalian Toll-like receptor 20. Immunogenetics 66: 123-141. doi: 10.1007/s00251-013-0751-4

Pond SL, Frost SD, Muse SV 2005. HyPhy: hypothesis testing using phylogenies. Bioinformatics 21: 676-679. doi: 10.1093/bioinformatics/bti079

Quach H, et al. 2013. Different selective pressures shape the evolution of Toll-like receptors in human and African great ape populations. Hum Mol Genet 22: 4829-4840. doi: 10.1093/hmg/ddt335

Raetz M, et al. 2013. Cooperation of TLR12 and TLR11 in the IRF8-Dependent IL-12 Response to Toxoplasma gondii Profilin. The Journal of Immunology 191: 4818-4827. doi: 10.4049/jimmunol.1301301

Roach JC, et al. 2005. The evolution of vertebrate Toll-like receptors. Proc Natl Acad Sci U S A 102: 9577-9582. doi: 10.1073/pnas.0502272102

Shan S, et al. 2018. Non-mammalian Toll-like receptor 18 (Tlr18) recognizes bacterial pathogens in common carp (Cyprinus carpio L.): Indications for a role of participation in the NF-kappaB signaling pathway. Fish Shellfish Immunol 72: 187-198. doi: 10.1016/j.fsi.2017.09.081

Shen T, et al. 2012. Adaptive evolution and functional constraint at TLR4 during the secondary aquatic adaptation and diversification of cetaceans. BMC Evol Biol 12: 39. doi: 10.1186/1471-2148-12-39

Simakov O, et al. 2015. Hemichordate genomes and deuterostome origins. Nature 527: 459-465. doi: 10.1038/nature16150

Simakov O, et al. 2013. Insights into bilaterian evolution from three spiralian genomes. Nature 493: 526-531. doi: 10.1038/nature11696

Simoes BF, et al. 2016. Visual Pigments, Ocular Filters and the Evolution of Snake Vision. Mol Biol Evol 33: 2483-2495. doi: 10.1093/molbev/msw148

Song WS, Jeon YJ, Namgung B, Hong M, Yoon SI 2017. A conserved TLR5 binding and activation hot spot on flagellin. Sci Rep 7: 40878. doi: 10.1038/srep40878

Stamatakis A 2006. RAxML-VI-HPC: maximum likelihood-based phylogenetic analyses with thousands of taxa and mixed models. Bioinformatics 22: 2688-2690. doi: 10.1093/bioinformatics/btl446

Sutton TC 2018. The Pandemic Threat of Emerging H5 and H7 Avian Influenza Viruses. Viruses 10. doi: 10.3390/v10090461

Suyama M, Torrents D, Bork P 2006. PAL2NAL: robust conversion of protein sequence alignments into the corresponding codon alignments. Nucleic acids research 34: W609-W612. doi: 10.1093/nar/gkl315

Swanson WJ, Yang Z, Wolfner MF, Aquadro CF 2001. Positive Darwinian selection drives the evolution of several female reproductive proteins in mammals. Proc Natl Acad Sci U S A 98: 2509-2514. doi: 10.1073/pnas.051605998

Talavera G, Castresana J 2007. Improvement of phylogenies after removing divergent and ambiguously aligned blocks from protein sequence alignments. Syst Biol 56: 564-577. doi: 10.1080/10635150701472164

Tanji H, et al. 2015. Toll-like receptor 8 senses degradation products of single-stranded RNA. Nat Struct Mol Biol 22: 109-115. doi: 10.1038/nsmb.2943

Tassia MG, Whelan NV, Halanych KM 2017. Toll-like receptor pathway evolution in deuterostomes. Proc Natl Acad Sci U S A 114: 7055-7060. doi: 10.1073/pnas.1617722114

Travassos LH, et al. 2004. Toll-like receptor 2-dependent bacterial sensing does not occur via peptidoglycan recognition. EMBO Rep 5: 1000-1006. doi: 10.1038/sj.embor.7400248

Tsukada H, et al. 2005. Fish soluble Toll-like receptor 5 (TLR5S) is an acute-phase protein with integral flagellin-recognition activity. International Journal of Molecular Medicine 15: 519-525.

Velova H, Gutowska-Ding MW, Burt DW, Vinkler M 2018. Toll-like receptor evolution in birds: gene duplication, pseudogenisation and diversifying selection. Mol Biol Evol. doi: 10.1093/molbev/msy119

Wang J, Zhang Z, Liu J, Zhao J, Yin D 2016. Ectodomain Architecture Affects Sequence and Functional Evolution of Vertebrate Toll-like Receptors. Sci Rep 6: 26705. doi: 10.1038/srep26705

Wang Y, Liu L, Davies DR, Segal DM 2010. Dimerization of Toll-like receptor 3 (TLR3) is required for ligand binding. J Biol Chem 285: 36836-36841. doi: 10.1074/jbc.M110.167973

Wei T, et al. 2009. Homology modeling of human Toll-like receptors TLR7, 8, and 9 ligand-binding domains. Protein Sci 18: 1684-1691. doi: 10.1002/pro.186

Werling D, Jann OC, Offord V, Glass EJ, Coffey TJ 2009. Variation matters: TLR structure and species-specific pathogen recognition. Trends in Immunology 30: 124-130. doi: 10.1016/j.it.2008.12.001

Wiens M, et al. 2007. Toll-like receptors are part of the innate immune defense system of sponges (demospongiae: Porifera). Mol Biol Evol 24: 792-804. doi: 10.1093/molbev/msl208

William ED, Linda. 1994. Biology of Amphibians. Baltimore and London: The Johns Hopkins University Press.

Wlasiuk G, Nachman MW 2010. Adaptation and constraint at Toll-like receptors in primates. Mol Biol Evol 27: 2172-2186. doi: 10.1093/molbev/msq104

Yang J, et al. 2015. The I-TASSER Suite: protein structure and function prediction. Nat Methods 12: 7-8. doi: 10.1038/nmeth.3213

Yang Z, Wong WS, Nielsen R 2005. Bayes empirical bayes inference of amino acid sites under positive selection. Mol Biol Evol 22: 1107-1118. doi: 10.1093/molbev/msi097

Yang ZH 2007. PAML 4: Phylogenetic analysis by maximum likelihood. Molecular biology and evolution 24: 1586-1591. doi: 10.1093/molbev/msm088

Yarovinsky F, et al. 2005. TLR11 activation of dendritic cells by a protozoan profilin-like protein. Science 308: 1626-1629. doi: 10.1126/science.1109893

Yeh DW, et al. 2017. CpG-oligodeoxynucleotides developed for grouper toll-like receptor (TLR) 21s effectively activate mouse and human TLR9s mediated immune responses. Sci Rep 7: 17297. doi: 10.1038/s41598-017-17609-2

Zhang G, et al. 2014. Comparative genomics reveals insights into avian genome evolution and adaptation. Science 346: 1311-1320. doi: 10.1126/science.1251385

Zhang J, Nielsen R, Yang Z 2005. Evaluation of an improved branch-site likelihood method for detecting positive selection at the molecular level. Mol Biol Evol 22: 2472-2479. doi: 10.1093/molbev/msi237

Zhang Z, et al. 2016. Structural Analysis Reveals that Toll-like Receptor 7 Is a Dual Receptor for Guanosine and Single-Stranded RNA. Immunity 45: 737-748. doi: 10.1016/j.immuni.2016.09.011
