# Supplementary figures and images for: Evolutionary History of the Toll-Like Receptor Gene Family across Vertebrates
Source: Genome Biol Evol. 2019 Dec 4;12(1):3615–34. doi: 10.1093/gbe/evz266 (PMC6946030; doi:10.1093/gbe/evz266)

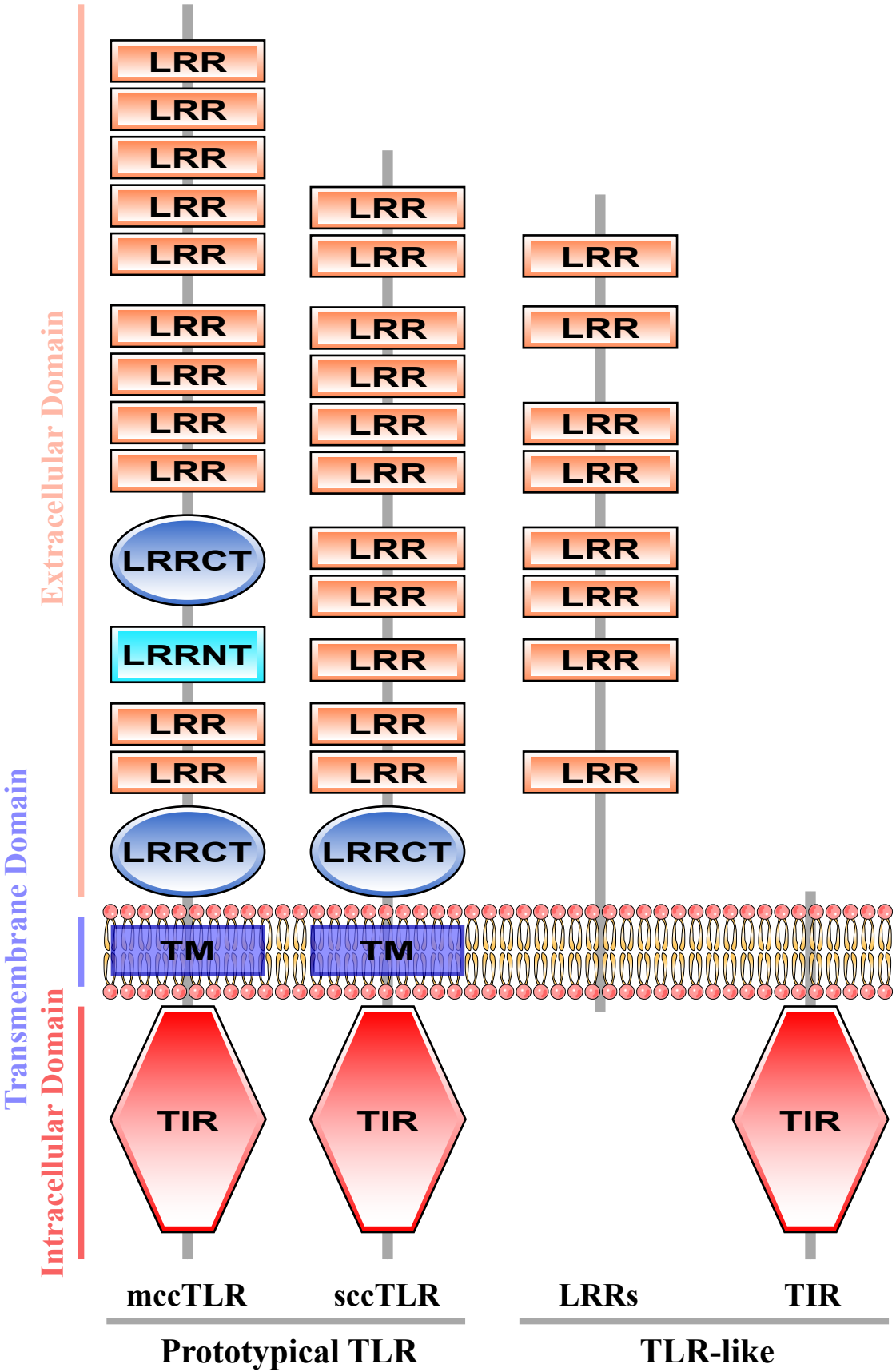

Supplement: evz266_Supplementary_Data [file evz266_supplementary_data.zip › Supplementary Figure S1.pdf]

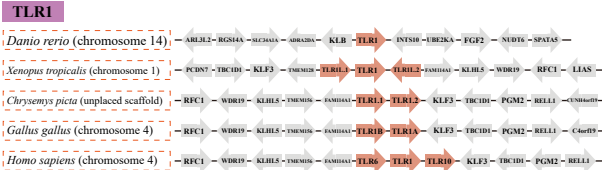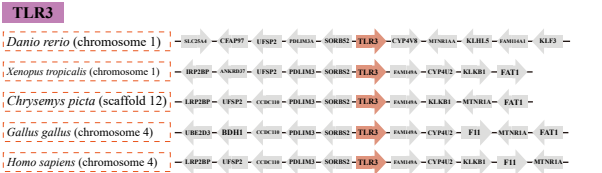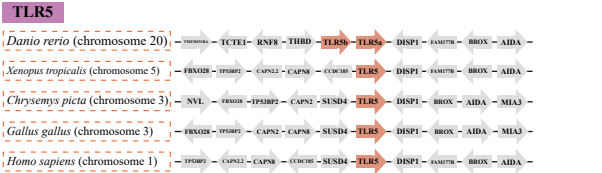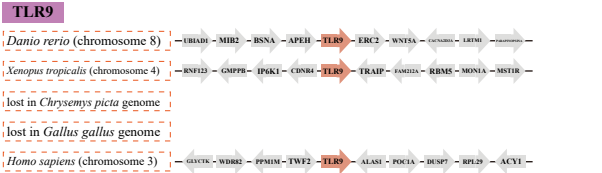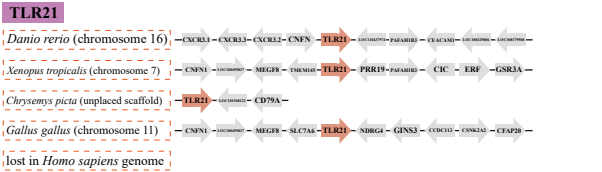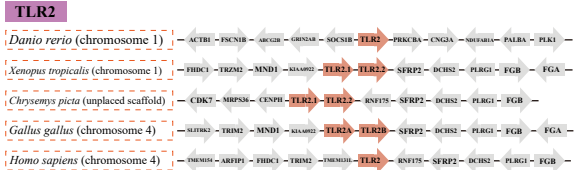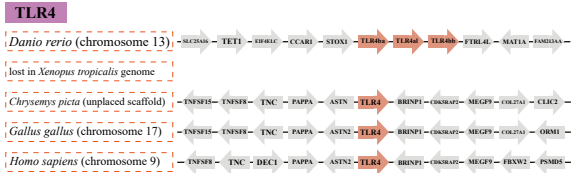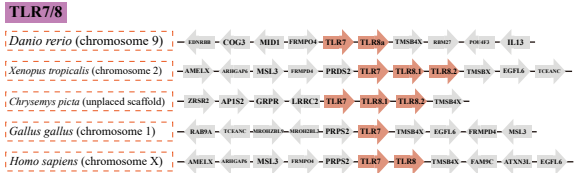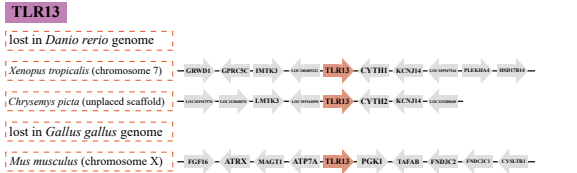

Supplement: evz266_Supplementary_Data [file evz266_supplementary_data.zip › Supplementary Figure S2.pdf]

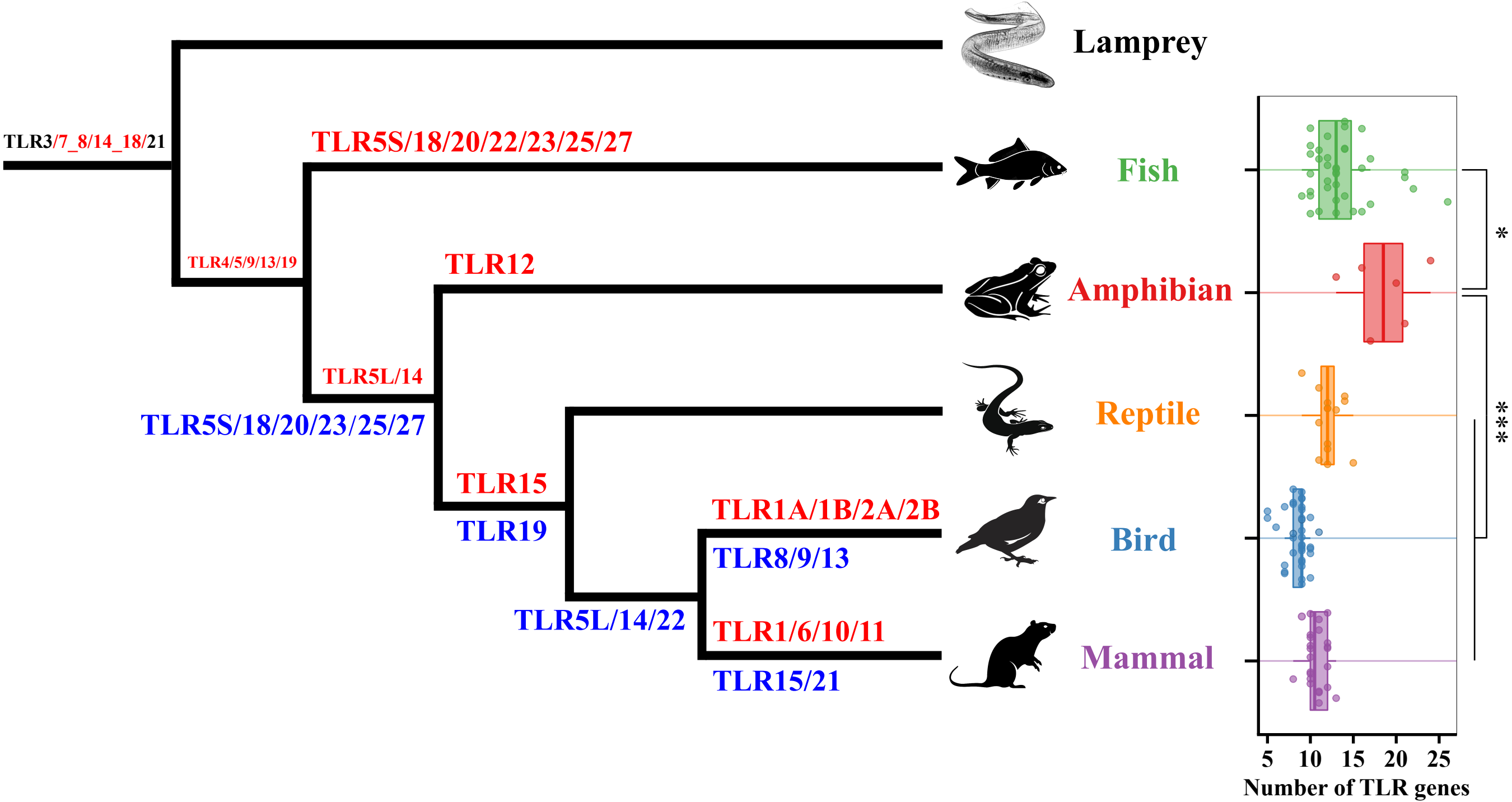

Supplement: evz266_Supplementary_Data [file evz266_supplementary_data.zip › Supplementary Figure S3.pdf]
